# Type 2 cannabinoid receptor expression on microglial cells regulates neuroinflammation during graft-versus-host disease

**DOI:** 10.1172/JCI175205

**Published:** 2024-04-25

**Authors:** Alison Moe, Aditya Rayasam, Garrett Sauber, Ravi K. Shah, Ashley Doherty, Cheng-Yin Yuan, Aniko Szabo, Bob M. Moore, Marco Colonna, Weiguo Cui, Julian Romero, Anthony E. Zamora, Cecilia J. Hillard, William R. Drobyski

**Affiliations:** 1 Department of Medicine,; 2 Department of Pharmacology, and; 3 Division of Biostatistics, Institute of Health and Equity, Medical College of Wisconsin, Milwaukee, Wisconsin, USA.; 4 College of Pharmacy, Department of Pharmaceutical Sciences, University of Tennessee Health Science Center, Memphis, Tennessee, USA.; 5 Department of Pathology and Immunology, Washington University, Saint Louis, Missouri, USA.; 6 Department of Pathology, Northwestern University, Chicago, Illinois, USA.; 7 Faculty of Experimental Sciences, Francisco de Vitoria University, Madrid, Spain.

**Keywords:** Neuroscience, Transplantation, Bone marrow transplantation, Immunotherapy

## Abstract

Neuroinflammation is a recognized complication of immunotherapeutic approaches such as immune checkpoint inhibitor treatment, chimeric antigen receptor therapy, and graft versus host disease (GVHD) occurring after allogeneic hematopoietic stem cell transplantation. While T cells and inflammatory cytokines play a role in this process, the precise interplay between the adaptive and innate arms of the immune system that propagates inflammation in the central nervous system remains incompletely understood. Using a murine model of GVHD, we demonstrate that type 2 cannabinoid receptor (CB2R) signaling plays a critical role in the pathophysiology of neuroinflammation. In these studies, we identify that CB2R expression on microglial cells induces an activated inflammatory phenotype that potentiates the accumulation of donor-derived proinflammatory T cells, regulates chemokine gene regulatory networks, and promotes neuronal cell death. Pharmacological targeting of this receptor with a brain penetrant CB2R inverse agonist/antagonist selectively reduces neuroinflammation without deleteriously affecting systemic GVHD severity. Thus, these findings delineate a therapeutically targetable neuroinflammatory pathway and have implications for the attenuation of neurotoxicity after GVHD and potentially other T cell–based immunotherapeutic approaches.

## Abstract 2

Type 2 cannabinoid receptor expression on microglial cells regulates neuroinflammation during graft-versus-host disease and can be therapeutically targeted using a brain penetrant CB2R inverse agonist/antagonist.

## Graphical abstract

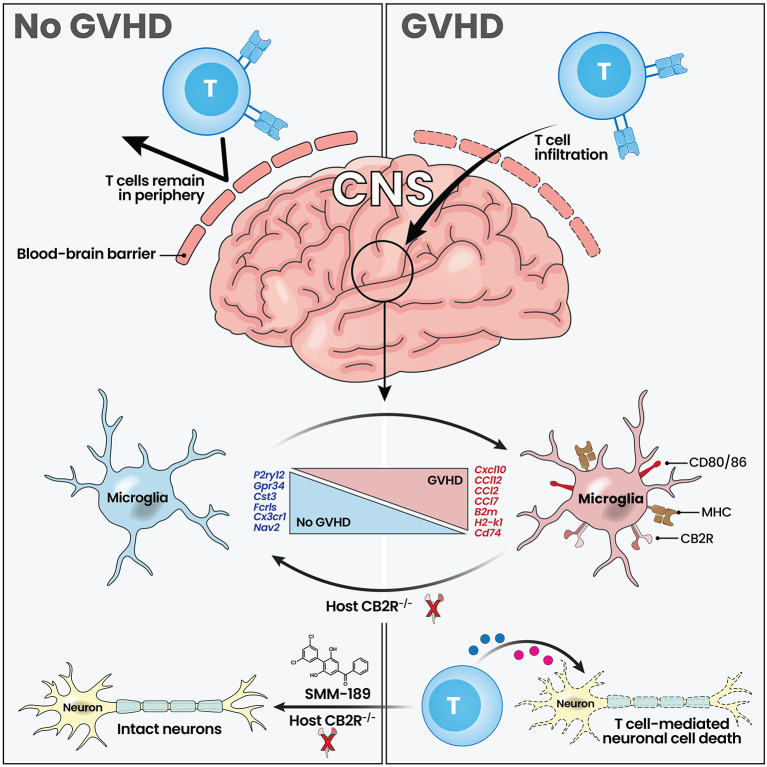

## Introduction

Neurological inflammation and cognitive dysfunction are increasingly recognized complications of cancer immunotherapeutic approaches such as immune checkpoint inhibitor treatment (1, 2), chimeric antigen receptor therapy (3, 4), and graft versus host disease (GVHD) occurring after allogeneic hematopoietic stem cell transplantation (5–9). Adverse neurological events that have been described include encephalitis, vasculitis, demyelinating disorders, aphasia, and seizures, all of which can lead to severe cognitive alterations and, in some instances, death (6–8, 10). The pathophysiology of this complication is thought to be due to the activation of T cells and subsequent release of inflammatory cytokines that promote pathological damage within the CNS (9, 11, 12). Notably, these T cell populations do not reside in the brain but are able to enter from the periphery under inflammatory conditions when there is breakdown of the blood brain barrier (13). Resident immune cells in the brain, specifically microglial cells, have also been implicated in this pathological process (11, 14), although the mechanistic pathways by which these cells contribute to neuroinflammation have not been completely delineated. Moreover, the mechanisms by which microglia, the primary innate immune cells of the brain, interact with nonresident T cells to promote this inflammatory milieu are not well understood.

Endocannabinoids are endogenously produced, bioactive lipids that are produced on demand and are present in all brain regions (15, 16). The 2 primary endocannabinoids are anandamide (*N*-arachidonoylethanolamine) (AEA) (17) and 2-arachidonoyl glycerol (2-AG) (18). Endocannabinoid effects are mediated by 2 G protein coupled receptors; the type 1 cannabinoid receptor (CB1R) (19) that is highly expressed in the CNS (20), and the type 2 cannabinoid receptor (CB2R) (21), which is predominantly expressed on immune cells (22). The CB1R plays a role in the regulation of synaptic activity at glutamatergic and GABAergic receptors through presynaptic inhibition of neurotransmitter release (23), whereas the CB2R has been shown to exert immunoregulatory effects on T cells, B cells, macrophages, and microglial cells (24–27). Thus, endocannabinoids have both neurological and immunological effects and, importantly, the CB2R is expressed on cells that constitute the innate and adaptive arms of the immune system, both of which have pathophysiological roles in immunotherapy-mediated neuroinflammation.

In the current report, we employed a murine model to examine the pathophysiology of GVHD-induced neuroinflammation. These studies demonstrated that endocannabinoid signaling through the CB2R plays a critical role in the regulation of inflammation within the CNS during this disease. Mechanistically, host expression of the CB2R potentiated inflammation in the brain by promoting the accumulation of proinflammatory donor T cells, augmenting microglial cell numbers and increasing neuronal cell death. Specifically, GVHD augmented CB2R expression on microglial cells resulting in an activated proinflammatory phenotype that was independent of interleukin 6 (IL-6) signaling blockade. Conversely, conditional deletion of the CB2R from microglia significantly reduced the accumulation of inflammatory T cells, decreased expression of genes associated with TNF signaling and chemotaxis, and attenuated neuronal cell death. In addition, pharmacological inhibition of this pathway with a brain penetrant CB2R inverse agonist/antagonist mitigated neuroinflammation without exacerbating systemic GVHD, indicating that this pathway is amenable to focused pharmacological intervention. Thus, these results define microglial cell expression of the CB2R as a critical regulator of GVHD-induced neuroinflammation.

## Results

### Donor T cells accumulate in all regions of the brain during GVHD and induce neuronal cell death.

**Figure 1:**
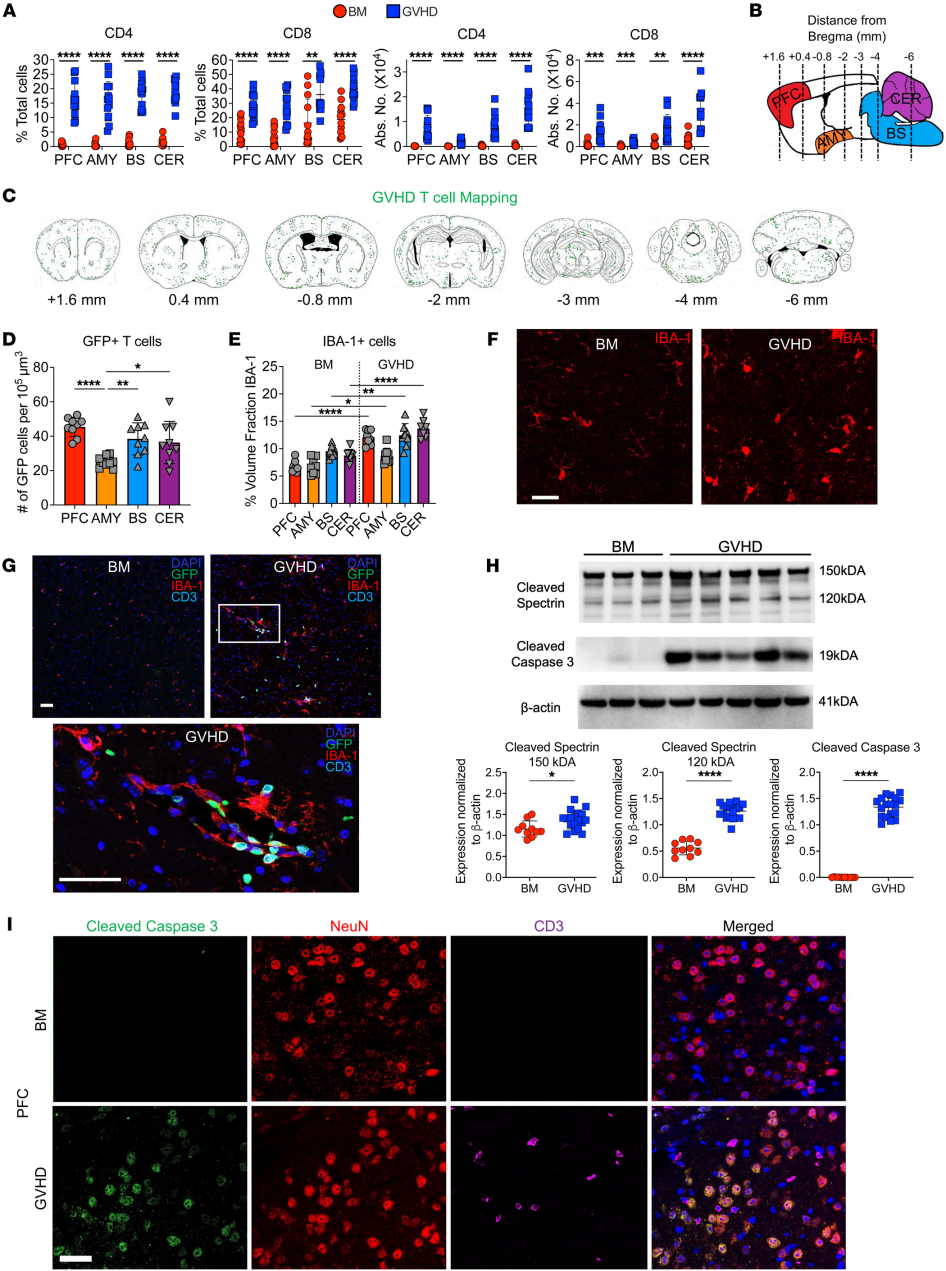
Donor T cells disseminate widely throughout the brain and induce neuronal cell death during GVHD. (**A**) Lethally irradiated Balb/c mice were transplanted with B6^EGFP^ BM alone or B6^EGFP^ BM and B6^EGFP^ spleen cells (adjusted to yield an αβ T cell dose of 0.75 × 10^6^). The percentage and absolute number of donor CD4^+^ and CD8^+^ T cells in the prefrontal cortex (PFC), amygdala (AMY), brainstem (BS), and cerebellum (CER) 14 days after transplantation. Results are from 3 experiments (*n* = 13 mice/group). (**B**–**G**) Balb/c mice were transplanted with B6 Rag-1 BM alone (BM) or with purified splenic B6^EGFP^ CD4^+^ (0.9 × 10^6^) and CD8^+^ (0.55 × 10^6^) T cells (GVHD). (**B**) Sagittal graphical representation of the brain depicting the location of specified regions along with the distance (in millimeters) from the bregma that selected coronal slices were examined to assess donor T cell infiltration. (**C**) Distribution of GFP-labeled T cells in specified coronal sections of GVHD mice 14 days after transplantation. (**D**) The number of GFP^+^ T cells in specified brain regions per 10^5^ um^3^. (**E** and **F**) Number of IBA-1^+^ cells in brain regions of GVHD animals depicted as the percentage of the area fraction (**E**) and representative immunofluorescence images (**F**). Scale bar: 30 μm. Results are from 2 experiments (*n* = 8 mice/group). (**G**) Representative immunofluorescence images depicting colocalization of GFP^+^ T cells and IBA-1^+^ cells in the PFC. Scale bar: 30 μm. (**H**). Representative Western blot images and scatterplots depicting normalized expression of cleaved spectrin (150 and 120 kDA) and cleaved caspase 3 in the brain from Balb/c mice transplanted with B6 BM or B6 BM and spleen cells. Results are from 2 experiments (*n* = 10–18 mice/group). (**I**) Representative immunofluorescence images showing expression of cleaved caspase 3, NeuN (neurons), and CD3 (T cells) along with merged compilation. Scale bar: 30 μm. Data are presented as mean ± SD and were analyzed using a *t* test with Welch’s correction. *P < 0.05, **P < 0.01, ***P < 0.001, ****P < 0.0001. Source data are provided as a Supporting Data Values file.

The clinical manifestations of neuroinflammation that develop during GVHD are varied and include a broad spectrum of cognitive and functional neurological deficits (6–8), suggesting that multiple areas of the brain can be affected. Using an established B6**→**Balb/c acute GVHD model, we transplanted recipients with β actin–driven GFP-labeled T cells and observed that CD4^+^ and CD8^+^ donor T cells were present in significantly higher frequency and absolute numbers in GVHD as opposed to BM control mice in the prefrontal cortex (PFC), amygdala, brainstem, and cerebellum (Figure 1A) which represent regions of the brain that are critical for the processing of higher cortical function, emotional inputs, regulation of key involuntary actions, and balance, respectively (28–31). Nearly all T cells in these recipients were donor-derived (Supplemental Figure 1A; supplemental material available online with this article; https://doi.org/10.1172/JCI175205DS1). In contrast, syngeneic marrow transplant recipients had a negligible number of donor T cells in these same brain regions, indicating virtually no accumulation in the absence of alloreactivity (Supplemental Figure 1B). Gene expression of proinflammatory cytokines in whole brain tissue was significantly increased in GVHD animals (Supplemental Figure 1C). In addition, we noted that the frequency and absolute number of donor CD4^+^ and CD8^+^ T cells were augmented (Supplemental Figure 1D) and these cells produced multiple inflammatory cytokines (i.e., IFN-γ, TNF-α, IL-6, and GM-CSF) (Supplemental Figure 1, E and F), indicative of generalized inflammation. Immunofluorescence staining of whole mounted brains from animals transplanted with GFP^+^ T cells confirmed the wide dissemination of these cells throughout white and gray matter in GVHD animals (Figure 1, B and C). To account for the existing size disparity of these examined regions, we performed a density-based analysis, which revealed similar numbers of T cells in equivalently demarcated areas of each region, except for the amygdala, which had relatively reduced T cell accumulation (Figure 1D). We also observed an increased density of IBA-1^+^ macrophage/microglial cells in the PFC, amygdala, brain stem, and cerebellum in GVHD mice when compared with BM controls (Figure 1E). Most IBA-1^+^ cells in BM control animals displayed a ramified morphology, indicative of a resting phenotype (32), whereas the morphology in GVHD mice was hyper-ramified and ameboid, indicative of a more activated phagocytic phenotype (33), particularly within the PFC (Figure 1F). Immunofluorescence staining demonstrated that there were adjacent GFP^+^ T cells and IBA-1^+^ cells in the brains of these mice (Figure 1G). To determine if this proinflammatory environment resulted in cellular damage, we performed Western blot analysis, which revealed increased cleaved spectrin and cleaved caspase 3 expression in the whole brains of GVHD compared with control animals (Figure 1H). The more significant increase in the 120 kDa compared with the 150 kDa cleaved spectrin fragment in GVHD mice was indicative of a more prominent role for apoptotic as opposed to necrotic cell death (34). Immunofluorescence with NeuN also revealed that caspase-positive cells were essentially all neuronal cells and depicted cleaved caspase 3 positive neurons in anatomic proximity to donor-derived T cells (Figure 1I). Finally, behavioral testing revealed that GVHD animals had a significant decrease in sociability, hedonic drive, stress coping, and motor function (Supplemental Figure 2). Collectively, these studies indicated that GVHD induced an inflammatory environment in the brain that was comprised of T cells, microglia/macrophages, and proinflammatory cytokines, which resulted in behavioral alterations and neuronal cell death.

### Microglial cells acquire an inflammatory transcriptional signature during GVHD.

**Figure 2:**
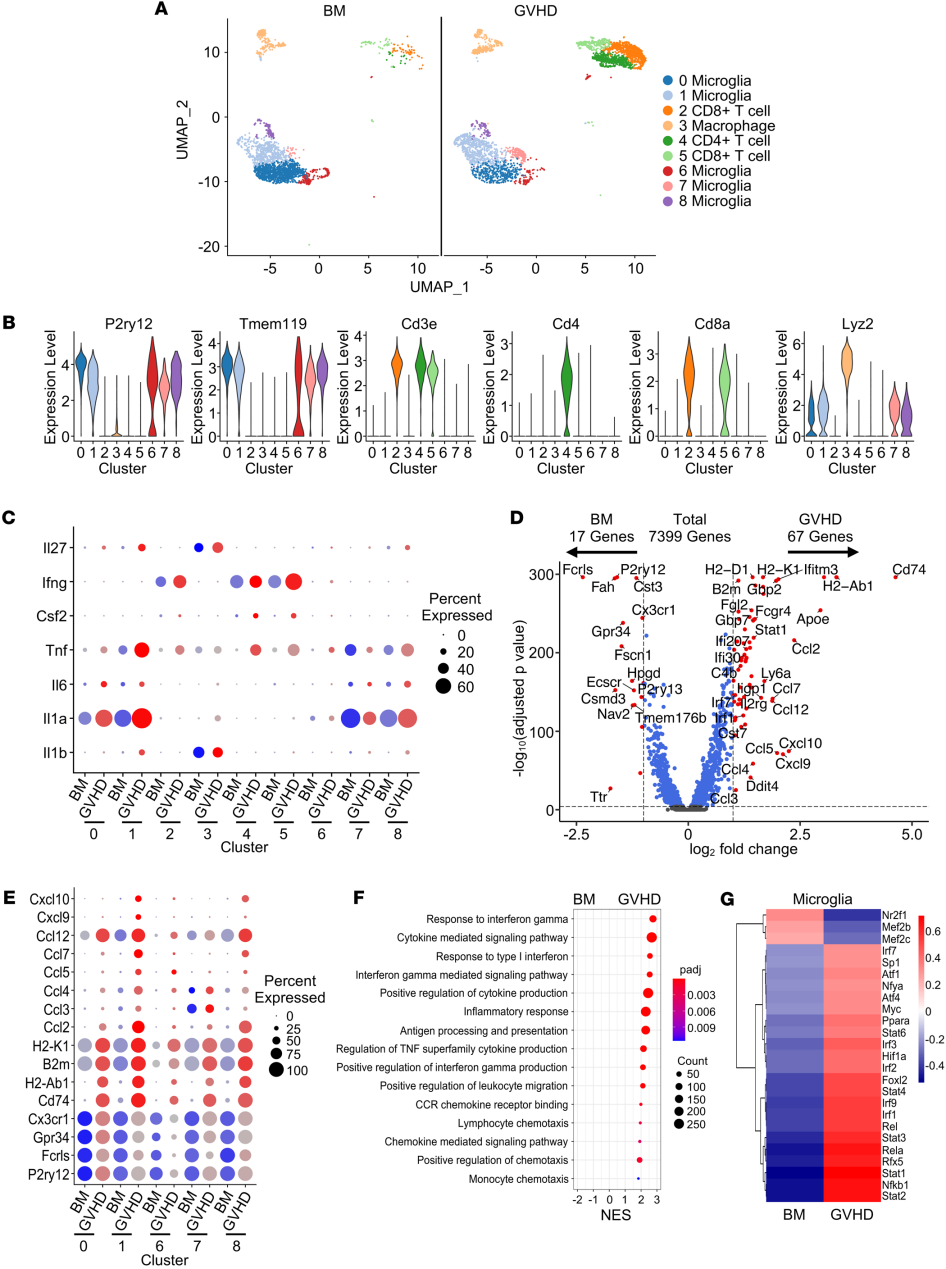
Microglial cells acquire an inflammatory transcriptional signature during GVHD. Lethally irradiated B6 mice were transplanted with B10.BR BM (5 × 10^6^) alone (BM) or together with B10.BR spleen cells (adjusted to yield an αβ T cell dose of 5 × 10^6^) (GVHD). Single live cells from pooled brains (*n* = 5/group) were sorted 14 days after transplantation. (**A**) Uniform manifold approximation and projection (UMAP) dimensional reduction of scRNAseq data of flow-sorted live cells from pooled brains. Unsupervised clustering using Seurat revealed 9 transcriptionally distinct clusters using a resolution of 0.5. (**B**) Violin plots showing log normalized expression of indicated microglia (*P2ry12* and *Tmem119*), T cell (*CD3*, *CD4*, and *CD8*) and macrophage markers (*Lyz2*). (**C**) Bubble plots depicting inflammatory cytokine profile in each cluster. (**D**) Volcano plot showing over/underrepresented genes in aggregated microglial clusters from BM versus GVHD mice. Cutoff parameters were |log_2_(FC)| > 1.0 and *P*_adj_ < 0.0001. (**E**) Bubble plots depicting microglia-specific, MHC class I and II, and chemokine gene expression in each microglial cell cluster. (**F**) Bubble plot demonstrating normalized enrichment score (NES) for pathways identified using the GO database. (**G**) Heatmap showing binary regulon activity of the top 25 regulons that were differentially expressed in microglial cell clusters from BM versus GVHD animals. In all bubble plots, the size of the dot represents the percent of cells that express a given transcript, whereas the intensity of the color represents the average expression of a given gene within the cells of that cluster. Source data are provided as a Supporting Data Values file.

To further delineate the inflammatory environment in the brain, we employed a B10.BR**→**B6 GVHD model and performed single-cell RNA sequencing (scRNAseq) analysis on immune cells isolated from the brains of BM and GVHD mice. This analysis revealed 9 transcriptionally distinct clusters that represented CD8^+^ T cells (2 clusters), CD4^+^ T cells (1 cluster), macrophages (*Lyz2*; 1 cluster), and microglia (*P2ry12/Tmem119*; 5 clusters), which constituted the largest set of clusters (Figure 2, A and B). The majority of analyzed cells consisted of microglia and T cells, with only a small percentage (approximately 10%) consisting of macrophages (Supplemental Figure 3A). T cells represented 44% of all cells in GVHD mice, but only 5% in BM control animals. T cells were donor-derived in both groups in this GVHD model (Supplemental Figure 3B). Transcriptional analysis of inflammatory cytokines in the brain revealed that CD4^+^ and CD8^+^ T cells primarily produced IFN-γ, TNF-α, and GM-CSF (clusters 2, 4 and 5), macrophages produced IL-1β and IL-27 (cluster 3), and microglia produced IL-1α, TNF-α, and, to a lesser extent, IL-6 (clusters 0, 1, 6, 7 and 8) (Figure 2C). There was no detectable expression of IL-10, IL-12, IL-17A, IL-22, or IL-23 in any of these cell types (data not shown). The inflammatory cytokine transcripts identified in T cells were consistent with cytokine protein expression that was observed by flow cytometry (Supplemental Figure 1, E and F). T cells from GVHD mice had increased expression of *Icos*, *Stat1*, *Il12rb2*, *Ly6c*2, and *Ly6a*, indicative of an activated memory phenotype (Supplemental Figure 3C and Supplemental Table 1), whereas the single macrophage cluster from GVHD mice demonstrated increased expression of complement (*C1qa* and *C1qb*), chemokine (*Ccl5*, *Cxcl9*, and *Cxcl10*), and S100a genes (*S100a8* and *S100a11*), which all are associated with inflammatory pathways (Supplemental Figure 3D and Supplemental Table 2).

Since microglia constituted the majority of total immune and identified clusters, we performed a more detailed transcriptome analysis focused on the 5 microglial cell subsets. This revealed that there were 84 differentially expressed genes with 17 over expressed in BM and 67 over expressed in GVHD mice based on defined cutoff criteria (log_2_ FC) > 1.0 and *P*_adj_ < 0.0001, full list available in Supplemental Table 3), representing approximately 1.1% of the total sequenced transcriptome (Figure 2D). Microglial cells from BM control animals had increased expression of genes that are characteristic of a homeostatic phenotype (i.e., *Cx3cr1*, *Gpr34*, *Fcrls*, and *P2ry12*) (35) (Figure 2E). In contrast, microglia from GVHD mice exhibited increased expression of major histocompatibility complex class I and II MHC genes (i.e., H2-*K1*, *B2m*, *CD74*, and *H2-Ab1*), as well as chemokine genes (i.e., *Ccl2*, *Ccl3*, *Ccl4*, *Ccl5*, *Ccl7*, *Ccl12*, *Cxcl9*, and *Cxcl10*), indicative of an activated inflammatory phenotype (Figure 2E). This phenotype was most prominent in clusters 0, 1, and 8, which constituted the majority of microglial cells in the brain (Supplemental Figure 3A). Gene set enrichment analysis (GSEA) using the Gene Ontology (GO) data set confirmed that microglia from GVHD mice had increased expression of genes associated with inflammatory pathways (e.g., cytokine mediated signaling pathway, response to IFN-γ, antigen processing and presentation, and TNF superfamily cytokine production) and chemotaxis (i.e., chemokine receptor binding, positive regulation of chemokine production, and leukocyte chemotaxis) (Figure 2F). The activation and differentiation of microglia is driven by the expression of transcription factors and downstream target genes, which constitute a gene regulatory network (i.e., regulons) and can be interrogated using DoRothEA (Discriminant Regulon Expression Analysis), which is a computational method for gene regulatory network construction of scRNAseq data (36). Using this approach, we observed that microglia from GVHD animals had enriched regulon activity for Stat (i.e., *Stat1*, *Stat2*, *Stat3*, *Stat4*, and *Stat6*), NF-κB (i.e., *Nfkb1*, *Rela*, and *Rel*), and IFN regulatory factor (i.e., *Irf1*, *Irf2*, *Irf3*, *Irf7*, and *Irf9*) family transcription factors (Figure 2G), which are all constituents of regulatory networks that mediate inflammation (37). Thus, GVHD induced a transcriptionally coordinated, inflammatory microglial phenotype that was characterized by prominent expression of chemokine genes.

### Microglial cells regulate neuronal cell death during GVHD.

**Figure 3:**
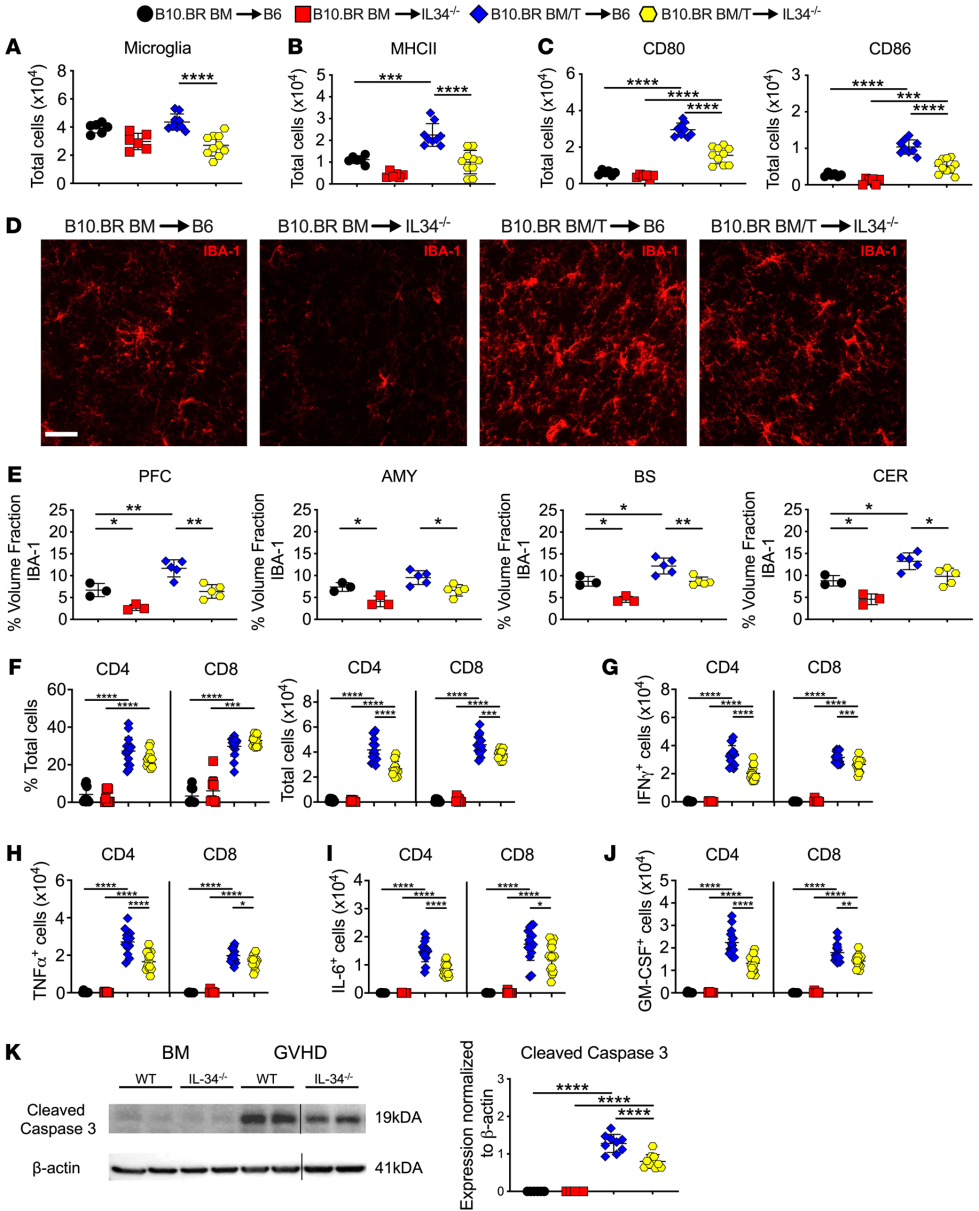
Microglial cells regulate neuronal cell death. (**A**–**K**) Lethally irradiated (1,000 cGy) B6 or IL-34^–/–^ mice were transplanted with B10.BR BM alone or together with B10.BR spleen cells (adjusted to yield an αβ T cell dose of 4 × 10^6^ T cells). (**A**) Absolute number of microglial cells as defined by expression of CD45^lo^ CD11b^+^. (**B** and **C**) Absolute number of MHC class II, CD80 and CD86 expressing microglial cells. Analysis of microglial cells was performed by flow cytometry. Results are from 2 experiments (*n* = 6–10 mice/group). (**D**) Representative immunofluorescence images of IBA-1^+^ cells in the PFC. Scale bar: 30 μm. (**E**) Quantification of IBA-1^+^ cells in the PFC, amygdala, brainstem, and cerebellum (*n* = 3–5 mice/group). (**F**–**J**) The frequency and absolute number of donor-derived CD4^+^ and CD8^+^ T cells and the absolute number of CD4^+^ and CD8^+^ T cells that produced IFN-γ, TNF-α, IL-6, or GM-CSF in the brains of mice 14 days after transplantation. Analysis of T cells was performed by flow cytometry. Data are from 3 experiments (*n* = 8–15 mice/group). (**K**) Representative Western blot images and scatter plots depicting normalized expression of cleaved caspase 3 from B6 or IL-34^–/–^ mice transplanted with B10.BR BM alone or together with B10.BR spleen cells. Results are from 2 experiments (*n* = 5–10 mice/group). Vertical lines on Western blots denote noncontiguous gel lanes. Data are presented as mean ± SD and were analyzed using a 1-way ANOVA with Tukey’s test for multiple group comparisons. *P < 0.05, **P < 0.01, ***P < 0.001, ****P < 0.0001. Source data are provided as a Supporting Data Values file.

The acquisition of an inflammatory microglial transcriptional profile led us to examine the functional role of these cells in promoting neuroinflammation within the CNS. To address this question, we employed a B10.BR**→**B6 GVHD model and employed IL-34^–/–^ (B6 background) mice as recipients, since the maintenance of microglial cell homeostasis is dependent upon interactions between the endogenous ligands CSF-1 and IL-34 with their cognate receptor CSF-1R (38, 39), and the genetic absence of IL-34 results in a significant reduction in microglial cell numbers (40). Consistent with these prior reports, the absence of IL-34 expression in B10.BR**→**B6 recipient mice resulted in a significant reduction in the number of microglia compared with WT GVHD animals (Figure 3A). This was accompanied by a reduction in the absolute number of microglia (CD45^lo^ CD11b^+^) that expressed MHC class II (Figure 3B) and the costimulatory molecules CD80 and CD86 (Figure 3C). Immunofluorescence confirmed that there was a significant reduction in IBA-1^+^ microglia in the brains of IL-34^–/–^ versus WT GVHD animals (Figure 3, D and E). Correspondingly, we observed that there was no decrease in the frequency of donor-derived CD4^+^ or CD8^+^ T cells, but that there was a significant reduction in the absolute number of these T cell populations (Figure 3F) as well as the total number of CD4^+^ and CD8^+^ T cells that produced inflammatory cytokines (i.e., IFN-γ, TNF-α, IL-6, and GM-CSF) (Figure 3, G–J). Notably, there was also a decrease in neuronal cell death in IL-34^–/–^ GVHD recipients as evidenced by reduced cleaved caspase 3 protein expression (Figure 3K). Collectively, these results provide evidence that microglial cells promote the accumulation of proinflammatory T cells into the CNS and the induction of neuronal cell death during GVHD.

### Host expression of the CB2R drives neuroinflammation during GVHD.

Given the inflammatory milieu and ensuing neuronal cell death driven by T cells and microglia, we sought to uncover mechanistic pathways that coordinately regulated these cell populations in the development of GVHD-induced neuroinflammation. We hypothesized that signaling through the CB2R expressed on immune cell populations might play an important pathophysiological role, since the CB2R has been shown to regulate both adaptive and innate immune responses during systemic GVHD (26) and our data indicate that both arms of the immune system are involved in neuroinflammation. To test this hypothesis, we first examined the role of CB2R expression on donor immune cells in CNS inflammation by transplanting recipients with marrow grafts from either WT or CB2R^–/–^ donors. We observed that there was no difference in expression of inflammatory cytokines in whole brains of animals reconstituted with marrow grafts from WT versus CB2R^–/–^ mice (Supplemental Figure 4A). Given the requirement for donor T cells in the induction of GVHD-induced neuroinflammation, we examined the role of CB2R expression on donor T cells and noted that absence of CB2R expression resulted in a significant decrease in the percentage and absolute number of donor-derived CD4^+^, but not CD8^+^, T cells in GVHD mice (Supplemental Figure 4B). There were also reduced numbers of CD4^+^ T cells that produced IFN-γ, TNF-α, and GM-CSF, whereas the total number of CD8^+^ T cells that expressed these cytokines was not different between WT and CB2R^–/–^ groups (Supplemental Figure 4, C–F). No difference was observed in the absolute number of microglia (Supplemental Figure 4G) or microglia expressing MHC class II, CD80, or CD86 (Supplemental Figure 4, H and I). Notably, cleaved caspase 3 levels were not significantly different between animals in these 2 groups (Supplemental Figure 4J), indicating that absence of the CB2R on donor T cells reduced the number of proinflammatory CD4^+^ T cells but had no effect on the accumulation of inflammatory CD8^+^ T cells and did not prevent neuronal cell death.

**Figure 4:**
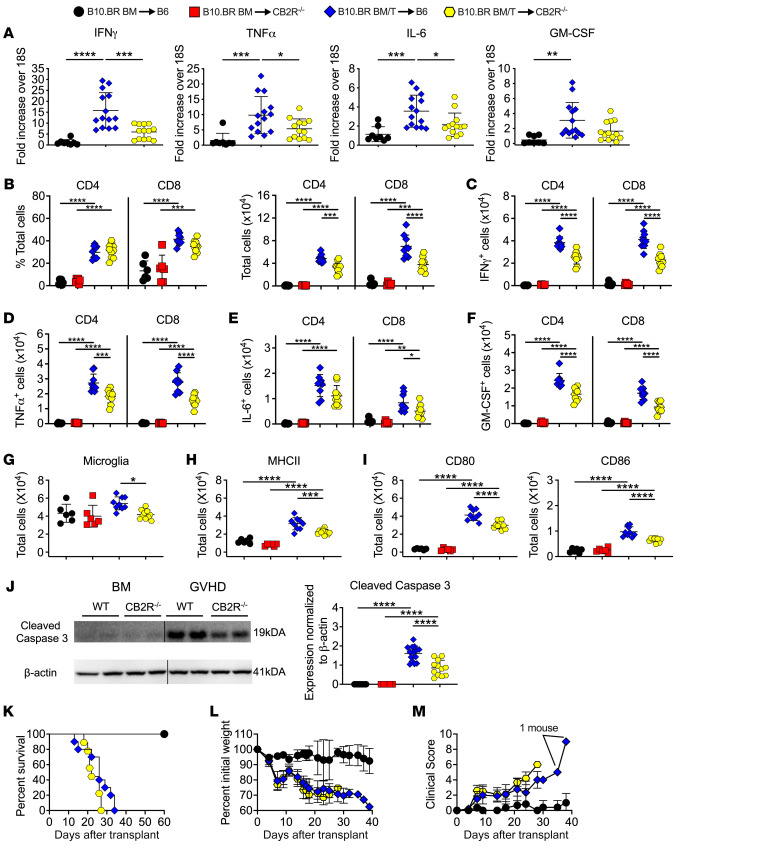
Host CB2R expression regulates neuroinflammation in the brain. Lethally irradiated (1,100 cGy) B6 or CB2R^–/–^ mice were transplanted with B10.BR BM (5 × 10^6^) together with B10.BR spleen cells (adjusted to yield a αβ T cell dose of 4.5–5 × 10^6^ T cells). B6 animals transplanted with B10.BR BM served as controls. (**A**) IFN-γ, TNF-α, IL-6, and GM-CSF mRNA expression is depicted in whole brain. Results are from 3 experiments (*n* = 8–14 mice/group). (**B**–**J**) Lethally irradiated B6 or CB2R^–/–^ mice were transplanted with B10.BR BM (5 × 10^6^) alone or together with B10.BR spleen cells (adjusted to yield a T cell dose of 4.5–5 × 10^6^ T cells). (**B**) The percentage and absolute number of donor-derived CD4^+^ and CD8^+^ T cells in the brains of mice 14 days after transplantation. (**C**–**F**) The absolute number of CD4^+^ and CD8^+^ T cells that produced IFN-γ, TNF-α, IL-6, or GM-CSF. (**G**) Absolute number of microglial cells. (**H** and **I**) Absolute number of MHC class II, CD80, and CD86 expressing microglial cells. Data in panels **B**–**I** are from 2 experiments (*n* = 6–10 mice/group). (**J**) Representative Western blot images and scatter plots depicting normalized expression of cleaved caspase 3 in the brain from B6 or CB2R^–/–^ mice transplanted with B10.BR BM and spleen cells. Vertical lines on Western blots denote noncontiguous gel lanes. Results are from 2 experiments (*n* = 8–14 mice/group). (**K**–**M**) Lethally irradiated B6 or CB2R^–/–^ animals were transplanted with B10.BR BM and spleen cells. B6 mice transplanted with B10.BR BM alone served as controls. Overall survival (panel **K**), serial weight curves (panel **L**), and clinical score (panel **M**) are shown. Results are from 2 experiments (*n* = 6–10 mice/group). Data are presented as mean ± SD and were analyzed using a 1-way ANOVA with Tukey’s test for multiple group comparisons. *P < 0.05, **P < 0.01, ***P < 0.001, ****P < 0.0001. Source data are provided as a Supporting Data Values file.

In contrast, when recipient mice lacked CB2R expression, we observed a significant reduction in gene expression of IFN-γ, IL-6, and TNF-α in the brain (Figure 4A). There was also a decrease in the absolute number of donor-derived CD4^+^ and CD8^+^ T cells (Figure 4B) as well as CD4^+^ and CD8^+^ T cells that produced IFN-γ, IL-6 (CD8 only), TNF-α, and GM-CSF in the brains of CB2R^–/–^ versus WT recipients (Figure 4, C–F). This was accompanied by a significant reduction in the total number of microglial cells (Figure 4G) as well as the absolute number of microglia expressing MHC class II, CD80, and CD86 (Figures 4, H and I). Correspondingly, we noted decreased expression of cleaved caspase 3 in the brains of CB2R^–/–^ recipient mice (Figures 4J), indicating that host CB2R expression potentiated neuronal cell death. Of note, there was no increase in overall GVHD lethality in CB2R^–/–^ recipients (Figure 4K) nor any differences in weight loss or clinical score when compared with WT GVHD controls (Figure 4, L and M), demonstrating that absence of CB2R in the host did not exacerbate systemic GVHD, unlike what has been reported after transplantation with donor CB2R^–/–^ immune cell populations (26). Thus, these studies revealed that absence of recipient CB2R expression resulted in decreased accumulation of proinflammatory donor T cells, reduced numbers of microglia with an activated phenotype, and significantly diminished neuronal cell death in the brain.

### A brain penetrant, but not peripherally restricted, CB2R inverse agonist/antagonist attenuates inflammation in the brain.

Since GVHD is a systemic disease, inflammation is simultaneously induced in the periphery and in the CNS where host CB2R-expressing cells both reside. Therefore, to define the location of the recipient CB2R^+^ cell population that was most critical for driving neuroinflammation, as well as to determine if the CB2R signaling pathway could be therapeutically targeted, we pursued a pharmacological strategy in which mice were treated with either a peripherally restricted (SR144528) or brain penetrant (SMM-189) CB2R inverse agonist/antagonist. SR144528 has been reported to not be distributed into the CNS (41) and to be an optimal tool for in vivo murine studies due to its high selectivity profile for the receptor (42). To corroborate that SR144528 did not enter the CNS, we treated mice with SR144528 and performed isotope-dilution mass spectrometric analysis to quantitate and compare the concentration of SR144528 in the blood versus the brain of transplant recipients (Supplemental Figure 5). We observed that SR144528 (Supplemental Figure 6A) was measurable in the serum with mean concentrations of 150–250 pg/μl with no differences between naive, BM controls or GVHD recipients (Supplemental Figure 6B). In contrast, while SR144528 was detectable in the brain (mean 5–10 pg/mg), concentrations were significantly lower (15–50-fold) compared with serum (Supplemental Figure 6C), indicating that very little SR144528 distributes to the CNS. To assess the functional effects of CB2R signaling blockade with this agent, we treated mice daily for 14 days and observed that there was a reduced frequency of CD4^+^ T cells but no difference in the absolute number of CD4^+^ or CD8^+^ T cells in the brains of animals treated with SR144528 versus a vehicle control (Supplemental Figure 6D). Moreover, we noted no significant difference in the total number of CD4^+^ and CD8^+^ T cells that produced IFN-γ, TNF-α, or IL-6 in CB2R antagonist-treated animals (Supplemental Figure 6, E–G). Only CD4^+^ and CD8^+^ T cells that produced GM-CSF were found to be decreased in animals that were treated with SR144528 (Supplemental Figure 6H). In addition, administration of SR144528 had no effect on cleaved caspase 3 expression levels (Supplemental Figure 6I) when compared with vehicle-treated mice. Thus, these studies indicated that pharmacological blockade with a peripherally restricted CB2R inverse agonist/antagonist had no substantive effect on mitigating neuroinflammation or preventing neuronal cell death in GVHD mice.

**Figure 5:**
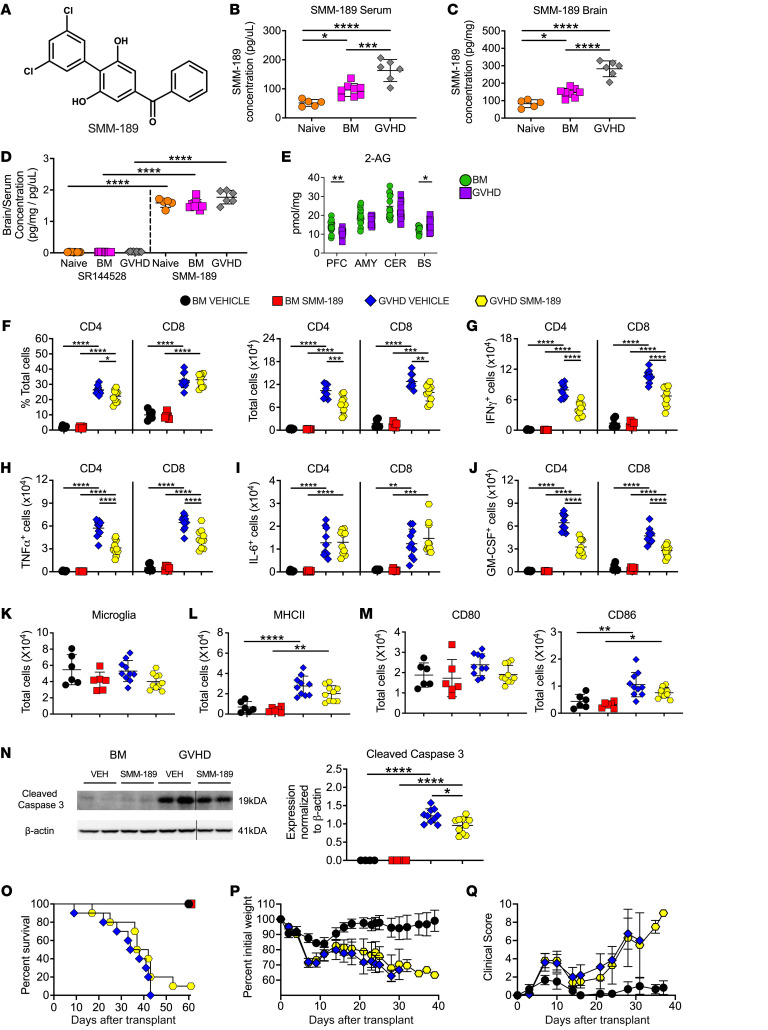
Pharmacological administration of a brain-penetrant CB2R inverse agonist/antagonist reduces inflammation in the CNS during GVHD. (**A**) Chemical structure of SMM-189. (**B** and **C**) Serum level (**B**) and brain concentration (**C**) of SMM-189 in naïve and Balb/c mice transplanted with B6 BM or B6 BM and spleen cells. (**D**) Ratio of brain to serum SR144528 and SMM-189 concentrations. Results in **B**–**D** are from 2 experiments (*n* = 5–10 mice/group). (**E**) 2-AG levels in the amygdala, brainstem, cerebellum, and prefrontal cortex 14 days after transplantation. Results are from 3 experiments (*n* = 14–15 mice/group). (**F**–**N**) Balb/c recipients were transplanted with B6 BM alone or B6 BM and spleen cells. Animals were then treated with SMM-189 or vehicle control. (**F**) The percentage and absolute number of donor CD4^+^ and CD8^+^ T cells in the brains of mice 14 days after transplantation. (**G**–**J**) The absolute number of CD4^+^ and CD8^+^ T cells that produced IFN-γ, TNF-α, IL-6, or GM-CSF. (**K**) Absolute number of microglial cells. (**L** and **M**) Absolute number of MHC class II, CD80, and CD86 expressing microglial cells. Results in panels **F**–**M** are from 2 experiments (*n* = 6–10 mice/group). (**N**) Representative Western blot images and scatterplots depicting normalized expression of cleaved caspase 3 in the brain from mice treated with either SMM-189 or a vehicle control. Vertical lines on Western blots denote noncontiguous gel lanes. Data are from 2 experiments (*n* = 4–10 mice/group). (**O**–**Q**) Balb/c recipients were transplanted with B6 BM alone (*n* = 6) or with B6 spleen cells (*n* = 10) and treated with SMM-189 or vehicle. Overall survival (**O**), serial weight curves (**P**), and clinical score (**Q**) are shown. Results are from 2 experiments (*n* = 6–10 mice/group). In panels **P** and **Q**, BM alone mice only received vehicle. Data are presented as mean ± SD. Statistics were performed using a 1-way ANOVA with Tukey’s test for multiple group comparisons. *P < 0.05, **P < 0.01, ***P < 0.001, ****P < 0.0001. Source data are provided as a Supporting Data Values file.

To then determine whether host CB2R-expressing cells within the brain played a critical pathophysiological role in promoting neuroinflammation, we examined the efficacy of SMM-189, which was synthesized in one of our labs (B.M. II). SMM-189 has a structurally unique triaryl core (43) (Figure 5A), is specific for the CB2R (43, 44), and has been shown to reduce inflammation in murine models of traumatic brain injury (45), suggesting that this agent distributes into the brain. To confirm this premise, we performed isotope-dilution mass spectrometric analysis to measure SMM-189 concentrations in serum and the brain (Supplemental Figure 7). We observed that GVHD mice had significantly higher concentrations of SMM-189 than BM control or naive animals in both the serum (Figure 5B) and the brain (Figure 5C). In addition, these studies revealed that SMM-189 accumulates in the brain in all 3 groups with a brain/serum ratio of greater than 1, whereas the ratio of brain-to-serum SR144528 concentration averaged only 0.02–0.03 (Figure 5D). Since SMM-189 competes with 2-AG, the natural ligand for the CB2R (15), we measured 2-AG levels in specified regions of the brain to ascertain whether GVHD altered levels of this endocannabinoid. These studies demonstrated some discordant regional variation in the brainstem and PFC, but, overall, no evidence that GVHD uniformly altered 2-AG levels (Figure 5E). Subsequent administration of SMM-189 resulted in a significant reduction in the frequency of CD4^+^ T cells, the absolute number of total CD4^+^ and CD8^+^ T cells (Figure 5F), and the total number of CD4^+^ and CD8^+^ T cells that produced IFN-γ, TNF-α, and GM-CSF (Figure 5, G–J). Treatment with SMM-189 had no effect on the absolute number of microglia (Figure 5K) or the total number of microglia with an activated phenotype (i.e., expressing MHC class II, CD80, or CD86) (Figure 5, L and M), but did result in a significant decrease in the expression of cleaved caspase 3 (Figure 5N), indicative of reduced neuronal cell death. In addition, whereas administration of SR144258 exacerbated GVHD lethality (26), treatment with SMM-189 had no deleterious effect on overall survival, weight loss, or clinical score (Figure 5, O–Q), demonstrating that CNS-directed blockade of CB2R signaling with SMM-189 selectively ameliorated neuroinflammation. Collectively, these results demonstrated that pharmacological blockade with a brain penetrant CB2R inverse agonist/antagonist attenuated GVHD-induced neuroinflammation and provided evidence that CB2R expression on a CNS-resident population was critical for regulating inflammation in this tissue site.

### CB2R expression is increased on microglial cells during GVHD and is not regulated by IL-6.

**Figure 6:**
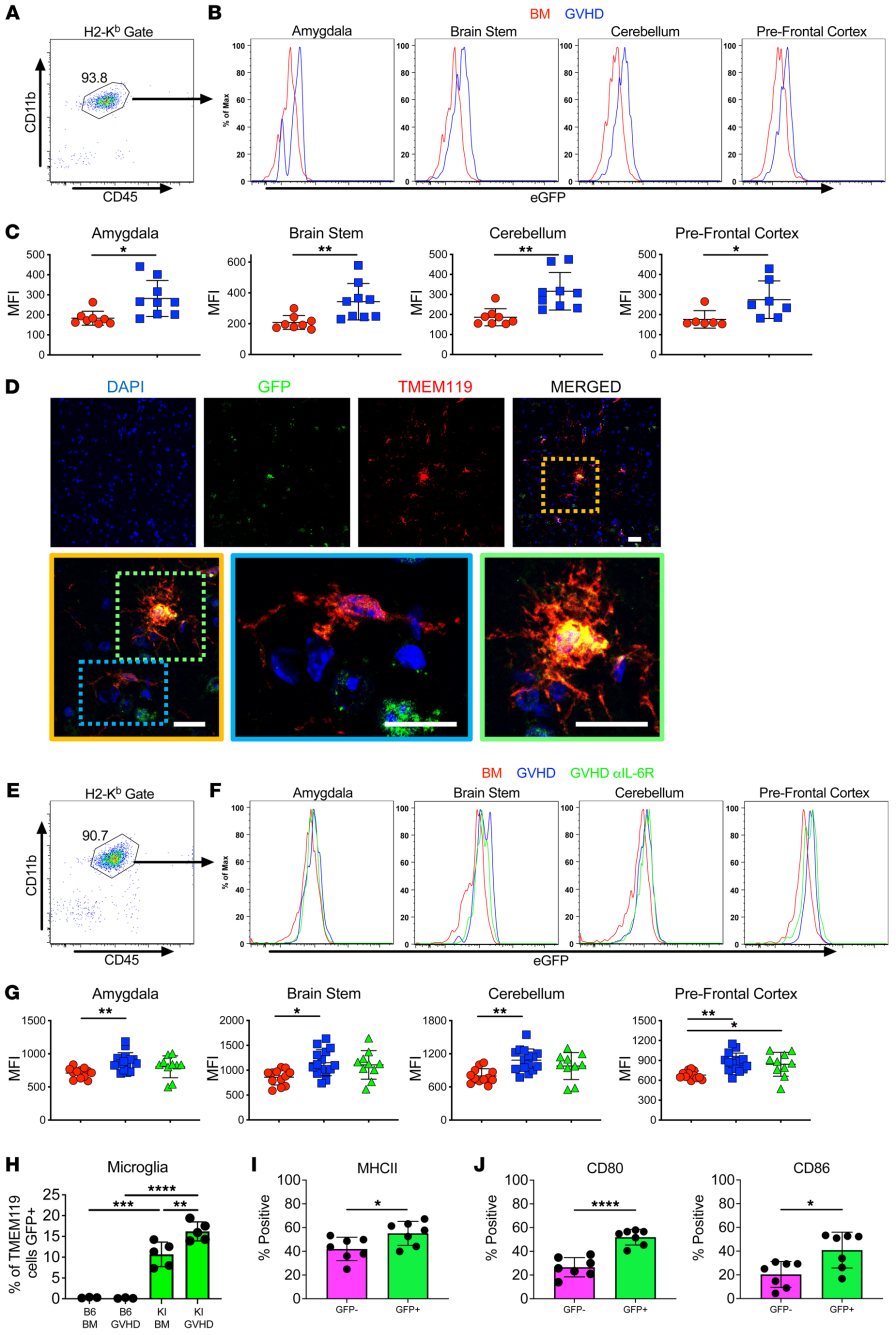
Microglial cell expression of the CB2R induces an activated phenotype and is not regulated by IL-6 signaling. (**A**–**C**) CB2R^EGFP+^ mice were transplanted with B10.BR BM alone or with B10.BR spleen cells. Animals were euthanized 14 days after transplantation. (**A**) Representative dot plot depicting recipient H-2K^b+^ CD45^+^ CD11b^+^ microglial cells. (**B**) Representative histograms depicting EGFP expression on recipient microglial cells from the amygdala, brain stem, cerebellum, and prefrontal cortex of CB2R^EGFP^ animals reconstituted with B10.BR BM (BM, red line) or B10.BR BM and spleen cells (GVHD, blue line). (**C**) Scatterplot showing cumulative median fluorescence intensity (MFI) shifts from 4 replicate experiments. Each data point represents pooled results from 2 mice (*n* = 8–9 data points). (**D**) Representative immunofluorescence staining showing CB2R (GFP), microglia (TMEM119), and merged images from the prefrontal cortex of CB2R^EGFP+^ mice transplanted with B10.BR BM and spleen cells. Magnified yellow insert box depicts microglial cell that is CB2R^–^ (blue box) and 1 that expresses the CB2R (green box). Scale bars: 10 μm. (**E**–**G**) CB2R^EGFP+^ mice were transplanted with B10.BR BM alone or with B10.BR spleen cells and treated with an anti-IL-6R or isotype control antibody. (**E**) Representative dot plot depicting recipient H-2K^b+^ CD45^+^ CD11b^+^ microglial cells. (**F**) Representative histograms depicting EGFP (CB2R) expression on microglial cells obtained from specified brain regions of CB2R^EGFP^ animals reconstituted with B10.BR BM only (BM, red line) or with B10.BR BM and spleen cells and treated with an isotype (GVHD, blue line) or anti-IL-6R antibody (GVHD, αIL-6R, green line). (**G**) Scatterplot data showing cumulative MFI shifts. Each data point represents pooled results from 2 mice. Results are from 5 experiments (*n* = 10–15 data points). (**H**–**J**) CB2R^EGFP+^ (KI) or B6 mice were transplanted with B10.BR BM alone or with B10.BR spleen cells. Percentage of microglial cells expressing CB2R (EGFP) (**H**), and frequency of GFP^+^ (CB2R^+^) and GFP^–^ (CB2R^–^) microglia expressing MHC class II, CD80, and CD86 (**I** and **J**). Results are from 2 experiments (*n* = 3–7 mice/group). Data are presented as mean ± SD. Statistics were performed using a *t* test with Welch’s correction for pairwise comparisons and a 1-way ANOVA with Tukey’s test for multiple group comparisons. *P < 0.05, **P < 0.01, ***P < 0.001, ****P < 0.0001. Source data are provided as a Supporting Data Values file.

To define hematopoietically derived recipient CB2R^+^ cells in the brain of GVHD mice, we gated on H-2K^b+^ CD45^+^ cells and observed that virtually all were CD45^lo^ CD11b^+^, indicative of a microglial cell phenotype (Figure 6A). Employing CB2R^EGFP^ reporter mice (46), we noted increased expression of the CB2R on microglia in the amygdala, brainstem, cerebellum, and prefrontal cortex in GVHD animals compared with BM controls (Figures 6B and 6C), indicating that microglial cell expression of the CB2R was increased throughout the CNS under inflammatory conditions. This was further confirmed by immunofluorescence that demonstrated colocalization of GFP and TMEM119 expression in microglia from GVHD-recipient CB2R^EGFP^ reporter mice (Figure 6D). Blockade of IL-6 signaling, which has been shown to be ineffectual for the prevention and treatment of neuroinflammation occurring as a complication of immunotherapy in humans (47) had no effect on CB2R expression (Figure 6, E–G), demonstrating that microglial expression of CB2R was not regulated by IL-6. A more quantitative assessment revealed that the CB2R was expressed on only a small percentage (approximately 15%) of all microglia in GVHD animals (Figure 6H). Notably, the percentage of CB2R^+^ microglial cells that expressed MHC class II, CD80, and CD86 was significantly higher than CB2R nonexpressing microglial cells (Figure 6, I and J), indicating that CB2R expression was associated with an activated microglial phenotype.

### CB2R expression on microglial cells regulates proinflammatory T cells and neuronal cell death in the brain.

**Figure 7:**
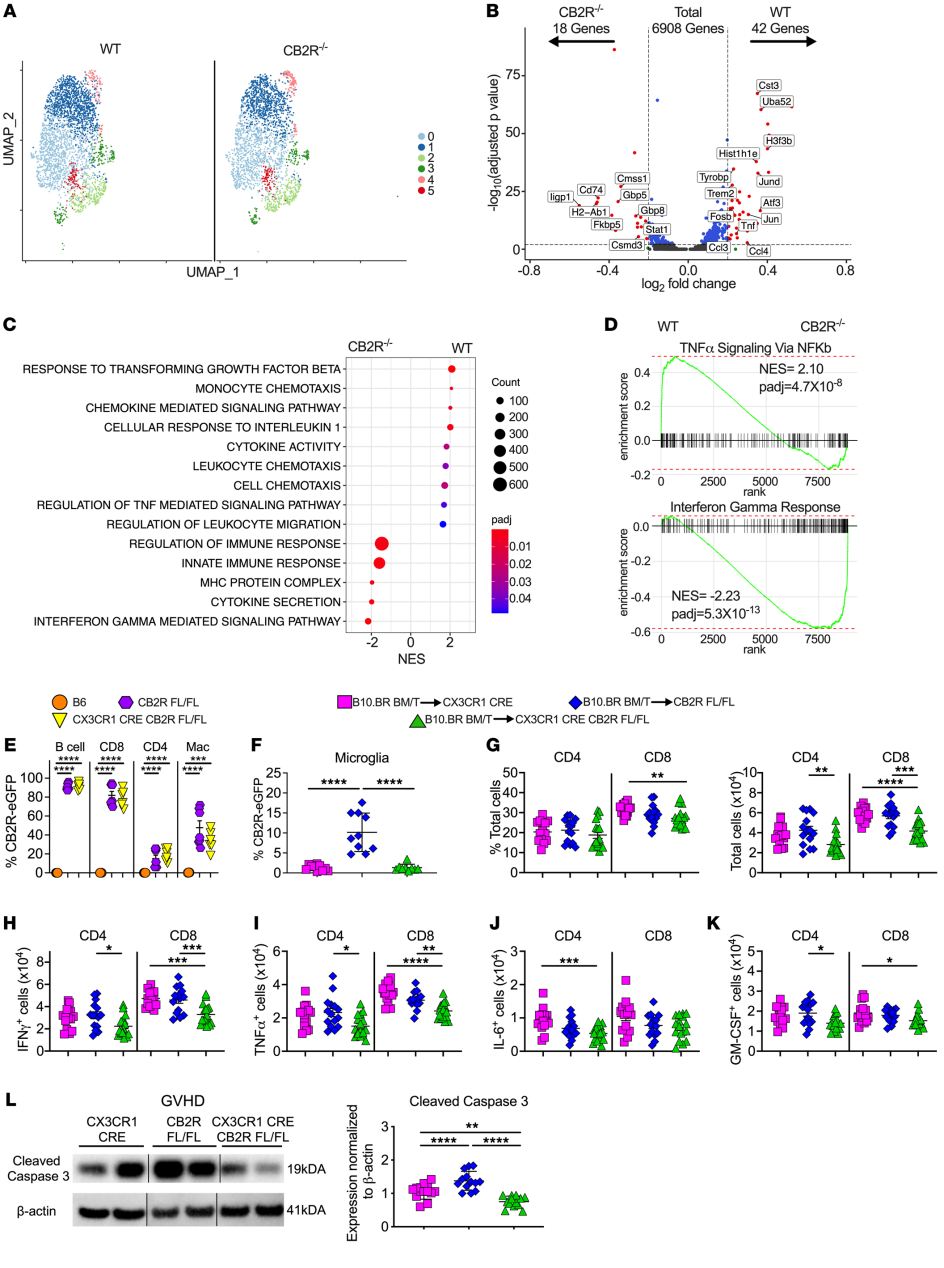
Conditional deletion of CB2R in microglial cells attenuates proinflammatory T cell accumulation and neuronal cell death. (**A**–**D**) B6 or CB2R^–/–^ mice were transplanted with B10.BR BM and spleen cells. Single live microglial cells from pooled brains (*n* = 5/group) were sorted 14 days after transplantation. (**A**) UMAP dimensional reduction of scRNAseq data of flow-sorted live cells from pooled brains. Unsupervised clustering using Seurat revealed 6 transcriptionally distinct clusters using a resolution of 0.3. (**B**) Volcano plot showing over/underrepresented genes in aggregated microglial clusters from B6 versus CB2R^–/–^ mice. Cutoff parameters were |log_2_(FC)| > 0.2 and *P*_adj_ < 0.01. (**C**) Bubble plot demonstrating normalized enrichment score (NES) for pathways identified using the GO database. (**D**) GSEA using Hallmark database comparing expression of TNF-α signaling and IFN-response genes in microglial cells from B6 versus CB2R^–/–^ mice. (**E**) Percentage of CB2R expression on splenic B cells, CD8^+^ T cells, CD4^+^ T cells, and macrophages in naive, CB2R^EGFP^ ^fl/fl^ or CX3CR1-Cre CB2R^EGFP^ ^fl/fl^ mice. Data are from 2 experiments (*n* = 6–7 mice/group). (**F**–**L**). CX3CR1-Cre, CB2R^fl/fl^ or CX3CR1-Cre CB2R^fl/fl^ mice were transplanted with B10.BR BM and spleen cells. Mice were euthanized 14 days after transplantation. (**F**) The percentage of microglial cells that expressed the CB2R based on eGFP expression. Results are from 2 experiments (*n* = 9–10 mice/group). (**G**) The percentage and absolute number of donor CD4^+^ and CD8^+^ T cells in the brain. (**H**–**K**) The absolute number of CD4^+^ and CD8^+^ T cells that produced IFN-γ, TNF-α, IL-6, or GM-CSF. (**L**) Representative Western blot images and summary data depicting normalized expression of cleaved caspase 3. Results are from 3 experiments in panels **G**–**L** (*n* = 13–17 mice/group). Vertical lines on Western blots denote noncontiguous gel lanes. Data are presented as mean ± SD and were analyzed using a 1-way ANOVA with Tukey’s test for multiple group comparisons. *P < 0.05, **P < 0.01, ***P < 0.001, ****P < 0.0001. Source data are provided as a Supporting Data Values file.

To further delineate the effect of CB2R expression on microglia and determine whether these cells had a more inflammatory signature, we performed scRNAseq analysis on sorted microglial cells obtained from the brains of WT versus CB2R^–/–^ GVHD recipient mice. This analysis revealed 6 transcriptionally distinct microglial clusters of which 2 were dominant (clusters 0 and 1) (Figure 7A). Two clusters that identified as T cells and macrophages, representing 1% of all cells, were deemed to be sort contaminants and were excluded from the analysis. Transcriptional analysis of these 6 clusters revealed 60 differentially expressed genes with 42 overexpressed in microglia from WT and 18 overexpressed in microglia from CB2R^–/–^ animals based on defined cutoff criteria (log_2_ FC) > 0.2 and *P*_adj_ < 0.01, full list available in Supplemental Table 4), representing approximately 0.9% of the total sequenced transcriptome (Figure 7B). Microglial cells from WT mice had increased expression of inflammatory mediators such as *CCl3*, *CCl4*, and *TNF*, whereas CB2R^–/–^ microglial cells had increased expression of genes associated with IFN-γ signaling such as the guanylate-binding proteins (*Gbp5* and *Gbp8)* (48) and *Iigp1* (49). To uncover biologically relevant pathways, we employed GSEA using the GO database, which revealed increased expression of genes associated with TNF-α signaling, TGF-β responsiveness, leukocyte chemotaxis, and chemokine signaling in sorted WT microglia, whereas there was augmented expression of IFN-γ response genes in CB2R^–/–^ microglia (Figure 7C). Similarly, GSEA using Hallmark gene annotation confirmed increased expression of genes associated with TNF-α signaling and IFN-γ response genes in WT and CB2R^–/–^ microglia, respectively (Figure 7D). To determine whether CB2R expression on microglial cells directly regulated neuroinflammation, we generated CX3CR1-Cre CB2R^fl/fl^ mice in which CB2R is deleted from microglial cells, which express the fractalkine receptor CX3CR1 (50), allowing microglia to be genetically targeted (51–53). Examination of CB2R expression on spleen cells from normal CX3CR1-Cre CB2R^fl/fl^ mice revealed that Cre-mediated deletion had no effect on B cells, T cells, or macrophages (Figure 7E). In contrast, there was an 87% average reduction in CB2R expression on microglia from CX3CR1-Cre CB2R^fl/fl^ GVHD mice compared with CB2R^fl/fl^ controls (Figure 7F), indicative of effective Cre-mediated recombination. To define the role of microglial CB2R expression, recipient CX3CR1-Cre, CB2R^fl/fl^, or CX3CR1-Cre CB2R^fl/fl^ animals were transplanted with MHC-mismatched BM and splenocytes from B10.BR mice. These studies revealed that there was a significant reduction in the absolute number of CD8^+^ T cells in the brains of recipient CX3CR1-Cre CB2R^fl/fl^ animals compared with CX3CR1-Cre and CB2R^fl/fl^ control mice (Figure 7G). In addition, we observed a significant decrease in the absolute number of CD8^+^ T cells that produced IFN-γ and TNF-α (Figure 7, H–K). There was also a corresponding reduction in cleaved caspase 3 expression in CX3CR1-Cre CB2R^fl/fl^ recipients (Figure 7L). Thus, microglial cell expression of the CB2R regulated TNF-α signaling and chemotaxis/chemokine signaling gene pathways, promoted the accumulation of proinflammatory CD8^+^ T cells, and augmented neuronal cell death in the brain during GVHD.

## Discussion

Neuroinflammation in the brain is a recognized complication of immunotherapeutic approaches, such as allogeneic HSCT (5–9), immune checkpoint inhibitor treatment (1, 2, 54), and chimeric antigen receptor therapy (3, 4) all of which are increasingly being used to treat patients with underlying malignant conditions. A distinguishing characteristic of this pathophysiological process is that the inciting immunological event, which is largely driven by T cells, occurs in the periphery but subsequently extends into the CNS due to T cells that cross the blood-brain barrier and interact with resident immune cells in the brain (50). The mechanistic pathways by which cells of the adaptive and innate arms of the immune system intersect to promote a neuroinflammatory milieu in the CNS, however, have not been well delineated. Herein, using a murine model of GVHD, we have identified a critical role for CB2R signaling in the pathophysiology of immune-mediated CNS inflammation. Our studies indicate that host, but not donor, expression of the CB2R promotes the accumulation of proinflammatory T cells, increases microglial cell numbers, and induces neuronal cell death in the brain. Mechanistically, we show that microglial cell expression of the CB2R in the context of GVHD is associated with an activated phenotype characterized by increased expression of MHC class II and costimulatory molecules. Conversely, cell-specific deletion of the CB2R from microglia decreased the accumulation of inflammatory T cells, reduced expression of inflammatory and chemokine signaling gene pathways, and attenuated neuronal cell death. Further, we demonstrate that targeting the CB2R with a brain penetrant, CB2R-specific inverse agonist/antagonist mitigated neuroinflammation without exacerbating systemic GVHD-induced lethality, indicating that this signaling pathway is amenable to CNS-directed pharmacological intervention.

Microglial cells have numerous functions that include phagocytosis, antigen presentation capabilities, and the production of inflammatory cytokines and chemokines (35, 55), indicative of their role as a primary regulator of innate immunity in the CNS. During GVHD, we observed that these cells acquired an activated, inflammatory phenotype, characterized by a transcriptional profile that revealed increased expression of MHC class I and II genes, along with a wide array of chemokine genes (i.e., *Ccl2*, *Ccl3*, *Ccl4*, *Ccl5*, *Ccl7*, *Ccl12*, *Cxcl9*, and *Cxcl10*), which function to recruit monocytes, T cells, and other immune cells into the brain. This contrasted with the profile of microglia from BM control animals, which maintained a more homeostatic phenotype (i.e., *P2ry12*, *Gpr34*, *Fscn1*, *Cx3CR1*, and *Fcrls*). Further, applying DoRothEA analysis to the scRNAseq data set, microglia from GVHD animals had increased expression of Stat, NF-KB, and IRF family transcription factors that have been shown to be pathways by which microglia promote inflammation (56–58). The maintenance of microglial cell homeostasis is dependent upon interactions between the endogenous ligands CSF-1 and IL-34 with their cognate receptor, CSF-1R (38, 39), and the genetic absence of IL-34 results in a profound reduction in microglial cell numbers (40). To formally define a role for microglia in mediating neuroinflammation, we employed IL-34^–/–^ mice as transplant recipients and demonstrated that these animals had a significant reduction in the absolute number of activated microglial cells. This reduction was associated with a commensurate reduction in proinflammatory cytokine–producing CD4^+^ and CD8^+^ T cells as well as a decrease in cleaved caspase 3 expression, indicative of reduced neuronal cell death. Thus, these data indicated that microglial cells play a critical role in mediating neuroinflammation during GVHD.

The critical roles that T cells and microglia have in mediating GVHD-associated neuroinflammation prompted us to examine the role of the type 2 cannabinoid receptor, since this signaling pathway is known to regulate inflammatory cytokine production by T cells (25, 27), and the receptor is also expressed on microglia (59). To address this question within the context of a murine GVHD model in which there are bidirectional immune responses, we first examined the role of donor CB2R expression and observed that absence of this receptor had very little effect on CNS inflammation. Whereas transplantation with CB2R^–/–^ marrow grafts resulted in a reduction in proinflammatory CD4^+^ T cells, there was no effect on CD8^+^ T cells nor did mice have any decrease in neuronal cell death, as assessed by cleaved caspase 3 expression. Interestingly, earlier studies in which animals were transplanted with donor CB2R^–/–^ marrow grafts (26) had demonstrated increased GVHD-induced lethality due to augmented systemic inflammation, indicative of discordant effects in the CNS versus periphery. In contrast, absence of host CB2R expression significantly reduced GVHD-induced neuroinflammation, as evidenced by a decrease in the accumulation of proinflammatory, donor-derived CD4^+^ and CD8^+^ T cells, a reduced number of microglial cells that possessed an activated phenotype, and significantly less neuronal cell death. In addition, CB2R^–/–^ recipient mice had no increase in GVHD-induced mortality, indicating that absence of recipient, in contrast to donor, CB2R expression did not result in discordant inflammatory responses in the periphery and the brain. Thus, recipient CB2R expression selectively regulated inflammation in the CNS during this disease.

Pharmacological targeting of the CB2R is complex and dependent upon several factors, which include efficacy, ligand affinity, selectivity for intracellular signaling transduction pathways, and tissue distribution (42, 60). Within the context of GVHD-induced neuroinflammation, tissue distribution is of particular importance, since inflammation occurs concurrently within peripheral and central tissue sites, and our prior studies (26), coupled with current data, demonstrated that blockade of the CB2R signaling pathway resulted in discordant immune responses. Thus, additional questions that arise in this setting are whether effective pharmacological targeting is dependent upon penetration into the brain and if therapeutic blockade of the CB2R signaling pathway results in concordant or discordant effects in the periphery and CNS. To address these questions, we initially employed SR144528, which has been validated as the optimal CB2R inverse agonist/antagonist in murine studies due to its high selectivity profile (42). Mass spectrometry revealed that there was minimal accumulation of SR144528 in the brain under inflammatory conditions, demonstrating that this agent is essentially peripherally restricted. More importantly, treatment with this agent had only modest effects on the accumulation of proinflammatory T cells and did not mitigate neuronal cell death, indicating limited therapeutic utility in this context. In contrast, administration of a CB2R inverse agonist/antagonist, SMM-189, had excellent penetration into the brain, which was further augmented under inflammatory conditions in GVHD recipients. Correspondingly, SMM-189 treatment of GVHD mice significantly reduced the accumulation of proinflammatory CD4^+^ and CD8^+^ T cells and decreased neuronal cell death. Further, this agent had no adverse impact on overall survival indicating that, unlike SR144528 (26), there was no exacerbation of overall GVHD-related mortality. Thus, these results demonstrated the feasibility of therapeutically targeting the CB2R signaling pathway in the brain with an inverse agonist/antagonist that can cross the blood-brain barrier and thereby ameliorate neuroinflammation. Presently, we do not know whether SMM-189 is acting primarily as a competitive antagonist (i.e., reducing endocannabinoid activation of CB2R signaling) or as an inverse agonist (i.e., reducing constitutive CB2R signaling). However, we did not find that GVHD produced consistent increases in brain 2-AG concentrations, which suggests that the inverse agonist efficacy of SMM-189 is of primary importance. From a clinical perspective, while several high-affinity CB2R agonists, antagonists, and inverse agonists are available for preclinical studies, none have yet been approved for use in humans. However, the solution of the crystal structure for the CB2 receptor (61) has enabled rational drug design approaches (62, 63), which have the potential to provide candidate drug molecules that could be tested in the clinic in the near future.

IL-6 has been shown to have a pivotal role in the pathophysiology of GVHD. Experimental studies in mice have demonstrated that blockade of this signaling pathway is effective for the prevention of GVHD target organ damage in the periphery (64, 65). In addition, clinical studies have shown that inhibition of IL-6 by administration of tocilizumab, a humanized anti-IL-6R antibody, has efficacy for both the treatment and prevention of this disease (66–69). Within the context of GVHD-induced neuroinflammation, however, blockade of IL-6 signaling in preclinical murine models resulted in only partial protection and failed to correct the accumulation of neurotoxic kynurenine metabolites in the brain (11). Moreover, in other forms of immunotherapy such as CAR T cell administration, inhibition of IL-6 has proven efficacious for the abrogation of systemic side effects (70) but had little benefit for the prevention and/or treatment of neurotoxicity (47). Collectively, these data suggest that IL-6–independent pathways exist and contribute to immune-mediated neuroinflammation. In the current study, we observed that the increased expression of CB2R on microglial cells observed during GVHD was unaffected by administration of an anti-IL-6R antibody, and that this was evident in distinct regions of the brain that have unique functional roles. Since expression of this receptor conveys an inflammatory phenotype on microglial cells, this was evidence of a pathway in the CNS that can promote inflammation and is not inhibited by IL-6 blockade. Additionally, these results provide a potential mechanistic explanation for the limited ability of IL-6-directed strategies to prevent and/or attenuate neurological complications that occur after GVHD and other forms of immunotherapy.

We observed that microglia were the only recipient hematopoietic population that expressed the CB2R in the brain, and that GVHD significantly increased expression of the receptor above that observed in BM control animals. This finding is consistent with earlier studies, which have shown that CB2R expression is very low in resting microglia but is upregulated by inflammatory signals, such as IFN-γ and granulocyte macrophage colony stimulating factor (71), which induce microglial progression to responsive and primed states (72). While microglial cells have been shown to express the CB2R in diverse models including Alzheimer’s, Huntington’s Disease (57), and traumatic brain injury, the role of receptor expression has been controversial. Whereas some studies have shown that CB2R activation inhibits cytokine release by activated microglia (73, 74), others have demonstrated that in vivo–activated CB2R^–/–^ microglial cells release significantly lower amounts of proinflammatory cytokines than WT microglia (75). A confounding factor has been that most studies have utilized global knockout CB2R^–/–^ animals, which has precluded the ability to define the role of CB2R expression on microglial cells only (73). Further, expression of this receptor on microglial cells has been difficult to quantitate accurately using flow cytometric approaches due to antibody nonspecificity and low receptor expression levels (76). To circumvent this obstacle, we created and utilized a CB2R reporter mouse (46) that faithfully identifies CB2R-expressing immune cells (26). Using this reporter mouse, we observed somewhat surprisingly that only approximately 10%–15% of microglial cells expressed the CB2R during GVHD-induced inflammation. To address the functional role of this receptor on microglial cells, we selectively deleted CB2R from these cells and observed that recipients had reduced accumulation of proinflammatory T cells along with a reduction in neuronal cell death, indicating that a minor population of CB2R-expressing microglia exerted potent immunomodulatory effects in the brain.

To delineate mechanistic pathways by which microglial expression of the CB2R modulated inflammatory responses in the brain, we performed GSEA on sorted microglial cells from WT or CB2R^–/–^ recipient mice. This analysis revealed higher expression of genes associated with chemotaxis, chemokine signaling, and leukocyte migration in microglia from WT compared with CB2R^–/–^ recipients, supporting the premise that one of the main functions of CB2R signaling is to promote leukocyte recruitment into the brain during GVHD. These results are consistent with increased expression of chemokine pathway genes in microglia of GVHD mice compared with BM controls (Figure 2). GSEA also revealed enrichment of genes associated with TNF-α signaling in recipients of WT compared with CB2R^–/–^ grafts, suggesting that activation of TNF-α–mediated signaling may be another mechanistic pathway by which CB2R expression on microglia augments neuroinflammation. This observation is consistent with a prior report in which microglial cell production of TNF-α was shown to play a role in the pathogenesis of CNS inflammation during GVHD (14). Interestingly, we noted that microglia from recipient CX3CR1-Cre CB2R^fl/fl^ mice, which were protected from neuroinflammation, had increased expression of genes associated with the IFN-γ signaling pathway. Whereas IFN-γ has been shown to promote CNS inflammation, its role appears to be more nuanced as it can also exert antiinflammatory effects in certain contexts and antagonize proinflammatory functions of TNF-α (77). Thus, another role of microglial CB2R signaling, in addition to the regulation of chemotaxis and leukocyte recruitment, may be to alter the balance between TNF-α and IFN-γ signaling pathways, thereby regulating neuroinflammation during GVHD.

In summary, these studies have identified a critical role for the CB2R signaling pathway in the regulation of GVHD-induced neuroinflammation. Mechanistically, CB2R expression on microglia was associated with an activated inflammatory phenotype, potentiated the accumulation of donor-derived proinflammatory T cells, regulated chemokine and TNF-α gene regulatory networks, and promoted neuronal cell death. Conversely, administration of a brain-penetrant CB2R inverse agonist/antagonist attenuated CNS inflammation without exacerbating systemic GVHD, indicating that this pathway can be therapeutically targeted, which has clinical implications for the mitigation of GVHD-mediated neuroinflammation and potentially other forms of immunotherapy that induce neurological dysfunction.

## Methods

### Sex as a biological variable.

Only male mice (6–12 weeks) were employed as donors and recipients and used to facilitate randomization between control and experimental groups. Male mice within this age range are more tolerant of high-dose total body irradiation in BM transplant experiments.

### Mice.

C57BL/6 (B6) (H-2^b^), Balb/c (H-2^d^), B10.BR (H-2^k^), B6 ACTb-EGFP, CX3CR1-Cre, CB2R^–/–^, IL-34^–/–^, CB2R^fl/fl^ eGFP, and CX3CR1-Cre CB2R^fl/fl^ eGFP mice were bred in the Biomedical Resource Center (BRC) at the Medical College of Wisconsin (MCW) or purchased from Jackson Laboratories. CB2R^EGFP^ reporter mice were constructed by inserting an enhanced green fluorescent protein preceded by an internal ribosomal entry site (IRES) into the 3′ untranslated region of the *cnr2* mouse gene and have been previously described (46). In addition, the entire exon 3, including the 3′ UTR and knocked-in reporter, is flanked by *lox*P sites, which allows for the conditional inactivation of the *cnr2* gene in cells expressing Cre recombinase. CB2R^–/–^ mice were generated by mating the CB2R^EGFP^ mice to CMV-Cre-recombinase–expressing females and have been previously described (46). IL34^–/–^ mice that were created by using Cre recombinase to delete exons 3–5 and replacing them with an IRES-lacZ construct have been described (40). All animals were housed under specific pathogen-free conditions in the Association for Assessment and Accreditation of Laboratory Animal Care–accredited (AAALAC-accredited) BRC of the MCW. Mice received standard 5L0D mouse chow and autoclaved acidified tap water ad libitum.

### BM transplantation.

BM was flushed from donor femurs and tibias with Dulbecco’s modified media (DMEM) (Thermo Fisher Scientific) and passed through sterile mesh filters to obtain single-cell suspensions. Splenocytes were processed by mechanical disruption, and red blood cells were lysed using Tris-buffered ammonium chloride (ACT) solution. Recipient mice were conditioned with total body irradiation (TBI) administered as a single exposure at a dose rate of 900-1100cGy using a Shepherd Mark I Cesium Irradiator (JL Shepherd and Associates). Irradiated recipients received a single intravenous injection in the lateral tail vein of BM with or without added spleen cells. Donors and recipients were sex matched in all transplant experiments and were 6–12 weeks of age. Mice were weighed 2–3 times per week and were euthanized when they attained predefined morbidity criteria.

### Systemic GVHD assessment.

Weights were monitored 3 times per week in all experimental groups. The degree of systemic GVHD was assessed twice weekly using a clinical scoring system that comprises weight loss, fur texture, activity, posture, and skin integrity, as previously described (78). Individual mice in cages were assessed in a blinded fashion and graded from 0 to 2 for each criterion (total score of 10).

### Reagents.

Anti-IL-6R antibody (MR-16-1; Chugai Pharmaceuticals) is a rat IgG antibody that has been previously described (58). Animals received a loading dose of 2 mg intravenously on day 0, and then were treated with 0.5 mg on day 7 by intraperitoneal injection. Rat IgG (Jackson Immunoresearch Laboratories) was used as a control for MR-16-1. SR144528 is a CB2R inverse agonist/antagonist and was purchased from Tocris Bioscience. SR144528 was dissolved in 100% ethanol at a concentration 20 times greater than the final concentration, then an equal volume of Cremophor EL (Sigma-Aldrich) was added with vigorous vortexing, followed by the dropwise addition of sterile saline (18 times the volume of ethanol). Mice received a 3 mg/kg intraperitoneal injection of SR144528 daily for 14 days. Control mice received vehicle only (1:1:18 ratio of ethanol, Cremophor EL, and saline) by intraperitoneal injections. SMM-189, a CB2R inverse agonist/antagonist (43, 44) was prepared using the same method as for SR144528 and administered at a dose of 6 mg/kg via daily intraperitoneal injection for 14 days.

### Isolation of cells.

Cells were isolated from the brain by mechanical disruption followed by collagenase D digestion (Roche Pharmaceuticals). The resulting cell suspension was resuspended in 40% Percoll (GE Healthcare Biosciences) in DMEM and layered on 70% Percoll in DMEM. The resultant gradients were centrifuged at 800*g* with no brake or acceleration at 4°C for 30 minutes. The interface was collected for further analysis. Cell counts were obtained from half of a brain for each experimental animal unless otherwise specified.

### Flow cytometry.

Isolated cells from the brain were labelled with LIVE/DEAD Fixable Aqua Dye (Thermo Fisher Scientific) according to the manufacturer’s instructions. Cells were then stained with monoclonal antibodies conjugated to fluorescent molecules as listed in Supplemental Table 5 after treatment with Fc Block (BD Biosciences). Cells were analyzed on either a BD LSR II or BD LSRFortessa X-20 flow cytometer running BD FACSDiva software or a Cytek Aurora spectral cytometer running SpectroFlo software and analyzed using FlowJo software (TreeStar).

### Intracellular cytokine staining.

Lymphocytes isolated from brain were stimulated with 50 ng/mL PMA, 750 ng/mL Ionomycin, and 2 μM monensin (Thermo Fisher Scientific) for 3.5 hours. Cells were subsequently stained for viability and surface antigens and then intracellularly stained using the eBioscience Intracellular Fixation and Permeabilization Kit (Thermo Fisher Scientific) using the antibodies and reagents listed in Supplemental Table 5.

### cDNA preparation.

Total RNA was extracted from tissues by homogenization in Trizol (Thermo Fisher Scientific) followed by Phenol/Chloroform extraction, washing with isopropyl alcohol followed by 75% EtOH, drying pellets, and resuspending in RNAse free water. cDNA was then made using the QuantiFast Reverse Transcription Kit (Qiagen).

### Real-time q-PCR.

Real-time q-PCR was performed using QuantiTect SYBR Green PCR Kit (Qiagen) and run in a CFX C1000 Real-time Thermal Cycler (Bio-Rad). The 18S reference gene was amplified using QuantiTect Primer Assay Kit (Qiagen). The primers were purchased from Integrated DNA Technologies and are listed in Supplemental Table 6. Primer specificity was verified by melt curve analysis. To calculate fold change in gene expression, the average C(t) value from triplicate wells was compared with the average 18S C(t) value from triplicate wells.

### Statistics.

Data were analyzed with a student’s 2-tailed *t* test with Welch’s correction for 2 group comparisons, and a 1-way ANOVA with Tukey’s test for multiple group comparisons using GraphPad Prism software. Survival curves were compared using the log rank test. Results were considered significant at a *P* value of less than 0.05.

### Study approval.

All animal experiments were carried out under protocols approved by the MCW Institutional Animal Care and Use Committee.

### Data availability.

The authors declare that all data pertaining to the current study are available within the article, Supplemental Information, the Supporting Data Values file, or from the corresponding author upon request. The scRNAseq data from this paper is available in the GEO database with the accession number GSE252964. All other raw data is available from the corresponding author upon request.

Further information is available in Supplemental Methods.

## Author contributions

AM and AR performed animal studies, flow cytometric analysis, single cell RNA sequencing, immunofluorescence staining, and wrote the manuscript. GS performed mass spectrophotometric analysis. AD performed behavioral studies. CYY performed experimental research. BMM provided pharmacological CB2R antagonists. MC and JR provided critical reagents. AS assisted with biostatistical analysis. RKS, WC, and AEZ assisted with single-cell RNA sequence analysis and edited the manuscript. CJH and WRD developed the overall concept, designed experiments, analyzed data, supervised the study, and wrote the manuscript. The authorship order among the cofirst authors was determined on the basis that AM predated AR in conducting experimental studies that formed the basis for this manuscript.

## Supplementary Material

Supplemental data

Unedited blot and gel images

Supporting data values

## Version 1 04/25/2024

In-Press Preview

## Version 2 06/03/2024

Electronic publication
