# Supplementary material for: Type 2 cannabinoid receptor expression on microglial cells regulates neuroinflammation during graft-versus-host disease
Source: J Clin Invest. 2024 Apr 25;134(11):e175205. doi: 10.1172/JCI175205 (PMC11142740; doi:10.1172/JCI175205)
Supplement: Supplemental data [file jci-134-175205-s201.pdf]

SUPPLEMENTARY DATA

TYPE 2 CANNABINOID RECEPTOR EXPRESSION ON MICROGLIAL CELLS REGULATES NEUROINFLAMMATION DURING GRAFT-VERSUS-HOST DISEASE

Alison Moe, Aditya Rayasam, Garrett Sauber, Ravi K. Shah, Ashley Doherty, Cheng-Yin Yuan, Aniko Szabo, Bob M. Moore II, Marco Colonna, Weiguo Cui, Julian Romero, Anthony E. Zamora, Cecilia J. Hillard, and William R. Drobyski

SUPPLEMENTAL METHODS

Fluorescent Microscopy and Image Analyses. For immunofluorescence, mice were first perfused with cold PBS, followed by perfusion with 4% PFA/PBS. Harvested tissues were left in 25% sucrose/PBS overnight at 4°C. 10-40 μm-thick tissue cryosections were cut and stored at −20°C in glycerol/ethylene glycol-based antifreeze solution. Floating sections were incubated in PBS 2 times for 10 minutes min at room temperature before blocking with 5% normal goat serum in PBS for one hour. Tissue was then incubated with primary conjugated antibodies in staining buffer (PBS/ with 2% BSA/ and 0.1% Sodium Azide) with 0.1% Triton X-100 (1:1000) overnight at 37°C. Sections were then washed 2 times for 10 min each time with PBS and secondary antibodies were applied in PBS (1:500) for 2 hours if necessary. Lastly, sections were washed 3 times for 10 min each time with PBS and mounted with ProLong Gold antifade reagent containing DAPI (Invitrogen, Carlsbad, CA). All images were acquired with a confocal microscope (Leica SP8 Upright Confocal Microscope, Buffalo Grove, IL USA). The brightness/contrast of the acquired digital images was applied equally across the entire image and equally to control images and analyzed using Adobe Photoshop CS4 software (Adobe Systems Inc., San Jose, CA) and Image J/FIJI software (National Institute of Health, Bethesda, MD). Cells were quantified as a measure of number per volume or area/volume fraction per volume in 5-8 fields of view (FOV)/hemisphere/region per mouse. Automated protocols for signal intensity (SI) in a $1{\times}10^6/\mu m^3$ voxel and masks were created using ~2SD SI threshold from the Mean Fluorescence Intensity (MFI) with background SI subtracted. Coronal/Sagittal brain maps were created utilizing Adobe Photoshop CS4 software. The following antibodies were purchased from AbCAM: Rat monoclonal anti-CD3 [CD3-12] (ab11089) and polyclonal anti-Cleaved Caspase-3 (ab4051). Polyclonal anti-IBA-1 was purchased from WAKO. Anti-NeuN [A60] (MAB377) was purchased from EMD Millipore. Polyclonal GFP Antibody (A10262) and all fluorescently conjugated secondary antibodies were purchased from Thermo Fisher.

Western blot. Western brain lysates were homogenized in RIPA buffer with phosphatase/protease inhibitors (Thermo Fisher Scientific) and proteins were separated on SDS-polyacrylamide gels (Invitrogen Life Technologies) before being transferred to PVDF membranes. Transferred membranes were washed with TBST and incubated with anti-Spectrin [AA6] (EMD Millipore, Burlington, MA), anti-Cleaved Caspase 3 [ASP175], (Cell Signaling, Danvers, MA), or anti-β-actin (1:5000 Sigma Aldrich) in 5% milk in 0.2% Tween 20/TBS, 4°C, overnight. Membranes were washed and stained with appropriate HRP-labeled secondary antibodies and incubated with enhanced chemiluminescence (ECL) based substrates before being imaged utilizing ChemiDoc Bio-Rad imaging system (Hercules, CA). Band intensities were quantified using Image J/FIJI software (National Institute of Health, Bethesda, MD).

Brain 2-AG Quantification. Brains were harvested from animals and immediately frozen in liquid nitrogen. Frozen brains were thawed enough for dissections; brain regions were quickly weighed then transferred to a liquid nitrogen cooled mortar and disrupted to a fine powder with the pestle. Pulverized, frozen samples were transferred to heavy-walled borosilicate glass tubes with a cell scraper. Two mL of cold acetonitrile and 9 μL of internal standard (deuterated 2-AG; 1800 pg/μl) were added to each tube. After brief homogenization with a glass rod, tubes were transferred to a bath sonicator at 4°C and sonicated for 30 minutes. Protein precipitation was carried out in each tube at -20°C for several hours. Samples were centrifuged at 2400rpm for 3 minutes and the supernatant was transferred to a fresh tube and dried down under nitrogen. Samples were resuspended in 400μL of methanol, and dried. Finally, samples were resuspended in 60μL of methanol. Samples were analyzed with an Agilent 6460 Triple Quadrupole Mass Spectrometer equipped with an HPLC system (Agilent Technologies, Santa Clara, CA), 5μL were used for injection following the methods reported previously (79).

Behavioral Assays. A modification of the procedures described in Roth et al (80) was used to assess behaviors that are associated with sickness. Mice were exposed to a battery of tests over a five-day period, beginning on day 14 after transplant. These tests were designed to interrogate various behavioral domains,

including social behaviors (sociability test); motor function (rotarod and open field); exploration (open field); hedonic drive (sucrose consumption); anxiety (open field center time); sensation (spray test); and stress coping (forced swim test).

Sociability: Experimental mice are singly acclimated to a rectangular arena (14.75” x 14.75” x 12”) for 10 minutes in the absence of any other apparatus. At the same time, a test mouse of the same sex is acclimated in its home cage to the wire holding chamber (7.25” in height x 3.5” in diameter.) After the habituation period, two wire holding chambers are placed at opposite ends of the arena 8” from each other; the test mouse is placed into one of the chambers and the experimental mouse is reintroduced to the arena for 10 min. Time spent interacting with the wire chambers is recorded using Sony HandyCam HDR-CX405 video camera and analyzed using Anymaze software (Stoelting). The outcome measure is the time spent interacting with the chamber containing the test mouse. The arena and wire chambers are cleaned between mice with 70% ethanol and allowed to dry.

Rotarod test: Mice are habituated in their home cages to the testing room for 30 min. On days 1, 2 and 3, mice are placed onto the rotarod apparatus (IITC Life Sciences) set to accelerate from 8 to 40 revolutions per min over 240 seconds. Each mouse is given 3 trials each day. The outcome measure is the average time to fall in three trials on the third (test) day. The apparatus is cleaned between mice with 70% ethanol and allowed to dry.

Open field test: Mice are habituated in their home cages to the testing room for 30 min then placed into round (12.5” in height x 19” in diameter), plexiglass arenas for 30 min. Movement is recorded using Sony HandyCam HDR-CX405 video camera and analyzed using Anymaze software. Outcome measures are: total distance traveled, time immobile, time and distance in the inner zone (defined as the inner circle, 16.3” in diameter) and time in the outer zone (defined as the outer 1.35” ring.) The apparatus is cleaned between mice with 70% ethanol and allowed to dry.

Forced swim test (FST): Mice were placed individually in 700-800 ml of tap water at 24 ± 2°C for six min and behaviors were recorded. Scorers blinded to experimental group determined the time spent struggling and time in which animals were immobile during the last 5 min of the test. Immobility was defined as no

movement other than that required to maintain balance or keep the animal's head above water. Struggling was defined as the movement of three or more limbs at the same time.

Sucrose consumption: Mice were individually housed starting at the end of day 3 of the testing period. On the morning of day 4, water bottles were replaced with two bottles, one containing standard, hypochlorinated water used in the vivarium and the other containing 3% sucrose in the same water. Each bottle contains approximately 50 ml of liquid and is weighed before placing in the cage and after 12 hours. At the end of day 4, at the beginning of the dark period, the sucrose water bottle and hypochlorinated water bottle are switched to eliminate side preference. The bottles are weighed again at the end of 12 hours. The outcome measure is difference in weight of the sucrose-containing bottle minus the difference in weight of the water-containing bottle after the 24-hour period.

Sucrose spray: Mice are singly housed, and their cages are placed under the video camera with a plexiglass top over the cage. Mice were accustomed to this apparatus for 30 min. Each mouse is sprayed on the hindquarters with a 10% sucrose solution until the fur is coated but not dripping wet. Behavior is recorded for seven minutes, and grooming time is scored over the last six minutes by someone blinded to treatment condition. The time spent grooming is the outcome measure for this test.

Composite behavioral score: Using the data generated in the battery of behavioral assays, we calculated the top five most significant categories: Immobility time (open field), struggle time (forced swim), latency to fall (rotarod), interacting time (sociability), and sucrose preference (two bottle choice). The mean and standard deviation were calculated for each category of data, as well as the factor loading to the first principal component (PC1). Individual Z-scores for each test were calculated per mouse. The Z-scores were multiplied by the parameter loading value for each test. The Z-score*parameter loading values for immobility time, struggle time, latency to fall, interacting time, and sucrose preference were added together to generate a composite score for each mouse.

Mass Spectrometry and Assessment of Brain/Blood Distribution of SMM-189 and SR144528. SMM-189 and ^{2}H-SMM-189 (SMM-189-D5) were synthesized as described previously (43). SR144528 was

purchased from Abcam (Waltham, MA, catalog # ab146185) and ^{2}H-SR144528 (SR144528-D7) was provided by the National Institute on Drug Abuse Drug Supply Program (Research Triangle Park, NC). HPLC-grade acetonitrile and water were obtained from Fisher Scientific (Waltham, MA) and reagent-grade formic acid from Sigma Aldrich. SMM-189 and SR144528 concentrations were measured in brain and whole blood using high performance liquid chromatography (HPLC) tandem mass spectrometry (MS/MS). HPLC separation was carried out on a Phenomenex Luna Omega PS C18 50x2.1 mm column (1.6 µm particle size) and MS was carried out on an Agilent 6460 mass spectrometer with Jetstream ionization source and equipped with an Agilent 1290 binary pump and autosampler.

For SMM-189, the aqueous mobile phase (A) was 0.1% formic acid in HPLC-grade water and the organic mobile phase (B) was 0.1% formic acid in acetonitrile. The flow rate was set to 0.2 mL/min. and the elution gradient was held at 10% B for 1.5 minutes, increased to 100% B over the next 6 minutes, held at 100% for 2.5 minutes, returned to 10% B over 2.5 minutes, and held at 10% B for an additional 2.5 minutes. The mass spectrometer was operated in the negative ion mode and the identified ion transitions for SMM-189 and SMM-189-D5 were 356.9 → 184.8 and 361.9 → 184.8, respectively. Retention times for both SMM-189 and SMM-189-D5 were between 6.5 and 7 min. Optimum ion signal was obtained with fragmentor voltage set to 160V and collision-induced dissociation at 25V. Quantification was carried out using a standard curve with 10, 50, 100, 500, 1000, 5000, 10000 ng/mL SMM-189, each also containing 1000 ng/mL SMM-189-D5 prepared in the initial mobile phase (90:10 0.1% formic acid in HPLC grade water: 0.1% formic acid in acetonitrile). A linear standard curve (concentration ratio versus peak area ratio) was generated from the prepared standards with $1/x^2$ weighting applied to the concentration ratio. To assess the brain and blood concentrations of SMM-189, the drug was prepared as described in “Reagents” and mice were injected intraperitoneally with SMM-189 (6 mg/kg) twice at 24 h intervals. Tissue collections were performed one-hour post-injection on the second day of treatment. Animals were anesthetized with isoflurane and then decapitated. Trunk blood was collected, and clotting was prevented by addition of 10% sample volume 0.5M K_2EDTA in water. All samples were kept at -80°C until analysis. Brains were

weighed and homogenized and sonicated in phosphate buffered saline (2 μL PBS/1 mg tissue) until a homogenous solution was obtained. For extraction, 50 μL of brain homogenate or whole blood were added to 200 μL of ice-cold acetonitrile containing 50 ng of SMM-189-D5. Samples were vortexed for 1 minute and then sonicated in ice water bath for 10 minutes. After, they sat on ice for 20 minutes and were centrifuged at 3000 x g for 10 minutes. Supernatants were dried under N_2 gas and resuspended in 50 μL acetonitrile for analysis. Tissue samples from untreated mice were extracted and no quantifiable data were obtained.

For SR144528, the aqueous mobile phase (A) was 0.1% formic acid in HPLC-grade water and the organic mobile phase (B) was 0.1% formic acid in acetonitrile. The flow rate was set to 0.2 mL/min. and the elution gradient was held at 30% B for 1.5 minutes, increased to 100% B over the next 6 minutes, held at 100% for 2.5 minutes, returned to 30% B over 2.5 minutes, and held at 10% B for an additional 2.5 minutes. The mass spectrometer was operated in the positive ion mode and the identified ion transitions for SR144528 and SR144528-D7 were 476.2 → 105.0 and 483.2 → 112.1 respectively. Retention times for both SR144528 and SR144528-D7 were between 5.5- and 6-min Optimum ion signal was obtained with fragmentor voltage set to 140V and collision-induced dissociation at 18V. Quantification was carried out using a standard curve with 1, 5, 10, 50, 100, 250, 500, ng/mL SR144528, each also containing 100 ng/mL SR144528-D7 prepared in the initial mobile phase (70:30 0.1% formic acid in HPLC grade water: 0.1% formic acid in acetonitrile). A linear standard curve (concentration ratio versus peak area ratio) was generated from the prepared standards with $1/x^2$ weighting applied to the concentration ratio. To assess the brain and blood concentrations of SR144528, the drug was prepared as noted in "Reagents" and mice were injected intraperitoneally with SR144528 (3 mg/kg) twice at 24 h intervals. Tissue collections were performed one-hour post-injection on the second day of treatment. Animals were anesthetized with isoflurane and then decapitated. Trunk blood was collected, and clotting was prevented by addition of 10% sample volume 0.5M K_2EDTA in water. Blood was centrifuged at 1000 x g for 10 min and the resulting plasma was harvested. All samples were kept at -80°C until analysis. Brains were weighed and

homogenized and sonicated in phosphate buffered saline (2 μL PBS/1 mg tissue) until a homogenous solution was obtained. For extraction, 100 μL of brain homogenate or plasma were added to 400 μL of ice-cold acetonitrile containing 40 ng of SR144528-D7. Samples were vortexed for 1 minute and then sonicated in ice water bath for 10 minutes. After, they sat on ice for 20 minutes and were centrifuged at 3000 x g for 10 minutes. Supernatants were dried under N_2 gas and resuspended in 50 μL acetonitrile for analysis. Tissue samples from untreated mice were extracted and no quantifiable data were obtained.

Single Cell RNA Sequencing. Cells were isolated from the brains of mice that were transplanted with BM alone or BM plus adjunctive spleen cells (GVHD) by collagenase D digestion and Percoll density centrifugation, and then stained with LIVE/DEAD Fixable Aqua Dye (Invitrogen) according to the manufacturer's instructions. For the analysis of immune cells in BM versus GVHD mice, cells were sorted for live single cells, resuspended in PBS with 2% BSA at a concentration of 700-800 cells per microliter, and used as input for a target cell recovery of 5000 cells. For the analysis of microglial cells from WT versus CB2R$^{-/-}$ animals, cells were sorted for live single recipient (H2K^{b+}) microglia (CD45^{low}, CD11b^{+}), resuspended in PBS with 2% BSA at a concentration of 700-800 cells per microliter, and used as input for a target cell recovery of 5000 cells. Cells from five individual mice were pooled for each sample and were sorted on a BD FACSAria II cell sorter using a 100-micron nozzle. Cells were processed using the 10X Genomics Chromium controller and either the Chromium GEM Single Cell 3' Reagent Kit v3 (10X Genomics, catalog # 1000092) or the Chromium Next GEM Single Cell 5' Reagent Kits v2 (Dual Index) (catalog #1000265). Specifically, sequencing of immune cell populations in the brain from BM control versus GVHD animals was done with the Chromium Next GEM Single Cell 5' Reagent Kit v2, while sequencing of microglia from WT and CB2R$^{-/-}$ mice was done using the Chromium Single Cell 3' Reagents Kit v3. 3' or 5' gene-expression (GEX) libraries were generated using 10X Genomics' library preparation kits according to the manufacturer's protocol. Following library preparation, 10X GEX libraries were converted and PCR amplification was carried out using Singular Genomics' non-indexed S1-SP1 and S2-

SP2 PCR primers at a final concentration of 0.3 μM each. PCR was performed with 2 ng of library input per reaction and 7 cycles of amplification using Q5 DNA polymerase (New England Biolabs, catalog # M0491S). Libraries were quantified using a Qubit 4 Fluorometer (Thermo Fisher Scientific) using the High Sensitivity dsDNA Assay kit (Thermo Fisher Scientific; catalog # Q32854). The quality of the libraries was assessed on a TapeStation 4200 (Agilent Technologies) using High Sensitivity D5000 ScreenTapes (Agilent Technologies; catalog # 5067-5592). Sample-specific GEX libraries were independently loaded into separate lanes of the G4 F2 flow cells and run on Singular Genomics' G4 sequencer using G4 F2 Sequencing Kits (100 cycles) with the following conditions: 28 cycles for read 1 and 91 cycles for read 2.

Single-Cell Gene Expression Data Analysis. Single-cell gene-expression data were processed using Cell Ranger (v.7.0.1, 10X Genomics) and aligned to the mouse (mm10) 2020-A reference. The resulting GEX matrices were analyzed using Seurat (81) (version 4.3.0) within the R statistical environment. For the initial QC step, cells were filtered out cells that expressed <200 or >6,000 genes for the BM versus GVHD analysis, and <200 or >7500 genes for the WT versus CB2R$^{-/-}$ analysis. Cells with >10% mitochondrial transcripts were removed. scRNAseq datasets were integrated and then cell cycle scores were regressed when scaling gene expression values. Dimensionality reduction by principal component analysis was then performed on scaled and normalized data taking into consideration only the 2000 most variable genes in the dataset. Clustering was then performed on dimensionally reduced data using a shared nearest neighbor modularity optimization-based clustering algorithm based on the first 20 principal components and a clustering resolution of 0.5 for BM versus GVHD dataset and a clustering resolution of 0.3 for WT versus CB2R$^{-/-}$ dataset. Clustered data was then visualized using the UMAP algorithm using Seurat's default parameters.

Gene Set Enrichment Analysis (GSEA). To compare expression of certain gene sets between Seurat clusters, we used an R-based implementation of the GSEA method as previously published (82).

Differentially expressed genes were first identified and were then used as an input for GSEA. Hallmark and GO gene sets were loaded from MsigDB and normalized expression scores and adjusted p values were obtained using fgsea.

Dorothea Analysis. Regulon analysis was done using an R-based implementation of DoRothEA (36). Transcription factor-target interactions with confidence levels of A-C were used and the VIPER wrapper was run to determine the normalized enrichment scores for each transcription factor. Pheatmap was then used to graph the normalized enrichment scores.

SUPPLEMENTAL REFERENCES

79. Dean C, et al. Components of the cannabinoid system in the dorsal periaqueductal gray are related to resting heart rate. *Am J Physiol Regul Integr Comp Physiol.* 2016; 311: R254-62.

80. Roth S, et al. Detection of cytokine-induced sickness behavior after ischemic stroke by an optimized behavioral assessment battery. *Brain Behav Immun*. 2021; **91**: 668-672.

81. Hao Y, et al. Integrated analysis of multimodal single-cell data. *Cell*. 2021; 184, 3573-3587.e29. 10.1016/j.cell.2021.04.048.

82. Korotkevich G, et al. Fast gene set enrichment analysis. *bioRxiv*, 2019, doi:10.1101/060012

Supplemental Figure 1

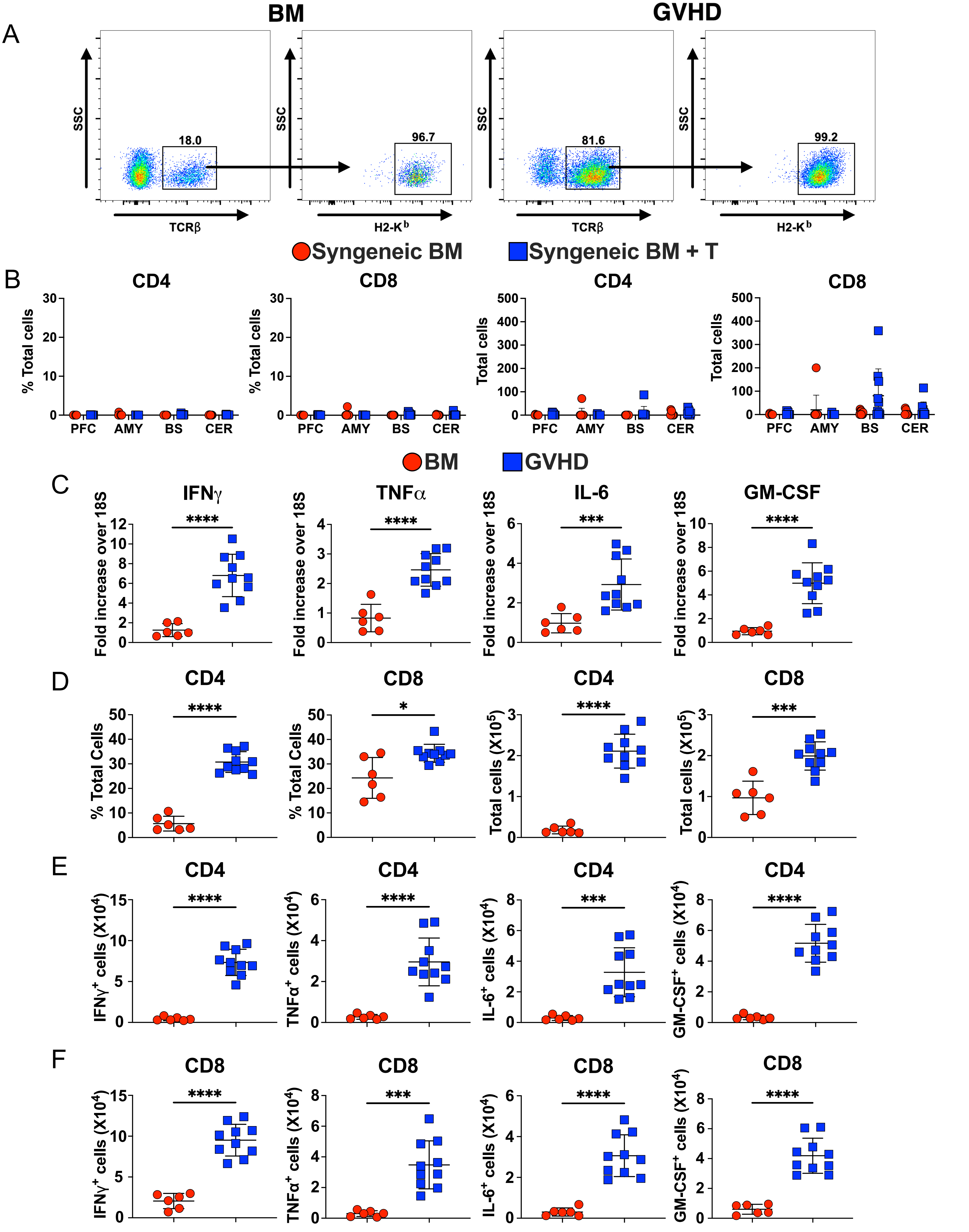

Supplemental Figure 1: **Donor T cells from the brains of GVHD mice are polyfunctional and produce multiple inflammatory cytokines.** (A). Lethally irradiated (900 cGy) Balb/c mice were transplanted with B6 BM alone (5 x 10^6) or B6 BM and spleen cells (adjusted to yield αβ T cell dose of 0.75 x 10^6). Representative dot plots depicting the percentage of T cells (TCRβ$^+$) that were of donor origin (H-2K^{b+}) in the brains of recipients transplanted with BM alone (BM) or BM and spleen cells (GVHD). (B). Lethally irradiated (1000 cGy) B6.PL mice were transplanted with B6^{EGFP} BM (5 x 10^6) alone (BM) or together with B6^{EGFP} spleen cells (adjusted to yield αβ T cell dose of 0.75 x 10^6) (BM + T). The percentage and absolute number of donor derived CD4$^+$ and CD8$^+$ T cells is shown. Data are from two experiments (n=10 mice/group). (C-F). Lethally irradiated (900 cGy) Balb/c mice were transplanted with B6 BM alone (5 x 10^6) or B6 BM and spleen cells (adjusted to yield αβ T cell dose of 0.75 x 10^6). Animals were euthanized 14 days post transplantation. (C). IFN-γ, TNF-α, IL-6, and GM-CSF mRNA expression is depicted in the brain. (D). The percentage and absolute number of donor derived CD4$^+$ and CD8$^+$ T cells. (E, F). The absolute number of CD4$^+$ (panel E) and CD8$^+$ (panel F) T cells that produced IFN-γ, TNF-α, IL-6 or GM-CSF. Results are from two experiments (n=6-10 mice/group). Data are presented as mean ± SD. Statistics were performed using a T test with Welch's correction. *$p<0.05$; **$p<0.01$.

Supplemental Figure 2

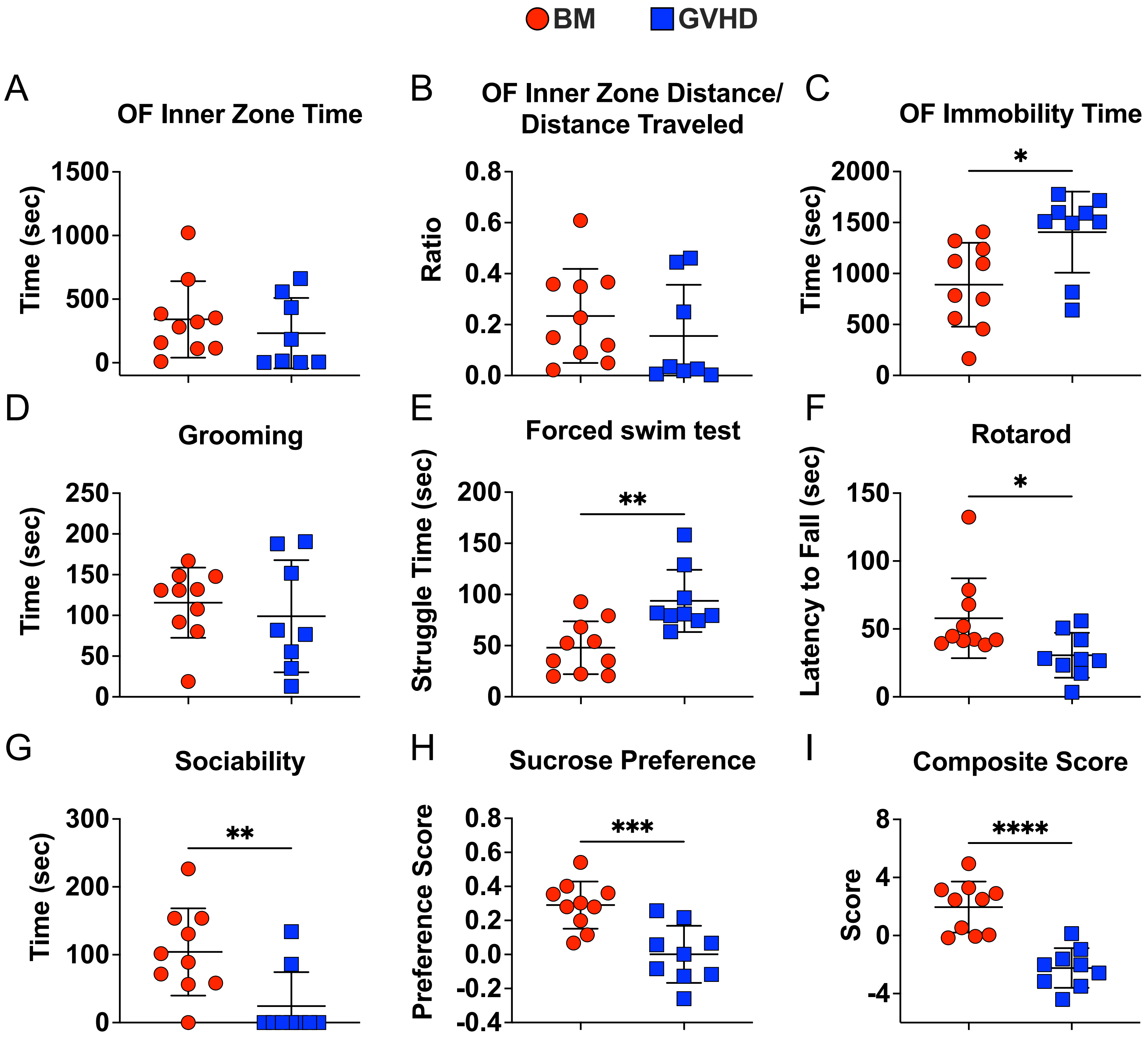

Supplemental Figure 2: **GVHD induces behavioral alterations in mice.** Lethally irradiated (900 cGy) Balb/c mice were transplanted with B6 BM alone (5 x 10^6) or B6 BM and spleen cells (adjusted to yield αβ T cell dose of 0.75 x 10^6). Mice in each cohort were subjected to a battery of behavioral tests 14-18 days post transplantation. (A). Time spent in the inner zone of open field test (OF). (B). Ratio of distance traveled in the inner zone versus total distance in OF test. (C). Total time spent immobile in OF test. (D). Total time grooming after sucrose spray administration. (E). Time spent struggling in forced swim test. (F). Time subject maintained balance prior to fall. (G). Time spent interacting with the chamber containing the test mouse. (H). Difference of weight in sucrose containing bottle minus the difference of weight in water containing bottle. (I). Composite score (see Methods for calculation of score). Data are presented as mean ± SD and were from two experiments (n=8-10 mice/group). Statistics were performed using a T test with Welch's correction. *p<0.05; **p<0.01, ***P<0.001.

Supplemental Figure 3

A

Cluster	Cell Type	BM	GVHD	BM %	GVHD %
0	Microglia	1046	404	42.7	11.2
1	Microglia	570	665	23.3	18.4
2	CD8 T cell	52	762	2.1	21.1
3	Macrophage	233	279	9.5	7.7
4	CD4 T cell	16	491	0.7	13.6
5	CD8 T cell	47	308	1.9	8.5
6	Microglia	185	107	7.6	3.0
7	Microglia	21	203	0.9	5.6
8	Microglia	113	83	4.6	2.3

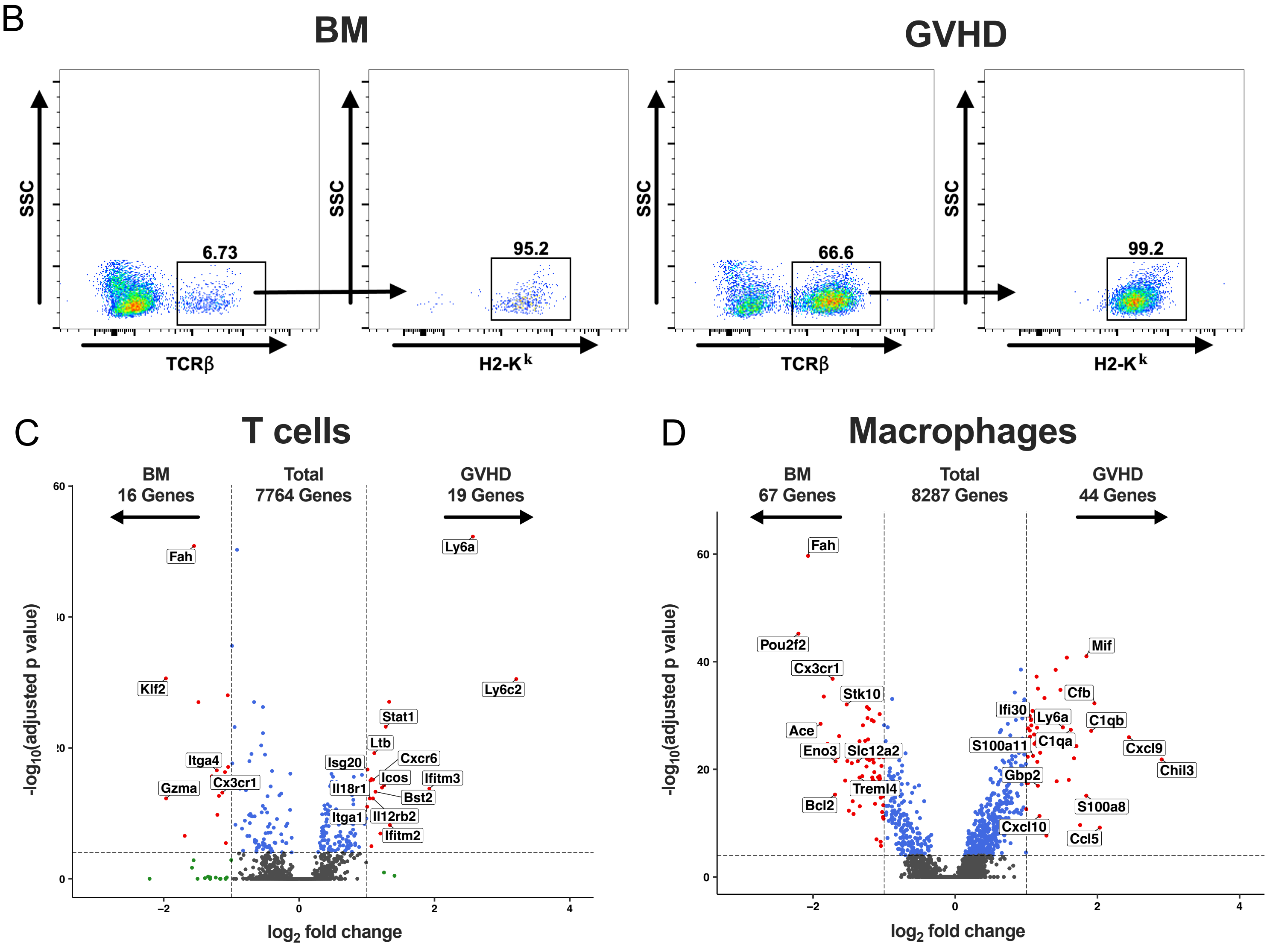

Supplemental Figure 3: **Transcriptional profile of T cells and macrophages in the brain of GVHD mice.** Lethally irradiated (1100 cGy) B6 mice were transplanted with B10.BR BM (5 x 10^6) alone (BM) or together with B10.BR spleen cells (adjusted to yield an αβ T cell dose of 5 x 10^6) (GVHD). (A). Single live cells from pooled brains (n=5/group) were sorted 14 days post transplantation. Absolute number and percentage of cells in each cluster from BM and GVHD mice that underwent single cell RNA sequence analysis. Six clusters representing 7% of cells in the BM scRNAseq dataset and 9.5% of cells in the GVHD dataset were excluded from the analysis. The transcriptional signature of these cells identified them as astrocytes, red blood cells, neurons, NK cells, an unclassifiable subset that had markers of T cells and microglia, and a minor macrophage population. (B). Representative dot plots depict the percentage of T cells (TCRβ^+) that were of donor origin (H-2K^{k+}) in the brains of recipients transplanted with BM alone (BM) or BM and spleen cells (GVHD). (C). Volcano plot showing genes in aggregated T cell clusters that were over expressed in BM versus GVHD mice. Cutoff parameters were |log2(fold change)| > 1.0 and $p_{adjusted}$ <0.0001. (D). Volcano plot showing genes in macrophage cluster that were over expressed in BM versus GVHD mice. Cutoff parameters were |log2(fold change)| > 1.0 and $p_{adjusted}$ <0.0001.

Supplemental Figure 4

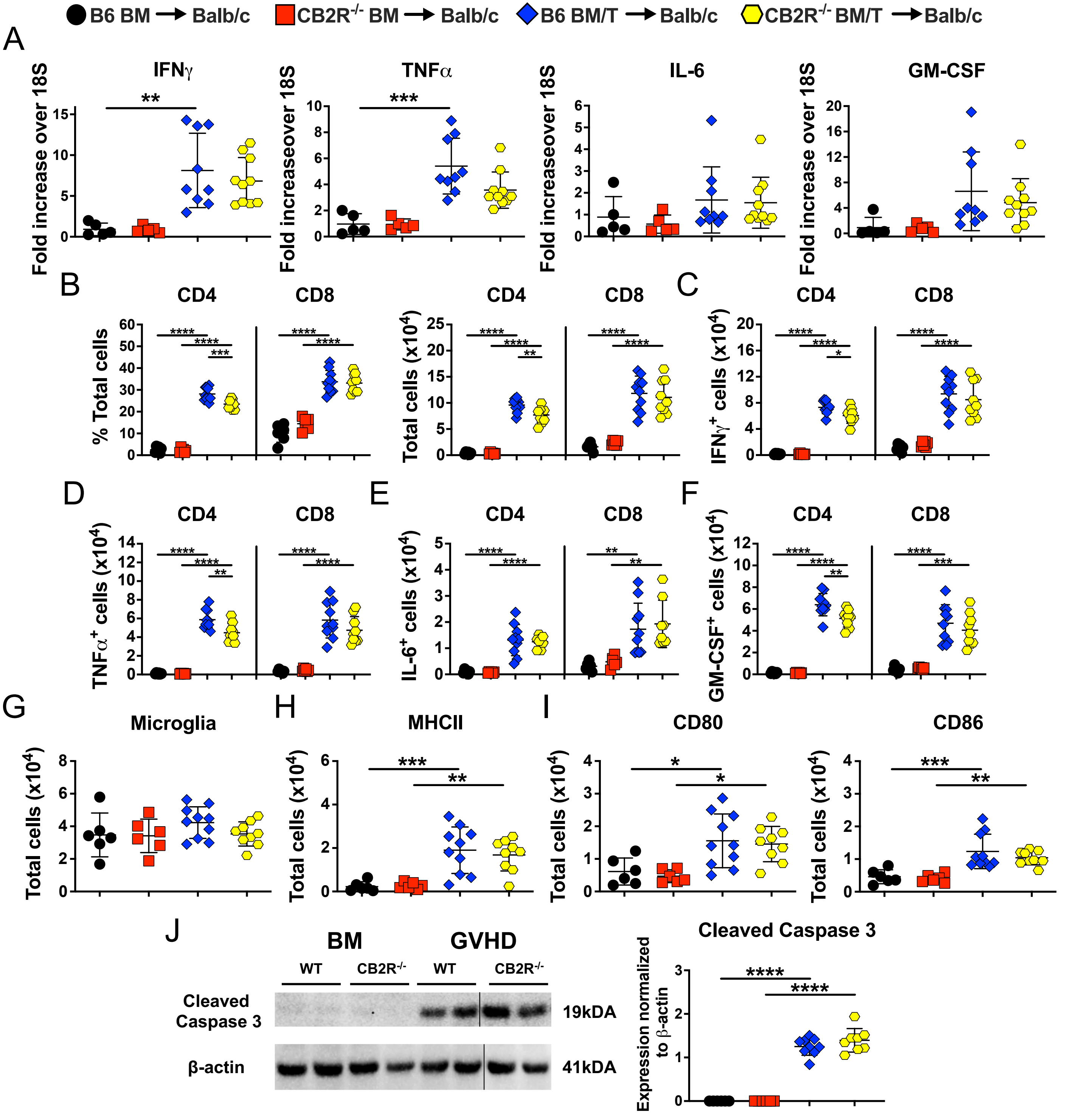

Supplemental Figure 4: **Donor-derived CB2R expression has only modest effects on reducing inflammation in the brain during GVHD.** Lethally irradiated (900 cGy) Balb/c mice were transplanted with B6 or CB2R$^{-/-}$ BM alone or together with B6 or CB2R$^{-/-}$ spleen cells (adjusted to yield an αβ T cell dose of 0.75 x 10^6 T cells). Animals were euthanized 14 days post transplantation. (A). IFN-γ, TNF-α, IL-6, and GM-CSF mRNA expression in the whole brain. Results are from three experiments (n=5-10 mice/group). (B). The percentage and absolute number of donor derived CD4$^+$ and CD8$^+$ T cells. (C-F). The absolute number of donor-derived CD4$^+$ and CD8$^+$ T cells that produced IFN-γ, TNF-α, IL-6 or GM-CSF 14 days post transplantation. Data in panels B-F are from two experiments (n=6-10 mice/group). (G). The absolute number of microglial cells. (H, I). The absolute number of microglia expressing MHC class II, CD80 and CD86. Results are from two experiments (n=6-10 mice/group). (J). Representative western blot images and scatterplots depicting normalized expression of cleaved caspase 3 in the brain. Vertical lines on western blots denote noncontiguous gel lanes. Data are from two experiments (n=6-9 mice/group. Results are presented as mean ± SD. Statistics were performed using a one-way ANOVA with Tukey's test. *p<0.05, **p<0.01, ***p<0.001, ****p<0.0001.

Supplemental Figure 5

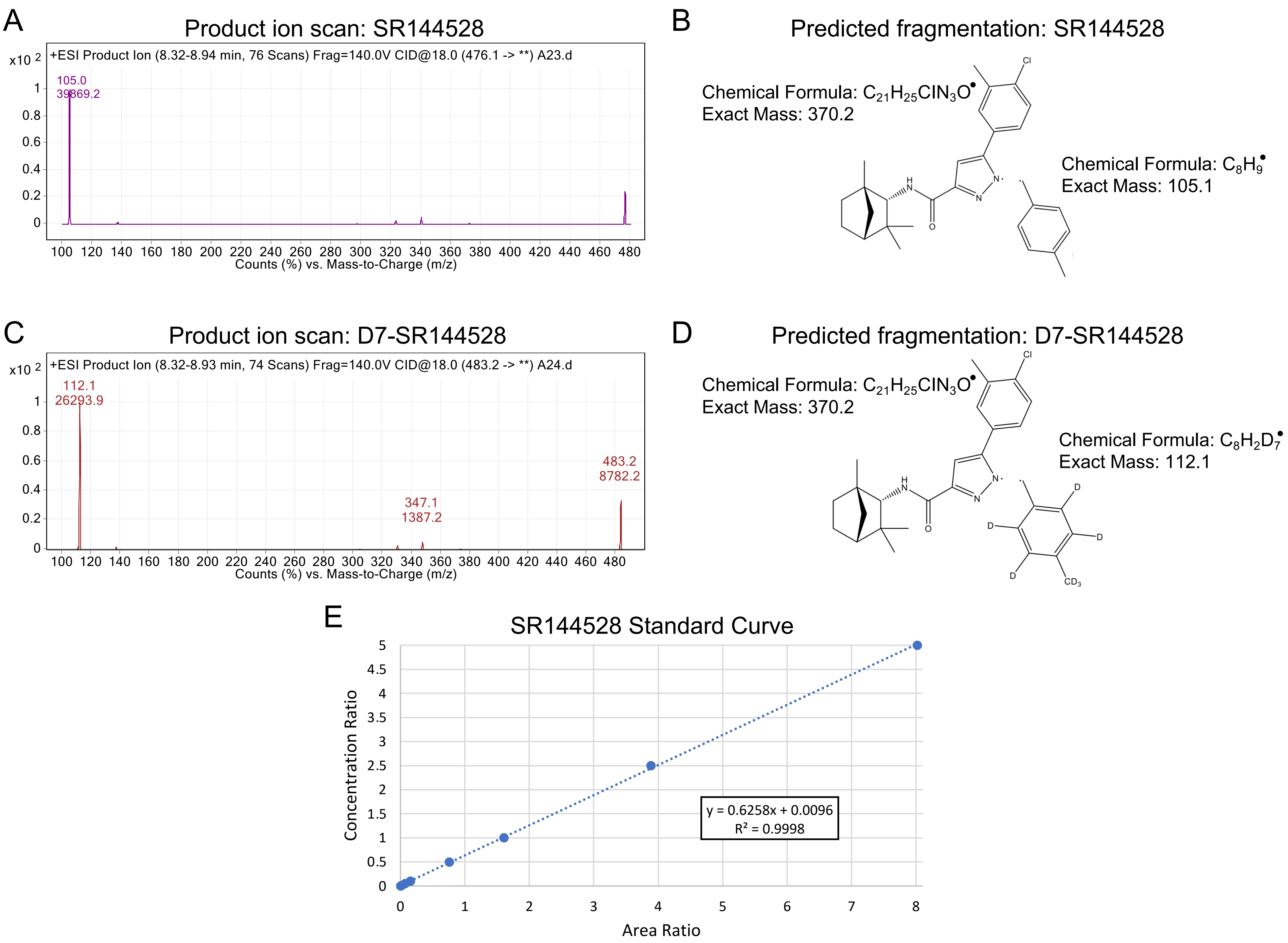

Supplemental Figure 5: **Mass spectral characteristics of SR144528.** (A). Product ion scan of standard SR144528. The compound was injected directly into the mass spectrometer and analyzed using the parameters outlined in Methods. (B). The most abundant daughter ion had an m/z of 105, which is consistent with the fragmentation pattern shown in panel B. (C, D). The daughter ion with greatest abundance for the deuterium labeled SR144528 had an m/z ratio of 112.1 (panel C), consistent with a similar fragmentation pattern and with the presence of seven deuterium atoms on the benzoyl moiety (panel D). These daughter ions were monitored using selective ion monitoring. (E). A representative standard curve constructed using SR144528 concentrations of 1, 5, 10, 50, 100, 250, 500 ng/mL and 100 ng/ml SR144528-D7. SR144528 concentration/100 ng/ml is plotted on the y axis and the area of the peaks for SR144528/SR144528-D7 is plotted on the x axis.

Supplemental Figure 6

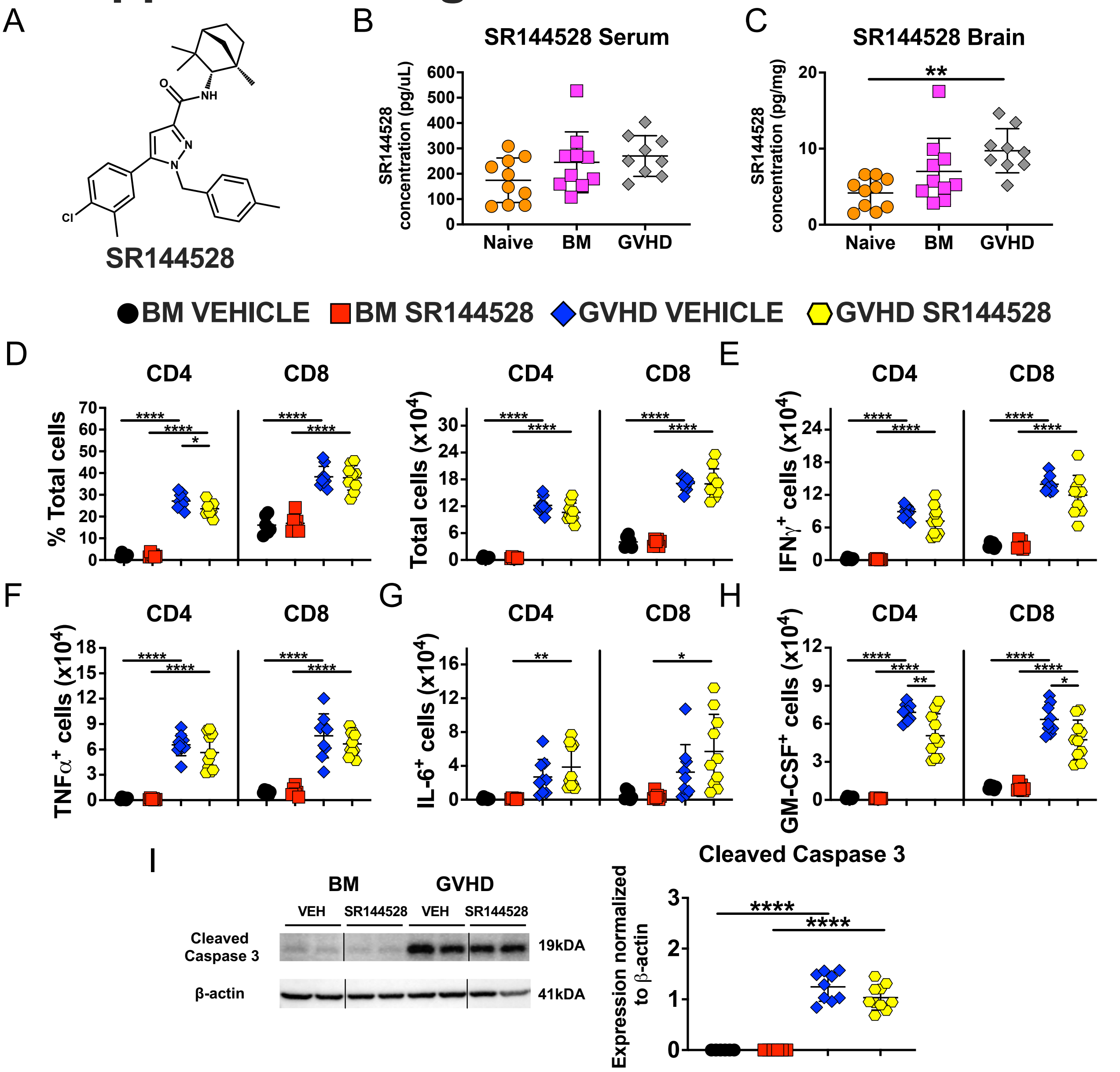

Supplemental Figure 6: **Pharmacological administration of a brain impermeable CB2R antagonist has no significant effect on mitigating CNS inflammation.** (A). Chemical structure of SR144528. (B). Serum levels (pg/µl) of SR144528 in naïve and lethally irradiated (900 cGy) Balb/c mice transplanted with B6 BM alone or together with B6 spleen cells (adjusted to yield an αβ T cell dose of 0.75×10^6). (C). Concentration (pg/mg) of SR144528 in the brain. Data in panels B and C are from two experiments (n=9-10 mice/group. (D-I). Lethally irradiated Balb/c recipients were transplanted with B6 BM (5×10^6) and spleen cells (adjusted to yield an αβ T cell dose of 0.75×10^6 cells). Animals were then treated with SR 144528 (3 mg/kg) or a vehicle control for 14 days beginning on day 0. Balb/c mice transplanted with B6 BM alone and then treated with either vehicle or a SR144528 served as controls. Animals were euthanized 14 days post transplantation. (D). The percentage and absolute number of donor derived $CD4^+$ and $CD8^+$ T cells in the brain. (E-H). The absolute number of $CD4^+$ and $CD8^+$ T cells that produced IFN-γ, TNF-α, IL-6 or GM-CSF. Data in panels D-H are from two experiments (n=6-10 mice/group). (I). Representative western blot images and scatterplots depicting normalized expression of cleaved caspase 3 in the brain. Vertical lines on western blots denote noncontiguous gel lanes. Results are from two experiments (n=6-10 mice/group). Data are presented as mean ± SD. Statistics were performed using a one-way ANOVA with Tukey's test. *$p<0.05$, **$p<0.01$, ***$p<0.001$, ****$p<0.0001$.

Supplemental Figure 7

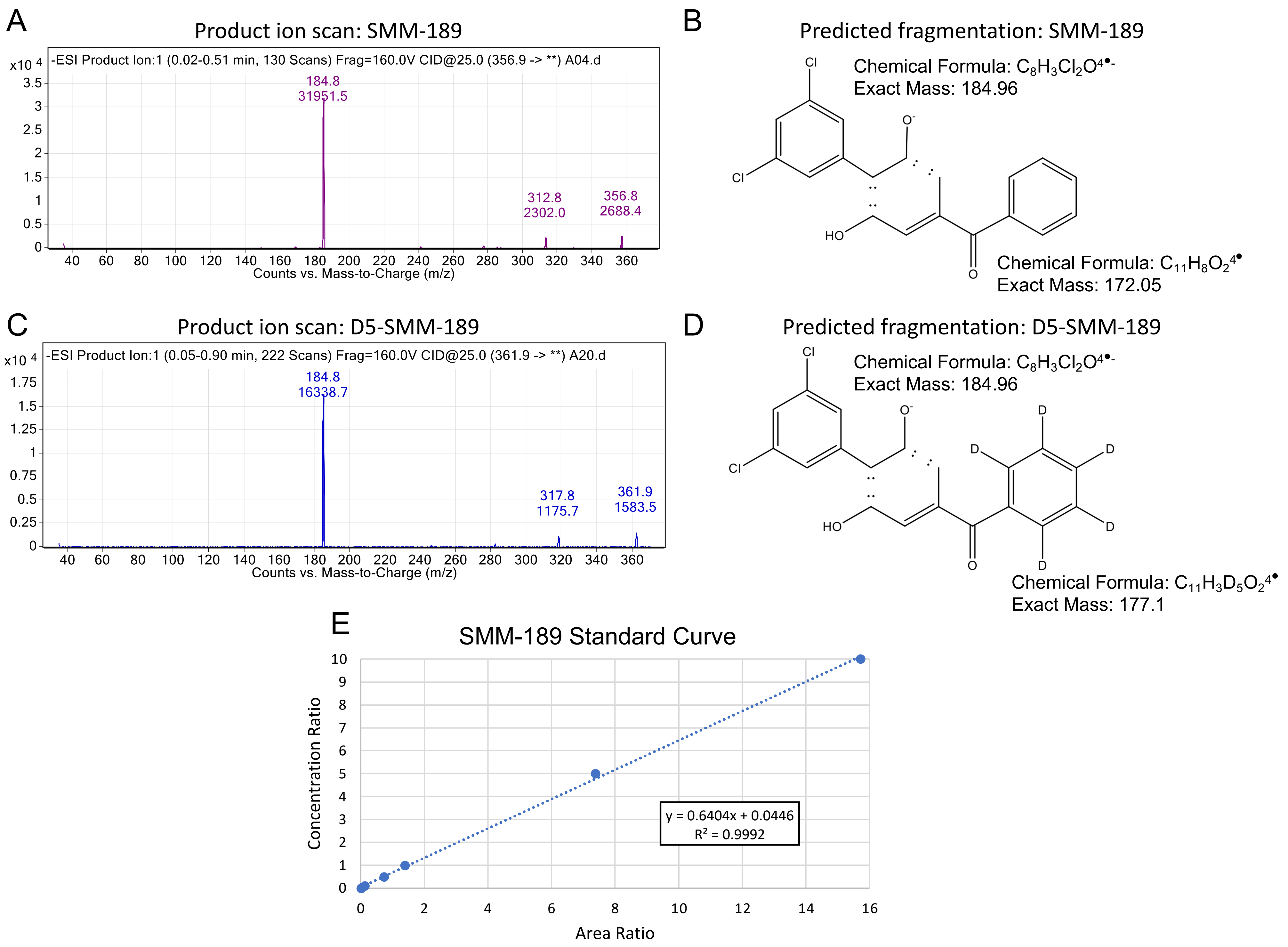

Supplemental Figure 7: **Mass spectral characteristics of SMM-189.** (A). Product ion scan of standard SMM-189. The compound was injected directly into the mass spectrometer and analyzed using the parameters outlined in the methods. (B). The most abundant daughter ion had an m/z of 184.8 which is consistent with the fragmentation pattern shown in panel B. (C, D). The same daughter ion was present in greatest abundance for the deuterium labeled SMM-189 (panel C) consistent with a similar fragmentation pattern (panel D). This daughter ions were monitored using selective ion monitoring. (E). A representative standard curve constructed using SMM-189 concentrations of 10, 50, 100, 500, 1000, 5000, 10000 ng/mL and 1000 ng/ml SMM-189-D5. SMM-189 concentration/1000 ng/ml is plotted on the y axis and the area of the peaks for SMM-189/SMM-189-D5 is plotted on the x axis.

SUPPLEMENTAL TABLE 1. Differentially Regulated Genes in GVHD versus BM T cells

Gene	avg_log2FC	p_val	p_val_adj
Ly6c2	3.208077	9.28E-36	3E-31
Ly6a	2.567448	1.56E-57	5.03E-53
Ifitm3	1.924385	5.08E-19	1.64E-14
Trbv19	1.409484	1.08E-05	0.350053
Ifitm2	1.341234	1.96E-13	6.33E-09
AU020206	1.330486	2.84E-32	9.15E-28
Stat1	1.279438	1.8E-28	5.81E-24
Plac8	1.256192	1.59E-19	5.13E-15
Trbv13-2	1.253727	3.43E-06	0.110689
Icos	1.224936	3.6E-19	1.16E-14
Cd4	1.201073	3.75E-12	1.21E-07
Bst2	1.127778	1.53E-18	4.96E-14
Ltb	1.111168	1.95E-24	6.31E-20
Cxcr6	1.092178	2.18E-20	7.05E-16
Il12rb2	1.090976	1.64E-17	5.3E-13
Inpp4b	1.069197	1.82E-20	5.88E-16
Ifi27l2a	1.068756	3.19E-10	1.03E-05
Rora	1.054915	2.85E-20	9.19E-16
Il18r1	1.043753	1.62E-17	5.24E-13
Isg20	1.011475	6.26E-22	2.02E-17
Itga1	1.005883	3.02E-16	9.74E-12
Jaml	0.993767	1.93E-14	6.22E-10
Tnfrsf4	0.990977	1.02E-07	0.003279
Gbp6	0.979603	1.6E-19	5.16E-15
Igtp	0.970512	9.52E-19	3.07E-14
Hif1a	0.96805	1.34E-13	4.32E-09
Zbp1	0.929497	2.93E-15	9.45E-11
Efhd2	0.927916	4.1E-21	1.32E-16
Slco3a1	0.918292	3.18E-15	1.03E-10
Tnf	0.898248	4.68E-09	0.000151
Ms4a6b	0.894583	1.81E-19	5.85E-15
St6galnac3	0.885649	4.38E-10	1.41E-05
Slfn1	0.880593	3.4E-15	1.1E-10
AA467197	0.865882	3.36E-15	1.08E-10
Mt1	0.861703	8.09E-09	0.000261
Ifng	0.859512	0.020298	1
Socs1	0.830511	4.28E-12	1.38E-07
Irf7	0.828664	1.18E-10	3.82E-06
Ctla4	0.828294	2.22E-10	7.18E-06
Il7r	0.816251	2.12E-08	0.000685
Rtp4	0.814762	8.43E-12	2.72E-07
Junb	0.812862	1.97E-08	0.000637
AW112010	0.807195	8.17E-21	2.64E-16
Srgn	0.805845	1.06E-18	3.42E-14

Satb1	0.802387	1.29E-16	4.18E-12
mt-Atp8	0.800684	2.64E-17	8.52E-13
Ifi47	0.797437	1.49E-13	4.82E-09
Lilr4b	0.797183	9.31E-13	3E-08
Shisa5	0.782012	5.17E-18	1.67E-13
Gstp3	0.781033	1.08E-13	3.48E-09
Glrx	0.779712	2.25E-09	7.26E-05
Cd40lg	0.777519	5.39E-09	0.000174
Gramd3	0.768432	3.48E-12	1.12E-07
Cd274	0.765578	2.36E-10	7.63E-06
Aldoa	0.761132	1.31E-14	4.23E-10
Gm36723	0.76083	1.37E-10	4.44E-06
Ifit3	0.759321	9.23E-09	0.000298
Arhgap26	0.757268	2.54E-12	8.2E-08
Bcl2a1b	0.755196	2.72E-12	8.78E-08
Lilrb4a	0.735931	5.65E-11	1.82E-06
Rnf213	0.733787	6.16E-10	1.99E-05
Gbp9	0.723811	2.23E-10	7.19E-06
Runx2	0.721161	1.07E-11	3.44E-07
Rgs1	0.713811	6.49E-08	0.002096
Cd69	0.712157	2.63E-06	0.084853
Rgs10	0.706416	3.88E-10	1.25E-05
Emb	0.706167	7.31E-11	2.36E-06
Irgm1	0.701917	8.53E-10	2.75E-05
2410006H16	0.697229	3.63E-11	1.17E-06
Gzmb	0.67888	0.278156	1
Isg15	0.67732	1.72E-09	5.55E-05
Tnfsf8	0.67702	4.09E-06	0.131913
Ifi213	0.669539	9.01E-08	0.002908
Cd226	0.666688	4.6E-09	0.000149
Ms4a4c	0.663378	7.55E-08	0.002438
Apol7e	0.651722	2.5E-10	8.07E-06
Ly6e	0.650538	4.64E-19	1.5E-14
Gbp10	0.644742	3.38E-09	0.000109
Stat3	0.643054	2.3E-11	7.43E-07
Ddit4	0.641827	5.51E-08	0.00178
Prkca	0.635565	8.68E-08	0.002804
Cd6	0.63289	5.57E-08	0.001798
Trbv13-3	0.632491	0.010368	1
Pde3b	0.628523	1.46E-08	0.00047
Xaf1	0.614219	2.65E-08	0.000857
Csf1	0.613714	4.88E-05	1
Nedd9	0.61152	9.01E-12	2.91E-07
Ifit1	0.610595	1.32E-05	0.425389
Prdx6	0.608777	2.12E-12	6.85E-08
Id2	0.603822	7.5E-11	2.42E-06
Parp9	0.603706	2.74E-10	8.86E-06
Ccl4	0.598105	0.98797	1

Sdf4	0.594783	1.92E-07	0.006211
Cxcr3	0.593859	1.28E-08	0.000412
Lpxn	0.586472	7.49E-11	2.42E-06
Gm10260	0.585503	1.7E-13	5.48E-09
Nrgn	0.585384	0.001744	1
H2-Q1	0.580907	8.48E-12	2.74E-07
Bcl2a1d	0.579047	1.98E-08	0.000639
Slfn2	0.576913	2.05E-08	0.00066
Themis	0.575268	2E-08	0.000646
Dnajc15	0.574685	9.05E-10	2.92E-05
Ifngr1	0.56603	1.01E-08	0.000326
Gbp4	0.564895	4.75E-08	0.001534
Rpl12	0.55992	1.1E-14	3.55E-10
Rps12	0.553	5.22E-20	1.68E-15
Zfp36l2	0.547805	1.12E-06	0.036233
Clint1	0.547117	7.08E-08	0.002284
Ccr8	0.546981	2E-05	0.645759
Fth1	0.546322	7.24E-16	2.34E-11
Rps2	0.546093	3.2E-15	1.03E-10
Btg1	0.544147	1.19E-11	3.85E-07
Peli1	0.541398	5.85E-08	0.001887
Gm19585	0.540119	6.14E-08	0.001982
Ehd1	0.539922	5.78E-07	0.018672
Crlf2	0.537648	3.02E-08	0.000975
Pag1	0.535883	2.07E-06	0.066693
Krt83	0.535579	2.28E-06	0.073625
Gas5	0.531706	1.65E-09	5.31E-05
4933406I18F	0.52867	5.04E-08	0.001627
H2-Q2	0.524532	1.48E-10	4.77E-06
Nfkbia	0.522275	0.000182	1
Tgtp2	0.518373	2.85E-08	0.000922
Gbp7	0.516668	7.2E-07	0.023259
Pdcd1	0.512739	0.002166	1
Rps14	0.512183	3.75E-23	1.21E-18
Batf	0.511283	2.36E-05	0.762662
Gldc	0.509709	2.13E-06	0.068712
Rps15a	0.503645	7.58E-20	2.45E-15
Rpl27a	0.500507	7.87E-21	2.54E-16
Dusp2	0.499367	2.95E-07	0.009517
Litaf	0.498504	3.44E-07	0.011116
Jun	0.498424	1.4E-07	0.004534
Gpr18	0.497137	1.2E-07	0.003884
Tnfsf10	0.497081	1.17E-07	0.00379
Pkm	0.495373	1.33E-09	4.29E-05
Ctsw	0.494299	9.44E-08	0.003048
Ifi208	0.493742	5.53E-07	0.017849
Oas3	0.493509	9.58E-06	0.309314
Smyd3	0.487761	3.99E-07	0.012872

Mapkapk3	0.485675	4.74E-06	0.153192
Rps23	0.483763	4.52E-20	1.46E-15
Tab2	0.483045	2.23E-05	0.719359
Epsti1	0.480123	3.9E-07	0.012602
Gm15283	0.479388	3.22E-06	0.103967
Rps24	0.478088	2.93E-21	9.46E-17
Lrp10	0.477849	2.33E-07	0.007517
Gbp2	0.477726	1.27E-09	4.09E-05
Relb	0.474035	1.92E-07	0.006193
Rpl9	0.470242	1.28E-19	4.13E-15
Traf1	0.469685	1.81E-06	0.058381
Podnl1	0.469114	0.000371	1
Pgk1	0.467341	4.22E-07	0.013614
Gbp8	0.46325	7.05E-06	0.22774
1600014C10	0.462138	1.39E-05	0.447176
Grina	0.460714	8.64E-06	0.278995
Tpi1	0.459356	0.000336	1
Psme2	0.458475	9.2E-09	0.000297
Rpl23	0.458293	2.23E-19	7.21E-15
Anxa1	0.457188	3.68E-10	1.19E-05
Rpl24	0.456554	2.65E-16	8.54E-12
Chmp4b	0.452948	2.49E-07	0.008052
Serpina3f	0.450512	0.000607	1
H2afy	0.446252	8.56E-07	0.02764
Lef1	0.444917	8.62E-05	1
Adam19	0.444002	6.61E-05	1
Ddx24	0.4438	1.93E-06	0.062249
Pdcd1lg2	0.441419	1.54E-08	0.000498
Cd3g	0.44106	7.12E-08	0.002297
Npm1	0.440044	1.75E-09	5.65E-05
Ubash3a	0.438538	7.67E-10	2.48E-05
Gm8909	0.437761	3.94E-08	0.00127
Sipa1l1	0.437462	6.81E-06	0.219722
Rpl7a	0.436804	2.79E-13	8.99E-09
Rplp1	0.435955	4.23E-15	1.37E-10
Tapbp	0.435011	5.25E-07	0.01695
Rps20	0.433794	3.77E-11	1.22E-06
Klk8	0.433719	4.01E-05	1
Il2ra	0.433488	3.65E-07	0.011779
Mif	0.432106	4.31E-06	0.13921
Gramd1a	0.429697	2.45E-06	0.079248
Grcc10	0.429253	4.06E-08	0.00131
Uhrf2	0.427688	7.82E-05	1
Rpl17	0.427376	1.18E-15	3.8E-11
Psmb9	0.426984	3.65E-09	0.000118
Igflr1	0.425969	1.85E-06	0.059703
Il2rg	0.425083	1.5E-08	0.000485
Cd5	0.424512	0.000127	1

Oas1a	0.423764	4.59E-05	1
Hopx	0.423646	0.000107	1
Fam102a	0.423202	4.26E-05	1
Samhd1	0.422202	9.59E-05	1
Rps9	0.421548	1.03E-15	3.31E-11
Rpl13	0.421074	1.24E-16	4.01E-12
Trac	0.420427	2.89E-07	0.00934
Rpl15	0.419772	1.09E-10	3.53E-06
Lncpint	0.418571	9.77E-05	1
Oasl2	0.416691	4.24E-05	1
Herc6	0.416022	0.000207	1
Gm12216	0.415454	6.36E-06	0.205274
Irgm2	0.414801	1.21E-06	0.038969
Orai1	0.413016	8.17E-07	0.026392
Rpl18	0.411463	2.89E-15	9.34E-11
Crot	0.411108	8.48E-06	0.273664
Nek7	0.411012	0.000999	1
Rpl29	0.409695	2.46E-14	7.94E-10
Ipcef1	0.409146	3.64E-05	1
H1f0	0.408731	9.23E-06	0.297984
Tap1	0.408202	2.41E-06	0.077785
Lag3	0.405971	0.085	1
Stat2	0.403813	0.000762	1
Rpl3	0.403622	2.98E-12	9.63E-08
Tecpr1	0.40337	0.0005	1
Irf1	0.401808	0.0002	1
Lgals3bp	0.401492	1.14E-05	0.367634
Rpl11	0.400703	6.13E-14	1.98E-09
Map2k2	0.397909	9.4E-07	0.030352
Cd86	0.397713	0.000158	1
Rpl26	0.397453	2.35E-13	7.59E-09
Gpr55	0.39682	1.87E-06	0.060517
Ppp3cc	0.395929	0.000109	1
Rbpj	0.395547	0.00035	1
Atf6	0.394983	0.00029	1
Gpr183	0.394678	0.000384	1
Gm7030	0.393947	4.45E-06	0.143668
Slamf1	0.393242	2.53E-05	0.816591
Ptpn18	0.391656	2.24E-08	0.000723
Rpl18a	0.390988	3.7E-15	1.19E-10
Ly75	0.390632	4.54E-06	0.146695
Mrpl52	0.388072	6.62E-07	0.021368
Fgl2	0.386055	0.000234	1
Rps13	0.385451	6.51E-16	2.1E-11
Ogt	0.384269	0.001286	1
Rabac1	0.384093	8.99E-06	0.290225
Ddx60	0.38349	1.5E-06	0.04835
Gng2	0.383315	1.25E-05	0.403631

Tigit	0.382022	0.000133	1
Celf2	0.381267	0.000104	1
Tnfrsf18	0.380983	0.007871	1
Rpl14	0.380512	4E-11	1.29E-06
Arl15	0.379447	0.010097	1
Arf5	0.378785	4.11E-07	0.013283
Coro2a	0.377987	6.43E-05	1
Atxn1	0.377515	0.000328	1
Vps54	0.377152	0.000196	1
Trim30a	0.3748	0.000792	1
Itk	0.374442	2.04E-06	0.065744
Rps21	0.374011	6.27E-09	0.000202
Ifit3b	0.373748	9.34E-05	1
Rpl30	0.373449	2.15E-12	6.93E-08
Rps10	0.37338	1.92E-16	6.18E-12
Itpr1	0.372986	0.000346	1
Mdfic	0.372528	0.000323	1
Il18rap	0.372275	4.21E-06	0.135841
Nme2	0.371639	3.05E-06	0.098537
Rpl6	0.371122	8.25E-12	2.66E-07
Fndc3a	0.370745	0.000137	1
Rpl10	0.369459	3.42E-10	1.1E-05
Ttr	0.369371	1.24E-08	0.000399
Marc2	0.369032	1.91E-05	0.616147
Dtx3l	0.36868	0.0004	1
Jak3	0.368317	0.00024	1
H2-Q4	0.367884	4.28E-08	0.001381
Arid5a	0.366534	0.00013	1
Rplp2	0.36617	2.04E-12	6.58E-08
Tap2	0.365908	1.18E-06	0.038252
Nfkbil1	0.365464	1.33E-05	0.429749
Rack1	0.360923	1.01E-09	3.25E-05
Rpl34	0.360223	3.23E-13	1.04E-08
Maf	0.358293	0.009774	1
Ypel3	0.357456	0.000113	1
Psma5	0.357047	2.68E-05	0.865931
Rpsa	0.356363	1.19E-11	3.83E-07
Rel	0.354154	0.005332	1
Rpl27	0.35371	9.64E-13	3.11E-08
Kxd1	0.353654	0.00069	1
Rgs16	0.352893	0.118375	1
Npc2	0.352104	4.95E-05	1
Itm2c	0.351662	0.00027	1
Sdcbp2	0.351092	3.56E-05	1
Gpr171	0.349853	3.54E-05	1
Ms4a4b	0.348955	6.38E-05	1
Flt3l	0.348197	0.000723	1
Nmi	0.346877	0.001085	1

Sdhaf1	0.345682	7.04E-05	1
Rps5	0.345571	3.68E-12	1.19E-07
Mrps34	0.345241	3.04E-05	0.982688
Rpl32	0.343427	4.21E-11	1.36E-06
Rps7	0.342839	3.4E-12	1.1E-07
Gstp1	0.342792	0.000608	1
Cish	0.342781	0.001084	1
Rpl8	0.342634	5.3E-10	1.71E-05
Ifi35	0.341955	0.000176	1
Cd96	0.340954	0.00016	1
Npepl1	0.340939	6.4E-05	1
Egr1	0.340747	0.001061	1
Tpt1	0.340422	4.37E-15	1.41E-10
H2-Q10	0.339813	5.18E-05	1
Rpl19	0.339418	1.58E-12	5.11E-08
Notch1	0.339393	0.001886	1
B2m	0.339328	4.78E-13	1.54E-08
Plscr1	0.338506	0.014701	1
Rpl5	0.337341	1.1E-06	0.035662
Cd47	0.337144	9.96E-06	0.321584
Ubac2	0.336715	7.5E-05	1
Ndfip1	0.3361	0.001249	1
Eef1b2	0.335657	1.54E-07	0.004957
Pfdn5	0.335461	3.75E-07	0.01211
Psmb8	0.334769	6.3E-09	0.000204
Gimap3	0.334759	1.04E-05	0.336065
Apobec3	0.334353	2.53E-05	0.815251
Nrp1	0.333263	0.026466	1
Cd8b1	0.331462	0.101471	1
Cytip	0.330319	0.000589	1
Gls	0.329876	0.001793	1
Slfn8	0.328395	5.05E-06	0.162962
Fxyd5	0.327838	2.53E-08	0.000818
Glmn	0.327575	0.00021	1
Adgrg5	0.327341	2.55E-06	0.082268
Rpl31	0.326294	3.26E-05	1
Rpl21	0.326013	6.8E-10	2.19E-05
Rps4x	0.325929	1.11E-11	3.59E-07
Tent5c	0.325447	4.02E-05	1
St6galnac4	0.325064	5.27E-05	1
Eif3e	0.324344	1.24E-05	0.401254
B3gnt2	0.324113	0.000765	1
Snhg12	0.324017	0.000177	1
Rps25	0.323766	6.69E-06	0.216139
Rps11	0.32298	2.77E-10	8.93E-06
Abhd17a	0.322921	0.000242	1
Tmem176a	0.322323	0.000567	1
Gm8369	0.322301	6.25E-05	1

Zup1	0.322106	0.003885	1
Rps27a	0.32112	3.11E-10	1E-05
Csf2	0.320513	0.021528	1
Thada	0.319654	0.00262	1
Cirbp	0.319448	0.00324	1
Rpl36	0.318477	1.2E-08	0.000388
Cysltr2	0.318169	0.000165	1
Nfkbiz	0.317827	0.109599	1
Susd6	0.316703	0.009793	1
Gpr68	0.316505	0.000157	1
Zfas1	0.315887	0.000915	1
Pcca	0.315587	3.6E-05	1
H2-M3	0.315161	0.000143	1
Uba7	0.314908	0.003284	1
Eea1	0.314815	0.760457	1
Ctla2a	0.313393	0.015595	1
Hcst	0.312908	0.000382	1
Dnaja1	0.310426	0.002423	1
Pbxip1	0.310385	0.009532	1
Ifi211	0.310327	3.17E-05	1
Oxsr1	0.310178	0.026596	1
Ptpn22	0.308644	0.00026	1
Slc3a2	0.308487	0.000713	1
Gpi1	0.307629	5.39E-05	1
Atp8b4	0.30761	0.001567	1
Rpl4	0.307001	8.61E-07	0.027794
Pde4d	0.305544	1.98E-05	0.638005
Rab2a	0.304245	7.4E-05	1
Rps16	0.304027	1.01E-10	3.27E-06
Klhl6	0.303705	0.000239	1
Selenow	0.30264	0.000511	1
Ube2l6	0.302432	0.01585	1
Armc7	0.301795	0.012328	1
Psme1	0.301657	1.43E-05	0.461259
Maml2	0.299927	0.018509	1
Tex2	0.299175	0.00143	1
Eef1g	0.299057	0.000138	1
Fkbp5	0.298413	0.004272	1
Hnrnpll	0.298361	0.000115	1
Pfkp	0.29769	0.0007	1
Pfkl	0.297316	0.00042	1
Tmem106b	0.296918	0.002771	1
Birc2	0.296516	9.8E-05	1
Eif4a2	0.295097	0.00231	1
Pgam1	0.294797	0.012963	1
Eef1d	0.293661	0.000371	1
Rbm3	0.293636	1.16E-05	0.375389
Trafd1	0.29248	0.012932	1

Fam78a	0.292255	0.007956	1
Hsp90ab1	0.292108	6.68E-05	1
Tor1aip1	0.291942	0.000492	1
Airn	0.291632	0.00712	1
Parp14	0.291211	0.009183	1
Zeb1	0.291206	0.005829	1
Endod1	0.290753	0.003082	1
Havcr2	0.290059	0.214617	1
Phf11b	0.288336	0.026447	1
Tspan31	0.288149	0.000527	1
Atp5g2	0.287313	7.94E-06	0.256303
Trim34a	0.286985	0.008224	1
Afg3l2	0.28674	0.000163	1
Hspbp1	0.286286	0.001476	1
Ccdc107	0.284389	0.000221	1
Klrb1c	0.283897	0.000229	1
Ikbkb	0.28376	0.003686	1
Rpl36a	0.282621	0.00012	1
Fau	0.282052	2.43E-09	7.85E-05
Ccnl1	0.281939	0.002042	1
Gmfg	0.281884	0.002042	1
Hpcal1	0.28163	0.001807	1
Tmem176b	0.28015	0.006479	1
Nisch	0.279717	0.004138	1
Cd160	0.279222	0.017141	1
Cebpb	0.278818	2.89E-05	0.932519
Stat4	0.278278	0.001033	1
Ggh	0.277965	0.001105	1
Sit1	0.277771	0.014688	1
4930549P19I	0.277706	0.000326	1
Izumo1r	0.277083	0.009163	1
Pde4b	0.27675	0.001267	1
Crim1	0.275354	0.001646	1
Pkp3	0.274869	0.08379	1
Zbtb7b	0.27419	4.53E-05	1
Cd3d	0.274076	0.000784	1
Sik3	0.273903	0.003554	1
Dhx58	0.27356	0.009928	1
Rps8	0.27293	7.12E-06	0.229864
Ybx3	0.272877	0.000935	1
Dis3l2	0.272389	0.006232	1
Skil	0.271395	0.007267	1
Tmem59	0.271119	0.000329	1
Acbd6	0.271112	0.003949	1
C1qbp	0.270786	0.017	1
Pdcd6	0.269536	0.001376	1
Rplp0	0.26952	1.7E-06	0.054995
Arap2	0.269002	0.097656	1

Mrpl23	0.26838	0.001583	1
Dcun1d5	0.267809	0.000652	1
Pld3	0.266123	0.006047	1
Rpl10a	0.266021	1.12E-05	0.362193
Eif3f	0.265868	4.64E-06	0.149956
Ppil4	0.26584	0.002859	1
Limd2	0.265643	0.000968	1
Mink1	0.265303	0.01061	1
Rapgef6	0.265149	0.001009	1
Mob2	0.264411	0.001688	1
Tnfaip3	0.263802	0.026266	1
Ric1	0.262129	0.000698	1
Abcb1b	0.261777	0.008766	1
Sema7a	0.261186	0.00014	1
Frmd8	0.260917	0.007438	1
Rpl13a	0.26067	1.13E-05	0.363694
Cycs	0.260298	0.000469	1
Lamtor4	0.259975	0.000687	1
Sfi1	0.259738	0.002313	1
Ifih1	0.259698	9.11E-06	0.293957
Ubr4	0.258806	0.000328	1
Eef1a1	0.258599	1.18E-07	0.003795
Pofut2	0.257924	0.002704	1
Ckb	0.256916	0.00135	1
D16Ertd472e	0.256419	0.003654	1
Btg2	0.256264	0.016397	1
Serpinb6a	0.256262	0.012877	1
Rsad2	0.255964	0.01394	1
Gbp5	0.255848	0.00127	1
Gimap4	0.25486	0.00232	1
Nfkbid	0.254229	0.208389	1
Lztfl1	0.254013	0.001969	1
Mif4gd	0.253556	0.002139	1
mt-Co2	0.252388	5.95E-05	1
Atp5g1	0.251965	0.002005	1
Cln3	0.251961	0.005906	1
Rnf181	0.250797	0.019874	1
Rtf2	0.250423	0.005244	1
Trmt1	0.250172	0.000736	1
Sntb1	0.249555	0.095687	1
Tmem50a	0.249004	0.000374	1
Dpm1	0.248956	0.012096	1
Llph	0.248819	0.006904	1
Fam53a	0.248809	0.00216	1
Idnk	0.248793	0.002502	1
Fyco1	0.248397	0.053558	1
Mndal	0.248383	0.005322	1
mt-Co1	0.248317	6.38E-06	0.206032

Irak2	0.248266	0.020802	1
Rbm17	0.247806	0.002439	1
Cd8a	0.247737	0.779781	1
Ifi203	0.247591	0.017411	1
Cyld	0.246451	0.001673	1
Ppp1r15a	0.245747	0.000681	1
Tmem173	0.245652	0.033656	1
Phc3	0.245377	0.035213	1
Ndufv3	0.245361	0.001711	1
Psmb10	0.245267	0.001298	1
Il12rb1	0.245195	0.003312	1
Itgb3	0.245182	0.002479	1
Ifi204	0.245133	0.00888	1
Serpina3g	0.245012	0.666432	1
Lcn4	0.244382	0.001419	1
Sptssa	0.2442	0.015344	1
Chd7	0.244152	0.031671	1
Pink1	0.244106	0.000608	1
Zfp292	0.243791	0.012093	1
Malat1	0.24354	0.001764	1
Trim12c	0.242701	0.00095	1
Rsrc1	0.242292	0.069594	1
Pecam1	0.242168	0.000267	1
Abr	0.242007	0.005836	1
Pde11a	0.241842	0.000144	1
Gimap6	0.241754	0.013828	1
BC029722	0.240806	0.001308	1
Smg1	0.24051	0.019366	1
Tmbim4	0.240057	6.47E-05	1
Mcrip1	0.239925	0.006444	1
Zc3h7a	0.239626	0.564199	1
Rps3a1	0.239434	4.28E-06	0.138122
Klrc1	0.239349	0.003054	1
Rundc3b	0.239322	0.000843	1
Rps3	0.239233	1.42E-08	0.000459
Rps19	0.239144	0.000329	1
Rpl22	0.238941	1.15E-05	0.371452
Cmpk2	0.238933	0.008854	1
Supt4a	0.238856	0.00908	1
Clec2d	0.237917	0.01103	1
Hgsnat	0.23777	0.001605	1
Got2	0.237032	0.004039	1
St14	0.236241	0.003128	1
Trappc9	0.236226	0.003585	1
Kdm2b	0.236142	0.070082	1
Rnf145	0.235406	0.012299	1
Fam20a	0.235317	3.58E-05	1
Raly	0.235269	0.005643	1

Pitpna	0.235242	0.012178	1
Ppm1h	0.234577	0.038327	1
Rnf19a	0.234523	0.124874	1
Zap70	0.234413	0.037822	1
Cst7	0.234107	0.015413	1
Aars	0.23362	0.025572	1
Eif3k	0.233071	0.001574	1
Kbtbd11	0.232388	0.028682	1
Hid1	0.232056	0.001132	1
Casp4	0.231785	0.014887	1
Cdk17	0.231699	0.018476	1
Adcy7	0.231479	0.020301	1
Tasor2	0.231471	0.029739	1
Gbp3	0.230586	0.000472	1
Ramp1	0.230478	0.006916	1
Rpl23a	0.230395	0.002065	1
Dkkl1	0.230175	0.001686	1
Ddit3	0.229616	0.004209	1
Mitd1	0.229482	0.051582	1
Nsun4	0.229424	0.000463	1
Smyd1	0.229348	0.007634	1
Ppp1r16b	0.228872	0.066336	1
Nfkb2	0.228367	0.007099	1
Psma3	0.228189	0.000488	1
Klrb1b	0.228171	0.00163	1
Mapre2	0.22791	0.022175	1
Rpl37	0.2279	1.54E-06	0.049583
Cflar	0.227037	0.001006	1
Rps27	0.226441	0.000328	1
Tprg	0.226225	0.011363	1
Gstm5	0.225792	0.000291	1
Srp72	0.22455	0.004348	1
Cpd	0.224513	0.000832	1
Mt2	0.22445	0.000847	1
Abhd18	0.224422	0.001097	1
Malt1	0.223796	0.118629	1
Sqstm1	0.22371	0.015189	1
Tomm20	0.223436	0.009944	1
Trbv12-2	0.223303	0.003842	1
Rnf114	0.222855	0.020835	1
Irf9	0.222833	0.013627	1
Slfn5	0.222774	0.000933	1
Cmip	0.222761	0.04837	1
Eno1b	0.221684	0.018878	1
Fdx2	0.221585	0.003769	1
Trak2	0.221429	0.002791	1
Cdkal1	0.221319	0.011833	1
Usp18	0.221275	0.024246	1

Fam174a	0.221126	0.000982	1
Ttc39c	0.220959	0.004419	1
Rilpl2	0.220861	0.078373	1
Eprs	0.220701	0.003501	1
Nosip	0.219666	0.008417	1
Plcg1	0.21955	0.008801	1
Ndufv1	0.219244	0.026079	1
Sp110	0.219063	0.049799	1
Plekhm3	0.218852	0.000955	1
Cd247	0.218387	0.03022	1
Gdpd5	0.218316	0.000759	1
Rpl7	0.218134	7.12E-05	1
Hspe1	0.217659	0.001068	1
Scand1	0.21733	0.006375	1
Gsto1	0.216987	0.003788	1
H2-T24	0.21666	0.010219	1
Hddc2	0.216348	0.006672	1
Wdfy1	0.216287	0.03067	1
Ext1	0.216108	0.027354	1
Lcp2	0.215877	0.025904	1
Gm12185	0.215473	0.065607	1
Trim30d	0.2151	0.041276	1
Birc6	0.2151	0.028601	1
Gm20400	0.214627	0.024255	1
Camk1d	0.214417	0.423069	1
Bhlhe40	0.213349	0.024518	1
Tuba4a	0.213307	0.01389	1
Ly6g5b	0.213293	0.000603	1
Asb2	0.212722	0.036134	1
Uqcrh	0.212591	0.002864	1
Sbds	0.212575	0.007889	1
AC149090.1	0.212386	0.163848	1
Ern1	0.212214	0.004734	1
Rpl22l1	0.212111	0.001922	1
Hspa5	0.211979	0.021169	1
Ppp1r12a	0.211939	0.081078	1
Tespa1	0.211853	0.110519	1
Eif2s2	0.211204	0.008351	1
Prxl2a	0.211107	0.0003	1
Aebp2	0.211029	0.027017	1
Rps6	0.210681	0.007307	1
Srpk1	0.210233	0.018254	1
Cmtm7	0.210201	0.085967	1
Tmem241	0.209896	0.001419	1
Max	0.209869	0.039635	1
Selenos	0.209854	0.011433	1
Aprt	0.209422	0.017867	1
Pcmtd1	0.209095	0.043914	1

Alcam	0.208741	0.129365	1
Znrf1	0.208697	0.024451	1
Naca	0.20853	0.000365	1
Rabgap1l	0.208486	0.559109	1
Atp2b4	0.208316	0.026363	1
Txn2	0.207384	0.004726	1
2610035D17	0.207365	0.005455	1
Slc39a11	0.206247	0.003868	1
Tor4a	0.206104	0.011102	1
Sec22a	0.206072	0.001283	1
Ldha	0.205776	0.01674	1
Hhat	0.205262	0.000108	1
Rrp1b	0.205259	0.005195	1
Lta	0.204378	0.072699	1
Rasgrp1	0.204375	0.067462	1
S100a6	0.204262	0.048174	1
Cox5a	0.204163	0.015009	1
Mlxip	0.204141	0.050366	1
Mier1	0.20404	0.001669	1
Akap13	0.20378	0.009767	1
Clip1	0.203542	0.003075	1
Psmb3	0.203346	0.009705	1
Ccdc85b	0.203184	0.055443	1
Kdm4d	0.202868	0.000479	1
Mov10	0.202809	0.031433	1
Iigp1	0.202669	5.07E-06	0.163694
Spry1	0.202389	0.000662	1
Kmt5b	0.20226	0.042779	1
2810013P06I	0.201906	0.028365	1
Chrac1	0.201262	0.006688	1
Ostf1	0.201208	0.022403	1
Got1	0.201114	0.02051	1
Smpd5	0.200946	0.013336	1
Vmn2r97	0.200735	0.001352	1
Gimap9	0.200526	0.029499	1
Kdm5b	0.200298	0.003843	1
Bbx	0.200253	0.026005	1
Rpl37a	0.200139	4.89E-05	1
Tmub1	0.199737	0.002929	1
Acot13	0.199547	0.005875	1
Vax2	0.198933	0.001316	1
Sla	0.198859	0.003412	1
Slc25a17	0.198636	0.058647	1
Crtam	0.198173	0.238985	1
Ece1	0.197651	0.117862	1
Capza2	0.197227	0.008177	1
Tvp23b	0.197097	0.007341	1
Nenf	0.196907	0.005138	1

Eif3i	0.196387	0.00726	1
Mapkapk2	0.196282	0.022068	1
Asb13	0.19628	0.003302	1
Ccz1	0.196215	0.045051	1
Dusp1	0.196156	0.907573	1
Spats2	0.196112	0.000249	1
Akr1a1	0.195702	0.014198	1
Pim1	0.195675	0.143048	1
Yy1	0.194649	0.03512	1
Ifi206	0.19419	0.221348	1
Cox7a2l	0.193885	0.014342	1
Zcchc7	0.19361	0.034867	1
Hcfc1r1	0.193544	0.087469	1
Actr10	0.193456	0.040194	1
Pycr2	0.193314	0.014537	1
Zc3h12a	0.192836	0.02412	1
Zfp53	0.192826	0.015821	1
Saysd1	0.192726	0.086184	1
Arhgap10	0.192689	0.088117	1
Galm	0.192577	0.005503	1
Stk17b	0.192525	0.020885	1
B4galnt4	0.192412	0.000369	1
Cd28	0.192234	0.036549	1
Acp1	0.192025	0.027298	1
Asb3	0.191861	0.058073	1
Nfkbib	0.191797	0.072774	1
Aph1a	0.191307	0.016228	1
Gm19705	0.190397	0.000486	1
Chd6	0.189907	0.043574	1
Micu2	0.189831	0.00279	1
Adam8	0.189498	0.040144	1
Gmds	0.189304	0.035408	1
Parp10	0.189234	0.041474	1
Aen	0.189141	0.035087	1
Agap2	0.188741	0.011858	1
Gimap5	0.188687	0.072622	1
Mast4	0.188317	0.000438	1
Ola1	0.188232	0.023254	1
Ubr1	0.187924	0.026612	1
Gpr132	0.18787	0.053353	1
Ptpn6	0.187669	5.13E-05	1
Nop53	0.187388	0.038862	1
Snx20	0.187367	0.008374	1
Mbd2	0.187063	0.016008	1
Smim10l1	0.187018	0.028509	1
Fam189b	0.186837	0.191222	1
Xlr4b	0.186747	0.024075	1
Soat1	0.186584	0.006541	1

Zfp1	0.186354	0.013597	1
Hibadh	0.186345	0.025893	1
Rnf125	0.186326	0.061673	1
Wwox	0.186209	0.028073	1
Zc3h6	0.18608	0.019948	1
Ufc1	0.186021	0.026525	1
Ifit2	0.185935	0.552854	1
Sorl1	0.185133	0.036195	1
Tmem192	0.185099	0.008468	1
Gm26510	0.184972	0.014399	1
Evi2a	0.184962	0.043489	1
Mrps6	0.18484	0.011694	1
Trappc6a	0.184528	0.012459	1
Rab11fip1	0.184432	0.021621	1
Mdn1	0.184418	0.02025	1
Exph5	0.184415	0.000587	1
Hdac2	0.184214	0.01909	1
Vmp1	0.1842	0.008921	1
Trnau1ap	0.184098	0.015409	1
Itgb7	0.18409	0.027466	1
Hspa8	0.184029	0.002139	1
Serbp1	0.18385	0.012482	1
Gm20559	0.183842	0.017615	1
Gstt2	0.183767	0.015417	1
Zc3h13	0.183722	0.078281	1
Gadd45b	0.183462	0.008577	1
Ncor2	0.183407	0.015661	1
Fam169b	0.183256	0.066849	1
Gm11361	0.183169	0.000761	1
Gm20275	0.182986	0.21823	1
Rrp1	0.182661	0.003833	1
Rsbn1	0.181891	0.039676	1
Desi2	0.181205	0.037864	1
Tmem189	0.181191	0.009516	1
Ythdc1	0.180788	0.131312	1
B230219D22	0.180668	0.014834	1
Il3ra	0.180578	0.023419	1
Ssbp4	0.180129	0.011204	1
Usf2	0.180051	0.001824	1
Ptpn1	0.180044	0.011067	1
Phb2	0.179816	0.004537	1
Slain1	0.179787	0.051638	1
Impdh2	0.179203	0.034117	1
Ppm1k	0.178909	0.007566	1
Eva1b	0.178592	0.097598	1
Slc39a14	0.178371	0.001981	1
Rabggtb	0.178361	0.063201	1
Asns	0.17808	0.001931	1

Setd3	0.177842	0.023194	1
Ppp1cc	0.177771	0.0098	1
Mrpl9	0.177511	0.028913	1
Bod1l	0.177287	0.08007	1
Aim2	0.176953	0.08239	1
Sgms1	0.176908	0.010747	1
Dctn2	0.176756	0.035866	1
Tmed2	0.176497	0.048035	1
Efr3a	0.176484	0.082076	1
Trim12a	0.175971	0.063054	1
Ifit1bl1	0.175584	9.3E-05	1
Mrpl58	0.175549	0.097968	1
Hectd1	0.17527	0.028492	1
1110038F14I	0.174683	0.114112	1
Etv6	0.174579	0.216029	1
Esyt2	0.174076	0.058828	1
Arntl	0.174009	0.040101	1
Arl6ip1	0.173922	0.012907	1
Thy1	0.173367	0.038089	1
Spata6	0.173244	0.021159	1
Fto	0.173238	0.020775	1
Xbp1	0.173196	0.029119	1
Sntb2	0.173101	0.00777	1
Ptpn11	0.173027	0.151098	1
Galnt10	0.173021	0.026004	1
Zc3hav1	0.172922	0.036879	1
Pip4k2a	0.172827	0.050044	1
Il27ra	0.172737	0.03799	1
Rpl28	0.172672	0.04616	1
Rab5if	0.172557	0.061521	1
Rnf166	0.172306	0.017319	1
Arid5b	0.172279	0.435331	1
Sigirr	0.171962	0.042562	1
Tmem251	0.171846	0.002181	1
Smad7	0.171784	0.015345	1
Tspan5	0.171663	0.025397	1
Psma2	0.171352	0.030135	1
Sytl1	0.171328	0.000171	1
Ubxn2a	0.170766	0.090211	1
Denr	0.170218	0.044884	1
Phyh	0.170109	0.087199	1
Pgap2	0.170095	0.0743	1
Apopt1	0.169937	0.040196	1
Susd3	0.169913	0.015462	1
Slc48a1	0.169762	0.019862	1
Fam162a	0.169553	0.030621	1
Nubp2	0.169522	0.06597	1
Dock8	0.169465	0.083331	1

Rasal3	0.169454	0.026028	1
Rest	0.169302	0.050128	1
Ech1	0.169183	0.060544	1
Tmem38b	0.169133	0.008951	1
Rpl36al	0.169014	0.011039	1
Btbd19	0.168592	0.097404	1
Trim14	0.168547	0.015013	1
Psma7	0.168515	0.026379	1
Hacd3	0.168491	0.136209	1
Atp5l	0.167878	0.005552	1
Ssh2	0.167807	0.052768	1
Serhl	0.167764	0.031085	1
Lsm14a	0.167604	0.014627	1
Trim21	0.167535	0.252308	1
Pik3ca	0.167483	0.026453	1
Farp1	0.167455	0.139477	1
Mpzl3	0.167441	0.00093	1
Golga5	0.167215	0.035784	1
Slk	0.166899	0.157298	1
Sdf2	0.166819	0.058258	1
Nab1	0.166794	0.295301	1
Tmem191c	0.165958	0.006079	1
Fam120c	0.165933	0.018522	1
Metrnl	0.165586	0.0523	1
Mpv17l2	0.165513	0.051005	1
Rnf5	0.164935	0.058043	1
Cry1	0.164478	0.02106	1
Zcchc2	0.164443	0.027869	1
Dock9	0.164383	0.16009	1
Cox14	0.164366	0.078266	1
Nars	0.16426	0.277573	1
Nup153	0.164195	0.055606	1
Btbd11	0.163894	0.092874	1
Dcaf8	0.163731	0.044148	1
Sart1	0.16367	0.050858	1
Epb41l4aos	0.163484	0.006946	1
Samd10	0.163108	0.0071	1
Ndufs7	0.163103	0.002048	1
Rpl35a	0.163079	0.00037	1
Fam32a	0.162973	0.125953	1
Mat2b	0.162747	0.030724	1
Mir155hg	0.162639	0.248212	1
Zfp945	0.16225	0.074478	1
Ssu72	0.1619	0.05993	1
Tet3	0.161896	0.058857	1
Gm49417	0.161857	0.060625	1
Grhpr	0.16184	0.035979	1
Dusp6	0.161831	0.078637	1

Get4	0.161626	0.059635	1
Znhit2	0.161583	0.002438	1
Mfsd6	0.161539	0.010794	1
Ndufa6	0.161535	0.046063	1
Txnrd2	0.161515	0.020505	1
Polr3k	0.161171	0.087775	1
Cotl1	0.161118	0.158151	1
4833438C02I	0.160897	0.016998	1
Ppp5c	0.160547	0.159347	1
Mcts1	0.160408	0.046177	1
Ssr4	0.160273	0.020392	1
Ddx6	0.159887	0.093825	1
Xpot	0.159878	0.007688	1
Tut4	0.1597	0.150565	1
Pithd1	0.159694	0.005103	1
Cpne3	0.159486	0.005953	1
Ubald1	0.159404	0.018096	1
Parp8	0.159391	0.090376	1
Odc1	0.159205	0.141465	1
Fancc	0.159136	0.046618	1
Snd1	0.158792	0.04194	1
Filip1	0.158743	0.006968	1
Camk4	0.158615	0.112243	1
Erich1	0.158584	0.197745	1
Tmem131	0.158563	0.018725	1
Helz2	0.158162	0.101641	1
Gngt2	0.157991	0.019408	1
Sdhd	0.157966	0.062242	1
Slc7a5	0.157685	0.024202	1
Lrrc61	0.157556	0.008144	1
Fyb	0.157552	0.061356	1
Card6	0.157291	0.073996	1
Eya2	0.157224	0.008451	1
Ctdnep1	0.157004	0.136524	1
Mmadhc	0.157	0.056888	1
Foxj3	0.15686	0.023594	1
Ahr	0.156807	0.032915	1
Gtpbp2	0.156698	0.048319	1
Dynlt3	0.156662	0.007926	1
Cdk5rap1	0.156421	0.01677	1
Rmnd5b	0.156067	0.055142	1
Qrfp	0.155965	0.101196	1
Mapk14	0.155889	0.068467	1
Crbn	0.155694	0.19713	1
Tnfsf11	0.155608	0.009126	1
Tesc	0.155595	0.015112	1
Dennd4a	0.155373	0.073311	1
Las1l	0.155098	0.142584	1

Eif4e3	0.155095	0.053312	1
2310011J03F	0.154881	0.087457	1
Rala	0.154737	0.043264	1
Tpr	0.154686	0.110123	1
Chordc1	0.154615	0.158918	1
Phf21a	0.154517	0.082237	1
Il15ra	0.154419	0.071931	1
Snhg1	0.154413	0.052857	1
BC031181	0.154388	0.104651	1
Rab5c	0.154189	0.170993	1
Hspa9	0.154122	0.325959	1
Araf	0.153956	0.124242	1
Pcgf5	0.153954	0.056354	1
Tacc1	0.153822	0.093321	1
Fis1	0.153653	0.049946	1
2900041M22	0.153541	0.002962	1
Gadd45gip1	0.15346	0.0329	1
Foxp4	0.152976	0.11127	1
Eif3m	0.152537	0.00908	1
Pdhb	0.152533	0.092314	1
Ppt2	0.151893	0.019738	1
Focad	0.151879	0.113167	1
Tnfsf14	0.151838	0.032282	1
Skap1	0.15172	0.09303	1
Oxa1l	0.151704	0.031016	1
Apex1	0.151611	0.023825	1
Grap2	0.151424	0.130658	1
Mfng	0.150774	0.100733	1
Fbl	0.150704	0.172962	1
Pgam5	0.150556	0.03993	1
Tmem39a	0.150503	0.07702	1
Crnkl1	0.150497	0.102522	1
Trib2	0.15004	0.008123	1
Gch1	0.150021	0.118176	1
H2-Ke6	0.150019	0.070964	1
Cox7b	0.149982	0.023578	1
Ttc39b	0.149897	0.230808	1
Rrs1	0.149625	0.024148	1
Sun2	0.149269	0.133933	1
Ms4a6c	0.149207	0.210021	1
Bnip3l	0.14895	0.046682	1
Cd3e	0.148599	0.251752	1
Oprm1	0.148565	0.013936	1
mt-Nd1	0.148481	0.138163	1
Gm11707	0.148475	0.039366	1
Rhbdd1	0.148402	0.110892	1
Dnajc5	0.14831	0.07146	1
Sod1	0.147962	0.069994	1

Ccdc28b	0.147522	0.040672	1
Gm49359	0.147179	0.034719	1
Eci2	0.147146	0.076736	1
Hilpda	0.147136	0.007	1
Izumo4	0.147116	0.167425	1
Caprin1	0.14705	0.082793	1
Phf11c	0.147014	0.421344	1
Nt5m	0.146856	0.01452	1
Tmem140	0.14672	0.158739	1
Atpaf1	0.146633	0.043634	1
Wdr83os	0.14618	0.10365	1
Ell2	0.14597	0.004294	1
Chchd3	0.14593	0.013722	1
Wdr7	0.145912	0.04847	1
Cltb	0.14591	0.1587	1
Orc3	0.145801	0.097917	1
Dgat1	0.145553	0.054659	1
AI662270	0.145476	0.161684	1
Rps15	0.145428	0.070146	1
Leprotl1	0.14528	0.039535	1
Dennd2d	0.144985	0.04798	1
Rtraf	0.144751	0.092907	1
Ifi214	0.144738	0.24934	1
Pdhx	0.144588	0.007503	1
Ldah	0.14443	0.151228	1
Nucb1	0.144398	0.106583	1
Phax	0.144393	0.031551	1
Matk	0.144363	0.004797	1
Capg	0.144306	0.060877	1
Rab43	0.144279	0.003819	1
Prr13	0.144165	0.019815	1
Nub1	0.144141	0.09455	1
Acpp	0.144071	0.008706	1
Atg3	0.144037	0.131368	1
Zdhhc18	0.144016	0.080708	1
Vps13d	0.143961	0.193518	1
Uri1	0.143851	0.132836	1
Foxo3	0.14384	0.055207	1
Plaat3	0.14381	0.00879	1
Usp48	0.143668	0.266139	1
Pxn	0.143578	0.061251	1
Parl	0.143558	0.036469	1
Nfatc2	0.143546	0.323864	1
Kiz	0.143481	0.021189	1
Ccdc92	0.143399	0.022811	1
Dph6	0.14313	0.175715	1
P4ha1	0.142901	0.06585	1
Senp6	0.142851	0.094427	1

Fbxo38	0.142848	0.022103	1
Cep120	0.142842	0.227127	1
Selenok	0.1428	0.114676	1
Ptprj	0.142775	0.304731	1
1500011B03I	0.142713	0.046493	1
Clcc1	0.142536	0.076381	1
Ndufaf4	0.142433	0.063772	1
D5Ertd579e	0.142376	0.094837	1
Selenoo	0.142255	0.043717	1
Exosc4	0.142063	0.004435	1
Adamts6	0.142041	0.331967	1
Cir1	0.141656	0.072411	1
Plcl1	0.141608	0.010404	1
Plekho2	0.141343	0.131447	1
Rinl	0.141176	0.305404	1
Tulp4	0.141092	0.18387	1
Man2c1	0.14101	0.038482	1
Eefsec	0.140936	0.081529	1
Yipf2	0.14084	0.002192	1
Crtc2	0.140802	0.25184	1
Fam53b	0.140784	0.059928	1
Ezh1	0.140638	0.095893	1
Chchd4	0.140633	0.018206	1
Bdh1	0.140371	0.052372	1
Zfp397	0.140211	0.043468	1
Pigx	0.140105	0.03156	1
Mtmr1	0.14009	0.044735	1
Spint2	0.140024	0.227803	1
Dnajc3	0.139923	0.097788	1
Zfp672	0.139799	0.127022	1
Csnk2b	0.139646	0.108037	1
Mtfr1l	0.139525	0.100071	1
Coq10b	0.139109	0.116698	1
Stk19	0.139004	0.054443	1
Mbp	0.138931	0.110951	1
Wwp1	0.138916	0.025443	1
Gpr65	0.138769	0.29532	1
0610012G03	0.138674	0.015579	1
Mrpl37	0.138532	0.094681	1
Med13l	0.13853	0.085682	1
Rassf5	0.138508	0.241675	1
Agfg1	0.13843	0.19943	1
Akr7a5	0.138413	0.034928	1
Scamp3	0.138323	0.018595	1
Sh2b1	0.138071	0.023521	1
Snhg8	0.138012	0.046684	1
Zfp683	0.137887	0.02755	1
Aar2	0.137689	0.069789	1

Ago2	0.137255	0.261589	1
Nkiras1	0.137211	0.013302	1
Utp4	0.137054	0.203815	1
Abhd12	0.136914	0.046013	1
Tk2	0.13691	0.062883	1
Ginm1	0.136894	0.112771	1
Asap1	0.136859	0.462596	1
Ddx41	0.136838	0.254215	1
Ubr5	0.136788	0.152778	1
Dop1b	0.13678	0.236065	1
Aarsd1	0.136749	0.164862	1
Tmem9	0.136682	0.041714	1
Card19	0.136676	0.252931	1
Slc25a30	0.136588	0.022578	1
Uqcc3	0.136557	0.052073	1
Ubn2	0.136513	0.044734	1
Tiparp	0.136254	0.024145	1
Pebp1	0.136065	0.089181	1
L3mbtl3	0.135972	0.177769	1
Sars	0.135866	0.314931	1
Arf3	0.135689	0.051328	1
Commd8	0.135588	0.338757	1
Noc4l	0.135503	0.067882	1
Mdp1	0.135444	0.163488	1
Cyp4f13	0.135414	0.030401	1
Daxx	0.135078	0.035928	1
Sec24b	0.135033	0.068072	1
Mrps26	0.134784	0.110116	1
2410002F23I	0.134752	0.112891	1
Aig1	0.134672	0.044409	1
Insig1	0.134602	0.011678	1
Ocel1	0.134591	0.030116	1
Crem	0.134145	0.060887	1
Asb8	0.134079	0.078576	1
Uqcr10	0.134004	0.052059	1
Fmnl3	0.133999	0.258273	1
Pbx3	0.13384	0.033481	1
mt-Nd5	0.133812	0.121001	1
Pin4	0.13361	0.216532	1
Tollip	0.133605	0.06389	1
Nprl3	0.133516	0.0871	1
Xpo4	0.133485	0.192172	1
Clk2	0.133365	0.019129	1
Hbp1	0.133358	0.01166	1
Sar1b	0.133187	0.267423	1
Snx29	0.133013	0.264769	1
Emc7	0.132996	0.240106	1
Elavl1	0.132927	0.063253	1

Bclaf1	0.132916	0.118308	1
Pde7a	0.132828	0.710993	1
Gpatch4	0.132791	0.026654	1
Dnttip1	0.13261	0.011656	1
Myo3b	0.132568	0.237383	1
Abi2	0.132506	0.071017	1
Fut8	0.132192	0.157623	1
Stxbp5	0.132181	0.129798	1
Chka	0.13217	0.057695	1
Usp15	0.131536	0.311724	1
Ssbp2	0.131341	0.005393	1
Eif4ebp1	0.131243	0.025689	1
Zmynd8	0.13121	0.139801	1
Ifrd1	0.131174	0.166646	1
Ccdc102a	0.131113	0.025825	1
Tbl1xr1	0.131027	0.244972	1
Kdsr	0.130905	0.115705	1
Usp3	0.130804	0.198583	1
Cox8a	0.129935	0.026302	1
Stx11	0.129821	0.153524	1
Ube2v1	0.129648	0.227493	1
Stx6	0.129566	0.270055	1
Prdx5	0.129299	0.039432	1
Bcas3	0.129291	0.33902	1
Ntng2	0.129148	0.018316	1
Eif3d	0.128942	0.12011	1
Trim44	0.12879	0.024063	1
A430093F15I	0.12877	0.532107	1
Vps8	0.128698	0.100676	1
Ms4a6d	0.128683	0.089636	1
Fkrp	0.128565	0.011145	1
Phf20l1	0.128471	0.059852	1
Elmsan1	0.128462	0.040693	1
Chmp1b	0.128418	0.048362	1
Uvssa	0.128387	0.005864	1
Ndufb9	0.128322	0.125107	1
Npepps	0.128189	0.361137	1
Snhg20	0.128165	0.057803	1
Taf3	0.128116	0.283468	1
Rnf187	0.128101	0.085408	1
Gabarapl2	0.127966	0.015186	1
Poglut1	0.127841	0.01262	1
Zfp414	0.127551	0.163799	1
Ucp2	0.127415	0.12016	1
Spr	0.127288	0.257476	1
Gfer	0.127161	0.223616	1
Lars	0.126853	0.116466	1
Mycbp2	0.126761	0.046378	1

Map2k3	0.126705	0.474805	1
Slc4a7	0.126207	0.111428	1
Phtf1	0.126174	0.607037	1
2310015A10I	0.126121	0.004699	1
Cntrl	0.126018	0.432187	1
BC147527	0.125677	0.131105	1
Rhbdd2	0.125631	0.03162	1
Ubl7	0.125363	0.085181	1
9930021J03F	0.125324	0.153498	1
Mtln	0.125187	0.09165	1
Cln6	0.12505	0.011454	1
Tmem164	0.124989	0.099678	1
Zc3h12d	0.124726	0.007147	1
F2r	0.124619	0.149823	1
Fuom	0.12458	0.017201	1
Herc3	0.124576	0.143736	1
Gm47283	0.124534	0.063652	1
Dcaf6	0.124497	0.060055	1
Ksr1	0.124497	0.34046	1
Hsp90aa1	0.124495	0.204078	1
Yars	0.124481	0.304057	1
Pus10	0.124396	0.01534	1
Ino80	0.124376	0.110205	1
Ccr2	0.124329	0.068337	1
Rc3h2	0.124295	0.168498	1
Ggct	0.124116	0.159829	1
Vapa	0.123968	0.254423	1
Fnbp1	0.123962	0.158702	1
Dmtf1	0.123946	0.484979	1
Retreg1	0.123853	0.090035	1
Exosc7	0.123793	0.31658	1
Rps28	0.123679	0.019021	1
Fbxo34	0.123627	0.009478	1
Exoc6b	0.123579	0.213042	1
Lyrm9	0.123377	0.001356	1
Cluh	0.123339	0.005334	1
Nol7	0.1233	0.099229	1
Psme4	0.123237	0.139259	1
Pdcd2	0.123221	0.088909	1
Rad23b	0.123196	0.309991	1
Gyg	0.12313	0.123213	1
Pip4k2b	0.122936	0.16164	1
Stx16	0.12293	0.13658	1
Prpf18	0.122911	0.015484	1
Dnajb13	0.122895	0.04074	1
Eno1	0.122776	0.110682	1
Fam49a	0.122744	0.074592	1
Arhgef12	0.122584	0.011698	1

Map3k8	0.12249	0.243622	1
Slc35c2	0.122396	0.336486	1
Insl6	0.122279	0.146212	1
Inpp4a	0.122008	0.239985	1
Suclg1	0.121958	0.194026	1
Gypc	0.121859	0.042238	1
Timm50	0.121856	0.103075	1
Gm26887	0.12184	0.087311	1
1700028E10I	0.121793	0.036092	1
1700123O20	0.12174	0.139656	1
Als2	0.12146	0.055233	1
Rnf149	0.121456	0.039291	1
Rab19	0.121318	0.150029	1
Oma1	0.121256	0.226501	1
Rabggta	0.121201	0.253162	1
Nxpe3	0.121134	0.09461	1
Phgdh	0.121083	0.179901	1
Atf2	0.121039	0.057955	1
Uhrf1bp1l	0.120982	0.001025	1
1700021A07I	0.1209	0.017766	1
2310039H08	0.120831	0.063343	1
Slc19a1	0.1208	0.014436	1
Fbxl17	0.120742	0.042256	1
Esf1	0.120708	0.261686	1
Axl	0.120678	0.005884	1
Arl14ep	0.120674	0.021069	1
Ndufaf2	0.120397	0.050073	1
Parp11	0.120309	0.36895	1
Lancl1	0.120293	0.12864	1
Uck2	0.120054	0.251989	1
Slc25a5	0.120029	0.09421	1
Psmg4	0.119974	0.195818	1
Tmem154	0.11991	0.39062	1
Ahi1	0.119796	0.220915	1
Trmt11	0.119699	0.100219	1
Rfx7	0.119691	0.500798	1
Hps3	0.119639	0.096532	1
Epb41	0.119522	0.394708	1
Tlk2	0.119235	0.222413	1
Ppa1	0.118842	0.072006	1
Itpr2	0.118589	0.18557	1
Mrpl42	0.118552	0.204274	1
mt-Co3	0.118547	0.084637	1
Phactr2	0.118544	0.373141	1
Gem	0.118526	0.889314	1
Rnft1	0.118366	0.056767	1
Oser1	0.118318	0.132048	1
Mrpl2	0.118272	0.254803	1

Fam71b	0.118201	0.061947	1
Itgav	0.118098	0.154045	1
Nufip2	0.118047	0.318344	1
Hipk1	0.117944	0.162368	1
Cyb5a	0.117909	0.03062	1
Tmem134	0.117885	0.161712	1
Gna15	0.117882	0.091556	1
Irak4	0.117736	0.068092	1
Pgm1	0.117669	0.078218	1
Pomp	0.11765	0.19147	1
Slc9a9	0.117612	0.35097	1
Vps13b	0.117561	0.067688	1
Prps1	0.117512	0.215263	1
Taf8	0.117448	0.193104	1
Abcf3	0.117103	0.079888	1
Nop14	0.117001	0.175166	1
Gpr160	0.116672	0.085162	1
5430405H02	0.116575	0.096502	1
Acot9	0.116337	0.154092	1
Car5b	0.116262	0.161255	1
Gas2	0.116101	0.019312	1
Utp11	0.11606	0.257327	1
Riox2	0.116052	0.186881	1
Bag4	0.115898	0.075942	1
Rai1	0.115653	0.123833	1
Gnl3	0.115502	0.345904	1
Tbc1d15	0.115498	0.224667	1
Ralgapa2	0.115452	0.256743	1
Pex16	0.115397	0.352538	1
Ahcyl1	0.115296	0.112025	1
Ttpal	0.115252	0.386067	1
N4bp1	0.115126	0.032929	1
Invs	0.115087	0.209188	1
Atg10	0.114953	0.331127	1
Ccdc58	0.114929	0.023488	1
Nol12	0.114851	0.203753	1
Phip	0.11463	0.129714	1
Nmrk1	0.114586	0.079739	1
Scyl3	0.114576	0.245725	1
Mgst2	0.114506	0.153207	1
Mzt2	0.11448	0.011551	1
Hip1	0.114385	0.240794	1
Lck	0.114341	0.094879	1
Tusc2	0.114313	0.056321	1
Ttc1	0.114187	0.288513	1
Spata5	0.113963	0.061221	1
Larp1	0.11383	0.396027	1
F8a	0.113811	0.364689	1

Clock	0.113769	0.085311	1
Hagh	0.113565	0.190033	1
Fam204a	0.113278	0.383797	1
1600002K03I	0.113209	0.039606	1
Pih1d1	0.113044	0.201734	1
B4galnt1	0.113008	0.294663	1
Nif3l1	0.112979	0.083586	1
Rpap2	0.11284	0.049412	1
Cdk7	0.11283	0.141589	1
Lpin2	0.112826	0.240628	1
Ints11	0.112693	0.187097	1
Ip6k1	0.112641	0.10662	1
Zrsr2	0.11259	0.185784	1
1190007I07F	0.11241	0.167513	1
Hmg20b	0.112402	0.150319	1
Trim24	0.112372	0.434101	1
Gde1	0.112355	0.206598	1
Hs3st3b1	0.112166	0.014648	1
Parp4	0.112044	0.154498	1
Mtx2	0.111593	0.302847	1
Cracr2a	0.111578	0.3701	1
Mesd	0.111373	0.086812	1
Snip1	0.111301	0.171514	1
Tor3a	0.111129	0.231636	1
Mrps28	0.110955	0.146068	1
Gimap1	0.110917	0.149439	1
L3mbtl2	0.110714	0.201981	1
Csnk1g1	0.110507	0.174525	1
Ino80b	0.110444	0.150353	1
Tox4	0.110364	0.288871	1
Clta	0.110227	0.05031	1
Picalm	0.110175	0.43153	1
Abhd4	0.110097	0.014548	1
Prelid1	0.109837	0.209271	1
Rwdd4a	0.109713	0.151863	1
Ptpn3	0.109655	0.075092	1
Marveld2	0.109545	0.098515	1
Lrif1	0.109515	0.143171	1
Tubgcp2	0.109476	0.279601	1
Dpy19l3	0.109275	0.116092	1
Slc30a9	0.109195	0.291556	1
Atf4	0.109168	0.489311	1
AY036118	0.109144	0.334454	1
Surf1	0.109098	0.008329	1
Klhl36	0.108927	0.137664	1
Ncoa2	0.108873	0.25721	1
Gnl2	0.108866	0.191859	1
Tsn	0.108663	0.363163	1

Tbrg1	0.108592	0.00989	1
Tmem160	0.108265	0.052895	1
Zcchc17	0.108243	0.454263	1
Pim3	0.10822	0.035497	1
Fbxl20	0.108201	0.016766	1
Chmp2a	0.108096	0.230123	1
Zer1	0.108071	0.07764	1
Alg8	0.108041	0.050425	1
Cnppd1	0.107769	0.053303	1
H2-T22	0.107734	0.210732	1
Ppp1r9b	0.107673	0.170211	1
Arfrp1	0.107638	0.172293	1
Glrx5	0.107632	0.004883	1
Mtfmt	0.107533	0.071915	1
Ripk2	0.107507	0.09942	1
Rngtt	0.107465	0.293507	1
Golgb1	0.107348	0.083849	1
Tax1bp1	0.10733	0.021377	1
Stk26	0.107247	0.451689	1
Rc3h1	0.107231	0.100258	1
Galk2	0.107227	0.120443	1
Aup1	0.107103	0.165475	1
Gramd1b	0.106876	0.347496	1
2310061I04F	0.106809	0.206515	1
Rictor	0.106644	0.374203	1
Bcl7b	0.106288	0.401648	1
Phf12	0.106285	0.221602	1
Arfgap1	0.106147	0.129485	1
Zmpste24	0.105965	0.199473	1
Gm2a	0.105871	0.230191	1
Maip1	0.105824	0.083029	1
Rmdn1	0.105762	0.032057	1
Sptlc1	0.105739	0.285059	1
Phkg2	0.105733	0.058828	1
Dalrd3	0.1057	0.046005	1
Chd1	0.105528	0.072054	1
Trim27	0.105318	0.100255	1
Coa7	0.105276	0.055956	1
Eif1a	0.105269	0.094227	1
Cd52	0.105263	0.158527	1
Pgls	0.105226	0.168323	1
Pnpla7	0.105158	0.112111	1
Rbck1	0.105115	0.209131	1
Rnf157	0.105068	0.108577	1
Spsb3	0.104884	0.073067	1
Amn1	0.104865	0.156314	1
St3gal1	0.104782	0.255241	1
Csnk1a1	0.104685	0.326105	1

Lig3	0.10457	0.067494	1
Sil1	0.104541	0.149678	1
Serpinb1a	0.104278	0.067516	1
Rarg	0.10422	0.056558	1
Gm39323	0.104212	0.107698	1
Tmem64	0.104067	0.04211	1
Haghl	0.104049	0.067398	1
Tbcb	0.10392	0.16373	1
Phospho2	0.103904	0.100097	1
Gtf3c2	0.103824	0.170534	1
Zfp593	0.103808	0.215724	1
Lrrc8d	0.103806	0.197336	1
Aurkaip1	0.1038	0.193524	1
Tex264	0.1038	0.460321	1
Wipi2	0.103598	0.164356	1
Sinhcaf	0.103595	0.177342	1
Socs3	0.103446	0.266902	1
A330040F15I	0.103336	0.480408	1
Dnajc19	0.103297	0.410111	1
Tor1aip2	0.103188	0.036931	1
Rnf14	0.102958	0.686404	1
Pcnx	0.102834	0.237188	1
Iars	0.102507	0.33495	1
Clptm1l	0.102498	0.076398	1
Mrrf	0.102341	0.065372	1
Ccnq	0.102313	0.254161	1
Xpr1	0.102273	0.136192	1
Magohb	0.102259	0.228074	1
Lrrc59	0.102218	0.141718	1
Snx15	0.102176	0.094586	1
Creb1	0.102155	0.305885	1
Ptpn7	0.102087	0.296579	1
B4galt5	0.102077	0.302635	1
Meis3	0.102063	0.04685	1
Asf1a	0.102041	0.126552	1
Rbms1	0.101818	0.295606	1
E330009J07F	0.101717	0.036827	1
Ankzf1	0.101533	0.212124	1
Cnot11	0.101506	0.121918	1
Kidins220	0.101493	0.395917	1
Tigd2	0.101391	0.473978	1
Commd6	0.101379	0.119566	1
Cdc123	0.101284	0.335172	1
Rab27a	0.101141	0.34473	1
Sv2c	0.101103	0.071607	1
Celsr1	0.100918	0.178309	1
Emp1	0.100872	0.133841	1
Wdr45b	0.100823	0.564921	1

Rxylt1	0.100777	0.060628	1
Ddx27	0.100547	0.280889	1
Med30	0.100392	0.159455	1
Nolc1	0.100376	0.450983	1
Zfp64	0.100338	0.031896	1
Cmss1	0.100328	0.248546	1
B3gntl1	0.100293	0.174021	1
Actr6	0.100208	0.021096	1
Zxdc	0.100107	0.113841	1
Trps1	0.100033	0.407791	1
Tfrc	0.099987	0.109761	1
A930037H05	0.099965	0.115587	1
2300009A05I	0.099915	0.046087	1
Poc5	0.099888	0.102666	1
Wbp11	0.099839	0.452375	1
Pank2	0.099631	0.134469	1
Slc25a20	0.099577	0.087769	1
Spg7	0.099493	0.063107	1
Idh3b	0.09946	0.404298	1
Dnajb9	0.099392	0.34842	1
Cdk13	0.099107	0.090591	1
Mettl16	0.099036	0.726141	1
Rnh1	0.098941	0.319822	1
F730043M19	0.098868	0.778877	1
Smyd5	0.098856	0.018861	1
Fam210b	0.09884	0.465006	1
Phf6	0.09879	0.319896	1
Nbdy	0.098778	0.067682	1
Plekha3	0.098673	0.173993	1
Arl1	0.09867	0.440321	1
Aak1	0.098657	0.377611	1
F2rl2	0.098641	0.422105	1
Dcaf5	0.09861	0.13812	1
Ccni	0.098607	0.07135	1
Nr4a1	0.098587	0.039251	1
Dnase1l1	0.098506	0.038326	1
Polr3b	0.098471	0.088524	1
Sgsh	0.09843	0.138558	1
Thap7	0.098219	0.309691	1
Furin	0.098208	0.054773	1
Stat5a	0.098198	0.643651	1
Atp10a	0.098162	0.042968	1
Itpkb	0.098149	0.337491	1
Ndufs3	0.09814	0.172903	1
Spata5l1	0.098051	0.097281	1
Dtd2	0.097887	0.118389	1
Tpcn1	0.097871	0.072491	1
Apba3	0.097844	0.116981	1

Mia2	0.097828	0.412808	1
N4bp2l2	0.097807	0.208916	1
Nek6	0.097753	0.262955	1
Tob2	0.097517	0.354372	1
Ccnc	0.097515	0.249863	1
Tmem203	0.097468	0.068025	1
Faf2	0.097434	0.13805	1
Alkbh3	0.097407	0.28998	1
Nanp	0.09732	0.224092	1
Zfp110	0.097267	0.146645	1
Slc37a3	0.097067	0.211908	1
Srek1ip1	0.097015	0.045563	1
Mfap1b	0.096928	0.41222	1
Trerf1	0.096895	0.423473	1
Api5	0.096892	0.520867	1
Setd1a	0.096869	0.220097	1
Ctnna1	0.096842	0.170604	1
Crybg1	0.09668	0.469578	1
Kri1	0.096679	0.485733	1
Angptl4	0.096548	0.114538	1
Ears2	0.096547	0.088034	1
Cdk9	0.096318	0.362135	1
Runx2os1	0.096306	0.065454	1
Gba	0.096166	0.439497	1
2410022M11	0.095906	0.081311	1
Tnip2	0.095709	0.010831	1
Pdk1	0.095678	0.251779	1
Ndufaf1	0.095608	0.104285	1
Taz	0.095568	0.105225	1
Prkar1a	0.095466	0.274933	1
Rbm41	0.095374	0.332824	1
Ptcd3	0.095307	0.195652	1
Mrpl38	0.095244	0.236209	1
Ccnh	0.095166	0.157164	1
6030458C11	0.095149	0.347907	1
Bicdl1	0.095064	0.047724	1
Ddx18	0.095045	0.040783	1
Bcl7c	0.094998	0.585822	1
Suco	0.09497	0.52336	1
Prxl2c	0.094936	0.016866	1
Stk25	0.094922	0.087455	1
Snu13	0.094878	0.405025	1
Cct3	0.094749	0.337793	1
Rfxap	0.0946	0.230421	1
Plcxd2	0.094576	0.275434	1
Ndufa5	0.094545	0.183392	1
Ndufs6	0.094542	0.309277	1
Zbtb2	0.094497	0.221481	1

Uap1l1	0.094495	0.098444	1
Rabepk	0.094276	0.514753	1
CAAA0111183	0.094272	0.382637	1
Fosb	0.094249	0.501015	1
Tbc1d9b	0.09422	0.495927	1
Aacs	0.094206	0.107734	1
Abi3	0.094127	0.103477	1
Mrpl46	0.093998	0.330496	1
Ccdc130	0.093964	0.07467	1
Evl	0.093889	0.188784	1
Ercc6	0.093874	0.056906	1
Zfp280c	0.093851	0.230072	1
Cfap36	0.093742	0.5086	1
Nlrc5	0.093736	0.767867	1
Lrrc45	0.093733	0.060568	1
Unc50	0.093666	0.401882	1
Zdhhc3	0.093599	0.028956	1
Mettl23	0.093434	0.263732	1
1110032A03	0.093391	0.025224	1
Ptpmt1	0.093345	0.222008	1
Usp53	0.093269	0.210426	1
Irf2bp2	0.093214	0.28578	1
Ralgapb	0.093171	0.136174	1
Nol11	0.093164	0.188337	1
Cyb5r1	0.093019	0.104679	1
Stimate	0.093009	0.008765	1
Mrpl45	0.092992	0.023234	1
Ypel5	0.092964	0.207728	1
Phb	0.092884	0.321988	1
Eif2ak1	0.092759	0.256028	1
Fbxl3	0.092732	0.194298	1
Dgcr2	0.09266	0.188564	1
Hsd17b12	0.092595	0.705857	1
Ddx3x	0.092547	0.139502	1
Casc3	0.092518	0.320181	1
Npm3	0.092355	0.295199	1
Micu3	0.092178	0.264134	1
Sclt1	0.092146	0.313113	1
Rwdd1	0.092071	0.521686	1
Ubac1	0.091975	0.152942	1
Mtif2	0.091945	0.243473	1
Sumf1	0.09189	0.140527	1
Ndufb6	0.091879	0.431372	1
Trmt112	0.091845	0.222747	1
Guk1	0.091802	0.302387	1
Tnrc18	0.091779	0.209402	1
Rab11fip2	0.091737	0.130786	1
Lrrc28	0.091719	0.089629	1

Mbtps1	0.091714	0.196474	1
Ccl3	0.091658	0.006486	1
Mtg2	0.091607	0.38264	1
Abcc1	0.091528	0.106	1
Zfp664	0.09146	0.19394	1
Stam2	0.091357	0.167007	1
Arhgap27os3	0.091179	0.442215	1
Lat	0.091042	0.19595	1
Nufip1	0.090944	0.273025	1
Itgb3bp	0.090912	0.22345	1
Pmm2	0.090814	0.228468	1
Polr3h	0.090747	0.376646	1
Parp12	0.09073	0.15108	1
Pdha1	0.090673	0.228323	1
Mboat7	0.090638	0.502515	1
Ptpn9	0.090623	0.238708	1
Tle5	0.090621	0.154892	1
Ndufab1	0.090593	0.3402	1
Rnf146	0.09042	0.174897	1
Ndufa12	0.090416	0.410275	1
Dph3	0.090345	0.115842	1
Bcl6	0.090337	0.242886	1
Bola3	0.090047	0.179742	1
Ring1	0.090011	0.369175	1
Mrpl14	0.090001	0.291744	1
5530601H04	0.089685	0.135545	1
Dok1	0.089661	0.061009	1
2700062C07	0.089485	0.207133	1
Noa1	0.089423	0.333126	1
N4bp2l1	0.089412	0.566503	1
Srsf5	0.08934	0.33735	1
Fahd2a	0.089333	0.188176	1
Fam120b	0.089316	0.338789	1
Birc3	0.089304	0.022878	1
Alg9	0.08929	0.059485	1
Txndc9	0.089274	0.297431	1
Psmf1	0.089193	0.254578	1
Ankrd37	0.089125	0.321837	1
Ints14	0.089082	0.275075	1
Tbc1d14	0.089071	0.353905	1
Klf13	0.088946	0.192267	1
Tpgs2	0.088869	0.267696	1
Spcs2	0.088823	0.216514	1
Nfat5	0.088768	0.211476	1
Psma4	0.088573	0.274401	1
Taf10	0.088522	0.543394	1
Tmem268	0.088483	0.162333	1
Pold4	0.088404	0.004589	1

Tmem42	0.088362	0.108811	1
Mrpl24	0.088342	0.27294	1
Raet1e	0.088313	0.350402	1
Slco4a1	0.088306	0.045537	1
Dus3l	0.088245	0.469743	1
Yipf4	0.088233	0.31776	1
Cars	0.088231	0.227385	1
Wdr45	0.088199	0.23491	1
Dars	0.088111	0.544914	1
Socs4	0.088024	0.322877	1
Stambpl1	0.087921	0.409842	1
Gne	0.087843	0.665768	1
Gtf3c1	0.087692	0.195077	1
Tmem14c	0.087579	0.264981	1
Poldip2	0.087558	0.092695	1
Rlim	0.087491	0.640442	1
Kdm4b	0.087473	0.094972	1
Psma6	0.087459	0.108683	1
Proser1	0.087453	0.15771	1
Cyc1	0.087415	0.114358	1
A430005L14I	0.087399	0.170821	1
Slc2a3	0.087219	0.417611	1
Atxn7	0.087181	0.177948	1
Ccdc86	0.087102	0.214054	1
Rspry1	0.087085	0.399621	1
Bfar	0.086982	0.432022	1
Hdac8	0.086948	0.327817	1
A930024E05I	0.086894	0.19224	1
Hmgxb3	0.086877	0.111802	1
Snx5	0.086825	0.360518	1
Bet1l	0.086812	0.135237	1
Oxld1	0.086808	0.127686	1
Rae1	0.0868	0.268558	1
Abhd2	0.086737	0.330131	1
Map2k4	0.086678	0.460228	1
Rpp14	0.086509	0.093367	1
Dpp4	0.086463	0.368177	1
Lst1	0.086432	0.080773	1
Rad18	0.086398	0.43844	1
Ndst1	0.08631	0.01533	1
Tgif1	0.086178	0.35626	1
Acaca	0.086081	0.257008	1
Ktn1	0.086072	0.25484	1
Zfp810	0.085978	0.535302	1
Adam9	0.085975	0.289264	1
Ccl25	0.08597	0.189652	1
Carnmt1	0.085885	0.141811	1
Mterf3	0.085844	0.016856	1

Lrpprc	0.085809	0.160297	1
Cdc37l1	0.085703	0.164962	1
Taf11	0.085662	0.204001	1
Slc7a6	0.08566	0.348082	1
Pmpcb	0.085636	0.295165	1
C87436	0.085616	0.195025	1
Rab18	0.085542	0.57474	1
Khnyn	0.085421	0.720766	1
Lap3	0.085381	0.088864	1
Mtap	0.085313	0.118799	1
Pknox1	0.085262	0.240323	1
Tor2a	0.085137	0.072361	1
Cdv3	0.085132	0.557449	1
Wdr73	0.085113	0.258143	1
Chd1l	0.085061	0.084473	1
Ydjc	0.084898	0.0521	1
Rcor3	0.084893	0.031681	1
Ctss	0.08483	0.004677	1
Sec24c	0.084793	0.571728	1
Phf11a	0.084707	0.933524	1
Mrpl22	0.084707	0.402304	1
Nabp1	0.084707	0.135822	1
Ggps1	0.084618	0.498966	1
Iscu	0.084592	0.276923	1
Cep164	0.084499	0.224667	1
Gnl3l	0.084481	0.260441	1
Gm4316	0.084474	0.235041	1
Mapk9	0.084458	0.130307	1
Hsh2d	0.084447	0.299727	1
Il1rap	0.084377	0.294707	1
Smco4	0.084294	0.219668	1
Galt	0.084242	0.32755	1
Creb3l2	0.084199	0.072032	1
Zfp408	0.084158	0.197031	1
Rnf7	0.084144	0.472703	1
Mib1	0.0841	0.096896	1
Arfgef1	0.084087	0.262661	1
Zfp87	0.084041	0.1765	1
Padi2	0.08401	0.019602	1
Cabin1	0.083968	0.405944	1
Nit2	0.083835	0.416377	1
Emg1	0.083796	0.472541	1
Uqcrq	0.083748	0.108007	1
Ubqln1	0.083732	0.265448	1
Gtf2b	0.083611	0.690072	1
Zdhhc12	0.083601	0.113879	1
Zdhhc17	0.083526	0.04902	1
Ecd	0.083446	0.555971	1

5430416N02	0.083427	0.246862	1
Slc25a16	0.083399	0.17259	1
Ipo4	0.083245	0.1245	1
Aff4	0.083182	0.079191	1
Clns1a	0.083154	0.206129	1
Prkdc	0.083147	0.170188	1
Gm5547	0.082853	0.153157	1
Atp5f1	0.082754	0.403911	1
Morf4l2	0.082743	0.306838	1
Zfp36l1	0.08272	0.006114	1
Eid1	0.082689	0.146905	1
Trmt6	0.082625	0.178334	1
Pfkfb3	0.082498	0.299762	1
Ercc5	0.082488	0.262032	1
Gm47015	0.082471	0.179047	1
Gmfb	0.082457	0.266021	1
AW549877	0.082446	0.216585	1
Itpk1	0.08243	0.216631	1
Mettl22	0.082396	0.138153	1
Sema4d	0.082354	0.156139	1
Zfp141	0.082274	0.147316	1
Smpd4	0.082243	0.143643	1
Usp45	0.082212	0.235974	1
Rac1	0.082035	0.360148	1
Inpp5k	0.081957	0.108165	1
Gmppa	0.081941	0.351579	1
Slc16a6	0.081811	0.446346	1
Nsmce3	0.081755	0.306263	1
Rusc1	0.081744	0.1297	1
Med28	0.081693	0.482103	1
Paox	0.081675	0.133755	1
Tfdp2	0.081596	0.279648	1
Swt1	0.081545	0.183773	1
Gpaa1	0.081531	0.345797	1
Dpy19l4	0.081506	0.268392	1
Maf1	0.08147	0.123276	1
Tmem184b	0.081432	0.317973	1
Yars2	0.081318	0.050434	1
Oas1c	0.081313	0.474155	1
Hltf	0.08123	0.132279	1
Mthfsl	0.081189	0.409147	1
Arhgef6	0.081159	0.447071	1
Gon4l	0.081142	0.792028	1
Aldh18a1	0.081067	0.528509	1
Gstcd	0.081053	0.22976	1
Anapc13	0.081047	0.487687	1
Dnajc4	0.081015	0.272278	1
Gimap8	0.081003	0.324012	1

Kdm5d	0.080989	0.323749	1
Aptx	0.080876	0.790503	1
Imp4	0.08087	0.662005	1
Vps39	0.08087	0.350102	1
Sgf29	0.080837	0.331547	1
Tpp2	0.080794	0.740195	1
Zswim7	0.080776	0.216567	1
Mrps5	0.080761	0.535684	1
Dclre1b	0.080649	0.33518	1
Xab2	0.080637	0.740634	1
Wsb1	0.080628	0.710089	1
Ccnk	0.080604	0.105764	1
Med9	0.080603	0.525644	1
Rnf139	0.080559	0.127598	1
Cyb5r4	0.080536	0.262277	1
Nme1	0.080459	0.203157	1
Rmnd5a	0.080423	0.100774	1
Zfp644	0.080379	0.193033	1
Fcf1	0.080363	0.207347	1
Rnf113a2	0.080353	0.077468	1
Gm36551	0.080328	0.124467	1
Ndufb3	0.080228	0.53126	1
Calhm6	0.080203	0.57629	1
Cmtr1	0.080157	0.075269	1
Mcur1	0.080119	0.019381	1
Epas1	0.080081	0.190357	1
Per1	0.07999	0.149938	1
Kbtbd3	0.079886	0.617829	1
Slc25a22	0.079833	0.477211	1
Rrn3	0.0798	0.192234	1
1700017B05I	0.079774	0.350242	1
Rab28	0.079759	0.208037	1
Nbas	0.079627	0.299682	1
Hlcs	0.079533	0.060805	1
Brms1	0.079405	0.268121	1
Dtd1	0.079376	0.115919	1
Hipk3	0.079367	0.281353	1
Dhrs4	0.079362	0.19191	1
Dad1	0.079334	0.203846	1
Stat5b	0.079325	0.642643	1
Rgcc	0.079144	0.262125	1
AC113595.1	0.079108	0.148108	1
Slc46a3	0.079056	0.371041	1
Timm13	0.078932	0.542485	1
Tmed3	0.078927	0.046133	1
Herc4	0.07885	0.723434	1
Nin	0.078814	0.178516	1
Vcpkmt	0.078802	0.143431	1

Trim11	0.078738	0.181147	1
Micos13	0.078709	0.486747	1
Dubr	0.078637	0.383438	1
A430088P11I	0.07862	0.260809	1
Setdb1	0.078555	0.257019	1
Decr1	0.078536	0.303828	1
Drosha	0.078517	0.134731	1
Pcid2	0.0785	0.53497	1
Slc39a4	0.078471	0.117733	1
Commd7	0.078308	0.30882	1
Khdc4	0.078219	0.372217	1
Eif4enif1	0.078179	0.291274	1
Exosc9	0.078153	0.527597	1
Ppfibp1	0.078134	0.104757	1
Polr2m	0.078025	0.046265	1
Slc7a6os	0.078012	0.406612	1
Mecr	0.07796	0.245852	1
Eef2	0.07794	0.202282	1
Impact	0.077939	0.216076	1
Nfe2l1	0.07788	0.287609	1
Prrc2b	0.077844	0.518744	1
Ercc6l2	0.077831	0.704727	1
Cdkn1b	0.077828	0.676358	1
Hsd17b10	0.077781	0.374325	1
Fbxw11	0.077773	0.284535	1
Rps19bp1	0.077729	0.359816	1
Gna13	0.077689	0.333728	1
Pex3	0.077673	0.792455	1
Taf1c	0.077632	0.317257	1
Mrpl30	0.077628	0.398748	1
Uqcrfs1	0.077547	0.38912	1
Slc25a39	0.077543	0.274478	1
Wars	0.077497	0.144086	1
Srp9	0.07749	0.154787	1
Lamc1	0.077488	0.189913	1
Aggf1	0.077464	0.611272	1
Vwa5a	0.077455	0.150781	1
Mettl1	0.077337	0.056586	1
Wapl	0.07731	0.350505	1
2310001H17	0.077232	0.411884	1
Nfatc1	0.077226	0.419441	1
Map3k14	0.077202	0.298141	1
Psmd4	0.077173	0.530149	1
Odr4	0.077125	0.313624	1
Gm6034	0.077093	0.089467	1
Tcta	0.077077	0.091124	1
Klhl12	0.077068	0.246744	1
Mpp7	0.077009	0.308702	1

Dhodh	0.076929	0.282204	1
Eef1akmt4	0.076916	0.129309	1
Chchd5	0.076902	0.16003	1
Ppp1r12c	0.076781	0.464026	1
Deaf1	0.076704	0.306494	1
Apool	0.076521	0.550803	1
Insyn2b	0.076513	0.906967	1
Ttc5	0.0765	0.411025	1
Yif1b	0.076496	0.11559	1
CAAA011473	0.076463	0.451875	1
Gm44148	0.076418	0.378229	1
Mrpl13	0.076288	0.178486	1
Pdss2	0.076269	0.494538	1
Mfsd8	0.076247	0.108828	1
Ascc3	0.076233	0.554839	1
Zfp995	0.076154	0.069595	1
9130401M01	0.07606	0.133457	1
Cd3eap	0.075899	0.24953	1
Wdr75	0.075897	0.147927	1
Kctd17	0.07589	0.318023	1
Sirt6	0.075877	0.247952	1
Ctcf	0.075866	0.319693	1
Mta3	0.075805	0.092527	1
Ski	0.075804	0.368126	1
Atf6b	0.075771	0.72664	1
Cbr4	0.075683	0.161521	1
Vezt	0.075532	0.147907	1
Elp5	0.075409	0.407171	1
2610507B11	0.075327	0.221556	1
Slc35a1	0.075189	0.304135	1
Mapkap1	0.075162	0.318325	1
Aldh7a1	0.07509	0.289363	1
Zbtb4	0.075007	0.365498	1
Yae1d1	0.074987	0.26839	1
Ammecr1	0.074861	0.318192	1
B3galt6	0.074782	0.239503	1
Ints12	0.074778	0.059706	1
Zfp318	0.074678	0.078562	1
Ctso	0.074668	0.218849	1
Eif2b3	0.074568	0.211332	1
Osgin1	0.074467	0.013373	1
1110065P20	0.074452	0.13125	1
Aimp2	0.074446	0.513731	1
Psmg2	0.074444	0.523764	1
Slc25a3	0.074396	0.527685	1
Nsun3	0.074377	0.3109	1
Nlk	0.07436	0.469889	1
Pgghg	0.074312	0.144775	1

Atp6ap2	0.074228	0.735367	1
Blcap	0.074225	0.247196	1
Adss	0.074155	0.379142	1
Rab9	0.074143	0.19924	1
Mrps10	0.074085	0.216867	1
Cnih4	0.074055	0.305801	1
Acat1	0.074032	0.303108	1
Krcc1	0.073959	0.354015	1
Rbm25	0.073933	0.692446	1
Cinp	0.073933	0.234933	1
Psd4	0.073901	0.428147	1
Casp6	0.073848	0.288111	1
Malsu1	0.073808	0.206995	1
2610002M06	0.073765	0.256265	1
Pmepa1	0.073751	0.30669	1
Ttll12	0.073707	0.452621	1
Ofd1	0.073606	0.183851	1
Pikfyve	0.073577	0.299318	1
Srp14	0.07354	0.425618	1
Txndc12	0.073539	0.250702	1
Pnpo	0.073453	0.072766	1
Slc43a2	0.073433	0.080054	1
Anks3	0.073424	0.092136	1
Ascc1	0.073264	0.930886	1
Tstd3	0.073102	0.125575	1
Fbxw2	0.073059	0.788544	1
2900097C17	0.073014	0.255024	1
Dedd2	0.073012	0.127661	1
Gm17745	0.073008	0.277029	1
Exosc1	0.072924	0.418306	1
Tomm40	0.072904	0.745822	1
Krtcap2	0.072833	0.361836	1
Itfg1	0.072629	0.112657	1
Rraga	0.072607	0.341792	1
Rbis	0.072507	0.297736	1
Riox1	0.072471	0.158522	1
Notch2	0.072383	0.600642	1
BC005537	0.072347	0.235538	1
Dpm2	0.072273	0.298452	1
Ap3d1	0.072231	0.28811	1
Acp5	0.07222	0.615499	1
Xylt2	0.072215	0.530565	1
Prrc2c	0.072023	0.831571	1
Vapb	0.072022	0.352664	1
Polr2e	0.071876	0.733652	1
Ppme1	0.071872	0.135419	1
Akr1b10	0.071844	0.265323	1
Slc2a8	0.071782	0.024759	1

Nr2c2	0.071775	0.577831	1
Arhgef3	0.071737	0.493789	1
Ubxn1	0.071684	0.478751	1
Mthfsd	0.071676	0.196904	1
AU022252	0.071487	0.090384	1
2210016L21I	0.071481	0.553331	1
Pcf11	0.07145	0.157739	1
Gin1	0.071378	0.294414	1
Cstf1	0.071352	0.132724	1
Ilf3	0.071341	0.641897	1
Ndufs4	0.071331	0.619383	1
Tfam	0.071331	0.215781	1
Psmd9	0.071188	0.761527	1
Arid4b	0.071099	0.929274	1
Cplane1	0.070833	0.281269	1
Cops7b	0.070823	0.628309	1
Pwp1	0.070823	0.358627	1
Ndufaf3	0.070708	0.218015	1
Mrps11	0.070409	0.336049	1
Mettl2	0.0704	0.387568	1
Rnf185	0.070277	0.184387	1
Hsdl1	0.070257	0.113147	1
Khdrbs1	0.070197	0.502323	1
Eif3h	0.070174	0.110692	1
Arf4	0.07012	0.136345	1
Usf1	0.070104	0.33623	1
2310009B15I	0.070092	0.600019	1
Tmem94	0.070055	0.104924	1
Use1	0.070006	0.488247	1
Dnajb6	0.069965	0.540345	1
Zyg11b	0.069899	0.126869	1
Tprgl	0.069881	0.419331	1
Snrpb2	0.06984	0.697936	1
Rsu1	0.069727	0.307201	1
Surf2	0.069642	0.429835	1
Gm43672	0.069579	0.164077	1
Mboat1	0.069521	0.064941	1
Atp9b	0.069512	0.42395	1
Aagab	0.069469	0.508017	1
Dennd4c	0.069435	0.261485	1
mt-Nd2	0.069375	0.480413	1
Pprc1	0.069369	0.371181	1
Pes1	0.069313	0.504329	1
Idh3g	0.069255	0.330677	1
Supt20	0.069218	0.38227	1
Gm44174	0.069217	0.588727	1
Cnbp	0.069137	0.06776	1
6530402F18I	0.069108	0.057368	1

Acads	0.069082	0.189599	1
Eif2ak2	0.068997	0.639385	1
Cep44	0.068947	0.46861	1
Tasp1	0.068874	0.363501	1
Cfap97	0.06884	0.165632	1
Wbp1	0.06879	0.129791	1
Fam50a	0.068788	0.758125	1
Zfp512	0.068721	0.630884	1
Txnip	0.068711	0.1207	1
Spag9	0.068707	0.515196	1
Ar	0.068674	0.924444	1
Bzw2	0.068672	0.171868	1
Psmc5	0.06866	0.20308	1
Zfp560	0.068587	0.142703	1
Sumo1	0.068554	0.338041	1
Sco2	0.068469	0.441546	1
Ift27	0.068269	0.389795	1
Lias	0.068232	0.168892	1
Phlpp1	0.068194	0.520953	1
Rab24	0.068073	0.353434	1
Pex13	0.068002	0.35338	1
Atp6v0e	0.067925	0.565799	1
Tsnax	0.067907	0.541216	1
Alad	0.067882	0.691822	1
Cyth4	0.067761	0.160875	1
Fbxw17	0.067706	0.387732	1
Rras	0.067585	0.142692	1
Zfp280b	0.067575	0.329413	1
Vps28	0.067495	0.584107	1
Tmem120a	0.067434	0.179268	1
Ei24	0.067246	0.252666	1
Trappc3	0.06717	0.965078	1
Slc35b3	0.067112	0.411727	1
Unc119b	0.067062	0.137427	1
Trp53	0.06693	0.278806	1
Sugp1	0.066925	0.606688	1
Ppan	0.066862	0.45056	1
Pcbp2	0.066838	0.487767	1
Galnt12	0.06682	0.086498	1
Zfp445	0.066818	0.971719	1
Pam16	0.066807	0.99154	1
Nudt18	0.066685	0.487121	1
Nvl	0.066671	0.843365	1
Nudcd2	0.066659	0.067431	1
Rbm39	0.066632	0.165896	1
Zfp217	0.066627	0.352676	1
Dmap1	0.066594	0.396411	1
Zfp942	0.066521	0.484953	1

Grap	0.066501	0.557575	1
Atp6v1h	0.066483	0.382216	1
Sik2	0.066478	0.341657	1
Nabp2	0.066471	0.36666	1
Ndufa7	0.066471	0.455202	1
Pdcd11	0.066444	0.422488	1
Fam122a	0.066367	0.339203	1
Zbtb17	0.066349	0.33177	1
Vars	0.066302	0.281391	1
Slc25a32	0.066263	0.440717	1
Rex1bd	0.066213	0.144094	1
Ak6	0.066153	0.629954	1
Slc35d2	0.066129	0.184618	1
Slfn9	0.066041	0.507976	1
Tmem128	0.066027	0.099365	1
Utp14a	0.065998	0.211383	1
Rnf123	0.065954	0.158468	1
Dctn3	0.065924	0.357229	1
Bet1	0.065766	0.445177	1
Cops8	0.065701	0.73191	1
Ttc19	0.065672	0.129738	1
Thumpd3	0.065599	0.758717	1
Ccpg1	0.065589	0.286393	1
Huwe1	0.065573	0.835449	1
BC003965	0.065558	0.65047	1
Ftsj3	0.06545	0.552041	1
Elovl5	0.065378	0.920941	1
Tbc1d4	0.065102	0.65467	1
Chic2	0.065087	0.420767	1
Znhit3	0.065031	0.790636	1
Nipsnap1	0.064883	0.390961	1
Mpp6	0.064729	0.541888	1
Tent4b	0.064625	0.54883	1
Cd44	0.064606	0.692285	1
Snhg6	0.064481	0.598423	1
Ncf4	0.064423	0.060004	1
Taf7	0.06441	0.062278	1
Gpx4	0.064357	0.123217	1
Sos2	0.064317	0.973293	1
Rnf44	0.064265	0.666831	1
Nktr	0.064224	0.298554	1
Dcun1d3	0.064196	0.102786	1
Amdhd2	0.064113	0.202926	1
Rexo4	0.064005	0.76934	1
Ctu2	0.063968	0.568412	1
Vti1a	0.063946	0.214905	1
Ogfr	0.06394	0.208082	1
Spop	0.063825	0.678508	1

Alkbh6	0.063746	0.444135	1
Rpl41	0.063726	0.118835	1
Traf6	0.063699	0.311726	1
Fnbp4	0.063676	0.560964	1
Chmp1a	0.063605	0.944318	1
4933434E20I	0.063574	0.465514	1
Zfp276	0.063448	0.108941	1
Nop2	0.06341	0.29908	1
Tex14	0.063382	0.422766	1
Stam	0.063364	0.427562	1
Lyrm1	0.063344	0.455558	1
2010016I18F	0.063333	0.776569	1
Tg	0.063275	0.547802	1
Eaf1	0.06326	0.450058	1
Sod2	0.063174	0.503799	1
Ppil2	0.063146	0.199823	1
Zfyve1	0.06314	0.108821	1
Utp23	0.063117	0.537811	1
Iah1	0.063074	0.734961	1
Tmed4	0.063028	0.317997	1
Polm	0.063011	0.316554	1
Jtb	0.06298	0.471797	1
Klhl24	0.062979	0.045452	1
Cd200	0.062972	0.264989	1
Dph7	0.062958	0.414459	1
Plagl2	0.062909	0.283679	1
Stx18	0.062828	0.326469	1
Tbcel	0.062732	0.176484	1
Golph3	0.062713	0.490352	1
Zfr	0.06265	0.34091	1
Slc33a1	0.062525	0.371515	1
Arhgap9	0.062488	0.252262	1
Ankrd28	0.062484	0.078441	1
Myd88	0.06244	0.317795	1
Nmt1	0.062387	0.332895	1
Metap1	0.062281	0.550337	1
Cd37	0.062254	0.008054	1
Snai3	0.06224	0.408223	1
Ifi209	0.062237	0.294164	1
Optn	0.062224	0.074249	1
Gprin3	0.062199	0.960009	1
Terf2ip	0.062112	0.574273	1
Git2	0.06208	0.977126	1
Gpatch1	0.06208	0.510149	1
Entpd5	0.061989	0.724407	1
Cbr1	0.061871	0.31239	1
Zfp429	0.06171	0.268321	1
Flywch1	0.06164	0.488362	1

Rnmt	0.061616	0.553418	1
Manba	0.061577	0.78174	1
Shld2	0.061556	0.298233	1
Cript	0.061532	0.413733	1
Thoc1	0.061523	0.474625	1
Pnpla8	0.061512	0.199605	1
Maz	0.061499	0.542295	1
Utp15	0.061492	0.220228	1
Tmem106a	0.061492	0.177484	1
Tnfsf9	0.06145	0.871749	1
Tnrc6c	0.061401	0.279006	1
Chtf8	0.061332	0.390349	1
Ssbp3	0.0613	0.511604	1
Tep1	0.061202	0.201175	1
Pick1	0.061171	0.513062	1
Phf1	0.061147	0.827267	1
Ctsa	0.061107	0.406873	1
Fech	0.061104	0.119486	1
Abcb1a	0.061101	0.261692	1
Pinx1	0.060996	0.469311	1
Mdm4	0.060986	0.87268	1
Dnajb4	0.060982	0.673244	1
Nepro	0.060975	0.243567	1
Wdr74	0.060953	0.269659	1
Srrm2	0.060877	0.452097	1
Pdrg1	0.060877	0.276386	1
Tprn	0.06087	0.224787	1
Tnrc6b	0.060863	0.501845	1
Galk1	0.060813	0.463059	1
Sec63	0.060782	0.284586	1
Tceanc2	0.060752	0.216467	1
Mvp	0.060743	0.262145	1
Cpped1	0.060628	0.565212	1
Art2b	0.060527	0.723539	1
Zdhhc15	0.060524	0.829548	1
Gbe1	0.060418	0.954335	1
Osbpl7	0.060397	0.894586	1
Zfpm1	0.060365	0.361248	1
Kras	0.06035	0.881946	1
Prpf31	0.06023	0.910341	1
Ccdc115	0.060101	0.412053	1
Psmd12	0.060081	0.244828	1
Dnajc1	0.060013	0.573926	1
Specc1l	0.059988	0.406212	1
Dnajc24	0.059956	0.421298	1
Mau2	0.05991	0.428212	1
Plk3	0.05989	0.127326	1
Lyrm2	0.059853	0.467953	1

Arhgap31	0.059835	0.963906	1
Timm9	0.059816	0.066254	1
Snapc2	0.059774	0.275598	1
Pop7	0.059659	0.232874	1
Dusp4	0.05963	0.8327	1
Tmem248	0.05962	0.0734	1
Faap20	0.059289	0.594028	1
Hp1bp3	0.059148	0.754291	1
Dhx38	0.059109	0.357505	1
Polr1d	0.058935	0.300605	1
2810001G20	0.058876	0.433351	1
Itch	0.058793	0.819247	1
Strn4	0.058708	0.290962	1
Gskip	0.058691	0.432273	1
Helz	0.058631	0.545221	1
Tmem87b	0.05861	0.465364	1
Dusp12	0.058581	0.448514	1
Eif3b	0.058569	0.356438	1
Rsph3b	0.058561	0.161372	1
Slc37a4	0.058534	0.509278	1
Kat6b	0.058406	0.360817	1
Slc38a6	0.058401	0.301401	1
Eipr1	0.058369	0.621857	1
3830406C13	0.058289	0.512578	1
Prorsd1	0.058278	0.412257	1
Bcap29	0.05825	0.188652	1
Ddx19b	0.058238	0.385552	1
Mctp2	0.058233	0.824389	1
Fam172a	0.058117	0.523889	1
Pgrmc2	0.058088	0.648413	1
Mrpl11	0.05808	0.348415	1
Dcaf4	0.058072	0.467334	1
Ube4a	0.058037	0.650565	1
Bmt2	0.05799	0.747477	1
Akap8	0.057915	0.535048	1
Gpalpp1	0.057911	0.611161	1
Nap1l4	0.057886	0.683768	1
Tmem209	0.057884	0.253368	1
Gcc1	0.057879	0.468826	1
Rap2c	0.057854	0.27246	1
Fkbp3	0.057758	0.599676	1
Stap1	0.057653	0.336997	1
Sufu	0.057642	0.643435	1
Spryd7	0.057585	0.541929	1
Slc2a9	0.057582	0.415067	1
Gnaq	0.057566	0.235211	1
Fkbp15	0.05755	0.163908	1
Fbxl8	0.057548	0.448184	1

Mtf2	0.057537	0.774373	1
Dnajc17	0.057468	0.240608	1
Dennd1b	0.05745	0.766687	1
Gsk3a	0.057388	0.246414	1
Cdc42se2	0.057277	0.511654	1
Pex14	0.057246	0.768882	1
Ift52	0.057103	0.736299	1
Tes	0.057071	0.390859	1
Ccnt1	0.057058	0.567688	1
Spg21	0.056986	0.465737	1
Fundc1	0.056915	0.974758	1
9330175E14I	0.056794	0.453514	1
Blvra	0.056695	0.841857	1
Zfp617	0.056623	0.622318	1
Dguok	0.056601	0.184239	1
Atg7	0.056487	0.534898	1
Poli	0.056391	0.602672	1
Hivep2	0.056389	0.96203	1
Mff	0.056377	0.937709	1
Heg1	0.056371	0.635502	1
Acaa2	0.056352	0.350106	1
Ppp2r2d	0.056343	0.337859	1
Ccdc25	0.05632	0.284887	1
Coa3	0.056303	0.577787	1
Rbks	0.056225	0.204852	1
Reep3	0.056214	0.257435	1
Parp6	0.056168	0.195893	1
Gsdmd	0.056141	0.802561	1
Rit1	0.056108	0.617631	1
Tmbim1	0.056011	0.4978	1
Ccrl2	0.056001	0.997869	1
Rnf34	0.055975	0.326755	1
Fgf13	0.055964	0.880646	1
Ptges3	0.055941	0.301401	1
Stk3	0.055886	0.344576	1
Rps29	0.055783	0.27387	1
Pigu	0.055625	0.178181	1
Rsf1	0.055537	0.268687	1
Opa3	0.055528	0.113074	1
Casp2	0.055511	0.713208	1
Disp1	0.055479	0.564265	1
Jmjd6	0.055473	0.787819	1
Riok3	0.055385	0.813627	1
Zkscan1	0.055376	0.950822	1
Ube2j2	0.055367	0.604221	1
Sra1	0.055339	0.480027	1
Exoc3	0.055327	0.405294	1
Zfpl1	0.055311	0.102783	1

Crtc3	0.055166	0.361455	1
Rhoh	0.055162	0.369991	1
March6	0.055076	0.542267	1
Dcun1d1	0.055013	0.932571	1
Atp5c1	0.055005	0.59907	1
Lyar	0.054913	0.997964	1
Srpr	0.054861	0.225116	1
Ccdc125	0.054822	0.859296	1
Ddx42	0.05478	0.535377	1
Ambra1	0.054754	0.830764	1
Mthfd2	0.054685	0.949036	1
Ddx5	0.054625	0.280712	1
Edc3	0.054591	0.356533	1
Adar	0.05458	0.66571	1
Thoc3	0.054574	0.50712	1
Tbc1d22a	0.054543	0.291761	1
Cep135	0.054469	0.754343	1
Gcsh	0.054436	0.257683	1
Fas	0.054417	0.094397	1
Cic	0.05422	0.92548	1
Ube2z	0.054189	0.409714	1
C330018D20	0.054092	0.086383	1
1810013L24I	0.054074	0.615647	1
Zfp622	0.053985	0.705372	1
Usp8	0.05395	0.423599	1
Dzip3	0.053854	0.367614	1
1110038B12I	0.053815	0.299141	1
Trgv2	0.053814	0.196542	1
Sirt5	0.053779	0.524818	1
Cdk5rap3	0.053753	0.766518	1
Prmt6	0.053693	0.048204	1
Mrps31	0.053628	0.52255	1
Gm14326	0.053617	0.121868	1
Bnip1	0.05356	0.304854	1
Chmp6	0.053548	0.625458	1
Kif1bp	0.053495	0.505375	1
Adck1	0.053487	0.279673	1
Tmed7	0.053404	0.406783	1
Kdm6a	0.0534	0.986961	1
Atp6v1d	0.0533	0.553509	1
Bag3	0.053252	0.662686	1
4632427E13I	0.05323	0.371537	1
Tbl3	0.053204	0.600131	1
Cdyl2	0.053177	0.238732	1
Scap	0.053164	0.773936	1
Crk	0.053021	0.64358	1
Dhx57	0.052958	0.873815	1
Mlkl	0.052917	0.388026	1

Trim65	0.052911	0.688455	1
Mrps7	0.052897	0.780362	1
Marcksl1	0.052803	0.988143	1
Isoc2b	0.052711	0.146392	1
Vps26b	0.052663	0.68099	1
Kdm4c	0.052643	0.663149	1
Stk40	0.052623	0.291592	1
Zfp213	0.052567	0.604554	1
Rab2b	0.052527	0.590062	1
Ints3	0.052508	0.839231	1
Rnf41	0.052444	0.963394	1
Fam136a	0.052364	0.467963	1
Eya3	0.052292	0.369233	1
Eif2ak3	0.052247	0.306561	1
4930523C07I	0.052237	0.387268	1
Rbm33	0.052229	0.479303	1
Cmc4	0.05216	0.576478	1
Smdt1	0.052004	0.440818	1
G6pc3	0.051986	0.387147	1
Vps9d1	0.051954	0.270558	1
Prpf4	0.051927	0.879161	1
Etfrf1	0.051867	0.461601	1
Ptges2	0.051864	0.34382	1
Ggact	0.051739	0.380043	1
Sar1a	0.051711	0.884689	1
Gtpbp4	0.051575	0.193234	1
Taf13	0.051499	0.383216	1
Coa4	0.051467	0.583824	1
Dnajb12	0.051302	0.602814	1
Mfn1	0.051297	0.744243	1
Alg5	0.051292	0.03141	1
Slc50a1	0.051247	0.50073	1
Edem2	0.051235	0.352756	1
Patl1	0.051186	0.847621	1
Esm1	0.05118	0.7823	1
Sec11a	0.051173	0.490639	1
Cox15	0.051118	0.678311	1
Polr3e	0.0511	0.279643	1
Slc7a1	0.051068	0.715962	1
Tmem123	0.051022	0.578532	1
Btf3	0.050988	0.229632	1
Actr8	0.050988	0.741711	1
Mex3c	0.050903	0.574519	1
Anapc2	0.050791	0.362092	1
Nt5dc1	0.050724	0.396403	1
Chchd7	0.050687	0.506923	1
Cpsf3	0.050545	0.47625	1
Zfp507	0.050544	0.287285	1

Top3a	0.050467	0.398811	1
Nudt13	0.050463	0.173547	1
Hax1	0.050437	0.090956	1
Bcl11b	0.050361	0.092512	1
Gm5617	0.050338	0.625269	1
Med4	0.050245	0.929779	1
2610008E11I	0.050211	0.267634	1
Ecpas	0.05019	0.911198	1
Dhx40	0.050183	0.327871	1
Zzz3	0.050175	0.283377	1
Ube3a	0.050145	0.593402	1
Grsf1	0.050129	0.829272	1
Rbm19	0.050121	0.353588	1
Usp9x	0.050073	0.405893	1
Slc39a7	0.050055	0.825439	1
Acbd3	0.050032	0.317482	1
Mfsd1	0.049988	0.854614	1
1600020E01I	0.049961	0.598092	1
Tmem126b	0.049874	0.081346	1
Plbd2	0.049865	0.008997	1
Chd2	0.049783	0.082447	1
Pacsin1	0.049768	0.351639	1
Mknk2	0.049669	0.823745	1
Nsmce1	0.049645	0.466447	1
Exosc2	0.049639	0.517923	1
Tnfsf13b	0.049625	0.069779	1
Zdhhc6	0.049607	0.474812	1
Il18bp	0.049602	0.003297	1
Csnk2a2	0.049594	0.169819	1
Nudt2	0.049584	0.205256	1
Zkscan14	0.049463	0.968908	1
Abcd4	0.049461	0.400455	1
Cited2	0.049413	0.792436	1
Tmem106c	0.049409	0.455756	1
Stard7	0.049367	0.7778	1
Eif4ebp2	0.049355	0.731643	1
4931406P16I	0.049275	0.728473	1
Adpgk	0.049241	0.264163	1
Rab3gap1	0.049211	0.911283	1
Prcc	0.04917	0.531899	1
U2af1l4	0.049156	0.806444	1
Kansl1l	0.049093	0.379177	1
Hacl1	0.049091	0.201385	1
Snapc3	0.049077	0.896065	1
Uggt1	0.049075	0.951153	1
Mrpl55	0.049035	0.370949	1
Polb	0.049014	0.498586	1
Secisbp2l	0.048989	0.435535	1

Ago1	0.04898	0.376826	1
Sptlc2	0.048948	0.910332	1
Mkrn1	0.048876	0.556742	1
Cdk10	0.048863	0.519591	1
Tmem230	0.048826	0.516847	1
Rgs3	0.048757	0.459037	1
Znhit1	0.048757	0.739834	1
Pgs1	0.048584	0.38291	1
Arglu1	0.048583	0.929871	1
Usb1	0.048505	0.762858	1
Dhcr7	0.048462	0.713009	1
Gm9725	0.048433	0.493969	1
Nop10	0.048422	0.576175	1
Bach1	0.048402	0.559808	1
Fam168a	0.048331	0.841789	1
Zfp984	0.048291	0.40252	1
Mfn2	0.048284	0.304598	1
Exoc7	0.048272	0.728611	1
Yipf1	0.048227	0.375453	1
Ergic1	0.048176	0.03373	1
Kat2b	0.048175	0.449453	1
Lsg1	0.048173	0.882002	1
Fra10ac1	0.04817	0.044117	1
Acadl	0.04816	0.691217	1
Nrde2	0.048132	0.313619	1
Cluap1	0.047954	0.904271	1
Mindy3	0.047942	0.414793	1
Tsr1	0.04794	0.601899	1
Letm2	0.047928	0.478293	1
Aatf	0.047852	0.383536	1
Stk16	0.047838	0.367856	1
Eml2	0.047825	0.219143	1
Lpgat1	0.047817	0.795544	1
Zfp281	0.047805	0.551722	1
Pex2	0.047793	0.342558	1
Rufy2	0.04776	0.436231	1
Kmt2b	0.047724	0.416746	1
Gclc	0.0477	0.185524	1
Phf3	0.047634	0.762768	1
Mbd5	0.047626	0.463883	1
Ndel1	0.04761	0.212707	1
Lrrc40	0.047598	0.179716	1
5730455P16	0.047587	0.276895	1
Bag1	0.047584	0.444333	1
Arl4a	0.047568	0.643329	1
Mrpl21	0.047555	0.542432	1
Wdr3	0.047304	0.837627	1
Ppargc1b	0.047291	0.18796	1

Tmem104	0.047268	0.774593	1
Gm26740	0.047204	0.815301	1
Cenpb	0.047106	0.617173	1
Zfp950	0.047012	0.352474	1
Washc4	0.04701	0.555644	1
Thumpd2	0.046973	0.823901	1
Tnrc6a	0.046881	0.714263	1
Trit1	0.046835	0.371038	1
Ormdl2	0.04682	0.610134	1
Vipas39	0.04671	0.601092	1
Tcof1	0.046695	0.905524	1
Tmem147	0.04668	0.460118	1
Ndufa9	0.046673	0.521459	1
Lpcat1	0.04665	0.521086	1
Mark2	0.04654	0.797047	1
Afg1l	0.046499	0.449662	1
Orc5	0.046417	0.613969	1
Armt1	0.046283	0.535154	1
Eftud2	0.046218	0.622929	1
Plekhf1	0.046161	0.755943	1
Trim39	0.046153	0.286339	1
Arfgap3	0.046131	0.421552	1
Polr2f	0.04611	0.592526	1
AI987944	0.046096	0.232241	1
Tmem165	0.046053	0.734524	1
Irak1	0.046045	0.480344	1
Brd2	0.046037	0.671687	1
Gtpbp1	0.045956	0.729552	1
Mpg	0.045891	0.51027	1
Ccnl2	0.045863	0.906189	1
Nsa2	0.045853	0.683868	1
Zfand6	0.045798	0.793694	1
Ahsa1	0.045748	0.953537	1
Ube2h	0.045739	0.885621	1
Lrrc14	0.045713	0.33169	1
Mrpl19	0.045711	0.532307	1
Tmem138	0.04571	0.526548	1
Prpf39	0.045613	0.79634	1
Aldh3a2	0.045607	0.063459	1
Wdr12	0.045515	0.829939	1
Hint2	0.04548	0.004159	1
Pmaip1	0.045477	0.745152	1
Ralgapa1	0.045435	0.898158	1
Psip1	0.045428	0.966808	1
Kansl1	0.045423	0.701706	1
Ndufb10	0.045359	0.51127	1
Snupn	0.045294	0.64805	1
Rps26	0.045223	0.815844	1

Tomm5	0.045218	0.694031	1
Zfp609	0.04521	0.693968	1
Pnpt1	0.045175	0.773585	1
Nifk	0.04516	0.779455	1
Katnbl1	0.045088	0.750135	1
Wdr77	0.045036	0.299296	1
Ccdc9	0.044994	0.643015	1
Smim3	0.044966	0.166039	1
Trappc2l	0.044951	0.771579	1
Pop1	0.044929	0.173643	1
Ero1l	0.044884	0.035932	1
Ddx21	0.044822	0.66202	1
Resf1	0.044783	0.654619	1
Kdm3a	0.044782	0.93055	1
Fbxo8	0.044674	0.28473	1
Rnf121	0.044632	0.43668	1
Ttc32	0.044575	0.287431	1
Ap2a1	0.044574	0.74954	1
Golga7	0.044558	0.741644	1
Apoo	0.044518	0.393329	1
Ubald2	0.044499	0.460977	1
Gm36975	0.044464	0.391875	1
Mbtd1	0.044451	0.580108	1
Dusp19	0.04445	0.833584	1
Ube2e1	0.044415	0.512616	1
D430042O09	0.044414	0.690927	1
Ice2	0.044372	0.3912	1
Dop1a	0.04435	0.904116	1
Ildr1	0.044349	0.994057	1
Atxn7l3b	0.044328	0.688559	1
Tarbp2	0.04421	0.160025	1
Sh3glb1	0.044019	0.671903	1
Cox10	0.043924	0.890309	1
Zfp738	0.043859	0.48256	1
Trim35	0.043804	0.553795	1
Snhg16	0.043799	0.472226	1
Yaf2	0.043641	0.786121	1
Cdadc1	0.043603	0.721986	1
Tbc1d17	0.043565	0.45489	1
Noc3l	0.043563	0.794853	1
Metap1d	0.043549	0.394542	1
Mapk8ip3	0.043541	0.744023	1
Ngrn	0.043533	0.702214	1
Napa	0.043511	0.36504	1
Polr2d	0.043467	0.597851	1
Bora	0.043446	0.387926	1
Siva1	0.043417	0.927101	1
Phlda1	0.043396	0.420302	1

Lats1	0.043387	0.484092	1
Gm39469	0.04338	0.94038	1
Trappc11	0.043359	0.947349	1
Rheb	0.043331	0.733703	1
Cd101	0.043314	0.417527	1
Pcbd2	0.043276	0.879192	1
Lxn	0.043189	0.701066	1
Ust	0.043183	0.849581	1
Gm16565	0.04316	0.343276	1
Trappc8	0.043128	0.772308	1
Hrh2	0.043077	0.469431	1
Smyd2	0.043057	0.642385	1
Gabarap	0.043029	0.677725	1
A530088E08I	0.04296	0.295904	1
Crebzf	0.042943	0.691182	1
Avl9	0.042936	0.555413	1
Setd4	0.042909	0.591516	1
Pyroxd1	0.042878	0.57257	1
Znrf2	0.042872	0.527224	1
Chmp3	0.04284	0.807039	1
Elp2	0.042771	0.732396	1
Dhx36	0.042728	0.915566	1
Zmiz1	0.042693	0.742338	1
Atp5d	0.042611	0.61128	1
Ptpn12	0.042579	0.946212	1
Acsf2	0.042569	0.241032	1
Cdip1	0.042498	0.329333	1
Relch	0.042477	0.757264	1
Gcfc2	0.042458	0.58287	1
Mtf1	0.042443	0.990045	1
Eif2b2	0.042421	0.36812	1
Mettl26	0.042379	0.712468	1
Ankrd46	0.04234	0.280274	1
Mrps18c	0.042318	0.378703	1
Pnisr	0.042205	0.484964	1
Lamb3	0.042199	0.556917	1
Ivd	0.042087	0.644126	1
LTO1	0.042055	0.509142	1
Wasl	0.041979	0.770856	1
Arel1	0.041938	0.426496	1
Isg20l2	0.041876	0.615079	1
Zfp831	0.041864	0.458694	1
Pip4p2	0.041862	0.65364	1
Ppp3cb	0.041853	0.908941	1
Timm10	0.041683	0.933112	1
Enoph1	0.041682	0.468222	1
Zc3hc1	0.041664	0.12269	1
Chd4	0.041656	0.950351	1

Eri3	0.041621	0.761472	1
Ctla2b	0.041515	0.782935	1
Armc10	0.041441	0.662623	1
Tomm40l	0.041332	0.755039	1
Nudt7	0.041282	0.549415	1
Gmeb2	0.041261	0.844134	1
Rtcb	0.041207	0.158919	1
Zfp451	0.0412	0.057086	1
Zscan26	0.04117	0.726218	1
Hsbp1	0.041167	0.533382	1
Slc16a3	0.041126	0.862387	1
Slc25a45	0.041035	0.879856	1
Babam2	0.041009	0.673496	1
Vcpip1	0.040924	0.979589	1
Foxk1	0.040853	0.869304	1
Mcm9	0.040844	0.912329	1
Ap3m2	0.04083	0.526735	1
Cul2	0.040807	0.982345	1
Nras	0.040707	0.935654	1
Trim56	0.040702	0.467074	1
Mthfs	0.040694	0.972561	1
Zfp472	0.040666	0.42844	1
Zfp932	0.040647	0.466919	1
AW554918	0.0406	0.817466	1
Znhit6	0.040575	0.57842	1
Bccip	0.040573	0.656438	1
Rab37	0.040567	0.494511	1
E2f3	0.040553	0.992345	1
Tubgcp5	0.040549	0.572787	1
Nmnat3	0.040539	0.987772	1
Napg	0.040495	0.619529	1
Lin52	0.04049	0.769495	1
1810058I24F	0.040336	0.493074	1
Arfip2	0.040291	0.9234	1
Elac2	0.040288	0.492617	1
Nfyc	0.04022	0.665596	1
Clp1	0.04021	0.622034	1
Sec62	0.040197	0.991296	1
Map3k5	0.040181	0.67162	1
Shprh	0.040101	0.92527	1
Xlr4a	0.040015	0.293012	1
Hps1	0.04	0.752593	1
Dus1l	0.039987	0.918121	1
Cd99l2	0.039957	0.311226	1
Rbm45	0.039913	0.822475	1
Ebag9	0.039905	0.412838	1
Jund	0.039877	0.36692	1
Itpripl2	0.039876	0.326596	1

Gm16124	0.039862	0.287622	1
Klhl26	0.039848	0.406591	1
Edem1	0.039846	0.611589	1
Manbal	0.039814	0.723323	1
Nmt2	0.039759	0.817623	1
Msi2	0.039747	0.891626	1
Tcirg1	0.039685	0.937081	1
Apobr	0.039657	0.073527	1
Gatd3a	0.039625	0.21149	1
Crlf3	0.039571	0.746181	1
Slc29a1	0.039541	0.533564	1
Mt3	0.039537	0.180354	1
Timm23	0.039523	0.543876	1
Rab22a	0.039513	0.918699	1
Lcorl	0.039509	0.888865	1
Plekhb2	0.039479	0.661641	1
Caap1	0.039472	0.250288	1
Ice1	0.039468	0.40491	1
Dusp11	0.039466	0.724677	1
Akap11	0.039374	0.63937	1
Pigk	0.039373	0.447802	1
Lactb	0.039344	0.274258	1
Ciao3	0.039312	0.435403	1
Impa1	0.039311	0.451873	1
Serinc1	0.039306	0.863478	1
Eci1	0.039304	0.037973	1
Kctd13	0.039268	0.386167	1
Ccdc97	0.039107	0.574489	1
Med24	0.039076	0.760406	1
Bdp1	0.039023	0.7963	1
Anks1	0.038987	0.891811	1
Med11	0.038939	0.749473	1
Parg	0.038889	0.896142	1
Marf1	0.038874	0.965188	1
Pigm	0.038869	0.665326	1
Trmt10c	0.038752	0.678667	1
Sesn3	0.038745	0.159417	1
Suds3	0.03872	0.49646	1
Aimp1	0.038717	0.89953	1
Ten1	0.038677	0.606714	1
Agpat2	0.038675	0.258006	1
Asl	0.038661	0.222342	1
Gosr2	0.038642	0.749078	1
Gm34794	0.038635	0.618325	1
Slc25a25	0.038566	0.761016	1
Pno1	0.038558	0.370301	1
H13	0.038549	0.409928	1
Mtor	0.038526	0.708682	1

Terf2	0.038514	0.827365	1
Nedd8	0.038438	0.319423	1
4932438A13I	0.038402	0.841787	1
Cpeb4	0.038398	0.710274	1
Camk2d	0.038369	0.939999	1
Sri	0.038299	0.863568	1
Fam210a	0.038194	0.645297	1
Coq9	0.038163	0.42143	1
Pced1b	0.038159	0.787211	1
Maco1	0.03811	0.329487	1
Gpn3	0.037968	0.734097	1
Rrp7a	0.037911	0.606853	1
Rnf13	0.037892	0.57534	1
Cpsf1	0.037788	0.807064	1
Snw1	0.037691	0.371556	1
Ndufs1	0.03766	0.6871	1
Dcakd	0.037575	0.52838	1
Chuk	0.037531	0.988096	1
Tma16	0.037492	0.691124	1
Zpr1	0.037446	0.397784	1
Mtfr1	0.037377	0.506047	1
Exosc5	0.03736	0.619467	1
Papolg	0.037328	0.842249	1
Timm10b	0.037289	0.334662	1
Slc25a19	0.037264	0.793195	1
Cox18	0.037166	0.592078	1
Eif3c	0.037157	0.830821	1
Scaf11	0.037136	0.466115	1
Sp3	0.037098	0.461363	1
Trp53i13	0.037095	0.786984	1
Mosmo	0.037094	0.538847	1
Zfp706	0.037079	0.448466	1
Zfp260	0.036996	0.804279	1
Mrpl32	0.036942	0.520325	1
Gm35037	0.03693	0.448577	1
Cbx6	0.036871	0.339134	1
Zfyve26	0.0368	0.522398	1
Tmcc1	0.036768	0.391963	1
Vwa8	0.03676	0.418079	1
Pradc1	0.036679	0.363012	1
Zmat2	0.036596	0.424084	1
Icosl	0.036521	0.317009	1
Cbx4	0.036495	0.978612	1
Rft1	0.036452	0.411292	1
Dnlz	0.036409	0.951504	1
Exoc1	0.036403	0.751727	1
Stard4	0.036399	0.415091	1
Hccs	0.036336	0.451291	1

Dimt1	0.036331	0.929516	1
Apex2	0.03628	0.852739	1
Mxd1	0.036276	0.233136	1
Ostc	0.03626	0.666104	1
Fam173a	0.036227	0.559182	1
Vps26a	0.036189	0.450012	1
Tbp	0.036167	0.436768	1
Pstpip1	0.036151	0.643497	1
Snap23	0.036119	0.921436	1
Acadsb	0.036117	0.559879	1
Fasn	0.036094	0.576941	1
A930015D03	0.036073	0.466245	1
Hivep1	0.035944	0.885812	1
Smpdl3a	0.035722	0.583035	1
Pea15a	0.035715	0.820124	1
Sipa1l3	0.035714	0.800826	1
Kmt2a	0.035706	0.343166	1
Rbm18	0.035691	0.176816	1
Vps52	0.03566	0.973599	1
Larp1b	0.035598	0.33806	1
Nbn	0.035596	0.356073	1
Zfand5	0.035591	0.629529	1
Rcor1	0.035554	0.264339	1
Styx	0.03553	0.401694	1
Otud6b	0.035501	0.258663	1
Tmem161a	0.035494	0.755436	1
Herpud2	0.035458	0.990427	1
Tsc1	0.035427	0.858245	1
Slc35a3	0.035367	0.887833	1
Traf3	0.035311	0.425911	1
Mllt6	0.035141	0.836002	1
Mcph1	0.035127	0.581336	1
Hsph1	0.035107	0.494312	1
Zfp282	0.035106	0.83761	1
Hif1an	0.035082	0.367158	1
Dexi	0.035077	0.812263	1
Erp44	0.035059	0.50537	1
Mast3	0.03501	0.763592	1
Dennd6a	0.03494	0.374496	1
Timm21	0.034892	0.715749	1
Paip2	0.034889	0.419473	1
Slc35d1	0.034883	0.920594	1
Zmynd11	0.03482	0.694682	1
Rab5a	0.034796	0.842309	1
Zcchc9	0.034775	0.02607	1
mt-Nd4l	0.034774	0.477501	1
Rnf10	0.034743	0.585511	1
Ccdc84	0.034691	0.393374	1

Miip	0.034582	0.722356	1
Ep400	0.034571	0.747364	1
Mrps27	0.034554	0.706324	1
Snrnp25	0.034542	0.356938	1
Bloc1s6	0.034515	0.727832	1
Fem1b	0.034493	0.760074	1
C030006K11	0.034477	0.966522	1
Smim26	0.034476	0.899059	1
Casp8	0.034454	0.646192	1
Edem3	0.034423	0.708102	1
Pdpk1	0.034352	0.948711	1
Skap2	0.03431	0.439242	1
Sh3kbp1	0.034268	0.845817	1
Gtf3c5	0.03425	0.492064	1
Ppp1r14b	0.034245	0.609827	1
Cds2	0.034237	0.670017	1
Zfp668	0.034212	0.40837	1
Xpo7	0.034207	0.983529	1
Hnrnpa1	0.034122	0.676738	1
Nol6	0.034071	0.692461	1
Zfp866	0.034064	0.735155	1
Etf1	0.034061	0.390975	1
Mrpl34	0.034059	0.493808	1
Immt	0.03404	0.554671	1
Zdhhc5	0.033994	0.664232	1
Retreg2	0.033983	0.463546	1
Abce1	0.033949	0.506068	1
Ercc4	0.033866	0.962755	1
Map3k7	0.033812	0.770428	1
Coa6	0.033786	0.949882	1
Rad54l2	0.033751	0.71379	1
Mrps24	0.033643	0.972481	1
Jade2	0.0336	0.488525	1
Ntpcr	0.033467	0.412229	1
Klf6	0.033458	0.991125	1
Polr3f	0.033421	0.837251	1
Ddx58	0.033371	0.987582	1
Ubfd1	0.033363	0.632835	1
Tmem183a	0.033363	0.736998	1
Mak16	0.033323	0.887412	1
Dnajc2	0.033272	0.966087	1
Gdap2	0.033252	0.559612	1
Gm5914	0.033207	0.556426	1
Gabarapl1	0.033178	0.861976	1
Coq3	0.033164	0.623017	1
Ubr3	0.033137	0.424782	1
Gpsm3	0.033133	0.745029	1
Tmem50b	0.033055	0.43767	1

Mrps9	0.033026	0.566123	1
Brf2	0.03301	0.511527	1
Mrm1	0.033	0.867965	1
Zc3h10	0.032995	0.314127	1
Dclre1c	0.032892	0.848578	1
Ddx20	0.032879	0.762975	1
Ibtk	0.032841	0.888617	1
Kcna3	0.032829	0.606767	1
Pnkp	0.03277	0.964957	1
Tomm7	0.032705	0.75692	1
Clpp	0.032688	0.809936	1
Mtg1	0.032661	0.727753	1
Flcn	0.032634	0.521158	1
Mri1	0.032599	0.586716	1
Cmc1	0.032529	0.809081	1
Cep162	0.032505	0.92537	1
Uqcc1	0.032482	0.855724	1
Gpatch2	0.032449	0.835769	1
Sys1	0.032415	0.221505	1
Rpap3	0.032332	0.961435	1
Il21r	0.03221	0.660131	1
Exo5	0.03217	0.477249	1
Taf1a	0.032138	0.426964	1
Qdpr	0.032126	0.963983	1
Cul5	0.032121	0.529373	1
2700097O09	0.032015	0.809516	1
Higd2a	0.031878	0.72578	1
Alkbh1	0.031844	0.831207	1
Brd9	0.031799	0.614735	1
Suclg2	0.031766	0.526834	1
Arhgap39	0.031762	0.214758	1
Prr7	0.031752	0.590697	1
Rnf138	0.031719	0.861409	1
Spty2d1	0.031704	0.36299	1
Lrch1	0.031677	0.867776	1
Mrpl48	0.031671	0.77803	1
Slc8b1	0.031666	0.866657	1
Bpgm	0.031611	0.644083	1
Gpr180	0.03159	0.7858	1
Prep	0.031578	0.950704	1
Yeats2	0.031572	0.884531	1
Itfg2	0.031554	0.763142	1
Zfp263	0.031506	0.694186	1
Vps72	0.0315	0.934121	1
Ppie	0.031486	0.46903	1
Paxbp1	0.031412	0.905897	1
Fam160b2	0.031376	0.476604	1
Micall1	0.031327	0.391983	1

Iqce	0.031313	0.85282	1
Sppl2b	0.031313	0.808979	1
Glo1	0.031291	0.932122	1
Tmem260	0.031273	0.624069	1
Mettl4	0.031273	0.359756	1
Nle1	0.031235	0.677964	1
Polr3a	0.031229	0.757099	1
Sympk	0.031227	0.993068	1
Upf3a	0.031186	0.546459	1
Dpf2	0.03117	0.81131	1
Cdc25a	0.031146	0.666186	1
Shmt2	0.031083	0.597892	1
Atp2a2	0.031075	0.433527	1
Dhx35	0.031072	0.859759	1
Pus3	0.031067	0.620215	1
Cox6c	0.031032	0.795505	1
Oard1	0.031029	0.97742	1
Dhdds	0.031001	0.947497	1
Alkbh7	0.030905	0.580047	1
Agps	0.030826	0.947555	1
Gclm	0.030725	0.45883	1
Nudt16	0.030724	0.35893	1
Nol9	0.030685	0.375283	1
Dhx29	0.030683	0.559937	1
Pot1a	0.030589	0.703342	1
Mga	0.030587	0.776876	1
Timm8a1	0.030569	0.888137	1
Thap4	0.030488	0.488142	1
Gsr	0.030485	0.254276	1
Twf1	0.030474	0.567535	1
Ppp2cb	0.030468	0.785091	1
Rlf	0.030441	0.281433	1
Asna1	0.030397	0.885719	1
Tmem179b	0.030295	0.45918	1
Whamm	0.030236	0.944216	1
Dnmt3a	0.030205	0.776403	1
Slc17a9	0.030202	0.688496	1
Tsfm	0.030197	0.998198	1
Kpna1	0.030075	0.666563	1
Prickle3	0.030052	0.319957	1
Arhgap12	0.030031	0.446557	1
Ccdc171	0.02983	0.753823	1
Uhmk1	0.029821	0.940959	1
Cyth2	0.029734	0.912572	1
Arpc1a	0.029718	0.209544	1
Rpl39	0.029706	0.521858	1
Txndc11	0.029681	0.858022	1
Retreg3	0.029657	0.738782	1

Tacc2	0.029583	0.372522	1
Atg4b	0.029546	0.905784	1
Arfgef2	0.029544	0.658267	1
Pik3r4	0.029417	0.933308	1
Xcl1	0.029384	0.211936	1
Naa60	0.029186	0.973717	1
Nploc4	0.029162	0.59131	1
Sap30bp	0.029159	0.572716	1
Snapc1	0.029126	0.803718	1
Cnot4	0.029125	0.748096	1
Zfp983	0.029092	0.742023	1
Pigf	0.029049	0.936205	1
Krit1	0.029014	0.42807	1
Phf7	0.028988	0.847421	1
Acadvl	0.028963	0.986162	1
Gm4876	0.028875	0.818144	1
Kmt2d	0.028862	0.512405	1
Uap1	0.028833	0.331782	1
Pi4ka	0.02874	0.79812	1
Sap130	0.028722	0.564657	1
Mpst	0.028638	0.873602	1
Zdhhc13	0.028614	0.810236	1
Gfi1	0.028584	0.99388	1
Ahsa2	0.028575	0.887787	1
Hnrnph2	0.028509	0.52017	1
Gt(ROSA)26Sc	0.028471	0.460378	1
Ogg1	0.028421	0.428003	1
Ube2q2	0.028402	0.40572	1
Pcgf6	0.028386	0.58367	1
Rpap1	0.028358	0.637239	1
Sqle	0.028333	0.425265	1
Stx17	0.028318	0.810499	1
Ankrd54	0.02828	0.345951	1
Nat2	0.028238	0.620753	1
Ppcdc	0.028174	0.979976	1
Snx27	0.028173	0.640992	1
0610030E20I	0.028172	0.440719	1
Ilrun	0.028135	0.392133	1
Wdr61	0.028127	0.813618	1
Zfp410	0.028127	0.913714	1
Nkapd1	0.028087	0.928622	1
Nceh1	0.028075	0.647878	1
Hint3	0.028068	0.815412	1
Alg3	0.028009	0.723163	1
5830428M24	0.028009	0.887365	1
Topors	0.027966	0.851346	1
Eif1ad	0.027897	0.835678	1
Sem1	0.027875	0.916426	1

Cmas	0.027825	0.65162	1
Tmem223	0.027785	0.63521	1
Polr1e	0.027752	0.890519	1
Dera	0.02774	0.591405	1
Mipol1	0.027679	0.50563	1
0610009B22	0.027571	0.653685	1
Glrx2	0.027569	0.566646	1
Mphosph6	0.027558	0.747878	1
Nacc1	0.027502	0.941367	1
Rnasek	0.027378	0.205846	1
Taco1	0.02731	0.921853	1
Rab10	0.027304	0.448986	1
Msra	0.027274	0.794669	1
Sumo3	0.027273	0.143637	1
Zfp84	0.027191	0.802764	1
Sergef	0.027177	0.12512	1
Rnf25	0.027173	0.758557	1
Heatr5a	0.02711	0.245948	1
Btg3	0.027089	0.533688	1
Echs1	0.027079	0.632444	1
M6pr	0.027045	0.531618	1
Dyrk2	0.027003	0.866824	1
Slc35a2	0.026992	0.979019	1
Jade1	0.026859	0.367814	1
Gm16033	0.026841	0.868412	1
Ythdf3	0.026818	0.412473	1
Srrm1	0.026785	0.953328	1
Tbc1d23	0.026783	0.60015	1
Coq7	0.026707	0.363193	1
Gm15821	0.026687	0.953165	1
Lzic	0.026652	0.629705	1
Atr	0.026637	0.431701	1
Emc6	0.026597	0.961779	1
Cars2	0.026517	0.693652	1
Ranbp9	0.026515	0.956035	1
Fam53c	0.026503	0.840364	1
Entr1	0.026419	0.28699	1
Smad2	0.026401	0.793405	1
Gosr1	0.026377	0.917463	1
Vamp4	0.026308	0.691481	1
Hmgcl	0.026293	0.951379	1
Rab8a	0.026266	0.903428	1
Synrg	0.026248	0.847082	1
Sel1l	0.026244	0.538414	1
Narf	0.026186	0.563859	1
Cnih1	0.026123	0.086631	1
Rbm47	0.026113	0.735663	1
Med27	0.026107	0.934726	1

Mrpl36	0.026082	0.818922	1
Isy1	0.02598	0.907494	1
Nop9	0.02593	0.293449	1
Nrbf2	0.025908	0.800614	1
Zdhhc7	0.025799	0.653115	1
Ncoa7	0.025709	0.793566	1
Tfb2m	0.025689	0.618021	1
Sbno2	0.025653	0.854877	1
Ltn1	0.02563	0.802832	1
Pus1	0.025597	0.931655	1
Jakmip1	0.025596	0.896794	1
Spopl	0.025575	0.553482	1
Ing5	0.025564	0.393694	1
Trappc13	0.02552	0.888111	1
Etnk1	0.02552	0.766968	1
Aifm1	0.025479	0.611572	1
Ccdc134	0.025473	0.791351	1
Ctdsp2	0.025469	0.430399	1
Aopep	0.025446	0.3668	1
Atad3a	0.025357	0.309399	1
Fbxo32	0.025328	0.672318	1
Rexo2	0.025296	0.467952	1
Cradd	0.025241	0.609967	1
2810004N23	0.025234	0.78856	1
Mettl21a	0.025205	0.422943	1
Mtpap	0.025145	0.601356	1
Sacs	0.025103	0.600273	1
Coq6	0.02501	0.787425	1
Ighmbp2	0.024996	0.618577	1
Tgfbr2	0.024974	0.917743	1
Nsd3	0.024956	0.576626	1
Rnf38	0.024824	0.985603	1
Fbrs	0.024821	0.279357	1
Zfp740	0.024782	0.830187	1
Tspo	0.024654	0.945157	1
Fam214a	0.024613	0.575832	1
Unkl	0.024595	0.670912	1
Tut1	0.024534	0.377987	1
Pold3	0.024504	0.8195	1
Kars	0.024503	0.705059	1
Psmd2	0.024486	0.494229	1
Nrd1	0.024449	0.930171	1
Sacm1l	0.024444	0.966582	1
Hcfc2	0.02443	0.909227	1
Zfand1	0.024411	0.777939	1
Ndufb5	0.02439	0.954803	1
Ncoa1	0.024347	0.697211	1
Pisd	0.024157	0.950333	1

Nr2c2ap	0.024155	0.572213	1
Hmbs	0.024117	0.917695	1
Vmn2r96	0.024104	0.652253	1
Hdhd5	0.024084	0.757895	1
Pan2	0.02405	0.599353	1
Lcmt1	0.024049	0.563412	1
Rundc1	0.02399	0.868714	1
Trp53bp1	0.023939	0.744035	1
Mrps25	0.023922	0.873282	1
Zbtb44	0.02389	0.643484	1
Scnm1	0.023848	0.447776	1
Zfp639	0.023837	0.617868	1
Rnf40	0.023801	0.961993	1
Pptc7	0.023777	0.948459	1
Mms19	0.023614	0.83804	1
Nlrc3	0.023569	0.901959	1
Eloc	0.023536	0.959376	1
Pdcd5	0.023527	0.600587	1
4930469K13	0.023522	0.680788	1
5330438D12	0.023498	0.650284	1
Gtf2h1	0.023448	0.870498	1
Foxred1	0.023346	0.614271	1
Gvin1	0.023314	0.852599	1
Qars	0.023247	0.936309	1
1110059G10	0.023166	0.977605	1
Yipf6	0.023124	0.800779	1
Klhdc3	0.023036	0.920763	1
Acsl5	0.023013	0.690583	1
Elp3	0.023005	0.838545	1
Dcp1a	0.022993	0.690903	1
Actr5	0.022935	0.874739	1
Zmym4	0.02287	0.658587	1
Mad2l2	0.022865	0.755156	1
Impa2	0.022838	0.425252	1
Pfdn4	0.022805	0.87656	1
Ngdn	0.022712	0.901701	1
Psmd1	0.022706	0.841749	1
Hnrnph3	0.022687	0.688833	1
Tcf20	0.022606	0.801467	1
Elk3	0.022564	0.937438	1
Rftn1	0.022563	0.271493	1
Mrpl27	0.022514	0.790603	1
Ssh1	0.0225	0.960987	1
Gtf2f1	0.022449	0.761075	1
Pms1	0.022407	0.823871	1
Sdr39u1	0.022399	0.792392	1
Slc39a3	0.022366	0.777302	1
D3Ertd751e	0.022359	0.866065	1

Exoc5	0.022275	0.42068	1
Ankrd13a	0.022203	0.730209	1
Zfp687	0.022074	0.791429	1
Pom121	0.02205	0.357539	1
Rrm2b	0.022038	0.797344	1
Polr2b	0.022038	0.817103	1
Mynn	0.022019	0.584292	1
Ybx1	0.022001	0.713257	1
Ccdc51	0.021917	0.541004	1
Cbx8	0.021861	0.691921	1
Zfp142	0.021852	0.833226	1
Tmed5	0.021835	0.985785	1
Rbm8a	0.021821	0.820735	1
Mfap1a	0.021814	0.837775	1
Prmt9	0.021806	0.744939	1
Rrnad1	0.021769	0.82742	1
Nf2	0.021726	0.715803	1
Acbd4	0.021713	0.637094	1
Sirt3	0.021693	0.278926	1
Rnaseh2a	0.0216	0.233183	1
Brms1l	0.021595	0.750462	1
Mgat4a	0.021571	0.851054	1
Cdk19	0.02154	0.992747	1
Emsy	0.021534	0.68764	1
Csgalnact2	0.021497	0.503111	1
Ddhd2	0.02142	0.718925	1
Rbmxl1	0.0214	0.393695	1
Eef1e1	0.021395	0.914415	1
Ppid	0.021379	0.985646	1
Mfhas1	0.021296	0.587811	1
Ift20	0.021281	0.631142	1
Pafah1b2	0.021273	0.356285	1
Polr3gl	0.021257	0.902906	1
Rnf19b	0.021212	0.496964	1
Strada	0.02105	0.98937	1
Mon1b	0.021041	0.735712	1
Gm28935	0.021013	0.942734	1
Epn1	0.02101	0.232368	1
Vkorc1	0.020933	0.092602	1
Rrp36	0.02092	0.926412	1
Eif1ax	0.020831	0.738519	1
Fam160b1	0.020809	0.929455	1
Pcnx3	0.020787	0.685904	1
Nfs1	0.020776	0.963549	1
Brd7	0.020763	0.986386	1
Tmtc4	0.020729	0.775811	1
Paqr7	0.020724	0.457489	1
2510002D24	0.0207	0.517654	1

Tmem208	0.020687	0.760074	1
Fgd3	0.020685	0.988838	1
Map4k4	0.020676	0.28519	1
Tnfrsf1b	0.020673	0.963944	1
Zfp444	0.02064	0.960757	1
Dram2	0.020626	0.672221	1
Syap1	0.020617	0.646581	1
Dolpp1	0.020594	0.940911	1
Ankrd52	0.020572	0.769347	1
Pabpc4	0.020542	0.778865	1
Xrcc5	0.020481	0.778402	1
Cops6	0.020459	0.624292	1
Dctn4	0.020452	0.569622	1
Golph3l	0.020404	0.939374	1
Ttc7b	0.020355	0.830497	1
Mrtfb	0.02032	0.712706	1
Tmem219	0.020296	0.777584	1
Slc25a13	0.020177	0.97907	1
Zfp524	0.020142	0.856595	1
Mien1	0.02013	0.829541	1
Plekhm1	0.020088	0.320175	1
Tti2	0.02005	0.637605	1
Tab1	0.02005	0.777237	1
Tmx4	0.020021	0.572843	1
Hip1r	0.019972	0.54881	1
Enox2	0.019897	0.931461	1
Rad51d	0.019757	0.847355	1
Ywhag	0.019728	0.880314	1
Tpgs1	0.019587	0.476321	1
Mospd2	0.019569	0.723131	1
Rpp21	0.019557	0.534435	1
Qrsl1	0.019484	0.378067	1
Gtf2ird2	0.019474	0.854916	1
Stk24	0.019393	0.988965	1
Zcchc8	0.019368	0.984039	1
Farsb	0.019365	0.724552	1
Dffb	0.019345	0.995915	1
Fam98a	0.019311	0.840026	1
Fam114a2	0.019276	0.958415	1
Ift80	0.019256	0.93635	1
Fbxo46	0.019233	0.858587	1
Gm3448	0.019209	0.493543	1
Usp7	0.019068	0.969105	1
Dcaf11	0.019008	0.795559	1
Mrpl57	0.018953	0.930255	1
Gpr174	0.018911	0.920358	1
Ptar1	0.018864	0.466718	1
Polr2j	0.018815	0.788823	1

Igf2r	0.018715	0.743989	1
Pik3ap1	0.018701	0.950775	1
Siae	0.018693	0.815153	1
Arl6ip4	0.018661	0.207313	1
Mul1	0.018602	0.875238	1
Immp1l	0.018597	0.989678	1
Cactin	0.018582	0.621559	1
Ttc9c	0.018565	0.681921	1
Zfp346	0.018434	0.516438	1
Mphosph8	0.018428	0.560236	1
Nfkbie	0.018389	0.265601	1
Eif2ak4	0.018383	0.440898	1
Slc39a9	0.018287	0.765391	1
2310033P09I	0.018273	0.63244	1
Clk1	0.018255	0.725656	1
Sh3bp2	0.018244	0.844863	1
Prmt1	0.018242	0.391148	1
Ankib1	0.018202	0.736064	1
Gtf2e1	0.018202	0.697233	1
Zfp322a	0.018189	0.876777	1
Pogz	0.018178	0.53391	1
Zcchc4	0.018172	0.898549	1
Czib	0.018172	0.920268	1
Man2b2	0.018153	0.917306	1
Mphosph10	0.018129	0.850525	1
Gemin2	0.018096	0.389873	1
Trbc1	0.017963	0.275472	1
Nus1	0.017944	0.658792	1
Ctdp1	0.017939	0.951616	1
Irf3	0.017934	0.790462	1
Platr25	0.01787	0.47019	1
Ubp1	0.017848	0.838461	1
Atxn7l1	0.017835	0.495554	1
Clcn7	0.0178	0.445224	1
Gm2245	0.017754	0.770814	1
Sec22b	0.017711	0.852618	1
Nipa2	0.017634	0.654796	1
Smim14	0.017573	0.631417	1
Txndc17	0.017563	0.368939	1
Btbd8	0.017553	0.631894	1
Pum3	0.017526	0.843844	1
Stx8	0.017524	0.990531	1
Ccdc174	0.01748	0.880656	1
Prmt2	0.017479	0.394526	1
Ankrd11	0.017438	0.704514	1
Med29	0.017412	0.799305	1
Neil1	0.017372	0.550312	1
Cdk12	0.017325	0.69042	1

Lonp1	0.017318	0.490442	1
Mybbp1a	0.017259	0.336057	1
Bcl2l1	0.017236	0.678877	1
Ppa2	0.017136	0.652029	1
Usf3	0.017135	0.889514	1
Rpe	0.017113	0.890135	1
Ip6k2	0.017109	0.605428	1
Rprd1b	0.017088	0.681745	1
Ftx	0.017087	0.832397	1
Tbccd1	0.01707	0.693979	1
Tom1	0.017065	0.731742	1
Calhm2	0.017017	0.829024	1
Tsc22d3	0.016823	0.219073	1
Cstb	0.016784	0.683297	1
Pcgf1	0.016757	0.699773	1
Mvb12b	0.016755	0.23916	1
Mrpl16	0.016733	0.88314	1
Fbxo21	0.01669	0.916837	1
Psmd13	0.016592	0.874971	1
Dcxr	0.01657	0.994564	1
Borcs6	0.016456	0.63522	1
Lrig2	0.016452	0.494005	1
Wdr41	0.016434	0.039628	1
Prelid2	0.01643	0.819829	1
Pdcl3	0.016427	0.975361	1
Otud7b	0.016381	0.714613	1
Atp5md	0.016359	0.998324	1
Tada2a	0.016353	0.385343	1
Atg2b	0.016339	0.984957	1
Pacs1	0.016324	0.918706	1
Etfdh	0.016311	0.780416	1
Eif3g	0.016229	0.844363	1
Tmem70	0.016195	0.511443	1
Tnip1	0.016176	0.935247	1
Rps6ka4	0.01617	0.685081	1
Patz1	0.01612	0.851687	1
Rnf11	0.01611	0.773876	1
Bud23	0.016053	0.678794	1
Srsf4	0.016044	0.812975	1
Dvl2	0.01598	0.748287	1
Ubl5	0.015972	0.901382	1
Ahdc1	0.015881	0.935356	1
Dnajb1	0.015852	0.798063	1
Ripk3	0.015766	0.707653	1
Map2k7	0.015707	0.93275	1
5430427O19	0.015671	0.151453	1
Top1	0.015649	0.964085	1
2510039O18	0.01556	0.429382	1

Klhl25	0.015539	0.896633	1
Trmt2a	0.01545	0.887735	1
Vps37c	0.015435	0.651797	1
Tubgcp6	0.015343	0.711027	1
Upf3b	0.015339	0.870856	1
Bcat2	0.015337	0.727605	1
Llgl1	0.015336	0.697744	1
Gnpnat1	0.015316	0.592908	1
Atl3	0.015315	0.349922	1
Herc2	0.015243	0.947643	1
Fgfr1op	0.015222	0.870937	1
Xdh	0.01514	0.683972	1
Ankle2	0.01507	0.923943	1
Eif2a	0.01506	0.999107	1
Slirp	0.014958	0.841884	1
Sin3b	0.014908	0.597031	1
Trim26	0.014893	0.796988	1
Pym1	0.014885	0.602664	1
Ehmt2	0.014842	0.759348	1
Cep63	0.014811	0.732106	1
Commd9	0.014808	0.775439	1
Tmem245	0.014804	0.307785	1
Serpine2	0.014739	0.077892	1
Brwd1	0.014728	0.732354	1
Ikbke	0.014608	0.938568	1
Vps45	0.014595	0.86601	1
Mrpl1	0.014375	0.964903	1
Ube2j1	0.014362	0.903879	1
Atp6v0c	0.014358	0.910147	1
Prr14	0.014356	0.789738	1
Tmx3	0.014354	0.71947	1
Ccdc127	0.014352	0.775179	1
Pias2	0.014264	0.87298	1
Ppif	0.014253	0.973763	1
Atraid	0.01418	0.644483	1
Spout1	0.014113	0.980693	1
Spast	0.014112	0.83257	1
Oxct1	0.014079	0.999823	1
Mysm1	0.014075	0.972465	1
Zfp146	0.014031	0.396804	1
Atg16l1	0.014023	0.821449	1
Cnst	0.014004	0.193972	1
Chm	0.013967	0.854204	1
Paxx	0.013953	0.954094	1
Eef2kmt	0.013952	0.589533	1
Pkn1	0.013915	0.235932	1
Emc1	0.013843	0.913999	1
Rbm7	0.013841	0.756059	1

Dkc1	0.013802	0.962068	1
Neu1	0.013794	0.243312	1
Zbtb9	0.013779	0.687827	1
Letmd1	0.013751	0.957829	1
Prpsap2	0.013729	0.88067	1
Dynlt1c	0.013714	0.948395	1
Cib1	0.013699	0.910383	1
Rbfa	0.013696	0.339064	1
Vav3	0.01361	0.768536	1
Pvt1	0.013604	0.781045	1
Cox16	0.01352	0.569055	1
Fadd	0.013505	0.963007	1
Tmem185b	0.013445	0.77605	1
Mterf1a	0.013432	0.400271	1
Dnase2a	0.013397	0.492355	1
Zfp335	0.013395	0.473833	1
Trp53i11	0.013345	0.60591	1
Nt5c2	0.013345	0.913826	1
Nt5c3	0.01333	0.333707	1
Kcnn4	0.013286	0.488494	1
Prdx2	0.013268	0.450525	1
C1galt1c1	0.013232	0.994203	1
Mrto4	0.013212	0.998543	1
Smad4	0.013175	0.430303	1
Rab3ip	0.013151	0.982766	1
Crybg3	0.013136	0.383941	1
Dld	0.013135	0.816793	1
Sik1	0.01306	0.189645	1
Golga2	0.013043	0.828803	1
Pik3c3	0.013026	0.706331	1
Ada	0.013021	0.507007	1
Otulin	0.012982	0.806833	1
Aga	0.012873	0.974027	1
Zswim1	0.01282	0.947789	1
Tspan14	0.012794	0.391652	1
Gpank1	0.01278	0.731483	1
Rmnd1	0.012774	0.784966	1
Ppp1r8	0.012762	0.884752	1
Tnpo3	0.012748	0.912654	1
Mettl14	0.012695	0.897517	1
Thap1	0.01269	0.701634	1
Ube2w	0.012659	0.721811	1
AI413582	0.01262	0.637983	1
Ddx28	0.012574	0.952061	1
Kat8	0.012523	0.176135	1
Armc8	0.012509	0.956961	1
Acap2	0.012476	0.945945	1
Nudt8	0.012419	0.577557	1

Tjap1	0.012401	0.811446	1
Slc25a10	0.012315	0.718447	1
Cep41	0.012286	0.464184	1
Ppp1r2	0.012264	0.812573	1
Ripk1	0.012245	0.501905	1
Mgat2	0.012242	0.693144	1
Dnajc10	0.012142	0.795164	1
Wdr83	0.012045	0.906045	1
Gm16286	0.01204	0.731096	1
Fdft1	0.012022	0.676862	1
Ddx31	0.012005	0.806055	1
Gm29243	0.011996	0.405836	1
Gm9856	0.011993	0.526096	1
Cog8	0.011989	0.659798	1
Letm1	0.011977	0.885175	1
Cep95	0.011963	0.673488	1
Cdc14a	0.011962	0.607739	1
Tbc1d2b	0.011948	0.899148	1
Tpra1	0.011857	0.933491	1
Prmt5	0.011836	0.840635	1
Usp32	0.011734	0.692203	1
Tbcd	0.011727	0.991836	1
Zfp871	0.011662	0.866903	1
Clasp1	0.011648	0.9555	1
Tssc4	0.011587	0.917117	1
1600010M07	0.011499	0.90392	1
Rasa1	0.01149	0.483259	1
Il4ra	0.011486	0.809322	1
Utp6	0.011365	0.725573	1
Kansl3	0.011342	0.513832	1
Wdr46	0.011286	0.828872	1
Nars2	0.011274	0.841856	1
Cela1	0.011264	0.880137	1
Ubap2	0.011235	0.962233	1
Pygo2	0.011189	0.98902	1
Ciao2b	0.01115	0.910496	1
Tomm70a	0.011136	0.621182	1
Wdr37	0.011045	0.914633	1
Fxr1	0.011023	0.651637	1
Dapp1	0.011011	0.840024	1
Cebpzos	0.01098	0.18223	1
Ss18l2	0.010933	0.877545	1
Fnip1	0.010917	0.652414	1
Adprhl2	0.010904	0.784926	1
Smim7	0.010802	0.953632	1
Tent5a	0.010781	0.502216	1
Myg1	0.010733	0.9177	1
Ddx49	0.010731	0.910334	1

Sft2d2	0.01062	0.836884	1
Tfb1m	0.010583	0.690822	1
Appl1	0.010555	0.382622	1
Rpl38	0.010536	0.825236	1
Fam117b	0.010503	0.701554	1
Urm1	0.010499	0.270266	1
Pja1	0.010387	0.831617	1
Snap29	0.010335	0.494038	1
Asb1	0.010286	0.667021	1
Drg2	0.010237	0.665159	1
Fam222b	0.010212	0.655971	1
Noc2l	0.0102	0.589161	1
Zswim8	0.0102	0.297366	1
Otud4	0.010176	0.986537	1
Cdc40	0.010136	0.140071	1
Gemin8	0.010109	0.563424	1
Apc	0.010025	0.573717	1
Slain2	0.010014	0.543193	1
Zfp808	-0.01	0.332212	1
Osbp	-0.01004	0.653745	1
Itgal	-0.01012	0.772146	1
Prpf19	-0.01015	0.538447	1
Brap	-0.01015	0.616785	1
Ddx19a	-0.01026	0.807524	1
Gphn	-0.01028	0.377175	1
Dicer1	-0.01029	0.569382	1
Fem1c	-0.01034	0.712198	1
Map2k5	-0.01034	0.537259	1
Naga	-0.01035	0.460046	1
Ap1ar	-0.01035	0.079419	1
Hspa4	-0.01041	0.971789	1
Wrap73	-0.01041	0.495174	1
Ablim1	-0.01042	0.956612	1
Bms1	-0.01042	0.814538	1
Ube3b	-0.01044	0.583138	1
Clk3	-0.01044	0.694719	1
Cox11	-0.01045	0.520503	1
Borcs8	-0.01057	0.985993	1
Cfap20	-0.01064	0.986256	1
Scaper	-0.01066	0.750069	1
Kdm1b	-0.0107	0.445833	1
Phf5a	-0.0107	0.831737	1
Cdc23	-0.01075	0.447675	1
Dnajc21	-0.01077	0.641607	1
Abhd14a	-0.01086	0.336397	1
Smchd1	-0.01087	0.69863	1
Dglucy	-0.01089	0.46477	1
G3bp2	-0.0109	0.570111	1

Ube2n	-0.01091	0.828066	1
Rps27rt	-0.01092	0.956743	1
Gm9844	-0.01097	0.666755	1
Ide	-0.01099	0.927065	1
Polr2h	-0.011	0.584569	1
Gtf2a1	-0.01103	0.686585	1
Lrrc41	-0.01103	0.886825	1
Naxe	-0.01114	0.635614	1
Grn	-0.01116	0.0583	1
Exd2	-0.0112	0.576797	1
Rapgef2	-0.01126	0.85461	1
Shfl	-0.01126	0.779728	1
Gm15472	-0.01129	0.531034	1
Mob3a	-0.01133	0.905397	1
Gspt1	-0.01135	0.961163	1
Atxn10	-0.01136	0.634485	1
Nup214	-0.01138	0.90457	1
Bop1	-0.01143	0.654793	1
Tnfrsf13b	-0.01145	0.058131	1
Ankhd1	-0.01146	0.609323	1
Fam193a	-0.01146	0.812375	1
Lcp1	-0.01155	0.828736	1
Esco1	-0.01159	0.639314	1
Usp50	-0.01162	0.391719	1
Xrcc1	-0.01166	0.517528	1
Dnajc11	-0.01171	0.553458	1
Gm15489	-0.0118	0.455937	1
Slc2a1	-0.01182	0.993145	1
Rab11fip4	-0.01187	0.654632	1
Wdr44	-0.01194	0.908528	1
Fem1a	-0.01196	0.786129	1
Dele1	-0.01199	0.877032	1
Timm44	-0.01199	0.625476	1
Bak1	-0.012	0.924079	1
Prrc1	-0.01203	0.442743	1
Rfesd	-0.01207	0.974198	1
Polr1c	-0.01207	0.985142	1
Zfp934	-0.01219	0.810535	1
Lmf1	-0.01221	0.84299	1
Sap30	-0.01221	0.953605	1
Slc28a2	-0.01224	0.560933	1
Tmem80	-0.01224	0.441419	1
Man1a2	-0.01229	0.850173	1
Rab1a	-0.01229	0.553109	1
Supv3l1	-0.01231	0.617912	1
Mtrf1l	-0.01238	0.565266	1
Itgb1bp1	-0.01242	0.876926	1
Usp36	-0.01243	0.666559	1

Fyttd1	-0.01248	0.784253	1
H2-T3	-0.0125	0.565775	1
Clptm1	-0.01252	0.836245	1
Xpnpep1	-0.01256	0.62762	1
Hk2	-0.01257	0.309242	1
Gpat4	-0.01259	0.997181	1
Phkb	-0.01268	0.975743	1
Nomo1	-0.0127	0.593011	1
Gatad2a	-0.01271	0.340642	1
Preb	-0.01276	0.957474	1
Zfp287	-0.01279	0.991532	1
Fdx1	-0.01293	0.649975	1
Klc2	-0.01294	0.920185	1
Aco2	-0.01295	0.847464	1
Osgep	-0.01296	0.467946	1
Ptp4a3	-0.01303	0.764326	1
Zfp326	-0.01303	0.728804	1
Anxa7	-0.01306	0.739796	1
Sdad1	-0.01311	0.937174	1
6430590A07I	-0.01324	0.99506	1
Tmem181a	-0.0133	0.562233	1
Lin37	-0.01331	0.906265	1
Txlng	-0.01335	0.588784	1
Gpn2	-0.01336	0.659263	1
Alkbh5	-0.0134	0.569756	1
Parp3	-0.01343	0.877861	1
Rpl35	-0.01344	0.98423	1
Med17	-0.01345	0.464393	1
Creb3	-0.01347	0.892577	1
Usp47	-0.01347	0.939769	1
Psmc1	-0.01352	0.983983	1
Cox19	-0.01352	0.675738	1
Mydgf	-0.01356	0.011413	1
Tbc1d8b	-0.01357	0.470903	1
H2afj	-0.01359	0.873576	1
Rgl2	-0.01363	0.547156	1
Tex261	-0.01363	0.629104	1
Acin1	-0.01373	0.877336	1
Zkscan6	-0.01376	0.578534	1
Anapc10	-0.01379	0.797019	1
Nsrp1	-0.01382	0.614223	1
Adora2a	-0.01383	0.85092	1
Ptpn2	-0.01384	0.774388	1
Ncl	-0.01384	0.516882	1
Cep57l1	-0.01386	0.287391	1
Dynlrb1	-0.01401	0.733383	1
Mettl5	-0.01402	0.633676	1
C1galt1	-0.01403	0.588274	1

2310057M21	-0.01407	0.981608	1
Cyren	-0.01408	0.432518	1
Vrk1	-0.01413	0.609652	1
Clcf1	-0.01421	0.551156	1
Cd200r1	-0.01424	0.626599	1
Zfp511	-0.01428	0.829119	1
Rhbdf2	-0.01432	0.858483	1
Ddt	-0.01435	0.34744	1
Eif2d	-0.01436	0.297569	1
Fars2	-0.01441	0.682594	1
Abcc4	-0.01444	0.642487	1
Ube2i	-0.01447	0.554657	1
Nr1h2	-0.01451	0.371348	1
Zbtb41	-0.01458	0.325981	1
Limk1	-0.01458	0.507032	1
Fbxo33	-0.01463	0.991552	1
Dcaf13	-0.01472	0.868915	1
Vma21	-0.01481	0.475059	1
Lamtor5	-0.01489	0.755559	1
Gng5	-0.01495	0.83121	1
Kat5	-0.01496	0.411949	1
H3f3b	-0.01497	0.506599	1
Sidt2	-0.015	0.53273	1
Rpf1	-0.015	0.578951	1
Golt1b	-0.015	0.12788	1
D11Wsu47e	-0.01502	0.333137	1
Nop16	-0.01505	0.996496	1
Alg2	-0.01505	0.767862	1
Mocs2	-0.01506	0.878607	1
Mlf2	-0.01506	0.761686	1
Atp6v0d1	-0.01507	0.974092	1
Uxs1	-0.0151	0.761318	1
Slc9a1	-0.01512	0.247644	1
Aasdhppt	-0.01516	0.592455	1
Grpel1	-0.01523	0.695701	1
Fkbp1a	-0.01523	0.716848	1
Twsg1	-0.01532	0.895324	1
Rars2	-0.01533	0.923757	1
Supt7l	-0.01534	0.45661	1
Naa10	-0.01537	0.695746	1
Amfr	-0.01548	0.633816	1
Bmp2k	-0.01553	0.953931	1
Nsmce4a	-0.01557	0.279154	1
Mrpl3	-0.01558	0.402777	1
Gm48719	-0.01558	0.719981	1
Kat7	-0.01559	0.858174	1
Cwf19l1	-0.01562	0.52699	1
Prkcz	-0.01568	0.121003	1

Fbxl6	-0.01576	0.235964	1
Timmdc1	-0.01581	0.685563	1
Dcun1d2	-0.01582	0.871408	1
mt-Nd6	-0.01584	0.561618	1
Emc4	-0.01588	0.683326	1
Fam118b	-0.01607	0.584253	1
D230025D16	-0.01607	0.341649	1
Usp24	-0.0161	0.322779	1
Phf23	-0.01611	0.258509	1
Sec13	-0.01618	0.821359	1
R3hdm4	-0.01619	0.816768	1
Gm27008	-0.01625	0.711478	1
Sirt1	-0.01627	0.356517	1
Tspyl1	-0.0163	0.679035	1
Washc3	-0.01639	0.308614	1
Gtf2f2	-0.0164	0.978773	1
Trrap	-0.01642	0.952882	1
Srcap	-0.0165	0.55106	1
Rac2	-0.01651	0.402626	1
Jazf1	-0.01653	0.528352	1
Ccny	-0.01653	0.904199	1
Cfdp1	-0.01659	0.703181	1
Mief1	-0.01667	0.469625	1
Nelfcd	-0.01673	0.586807	1
Ehd3	-0.01675	0.854837	1
Fibp	-0.01675	0.60697	1
Tufm	-0.01684	0.828957	1
Cdk8	-0.01689	0.003589	1
Cfap298	-0.01692	0.889613	1
Snx19	-0.01695	0.267304	1
Arf6	-0.01696	0.807167	1
Acss2	-0.01703	0.170541	1
Trpm7	-0.01705	0.846134	1
Gtf2h5	-0.01709	0.767258	1
Atg2a	-0.01712	0.64945	1
Atg13	-0.01713	0.972047	1
Siah2	-0.01721	0.85195	1
Pcgf2	-0.01722	0.170736	1
Pdxdc1	-0.01728	0.994816	1
Tor1b	-0.01735	0.435978	1
Pcnt	-0.01736	0.770346	1
Wac	-0.01742	0.447457	1
Ss18	-0.01757	0.306068	1
Atp5o	-0.01762	0.918013	1
Irf4	-0.01765	0.362108	1
Ppp2r3d	-0.0177	0.776662	1
Bsdc1	-0.01772	0.286826	1
Utp18	-0.01784	0.660923	1

Chn2	-0.01786	0.631874	1
Keap1	-0.01789	0.720721	1
Zfyve19	-0.01789	0.40544	1
Trap1	-0.01793	0.828042	1
Stx12	-0.01796	0.500484	1
Arid3b	-0.01801	0.783601	1
Stt3b	-0.01803	0.531126	1
Prnp	-0.01807	0.296977	1
Zbtb11os1	-0.01811	0.774141	1
Fancl	-0.01815	0.571376	1
Taf5	-0.0182	0.372439	1
Ostm1	-0.01823	0.778744	1
Zfp120	-0.01823	0.7515	1
Smurf2	-0.01824	0.754508	1
Utrn	-0.01824	0.926104	1
Tamm41	-0.01826	0.848184	1
Vps11	-0.0183	0.964029	1
Smg6	-0.0183	0.993389	1
Abl2	-0.01831	0.496997	1
Nupl2	-0.01836	0.68337	1
Tia1	-0.01837	0.229979	1
Tmem214	-0.01839	0.587582	1
Manf	-0.01842	0.880355	1
Nudc	-0.01865	0.641978	1
Vps16	-0.01873	0.209187	1
Zfp646	-0.01874	0.738569	1
Ncbp3	-0.01877	0.395742	1
Ndufb8	-0.01884	0.612316	1
Dus2	-0.01887	0.742713	1
Ddx10	-0.01887	0.497047	1
Ankfy1	-0.01888	0.40024	1
Clk4	-0.01895	0.465735	1
Cisd3	-0.01903	0.957239	1
Ate1	-0.01903	0.209465	1
Dnajb11	-0.01905	0.665711	1
Adck2	-0.01934	0.903301	1
Zfp715	-0.01935	0.82245	1
Pkn2	-0.01937	0.837551	1
Tm9sf1	-0.01937	0.890555	1
Rab29	-0.0194	0.383029	1
Prpsap1	-0.0194	0.610521	1
Fam207a	-0.01942	0.50038	1
Polr3d	-0.01944	0.535026	1
Naa20	-0.01955	0.889133	1
Man2a1	-0.01963	0.739661	1
Mrps2	-0.01965	0.655964	1
Xpo6	-0.01967	0.99741	1
Dhx30	-0.01967	0.586627	1

Tlnrd1	-0.01968	0.542302	1
Faap24	-0.01972	0.665194	1
Mroh1	-0.01973	0.706843	1
Slc35b4	-0.01978	0.935688	1
Abitram	-0.01979	0.442506	1
Stoml1	-0.0198	0.723781	1
Socs2	-0.0198	0.0553	1
Slc9a8	-0.01982	0.873544	1
Mrps23	-0.01984	0.775849	1
AI504432	-0.0199	0.572749	1
Ubqln4	-0.01998	0.853914	1
Ebna1bp2	-0.02	0.963531	1
Rnf111	-0.02003	0.574354	1
Cc2d1b	-0.02007	0.996852	1
2610001J05F	-0.0201	0.816687	1
Tmem242	-0.02014	0.640648	1
Abraxas1	-0.02014	0.640285	1
Zadh2	-0.02014	0.500379	1
G6pdx	-0.02019	0.61873	1
Ube2l3	-0.02023	0.983727	1
Frg1	-0.02024	0.951385	1
Cep97	-0.0203	0.432584	1
Zfp780b	-0.02032	0.412794	1
Thap11	-0.02034	0.992295	1
Prkx	-0.02037	0.452977	1
Ccdc43	-0.02038	0.469284	1
Tmem19	-0.0204	0.989504	1
Dennd1a	-0.02042	0.608652	1
Trim41	-0.02047	0.422072	1
Cep76	-0.02049	0.276202	1
Amz2	-0.02054	0.789963	1
Smim8	-0.02057	0.357622	1
Idh2	-0.02061	0.72727	1
Itgb1	-0.02064	0.750343	1
Gcc2	-0.02065	0.684357	1
Hgs	-0.02066	0.503131	1
Fig4	-0.02069	0.570329	1
Zfp160	-0.02071	0.267548	1
Sgpp1	-0.02071	0.476846	1
Slc12a9	-0.02074	0.865863	1
Apaf1	-0.02075	0.491862	1
Mettl3	-0.02078	0.580553	1
Lsm7	-0.02079	0.850832	1
Gabpb1	-0.02079	0.436155	1
Kmt2c	-0.0208	0.83183	1
Nhlrc3	-0.02082	0.590685	1
Pigq	-0.02082	0.425702	1
Fiz1	-0.02083	0.348766	1

Taf1d	-0.02083	0.899485	1
Slc17a5	-0.02095	0.418083	1
Akirin1	-0.02098	0.763101	1
Tpk1	-0.02101	0.80134	1
Cdk2ap2	-0.02101	0.983497	1
Dph5	-0.02107	0.732182	1
Bcl3	-0.02109	0.579059	1
Ptger4	-0.02111	0.240439	1
Zfp869	-0.02118	0.918842	1
Ciz1	-0.02127	0.725031	1
Brcc3	-0.02127	0.768087	1
Trim59	-0.02127	0.688557	1
Pelp1	-0.02128	0.369022	1
BC023719	-0.02142	0.908196	1
Rnf20	-0.02147	0.427036	1
Selenoi	-0.0215	0.320721	1
Ppp6r1	-0.02156	0.481724	1
Inpp1	-0.02165	0.857164	1
Clasrp	-0.02171	0.88998	1
Ric8b	-0.02172	0.472198	1
Spcs1	-0.02172	0.675528	1
N6amt1	-0.02173	0.421261	1
Ctsb	-0.02173	0.56502	1
Hspa4l	-0.02178	0.981408	1
Pitpnb	-0.02178	0.837526	1
1110012L19I	-0.0218	0.833098	1
Wdr33	-0.02181	0.748879	1
Axin1	-0.02197	0.21574	1
Lbh	-0.02202	0.400157	1
Trappc5	-0.02204	0.576904	1
Acox1	-0.02209	0.598784	1
Daglb	-0.02212	0.281957	1
Eif5a	-0.02215	0.989405	1
Mis12	-0.02222	0.858905	1
Snhg4	-0.02223	0.326849	1
Cep152	-0.02224	0.722615	1
Vti1b	-0.02225	0.553876	1
Fbrsl1	-0.0223	0.928768	1
Tspan3	-0.02231	0.973316	1
Ubr2	-0.02233	0.643645	1
Slc6a6	-0.02234	0.305616	1
Pola2	-0.02236	0.664919	1
Ulk3	-0.02244	0.975709	1
Ctr9	-0.0225	0.803436	1
Med6	-0.02254	0.504129	1
Twistnb	-0.02264	0.445052	1
Tmem184c	-0.02265	0.417938	1
Timm22	-0.02265	0.291665	1

Chd8	-0.02266	0.510876	1
2410004B18I	-0.02268	0.81804	1
Tcea1	-0.02268	0.673855	1
Arih1	-0.0227	0.951893	1
Usp38	-0.02273	0.729613	1
Mecp2	-0.02282	0.833741	1
Klf7	-0.02287	0.628243	1
Med20	-0.02291	0.247704	1
Washc5	-0.02292	0.505247	1
Zfand4	-0.02293	0.449027	1
Oip5os1	-0.023	0.767965	1
Mtrr	-0.023	0.892062	1
Rdh14	-0.02301	0.810512	1
Vsir	-0.02302	0.408134	1
Hdac4	-0.02303	0.890613	1
Ptk2b	-0.02304	0.955137	1
Matr3	-0.02308	0.913146	1
Nemf	-0.02315	0.590382	1
Gm17018	-0.02327	0.646867	1
Vps41	-0.0233	0.707204	1
Hdgfl2	-0.02334	0.703253	1
Cep83os	-0.02334	0.848792	1
Tmem127	-0.02338	0.624529	1
S1pr2	-0.02339	0.387873	1
Pdss1	-0.02339	0.271846	1
Phf2	-0.02345	0.769964	1
9430038I01F	-0.02345	0.141524	1
Fbh1	-0.02349	0.7058	1
Nudt19	-0.02361	0.772944	1
Zfp729b	-0.02363	0.351747	1
Mrps30	-0.02366	0.924618	1
Cep68	-0.02368	0.529104	1
Cad	-0.02371	0.640267	1
Lamtor1	-0.02377	0.74726	1
Arl13b	-0.02379	0.517333	1
Trpc4ap	-0.02379	0.550535	1
Mtch2	-0.02381	0.607196	1
Rpf2	-0.02381	0.793052	1
Nudt16l1	-0.02382	0.901007	1
Imp3	-0.02383	0.671954	1
Spred1	-0.02395	0.274414	1
Spata2	-0.02398	0.581416	1
Pigb	-0.024	0.765755	1
Mrpl20	-0.024	0.985673	1
Cndp2	-0.024	0.698157	1
2900076A07I	-0.02401	0.522885	1
Heca	-0.02403	0.804711	1
Vps4b	-0.02403	0.558744	1

Zfp362	-0.02407	0.804549	1
Gabbr1	-0.02407	0.940444	1
Nup54	-0.02412	0.727441	1
Mpp1	-0.02421	0.435608	1
Faap100	-0.02422	0.225801	1
Cetn3	-0.02424	0.328602	1
Cryl1	-0.02424	0.254956	1
Pim2	-0.02426	0.957506	1
Tprkb	-0.02429	0.318764	1
Setdb2	-0.02433	0.147174	1
Skp1a	-0.02435	0.974964	1
Elof1	-0.0244	0.515826	1
Specc1	-0.02445	0.212851	1
Gars	-0.0245	0.537589	1
Tbc1d20	-0.0245	0.643501	1
Jak2	-0.02451	0.905519	1
Rce1	-0.02456	0.740322	1
Emc3	-0.02457	0.87747	1
Syf2	-0.02461	0.92141	1
Kdm6b	-0.02463	0.458052	1
Mtmr2	-0.02464	0.467839	1
Tdrd3	-0.02468	0.264651	1
Churc1	-0.0247	0.419617	1
Pitpnc1	-0.02471	0.449358	1
Prdx1	-0.02478	0.61559	1
N4bp3	-0.02481	0.240578	1
Vps18	-0.02483	0.656237	1
Eif5	-0.02489	0.838923	1
Slc25a46	-0.02491	0.574171	1
Tspyl4	-0.02492	0.6181	1
Zfp52	-0.02506	0.667803	1
Cyp20a1	-0.02511	0.389255	1
Nom1	-0.02513	0.798208	1
Heatr3	-0.02513	0.655524	1
Prmt3	-0.02514	0.973053	1
Alg14	-0.02515	0.191798	1
Psmc2	-0.02519	0.460226	1
Ubr7	-0.0252	0.96911	1
Cyhr1	-0.02521	0.431542	1
Lgals8	-0.02522	0.977996	1
Nsf	-0.02523	0.296126	1
Csde1	-0.02536	0.872698	1
Cyp4v3	-0.02537	0.233512	1
Nagpa	-0.02538	0.750838	1
Erap1	-0.0254	0.944283	1
Lmbrd1	-0.02541	0.404972	1
Synj2bp	-0.02544	0.567017	1
Stx5a	-0.02546	0.787369	1

R3hcc1l	-0.02553	0.682027	1
Ubxn2b	-0.02555	0.339823	1
Zfp770	-0.02558	0.217251	1
C2cd2l	-0.02561	0.665	1
Pdcd7	-0.02569	0.34441	1
Samd8	-0.02569	0.521513	1
Arhgef7	-0.02575	0.675221	1
Gpr107	-0.02578	0.962249	1
Ccdc6	-0.02581	0.747159	1
Cdan1	-0.02585	0.08859	1
Fhod1	-0.02592	0.732389	1
Tcrg-C2	-0.02592	0.984497	1
Cope	-0.02595	0.855794	1
Ppp4r2	-0.02597	0.559737	1
Tbc1d13	-0.02602	0.781933	1
Fancf	-0.0261	0.452201	1
Pdcd10	-0.02612	0.684593	1
Rpa3	-0.02612	0.717887	1
Rad50	-0.02614	0.931839	1
Ncoa5	-0.02615	0.760777	1
Acox3	-0.02616	0.470619	1
Pi4kb	-0.02617	0.829958	1
Glmp	-0.02618	0.774126	1
Dhrs7b	-0.0262	0.733811	1
Lactb2	-0.0262	0.230471	1
Psma1	-0.02623	0.951646	1
Limd1	-0.02627	0.374705	1
Cisd1	-0.02629	0.702252	1
Hivep3	-0.02632	0.404688	1
Sars2	-0.02634	0.886557	1
Trim28	-0.0264	0.726869	1
Cpsf6	-0.0264	0.530368	1
Prkag2	-0.02647	0.380264	1
Ubqln2	-0.0265	0.887444	1
Zfand2b	-0.02654	0.333102	1
Mtr	-0.02654	0.608996	1
Prpf8	-0.02656	0.836074	1
Dhrs11	-0.02663	0.462686	1
Cog5	-0.02666	0.33639	1
Wdr43	-0.02671	0.995783	1
Ube4b	-0.02681	0.615505	1
Gm7072	-0.02683	0.9206	1
Rpusd4	-0.0269	0.447166	1
Ppp1r12b	-0.02691	0.928047	1
Snapin	-0.02694	0.850911	1
Triobp	-0.02698	0.74332	1
Cetn2	-0.02708	0.489306	1
D630008O14	-0.0271	0.441082	1

Cipc	-0.02716	0.516002	1
Abhd10	-0.02718	0.589084	1
Tmem11	-0.0272	0.654191	1
Zfp865	-0.02722	0.933358	1
Asrgl1	-0.02725	0.329592	1
Reps1	-0.02727	0.646857	1
Ube2b	-0.0273	0.762923	1
Mre11a	-0.02734	0.513617	1
Hipk2	-0.02738	0.730734	1
AU040320	-0.02743	0.773943	1
Ctc1	-0.02743	0.571235	1
Ugp2	-0.02747	0.233575	1
Mettl7a1	-0.0275	0.623104	1
Ccdc66	-0.02751	0.651311	1
Abtb1	-0.0276	0.907363	1
Ess2	-0.02762	0.908114	1
Seh1l	-0.02763	0.577646	1
Naa25	-0.02765	0.35818	1
Inpp5e	-0.02768	0.377536	1
Rbmx2	-0.0277	0.326479	1
Creld2	-0.02777	0.785362	1
Ndufb4	-0.02786	0.789733	1
Ppp2r5d	-0.02787	0.295934	1
Atxn2l	-0.02788	0.941856	1
Cdkl3	-0.02789	0.616174	1
Btla	-0.02792	0.111336	1
Fosl2	-0.02799	0.601904	1
Rfxank	-0.02802	0.645635	1
Traf3ip3	-0.02806	0.635559	1
Armh3	-0.02818	0.364475	1
Lsm12	-0.02822	0.827712	1
Trib1	-0.02831	0.720036	1
Dis3l	-0.02833	0.560317	1
Enpp1	-0.02835	0.412415	1
Tmed10	-0.02838	0.0664	1
Glt8d1	-0.02843	0.240531	1
Actr1b	-0.02844	0.814353	1
Cebpg	-0.02847	0.474711	1
Gm14305	-0.02849	0.264341	1
Znrd1	-0.02852	0.647831	1
Hacd2	-0.02854	0.708652	1
Aldh9a1	-0.02854	0.88147	1
Sugp2	-0.02855	0.935228	1
Sec61b	-0.02856	0.449654	1
Lrrc4	-0.02858	0.08659	1
Thoc5	-0.02862	0.474334	1
Epc1	-0.02865	0.996954	1
0610010K14	-0.0287	0.943593	1

Zmym3	-0.02873	0.522565	1
Atox1	-0.02882	0.740354	1
Klhdc10	-0.02885	0.256322	1
Syce2	-0.02887	0.269831	1
Chmp7	-0.02889	0.181083	1
Tapt1	-0.02891	0.397898	1
Myl12b	-0.02894	0.776253	1
Cryzl1	-0.02895	0.860761	1
Ubxn11	-0.02902	0.995796	1
Wdr18	-0.02903	0.57739	1
Tmem129	-0.02905	0.457455	1
Lpp	-0.02914	0.709613	1
Chfr	-0.02917	0.629817	1
Atpaf2	-0.02921	0.201771	1
Fbxo3	-0.02924	0.283014	1
Mapk1ip1l	-0.02925	0.911484	1
Helb	-0.02927	0.39252	1
Ppp2r5a	-0.02931	0.779101	1
Flvcr1	-0.02936	0.211555	1
Zfp131	-0.02936	0.756742	1
Insig2	-0.02937	0.641117	1
Luc7l	-0.02941	0.594716	1
Scyl2	-0.02941	0.438778	1
Ttf1	-0.02942	0.255421	1
Crkl	-0.02943	0.989274	1
Tomm34	-0.02948	0.28754	1
Pip5k1a	-0.02953	0.732175	1
H2-K1	-0.02969	0.788213	1
Higd1a	-0.02971	0.751666	1
Banp	-0.02981	0.913404	1
Pop5	-0.0299	0.952901	1
Mcmbp	-0.02995	0.501216	1
Map4k2	-0.03	0.954414	1
Mapk6	-0.03002	0.183866	1
Pitpnm2	-0.03003	0.745967	1
Pced1a	-0.03022	0.173518	1
Tm2d3	-0.03023	0.342157	1
Gprasp1	-0.03024	0.464101	1
Cmtm3	-0.03026	0.315296	1
Trim5	-0.03027	0.094012	1
Ints9	-0.03029	0.706268	1
Eral1	-0.0303	0.66647	1
Chtop	-0.03037	0.302912	1
Sprtn	-0.03038	0.328999	1
Ppp6r2	-0.0304	0.180389	1
Nprl2	-0.03041	0.282507	1
Ampd2	-0.03043	0.596098	1
Mrps36	-0.03052	0.417676	1

Dhps	-0.03062	0.524146	1
Sdhaf2	-0.03075	0.531339	1
Ufsp2	-0.03076	0.923206	1
Gpr108	-0.03081	0.617668	1
Mcm10	-0.03092	0.033073	1
Trabd	-0.03093	0.596012	1
Zfp398	-0.03104	0.326592	1
Ccdc117	-0.03108	0.545966	1
Hs2st1	-0.03111	0.90392	1
Psmd10	-0.0312	0.566511	1
Gle1	-0.03126	0.606149	1
Ppp1r11	-0.03126	0.821629	1
Spryd3	-0.03127	0.916948	1
Usp5	-0.03127	0.622566	1
Hus1	-0.03137	0.474251	1
Alg1	-0.03141	0.968652	1
Zbtb25	-0.03145	0.746262	1
Rufy1	-0.03147	0.921178	1
Entpd6	-0.0315	0.976961	1
Slc25a40	-0.03155	0.835487	1
Zscan29	-0.03178	0.577127	1
Snrpb	-0.03178	0.591917	1
Derl1	-0.03179	0.842581	1
Gm20324	-0.03182	0.400928	1
Abhd16a	-0.03185	0.93465	1
Glb1	-0.03187	0.461384	1
Smarcad1	-0.03198	0.362357	1
Snrpd2	-0.03206	0.779264	1
Coro1b	-0.03213	0.692626	1
Prr12	-0.03216	0.895092	1
Abhd17b	-0.03221	0.273353	1
Mtdh	-0.03222	0.974545	1
Tmem156	-0.03224	0.267302	1
Tbl1x	-0.03225	0.287899	1
Taok2	-0.03231	0.312164	1
Trappc12	-0.03231	0.575487	1
R3hdm2	-0.03237	0.486408	1
Usp16	-0.03239	0.523352	1
Fut7	-0.03248	0.282541	1
Fez2	-0.03259	0.375914	1
Impad1	-0.03268	0.278041	1
Zfc3h1	-0.03269	0.541977	1
Slc35a5	-0.03271	0.228265	1
Sass6	-0.03275	0.363031	1
Dnmt3b	-0.0328	0.243978	1
Mark3	-0.03282	0.465172	1
Foxk2	-0.03283	0.352473	1
D030056L22I	-0.03285	0.950232	1

Gatad1	-0.03287	0.303726	1
Heatr5b	-0.03292	0.292569	1
Atp6ap1	-0.03296	0.30862	1
Cacul1	-0.03297	0.821646	1
Lysmd3	-0.03298	0.144986	1
Rsl24d1	-0.03314	0.296143	1
Fhl2	-0.03315	0.262037	1
Adap1	-0.03315	0.646838	1
Lin7c	-0.03319	0.753149	1
Uck1	-0.03331	0.641127	1
Ptgr2	-0.03332	0.568342	1
Abca2	-0.03333	0.469508	1
Aste1	-0.03333	0.208903	1
Ccdc93	-0.03335	0.569321	1
4930522L14I	-0.03336	0.293824	1
Slc26a2	-0.03339	0.908227	1
Smap2	-0.03341	0.554107	1
Atp23	-0.03346	0.445618	1
Cops4	-0.03352	0.619844	1
Crcp	-0.03352	0.410655	1
Nsmf	-0.03352	0.30187	1
Ank	-0.03355	0.392854	1
Wbp1l	-0.03357	0.785288	1
Shoc2	-0.03359	0.055526	1
Abcf1	-0.03361	0.395843	1
Lats2	-0.03361	0.041926	1
Cop1	-0.03361	0.35083	1
St7	-0.03366	0.768287	1
Rab8b	-0.03379	0.934581	1
Fos	-0.03385	0.566537	1
5031425E22I	-0.03388	0.501914	1
Slc4a1ap	-0.0339	0.250138	1
Rnf170	-0.03393	0.078971	1
Fam126b	-0.03398	0.53183	1
Tra2a	-0.034	0.488201	1
Mrpl41	-0.03403	0.495057	1
Zfp330	-0.03408	0.981109	1
Mthfd1	-0.03409	0.352881	1
Rabep2	-0.03416	0.894745	1
Dffa	-0.03418	0.059557	1
Mybl2	-0.03419	0.265767	1
Pcsk7	-0.03433	0.44268	1
B4galt1	-0.03436	0.186167	1
Prr3	-0.03436	0.946726	1
Cnot6l	-0.03444	0.569597	1
Dnajc13	-0.03455	0.511424	1
Sp3os	-0.03459	0.449189	1
Zfp930	-0.03462	0.207188	1

Gm10076	-0.03462	0.49284	1
Tmsb15b1	-0.03463	0.594875	1
Scd2	-0.03465	0.34551	1
Ass1	-0.03466	0.772993	1
Rprd1a	-0.0347	0.867781	1
Sirt2	-0.03477	0.999506	1
Nipal3	-0.03484	0.425132	1
Tkt	-0.0349	0.519134	1
Paf1	-0.0349	0.309992	1
Csnk1e	-0.03496	0.325848	1
Blzf1	-0.03509	0.882445	1
Slc25a1	-0.03509	0.245989	1
Sos1	-0.03512	0.527518	1
Swi5	-0.03513	0.587575	1
Ppip5k2	-0.03515	0.982984	1
Wdtc1	-0.03518	0.882973	1
Mob4	-0.03524	0.28995	1
Gsap	-0.03525	0.766508	1
Ppp1r16a	-0.0353	0.708752	1
Zfp148	-0.03532	0.444168	1
Ube2d1	-0.03537	0.44769	1
Atp6v1e1	-0.03539	0.661501	1
4930581F22I	-0.03539	0.450949	1
Map1lc3b	-0.03539	0.692538	1
Zfp26	-0.03541	0.219733	1
Rsrp1	-0.03558	0.692616	1
Mgat1	-0.03559	0.551636	1
Gm10282	-0.03562	0.207275	1
Msl1	-0.03565	0.498928	1
Rab1b	-0.03567	0.426352	1
Hsf1	-0.03571	0.880534	1
Dnpep	-0.03573	0.692437	1
Tmem168	-0.03574	0.044979	1
Rps6ka3	-0.03591	0.989079	1
Nek9	-0.03597	0.162697	1
Srebf1	-0.03598	0.993752	1
Ppp4r3a	-0.03602	0.530088	1
Uxt	-0.03603	0.332592	1
Abl1	-0.03603	0.674502	1
Stx4a	-0.03605	0.168013	1
Sephs1	-0.03606	0.272723	1
Desi1	-0.03607	0.959426	1
Zfp319	-0.03608	0.616637	1
Dnaja3	-0.03609	0.40663	1
Tubg1	-0.03612	0.407346	1
Nemp2	-0.03614	0.283244	1
5033430I15F	-0.03615	0.974256	1
Dcaf15	-0.03617	0.166325	1

Acbd5	-0.03626	0.39972	1
Rnps1	-0.03627	0.660325	1
Tbce	-0.03627	0.426463	1
Cog3	-0.0363	0.548595	1
B3glct	-0.03641	0.46142	1
Cyfip1	-0.03642	0.887741	1
Cdc5l	-0.03652	0.332974	1
Abt1	-0.03653	0.628842	1
Prkra	-0.03655	0.472668	1
Dtnbp1	-0.03657	0.990098	1
1700097N02	-0.03665	0.17964	1
Fbxw5	-0.0367	0.986044	1
Pdcd4	-0.0367	0.265597	1
Pcmt1	-0.03673	0.328054	1
Cacybp	-0.03676	0.706207	1
Qsox1	-0.0369	0.509863	1
Cct4	-0.03696	0.619826	1
Ciao2a	-0.03698	0.868744	1
Zfp280d	-0.03699	0.792428	1
Ufl1	-0.037	0.746364	1
Zfp395	-0.03701	0.150542	1
Trp53rka	-0.03701	0.182348	1
Slmap	-0.03703	0.623887	1
Rmi1	-0.03706	0.323821	1
Pdpr	-0.03708	0.917891	1
Lage3	-0.03715	0.16644	1
Ilf2	-0.0372	0.952485	1
Thap12	-0.03726	0.410606	1
Cdyl	-0.03727	0.375694	1
Polk	-0.03732	0.495888	1
Cwc27	-0.03739	0.46111	1
Tsen15	-0.03747	0.335054	1
Nup188	-0.0375	0.150908	1
Cenps	-0.03751	0.20597	1
Ppp1r7	-0.03755	0.324667	1
Srp19	-0.03766	0.684908	1
Acadm	-0.03773	0.788012	1
Tgs1	-0.0378	0.342338	1
Ivns1abp	-0.0378	0.836247	1
Atpsckmt	-0.03785	0.649851	1
Peak1	-0.03798	0.445912	1
Snx1	-0.03799	0.795752	1
Cep19	-0.03803	0.043194	1
Sf3a1	-0.03806	0.60352	1
Diaph1	-0.03806	0.693409	1
Vrk2	-0.0381	0.858055	1
Nck2	-0.03811	0.860087	1
Slc31a2	-0.03819	0.12408	1

Dpy19l1	-0.0382	0.81801	1
Nat10	-0.03825	0.852502	1
Tnk2	-0.03827	0.458582	1
Trp53bp2	-0.03834	0.387417	1
Psmg3	-0.03836	0.86048	1
Arid4a	-0.03843	0.377747	1
Dhx34	-0.03844	0.282449	1
Tbc1d10b	-0.03844	0.362687	1
Tusc3	-0.03846	0.234184	1
Htatsf1	-0.03856	0.958557	1
Carmil2	-0.03861	0.90213	1
Ing4	-0.03867	0.653724	1
Dis3	-0.03872	0.509143	1
Sirt7	-0.03888	0.474505	1
Dcps	-0.03888	0.445694	1
Cdc34	-0.039	0.641013	1
Polr3g	-0.03904	0.331526	1
Ccdc12	-0.03904	0.449322	1
Dync1li2	-0.03905	0.561789	1
Kif1b	-0.03905	0.143139	1
Fundc2	-0.0391	0.721984	1
Med8	-0.03915	0.452722	1
Gm2000	-0.03916	0.827596	1
Cenpj	-0.03917	0.130343	1
Jagn1	-0.0392	0.265215	1
Scp2	-0.0392	0.788905	1
Klhl5	-0.03922	0.123111	1
Mtch1	-0.03923	0.792176	1
Dr1	-0.03924	0.668659	1
Lair1	-0.03925	0.123379	1
Tbrg4	-0.03926	0.804029	1
Iars2	-0.03927	0.466686	1
Tmem167	-0.03942	0.724022	1
Mllt10	-0.03947	0.943834	1
Larp4	-0.03952	0.912782	1
Aamdc	-0.03956	0.223673	1
Cab39	-0.03956	0.549914	1
Traf7	-0.03961	0.379554	1
Nbeal2	-0.03965	0.215616	1
Trim25	-0.03966	0.798911	1
Actl6a	-0.0398	0.426139	1
Pef1	-0.03981	0.117681	1
Thyn1	-0.03984	0.370408	1
Paics	-0.03992	0.868192	1
Msrb2	-0.04001	0.335618	1
Fam168b	-0.04003	0.431937	1
Ift140	-0.04009	0.599572	1
Nudt4	-0.04009	0.838418	1

Gtf2h3	-0.04022	0.12632	1
B9d2	-0.04026	0.702633	1
Gimap1os	-0.04033	0.596682	1
Ccdc166	-0.04035	0.530941	1
Elp1	-0.04036	0.915641	1
Agtpbp1	-0.04038	0.46971	1
Pvrig	-0.04044	0.417209	1
Prkar2a	-0.04048	0.624275	1
Mus81	-0.0405	0.33069	1
Gmpr2	-0.04053	0.691126	1
Glod4	-0.04054	0.762803	1
Tmco4	-0.04057	0.264319	1
Smn1	-0.04059	0.904783	1
Phka2	-0.04061	0.889978	1
Zkscan3	-0.04073	0.882197	1
Ints8	-0.04074	0.240432	1
Haus2	-0.04081	0.209962	1
Tmem60	-0.04083	0.435716	1
Bcl9l	-0.04088	0.374483	1
Ptcd1	-0.04089	0.190136	1
Slc36a3os	-0.04097	0.074682	1
Tbc1d5	-0.04097	0.971222	1
Snrnp27	-0.04104	0.766506	1
Smim20	-0.04107	0.5693	1
Fam3c	-0.0411	0.560094	1
Slc1a5	-0.04111	0.713932	1
Fus	-0.0412	0.766724	1
Cnpy2	-0.0412	0.571418	1
Ttll5	-0.04134	0.341969	1
Gmppb	-0.04139	0.272071	1
Zfp422	-0.04142	0.206188	1
Slc37a1	-0.04158	0.311404	1
Tars2	-0.04161	0.588252	1
Agtrap	-0.04165	0.869174	1
Ercc3	-0.04169	0.160108	1
Tom1l2	-0.0417	0.632862	1
Scmh1	-0.04182	0.394426	1
Dlg2	-0.04186	0.533902	1
Abcg1	-0.04187	0.837424	1
Pgd	-0.04191	0.831695	1
Ube2e3	-0.04194	0.151822	1
Smpd1	-0.04197	0.977071	1
Snrpf	-0.042	0.61415	1
Ppp4r3b	-0.042	0.288645	1
Bcl2	-0.04202	0.851312	1
Dbt	-0.04207	0.915839	1
Rfx5	-0.04207	0.30151	1
Galnt11	-0.04208	0.882066	1

Ankrd17	-0.04212	0.38037	1
Adprh	-0.04225	0.743106	1
Mrpl50	-0.04226	0.99556	1
Qtrt1	-0.0423	0.484948	1
Coq10a	-0.04232	0.025557	1
Ecsit	-0.04234	0.611779	1
Sec24d	-0.04234	0.922438	1
Sgta	-0.04248	0.52772	1
Ndufs8	-0.04251	0.632398	1
Primpol	-0.04254	0.260393	1
Agpat4	-0.04256	0.533177	1
Dnm1l	-0.04279	0.824077	1
Gm48678	-0.04293	0.064518	1
Gmcl1	-0.04294	0.432796	1
Piezo1	-0.04298	0.638853	1
Fuz	-0.043	0.10111	1
2610020C07I	-0.04305	0.094687	1
Ssr3	-0.04306	0.506417	1
Ap4b1	-0.0431	0.440651	1
Ncoa6	-0.0431	0.321845	1
Yipf3	-0.04312	0.934331	1
Ciao1	-0.04314	0.44917	1
Appl2	-0.04319	0.352127	1
Nxf1	-0.04321	0.386736	1
4921524J17I	-0.04321	0.9281	1
Mvd	-0.04327	0.131306	1
Snx6	-0.04333	0.914056	1
Tsen54	-0.0434	0.049912	1
Sf3b5	-0.04354	0.333012	1
Ube2v2	-0.04358	0.928153	1
Nadk	-0.04363	0.802966	1
Eif1	-0.04363	0.521339	1
Ap5m1	-0.04364	0.666796	1
Uqcrb	-0.04365	0.702152	1
Srsf9	-0.04366	0.032207	1
Prps1l3	-0.04371	0.259933	1
Lsm8	-0.04377	0.49717	1
Wrap53	-0.04387	0.242902	1
Ptger2	-0.04389	0.650616	1
Pik3cd	-0.04392	0.829074	1
Slc25a53	-0.04393	0.273649	1
Hps6	-0.04396	0.103627	1
Zmat3	-0.04399	0.709416	1
Szt2	-0.04413	0.075084	1
Vcp	-0.04414	0.673462	1
Sec61a1	-0.04425	0.618767	1
Zfp106	-0.0444	0.848619	1
Dhx37	-0.04446	0.281342	1

Psmd3	-0.04447	0.280524	1
Mdc1	-0.04449	0.660037	1
Sfr1	-0.0445	0.478824	1
Zmat5	-0.04451	0.559556	1
Parp2	-0.04453	0.60291	1
Gm11802	-0.04455	0.010613	1
Abhd8	-0.04458	0.925544	1
Acaa1a	-0.04462	0.347198	1
Nampt	-0.04471	0.634448	1
Natd1	-0.04473	0.174771	1
Cab39l	-0.04476	0.221771	1
Nup160	-0.04481	0.607668	1
Lmtk2	-0.04485	0.804067	1
Ephx1	-0.04486	0.798603	1
Faah	-0.04488	0.507003	1
Poc1b	-0.04501	0.210258	1
Vps26c	-0.04503	0.688534	1
Mrpl33	-0.04509	0.482471	1
Asb6	-0.04513	0.23637	1
Pear1	-0.04522	0.50253	1
Zfp386	-0.04524	0.806407	1
E330020D12I	-0.04524	0.59957	1
Fbxl12	-0.04526	0.641068	1
Ammecr1l	-0.04527	0.140657	1
Zbtb7a	-0.04528	0.350591	1
Chpf2	-0.04529	0.166799	1
Cln5	-0.04531	0.933606	1
Gtf2h4	-0.04533	0.129058	1
Crtap	-0.04541	0.339767	1
Sac3d1	-0.04543	0.653574	1
Gm17173	-0.04547	0.768792	1
Sh3glb2	-0.04549	0.301062	1
G3bp1	-0.04549	0.654951	1
Galnt1	-0.04551	0.383702	1
Ppib	-0.04552	0.841046	1
Exoc8	-0.04554	0.270561	1
Tmem18	-0.04556	0.045637	1
Ube2f	-0.04562	0.143883	1
Pcyt1a	-0.04562	0.236996	1
Dlgap4	-0.04567	0.717303	1
Nbeal1	-0.04574	0.684959	1
Kdm5c	-0.04576	0.930026	1
Znrf3	-0.0458	0.778737	1
Aida	-0.04581	0.355173	1
Taf6l	-0.04586	0.180019	1
Casp1	-0.04591	0.486609	1
Fastk	-0.04592	0.683852	1
Smad5	-0.04594	0.07567	1

Pum2	-0.04599	0.149337	1
Dcaf1	-0.04604	0.314	1
Tpcn2	-0.04605	0.144159	1
Arl16	-0.0462	0.072275	1
Glcci1	-0.04621	0.388203	1
Hscb	-0.04628	0.408479	1
Slc9a6	-0.04631	0.589773	1
Nmd3	-0.04643	0.681366	1
Med23	-0.04645	0.318438	1
Senp1	-0.04646	0.356767	1
Ttc4	-0.04648	0.402168	1
Maea	-0.04652	0.753976	1
Wdr81	-0.04655	0.554812	1
Coro1c	-0.04668	0.793966	1
Paip2b	-0.04669	0.867878	1
Cutc	-0.0467	0.746341	1
Galns	-0.04672	0.698475	1
Taf4	-0.04674	0.314577	1
Tmem243	-0.04676	0.732509	1
Fbxl5	-0.04677	0.220808	1
Aftph	-0.04677	0.256476	1
Snx9	-0.04682	0.65542	1
Nup98	-0.04683	0.917371	1
Rab5b	-0.04688	0.445513	1
Comtd1	-0.04692	0.527626	1
Kat6a	-0.04698	0.526825	1
4833420G17	-0.047	0.921535	1
Eif2s3y	-0.04705	0.098685	1
Cog2	-0.04706	0.525958	1
Fastkd1	-0.04706	0.156852	1
Itpa	-0.04708	0.080512	1
Rsl1d1	-0.04714	0.737623	1
Ankrd12	-0.04715	0.721564	1
Aldh16a1	-0.04718	0.590379	1
Tmem62	-0.04731	0.800926	1
Nelfa	-0.04741	0.641949	1
Nipsnap3b	-0.04742	0.569248	1
Snrpa	-0.04744	0.996655	1
Ranbp3	-0.04745	0.18339	1
Pole3	-0.04745	0.63543	1
Extl3	-0.04749	0.56222	1
Eif4a1	-0.04755	0.588165	1
Anapc16	-0.04756	0.302142	1
Ptrh2	-0.04762	0.439273	1
Utp20	-0.04764	0.473141	1
Rell1	-0.04773	0.176844	1
Tsr3	-0.0478	0.404197	1
Zgpat	-0.04801	0.58988	1

Scaf8	-0.04804	0.571043	1
Kcnq1ot1	-0.04807	0.610196	1
Atp5e	-0.04811	0.445242	1
Zfp688	-0.04831	0.396369	1
Zdhhc2	-0.04835	0.15483	1
Ppp2r5e	-0.04837	0.503994	1
Tex30	-0.04839	0.32921	1
2210016F16l	-0.04844	0.182796	1
Dbr1	-0.04853	0.427705	1
Pxylp1	-0.04866	0.964882	1
Rad52	-0.04873	0.059907	1
Akap9	-0.04878	0.524924	1
Zfp91	-0.04881	0.436331	1
Wdr24	-0.04895	0.156431	1
Itpripl1	-0.04901	0.40386	1
Tmsb10	-0.04903	0.656591	1
Pex7	-0.04903	0.506541	1
Vhl	-0.04904	0.234696	1
Gpatch8	-0.04908	0.634557	1
Gcnt1	-0.04915	0.749963	1
Gnptab	-0.04916	0.409589	1
Ankrd16	-0.04918	0.250247	1
Dtx2	-0.0492	0.352533	1
Atrx	-0.04923	0.467655	1
Cdk5	-0.04928	0.34176	1
Maml1	-0.04933	0.146042	1
Cep85l	-0.04933	0.004104	1
Mettl9	-0.04937	0.924823	1
Trpv2	-0.04938	0.277029	1
Copz1	-0.04939	0.338497	1
Tars	-0.04942	0.481079	1
Ankrd39	-0.04945	0.192817	1
Lypla1	-0.04949	0.339061	1
Asah1	-0.04949	0.960697	1
Sfmbt1	-0.04951	0.284765	1
Champ1	-0.04953	0.566122	1
Gipc1	-0.04955	0.92304	1
Plekhf2	-0.04967	0.841311	1
Phykpl	-0.0497	0.240916	1
Zhx1	-0.04972	0.266791	1
Fam20b	-0.04975	0.124726	1
Pepd	-0.04975	0.290474	1
Usp33	-0.04977	0.113131	1
Faim	-0.04979	0.591176	1
Rab40c	-0.04979	0.254961	1
Zfp771	-0.0498	0.790673	1
Tmco6	-0.04981	0.453603	1
Thoc7	-0.04983	0.362409	1

Sphk2	-0.04985	0.680524	1
Nsmce2	-0.04989	0.151585	1
Wdr26	-0.04991	0.587922	1
Erf	-0.04992	0.145974	1
Ppp2ca	-0.05002	0.231988	1
Rsbn1l	-0.05003	0.376843	1
Spin1	-0.05007	0.397036	1
Elk4	-0.05007	0.942534	1
Csrnp1	-0.05019	0.79689	1
Fam185a	-0.05027	0.926528	1
Ndufa2	-0.05031	0.560672	1
March5	-0.05032	0.997311	1
Ehmt1	-0.05033	0.906738	1
Tnpo2	-0.05039	0.114891	1
Ipo13	-0.05046	0.237971	1
1110008P14	-0.05056	0.626259	1
Camkmt	-0.05057	0.663273	1
Bloc1s3	-0.05068	0.783228	1
Ahctf1	-0.05084	0.74207	1
Stxbp3	-0.05091	0.210405	1
Pin1	-0.05096	0.228675	1
Immp2l	-0.05097	0.541317	1
Fam8a1	-0.05112	0.099267	1
Gtdc1	-0.05115	0.862378	1
Bid	-0.0512	0.258052	1
Dohh	-0.05121	0.378558	1
Lnpep	-0.05121	0.529329	1
Arpc1b	-0.05121	0.293548	1
Trim36	-0.05123	0.253937	1
Zbtb38	-0.05127	0.553369	1
Btrc	-0.05141	0.533408	1
Pet100	-0.05152	0.357129	1
Bcl2l12	-0.05154	0.740977	1
Agbl5	-0.05163	0.006095	1
Emc9	-0.05166	0.401435	1
Vps33a	-0.05168	0.22406	1
Tnfaip8	-0.05174	0.686904	1
Arfgap2	-0.05184	0.504457	1
H2-T23	-0.05186	0.44538	1
Mnt	-0.05187	0.438708	1
Ddx1	-0.0519	0.623672	1
Scamp2	-0.05194	0.516821	1
Cept1	-0.05198	0.19046	1
Traf2	-0.05198	0.839179	1
Depdc5	-0.052	0.334432	1
Mrpl54	-0.05207	0.511235	1
Mocs1	-0.05215	0.849658	1
Zfp574	-0.05221	0.070799	1

Clpx	-0.05221	0.411711	1
Cnot6	-0.05225	0.728748	1
Rxrb	-0.05226	0.557157	1
Cul1	-0.0523	0.943277	1
Wscd2	-0.0523	0.279115	1
Isca1	-0.05236	0.408674	1
Elovl1	-0.05241	0.37777	1
Pign	-0.05245	0.14353	1
Mepce	-0.05246	0.286335	1
Slc20a1	-0.0525	0.459334	1
Traf5	-0.05254	0.832822	1
Tmco3	-0.05272	0.028959	1
Timm8b	-0.05283	0.404682	1
Zfp800	-0.05284	0.632188	1
Myef2	-0.05293	0.392834	1
Tiam1	-0.05296	0.415685	1
Mrpl35	-0.05301	0.981811	1
Mmut	-0.05305	0.178393	1
Gm45716	-0.0531	0.522553	1
Sgcb	-0.05312	0.303016	1
8030462N17	-0.05314	0.298433	1
Acot2	-0.05316	0.34128	1
Nudt14	-0.05325	0.248088	1
P4hb	-0.05327	0.855988	1
Abhd6	-0.05328	0.738803	1
Tbcc	-0.05331	0.079489	1
Slc38a10	-0.05335	0.44021	1
Lpar6	-0.05343	0.879503	1
Uba3	-0.05351	0.287776	1
Nipsnap2	-0.05352	0.362769	1
Fbxo6	-0.05352	0.042413	1
Sppl3	-0.05357	0.78227	1
Paip1	-0.05358	0.487371	1
Actr2	-0.05363	0.118853	1
Kdm3b	-0.05372	0.741728	1
Dym	-0.05374	0.332126	1
Vamp5	-0.05375	0.763056	1
Baz2b	-0.05384	0.159312	1
Lsm4	-0.05388	0.608157	1
Gtpbp6	-0.05389	0.675795	1
Ddb2	-0.05389	0.389651	1
Tspan2	-0.05391	0.054453	1
Gm33104	-0.05393	0.88467	1
Magoh	-0.05397	0.506746	1
BC002059	-0.05397	0.805078	1
Gigyf2	-0.05399	0.452638	1
Mier2	-0.05402	0.052188	1
Rcc2	-0.05403	0.801815	1

Prdm10	-0.05407	0.065212	1
Chst11	-0.0541	0.928527	1
Rint1	-0.05411	0.138178	1
Parn	-0.05412	0.267407	1
Upf1	-0.05415	0.196693	1
Ash1l	-0.05427	0.427258	1
Mrps35	-0.05428	0.212281	1
Fh1	-0.05438	0.463746	1
Tcf25	-0.05442	0.830056	1
Aspscr1	-0.05443	0.728324	1
Enpp4	-0.05443	0.347422	1
Pcnp	-0.05448	0.614878	1
Scml4	-0.05449	0.267316	1
Ncdn	-0.05455	0.039068	1
Snrpd3	-0.05455	0.817944	1
Ube2q1	-0.05457	0.360583	1
Rad54b	-0.05461	0.008882	1
Set	-0.05465	0.534674	1
Trnt1	-0.05467	0.692329	1
Ube3c	-0.05472	0.426916	1
Efcab14	-0.05479	0.110795	1
Prdm2	-0.05479	0.815801	1
Trip11	-0.05485	0.465089	1
Saal1	-0.05497	0.566524	1
Tmem263	-0.05499	0.097911	1
Med31	-0.05502	0.344284	1
Cog1	-0.05507	0.862359	1
Oaz1	-0.0551	0.27836	1
Aamp	-0.05512	0.384645	1
Stard3	-0.05513	0.656082	1
Kdm7a	-0.05515	0.509076	1
Srfbp1	-0.05516	0.373	1
Dcaf10	-0.0553	0.547408	1
Exosc8	-0.05532	0.51569	1
Ppm1m	-0.05536	0.660971	1
Zbtb22	-0.05538	0.007804	1
Ssr1	-0.05539	0.372057	1
Btbd9	-0.05541	0.116411	1
Sh2d3c	-0.05543	0.129084	1
Pex11b	-0.05544	0.807006	1
Ccdc71l	-0.05549	0.061387	1
Tubgcp3	-0.05551	0.37384	1
Casp8ap2	-0.05562	0.405005	1
Ggta1	-0.05562	0.069119	1
Ftsj1	-0.05567	0.335117	1
Ptcd2	-0.05572	0.897243	1
Rbm4	-0.05576	0.182726	1
Polg	-0.05582	0.039668	1

Rbm28	-0.05589	0.793352	1
Dstn	-0.05592	0.06947	1
Cklf	-0.05592	0.435184	1
Pggt1b	-0.05593	0.344076	1
Brox	-0.05593	0.483557	1
Gm16845	-0.05594	0.092174	1
Hist3h2ba	-0.05599	0.244403	1
Slc36a1	-0.05601	0.053712	1
Xrcc6	-0.05603	0.340771	1
Cdpf1	-0.05606	0.843787	1
Spata13	-0.05606	0.140499	1
Hbs1l	-0.05609	0.448163	1
Naa35	-0.05611	0.321799	1
Topbp1	-0.05627	0.068534	1
Azi2	-0.05628	0.794462	1
Tmem234	-0.05629	0.923241	1
Abhd13	-0.05633	0.055901	1
Zfp719	-0.05635	0.048088	1
Vkorc1l1	-0.05641	0.066115	1
Limk2	-0.05656	0.361563	1
Nrarp	-0.05657	0.010041	1
1700021F05I	-0.0566	0.253071	1
Rragc	-0.05664	0.28501	1
Mtx1	-0.0567	0.216942	1
Plscr3	-0.05672	0.224647	1
Bcl2l11	-0.05673	0.678413	1
Brf1	-0.05673	0.229676	1
Ctnnbl1	-0.05679	0.351843	1
Haus5	-0.05679	0.644535	1
Fbxo7	-0.05684	0.277931	1
Rrp15	-0.05684	0.412431	1
Rbm15b	-0.05687	0.131024	1
Ticam1	-0.05689	0.646882	1
Nmb	-0.0569	0.389915	1
Ripor1	-0.05692	0.271207	1
Myo9a	-0.05692	0.871276	1
Sdccag8	-0.05697	0.683949	1
Snf8	-0.057	0.321388	1
Mad2l1bp	-0.05702	0.266843	1
Zscan25	-0.05703	0.2922	1
Rassf7	-0.05704	0.118612	1
Atp5g3	-0.05706	0.707911	1
Katna1	-0.05706	0.462616	1
Parvg	-0.05706	0.774223	1
Tfe3	-0.05706	0.640223	1
Abraxas2	-0.05709	0.545861	1
Ccs	-0.05714	0.840356	1
Efl1	-0.05716	0.455764	1

Dyrk1a	-0.05717	0.59251	1
Dot1l	-0.05717	0.991665	1
Wwp2	-0.05718	0.851577	1
P2ry14	-0.0572	0.690362	1
Polr1a	-0.05723	0.766871	1
Golga3	-0.05724	0.164122	1
Mrpl47	-0.05731	0.23027	1
Trim23	-0.05743	0.036374	1
Tsga10	-0.05745	0.018119	1
Mycbp	-0.05746	0.014968	1
Ube2g1	-0.0575	0.738271	1
Ttf2	-0.05751	0.688163	1
Cenpo	-0.05755	0.310416	1
Scpep1	-0.05755	0.956995	1
Sytl3	-0.0576	0.498102	1
Ciapin1	-0.05761	0.397867	1
Togaram1	-0.05762	0.404557	1
Plin2	-0.05762	0.472845	1
Cmpk1	-0.05764	0.576122	1
Nup155	-0.05766	0.528328	1
Fancm	-0.05771	0.058054	1
Rhot2	-0.05775	0.346654	1
Mtbp	-0.05776	0.369988	1
Rrp8	-0.05778	0.522514	1
Acap1	-0.05787	0.852424	1
Rmdn3	-0.05791	0.08914	1
Syngr2	-0.05793	0.716974	1
Ddx50	-0.05793	0.175905	1
Tubgcp4	-0.05797	0.612462	1
9930111J21I	-0.05797	0.652679	1
Xpa	-0.05805	0.816987	1
Mrpl49	-0.0581	0.254123	1
Cdipt	-0.0581	0.418767	1
E130307A14I	-0.05812	0.181888	1
Galnt3	-0.05813	0.350636	1
Ddx54	-0.05816	0.729836	1
Rab14	-0.05817	0.21373	1
Vgll4	-0.05817	0.427954	1
Stk11ip	-0.05818	0.620981	1
Zfp827	-0.05819	0.213694	1
Cemip2	-0.05819	0.555796	1
Rttn	-0.05823	0.046839	1
Gale	-0.05825	0.007938	1
Elk1	-0.05825	0.150529	1
Cbll1	-0.05826	0.318965	1
Nop56	-0.0583	0.299661	1
Prdx3	-0.05832	0.691017	1
BC052040	-0.05834	0.283614	1

Endov	-0.05836	0.336915	1
Copb1	-0.05839	0.264364	1
Washc2	-0.05848	0.315535	1
Ldb1	-0.05849	0.268176	1
Pot1b	-0.0586	0.175161	1
Med1	-0.05861	0.46018	1
6720427I07F	-0.05862	0.254546	1
Pygb	-0.05863	0.446402	1
Ino80d	-0.05868	0.977356	1
Skp2	-0.05874	0.009485	1
Zscan21	-0.05878	0.581761	1
Urgcp	-0.0588	0.198098	1
Hyou1	-0.05887	0.237846	1
Arnt	-0.05887	0.121613	1
Nsfl1c	-0.05888	0.715912	1
Clec2i	-0.05903	0.502855	1
Gins1	-0.05916	0.016008	1
Ppp2r3c	-0.0593	0.402369	1
Gng10	-0.05934	0.906144	1
Vps4a	-0.05935	0.34539	1
Etaa1	-0.05938	0.221268	1
Slc4a8	-0.05941	0.020541	1
Hmg20a	-0.05947	0.583933	1
Anp32b	-0.05949	0.908889	1
Cep78	-0.05955	0.063081	1
Rragb	-0.05956	0.748396	1
Sharpin	-0.05967	0.838155	1
Pglyrp2	-0.05973	0.388346	1
Setd5	-0.05975	0.654143	1
G2e3	-0.05977	0.012985	1
Ints5	-0.05978	0.359223	1
Cybc1	-0.05982	0.827095	1
Rbm26	-0.05983	0.361212	1
Ormdl1	-0.05987	0.19872	1
Rab3d	-0.05992	0.815152	1
Pibf1	-0.05994	0.502109	1
D130040H23	-0.05994	0.183709	1
Med7	-0.05997	0.107163	1
Hnrnph1	-0.06001	0.851328	1
Arhgap15	-0.06002	0.418948	1
Hmbox1	-0.06005	0.121172	1
1700037H04	-0.0601	0.122788	1
Rfc3	-0.06013	0.238181	1
Rbm48	-0.06016	0.225125	1
Cd80	-0.0602	0.69733	1
Ruvbl1	-0.06025	0.224989	1
Pnpla2	-0.06031	0.194033	1
Rap1gds1	-0.06037	0.73759	1

Ube2k	-0.06041	0.49541	1
Sh3bgrl	-0.06043	0.46472	1
Zfp24	-0.06046	0.146756	1
Mknk1	-0.06047	0.049006	1
Wdr48	-0.06051	0.286203	1
Mfap3	-0.06052	0.090178	1
Oga	-0.06057	0.302968	1
Pbx4	-0.0606	0.105226	1
Dnaja2	-0.06064	0.477198	1
Hist1h4c	-0.0607	0.133458	1
B4galt3	-0.06071	0.066061	1
Sde2	-0.06078	0.224857	1
Psmb2	-0.06085	0.810032	1
Tmem199	-0.0609	0.114816	1
Arl3	-0.06096	0.266192	1
Uqcrc1	-0.06097	0.41945	1
Kin	-0.06102	0.31225	1
Pgm2l1	-0.06103	0.040434	1
Pold2	-0.06108	0.305226	1
Orc2	-0.06108	0.563156	1
Gm37494	-0.06111	0.457324	1
Mlx	-0.06113	0.130324	1
Ints13	-0.06116	0.386234	1
Ranbp2	-0.06119	0.764179	1
Hps4	-0.06122	0.344977	1
Ccnd3	-0.06124	0.508566	1
Cdkn1a	-0.06125	0.44279	1
Uty	-0.0614	0.542664	1
Cherp	-0.06143	0.279841	1
Gm44175	-0.06153	0.370408	1
Ipo5	-0.06153	0.663291	1
Wtap	-0.06154	0.759356	1
Wee1	-0.06157	0.334451	1
Cd2bp2	-0.0616	0.367871	1
Disc1	-0.06161	0.00338	1
Nelfb	-0.06172	0.571656	1
Cct5	-0.06181	0.344353	1
Cdc26	-0.06183	0.345366	1
Eml4	-0.06185	0.917886	1
Kdm4a	-0.06188	0.437277	1
Bap1	-0.06191	0.293803	1
Ccdc50	-0.06191	0.187082	1
Adsl	-0.06196	0.926624	1
Psmb1	-0.06201	0.479493	1
Tango2	-0.06223	0.098172	1
Stard3nl	-0.06224	0.527834	1
Cd2ap	-0.0623	0.591799	1
Wls	-0.06242	0.228901	1

Ptms	-0.06244	0.583834	1
Brat1	-0.06253	0.985805	1
Gemin5	-0.06257	0.47801	1
Gtf3c4	-0.06259	0.003768	1
Ubxn6	-0.06273	0.904321	1
Cd151	-0.06273	0.308775	1
Rock1	-0.06276	0.268424	1
Ppp3r1	-0.0628	0.726197	1
Zfp597	-0.06284	0.414963	1
Unc45a	-0.06309	0.129653	1
Als2cl	-0.06311	0.010776	1
Rad9a	-0.06319	0.210636	1
Glipr1	-0.06321	0.309474	1
Fam133b	-0.06321	0.422733	1
Tial1	-0.06325	0.985775	1
Nsun2	-0.06326	0.549876	1
Leo1	-0.06327	0.243651	1
Trappc2	-0.06343	0.439747	1
Zfp62	-0.06345	0.202189	1
Naip2	-0.06357	0.098284	1
Orai3	-0.06358	0.723121	1
Zwint	-0.06359	0.864044	1
Ctps	-0.06361	0.723337	1
Fancg	-0.06369	0.029056	1
Hars	-0.06374	0.271768	1
Btbd6	-0.0638	0.161348	1
Ccdc32	-0.06398	0.250445	1
Zbtb11	-0.06399	0.774107	1
Slc38a9	-0.06401	0.914898	1
Fanca	-0.06404	0.014435	1
Tbk1	-0.06405	0.445582	1
Pwwp3a	-0.06411	0.08293	1
Fam120a	-0.06413	0.421947	1
Enkd1	-0.0643	0.003494	1
Tdg	-0.06432	0.229477	1
Mrpl53	-0.06434	0.087037	1
Numb	-0.06438	0.92073	1
Zfp868	-0.06444	0.587437	1
Txnl4a	-0.06458	0.190765	1
Zfyve27	-0.06459	0.491074	1
Plekha5	-0.06467	0.143713	1
Cspp1	-0.06472	0.466219	1
Plaa	-0.06476	0.323166	1
Tmem29	-0.06481	0.680712	1
Supt6	-0.06481	0.845731	1
Asxl2	-0.06485	0.352181	1
Slc12a7	-0.06493	0.109682	1
Ago3	-0.06506	0.723069	1

Cd68	-0.0651	0.112234	1
A930005H10	-0.06528	0.082428	1
Gnpat	-0.06529	0.486903	1
Sec11c	-0.06534	0.233744	1
Tut7	-0.06535	0.559918	1
Gm15232	-0.06537	0.13082	1
Rassf2	-0.06539	0.384892	1
Papola	-0.06539	0.547313	1
Slc30a7	-0.0654	0.559266	1
Atg101	-0.06549	0.229064	1
Arid2	-0.06551	0.094335	1
Myo9b	-0.06558	0.270168	1
Lin54	-0.06559	0.466588	1
Bloc1s1	-0.0656	0.737823	1
Psen1	-0.06563	0.21521	1
Zfp207	-0.06563	0.341425	1
Bloc1s4	-0.06564	0.43109	1
Eif4h	-0.06567	0.208192	1
Zdhhc4	-0.06576	0.886266	1
Klhl2	-0.06581	0.023553	1
Supt5	-0.06592	0.176237	1
Rfc5	-0.06595	0.077615	1
Iffo1	-0.066	0.661572	1
Cep170	-0.06601	0.179034	1
Cct6a	-0.06607	0.410021	1
Zcchc10	-0.06607	0.440622	1
BE692007	-0.06617	0.945811	1
Pgm3	-0.06624	0.421448	1
Rabep1	-0.06634	0.352333	1
Gtpbp10	-0.0664	0.074735	1
Cox6a1	-0.06644	0.44225	1
Atp5a1	-0.06647	0.186717	1
Mcoln2	-0.06654	0.322021	1
Ccnd2	-0.06659	0.489944	1
Chd9	-0.06659	0.296198	1
Ptp4a2	-0.06665	0.441482	1
Ormdl3	-0.06666	0.430391	1
Polr2i	-0.06666	0.553213	1
Atp8b2	-0.06672	0.066855	1
Cenpq	-0.06676	0.167895	1
Apeh	-0.06677	0.24969	1
Mat2a	-0.06683	0.317692	1
Stau1	-0.06697	0.58928	1
Smim24	-0.06701	0.307523	1
Sbno1	-0.06701	0.478837	1
A330023F24I	-0.06707	0.049692	1
Trappc10	-0.06707	0.123447	1
Ppp6c	-0.06718	0.254245	1

Med26	-0.06726	0.079673	1
Ulk2	-0.06728	0.189645	1
Zfp592	-0.0673	0.808042	1
Ric8a	-0.0673	0.411266	1
U2af2	-0.06735	0.405523	1
Alkbh8	-0.06736	0.333225	1
Nsun6	-0.06739	0.922663	1
Agk	-0.06739	0.110377	1
Smyd4	-0.06745	0.168384	1
Pitrm1	-0.06748	0.695814	1
B3galnt2	-0.0675	0.414563	1
Smc5	-0.06752	0.210828	1
Kansl2	-0.06757	0.919382	1
Nudt22	-0.06761	0.738764	1
Mical1	-0.06764	0.23439	1
Bcl10	-0.06767	0.349512	1
Gm11110	-0.0677	0.470627	1
Slc41a1	-0.06771	0.129378	1
Asxl1	-0.06776	0.128199	1
C1d	-0.06785	0.223546	1
Ctbp1	-0.06789	0.319439	1
Nubp1	-0.0679	0.267042	1
Zfp51	-0.06794	0.659507	1
Tepsin	-0.06797	0.079478	1
Calcoco1	-0.06798	0.444552	1
Pwwp2a	-0.06801	0.159895	1
Mad1l1	-0.06808	0.608865	1
Rnf214	-0.0681	0.118981	1
Setd1b	-0.06816	0.090724	1
Tm2d2	-0.06823	0.49859	1
Zc3h14	-0.06824	0.02854	1
Fbxw4	-0.06828	0.864132	1
Tsg101	-0.06832	0.163138	1
Tcf3	-0.06841	0.573601	1
Rmc1	-0.06848	0.821585	1
Rbm5	-0.06853	0.299191	1
Tmem126a	-0.06854	0.389825	1
Uqcr11	-0.06858	0.730949	1
Rnf169	-0.06861	0.332307	1
Rps17	-0.06864	0.647181	1
Naaa	-0.06874	0.021425	1
Gaa	-0.06875	0.645608	1
Ccdc186	-0.06887	0.955934	1
Snx25	-0.06891	0.328014	1
Cbwd1	-0.06899	0.466266	1
Ccdc138	-0.06901	0.041631	1
Rcn1	-0.06902	0.691895	1
Xpc	-0.06902	0.163025	1

Sec61a2	-0.06905	0.074284	1
Mxd4	-0.06907	0.324263	1
Popdc2	-0.06909	0.055075	1
Kdm8	-0.06915	0.113622	1
Elmo1	-0.06917	0.382114	1
Dazap2	-0.0692	0.12758	1
Nxn	-0.06922	0.288648	1
Anapc1	-0.06922	0.761335	1
Bloc1s2	-0.06924	0.43404	1
Mef2d	-0.06928	0.17331	1
Naa50	-0.06938	0.620759	1
Rabif	-0.06944	0.124885	1
Dxo	-0.06946	0.197424	1
Dctn1	-0.06947	0.340373	1
Arpp19	-0.06947	0.233276	1
Gm5141	-0.06953	0.221491	1
Rsph3a	-0.06961	0.529564	1
Smpd2	-0.06967	0.040585	1
Abcb7	-0.0697	0.623576	1
Oxr1	-0.06982	0.768426	1
Bcas2	-0.06984	0.132675	1
Zfp7	-0.06986	0.009554	1
Wdr4	-0.06992	0.40998	1
C330007P06I	-0.07003	0.470948	1
Usp10	-0.07004	0.381135	1
Timm17a	-0.07004	0.527772	1
Tmem9b	-0.07015	0.727571	1
Ddx3y	-0.07016	0.155856	1
Ddx59	-0.07018	0.185243	1
Tnfrsf1a	-0.07023	0.142977	1
Ext2	-0.07028	0.580158	1
Gigyf1	-0.07033	0.091433	1
Tm9sf2	-0.07033	0.377857	1
Usp4	-0.07041	0.293249	1
Hadh	-0.07042	0.825003	1
Ranbp1	-0.07044	0.62146	1
Hdac3	-0.07058	0.707758	1
Scoc	-0.07062	0.736816	1
Cers6	-0.07062	0.371289	1
Borcs5	-0.07062	0.337541	1
Zbtb5	-0.07065	0.038764	1
Pxmp2	-0.07075	0.03598	1
Vdac3	-0.07082	0.3376	1
Eef1akmt2	-0.07086	0.252567	1
Taok1	-0.0709	0.162654	1
Lime1	-0.07092	0.134415	1
Mbnl3	-0.07102	0.368702	1
Sart3	-0.07105	0.898598	1

Cwf19l2	-0.07106	0.365058	1
Tmem115	-0.0711	0.723839	1
Akt3	-0.07113	0.349821	1
Fam57a	-0.07114	0.778256	1
Gadd45g	-0.07129	0.88119	1
Emc8	-0.07139	0.477892	1
Dusp5	-0.07141	0.23435	1
Auh	-0.07142	0.180506	1
Sec24a	-0.07146	0.677098	1
Pigv	-0.07148	0.12675	1
Fbxw7	-0.07155	0.297417	1
Msl2	-0.07157	0.743535	1
St13	-0.0717	0.788205	1
Phc2	-0.0718	0.98293	1
Ccdc71	-0.07181	0.175883	1
Uspl1	-0.07184	0.399218	1
Nudt5	-0.07194	0.423939	1
Bcr	-0.07195	0.225801	1
Mpc1	-0.07195	0.779156	1
Mis18a	-0.07199	0.093664	1
Rap1b	-0.07206	0.42861	1
Adk	-0.07216	0.651484	1
Gfm2	-0.07218	0.785512	1
Pelo	-0.07223	0.86411	1
Plpbp	-0.0723	0.108391	1
Pgrmc1	-0.0724	0.521685	1
Zfand3	-0.07243	0.912109	1
Morf4l1	-0.07249	0.719283	1
Washc1	-0.0726	0.601493	1
Polr2l	-0.07274	0.240156	1
Sec23ip	-0.0729	0.36488	1
Tspyl3	-0.0729	0.064359	1
Pigyl	-0.07304	0.289239	1
Scai	-0.07307	0.215964	1
Ing1	-0.07307	0.063197	1
Rbm42	-0.0731	0.462071	1
Pnrc2	-0.07312	0.103238	1
2810403D21	-0.07316	0.574136	1
Vta1	-0.07317	0.522726	1
Celf1	-0.07322	0.164251	1
Thrap3	-0.07327	0.740266	1
4833439L19I	-0.07329	0.279411	1
Cdk2ap1	-0.07339	0.045157	1
Mllt3	-0.07342	0.123616	1
Zbtb45	-0.07343	0.127807	1
Bicral	-0.07357	0.154977	1
Alyref2	-0.07361	0.72355	1
Fopnl	-0.07376	0.184867	1

Trip12	-0.07384	0.096214	1
Ldlrad4	-0.07386	0.97771	1
Zbed4	-0.07387	0.068728	1
Lrch4	-0.07388	0.191686	1
Zzef1	-0.07389	0.615716	1
Gns	-0.07395	0.068577	1
Gins3	-0.074	0.138492	1
Mrgbp	-0.07404	0.506865	1
Apobec1	-0.07406	0.527285	1
Rab7	-0.07408	0.235626	1
Tm2d1	-0.07409	0.322688	1
Chmp5	-0.0741	0.646393	1
Ltv1	-0.0741	0.743594	1
A930006K02I	-0.07412	0.180905	1
Coasy	-0.07419	0.395648	1
Ppm1d	-0.0742	0.358992	1
4930453N24	-0.07429	0.12699	1
Zfp958	-0.07432	0.551681	1
Cox7c	-0.07432	0.444662	1
Dvl3	-0.07437	0.027209	1
E130308A19I	-0.07441	0.122379	1
Fkbp8	-0.07449	0.272755	1
Cyp51	-0.07452	0.177441	1
Dclk2	-0.07469	0.054211	1
Bckdha	-0.07469	0.378688	1
Itprip	-0.07472	0.170188	1
Pms2	-0.07479	0.365901	1
Exo1	-0.07485	0.01563	1
Erp29	-0.07485	0.972332	1
Slc10a3	-0.07487	0.228434	1
Mbnl2	-0.0749	0.627411	1
Zc3h7b	-0.07499	0.187612	1
Btd	-0.07506	0.039925	1
Ost4	-0.07512	0.142721	1
Pten	-0.07518	0.261687	1
Lsm5	-0.07522	0.799226	1
Npat	-0.07525	0.099792	1
Rnf4	-0.07528	0.5488	1
Ntmt1	-0.07534	0.792225	1
mt-Nd3	-0.07538	0.307144	1
Ergic3	-0.0754	0.601344	1
Mpdu1	-0.07543	0.467822	1
Tasor	-0.07547	0.364612	1
Antxr2	-0.07556	0.039676	1
Dhx16	-0.07558	0.139028	1
Bicra	-0.07562	0.373601	1
Cep295	-0.07563	0.137294	1
Akr1b3	-0.07566	0.613621	1

Riok1	-0.0757	0.118458	1
Fut11	-0.0757	0.080161	1
Btaf1	-0.07572	0.596661	1
Zfp553	-0.07576	0.723718	1
Vav1	-0.07585	0.224828	1
Brd1	-0.07586	0.245346	1
Ap3m1	-0.0759	0.125618	1
Lemd3	-0.07595	0.375334	1
Btbd7	-0.07602	0.284656	1
Gtpbp3	-0.07611	0.1706	1
Nbr1	-0.07619	0.755862	1
Casp9	-0.07623	0.432944	1
Gm13212	-0.07623	0.17354	1
Atp6v1f	-0.07624	0.107793	1
Eif6	-0.07627	0.230065	1
Ctsz	-0.07632	0.249593	1
Akap10	-0.07637	0.901369	1
Mrps17	-0.07643	0.939436	1
Naxd	-0.0765	0.186195	1
Arhgap27	-0.07653	0.454457	1
Wbp2	-0.07656	0.240369	1
Lrrfip2	-0.0766	0.133692	1
Arl5b	-0.07662	0.265781	1
Snhg3	-0.07664	0.437198	1
Wrnip1	-0.07664	0.11902	1
Bri3bp	-0.07681	0.8682	1
Ubc	-0.07683	0.549824	1
Gen1	-0.07685	0.002304	1
Fance	-0.07686	0.051289	1
Svbp	-0.07689	0.929879	1
Senp3	-0.0769	0.076992	1
Ykt6	-0.0769	0.189916	1
Frrs1	-0.07697	0.086053	1
Med25	-0.07703	0.177754	1
Coil	-0.07708	0.270605	1
Sdhaf4	-0.07711	0.606762	1
5830408C22	-0.07713	0.211837	1
Vps53	-0.07715	0.096172	1
Kif19a	-0.07721	0.002345	1
Ppox	-0.0773	0.036301	1
Spdl1	-0.07742	0.053532	1
Rab12	-0.07745	0.101547	1
Tpm1	-0.07746	0.272564	1
Iws1	-0.07746	0.256199	1
Uchl3	-0.07748	0.492412	1
Micu1	-0.07752	0.513584	1
Mxi1	-0.07752	0.103194	1
Wdr82	-0.07754	0.831335	1

Jdp2	-0.07754	0.308721	1
Gata3	-0.07755	0.789661	1
Rab6a	-0.07757	0.679592	1
Tdp1	-0.07765	0.010661	1
Ash2l	-0.07767	0.216809	1
Htatip2	-0.07774	0.319255	1
BC005624	-0.07779	0.978574	1
1700096K18I	-0.07783	0.45996	1
Adh5	-0.07784	0.331049	1
Mbd1	-0.07785	0.879724	1
Frmd4a	-0.07791	0.812305	1
Nudcd3	-0.07791	0.688147	1
Pcm1	-0.07796	0.209359	1
B4galt7	-0.07799	0.881513	1
Ptpn13	-0.07799	0.432738	1
Sms	-0.07804	0.384034	1
Donson	-0.07804	0.095585	1
Ipp	-0.07805	0.072847	1
Srsf10	-0.07807	0.158219	1
Epg5	-0.07807	0.816773	1
Rcl1	-0.07809	0.604132	1
Rnf219	-0.07817	0.685165	1
Sec61g	-0.07828	0.11745	1
Cuedc1	-0.07829	0.071516	1
Lrch3	-0.0783	0.591058	1
Hsf4	-0.07835	0.07651	1
Gnptg	-0.07836	0.128916	1
Col4a3bp	-0.07844	0.241647	1
March7	-0.07847	0.976312	1
Ptrhd1	-0.07849	0.522665	1
Lasp1	-0.07873	0.493724	1
Rpain	-0.0788	0.584585	1
Nsd1	-0.07888	0.313928	1
Klf16	-0.0789	0.269438	1
Arhgap35	-0.07891	0.124893	1
Ndufaf7	-0.07892	0.580933	1
Frmd4b	-0.07901	0.949798	1
Fam49b	-0.07904	0.512258	1
Ttc37	-0.07911	0.011902	1
Psmd5	-0.07912	0.934317	1
Them6	-0.0792	0.257999	1
Sertad2	-0.07929	0.252676	1
Nfatc2ip	-0.07933	0.432301	1
Smarcd1	-0.07942	0.143823	1
Zfp329	-0.07945	0.173258	1
Ilvbl	-0.07948	0.105222	1
Slc12a4	-0.0795	0.040932	1
Bptf	-0.07951	0.404984	1

Ier2	-0.07967	0.659886	1
Rab35	-0.0797	0.025312	1
Rad17	-0.07971	0.328092	1
Sumf2	-0.07977	0.073313	1
Arpc3	-0.07978	0.099318	1
Ccdc47	-0.07985	0.433399	1
Clcn3	-0.07988	0.2622	1
Tbpl1	-0.07988	0.041718	1
Mbtps2	-0.07997	0.06517	1
Ankle1	-0.08002	0.000348	1
Tradd	-0.08002	0.205788	1
Zhx3	-0.08023	0.014563	1
Bin3	-0.08024	0.765573	1
Cobll1	-0.08035	0.24824	1
Stk4	-0.08039	0.267552	1
Capn7	-0.0804	0.294311	1
Morc3	-0.08044	0.882286	1
Sema4b	-0.08054	0.264687	1
Gm5165	-0.08056	0.535836	1
Iqsec1	-0.08057	0.278535	1
Gemin6	-0.08059	0.403052	1
Prcp	-0.08064	0.763805	1
Dars2	-0.08071	0.016358	1
Psmb4	-0.08071	0.824727	1
Cyb561d2	-0.08085	0.060995	1
Cfl2	-0.0809	0.794815	1
Ahcyl2	-0.08099	0.524015	1
Rtca	-0.081	0.423654	1
Zfp809	-0.08101	0.088148	1
Rbm15	-0.08111	0.172361	1
Tmem63a	-0.08113	0.845268	1
Nhp2	-0.08122	0.404173	1
Akip1	-0.08124	0.129559	1
Gss	-0.0813	0.160334	1
Ypel2	-0.08146	0.076059	1
Srgap3	-0.08146	0.444231	1
Drg1	-0.08151	0.470877	1
Ppp4r1	-0.08158	0.024219	1
Rbm14	-0.08175	0.080425	1
Lnx2	-0.08176	0.346012	1
Rab6b	-0.08176	0.005207	1
Pde12	-0.08188	0.426774	1
Tram1	-0.08188	0.528095	1
Rpgrip1	-0.08193	0.047334	1
Sf3a2	-0.08193	0.072732	1
Eef1akmt1	-0.08197	0.420921	1
Gdi2	-0.082	0.430805	1
Ssbp1	-0.08211	0.92667	1

Chst2	-0.08219	0.00654	1
Cul4b	-0.08235	0.08457	1
Bcdin3d	-0.08253	0.242923	1
Mtif3	-0.08258	0.594043	1
Ccar2	-0.08259	0.071385	1
Telo2	-0.08263	0.40887	1
Surf4	-0.08266	0.440329	1
Mppe1	-0.08271	0.187422	1
Zfp69	-0.08273	0.012818	1
Mbnl1	-0.0828	0.205762	1
Rcn2	-0.08291	0.832181	1
Tmc8	-0.08294	0.601611	1
Cdkn2aip	-0.08294	0.223778	1
Agpat1	-0.08296	0.159303	1
Spns1	-0.08296	0.07229	1
Gga1	-0.08299	0.106991	1
Men1	-0.08306	0.123137	1
Hdac5	-0.08321	0.328179	1
Phlpp2	-0.08322	0.038595	1
Tfdp1	-0.08329	0.161284	1
Msh5	-0.08341	6.3E-05	1
Blmh	-0.08351	0.873324	1
Csnk1g2	-0.08354	0.528709	1
4930503L19I	-0.08362	0.095986	1
Hmces	-0.08364	0.836501	1
Btbd10	-0.08368	0.352073	1
Taf9b	-0.08372	0.078101	1
Tnfaip8l2	-0.08381	0.974789	1
Nit1	-0.08384	0.83517	1
Ccdc59	-0.08385	0.299954	1
Znrd2	-0.0839	0.276081	1
Rpia	-0.08393	0.713106	1
Fbxl14	-0.08401	0.155483	1
Mfsd10	-0.08409	0.41934	1
Rab21	-0.08412	0.689429	1
Pip4k2c	-0.08416	0.259857	1
Cd81	-0.08424	0.625254	1
Dynll2	-0.08424	0.191743	1
Cpsf2	-0.08425	0.173904	1
Vps35	-0.08425	0.273541	1
Vbp1	-0.08426	0.045842	1
Capn15	-0.08433	0.072218	1
Pmvk	-0.08438	0.087149	1
Gm43813	-0.08445	0.022447	1
Ppp1r21	-0.08448	0.101517	1
Rabgef1	-0.08452	0.015007	1
Zbtb8os	-0.08456	0.233204	1
Ikbkg	-0.08458	0.318311	1

2700049A03I	-0.08463	0.141028	1
Lmf2	-0.08464	0.328817	1
Tsc22d4	-0.08466	0.14135	1
Utp3	-0.08475	0.11545	1
Dnajc18	-0.08484	0.491738	1
St6gal1	-0.08488	0.90718	1
Lztr1	-0.08489	0.716774	1
Spg11	-0.08491	0.263374	1
Dync1h1	-0.08496	0.304998	1
Hprt	-0.08507	0.454593	1
Cbfb	-0.08507	0.27847	1
Lsm3	-0.08508	0.213924	1
Zswim6	-0.08511	0.731003	1
Lysmd4	-0.08515	0.207519	1
Mrpl40	-0.08516	0.198469	1
Ino80c	-0.08527	0.972627	1
Zfp317	-0.08529	0.029011	1
Slc52a3	-0.08529	0.624186	1
Sapcd2	-0.08533	0.075776	1
Ttc13	-0.0854	0.127243	1
Zwilch	-0.08548	0.03867	1
Ndufv2	-0.08551	0.529992	1
Cant1	-0.08553	0.692498	1
Nfx1	-0.08553	0.251222	1
Smg7	-0.08554	0.60973	1
Lpar5	-0.08557	0.220935	1
Acsl4	-0.08562	0.147526	1
Ddost	-0.08569	0.205672	1
Dusp10	-0.08573	0.499775	1
Acoxl	-0.08582	0.194248	1
Nup37	-0.08586	0.105926	1
Nup205	-0.08587	0.223166	1
Vdac1	-0.08607	0.382663	1
Prkrip1	-0.08612	0.041368	1
Thoc6	-0.08613	0.198158	1
Msto1	-0.08636	0.449597	1
Pcbp4	-0.08638	0.001856	1
Snrnp40	-0.08647	0.075766	1
Far1	-0.08648	0.207477	1
Nfkb1	-0.08655	0.755512	1
Phf8	-0.08658	0.054823	1
Ppig	-0.08658	0.93508	1
Cxxc1	-0.08661	0.391473	1
Ppat	-0.08663	0.204193	1
Ghitm	-0.08671	0.607289	1
Isoc2a	-0.08672	0.108236	1
Pip5k1c	-0.08676	0.066301	1
BC005561	-0.0868	0.078446	1

Dhrs1	-0.08692	0.212173	1
Exosc10	-0.08692	0.149452	1
Tcrg-C4	-0.08696	0.000189	1
Ndufa1	-0.08703	0.545725	1
Naa40	-0.08716	0.285888	1
Arl5a	-0.08728	0.02273	1
Panx1	-0.08741	0.060489	1
Psmd7	-0.08743	0.888227	1
Dnajc8	-0.08746	0.237789	1
Mnat1	-0.08752	0.285816	1
Vdac2	-0.08757	0.921252	1
Luc7l2	-0.08759	0.182971	1
Ralgps1	-0.08762	0.049356	1
Slx4	-0.08763	0.004421	1
Bspry	-0.08772	0.077575	1
Atl2	-0.08777	0.166641	1
Psat1	-0.08797	0.065506	1
Kif21b	-0.088	0.227101	1
Fam92a	-0.08802	0.661667	1
Snrpe	-0.08807	0.232122	1
Slc22a15	-0.08809	0.096275	1
Rab33b	-0.08817	0.057577	1
Bysl	-0.08821	0.05834	1
Yipf5	-0.08835	0.57252	1
Zfp518a	-0.08841	0.27031	1
Tpd52	-0.08849	0.024597	1
Rad54l	-0.08852	0.154118	1
Mllt11	-0.08856	0.047637	1
Ints6l	-0.08858	0.16639	1
Dmxl1	-0.08861	0.708561	1
Rtel1	-0.08864	0.324865	1
Inpp5d	-0.08865	0.289481	1
Mapk1	-0.08865	0.244575	1
Ppp6r3	-0.08892	0.611628	1
Rhno1	-0.08898	0.17628	1
Mrps22	-0.08902	0.034541	1
Hvcn1	-0.08903	0.472826	1
Tmem68	-0.08903	0.036361	1
Isyna1	-0.08921	0.956226	1
Scyl1	-0.08942	0.569384	1
Pitpnm1	-0.08946	0.127912	1
Cops5	-0.0895	0.792201	1
Fam45a	-0.08962	0.450133	1
Nol10	-0.08969	0.18519	1
Gfpt1	-0.08971	0.477967	1
Setx	-0.08971	0.186295	1
Wipf1	-0.08972	0.430494	1
Cul3	-0.08978	0.356308	1

Fancd2	-0.0898	0.042764	1
Thg1l	-0.08989	0.336103	1
Anxa4	-0.08991	0.714283	1
Coq8b	-0.08995	0.227408	1
Dnaaf2	-0.08995	0.088199	1
Mthfd1l	-0.09	0.794389	1
Pphln1	-0.09003	0.050674	1
Dscc1	-0.09007	0.113314	1
Mrpl39	-0.0902	0.222298	1
Rpp30	-0.0902	0.119793	1
Cdca7	-0.09021	0.045174	1
Fbxo22	-0.09022	0.072277	1
Phlda3	-0.09026	7.31E-05	1
Srp68	-0.09026	0.751733	1
Elp4	-0.09037	0.557623	1
Nkap	-0.09042	0.375691	1
Kif5b	-0.09043	0.210402	1
Fmr1	-0.09051	0.036834	1
Ptpre	-0.09059	0.275413	1
9830144P21l	-0.09067	0.124575	1
Gm26917	-0.09076	0.262862	1
Tmem229b	-0.09079	0.468095	1
1700056E22l	-0.09087	0.054379	1
Irf5	-0.09091	0.393094	1
Pias4	-0.09104	0.308304	1
Psmb5	-0.09109	0.512547	1
Cd84	-0.09127	0.191434	1
Nckap1l	-0.09131	0.283711	1
Foxn2	-0.09142	0.163178	1
Tmbim6	-0.09143	0.114382	1
Lipa	-0.09157	0.065134	1
Zfp729a	-0.09159	0.005842	1
L1cam	-0.09159	0.257084	1
Tcp1	-0.09159	0.341405	1
Ganc	-0.0916	0.927512	1
Senp5	-0.09173	0.158052	1
Odf2l	-0.09176	0.21727	1
Med13	-0.0918	0.571433	1
Ugdh	-0.0918	0.473921	1
Ankrd49	-0.09186	0.108857	1
Tcn2	-0.09192	0.300194	1
Exoc6	-0.09193	0.13611	1
Rad1	-0.09202	0.373931	1
Map11	-0.0922	0.119335	1
Exosc3	-0.0922	0.390178	1
Map1s	-0.09221	0.349354	1
March2	-0.09223	0.574209	1
Cacfd1	-0.09225	0.705979	1

Clcn4	-0.09225	0.214379	1
Txlna	-0.09226	0.37285	1
Chid1	-0.09226	0.052722	1
Strn3	-0.09229	0.124794	1
Fermt3	-0.0924	0.555165	1
Dgcr8	-0.09244	0.055003	1
Phpt1	-0.09246	0.134528	1
Ubxn7	-0.0925	0.485245	1
Smu1	-0.09261	0.176788	1
Pidd1	-0.09269	0.042237	1
2310022A10I	-0.09271	0.15423	1
Dlg1	-0.09283	0.138394	1
Slc35f2	-0.09293	0.167569	1
Rad51b	-0.09297	0.07773	1
Ankrd26	-0.09299	0.331127	1
Haus6	-0.09308	0.048191	1
Anxa11	-0.09309	0.701722	1
Foxd2os	-0.09317	0.010927	1
Slc30a6	-0.09324	0.081559	1
Gins2	-0.09326	0.079353	1
Mrps14	-0.09328	0.292539	1
Usp37	-0.09342	0.311893	1
Commd2	-0.09346	0.117751	1
Ttc28	-0.09347	0.044001	1
Trip4	-0.09353	0.174873	1
Acyp1	-0.09363	0.009599	1
Ncbp2	-0.09364	0.039168	1
Cep104	-0.09364	0.679772	1
Fbxo25	-0.09369	0.584709	1
Tfip11	-0.09376	0.0174	1
Sf3b4	-0.09393	0.548086	1
Camkk2	-0.09395	0.087826	1
Ythdc2	-0.09396	0.129841	1
Cep250	-0.09399	0.266483	1
Glrx3	-0.094	0.765659	1
Kifc5b	-0.09402	0.141935	1
Rpl7l1	-0.09421	0.437676	1
Pde4a	-0.09422	0.136496	1
Mrfap1	-0.09424	0.356167	1
Mettl17	-0.09432	0.341694	1
Pex1	-0.09451	0.031052	1
Shld1	-0.09455	0.057023	1
Rfk	-0.09458	0.883512	1
Atp13a1	-0.09464	0.100114	1
Nup50	-0.09467	0.247944	1
Plrg1	-0.09482	0.038849	1
BC030867	-0.09491	0.000374	1
Cpox	-0.09492	0.408791	1

Arid1b	-0.09495	0.309524	1
Cyb5d2	-0.09501	0.000922	1
Tmem222	-0.09502	0.301149	1
Chek1	-0.09515	0.164555	1
Cenpv	-0.0952	0.00066	1
Dtnb	-0.09521	0.099994	1
Gnai3	-0.09529	0.533025	1
Kif2a	-0.09531	0.876172	1
Gm14029	-0.09539	0.222588	1
Prpf40a	-0.09544	0.196563	1
Wnk1	-0.09545	0.183224	1
Umad1	-0.09558	0.678671	1
Sf3b3	-0.09567	0.749821	1
Cox6b1	-0.09568	0.171719	1
Ptch1	-0.09587	0.346551	1
Tm9sf4	-0.0959	0.226399	1
Gm33782	-0.09599	0.058482	1
Xylt1	-0.09604	0.165173	1
Cdc42ep3	-0.09605	0.517346	1
Creg1	-0.09605	0.982526	1
Slf2	-0.09612	0.14655	1
Psmc6	-0.09615	0.325497	1
Etv3	-0.09619	0.311848	1
Mlec	-0.09619	0.413416	1
Selenot	-0.09621	0.505684	1
Tmem171	-0.09633	0.097596	1
Bud13	-0.09634	0.172446	1
Gatad2b	-0.09638	0.107248	1
Zfp407	-0.09645	0.090611	1
Gbf1	-0.09645	0.507988	1
Mphosph9	-0.09651	0.138147	1
Lmnb2	-0.09655	0.011742	1
Pstk	-0.09656	0.209535	1
Ints10	-0.09657	0.264705	1
Idi1	-0.09666	0.524557	1
Pik3c2a	-0.09666	0.135215	1
N4bp2	-0.09672	0.044411	1
Ercc6l	-0.09672	4.65E-07	0.015019
Otub1	-0.09673	0.467178	1
Smim11	-0.09675	0.280901	1
Gm20069	-0.09676	0.045653	1
Cd164	-0.0968	0.542467	1
Abhd5	-0.09682	0.01149	1
Tstd2	-0.09692	0.078744	1
Slc12a6	-0.09694	0.244224	1
Sf3a3	-0.09697	0.0964	1
Syne2	-0.09711	0.044195	1
Puf60	-0.09719	0.186366	1

Asb7	-0.09719	0.01642	1
Ralbp1	-0.0972	0.585473	1
Grb2	-0.09726	0.254192	1
Ythdf2	-0.09727	0.563287	1
Moap1	-0.09733	0.008219	1
Xndc1	-0.09745	0.028652	1
Taf1	-0.09746	0.315024	1
Papss1	-0.09761	0.121779	1
Med21	-0.09767	0.023868	1
Copg2	-0.09769	0.191022	1
Heatr9	-0.09771	0.000832	1
Fdxr	-0.09772	0.017654	1
Haus1	-0.09774	0.064194	1
Raph1	-0.09795	0.262901	1
Pigt	-0.09796	0.190401	1
Bclaf3	-0.09799	0.008369	1
Ddx23	-0.09802	0.283882	1
Cxcr4	-0.09815	0.053177	1
Smndc1	-0.09822	0.055701	1
Usp14	-0.0983	0.046474	1
Mtmr6	-0.09842	0.250337	1
Yeats4	-0.09846	0.229545	1
Inpp5f	-0.09846	0.106292	1
Gtf2h2	-0.09851	0.03698	1
Itih5	-0.09853	0.265685	1
Trmt13	-0.09854	0.684432	1
9530068E07I	-0.0988	0.071076	1
Mrpl44	-0.09881	0.275923	1
Setd2	-0.09906	0.274702	1
Sept6	-0.09907	0.097269	1
Cyb5r3	-0.09908	0.19267	1
Elf2	-0.0991	0.108913	1
Acer3	-0.09912	0.163954	1
Armc1	-0.09918	0.244887	1
Gtse1	-0.09919	0.108016	1
Nip7	-0.09921	0.671856	1
Mfsd14a	-0.09933	0.175243	1
Pdk3	-0.09934	0.112971	1
Cnot7	-0.09938	0.067951	1
Acd	-0.09939	0.077451	1
Slx4ip	-0.09943	0.87794	1
2610318N02	-0.09945	0.029908	1
Pck2	-0.09955	0.108134	1
Zmym5	-0.09955	0.055427	1
Gm30054	-0.09955	0.342987	1
Dync2h1	-0.09962	0.056049	1
Tgoln1	-0.09964	0.751321	1
Dgkz	-0.09968	0.927189	1

Bard1	-0.09969	0.01176	1
Anp32a	-0.09971	0.109938	1
U2surp	-0.09973	0.396989	1
Neat1	-0.09976	0.181746	1
Ndufa13	-0.09982	0.049993	1
Naa16	-0.09988	0.249625	1
Cdc27	-0.09988	0.352706	1
Ppt1	-0.09988	0.079642	1
Rnf31	-0.09991	0.003639	1
Yif1a	-0.09993	0.030586	1
Lcor	-0.09996	0.441569	1
Pml	-0.10002	0.129226	1
Gins4	-0.10007	0.222148	1
Acsbg1	-0.10022	0.06597	1
Adck5	-0.1003	0.351144	1
3110009E18I	-0.10031	0.061363	1
Lcmt2	-0.10033	0.130821	1
Lmbr1l	-0.10033	0.201205	1
Ttc33	-0.10036	0.10592	1
Mindy1	-0.1004	0.202064	1
Trp53inp1	-0.10044	0.085403	1
Pcbp1	-0.10046	0.094966	1
Dennd1c	-0.1005	0.019311	1
Pif1	-0.1006	0.010307	1
Rbm6	-0.1006	0.094529	1
Srsf6	-0.10068	0.594012	1
Jpt2	-0.10082	0.021698	1
Zkscan17	-0.10087	0.119122	1
Syne3	-0.10096	0.240567	1
Ywhae	-0.10097	0.221082	1
Rb1	-0.10124	0.202984	1
Ccdc22	-0.10128	0.010439	1
Pik3cg	-0.10132	0.74926	1
Zfp710	-0.10134	0.033907	1
Umps	-0.10136	0.118783	1
Gcn1	-0.10136	0.293591	1
Wdr91	-0.1014	0.21735	1
Zc3h11a	-0.10146	0.030606	1
Cd1d1	-0.10151	0.017998	1
Lrrc42	-0.10155	0.363355	1
Dcaf17	-0.10156	0.02556	1
Rhot1	-0.10158	0.280366	1
Btbd1	-0.10159	0.107168	1
Rev3l	-0.10161	0.115046	1
Polh	-0.10161	0.177112	1
Mgat5	-0.10168	0.743936	1
Hdgfl3	-0.10177	0.402192	1
Spidr	-0.10198	0.006791	1

Ccdc88b	-0.10202	0.19207	1
Mrpl43	-0.10212	0.184454	1
Mrps15	-0.10217	0.649291	1
1300002E11I	-0.10221	0.193244	1
Micos10	-0.10229	0.244243	1
Recql5	-0.10233	0.143382	1
Grpel2	-0.10236	0.012358	1
Ensa	-0.10238	0.062174	1
Ddx47	-0.10245	0.399687	1
Cggbp1	-0.10247	0.126412	1
Tonsl	-0.10248	0.038449	1
Ints1	-0.10252	0.4038	1
Zfp3	-0.1026	0.004387	1
Thap3	-0.1027	0.25309	1
S100a1	-0.10289	0.038691	1
Pxk	-0.10301	0.321341	1
Zfp707	-0.10304	0.006569	1
Ebp	-0.10306	0.043493	1
Mapk7	-0.10308	0.129477	1
Ddhd1	-0.10309	0.508259	1
Tmed9	-0.1031	0.273758	1
Mkrn2	-0.10316	0.253572	1
Rbms2	-0.10318	0.011341	1
Gabpb2	-0.1032	0.287899	1
Msrb1	-0.10324	0.311281	1
Uchl5	-0.10329	0.504001	1
Smad3	-0.10346	0.026984	1
Rgs2	-0.10357	0.563447	1
Vrk3	-0.10363	0.267424	1
Dnttip2	-0.10364	0.464998	1
Triap1	-0.10364	0.444092	1
Smap1	-0.10365	0.211355	1
Eif4a3	-0.10365	0.461764	1
Rps6ka5	-0.10372	0.023565	1
Zfp367	-0.10379	0.164935	1
Rtf1	-0.10394	0.100107	1
Prpf38b	-0.10402	0.416265	1
Asnsd1	-0.10402	0.073602	1
Ell	-0.10411	0.107978	1
Tmc6	-0.10419	0.182344	1
2310009A05I	-0.10421	0.211559	1
St3gal3	-0.10422	0.18897	1
Cog6	-0.10434	0.026868	1
Naglu	-0.10434	0.005873	1
Erlin2	-0.10454	0.004878	1
Mrps18b	-0.10479	0.839777	1
Fuca2	-0.10482	0.266979	1
Slc9a7	-0.10495	0.023295	1

Phf13	-0.10498	0.000103	1
Ddx52	-0.10509	0.170332	1
Cops3	-0.10511	0.100983	1
Sp1	-0.10513	0.577573	1
Rars	-0.10515	0.089156	1
Tspan4	-0.10517	0.338451	1
Zfp58	-0.10517	0.005271	1
Cdc73	-0.10521	0.146344	1
Trmt10a	-0.10523	0.128488	1
Gm26782	-0.10523	0.415034	1
Snapc5	-0.10533	0.087491	1
Ptdss1	-0.10552	0.052098	1
Psmc3	-0.10557	0.276261	1
Saraf	-0.10563	0.401119	1
Casp7	-0.10566	0.213177	1
Arl8b	-0.10571	0.485321	1
Tent2	-0.10585	0.178881	1
Traip	-0.10598	0.013468	1
Zbtb24	-0.10604	0.035954	1
Comt	-0.10613	0.545218	1
Rsrc2	-0.10613	0.253433	1
Nutf2	-0.10615	0.035481	1
Stip1	-0.10616	0.163036	1
Helq	-0.10617	0.001055	1
Rida	-0.10622	0.160371	1
Sh2d2a	-0.10623	0.693395	1
Nqo2	-0.10635	0.025055	1
Ublcp1	-0.10637	0.360613	1
Slc25a36	-0.10641	0.350868	1
Msh3	-0.10645	0.213303	1
Zfp266	-0.1065	0.825911	1
Hexim1	-0.10653	0.638111	1
Ranbp10	-0.10655	0.092842	1
Haus8	-0.10656	0.115962	1
Gm28901	-0.1067	0.077942	1
Tyw1	-0.10676	0.007518	1
Hirip3	-0.10684	0.087679	1
Snx17	-0.10689	0.195401	1
Dctpp1	-0.10689	0.52992	1
Zc3h15	-0.10693	0.235795	1
Psmd8	-0.10694	0.237912	1
Kif1c	-0.107	0.014086	1
Atp8a1	-0.10702	0.203833	1
Tpd52l2	-0.10702	0.869074	1
Rnasel	-0.10716	0.492094	1
Pcyt2	-0.10716	0.075799	1
Slc25a38	-0.10727	0.508805	1
Ccm2	-0.10743	0.052248	1

Ppdpf	-0.10745	0.579195	1
Ttc21b	-0.1075	0.02836	1
Ier3ip1	-0.10754	0.226023	1
Smim15	-0.1076	0.07916	1
Hexa	-0.10764	0.183524	1
Wdr20	-0.1077	0.095748	1
Chtf18	-0.10783	0.001892	1
Ubtf	-0.10791	0.737869	1
Dazap1	-0.10797	0.509338	1
Lyrm7	-0.10806	0.24882	1
Wdr95	-0.10808	0.00745	1
Eloa	-0.10809	0.170044	1
Zfp382	-0.10812	0.035562	1
Arfip1	-0.10813	0.071391	1
Nup35	-0.10815	0.262199	1
H2-Q7	-0.1082	0.082628	1
Cyb5b	-0.10828	0.109043	1
Cd2	-0.10841	0.36548	1
Serpinb9	-0.10841	0.521226	1
Hadha	-0.10845	0.211239	1
Sec23b	-0.10846	0.122	1
Oaz2	-0.1086	0.091488	1
Trim33	-0.10877	0.379183	1
Foxo1	-0.10878	0.816902	1
Mcl1	-0.1089	0.417233	1
Zfp513	-0.10906	0.002314	1
Hist1h3a	-0.10913	0.001473	1
Hist4h4	-0.10924	0.01626	1
Ccdc167	-0.10928	0.117426	1
Ccnt2	-0.1094	0.023172	1
Snrpa1	-0.1094	0.402024	1
Gm33460	-0.10948	2.85E-09	9.19E-05
Tmprss13	-0.10955	1.68E-11	5.42E-07
Ipo8	-0.10973	0.042062	1
Cgas	-0.10975	0.002749	1
Usp40	-0.1098	0.060571	1
Herpud1	-0.10982	0.747871	1
Vac14	-0.10983	0.394963	1
Tefm	-0.10995	0.339991	1
Gabpa	-0.10997	0.005503	1
Tardbp	-0.1101	0.287404	1
Ddx46	-0.11011	0.255565	1
Csnk1g3	-0.11016	0.076714	1
Gpn1	-0.11016	0.149359	1
Slc25a11	-0.11018	0.124876	1
Timeless	-0.11019	0.061886	1
Usp1	-0.11027	0.06435	1
Ap1s1	-0.11036	0.146103	1

Ndufb7	-0.11038	0.289474	1
Rtn4	-0.11064	0.532631	1
Sp2	-0.11066	0.067273	1
Pqbp1	-0.11072	0.21403	1
Uqcrc2	-0.1108	0.127477	1
Wipf2	-0.11083	0.274341	1
Pbdc1	-0.11087	0.087492	1
Usp22	-0.11103	0.377944	1
Entpd7	-0.11113	0.005062	1
Lamp2	-0.11114	0.851096	1
Cand1	-0.11124	0.199246	1
Stk39	-0.11136	0.38071	1
Ulk4	-0.11137	0.004027	1
Ddx11	-0.11143	0.008815	1
Mtfr2	-0.11146	0.044497	1
D6Wsu163e	-0.11148	0.009706	1
Bcap31	-0.11162	0.118681	1
Snx11	-0.11169	0.468684	1
Snx12	-0.11171	0.397931	1
Ing3	-0.11175	0.732791	1
Zdhhc20	-0.1118	0.472433	1
Taf6	-0.11186	0.10566	1
Ppp3ca	-0.11191	0.067978	1
2900026A02I	-0.11192	0.430109	1
Mdm1	-0.11192	0.028375	1
Manea	-0.11195	0.073787	1
Lsm1	-0.11195	0.111007	1
Sucla2	-0.112	0.150654	1
Rap2b	-0.11206	0.05672	1
Itgax	-0.11207	0.676264	1
Akna	-0.11208	0.352603	1
Snx4	-0.11208	0.327097	1
Anxa5	-0.11208	0.246133	1
Usp42	-0.11214	0.150472	1
Dhx15	-0.11215	0.313906	1
Mfsd14b	-0.1122	0.035671	1
Lars2	-0.11223	0.000812	1
Rin3	-0.11227	0.2465	1
Ccnb1ip1	-0.11227	0.000788	1
Fam76a	-0.11229	0.452903	1
B230307C23I	-0.11229	0.091925	1
Eri2	-0.11234	0.002671	1
Rpp25l	-0.11238	0.557895	1
Nae1	-0.1124	0.036532	1
Ggt1	-0.1125	6.9E-07	0.022266
Tyk2	-0.11259	0.029693	1
Chst10	-0.11268	0.031444	1
Ptpa	-0.11269	0.443233	1

Txn1	-0.11285	0.716453	1
Amd1	-0.11291	0.039464	1
Lin9	-0.11291	0.004419	1
S100pbp	-0.11294	0.303246	1
Armc3	-0.11294	0.431832	1
Fasl	-0.11296	0.360505	1
Klhl18	-0.11297	0.069135	1
Msh6	-0.11316	0.012305	1
Igsf8	-0.11333	0.909626	1
Gpcpd1	-0.11335	0.228245	1
Egln2	-0.11343	0.079485	1
Melk	-0.11347	0.011843	1
Atp5j2	-0.11367	0.036849	1
Ylpm1	-0.11373	0.221989	1
Anks1b	-0.11391	0.020182	1
Ppfia1	-0.11391	0.129535	1
Inf2	-0.11398	0.259837	1
Tnfaip8l1	-0.11399	0.216201	1
Tcp11l2	-0.11401	0.89277	1
Zfyve16	-0.11402	0.282074	1
Vamp1	-0.11404	0.033985	1
Nop58	-0.11407	0.424416	1
Eapp	-0.11412	0.309567	1
Praf2	-0.11415	0.100421	1
Gm15564	-0.11425	0.240346	1
Gm20663	-0.11427	0.121712	1
Tmem135	-0.11429	0.119676	1
Zfp384	-0.11436	0.044552	1
Arrdc1	-0.11459	0.174734	1
Commd4	-0.11487	0.44322	1
Szrd1	-0.11506	0.237702	1
Ankmy2	-0.11508	0.303596	1
Ier5	-0.11517	0.390831	1
Ythdf1	-0.11518	0.316044	1
Arpc2	-0.11525	0.066165	1
Kbtbd4	-0.11534	0.475215	1
Scrib	-0.11543	0.000744	1
Stard5	-0.11545	0.020258	1
Entpd1	-0.11547	0.056442	1
Gm50287	-0.11549	0.023244	1
Cstf2	-0.1155	0.028134	1
Ftl1	-0.1155	0.860691	1
Snx30	-0.1156	0.009119	1
Atf7	-0.11562	0.233697	1
Ap1g1	-0.11565	0.116765	1
Spag7	-0.1157	0.048705	1
Lemd2	-0.11571	0.041264	1
Lrrc8a	-0.11573	0.065264	1

Nxt1	-0.11576	0.220882	1
Slc30a5	-0.11577	0.477952	1
Fbxo28	-0.11579	0.163736	1
Zfp68	-0.11584	0.050822	1
Poc1a	-0.11589	0.075223	1
Smarca2	-0.1159	0.133399	1
Dnajc14	-0.1159	0.104461	1
Mtmr14	-0.11591	0.185834	1
Hibch	-0.11597	0.088156	1
Ccdc82	-0.11602	0.320628	1
Rabl3	-0.1161	0.110822	1
Mrpl17	-0.11617	0.64714	1
Inpp5b	-0.11621	0.137194	1
Mpv17	-0.11625	0.048927	1
Eif4g3	-0.11629	0.055664	1
Ccdc61	-0.1163	0.025575	1
Tfg	-0.1163	0.31191	1
Ccdc124	-0.11635	0.473333	1
Hnrnpdl	-0.1164	0.789271	1
Mios	-0.11644	0.327064	1
Fam3a	-0.11646	0.012169	1
Zranb1	-0.11657	0.077441	1
Tox2	-0.11663	0.003742	1
Clic4	-0.11666	0.54727	1
Cdc14b	-0.11666	0.003105	1
Armc5	-0.11668	0.027992	1
Wbp4	-0.11669	0.262518	1
Gusb	-0.11671	0.139079	1
Pnp	-0.11679	0.080202	1
Hikeshi	-0.11682	0.880718	1
Bcor	-0.11698	0.267554	1
Gpkow	-0.11714	0.197359	1
Pex5	-0.1172	0.077013	1
Prag1	-0.1172	0.017073	1
Arhgap25	-0.11736	0.625263	1
Gimap7	-0.11745	0.201789	1
Zdhhc21	-0.11751	0.435873	1
Dctn6	-0.11752	0.26962	1
Osbpl5	-0.11754	0.154993	1
Bbs9	-0.11755	0.322338	1
Gpr137b	-0.11757	9.62E-06	0.310529
Mbd4	-0.11766	0.003347	1
Vamp3	-0.11785	0.079533	1
Ctdspl2	-0.11786	0.421929	1
Ly9	-0.11788	0.117899	1
Gtf3a	-0.11792	0.073912	1
Sc5d	-0.11796	0.06358	1
Atp6v0a2	-0.118	0.025672	1

Midn	-0.1181	0.078211	1
Safb2	-0.11811	0.488659	1
Egln1	-0.11828	0.290504	1
Eif4b	-0.11829	0.15213	1
Brd3	-0.11838	0.134667	1
Spock2	-0.1184	0.002303	1
Alas1	-0.11843	0.092221	1
Rassf1	-0.1185	0.704824	1
Ctps2	-0.11852	0.12357	1
Tmem175	-0.11853	0.079244	1
Pqlc3	-0.11854	0.036405	1
Cnot3	-0.11855	0.004094	1
Zfp113	-0.11857	0.019285	1
Kmt5c	-0.11863	0.089681	1
Il16	-0.1188	0.453405	1
Gak	-0.11882	0.019078	1
Rbm27	-0.11888	0.425227	1
Pbrm1	-0.11905	0.640778	1
Arhgap18	-0.11911	0.737987	1
Foxm1	-0.11911	0.04908	1
Brd4	-0.11915	0.141171	1
Ikzf1	-0.11918	0.256627	1
Ttk	-0.11923	0.002103	1
Flot1	-0.11928	0.33726	1
Rbbp5	-0.11936	0.193034	1
Atad2b	-0.11941	0.464897	1
Fdps	-0.11946	0.693894	1
Hcfc1	-0.1195	0.069816	1
Fubp3	-0.1195	0.004158	1
Gps2	-0.11952	0.091559	1
Mms22l	-0.11953	0.093975	1
Chaf1a	-0.11962	0.115805	1
Cstf3	-0.11982	0.094348	1
Uso1	-0.11988	0.046819	1
Ireb2	-0.11991	0.216804	1
Orai2	-0.12007	0.121333	1
Klhl11	-0.12026	0.002908	1
Serpinb9b	-0.1203	0.747185	1
Fam111a	-0.12035	0.098321	1
Rprd2	-0.12039	0.29265	1
Ppm1b	-0.12044	0.02733	1
Osm	-0.12048	0.374475	1
Ruvbl2	-0.12069	0.043745	1
Dpp8	-0.12073	0.326297	1
Arcn1	-0.12096	0.621736	1
Morc2a	-0.12097	0.540349	1
Abhd11	-0.12101	0.156764	1
Dcp2	-0.12105	0.297485	1

Taf12	-0.12107	0.011912	1
Arrb2	-0.12127	0.651224	1
Zfp943	-0.12131	0.131859	1
Kdm2a	-0.12136	0.152259	1
Parp1	-0.1215	0.207494	1
Zbtb1	-0.1215	0.041157	1
Trappc4	-0.12162	0.101072	1
Scly	-0.12165	0.063115	1
E430024P14I	-0.12174	0.109668	1
Ppp1r10	-0.12178	0.183544	1
Cdc7	-0.12179	0.003039	1
Zw10	-0.12179	0.065833	1
Scaf1	-0.12189	0.036237	1
Shkbp1	-0.12193	0.069828	1
Dtx3	-0.12193	0.338243	1
Rhof	-0.12193	0.229396	1
Gm37240	-0.12206	0.029203	1
Rfx1	-0.12209	0.000942	1
4930579G24	-0.12211	0.007323	1
Bnip2	-0.12215	0.092425	1
Smim12	-0.12229	0.8097	1
Smarca5	-0.1223	0.088561	1
Pspc1	-0.1224	0.264273	1
Il17ra	-0.12245	0.304567	1
U2af1	-0.12246	0.258306	1
Kntc1	-0.12248	0.063282	1
Dhx8	-0.12267	0.032098	1
Ier3	-0.12271	0.009328	1
Rnf215	-0.1228	0.260052	1
Purb	-0.12283	0.067617	1
Srp54a	-0.12335	0.055626	1
Qrich1	-0.12335	0.176996	1
1110004F10I	-0.12344	0.5107	1
Cacna1d	-0.1235	0.009196	1
Emd	-0.12356	0.490728	1
Cltc	-0.12358	0.095624	1
Sfswap	-0.12366	0.100169	1
Nfatc3	-0.12369	0.191246	1
Pak2	-0.1237	0.233477	1
Smg9	-0.12375	0.052958	1
Csnk1d	-0.12384	0.655924	1
Csnk2a1	-0.12385	0.164751	1
Ubn1	-0.12387	0.172391	1
Xpo1	-0.12396	0.416712	1
Ppard	-0.12401	0.029508	1
Klhdc4	-0.12404	0.252794	1
Sh3gl1	-0.12414	0.280916	1
Fbxw8	-0.12429	0.01501	1

Prdx4	-0.12431	0.412411	1
Rnf6	-0.1244	0.027725	1
Abcd1	-0.1244	0.086044	1
Syt11	-0.12447	0.02336	1
Tcf12	-0.12452	0.268661	1
Hnrnpa0	-0.12471	0.009807	1
Chd3	-0.12471	0.13214	1
Heatr1	-0.12488	0.241726	1
Eif3j1	-0.12488	0.024829	1
Gmeb1	-0.12505	0.004387	1
Stt3a	-0.12519	0.11285	1
Atp11c	-0.12545	0.097218	1
Map3k1	-0.12549	0.04853	1
Sept4	-0.12552	2.31E-05	0.747163
Mir99ahg	-0.12556	2.11E-07	0.006806
Pdia3	-0.1257	0.303106	1
Ncald	-0.1257	0.106582	1
Vps36	-0.1258	0.34224	1
Fkbp4	-0.1258	0.329108	1
Mob1a	-0.12603	0.159094	1
Dapk3	-0.12613	0.193876	1
Osbpl2	-0.12613	0.124893	1
Plekha2	-0.12615	0.516621	1
Tnik	-0.12622	0.4675	1
Fam107b	-0.12632	0.206835	1
Msantd2	-0.12636	0.487475	1
Psmd11	-0.12646	0.077074	1
Dcaf7	-0.12657	0.056204	1
Pdzd11	-0.12659	0.691648	1
Scaf4	-0.12668	0.049042	1
Gpbp1	-0.1268	0.492904	1
Zfp931	-0.12695	0.032731	1
Brpf1	-0.12711	0.033041	1
Ndufa8	-0.12735	0.209457	1
Dennd4b	-0.12735	0.156868	1
Rrp9	-0.12747	0.260484	1
Relt	-0.12748	0.171628	1
Brwd3	-0.12754	0.036431	1
Uimc1	-0.12764	0.129807	1
Tmem256	-0.12768	0.470445	1
Drap1	-0.1277	0.008512	1
Mns1	-0.12774	0.012259	1
Alg13	-0.12777	0.402798	1
Arl2	-0.12794	0.924866	1
Nptn	-0.12804	0.183863	1
Polr2g	-0.12807	0.067619	1
Taok3	-0.12819	0.32444	1
Itsn2	-0.12819	0.122738	1

Gnpda1	-0.12826	0.056312	1
Copg1	-0.12827	0.648497	1
Snx13	-0.12853	0.123158	1
Rbx1	-0.1287	0.051297	1
Ints6	-0.12881	0.118488	1
Arap1	-0.12884	0.010596	1
Mta2	-0.12895	0.435502	1
Orc6	-0.12896	0.056737	1
Diaph2	-0.12902	0.744222	1
Accs	-0.12932	0.003708	1
Supt3	-0.12932	0.434759	1
Bbip1	-0.1294	0.019893	1
Pnn	-0.12965	0.112164	1
Rreb1	-0.12967	0.323421	1
Armc4	-0.12968	2.7E-21	8.72E-17
Slc43a3	-0.12989	0.019737	1
Pak1ip1	-0.12993	0.303686	1
Ap1s3	-0.12994	0.036364	1
Hectd3	-0.13002	0.009008	1
Pdcd2l	-0.13005	0.237209	1
Sgk1	-0.13008	0.090412	1
Mrpl10	-0.13011	0.076586	1
Ndufa11	-0.13023	0.210229	1
Adam10	-0.13029	0.254225	1
Rnf141	-0.13037	0.001534	1
Clasp2	-0.13038	0.006978	1
Cpne1	-0.13045	0.105874	1
Dhrs7	-0.13047	0.09582	1
Mospd3	-0.1305	0.028129	1
Sae1	-0.13052	0.137519	1
Eif4e2	-0.13055	0.63114	1
Lmbrd2	-0.13061	0.02419	1
Rab3gap2	-0.13066	0.357663	1
Ska3	-0.1307	0.003734	1
Dap3	-0.13083	0.070755	1
Fam98b	-0.13087	0.060178	1
Zgrf1	-0.13091	0.011713	1
Taf1b	-0.13096	0.036118	1
Itga2	-0.13104	0.002315	1
Akt1	-0.13111	0.061097	1
Cep70	-0.13124	0.023513	1
Nf1	-0.13133	0.185494	1
Dpp3	-0.1314	0.020247	1
Degs1	-0.13142	0.098151	1
Rere	-0.1315	0.294803	1
Blm	-0.13157	0.116733	1
Prkaa1	-0.13169	0.009742	1
Bscl2	-0.13178	0.011365	1

Polr2a	-0.13188	0.545346	1
Cap1	-0.13206	0.018519	1
Stxbp2	-0.13223	0.407988	1
Lrp6	-0.13239	0.569408	1
Cenpl	-0.1324	0.002693	1
Plekhg3	-0.13241	0.664521	1
Zfp212	-0.13241	0.106529	1
Samsn1	-0.1326	0.200062	1
Cdk14	-0.1329	1.36E-06	0.043883
Steap3	-0.13295	9.24E-16	2.98E-11
Nrros	-0.13295	0.099376	1
Chpt1	-0.13301	0.041156	1
Fbxl2	-0.13302	0.000107	1
Vamp7	-0.13313	0.162814	1
Rbm43	-0.13325	0.070054	1
Tmco1	-0.1334	0.092681	1
Cmtm6	-0.13342	0.123651	1
Dpm3	-0.13346	0.290637	1
Dynlt1a	-0.13346	0.008715	1
Arl4c	-0.13354	0.624785	1
Mpnd	-0.13358	0.069159	1
Ccne1	-0.13366	0.036767	1
Wiz	-0.13369	0.043539	1
Crmp1	-0.13376	0.030899	1
Plekhg2	-0.13416	0.012263	1
Scfd1	-0.13424	0.42483	1
Nelfe	-0.13441	0.2947	1
Cep55	-0.13443	0.033908	1
Cdk11b	-0.13451	0.146878	1
Chp1	-0.13455	0.049447	1
Ccng1	-0.13476	0.183866	1
Klhl28	-0.13483	0.074794	1
Recql	-0.1349	0.174975	1
Wdyhv1	-0.13492	0.044477	1
Ddx55	-0.13519	0.014889	1
Errfi1	-0.1352	0.209572	1
Becn1	-0.13524	0.078305	1
Zfp180	-0.1353	0.028735	1
Dipk1a	-0.13555	0.010313	1
Gemin7	-0.13572	0.040062	1
Mapre1	-0.13573	0.2664	1
Stk11	-0.13578	0.072121	1
P2ry10	-0.13585	0.0629	1
Ptbp2	-0.13586	0.808273	1
Cpt1a	-0.13587	0.00428	1
Tdp2	-0.13601	0.005365	1
Rad23a	-0.13613	0.125087	1
Lsm10	-0.13626	0.811051	1

Dse	-0.13637	0.253471	1
Nudt1	-0.13655	0.017417	1
D1Ertd622e	-0.13657	0.007194	1
Rbl1	-0.13671	0.03937	1
Mapk3	-0.13677	0.008966	1
Mtmr12	-0.13678	0.008071	1
Cops9	-0.13704	0.072515	1
Sgpl1	-0.13707	0.058182	1
A630001G21	-0.13709	0.277913	1
Hcls1	-0.13716	0.355311	1
Nans	-0.13717	0.140006	1
Mylip	-0.13726	0.57904	1
Upf2	-0.13734	0.048853	1
Strbp	-0.13738	0.070858	1
Taf2	-0.13739	0.014725	1
Chkb	-0.13743	0.326294	1
Ptbp1	-0.13751	0.129808	1
Tsta3	-0.13758	0.181501	1
Pdzd8	-0.13762	0.027967	1
Hmox2	-0.13768	0.320485	1
E2f7	-0.13768	0.031856	1
Atm	-0.1377	0.047976	1
Espl1	-0.13778	0.000376	1
Cmc2	-0.13779	0.160279	1
Erg28	-0.1378	0.038968	1
Tnpo1	-0.13803	0.836829	1
Ap5b1	-0.13813	0.092859	1
3110056K07I	-0.13815	0.60849	1
Smarcd2	-0.1382	0.074912	1
Pnrc1	-0.13822	0.563456	1
Trmt1l	-0.13823	0.212135	1
Rdx	-0.1383	0.024746	1
Gripap1	-0.13836	0.22393	1
Camta1	-0.1384	0.015232	1
Slc35a4	-0.13858	0.01336	1
Ptprc	-0.13864	0.01387	1
Tapbpl	-0.13873	0.100179	1
Wdr36	-0.13875	0.052216	1
Cdc45	-0.13881	0.000443	1
Anapc4	-0.13884	0.019785	1
Ermp1	-0.13887	0.187015	1
Naa15	-0.13888	0.066244	1
Fam98c	-0.1389	0.004192	1
Cops7a	-0.13892	0.15336	1
Lnpk	-0.13901	0.028103	1
Hnrnpc	-0.13905	0.117802	1
Cdca4	-0.13919	0.406161	1
Kif18a	-0.13924	0.042107	1

Lman1	-0.13943	0.495113	1
Mr1	-0.13944	0.115652	1
Ndrg3	-0.13948	0.207527	1
Lsm6	-0.13956	0.132403	1
Klhl22	-0.13956	5.24E-06	0.169093
Pimreg	-0.13958	0.076635	1
Ociad1	-0.13962	0.067875	1
Katnb1	-0.13966	0.024719	1
Gm20721	-0.13967	0.096979	1
Hist1h2bg	-0.13974	0.004079	1
Brix1	-0.13982	0.038817	1
Srrt	-0.13993	0.031453	1
Polr2c	-0.13994	0.128923	1
Ganab	-0.13997	0.131807	1
Srsf1	-0.13999	0.102046	1
Pdia4	-0.14026	0.362765	1
Chaf1b	-0.14028	0.224399	1
Kbtbd2	-0.14032	0.069536	1
Tbx21	-0.14045	0.889815	1
Tmf1	-0.14048	0.044643	1
Commd10	-0.14053	0.02043	1
Cerkl	-0.14053	0.009235	1
Rnf8	-0.14059	0.201907	1
Chst12	-0.14068	0.091223	1
Derl2	-0.14077	0.061224	1
Kpnb1	-0.14083	0.172844	1
Pura	-0.14086	0.093654	1
Pcif1	-0.14087	0.036203	1
Ptprcap	-0.14097	0.013781	1
1810026B05	-0.14098	0.247648	1
Slf1	-0.14101	0.121414	1
Atp7a	-0.14105	0.041649	1
Cep57	-0.14105	0.057738	1
Pts	-0.14107	0.632528	1
Gm48099	-0.14122	0.102708	1
Sept1	-0.14123	0.022316	1
Gdi1	-0.14126	0.065655	1
Fryl	-0.14133	0.162335	1
Prkcsh	-0.14137	0.182934	1
Pop4	-0.14137	0.078012	1
Wdr6	-0.14166	0.020251	1
Ptpra	-0.14183	0.047051	1
Hsdl2	-0.1419	0.000582	1
Gm47882	-0.14216	0.168586	1
Trip13	-0.14233	0.002223	1
Nrip1	-0.14234	0.093605	1
Cenpn	-0.1426	1.59E-05	0.512938
Cers5	-0.14265	0.489395	1

Tedc1	-0.14296	0.000811	1
Vamp8	-0.14301	0.611518	1
Ino80e	-0.14327	0.125388	1
Naa38	-0.14334	0.279003	1
Cdc6	-0.14342	1.05E-06	0.033793
Usp34	-0.14343	0.035425	1
Cenpc1	-0.14353	0.026781	1
Mto1	-0.14359	0.075345	1
Cramp1l	-0.14361	0.001081	1
D10Wsu102e	-0.14369	0.568893	1
Sarnp	-0.14383	0.091487	1
AI506816	-0.14386	0.082752	1
Gxylt1	-0.14406	0.016863	1
Aff1	-0.1441	0.965096	1
Nfe2l2	-0.14433	0.026833	1
Knop1	-0.14438	0.185427	1
Tmem37	-0.14443	0.005454	1
Kpna3	-0.14443	0.057388	1
Cog4	-0.14446	0.4582	1
Tagap	-0.14459	0.008394	1
Kif3b	-0.14463	0.017123	1
Htra2	-0.14471	0.481408	1
Ero1lb	-0.14471	0.916049	1
Hmgxb4	-0.14474	0.030762	1
Dbnl	-0.14481	0.154281	1
Atp13a3	-0.14487	0.015249	1
Ccnb1	-0.14489	0.518474	1
Pdia6	-0.1449	0.567162	1
Tln1	-0.1451	0.081807	1
Lamtor2	-0.14512	0.094683	1
Zfp956	-0.14513	0.022538	1
Arl6ip5	-0.14536	0.189082	1
Tsc22d2	-0.14538	0.677486	1
Ska2	-0.14547	0.019386	1
Ndufb2	-0.14557	0.26442	1
Zfp36	-0.14567	0.022723	1
Pan3	-0.14571	0.07133	1
Prps2	-0.14575	0.081094	1
Dedd	-0.14593	0.3088	1
Ap4s1	-0.14608	0.020886	1
Ncoa4	-0.14618	0.666057	1
Mon2	-0.14621	0.060305	1
Ing2	-0.14639	0.003266	1
Tmem161b	-0.14641	0.05028	1
Stoml2	-0.14643	0.031607	1
Son	-0.14655	0.011339	1
Phf20	-0.14656	0.043299	1
Abca7	-0.14669	0.030765	1

Nipbl	-0.14674	0.056582	1
Mcub	-0.14685	4.86E-06	0.15702
Dut	-0.14693	0.273956	1
Uba52	-0.14695	0.033165	1
Pou2f2	-0.14696	0.67013	1
Tmem71	-0.147	0.77366	1
Mdm2	-0.147	0.00636	1
Smpdl3b	-0.14704	0.035901	1
Jkamp	-0.14725	0.008517	1
Gm10130	-0.14727	0.01688	1
Mvb12a	-0.14728	0.125069	1
Gtf2e2	-0.14729	0.118428	1
Larp7	-0.14745	0.359149	1
Smarcb1	-0.14746	0.052456	1
Smim4	-0.14749	0.123497	1
Fam83d	-0.1477	0.003116	1
Cd82	-0.1479	0.142443	1
1810009A15I	-0.14796	0.081045	1
Kctd20	-0.14799	0.014268	1
Ly86	-0.14804	0.139417	1
Hsd11b1	-0.14813	0.313872	1
Gsn	-0.14815	0.596411	1
Fnta	-0.14815	0.289165	1
Ankra2	-0.14818	0.171592	1
Cdc37	-0.14819	0.005087	1
Rdm1	-0.14822	0.011732	1
Flii	-0.14822	0.033304	1
Adcy3	-0.14827	0.000865	1
Pdlim5	-0.14835	0.175951	1
H2-DMa	-0.14843	0.31706	1
Anapc15	-0.14844	0.249056	1
Vopp1	-0.14855	0.2935	1
Fip1l1	-0.14866	0.055621	1
Raf1	-0.14867	0.094603	1
Ica1	-0.14868	0.026095	1
Aaas	-0.14874	0.046753	1
Erlec1	-0.14874	0.755399	1
Tbc1d10c	-0.1488	0.049758	1
Nod1	-0.14901	0.008382	1
Toe1	-0.14904	0.212944	1
Urod	-0.14917	0.073638	1
Rab11a	-0.1493	0.060734	1
Cdca5	-0.14959	0.007299	1
4930402H24	-0.1496	0.145836	1
Was	-0.14973	0.028451	1
Csrp1	-0.14978	0.040861	1
Rfx3	-0.14979	0.006736	1
Gga3	-0.14989	0.010187	1

Tspan32	-0.1499	0.142906	1
Snrk	-0.15001	0.022355	1
Psmd14	-0.15017	0.150627	1
Sept7	-0.15026	0.261287	1
Usp21	-0.1503	0.013669	1
Tle4	-0.15031	0.106575	1
D130009I18F	-0.15045	0.000601	1
Tbck	-0.15058	0.016813	1
Tatdn1	-0.15062	0.312461	1
Tomm6	-0.15066	0.044511	1
Timp2	-0.15076	0.998266	1
Calr	-0.15081	0.300042	1
Prpf38a	-0.15087	0.019288	1
Itga6	-0.15117	0.004405	1
Hmgb3	-0.15117	0.015542	1
Sorbs1	-0.15124	0.006889	1
Opa1	-0.15132	0.006694	1
Ipo9	-0.15139	0.037241	1
Cdk5rap2	-0.15165	0.208001	1
Brca1	-0.15166	0.038335	1
Mypopos	-0.15171	2.15E-05	0.692696
Haus4	-0.15172	0.082268	1
Zranb2	-0.15188	0.248332	1
Fanci	-0.15195	0.001403	1
Kdelr2	-0.15197	0.429997	1
Atp5mpl	-0.152	0.383416	1
Dpp9	-0.15201	0.031876	1
Prkd2	-0.15209	0.22928	1
Stub1	-0.15213	0.137348	1
Txndc16	-0.15217	0.00672	1
Cblb	-0.15217	0.045471	1
Lrmda	-0.15257	0.005175	1
Pxmp4	-0.15257	0.31996	1
Atad1	-0.15269	0.113039	1
Nup85	-0.15269	0.095154	1
Nedd1	-0.15274	0.033069	1
Slu7	-0.15292	0.000938	1
Rnf168	-0.15303	0.023268	1
Cep83	-0.15324	0.020125	1
Mmgt2	-0.15329	0.164487	1
Oat	-0.15331	0.949561	1
Selenof	-0.15331	0.079466	1
Cercam	-0.15342	0.000673	1
Arhgap19	-0.15357	0.000201	1
Necap2	-0.15358	0.137051	1
Wdr5	-0.15374	0.199159	1
Rapgef1	-0.15377	0.139927	1
Ramac	-0.15378	0.02468	1

Khsrp	-0.15385	0.006293	1
Golim4	-0.15397	0.512428	1
mt-Cytb	-0.15402	0.117003	1
Impdh1	-0.15416	0.072441	1
Prr14l	-0.15417	0.133099	1
Hmgcr	-0.15425	0.059771	1
Tshz1	-0.15432	0.019985	1
Tlk1	-0.15443	0.019062	1
Atp6v0b	-0.15451	0.399862	1
Eif2b4	-0.15482	0.012561	1
Wdr62	-0.15486	0.000133	1
Pds5b	-0.15496	0.041072	1
Syne1	-0.15496	0.307282	1
Zfp652	-0.15516	0.166506	1
Tbc1d1	-0.15543	0.001477	1
Zfp961	-0.1555	6.21E-05	1
Brca2	-0.15556	0.023752	1
Arhgap45	-0.15591	0.097864	1
Pigp	-0.15596	0.052211	1
Zc3h18	-0.15598	0.199411	1
Capns1	-0.15608	0.029876	1
Cnot10	-0.1561	0.107386	1
Gpr34	-0.15628	0.163274	1
Srprb	-0.1563	0.291535	1
Lrpap1	-0.15633	0.426281	1
Lrrc57	-0.15635	0.076446	1
Lsm2	-0.15655	0.24162	1
Tbc1d10a	-0.15662	0.013087	1
Pdik1l	-0.15668	0.01682	1
Akap8l	-0.15679	0.795156	1
Stat6	-0.1568	0.47905	1
Rp2	-0.15697	0.00185	1
Eif4e	-0.15712	0.020044	1
Rnaset2a	-0.15734	0.040718	1
Tra2b	-0.15748	0.144114	1
Spg20	-0.15754	0.773191	1
Gm31597	-0.15759	0.003473	1
Cuedc2	-0.15777	0.069789	1
Sp100	-0.15787	0.068813	1
Mkln1	-0.15793	0.055208	1
Nup133	-0.15809	0.001842	1
Il31ra	-0.15817	0.009997	1
Elob	-0.15839	0.065789	1
AI467606	-0.15851	0.067614	1
Bag6	-0.15863	0.023793	1
Nrbp1	-0.1588	0.185477	1
Mindy2	-0.15911	0.34999	1
Ap2s1	-0.15918	0.147164	1

Nup88	-0.15934	0.018762	1
Ccar1	-0.15947	0.169764	1
Ccser2	-0.15947	0.03093	1
Dock2	-0.15947	0.04034	1
Acad9	-0.15951	0.008235	1
Polr2k	-0.15953	0.023439	1
Ncoa3	-0.15957	0.074225	1
Ppm1g	-0.1597	0.143715	1
Rptor	-0.15987	0.033048	1
Haus3	-0.15993	0.022466	1
Ttc17	-0.16014	0.035327	1
Nhlrc2	-0.16017	0.11259	1
Snrpc	-0.16025	0.027167	1
Scfd2	-0.16032	0.166846	1
Sfxn1	-0.16038	0.116229	1
Slc25a12	-0.16041	0.023443	1
Gm26827	-0.16054	0.005439	1
Sun1	-0.16063	0.002249	1
Bckdk	-0.16081	0.345133	1
Ncaph2	-0.16113	0.239931	1
Hnrnpul1	-0.16126	0.095874	1
Ggnbp2	-0.16133	0.046307	1
Fgfr1op2	-0.16148	0.011867	1
Gtf3c6	-0.1615	0.413883	1
Irf2bpl	-0.16164	0.374548	1
Kmt5a	-0.16165	0.075223	1
Sult2b1	-0.16185	0.00881	1
Pias3	-0.16186	0.157167	1
1500009L16I	-0.16206	0.00596	1
Mtrex	-0.16216	0.016868	1
Poldip3	-0.16221	0.088293	1
Rhoq	-0.16232	0.002703	1
Dpysl2	-0.16245	0.021824	1
Stim2	-0.16281	0.042106	1
Fam149b	-0.16301	0.239954	1
Trappc1	-0.16307	0.03402	1
Nup93	-0.16322	0.013806	1
Pde4dip	-0.16351	0.080792	1
Atf1	-0.16359	0.083901	1
Brip1	-0.16362	0.017525	1
Ppp2r1a	-0.16362	0.016539	1
Srbd1	-0.16364	0.012992	1
4930444A19I	-0.16376	0.015825	1
Kcnk5	-0.16411	0.004397	1
Prim2	-0.1643	0.017882	1
Uba6	-0.16443	0.018151	1
Pgap1	-0.1645	0.273927	1
Rgs14	-0.1645	0.08968	1

Lax1	-0.16454	0.2098	1
Ndufc1	-0.1647	0.036047	1
Psph	-0.16471	0.066153	1
Senp7	-0.16476	0.068406	1
Pdlim7	-0.16478	0.121536	1
Pum1	-0.16485	0.011264	1
Pa2g4	-0.16492	0.07707	1
Gm10131	-0.16574	0.324464	1
Agpat5	-0.16583	0.006798	1
Actr1a	-0.16597	0.073685	1
Tmem163	-0.16602	0.210652	1
Kif14	-0.16607	0.000449	1
Atp11b	-0.16617	0.306691	1
Nek2	-0.16629	0.002317	1
Baz1a	-0.16633	0.106713	1
Cenpm	-0.16639	0.000122	1
Pds5a	-0.1665	0.015377	1
Klc1	-0.16651	0.018614	1
Ermard	-0.16666	0.012712	1
Commd3	-0.1668	0.064354	1
Rbm10	-0.16703	0.003413	1
Dsn1	-0.16703	1.95E-05	0.62922
Atf7ip	-0.16727	0.130674	1
Ist1	-0.1673	0.013985	1
Mrpl4	-0.1673	0.230706	1
Fam76b	-0.16749	0.01891	1
Herc1	-0.1676	0.031979	1
Chchd1	-0.16767	0.00619	1
Efcab11	-0.16771	0.003016	1
Dda1	-0.16792	0.016252	1
Lypla2	-0.16833	0.018797	1
Edf1	-0.16835	0.029531	1
Zpbp	-0.16849	0.019912	1
Fcho2	-0.16876	0.042917	1
Stn1	-0.16881	0.005203	1
Cyfip2	-0.16885	0.204491	1
Kdelr1	-0.16886	0.027991	1
Myo18a	-0.16892	0.00712	1
Ckap2	-0.16911	0.026447	1
Arl6ip6	-0.16919	0.010462	1
Spcs3	-0.16922	0.036694	1
Lman2	-0.16927	0.258952	1
Psenen	-0.16948	0.029703	1
Qk	-0.16957	0.157397	1
Plekhj1	-0.1697	0.008722	1
Spag5	-0.16997	0.016908	1
Elf1	-0.17015	0.02214	1
Npc1	-0.17016	0.141957	1

Lrwd1	-0.17019	0.168277	1
Ifnar2	-0.17046	0.086768	1
Rrbp1	-0.17052	0.399771	1
Net1	-0.17067	0.001952	1
Abcg3	-0.17071	0.014975	1
Klrd1	-0.17094	0.83378	1
Vps13c	-0.17095	0.021641	1
Prkacb	-0.17115	0.253225	1
Cnp	-0.17119	0.715421	1
Tcerg1	-0.17125	0.084626	1
Sumo2	-0.17174	0.111994	1
BC035044	-0.17177	0.000674	1
Hpf1	-0.17183	0.276597	1
Hes6	-0.17183	0.007433	1
Bad	-0.17199	0.040798	1
Pogk	-0.17208	0.00029	1
Cdc16	-0.17236	0.010418	1
Brip1os	-0.17238	0.073886	1
Itm2a	-0.17239	0.433016	1
Cuta	-0.17257	0.024365	1
Med12	-0.17263	0.103055	1
Btf3l4	-0.17266	3.81E-05	1
Eif2s1	-0.1727	0.069555	1
Hat1	-0.17282	0.141086	1
Def6	-0.17295	0.120087	1
Supt16	-0.17307	0.15014	1
Ncf1	-0.17314	0.655515	1
Ik	-0.17322	0.126734	1
Pabpn1	-0.17326	0.10249	1
Hspd1	-0.17334	0.350074	1
1700001O22	-0.17351	1.11E-13	3.57E-09
Mast2	-0.17352	0.039363	1
Hmgcs1	-0.17352	0.103866	1
Rfc4	-0.17362	0.052073	1
Ewsr1	-0.17369	0.015315	1
Knstrn	-0.17377	0.006063	1
Phactr4	-0.1738	0.307713	1
Arhgap30	-0.17386	0.052011	1
Gnai2	-0.17422	0.002371	1
Lonp2	-0.17447	0.077734	1
Hist1h2be	-0.1746	0.063013	1
Trim8	-0.17475	0.242291	1
Prpf3	-0.17518	0.006349	1
Gtf2i	-0.17519	0.033397	1
Snx3	-0.1755	0.036977	1
Psme3	-0.17564	0.024798	1
Tle3	-0.17566	0.00884	1
Slc29a3	-0.17603	0.601969	1

Aqr	-0.17629	0.029548	1
Syncrip	-0.17632	0.126919	1
Stk38	-0.17647	0.024312	1
Dpy30	-0.17662	0.206312	1
Carhsp1	-0.1769	0.02969	1
Abracl	-0.177	0.021788	1
Cnep1r1	-0.1771	0.027039	1
Serp1	-0.17717	0.776485	1
Sdhb	-0.1775	0.409549	1
Top3b	-0.17761	0.0129	1
Ppp1cb	-0.17766	0.008587	1
Sf1	-0.17782	0.048937	1
Strn	-0.17802	0.001547	1
Camta2	-0.17821	0.070673	1
Cdk2	-0.17843	0.000916	1
Sema4a	-0.17847	0.056924	1
Pon2	-0.1785	0.307643	1
Slamf6	-0.17857	0.000218	1
Ctdsp1	-0.17857	0.000287	1
Phf14	-0.17884	0.055239	1
Cox20	-0.17895	0.064994	1
Phrf1	-0.1793	0.02745	1
Lamtor3	-0.17948	0.008502	1
Msn	-0.17975	0.002127	1
Rnf216	-0.17991	0.001769	1
Svil	-0.18	0.068908	1
Ppp1r15b	-0.18015	0.026967	1
Pkmyt1	-0.18023	0.00232	1
Copb2	-0.18051	0.065202	1
Plcl2	-0.18061	0.230928	1
Lpcat3	-0.18062	0.221417	1
Commd1	-0.18067	0.135989	1
Wdhd1	-0.18084	0.012386	1
Dync1i2	-0.18087	0.012039	1
Prrc2a	-0.18091	0.174699	1
Kctd10	-0.18101	0.083315	1
Cpsf7	-0.18101	0.045276	1
Sap18	-0.1811	0.01146	1
Ubl3	-0.1811	0.122785	1
Ldlrap1	-0.18113	2.57E-06	0.082935
Spen	-0.18113	0.127632	1
Smarcc2	-0.18118	0.164458	1
Ap3b1	-0.18134	0.079273	1
Eny2	-0.18153	0.743636	1
Psmc4	-0.18194	0.018556	1
Hjurp	-0.18198	0.03992	1
Ticrr	-0.18259	0.135325	1
Mrpl18	-0.18268	0.111519	1

Abcb9	-0.18269	0.022953	1
Lfng	-0.1828	0.22048	1
Eif3l	-0.18292	0.048003	1
Tsc2	-0.18313	0.000139	1
Dido1	-0.18313	0.009446	1
Hdhd2	-0.18338	0.037463	1
Klrc2	-0.18355	0.2162	1
Gm38394	-0.18378	0.003106	1
Xrcc4	-0.18415	0.336227	1
Adrb1	-0.18416	1.69E-05	0.545678
Zfhx3	-0.18442	9.4E-06	0.303411
Cyb561a3	-0.18465	0.032208	1
Rnpep	-0.18485	0.076327	1
Uckl1	-0.18511	0.02235	1
Ctse	-0.18517	0.306787	1
Arpc5l	-0.18534	0.310353	1
Neurl3	-0.18538	0.135376	1
Nrf1	-0.18553	0.002947	1
Ninj1	-0.18558	0.457502	1
Sf3b1	-0.18561	0.001837	1
Tbxa2r	-0.18561	0.077241	1
Nt5c	-0.18582	0.199062	1
Mea1	-0.18582	0.027122	1
Tgfb1	-0.18628	0.01638	1
Sat1	-0.18638	0.522571	1
Pacsin2	-0.18665	0.078754	1
Igfbp7	-0.18671	7.68E-05	1
Pde1c	-0.18679	1.07E-10	3.46E-06
Tbl2	-0.18679	0.001027	1
I730030J21R	-0.18697	0.026364	1
Dynlt1f	-0.18709	0.022023	1
Ndufb1-ps	-0.18723	0.012465	1
Prkcd	-0.18725	0.177745	1
Ska1	-0.18725	0.003043	1
Adrm1	-0.18747	0.014011	1
Rnaseh2c	-0.18751	0.109852	1
Zfx	-0.18751	0.006436	1
Brk1	-0.18761	0.012227	1
Rpn2	-0.18765	0.039001	1
Ttc3	-0.18775	0.059931	1
Safb	-0.18789	0.003572	1
Cbl	-0.18801	0.016757	1
Spn	-0.18874	0.056583	1
Myo1e	-0.18889	0.145952	1
Hist1h2bh	-0.18908	0.000405	1
Osbpl8	-0.1891	0.008716	1
C2cd5	-0.18915	0.005409	1
Phtf2	-0.18947	0.000593	1

Eps15	-0.18958	0.028672	1
Filip1l	-0.18962	0.238891	1
Faf1	-0.18963	0.000751	1
Cep350	-0.19011	0.083663	1
Tma7	-0.19018	0.022034	1
Map4k1	-0.19045	0.027258	1
Ppp1ca	-0.19049	0.000956	1
Ube2g2	-0.19052	0.196115	1
Msh2	-0.19056	0.07391	1
Etfb	-0.19073	0.024185	1
Calcrl	-0.19076	0.27724	1
Hdlbp	-0.19086	0.130413	1
Sin3a	-0.19111	0.023844	1
Bub1	-0.19112	0.000243	1
Prr5	-0.19144	0.553406	1
Osbpl9	-0.19144	0.012151	1
Fam89b	-0.19152	0.091926	1
Dgka	-0.19153	0.057249	1
Snx2	-0.19157	0.916447	1
Nde1	-0.19166	0.002295	1
Adam17	-0.19183	0.010555	1
Ifnar1	-0.19211	0.026893	1
Dnaaf5	-0.19248	0.000222	1
Eif3a	-0.19249	0.053072	1
Timm17b	-0.19252	0.082194	1
Man1c1	-0.19258	1.13E-10	3.65E-06
Acot8	-0.1926	0.001501	1
Ano6	-0.19268	0.02246	1
Hnrnpul2	-0.19285	0.016589	1
Frs2	-0.19286	0.054759	1
Ppp1r18	-0.19299	0.024687	1
Ddx17	-0.19302	0.028421	1
Bsg	-0.1931	0.902873	1
Spred2	-0.19328	0.071432	1
Srek1	-0.1933	0.017387	1
Fcho1	-0.19352	0.019792	1
Ddx43	-0.19355	2.74E-13	8.84E-09
Rhog	-0.1937	0.05405	1
Hook3	-0.19373	0.023529	1
Srgap2	-0.19414	0.362162	1
Atp5b	-0.19451	0.005714	1
Exoc2	-0.19456	0.023244	1
Themis2	-0.19465	0.006102	1
Plekha1	-0.1948	0.000165	1
Eif4g1	-0.19499	0.030108	1
Prr11	-0.19499	0.003133	1
Laptm4a	-0.19535	0.451104	1
Ssr2	-0.19535	0.035523	1

Eri1	-0.19541	0.013913	1
Atp6v1c1	-0.19564	0.012037	1
Sft2d1	-0.19592	0.016003	1
Rexo1	-0.19608	0.010293	1
Pmm1	-0.19643	0.07156	1
Prelid3b	-0.1967	0.08211	1
Dapk2	-0.19676	0.010555	1
Fam13b	-0.1971	0.004556	1
Rab3a	-0.19718	0.004066	1
Ap2b1	-0.19771	0.033826	1
Pdlim1	-0.19803	0.011781	1
Ap2m1	-0.19803	0.075408	1
Cc2d1a	-0.19822	0.003585	1
Heatr6	-0.1985	0.077619	1
Dnajc7	-0.1985	0.8015	1
Meaf6	-0.19865	0.002685	1
Tmtc2	-0.19867	0.001275	1
Klf12	-0.19872	1.55E-05	0.49974
Vps29	-0.19875	0.040376	1
Babam1	-0.19878	0.009735	1
Rbl2	-0.19879	0.025684	1
Cs	-0.19884	0.132889	1
Selplg	-0.19888	0.042982	1
Bzw1	-0.19888	0.004774	1
Ndc80	-0.19947	0.032204	1
Ncapg2	-0.19979	0.008814	1
Gucd1	-0.1999	0.000354	1
Kmt2e	-0.20014	0.020615	1
Eps15l1	-0.20066	0.157216	1
Ddb1	-0.20074	0.019798	1
Dock5	-0.20092	0.010698	1
Zmiz2	-0.20095	0.018767	1
Pias1	-0.20098	0.129636	1
Trub2	-0.20141	0.001254	1
Pigc	-0.2015	0.021892	1
Actn1	-0.20162	0.000806	1
B3gnt5	-0.20184	0.012974	1
Ppm1j	-0.20213	0.014549	1
Dok2	-0.20263	0.206254	1
Tnks2	-0.20269	0.039815	1
Smarcc1	-0.20277	0.026788	1
Gm36738	-0.20376	0.148872	1
Gnb1	-0.20386	0.141459	1
Golga4	-0.20398	0.003064	1
Park7	-0.20431	0.015697	1
Cct8	-0.20433	0.076853	1
Prkab1	-0.20442	0.039158	1
Ugcg	-0.20457	0.005039	1

Ssb	-0.2047	0.013286	1
Dbf4	-0.20475	0.137961	1
Cdt1	-0.20485	0.0021	1
Ahcy	-0.2049	0.222159	1
AB124611	-0.20526	0.006756	1
Sgo1	-0.20539	0.000367	1
Rcc1	-0.20564	0.011646	1
Mrps21	-0.2061	0.014748	1
Numa1	-0.20641	0.030495	1
Upk1a	-0.20648	1.66E-05	0.53462
Mid1ip1	-0.20655	0.125817	1
Parpbp	-0.2068	0.105482	1
Cdkn2aipnl	-0.20691	0.005368	1
Zcrb1	-0.20699	0.453061	1
Hnrnpl	-0.20714	0.008225	1
Ncbp1	-0.20717	0.063027	1
Rock2	-0.20728	0.004127	1
Gm37401	-0.20739	0.000381	1
Slc44a2	-0.20742	0.010547	1
Napsa	-0.2078	0.697295	1
Ncstn	-0.20792	0.115881	1
Hist1h2ap	-0.20812	0.000272	1
Dock11	-0.20819	0.014634	1
Srsf7	-0.20824	0.068522	1
Tbca	-0.20852	0.009428	1
Kctd18	-0.20857	0.024829	1
Nck1	-0.20868	0.016298	1
Psmb6	-0.20868	0.008235	1
Canx	-0.20905	0.230625	1
Sf3b2	-0.20927	0.010039	1
Map3k3	-0.20954	0.130827	1
Capza1	-0.20973	0.034287	1
Shc1	-0.2098	0.061558	1
Gapvd1	-0.21017	0.046245	1
Cd38	-0.21019	0.00055	1
Rps27l	-0.21026	0.154467	1
Casp3	-0.21055	0.034546	1
Pfdn6	-0.21071	0.02257	1
Sppl2a	-0.21144	0.039492	1
Arpc4	-0.2116	0.000805	1
Psen2	-0.21198	0.071428	1
Nup62	-0.212	0.008501	1
Ppp2r1b	-0.21219	0.007851	1
Dtl	-0.21222	0.007371	1
Ndufa3	-0.21273	0.022657	1
Anapc11	-0.21286	0.006886	1
Polr3c	-0.21287	0.550373	1
Kif20a	-0.21299	0.002256	1

Tmpo	-0.21315	0.237426	1
Dusp22	-0.21317	0.312472	1
Rexo5	-0.21359	4.71E-05	1
Stag2	-0.2136	0.020721	1
Tinf2	-0.21363	0.000373	1
Pgm2	-0.21368	0.018744	1
1700037C18I	-0.21407	0.103672	1
Angel2	-0.21409	0.155621	1
Ppil1	-0.2141	0.064589	1
Klf10	-0.21425	0.000748	1
Ak3	-0.21434	0.001229	1
Myadm	-0.21454	7.78E-09	0.000251
Trim37	-0.2147	0.010325	1
Plk4	-0.21493	0.006027	1
Kifc1	-0.21518	0.107412	1
Arhgap4	-0.21529	0.070525	1
Atad5	-0.2155	0.0027	1
Nsd2	-0.21553	0.014337	1
Fzr1	-0.216	0.176775	1
Stx7	-0.21602	0.007665	1
Il10rb	-0.21616	0.00297	1
Stil	-0.21647	1.84E-06	0.059302
Otulinl	-0.2166	0.005711	1
Xrn1	-0.21662	0.023966	1
Hsd17b4	-0.21688	0.124264	1
Dlgap5	-0.21694	0.006389	1
Wdr76	-0.21698	0.00021	1
Dtymk	-0.21719	0.015175	1
Map3k11	-0.21737	0.000393	1
Myb	-0.2179	0.004231	1
Fxn	-0.2179	0.008015	1
Nln	-0.21803	0.229358	1
Cbx5	-0.21833	0.028677	1
Ppia	-0.21846	0.00347	1
Myl12a	-0.21872	0.003137	1
Fuca1	-0.21947	0.137108	1
Pfdn1	-0.2196	0.020292	1
Dynll1	-0.21964	0.146493	1
Map4	-0.21969	0.005376	1
Zbtb20	-0.21977	0.720709	1
Pdap1	-0.22	0.058239	1
Cdk4	-0.22002	0.083479	1
Psap	-0.2202	0.005429	1
Xiap	-0.22042	0.002839	1
Cwc15	-0.22061	0.07599	1
Atg4d	-0.22081	0.029264	1
1500004A13I	-0.22091	0.004386	1
Calm3	-0.22103	0.020183	1

Tfeb	-0.22124	0.227124	1
Rpa2	-0.22151	0.100794	1
Dhfr	-0.22178	0.002375	1
Gramd4	-0.22196	4.72E-05	1
Esd	-0.22199	0.00993	1
Ascc2	-0.22211	0.006495	1
Myo5a	-0.22215	0.043615	1
Fchsd2	-0.22225	0.580755	1
Epb41l2	-0.22235	0.181378	1
Slamf7	-0.22238	0.042412	1
Rab11b	-0.22247	0.053515	1
Cse1l	-0.22261	0.240716	1
Psmb7	-0.22278	0.223398	1
Cenph	-0.22288	9.79E-06	0.316188
Cpm	-0.22299	0.000563	1
Ssna1	-0.22306	0.027844	1
Fads1	-0.22357	3.19E-06	0.102835
Serinc3	-0.22399	0.381135	1
Fen1	-0.22402	0.062136	1
Tmcc3	-0.2241	0.001786	1
E2f8	-0.22424	0.006024	1
Por	-0.22428	0.732876	1
Alms1	-0.2243	1.72E-05	0.554358
Rbmx	-0.22495	0.092556	1
Tgfbr1	-0.22511	0.043803	1
Rad51ap1	-0.22588	7.46E-06	0.240936
Kif2c	-0.22604	2.17E-05	0.70135
Rtn4rl1	-0.22631	0.000627	1
Pfdn2	-0.22637	0.007104	1
Slc35b1	-0.22649	0.102945	1
Borcs7	-0.2267	0.000684	1
Virma	-0.22685	0.001232	1
Cox5b	-0.22686	0.026009	1
Ets1	-0.22736	0.004183	1
Irf2	-0.22738	0.032394	1
Ttc7	-0.2277	0.000796	1
B020010K11I	-0.22792	0.000315	1
Rap1a	-0.22796	0.00677	1
Atp5k	-0.22809	0.004341	1
Rbm22	-0.22823	0.039857	1
Zcchc18	-0.22827	3.82E-09	0.000123
Glud1	-0.2283	0.004961	1
Arhgap1	-0.22844	9.5E-05	1
Cers2	-0.22851	0.016308	1
Dbi	-0.22853	0.003323	1
Reep4	-0.22859	0.001353	1
Bub1b	-0.22872	0.003175	1
Dhx32	-0.22927	0.000577	1

Nr4a2	-0.2294	0.302245	1
Ccndbp1	-0.22953	0.00948	1
Hnrnpd	-0.22955	0.004371	1
Lta4h	-0.2299	0.07783	1
Rif1	-0.23012	0.097213	1
Fgr	-0.23052	8.1E-08	0.002615
Ube2d2a	-0.23071	0.000616	1
Nudt21	-0.23094	0.005422	1
Dync1li1	-0.23105	0.001035	1
Dnm2	-0.23111	0.006812	1
Selenoh	-0.23129	0.086981	1
Slc9a3r1	-0.23141	0.005933	1
Nab2	-0.23172	0.066714	1
Slc39a10	-0.23218	0.000223	1
Cnot8	-0.23233	0.052737	1
Vps35l	-0.23236	0.038483	1
Ap1s2	-0.23248	0.000928	1
Tspan13	-0.23308	0.391561	1
Hdgf	-0.23346	0.158689	1
Hist1h2bn	-0.23348	0.000779	1
Arid1a	-0.23351	0.004803	1
Nmral1	-0.23369	2.95E-05	0.950856
Ube2t	-0.23388	0.000778	1
Glyr1	-0.23392	0.025589	1
Ywhaz	-0.23463	0.000368	1
Ube2a	-0.23478	0.003816	1
Nono	-0.23489	0.007095	1
Hdac7	-0.23503	0.000538	1
Sigmar1	-0.23531	0.020548	1
Lpcat4	-0.23536	0.000749	1
Bub3	-0.23579	0.469927	1
Actn4	-0.23638	0.023157	1
C1qtnf6	-0.23645	0.001787	1
Coro1a	-0.23663	3.71E-05	1
Haao	-0.23679	0.001418	1
Usp19	-0.23709	0.022178	1
Haspin	-0.23734	0.000346	1
Laptm5	-0.23735	0.000486	1
Ccdc69	-0.2375	0.001649	1
Runx3	-0.23761	0.034583	1
Gse1	-0.23769	0.003601	1
Snx14	-0.23776	0.001716	1
Dlst	-0.23791	0.009781	1
Msl3	-0.23803	0.019322	1
Tax1bp3	-0.23822	0.00179	1
Srsf3	-0.23844	0.068628	1
Mbd6	-0.23876	0.393456	1
Prpf4b	-0.23877	0.0057	1

Eno3	-0.23889	0.000577	1
Suv39h1	-0.23932	0.000982	1
Afmid	-0.23949	0.003375	1
Sdf2l1	-0.23968	0.107795	1
5830432E09I	-0.23969	1.2E-06	0.038898
Nap1l1	-0.23978	0.01968	1
Cdkn2a	-0.23978	3.1E-05	1
Hist1h3g	-0.23991	2.1E-05	0.679424
Agfg2	-0.24017	0.000335	1
Tmem131l	-0.24056	0.008284	1
Hist1h3f	-0.24122	0.000951	1
Pnkd	-0.24127	0.002509	1
Med10	-0.24152	0.077682	1
Pbk	-0.24173	0.0001	1
Magt1	-0.24174	0.002096	1
Hist1h2bj	-0.24175	1.04E-06	0.033731
Pja2	-0.2418	0.002498	1
Mastl	-0.24208	0.000171	1
Pttg1	-0.24227	0.012986	1
Elf4	-0.24259	0.003465	1
Dap	-0.24262	0.020117	1
Gapdh	-0.24276	0.002046	1
Pole2	-0.24292	1.36E-07	0.00439
Myh9	-0.24316	0.003538	1
Stk32c	-0.24328	0.000179	1
Akt2	-0.24333	0.025059	1
Itpr3	-0.24359	0.005947	1
Hist1h4h	-0.24372	0.000725	1
Emc2	-0.2439	0.009889	1
Rnf130	-0.24409	5.03E-08	0.001625
Rcbtb2	-0.24445	0.002932	1
Sh3bp1	-0.24504	0.019954	1
Ogdh	-0.24517	0.000573	1
Odf2	-0.24612	0.000668	1
Eif4g2	-0.24664	0.000193	1
Ncf2	-0.24664	9.02E-05	1
Plxdc1	-0.24701	5.63E-11	1.82E-06
Hmgn5	-0.24719	0.000605	1
Wdr1	-0.24743	0.000403	1
Capn1	-0.24748	0.010021	1
Mpc2	-0.24779	0.4567	1
Cyba	-0.24804	1.44E-05	0.463839
Hist1h2an	-0.24805	8E-06	0.258132
Elmo2	-0.24817	0.000462	1
Brd8	-0.24828	0.032829	1
Spc25	-0.24874	1.13E-05	0.363396
Atp1b3	-0.24906	0.000208	1
Dleu2	-0.24914	0.003047	1

Kremen2	-0.24949	0.005262	1
Arid3a	-0.24963	0.236817	1
Banf1	-0.24996	0.119754	1
Fignl1	-0.25039	5.89E-06	0.190102
Kif20b	-0.25086	0.104254	1
Unc13d	-0.25122	0.008452	1
Icam2	-0.25136	5.77E-06	0.186374
Cep128	-0.25147	0.003909	1
Pdgfb	-0.25205	2.69E-09	8.69E-05
Slc39a6	-0.25248	0.003512	1
Rhoa	-0.25273	3.48E-05	1
Sipa1	-0.25355	0.005294	1
Ubl4a	-0.25362	0.017072	1
mt-Nd4	-0.25373	0.012022	1
Ssrp1	-0.25411	0.005223	1
Snrpg	-0.25468	0.000532	1
Selenop	-0.25491	0.809854	1
Mxd3	-0.25512	1.95E-06	0.062947
Copa	-0.25527	0.001268	1
Wasf2	-0.25537	0.000613	1
Taf15	-0.25619	0.002083	1
1700025G04	-0.25656	4.9E-07	0.015826
Tcf4	-0.25712	0.72586	1
Flnb	-0.2575	2.17E-05	0.699692
Cenpp	-0.25761	0.019295	1
Gnb4	-0.25767	1.03E-08	0.000334
Atp5h	-0.2581	0.000913	1
Slc37a2	-0.25854	0.001411	1
Hint1	-0.25888	0.002469	1
Cldnd1	-0.25895	0.001813	1
Atp2c1	-0.25916	0.000245	1
Prkag1	-0.2592	0.002401	1
Ube2m	-0.25967	0.002424	1
Plk1	-0.25976	0.024577	1
Gsk3b	-0.26101	0.001892	1
Zdhhc14	-0.26122	2.03E-08	0.000655
Hspa14	-0.26186	8.43E-05	1
Tecr	-0.26189	0.002715	1
Ppp2r5c	-0.26193	0.008123	1
R3hdm1	-0.26211	0.027972	1
Esco2	-0.26253	0.000777	1
Ifitm10	-0.26258	0.03285	1
Slc35c1	-0.26264	0.000109	1
Snrpd1	-0.26271	0.007949	1
Ncor1	-0.26278	0.000446	1
Memo1	-0.26344	0.003369	1
Twf2	-0.2635	0.058837	1
Tec	-0.26364	0.00116	1

Baz2a	-0.26369	0.010397	1
Vcl	-0.26376	0.000201	1
Chit1	-0.26387	4.92E-12	1.59E-07
Prim1	-0.26406	0.000325	1
Cbx3	-0.26426	0.033723	1
Plcb2	-0.26477	0.001749	1
Cenpk	-0.26488	1.38E-05	0.446533
Rbbp8	-0.26505	0.000107	1
Serpini1	-0.26556	2.96E-18	9.56E-14
Acss1	-0.26563	0.028292	1
Slc14a1	-0.26584	0.001561	1
Prf1	-0.26602	0.000494	1
Mcm7	-0.26627	0.001774	1
Atp6v1g1	-0.26644	0.027215	1
Pafah1b1	-0.26644	0.001838	1
Cdca7l	-0.26688	0.014542	1
Gpd1l	-0.26691	9.2E-05	1
Sfpq	-0.26696	0.068952	1
Vezf1	-0.26698	0.001729	1
Ctsc	-0.26815	0.000516	1
Mcee	-0.26874	0.042521	1
Slc4a2	-0.26899	0.091497	1
Nasp	-0.26963	0.005037	1
Sbf1	-0.27006	0.001169	1
Gfod1	-0.27008	0.000216	1
Ywhab	-0.2701	0.000498	1
Cenpx	-0.27129	0.045527	1
Tmem258	-0.2717	0.000485	1
H2-Oa	-0.27202	0.043013	1
Bcl2l13	-0.27208	3.22E-05	1
Ccng2	-0.27225	0.010554	1
Nfu1	-0.27234	0.069677	1
St3gal4	-0.27257	0.003869	1
1810037I17F	-0.27271	0.009711	1
Pole	-0.27335	0.000165	1
Rad51	-0.27389	0.000452	1
Tjp2	-0.27399	2.73E-05	0.879949
Rras2	-0.27412	1.98E-06	0.063986
Ubash3b	-0.27413	0.025105	1
Luc7l3	-0.2757	0.000447	1
Ccr5	-0.2761	0.321462	1
Ctnnb1	-0.27641	0.000214	1
Cdkn2c	-0.2766	8.77E-05	1
Scarb2	-0.27683	0.019766	1
Cast	-0.27684	0.001448	1
Tmsb4x	-0.2769	9.83E-08	0.003172
Arhgap17	-0.27727	0.004045	1
Sgo2a	-0.27733	0.000286	1

Hist1h2bc	-0.27795	0.088428	1
Aip	-0.27796	0.012834	1
Xrn2	-0.27824	0.000606	1
Ap3s1	-0.27826	0.004848	1
Camk2n1	-0.27845	0.001479	1
Tmod3	-0.27847	0.001566	1
Depdc1a	-0.27913	0.001119	1
Cenpw	-0.27963	0.000675	1
Ppp1r35	-0.27971	0.000752	1
Macf1	-0.27986	0.014255	1
Rps6ka1	-0.27993	0.003024	1
Alyref	-0.28043	0.005326	1
Golm1	-0.28074	0.006628	1
Cat	-0.28116	0.01826	1
Rbm34	-0.28134	0.720701	1
Dhx9	-0.28149	0.010734	1
Ndufs2	-0.28159	8.95E-05	1
Pkp4	-0.28192	2.13E-05	0.687065
Snx18	-0.28209	3.39E-05	1
Suox	-0.28298	0.000162	1
Hdac1	-0.28368	0.000823	1
Ngly1	-0.28384	0.00128	1
Sfxn3	-0.28409	8.6E-06	0.277656
Top2b	-0.28438	0.00061	1
Aurka	-0.28579	0.000176	1
Atp6v1b2	-0.28584	0.000444	1
Sike1	-0.28656	0.000945	1
Adgrg1	-0.28666	0.002796	1
Mmd	-0.28672	0.001061	1
Ddx39b	-0.28728	0.001306	1
Cd200r4	-0.28827	0.006502	1
Cnn2	-0.28858	0.000485	1
Ccdc88c	-0.28979	0.002158	1
Rangap1	-0.28992	0.001257	1
Ankrd10	-0.29	0.022711	1
Smarca4	-0.2901	0.121183	1
Cdkn2d	-0.29022	0.012259	1
Slc41a3	-0.29058	0.016334	1
Txnl1	-0.29117	0.000915	1
Skiv2l	-0.2913	8.89E-05	1
Smc1a	-0.29169	0.010633	1
Dgkd	-0.29176	0.00047	1
Apobec2	-0.29178	5.51E-18	1.78E-13
Card11	-0.29235	0.001347	1
Lrmp	-0.2925	0.000699	1
Anp32e	-0.29289	0.275657	1
Slc23a2	-0.293	0.003877	1
Glipr2	-0.29305	0.00108	1

Coro7	-0.29344	4.93E-05	1
Sytl2	-0.29366	0.067775	1
Tpp1	-0.29374	0.004927	1
Cks2	-0.29381	0.094947	1
Taldo1	-0.2939	0.003628	1
Hnrnpr	-0.29397	2.81E-05	0.906549
Ep300	-0.29402	0.000208	1
Acly	-0.29421	0.000155	1
Lsp1	-0.29585	0.000192	1
Shcbp1	-0.29675	0.000414	1
Tacc3	-0.29697	0.002448	1
Ehbp1l1	-0.29746	0.001557	1
S100a11	-0.29925	0.000145	1
B3gat3	-0.29946	3.32E-06	0.107072
Galnt7	-0.29965	0.000293	1
Arhgdib	-0.29979	1.25E-06	0.040374
Ankrd44	-0.29989	6.54E-05	1
Anln	-0.30063	0.000142	1
S1pr4	-0.30165	0.000602	1
Rtn3	-0.30184	0.000367	1
Pole4	-0.30203	0.002567	1
Nup107	-0.30235	0.001028	1
Fubp1	-0.30268	0.001604	1
Add1	-0.30281	0.004698	1
Gltp	-0.30326	0.000496	1
Kif15	-0.30332	0.324372	1
Atpif1	-0.30342	0.007549	1
Nfia	-0.30351	0.020372	1
Mcu	-0.30365	4.89E-05	1
Capn2	-0.30382	0.002597	1
Adamts10	-0.30426	0.000197	1
Mrtfa	-0.3046	0.000866	1
Tipin	-0.30513	0.001147	1
Cd27	-0.30518	0.004692	1
Ppp4c	-0.30571	1.37E-05	0.440838
Aplp2	-0.30571	0.000233	1
Tnfrsf9	-0.30601	0.000487	1
Chchd10	-0.3064	0.229532	1
Paqr4	-0.30671	8.69E-06	0.280633
Atad2	-0.30699	0.023248	1
Sdcbp	-0.30728	0.00881	1
Gmnn	-0.3083	0.009851	1
Mad2l1	-0.30886	0.140787	1
Hnrnpk	-0.30956	9.37E-06	0.302421
Man1a	-0.31041	0.003213	1
Plp2	-0.31164	0.02793	1
Hnrnpa3	-0.31177	6.32E-05	1
Cfl1	-0.31177	1.45E-07	0.004675

Grk2	-0.31182	0.000625	1
Ect2	-0.31209	0.000907	1
Ubap2l	-0.31213	0.001681	1
Arhgef2	-0.31252	0.000203	1
Prkcb	-0.31378	0.001994	1
Tanc2	-0.3142	4.99E-08	0.001612
Trf	-0.31481	0.00039	1
Mdh1	-0.31531	0.000599	1
Arhgdia	-0.31565	4.17E-06	0.134573
Tsen34	-0.31601	0.02303	1
Cux1	-0.31631	0.013749	1
Pkig	-0.3174	0.278429	1
Smc6	-0.31816	0.0119	1
Plcg2	-0.31844	0.002925	1
Fkbp2	-0.31877	0.009024	1
Eed	-0.31892	0.005329	1
Nuf2	-0.31902	0.000699	1
Pdcd6ip	-0.31929	6.33E-05	1
Tpm3	-0.31953	7.91E-06	0.255324
Cdca2	-0.31965	0.001767	1
Cdc20	-0.31972	0.006976	1
Samd3	-0.31988	2.11E-08	0.000681
Baz1b	-0.32103	0.002789	1
Acot7	-0.32128	0.000746	1
Ccdc34	-0.322	0.019182	1
Sypl	-0.32213	6.63E-07	0.021413
Cd72	-0.32245	1.34E-06	0.043136
H2afv	-0.32251	0.15156	1
Pycrl	-0.3237	0.007456	1
Dtx1	-0.32486	1.16E-05	0.373561
Arpc5	-0.32505	6.94E-05	1
Myo1c	-0.32527	0.278154	1
Depdc1b	-0.32546	4.4E-06	0.142034
Kif18b	-0.32599	5.44E-05	1
Idh3a	-0.3264	5.05E-05	1
Smc3	-0.32643	0.000122	1
Unc93b1	-0.32663	0.103331	1
Mirt1	-0.32687	5.54E-07	0.01787
Gpx1	-0.32734	0.300566	1
Crip2	-0.32763	2.27E-14	7.34E-10
Mcm2	-0.32874	0.004039	1
Pold1	-0.32894	7.33E-05	1
Hnrnpm	-0.32895	0.007482	1
Slc25a4	-0.3293	0.000181	1
Ergic2	-0.33062	0.011273	1
Vasp	-0.33166	0.00011	1
C1qb	-0.33222	0.718043	1
Galnt2	-0.33226	1.72E-06	0.055686

Chchd2	-0.33226	0.000517	1
Atp2b1	-0.33341	0.001179	1
Hnrnpu	-0.33379	6.6E-05	1
Icam1	-0.33379	0.075126	1
Rfc1	-0.33406	3.19E-05	1
Clspn	-0.33511	0.000104	1
Hist1h2bm	-0.33525	5.76E-05	1
Rnaseh2b	-0.33563	0.02613	1
Rpa1	-0.33586	0.000127	1
Rps18	-0.33599	0.131055	1
Bax	-0.33687	0.002992	1
Rpn1	-0.33767	0.005787	1
Spp1	-0.33839	0.006486	1
Rfc2	-0.34147	0.028317	1
Ap1g2	-0.34383	1.02E-06	0.032844
Tank	-0.34391	0.002189	1
Rbm38	-0.34519	0.000124	1
Rfwd3	-0.34562	0.000717	1
Cers4	-0.34574	0.000113	1
Arhgef1	-0.34635	1.38E-06	0.044458
Rbbp4	-0.34658	0.000593	1
Adamts14	-0.34697	5.32E-07	0.017178
Rbbp7	-0.34837	0.005019	1
Susd1	-0.34845	3.65E-06	0.117976
Ilk	-0.34848	2.06E-05	0.663762
Myl6	-0.34861	1.23E-08	0.000396
Slc25a24	-0.34894	7.15E-07	0.023096
Abi1	-0.34927	2.22E-05	0.715869
Bex3	-0.34937	0.000637	1
Cdc42se1	-0.34967	0.000664	1
Slc38a2	-0.34968	0.073571	1
Tsc22d1	-0.35009	1.23E-06	0.03963
Ralgps2	-0.35015	0.743744	1
Sell	-0.3502	1.06E-10	3.41E-06
Cdk6	-0.35049	0.000703	1
Anapc5	-0.35053	0.004751	1
Uhrf1	-0.35066	0.000766	1
Vps37b	-0.35091	6.38E-05	1
Hells	-0.35092	0.003487	1
Stim1	-0.35103	6.12E-05	1
Lrrk1	-0.3517	2.87E-05	0.926826
Nup210	-0.35234	6.29E-05	1
Cenpa	-0.35255	0.01231	1
Cdkn3	-0.35344	0.003029	1
Itgb2	-0.35441	2.83E-06	0.091421
Ulbp1	-0.35521	0.001529	1
Ikzf3	-0.35593	0.002212	1
Mcm4	-0.35625	0.128466	1

Ncapg	-0.35627	4.12E-05	1
Man2b1	-0.35685	0.00143	1
Nr3c1	-0.3575	0.000354	1
Chsy1	-0.35862	2.3E-06	0.074097
Hnrnpab	-0.35871	0.006598	1
Setbp1	-0.36006	5.36E-05	1
Hnrnpf	-0.36021	9.11E-08	0.002942
Rnf2	-0.36068	1.61E-06	0.051962
Cep85	-0.36184	6.29E-06	0.20294
Spc24	-0.36235	0.001583	1
Pfn1	-0.36257	9.17E-11	2.96E-06
Exoc4	-0.36289	0.005879	1
Jak1	-0.36293	0.000134	1
Rnaset2b	-0.363	0.000111	1
Pde6d	-0.36328	8.99E-09	0.00029
Kif13b	-0.36424	0.001113	1
Pabpc1	-0.36428	3.2E-08	0.001032
Kpna2	-0.365	0.008656	1
Kif4	-0.36543	0.001248	1
Dcp1b	-0.36562	7.66E-05	1
Swap70	-0.36567	0.001051	1
Gm42418	-0.36574	0.164627	1
Aspm	-0.36582	6.65E-06	0.21467
Adgre5	-0.36603	0.000811	1
Ptbp3	-0.3664	3.84E-07	0.012407
Map7d1	-0.36667	2.88E-06	0.093003
Ncaph	-0.36775	3.04E-05	0.981988
Mcm6	-0.36878	0.0117	1
Abtb2	-0.36887	0.006976	1
Ctsl	-0.36901	0.368355	1
Dmac1	-0.36957	7.59E-05	1
Cdca8	-0.36965	0.00069	1
Ncapd3	-0.36993	0.008824	1
Lclat1	-0.37139	8.8E-05	1
Tyms	-0.3722	0.00016	1
Rhoc	-0.37231	8.15E-05	1
Hnrnpa2b1	-0.37248	2.24E-07	0.007244
Rnf43	-0.37472	4.81E-13	1.55E-08
Mtpn	-0.37477	2.83E-05	0.912862
Hist2h4	-0.37506	0.247018	1
Ap1m1	-0.37519	3.12E-06	0.100828
Irf8	-0.37583	0.001995	1
Reep5	-0.37699	1.12E-07	0.003609
Cbx1	-0.37857	0.029052	1
Nfic	-0.38028	5.39E-06	0.174152
Arhgap11a	-0.38073	0.003861	1
Stag1	-0.38079	5.24E-05	1
Ywhah	-0.3818	0.000225	1

Tcf19	-0.38414	0.001829	1
Tpm4	-0.38429	0.000273	1
Iqgap1	-0.38449	2.6E-07	0.00838
Hist1h2bb	-0.38472	7.16E-06	0.231092
Hmmr	-0.3855	0.000305	1
Mis18bp1	-0.38555	6.7E-05	1
Flot2	-0.38603	0.001265	1
Rad21	-0.38615	0.000579	1
Ccnf	-0.38733	2.75E-05	0.887783
St8sia4	-0.38744	2.12E-07	0.006846
S100a13	-0.38899	2.55E-06	0.082198
Lrrfip1	-0.38918	3.11E-06	0.100469
Hist1h2ac	-0.38935	2.37E-05	0.764884
Cd9	-0.39025	0.388703	1
Ndfip2	-0.39204	0.000371	1
Dnajc9	-0.39355	0.001785	1
Ran	-0.39387	2.73E-05	0.880908
Mplkip	-0.39518	0.001083	1
Eml3	-0.39534	1.89E-09	6.09E-05
Gm11808	-0.39545	2.25E-07	0.007266
Hist1h3c	-0.39721	0.000124	1
Foxp1	-0.3978	0.411851	1
Jarid2	-0.39801	1.61E-05	0.519859
Cenpe	-0.39948	0.018826	1
Cmklr1	-0.39975	1.06E-21	3.42E-17
Prex1	-0.40008	0.013076	1
Azin1	-0.40155	0.00373	1
Sp4	-0.40244	7.92E-06	0.255548
Suz12	-0.40331	0.000141	1
Tk1	-0.40345	0.003791	1
Pcna	-0.404	0.000879	1
Hist2h2ac	-0.40492	0.019805	1
Uba2	-0.40497	0.000109	1
Stk10	-0.40818	3.21E-07	0.010373
Nucks1	-0.41067	0.012054	1
Morrbid	-0.41225	4.99E-07	0.016099
Ckap2l	-0.41249	0.00064	1
Gm19951	-0.41275	1.57E-05	0.506601
Kif23	-0.4148	7.33E-05	1
Neil3	-0.4157	1.75E-05	0.563813
Calm2	-0.41709	0.000114	1
Pik3r5	-0.41715	1.15E-09	3.7E-05
Perp	-0.41737	7.03E-10	2.27E-05
Aurkb	-0.41886	6.02E-05	1
Zbtb32	-0.41941	1.51E-05	0.487259
Slc27a4	-0.4198	2.37E-19	7.67E-15
Hypk	-0.42065	2.27E-05	0.733377
Serp2	-0.42151	2.64E-12	8.51E-08

Sub1	-0.42166	2.56E-09	8.27E-05
Lrrc8c	-0.42167	2.46E-06	0.079442
Nt5c3b	-0.4218	5.06E-06	0.163465
Ezr	-0.42188	5.44E-07	0.017548
Baiap3	-0.42319	2.39E-09	7.72E-05
Hmgb1	-0.42355	4.15E-06	0.133966
Gnb2	-0.42467	3.38E-08	0.001091
Cd244a	-0.4249	1.54E-09	4.99E-05
Gab2	-0.42685	0.00048	1
Dck	-0.42843	1.7E-05	0.549024
Cks1b	-0.42857	0.003515	1
Lgals3	-0.42871	0.003876	1
C1qa	-0.42902	0.61152	1
Jpt1	-0.43085	4.16E-06	0.134237
Plec	-0.43091	0.001225	1
Bin1	-0.43173	2.19E-07	0.007064
Hist2h2bb	-0.43203	0.016259	1
Cdca3	-0.4334	0.000356	1
Aff3	-0.43506	0.446372	1
Capzb	-0.43583	1.32E-13	4.26E-09
S100a4	-0.43602	0.000577	1
Actg1	-0.43665	2.72E-09	8.79E-05
Csk	-0.43717	2.73E-08	0.000881
Srsf2	-0.43726	0.000584	1
St3gal6	-0.43748	1.8E-05	0.580487
Hmga1	-0.43977	0.000132	1
Gnas	-0.44181	0.011164	1
Ptma	-0.44215	0.082586	1
Actr3	-0.44251	2.76E-10	8.92E-06
Ccnb2	-0.44252	0.000251	1
Ckap5	-0.44601	0.064646	1
Esyt1	-0.4463	1.23E-08	0.000396
Pola1	-0.44701	0.000533	1
Cit	-0.44748	1.2E-05	0.387413
Apbb1ip	-0.44751	1.12E-06	0.036151
Mef2a	-0.44808	0.000248	1
Sptan1	-0.44839	0.000232	1
Nkg7	-0.45129	3.37E-09	0.000109
S1pr1	-0.45559	4.69E-10	1.51E-05
Ddx39	-0.4563	5.02E-05	1
Gm45552	-0.4569	2.89E-12	9.32E-08
Snx10	-0.45798	2.27E-05	0.733561
Gm42047	-0.45829	1.39E-06	0.044728
Marcks	-0.45858	0.000978	1
Klf3	-0.45906	3.41E-13	1.1E-08
Hsp90b1	-0.46173	9.28E-05	1
Slbp	-0.46275	1.87E-06	0.060486
Sptbn1	-0.46367	0.000575	1

Erh	-0.46383	5.06E-07	0.016321
Hmgn1	-0.46437	0.000246	1
Glul	-0.46453	0.005821	1
Pde2a	-0.46654	7.5E-18	2.42E-13
Ehd4	-0.46675	1.37E-06	0.044256
Tagln2	-0.46953	0.001128	1
Ikzf2	-0.47069	1.73E-08	0.000558
Tceal9	-0.47156	5.11E-07	0.016482
Actb	-0.47233	2.26E-15	7.3E-11
Klra3	-0.47491	0.006759	1
Me2	-0.47585	4.32E-10	1.39E-05
Il10ra	-0.47809	7.69E-09	0.000248
Lat2	-0.47948	2.81E-06	0.09058
H3f3a	-0.48139	0.000183	1
Itgam	-0.48163	9.92E-09	0.00032
Fmnl1	-0.48171	2.31E-08	0.000746
Hexb	-0.4821	0.000223	1
Iqgap2	-0.48386	7.09E-05	1
Serpinb6b	-0.48508	1.92E-05	0.621296
Fam129a	-0.48557	0.000135	1
Syk	-0.48665	1.34E-07	0.004311
Lbr	-0.48703	5.44E-08	0.001757
Arsb	-0.48977	8.66E-06	0.279588
Dek	-0.49126	2.34E-05	0.75648
Ezh2	-0.49301	0.002172	1
Ctsd	-0.4935	5.01E-07	0.016182
Arhgef18	-0.49381	4.01E-06	0.129495
Rcsd1	-0.49394	1.09E-07	0.003532
Calm1	-0.49517	3.33E-12	1.07E-07
Runx1	-0.49575	1.28E-05	0.413581
Gm43305	-0.49632	8.38E-09	0.00027
Incenp	-0.49681	0.155433	1
E2f2	-0.49697	3.66E-10	1.18E-05
Vim	-0.49781	1.42E-05	0.459691
Mef2c	-0.49908	2.18E-07	0.007025
Ncapd2	-0.50161	0.01424	1
Gpd2	-0.50236	3.01E-08	0.000971
Pmf1	-0.50238	6.89E-06	0.22235
Car2	-0.50376	3.34E-24	1.08E-19
Cdc42	-0.5039	5.92E-16	1.91E-11
Lig1	-0.50559	0.00015	1
Ywhaq	-0.50833	1.57E-10	5.06E-06
Hist1h3i	-0.50941	1.73E-05	0.558585
Asf1b	-0.51652	0.001472	1
Cdk1	-0.51799	1.01E-05	0.325968
Nfyb	-0.52056	2.78E-11	8.99E-07
Pafah1b3	-0.52119	0.00756	1
Zeb2	-0.52424	0.011274	1

Add3	-0.52542	2.09E-08	0.000675
Kif22	-0.5281	2.1E-05	0.676572
Il2rb	-0.52862	1.88E-06	0.060756
Klrk1	-0.5323	0.002631	1
Dnah8	-0.53234	2.08E-10	6.71E-06
Kcnab2	-0.53269	7.87E-08	0.002539
Sidt1	-0.53333	9.48E-10	3.06E-05
Rom1	-0.53439	1.73E-27	5.58E-23
Nkain2	-0.5349	1.76E-31	5.67E-27
Nsmaf	-0.53515	9.36E-11	3.02E-06
Lockd	-0.53638	2.4E-07	0.007762
Arl5c	-0.53707	2.25E-16	7.25E-12
Fyn	-0.53998	2.16E-08	0.000696
Tpx2	-0.54377	0.004943	1
Mcm5	-0.54496	1.01E-06	0.032489
Osbpl3	-0.54908	1.45E-08	0.000467
Serf2	-0.55261	2.17E-15	6.99E-11
Pik3r1	-0.55386	1.48E-07	0.004764
Nusap1	-0.55724	0.001228	1
Pglyrp1	-0.55984	1.02E-05	0.330526
Gas7	-0.56022	1.29E-25	4.15E-21
Kcnq5	-0.56188	2.34E-05	0.756602
Mcm3	-0.56245	0.000126	1
Tuba1a	-0.56448	0.000128	1
Lyn	-0.56758	0.001144	1
Smc4	-0.5681	0.000366	1
Rrm1	-0.57612	4.12E-06	0.132984
Tubb4b	-0.57718	0.201645	1
Ube2s	-0.57996	0.072221	1
Ifi30	-0.58259	0.009566	1
Fbxo5	-0.58377	1.31E-05	0.423258
Cmah	-0.58508	3.1E-10	9.99E-06
Racgap1	-0.59033	9.63E-07	0.031079
Txk	-0.59532	4.21E-10	1.36E-05
Bin2	-0.59832	1.87E-10	6.04E-06
Fgfr2	-0.59912	0.000632	1
Smc2	-0.60052	0.007349	1
Ccna2	-0.60076	1.63E-05	0.527277
Birc5	-0.6074	6.55E-05	1
Lmo4	-0.61369	0.012002	1
P2ry12	-0.61785	0.508589	1
Pycard	-0.62147	5.69E-10	1.84E-05
Hmgn2	-0.62429	0.013636	1
Sh2d1a	-0.62812	1.39E-09	4.49E-05
Nrm	-0.63021	2.27E-09	7.33E-05
Foxn3	-0.6307	5.71E-08	0.001844
Kif11	-0.63087	0.001184	1
Prc1	-0.63208	1.79E-05	0.577466

Ptpn4	-0.63249	5.11E-11	1.65E-06
Cenpf	-0.63314	0.000816	1
Cd48	-0.64495	9.52E-11	3.07E-06
H2-Q6	-0.64598	5.15E-05	1
C1qc	-0.64766	0.299412	1
Hist1h1a	-0.65064	0.000886	1
Diaph3	-0.65415	0.000533	1
Agpat3	-0.65468	3.46E-11	1.12E-06
Sept11	-0.65616	6.97E-11	2.25E-06
S100a10	-0.65712	8.8E-15	2.84E-10
Nedd4	-0.66451	4.47E-08	0.001442
Cd55	-0.6657	3.03E-32	9.79E-28
H2-D1	-0.6695	0.512226	1
Dnmt1	-0.67911	5.7E-07	0.018407
Knl1	-0.68343	8.71E-06	0.281321
Tubb5	-0.68615	0.46288	1
Tm6sf1	-0.69089	8.94E-15	2.89E-10
Fcgr3	-0.69178	0.000385	1
Anxa6	-0.69271	1.35E-19	4.37E-15
Hist1h3e	-0.70203	0.002692	1
Fry	-0.70371	2.61E-24	8.41E-20
Txndc5	-0.71406	7.4E-15	2.39E-10
Ighm	-0.71824	0.000643	1
Gm2682	-0.71887	1.1E-05	0.35527
Atp2a3	-0.71899	3.9E-15	1.26E-10
Cdc25b	-0.72744	9.68E-14	3.13E-09
Rap1gap2	-0.73473	3.26E-23	1.05E-18
Myo1f	-0.73506	8.9E-12	2.87E-07
Zyx	-0.73792	5.58E-15	1.8E-10
Cd7	-0.74393	2.22E-05	0.71692
Ube2c	-0.75997	6E-05	1
Tox	-0.76555	8.89E-12	2.87E-07
Klre1	-0.77218	6.19E-05	1
Fcer1g	-0.78066	0.565798	1
Ndufa4	-0.78588	9.22E-19	2.98E-14
Rrm2	-0.79151	1.13E-06	0.03634
Myo1g	-0.79238	3.28E-19	1.06E-14
Klrg1	-0.8014	4.03E-14	1.3E-09
Plek	-0.82138	7.18E-09	0.000232
Pclaf	-0.82429	8.67E-05	1
Lmnb1	-0.82732	2.64E-05	0.852398
Bach2	-0.82798	1.52E-09	4.91E-05
Rasa3	-0.84341	6.7E-13	2.16E-08
Tuba1c	-0.84625	6.47E-06	0.208847
H2afz	-0.8596	2.63E-08	0.000848
Hmgb2	-0.87301	3.88E-06	0.125229
Anxa2	-0.87508	5.26E-19	1.7E-14
Ahnak	-0.88884	4.21E-09	0.000136

Prr5l	-0.91709	1.67E-55	5.38E-51
Hist1h4d	-0.93112	0.00177	1
H2afx	-0.93364	0.020795	1
Gzmk	-0.94066	1.73E-13	5.58E-09
Igkc	-0.95343	2E-28	6.47E-24
Top2a	-0.96565	0.000936	1
Crip1	-0.98697	7.06E-23	2.28E-18
Eomes	-0.99099	7.99E-41	2.58E-36
Stmn1	-1.00239	4.21E-08	0.00136
Emp3	-1.02766	3.55E-20	1.14E-15
Trbv3	-1.04778	2.52E-22	8.14E-18
Lpin1	-1.05453	2.79E-33	9E-29
Flna	-1.05758	2.29E-20	7.41E-16
Hist1h2af	-1.0682	1.92E-05	0.61956
Trav13-1	-1.08257	1.07E-10	3.44E-06
Trbv13-1	-1.08539	0.007027	1
Tuba1b	-1.08709	0.002087	1
Lgals1	-1.09578	1.52E-21	4.92E-17
Cx3cr1	-1.13437	2.15E-18	6.93E-14
Tyrobp	-1.16074	0.000138	1
Mki67	-1.16791	8.79E-05	1
Gm30211	-1.18466	6.66E-18	2.15E-13
Ccl5	-1.20784	5.24E-15	1.69E-10
Itga4	-1.21579	8.33E-22	2.69E-17
Cst3	-1.23164	2.02E-05	0.65309
Ripor2	-1.28846	6.81E-21	2.2E-16
Hist1h1c	-1.31309	1.84E-05	0.594979
Gm20628	-1.31324	0.000102	1
Hist1h2ae	-1.31444	0.000133	1
Hist1h2ab	-1.34462	1.4E-05	0.452098
Hist1h1e	-1.39219	2.49E-05	0.805139
Rasgrp2	-1.48674	3.14E-32	1.01E-27
Hist1h2ak	-1.50048	5.21E-05	1
Fah	-1.55221	4.34E-56	1.4E-51
Hist1h1b	-1.55922	4.54E-08	0.001467
Hist1h1d	-1.58273	6.24E-07	0.02015
Trbv1	-1.69083	8.5E-12	2.75E-07
Gzma	-1.96533	1.62E-17	5.24E-13
Klf2	-1.96821	7.37E-36	2.38E-31
Cd74	-2.20923	0.00124	1

SUPPLEMENTAL TABLE 2. Differentially Regulated Genes in GVHD versus BM Macrophages

Gene	avg_log2FC	p_val	p_val_adj
Chil3	2.903806	4.51E-27	1.46E-22
Cxcl9	2.442837	3.37E-31	1.09E-26
Ccl5	2.033277	2.12E-14	6.83E-10
Cfb	1.957911	1.67E-37	5.38E-33
C1qb	1.913075	2.22E-32	7.15E-28
Mif	1.845336	3.04E-46	9.8E-42
S100a8	1.843425	2.51E-20	8.12E-16
Arg1	1.756947	7.01E-15	2.26E-10
C1qc	1.704767	1.5E-29	4.84E-25
AA467197	1.670829	2.79E-27	9.01E-23
C1qa	1.625199	1.38E-32	4.47E-28
Atp1b3	1.597196	2.9E-23	9.35E-19
Cstb	1.569929	5.44E-46	1.76E-41
Ly6a	1.514247	4.82E-33	1.56E-28
Mt1	1.479602	5.39E-40	1.74E-35
AW11201C	1.455218	5.57E-30	1.8E-25
Gbp2b	1.426488	5.26E-23	1.7E-18
Naaa	1.412619	1.01E-43	3.26E-39
Saa3	1.282853	6.29E-13	2.03E-08
Capg	1.255354	1.75E-38	5.65E-34
Upp1	1.194638	2.57E-28	8.29E-24
Cxcl10	1.185069	1.46E-16	4.71E-12
Atp6v0c	1.16339	3.1E-40	1E-35
Gbp2	1.162065	3.46E-22	1.12E-17
Fth1	1.154309	1.24E-26	4E-22
Tpi1	1.150908	5.68E-33	1.83E-28
Ctsd	1.149506	3.08E-35	9.95E-31
Tbxas1	1.144055	1.83E-42	5.91E-38
Pgam1	1.139071	2.75E-30	8.87E-26
Fcgr2b	1.113581	5.5E-30	1.77E-25
Ctsc	1.108892	1.06E-31	3.41E-27
S100a11	1.095159	9.98E-28	3.22E-23
Aldoa	1.087262	4.34E-36	1.4E-31
Gbp4	1.070632	2.17E-33	7E-29
Cd1d1	1.067626	1.56E-34	5.05E-30
Ifi30	1.064698	2.34E-34	7.57E-30
Socs1	1.053212	2.67E-31	8.63E-27
Gbp7	1.04851	1.87E-32	6.05E-28
Txn1	1.047075	3.65E-35	1.18E-30
Ier3	1.024432	1.09E-22	3.51E-18
Dok2	1.024311	7.54E-33	2.43E-28
Fkbp5	1.019976	1.44E-27	4.66E-23
Sdc4	1.006327	1.16E-22	3.75E-18
H2-Eb1	1.000127	7.83E-18	2.53E-13

Lcn2	0.994687	8.96E-10	2.89E-05
S100a10	0.993781	3.18E-26	1.03E-21
H2-Ab1	0.981721	2.67E-23	8.63E-19
Shtn1	0.972734	3.17E-38	1.02E-33
Creb5	0.971936	5.82E-28	1.88E-23
Cd38	0.963919	1.07E-37	3.45E-33
Hif1a	0.960531	3.64E-18	1.17E-13
Glrx	0.949105	9.01E-26	2.91E-21
St7	0.944361	5.85E-31	1.89E-26
Ccr1	0.943944	1.05E-34	3.39E-30
Fcgr3	0.94241	1.48E-28	4.79E-24
Serpina3g	0.93499	1.25E-17	4.03E-13
Ninj1	0.933248	2.01E-22	6.49E-18
Ccl6	0.92701	6.41E-15	2.07E-10
C4b	0.92554	6.26E-17	2.02E-12
C3	0.924908	5.18E-14	1.67E-09
Sod2	0.924902	9.94E-15	3.21E-10
Gbp8	0.924511	6.2E-26	2E-21
Cox8a	0.92304	9.2E-44	2.97E-39
Tspo	0.89432	2.77E-28	8.94E-24
Lpl	0.887176	3.61E-17	1.16E-12
Pfkp	0.8857	1.8E-24	5.81E-20
Agtrap	0.884959	2.33E-24	7.53E-20
Fmnl2	0.879808	2.7E-20	8.71E-16
Ass1	0.877781	7.26E-14	2.34E-09
Iigp1	0.8684	9.04E-21	2.92E-16
Gbp5	0.867145	9.66E-18	3.12E-13
Ldha	0.861597	3.46E-23	1.12E-18
Bst1	0.856423	2.31E-30	7.45E-26
Clec4n	0.855922	4.8E-14	1.55E-09
Atp5g3	0.852845	2.09E-34	6.76E-30
Atp5g1	0.8494	1.63E-26	5.28E-22
Cfp	0.847871	1.25E-27	4.03E-23
Ccr5	0.841761	6.38E-24	2.06E-19
F10	0.841671	2.03E-17	6.56E-13
mt-Atp8	0.837301	1.64E-39	5.3E-35
Mertk	0.834458	2.01E-23	6.49E-19
Zc3h7a	0.829482	0.079924	1
Fbxl5	0.824349	3.36E-17	1.09E-12
Il18bp	0.816485	5E-25	1.61E-20
Acod1	0.815922	2.52E-07	0.008144
Dip2c	0.801431	4.45E-27	1.44E-22
Gatm	0.798568	1.02E-27	3.3E-23
H2-Aa	0.797788	5.24E-19	1.69E-14
Ms4a8a	0.79773	1.57E-24	5.07E-20
Tagln2	0.796502	8.54E-24	2.76E-19
App	0.793895	1.48E-25	4.79E-21
Ifi205	0.792014	7.31E-20	2.36E-15

Aprt	0.789039	1.13E-25	3.64E-21
Gapdh	0.787903	1.89E-29	6.1E-25
Frmd4b	0.786873	3.48E-19	1.12E-14
Cd164	0.782566	4.85E-29	1.57E-24
Spint1	0.781028	2.93E-26	9.46E-22
Snx5	0.77921	6.7E-25	2.16E-20
Wfdc21	0.778131	1.69E-11	5.45E-07
Ctsl	0.776484	4.3E-21	1.39E-16
Tfec	0.77453	1.52E-31	4.92E-27
Klhl6	0.772336	5.71E-19	1.85E-14
Gbp6	0.765402	2.97E-17	9.59E-13
Anxa4	0.765115	4.43E-24	1.43E-19
Thbs1	0.763252	7.76E-07	0.025042
Grn	0.762587	6.69E-21	2.16E-16
Lilr4b	0.762263	5.98E-19	1.93E-14
Pycard	0.756628	4.8E-19	1.55E-14
Prdx5	0.754031	1.65E-22	5.34E-18
Prelid1	0.752677	9.96E-27	3.21E-22
Cxcl16	0.749569	3.61E-16	1.17E-11
Jaml	0.749544	3.88E-20	1.25E-15
Chchd10	0.739599	1.08E-33	3.5E-29
Rps2	0.737055	5.79E-30	1.87E-25
Rpl12	0.736385	3.56E-22	1.15E-17
Slc31a2	0.730658	2.41E-19	7.79E-15
Cox7b	0.725499	1.65E-20	5.34E-16
Lap3	0.725312	3.98E-22	1.28E-17
Anxa3	0.723557	2.37E-24	7.65E-20
Mitf	0.723515	9.12E-19	2.95E-14
Sell	0.72168	6.37E-18	2.06E-13
Ctsz	0.721674	2.12E-21	6.86E-17
Kynu	0.72151	3.64E-19	1.18E-14
Gpx4	0.721163	5.21E-15	1.68E-10
Axl	0.717442	4.69E-23	1.52E-18
Cd74	0.713094	3.23E-16	1.04E-11
Nme2	0.711868	6.23E-24	2.01E-19
Myof	0.702245	3.91E-19	1.26E-14
Rrbp1	0.701046	6.34E-20	2.05E-15
Cxcl2	0.695396	1.33E-08	0.000429
Bcl2a1b	0.694163	6.02E-12	1.94E-07
Ecm1	0.69223	7.2E-21	2.33E-16
Wfdc17	0.691003	4.89E-13	1.58E-08
Clec12a	0.689439	2.37E-17	7.65E-13
mt-Co2	0.689195	1.65E-26	5.33E-22
mt-Co3	0.689164	3.3E-21	1.07E-16
Tuba1b	0.689115	5.62E-13	1.81E-08
Scimp	0.688973	1.59E-24	5.12E-20
Ly6i	0.682683	0.090609	1
C3ar1	0.678725	2.73E-23	8.82E-19

Gbp3	0.677923	6E-16	1.94E-11
C1qbp	0.675363	6.06E-19	1.96E-14
Plxdc2	0.672168	5.89E-09	0.00019
Pim1	0.667078	6.45E-16	2.08E-11
Rpl14	0.666962	7.04E-27	2.27E-22
Serpina3f	0.661871	9.06E-25	2.92E-20
Cd84	0.660691	4.69E-19	1.51E-14
Mpc1	0.659	1.23E-17	3.96E-13
Ran	0.656861	6.8E-16	2.2E-11
Vim	0.656262	5.05E-16	1.63E-11
Tubb5	0.655214	1.09E-09	3.51E-05
Tarm1	0.654578	2.6E-24	8.4E-20
Il1r2	0.652791	1.87E-15	6.04E-11
Slc7a2	0.646684	6.07E-14	1.96E-09
Rplp1	0.645917	1.59E-32	5.12E-28
Clec5a	0.645798	2.53E-14	8.16E-10
Slamf8	0.643568	9.7E-19	3.13E-14
Cox5b	0.642791	2.56E-23	8.27E-19
Pkm	0.642344	2.07E-17	6.69E-13
Mmp14	0.634331	1.61E-08	0.000519
Vdac2	0.629021	2.35E-19	7.59E-15
Nampt	0.628468	2.14E-16	6.9E-12
Dab2	0.627984	4.06E-32	1.31E-27
Hk3	0.625818	7.92E-12	2.56E-07
Lgals3bp	0.624823	2.35E-15	7.59E-11
Myo1e	0.623945	5.29E-25	1.71E-20
Atp5b	0.622097	1.77E-17	5.71E-13
Msr1	0.620088	2.1E-14	6.77E-10
Gm4951	0.612996	3.14E-18	1.02E-13
Lilrb4a	0.611757	2.6E-13	8.41E-09
Sh3pxd2b	0.611354	7.71E-23	2.49E-18
Akr1a1	0.60841	1.12E-18	3.6E-14
F7	0.607049	2.04E-20	6.58E-16
Eps8	0.605841	3.7E-15	1.19E-10
Npc2	0.605629	1.19E-18	3.83E-14
Atp5o	0.60409	7.97E-18	2.57E-13
H2-DMb2	0.601906	2.56E-16	8.27E-12
mt-Co1	0.601543	2.06E-22	6.65E-18
mt-Cytb	0.600054	1.47E-15	4.74E-11
Picalm	0.598295	9.14E-11	2.95E-06
Cdkn1a	0.596712	2.98E-13	9.61E-09
AY036118	0.596181	0.025351	1
Tubb4b	0.595577	3.89E-11	1.26E-06
Prdx6	0.587734	1.02E-14	3.3E-10
Rbm3	0.585703	7.62E-19	2.46E-14
H2-DMa	0.582849	1.31E-14	4.22E-10
Arhgap24	0.581887	6.11E-14	1.97E-09
mt-Nd1	0.580309	2.14E-11	6.92E-07

Nos2	0.580294	1.51E-07	0.004864
Pgk1	0.578259	4.84E-12	1.56E-07
Bst2	0.577393	1.38E-14	4.45E-10
Psma5	0.577168	3.43E-18	1.11E-13
Procr	0.576351	1.44E-14	4.65E-10
Atp5g2	0.57296	4.32E-18	1.4E-13
S100a1	0.571994	1.74E-25	5.62E-21
Eif5a	0.571303	6.67E-12	2.15E-07
mt-Atp6	0.568511	9.01E-16	2.91E-11
Ndufb8	0.568142	2.26E-16	7.29E-12
Aoah	0.568013	1.43E-15	4.61E-11
Hopx	0.565022	1.76E-12	5.68E-08
Cd300lf	0.56397	7.23E-16	2.34E-11
Csf2rb	0.563155	3.89E-12	1.26E-07
Fgl2	0.561953	5.28E-13	1.7E-08
Sdcbp	0.561425	1.7E-15	5.5E-11
AU020206	0.559445	1.79E-13	5.78E-09
Clcn7	0.556461	7.6E-18	2.45E-13
Lmna	0.554468	1.51E-17	4.87E-13
Calr	0.55421	5.11E-11	1.65E-06
Htr7	0.551978	9.82E-21	3.17E-16
Ndufa12	0.550757	8.76E-17	2.83E-12
Gns	0.546499	1.75E-12	5.66E-08
Igtp	0.544926	1.63E-15	5.26E-11
Atpif1	0.544554	4.67E-16	1.51E-11
Cndp2	0.544538	3.69E-14	1.19E-09
Trf	0.543939	0.000121	1
Fabp5	0.543867	0.066047	1
Lgals1	0.540306	2.21E-10	7.14E-06
Sdhd	0.539016	2.02E-14	6.51E-10
Cox6a1	0.537384	4.52E-15	1.46E-10
Efhd2	0.537166	5.14E-14	1.66E-09
Atp5j	0.533148	1.19E-15	3.84E-11
Slc3a2	0.532367	3.41E-13	1.1E-08
Socs3	0.529401	1.01E-09	3.25E-05
Slamf7	0.528996	4.88E-13	1.57E-08
Kcnn4	0.528397	8.65E-21	2.79E-16
Zfp703	0.528278	8.58E-23	2.77E-18
Slco3a1	0.527266	2.83E-09	9.14E-05
Cd24a	0.524121	4.65E-17	1.5E-12
Aif1	0.522834	1.29E-12	4.15E-08
Slc49a4	0.522296	7.85E-16	2.53E-11
Cd274	0.521216	1.14E-08	0.000367
Lgals9	0.52058	1.72E-11	5.56E-07
Anpep	0.519095	6.78E-16	2.19E-11
mt-Nd4l	0.518161	2.13E-11	6.88E-07
Vdac1	0.514616	1.79E-16	5.79E-12
Gda	0.512384	4.6E-12	1.49E-07

P2ry14	0.512271	1.87E-20	6.03E-16
Gpnmb	0.511499	1.64E-12	5.31E-08
Clic4	0.510517	5.12E-08	0.001653
Emb	0.509546	7.41E-12	2.39E-07
Acly	0.509095	2.43E-09	7.84E-05
Arid5b	0.507417	7.91E-09	0.000255
Tgfbi	0.507032	5.74E-10	1.85E-05
Dram1	0.504133	4.53E-09	0.000146
Crip1	0.503286	1.31E-08	0.000421
1600014C:	0.502613	8.07E-13	2.61E-08
Palld	0.498993	1.83E-23	5.9E-19
Ndufb6	0.498849	7.02E-10	2.26E-05
Eepd1	0.495928	1.24E-18	4.02E-14
Uck2	0.495739	2.52E-23	8.15E-19
Ctss	0.494115	1.12E-10	3.62E-06
Acsl1	0.491726	0.000176	1
Rps20	0.490741	8.18E-20	2.64E-15
Csf2rb2	0.489585	5.92E-16	1.91E-11
mt-Nd5	0.489312	1.38E-09	4.45E-05
Ppia	0.489185	2.13E-19	6.88E-15
Manf	0.488366	8.23E-11	2.66E-06
Isg15	0.485706	9.23E-09	0.000298
Psma3	0.484158	1.11E-12	3.58E-08
Nme1	0.48361	1.73E-12	5.57E-08
Oasl1	0.480925	3.77E-09	0.000122
Slfn1	0.47929	4.18E-11	1.35E-06
Htra3	0.477639	3.07E-11	9.91E-07
Pdia3	0.477164	1.5E-09	4.84E-05
Nhp2	0.476102	3.57E-14	1.15E-09
Rnf7	0.475616	4.82E-14	1.56E-09
Wars	0.47559	8.28E-13	2.67E-08
Hmgn2	0.475076	2E-10	6.45E-06
Atox1	0.473872	2.02E-12	6.52E-08
Fkbp2	0.472653	1.3E-12	4.21E-08
Rpl10a	0.472594	3.2E-15	1.03E-10
Klf9	0.47003	9.69E-17	3.13E-12
Cd14	0.469213	5.16E-12	1.67E-07
Aopep	0.46843	2.43E-16	7.85E-12
Hmga1	0.467083	2.98E-11	9.63E-07
Nek6	0.466428	1.44E-17	4.64E-13
Mrpl23	0.465838	9.63E-14	3.11E-09
Coa5	0.464495	2.85E-14	9.22E-10
Ddhd1	0.461705	7.87E-10	2.54E-05
Amdhd2	0.461228	6.1E-10	1.97E-05
Uqcrb	0.459076	1.24E-13	4.01E-09
Pde7b	0.458621	3.02E-06	0.097539
Ppib	0.45852	9.27E-11	2.99E-06
Il1rn	0.457997	4.99E-09	0.000161

Atrnl1	0.45655	9.2E-12	2.97E-07
Sema4a	0.45613	7.9E-09	0.000255
Ppargc1b	0.456102	9.27E-19	2.99E-14
Mki67	0.455267	6.73E-09	0.000217
Snrpf	0.45302	1.17E-09	3.78E-05
Igsf8	0.452783	9.89E-15	3.19E-10
Slc25a3	0.450962	1.21E-12	3.9E-08
Glipr2	0.449991	7.82E-14	2.52E-09
Got1	0.448803	8.7E-20	2.81E-15
Ccdc86	0.44871	2.33E-17	7.53E-13
Sdhb	0.444462	1.04E-12	3.35E-08
Acadl	0.443558	2.86E-14	9.24E-10
Resf1	0.442177	4.61E-06	0.148965
Ndufs3	0.441622	4.14E-12	1.34E-07
Psen2	0.441544	2.96E-12	9.56E-08
Chn2	0.440635	4.05E-06	0.130794
Micos10	0.440582	2.39E-12	7.7E-08
Ak2	0.439955	5.08E-10	1.64E-05
Slc25a5	0.439216	4.29E-14	1.38E-09
H2-DMb1	0.438957	9.5E-14	3.07E-09
Ndufc1	0.438319	8.25E-10	2.66E-05
Trerf1	0.438026	4.09E-10	1.32E-05
Map2k2	0.43669	7.77E-13	2.51E-08
Ctsh	0.434559	2.44E-10	7.86E-06
C1ra	0.434403	2.48E-18	8.02E-14
CAAA0114[illegible]	0.432677	3.29E-08	0.001063
Atp5d	0.429769	1.85E-11	5.97E-07
Gas7	0.429542	5.35E-15	1.73E-10
Card19	0.42843	3.24E-12	1.05E-07
Ndufv3	0.426788	9.53E-13	3.08E-08
Ube2c	0.426243	8.28E-05	1
Spon1	0.424089	5.42E-12	1.75E-07
Irf2bp2	0.423181	4.29E-12	1.39E-07
Arrdc4	0.421453	1.74E-14	5.61E-10
Dusp1	0.420028	2.21E-06	0.071503
Pld3	0.419684	1.01E-12	3.25E-08
Ccl2	0.419092	0.003971	1
Ccl4	0.418573	3.2E-05	1
Ttc39b	0.41844	5.04E-13	1.63E-08
Tlr4	0.417727	1.31E-11	4.24E-07
Pebp1	0.417008	4.64E-14	1.5E-09
Top2a	0.41696	0.041596	1
Stmn1	0.416834	5.66E-06	0.182738
Large1	0.416181	0.000536	1
Slc2a1	0.414209	2.37E-07	0.00766
Scarf1	0.414066	3.91E-14	1.26E-09
Psmb10	0.413989	1.01E-13	3.27E-09
Acp5	0.413857	1.04E-05	0.336813

Cox6b1	0.41324	2.03E-13	6.56E-09
Alas1	0.41255	6.51E-06	0.210293
Rpl32	0.412449	7.47E-14	2.41E-09
Apoe	0.412281	0.002644	1
Mgst1	0.412208	4.03E-07	0.012996
Pacsin2	0.411179	2.41E-10	7.77E-06
Pnpla2	0.411161	2.02E-10	6.51E-06
Qpct	0.411068	2.01E-08	0.00065
Nrp2	0.411006	1.23E-15	3.98E-11
Rasgef1b	0.410204	5.61E-10	1.81E-05
Ptpmt1	0.410169	1.25E-13	4.03E-09
Eno1	0.409666	3.72E-10	1.2E-05
Rps17	0.409539	1.53E-11	4.93E-07
Psma6	0.409538	2.11E-10	6.82E-06
Gng2	0.409327	2.71E-10	8.74E-06
Ly6c1	0.408845	1.68E-06	0.054194
Ndufa1	0.407172	1.34E-08	0.000433
Cnn3	0.407102	2.82E-10	9.09E-06
Bnip3	0.40709	1.25E-12	4.02E-08
Csrp1	0.406414	8.91E-12	2.88E-07
Ifi47	0.406308	5.65E-08	0.001825
Idh3b	0.404463	1.09E-09	3.52E-05
Snx2	0.404166	2.78E-08	0.000898
Tmem154	0.403543	2.78E-13	8.97E-09
Psme2	0.403378	3.26E-09	0.000105
Rps27l	0.40091	1.31E-08	0.000422
0610012G(	0.400341	3.74E-14	1.21E-09
F830016B(	0.398744	1.77E-15	5.71E-11
Uqcrq	0.398729	1.3E-10	4.21E-06
Gng12	0.397732	9.37E-11	3.02E-06
Ppa1	0.397021	6.63E-17	2.14E-12
Ndufa13	0.397003	4.05E-08	0.001307
Rab7b	0.395599	4.71E-11	1.52E-06
Anp32b	0.395442	4.99E-11	1.61E-06
Atp5a1	0.394656	1.53E-09	4.94E-05
Tcirg1	0.393587	1.15E-11	3.73E-07
Sla	0.393291	2.05E-06	0.066282
Atp6ap2	0.393045	5.65E-10	1.83E-05
Litaf	0.392755	3.39E-09	0.000109
Sdf2l1	0.392091	3.28E-08	0.00106
P4hb	0.391655	1.87E-09	6.03E-05
Hsd3b7	0.391371	3.31E-10	1.07E-05
Cox6c	0.391369	3.12E-11	1.01E-06
Rpl15	0.3911	4.24E-11	1.37E-06
Cox4i1	0.389911	2.5E-14	8.09E-10
Cox5a	0.38915	7.66E-11	2.47E-06
Lamp1	0.389029	6.39E-11	2.06E-06
Cycs	0.388945	4.12E-09	0.000133

Cd86	0.388157	9.32E-07	0.030097
Ndufb5	0.388021	6.27E-09	0.000202
Chmp4b	0.386225	2.55E-10	8.25E-06
Gpi1	0.385082	8.7E-09	0.000281
Ppt1	0.38422	1.09E-06	0.035031
Cd200r1	0.383146	6.18E-16	1.99E-11
Pmf1	0.382105	1.49E-15	4.82E-11
Plaat3	0.38194	3.64E-10	1.17E-05
Eci2	0.38158	5.3E-08	0.001711
Pnp	0.381328	1.4E-08	0.000453
Lrrc59	0.380953	9.45E-17	3.05E-12
Rps6	0.380901	2.91E-11	9.39E-07
Elob	0.379229	6.52E-12	2.1E-07
Comtd1	0.378563	7.46E-15	2.41E-10
Pfkl	0.378133	2.87E-11	9.25E-07
Srsf3	0.377963	3.63E-07	0.011712
Per1	0.377475	8.1E-11	2.61E-06
Ndufs5	0.377411	2.01E-09	6.5E-05
Cnih4	0.377255	1.05E-08	0.000339
Ccrl2	0.376943	2.62E-05	0.845735
Timm23	0.376768	2.26E-13	7.31E-09
Vdr	0.376315	5.22E-18	1.68E-13
C5ar1	0.375183	1.17E-13	3.77E-09
Cep83	0.375069	7.55E-12	2.44E-07
Mrps36	0.374627	1.05E-12	3.4E-08
Eif3i	0.374261	2.08E-09	6.72E-05
Hsp90b1	0.373952	2.76E-06	0.088947
Rexo2	0.373117	9.21E-10	2.97E-05
Timd4	0.37297	2.34E-05	0.756099
Cox7a2	0.372489	8.58E-12	2.77E-07
Pdia6	0.372023	8.2E-07	0.026462
Hspa9	0.371947	2.31E-11	7.44E-07
H2afz	0.371671	1.36E-08	0.000439
Cyp4v3	0.371634	4E-10	1.29E-05
H2-Q2	0.371594	2.16E-06	0.069887
Ifitm1	0.370186	0.008746	1
Cped1	0.369529	1.09E-15	3.51E-11
Txndc17	0.369338	2.43E-10	7.83E-06
Tmem173	0.368539	4.65E-11	1.5E-06
Ppp1r14b	0.368327	1.85E-17	5.97E-13
Ndufa5	0.367659	8.43E-10	2.72E-05
Socs2	0.367607	1.6E-18	5.17E-14
Nucb1	0.365227	3.23E-09	0.000104
Tma16	0.364698	4.12E-17	1.33E-12
Cyc1	0.364329	1.32E-07	0.004265
Gm4258	0.364265	8.19E-13	2.64E-08
Magohb	0.363486	7.57E-08	0.002445
Ndufb9	0.363087	7.48E-09	0.000241

Ndufab1	0.362556	2.66E-10	8.57E-06
Swi5	0.358316	8.9E-06	0.287215
Grina	0.357951	6.61E-05	1
Ptms	0.357418	4.58E-10	1.48E-05
Cish	0.357102	2.5E-17	8.06E-13
Ptgs1	0.356898	1.39E-13	4.49E-09
Idh3a	0.356447	8.66E-15	2.8E-10
Pa2g4	0.355868	1.24E-10	4.02E-06
Saraf	0.355657	6.4E-07	0.020663
Il1a	0.355345	3.76E-09	0.000121
Ciita	0.355297	1.01E-08	0.000325
Cyba	0.35445	6.19E-11	2E-06
Clic5	0.354343	4.82E-11	1.56E-06
Uqcr11	0.3541	5.2E-06	0.167878
Mdh2	0.354018	8.51E-09	0.000275
1110008P1	0.353903	5.42E-07	0.01749
Hmgb1	0.353581	3.66E-06	0.118103
Ccl9	0.352616	6.39E-05	1
Ssr4	0.352275	3.39E-10	1.1E-05
Colgalt1	0.352078	6.08E-11	1.96E-06
Cd40	0.351929	1.86E-10	6.01E-06
Pbx1	0.351691	3.11E-05	1
Ftl1	0.351501	1.03E-14	3.33E-10
Ptgs2	0.351499	2.49E-09	8.04E-05
Gna13	0.351399	9.94E-10	3.21E-05
Furin	0.351305	3.67E-09	0.000119
Vps29	0.350817	1.95E-09	6.31E-05
Higd1a	0.350588	2.94E-10	9.5E-06
Krtcap2	0.350484	7.63E-08	0.002464
Ltb4r1	0.349932	9.24E-07	0.029843
Snrpd2	0.349453	3.08E-10	9.95E-06
Hvcn1	0.349362	2.2E-13	7.1E-09
Nrg2	0.348996	8.48E-14	2.74E-09
Flt1	0.348099	6.23E-07	0.02011
Mrpl13	0.347341	1.34E-13	4.34E-09
Mrps28	0.34716	4.05E-13	1.31E-08
Tbc1d2	0.346627	1.14E-13	3.68E-09
Trem2	0.346148	4.33E-10	1.4E-05
Pdcd6	0.345778	2.89E-10	9.34E-06
Dnajc15	0.345106	3E-08	0.000968
Sectm1a	0.344769	1.43E-17	4.61E-13
Rab20	0.343807	9.46E-11	3.05E-06
Got2	0.343807	8.72E-07	0.028151
Stx3	0.34359	1.72E-15	5.56E-11
Gm10260	0.343359	3.27E-08	0.001057
Tomm20	0.343035	6.98E-08	0.002254
Fbxo6	0.342751	1.48E-11	4.76E-07
Nfia	0.342506	2.15E-10	6.94E-06

C1rl	0.342252	1E-13	3.23E-09
Lrp12	0.342216	1.31E-15	4.23E-11
Rbpj	0.341154	3.51E-08	0.001132
Uap1l1	0.341103	8.47E-15	2.73E-10
Psma4	0.34108	2.96E-08	0.000956
AI506816	0.340456	9.11E-11	2.94E-06
Npm1	0.340287	1.38E-05	0.446962
Mpv17l2	0.340235	3.95E-12	1.27E-07
Mrpl12	0.338632	3.35E-10	1.08E-05
Rnh1	0.337095	1.23E-07	0.003956
Ski	0.337068	6.17E-12	1.99E-07
Hcar2	0.33683	2.73E-11	8.83E-07
Tnfaip3	0.336319	3.95E-07	0.012749
Ranbp1	0.33591	1.7E-07	0.005494
Hspe1	0.335848	1.58E-06	0.05099
Ms4a6d	0.335712	1.17E-07	0.003774
Psmb2	0.335576	1.79E-08	0.000577
Plgrkt	0.334249	5E-06	0.161542
Psmb6	0.332601	1.63E-08	0.000528
Cope	0.332067	8.17E-07	0.026373
Eno1b	0.331909	3.47E-09	0.000112
Ly6c2	0.331318	1.03E-06	0.033143
Mrpl42	0.331049	1.14E-09	3.68E-05
Tmed2	0.329849	8.07E-08	0.002606
Olfm1	0.329314	3.67E-12	1.18E-07
Cpq	0.329171	3.15E-09	0.000102
Rpl23a	0.32884	2.9E-07	0.009357
Il1b	0.328159	0.270517	1
Nudt19	0.327899	8.82E-10	2.85E-05
Fuca1	0.327274	2.03E-06	0.065646
Rpl29	0.325392	5.74E-10	1.85E-05
Tmem14c	0.325388	4.1E-08	0.001323
Cct6a	0.324904	8.22E-07	0.026532
Gnaq	0.324333	2.23E-07	0.007199
Psmb4	0.324131	6.02E-09	0.000194
Cenpb	0.322968	4.43E-12	1.43E-07
Selenom	0.322821	1E-07	0.00324
Ddt	0.321516	1.25E-10	4.05E-06
Pla2g7	0.319638	0.00038	1
Rpl13	0.319373	1.73E-10	5.58E-06
Cd200r4	0.319085	6.67E-13	2.15E-08
Ndufa8	0.317572	3.09E-07	0.009964
Eva1b	0.317539	2.31E-14	7.46E-10
Atp5f1	0.316593	5.29E-07	0.017069
Gde1	0.316259	1.99E-09	6.42E-05
Rpl22l1	0.315446	4.88E-05	1
Hk1	0.314408	1.98E-06	0.063783
Tgm2	0.313266	0.024184	1

Cd33	0.31296	6.31E-17	2.04E-12
Def8	0.312218	2.77E-09	8.95E-05
Fam162a	0.311802	1.12E-09	3.62E-05
Fcgr1	0.311207	1.05E-08	0.000339
Adam8	0.310694	1.06E-06	0.034136
Cebpa	0.310667	8.43E-08	0.002722
Clec4a2	0.30915	4.02E-05	1
Calhm6	0.307838	7.23E-06	0.23357
Hscb	0.307482	3.21E-07	0.010352
Prdx4	0.307404	1.64E-10	5.29E-06
Rpl31	0.306751	4.9E-07	0.015811
Phf11b	0.306356	1.26E-05	0.40673
Ly86	0.306296	0.000122	1
Pitpna	0.306002	3.56E-07	0.011487
Zfp804a	0.305983	1.66E-06	0.053567
Slc29a3	0.305792	2.47E-10	7.97E-06
Sash1	0.303695	1.59E-08	0.000513
Lgals3	0.302511	7.16E-06	0.231233
Hipk2	0.302269	8.74E-07	0.028215
Nenf	0.301804	6.13E-14	1.98E-09
Snx10	0.301656	1.79E-06	0.05793
Adap2	0.301524	3.47E-12	1.12E-07
Cited2	0.301329	3.32E-06	0.107102
Atp6v1g1	0.300841	5.08E-07	0.016392
Vcp	0.30075	3.72E-05	1
Tapbp	0.299708	2.63E-05	0.850149
Pgd	0.298826	4.3E-07	0.013896
Rplp2	0.298139	3.66E-10	1.18E-05
Dnase2a	0.297865	3.29E-07	0.01062
Lrpap1	0.297564	6.75E-07	0.02178
Irgm1	0.29697	1.13E-05	0.363824
Cox7a2l	0.29693	0.000466	1
Snrpc	0.296792	1.13E-06	0.036501
Gsn	0.296748	4.02E-05	1
Cmklr1	0.296691	2.85E-10	9.2E-06
Lamtor1	0.295373	0.000156	1
Ndst1	0.295069	4.83E-11	1.56E-06
Tmem256	0.294446	1.27E-06	0.041032
Ptafr	0.294361	1.92E-09	6.18E-05
Fam20c	0.294181	8.28E-11	2.67E-06
Hspa5	0.293924	0.000941	1
Pvt1	0.293018	4.69E-10	1.51E-05
Nrip1	0.292875	1.1E-06	0.035612
Arf3	0.292818	1.26E-07	0.004082
Aco2	0.292233	6.33E-09	0.000204
Rps26	0.291787	1.95E-07	0.006303
S100a6	0.291483	0.000119	1
Junos	0.291417	3.61E-08	0.001167

Basp1	0.291206	4.49E-05	1
Stx7	0.291072	1.37E-05	0.442586
Rpn2	0.290699	1.1E-06	0.035355
Pgls	0.290454	6.42E-05	1
Mtln	0.288536	1.07E-15	3.46E-11
Cacybp	0.288522	1.64E-07	0.005293
Slc25a25	0.287553	2.21E-09	7.14E-05
1110038F1	0.28731	2.41E-09	7.79E-05
Ubald1	0.286808	0.000384	1
Hint1	0.28674	1.04E-05	0.335264
Eef1g	0.286507	5.82E-06	0.188004
Txn2	0.286436	1.88E-07	0.00608
Rps12	0.286241	7.31E-07	0.023585
Psmd7	0.28542	1.56E-09	5.03E-05
Sdhc	0.283901	8.72E-12	2.82E-07
Il12rb1	0.283641	2.8E-17	9.03E-13
Sdad1	0.282985	6E-07	0.019384
Timm13	0.282814	4.32E-07	0.013945
Ncl	0.282728	4.31E-05	1
Ndufv2	0.282523	4.96E-05	1
Rpl3	0.281949	3.16E-07	0.010208
Ifit2	0.281561	0.030914	1
Fbl	0.281516	1.11E-06	0.035818
Rnf217	0.2806	9.45E-13	3.05E-08
Ntan1	0.280552	2.85E-07	0.00921
Tomm40	0.28035	8.89E-10	2.87E-05
Vps54	0.28009	1.32E-07	0.004263
Parvg	0.279385	3.45E-08	0.001115
Itga5	0.279132	2.76E-07	0.008895
Eif4a1	0.278944	0.000212	1
Btf3	0.278917	1.17E-06	0.037844
Nus1	0.278688	1.05E-08	0.000338
Antxr2	0.278425	3.7E-06	0.119383
Dnase1l3	0.278231	1.99E-06	0.064162
Rwdd1	0.277952	2.61E-06	0.084249
Il2rg	0.277573	0.001203	1
Tbcb	0.277436	5.33E-05	1
H2-T22	0.276161	0.000568	1
Slc4a8	0.276159	3.74E-15	1.21E-10
Slfn4	0.275126	7.51E-05	1
Vcan	0.27474	0.000404	1
Reep3	0.274347	2.48E-06	0.080179
Bcl2l11	0.274267	3E-09	9.7E-05
Rtraf	0.273121	5.22E-06	0.168526
Odc1	0.273019	2.79E-13	9.01E-09
Sidt2	0.272658	3.97E-11	1.28E-06
Eif1a	0.272377	3.97E-07	0.012825
Chchd1	0.272193	8.04E-05	1

Pclaf	0.272116	5.74E-05	1
Serp1	0.272063	0.000704	1
Ptma	0.271933	0.00015	1
Rps25	0.271853	4.32E-07	0.013934
Dst	0.271701	1.11E-05	0.358197
Abhd16a	0.271355	1.84E-08	0.000594
Cemip2	0.271352	9.44E-10	3.05E-05
Mkrn1	0.270217	5.27E-05	1
Mxd1	0.269748	2.26E-06	0.073028
Mtx1	0.269727	3.06E-07	0.009893
Ndufv1	0.269122	3.91E-08	0.001262
Cotl1	0.269109	8.66E-05	1
Tmem86a	0.268847	1.13E-07	0.003642
Tspan3	0.268736	7.05E-11	2.28E-06
Hdlbp	0.26864	3.48E-05	1
Snx24	0.268495	5.25E-15	1.7E-10
Aldoc	0.268179	2.82E-11	9.12E-07
mt-Nd2	0.268152	2.59E-05	0.837504
Mfsd1	0.268125	6.4E-06	0.206516
Ddost	0.267922	5.45E-08	0.001759
Fundc2	0.267858	1.38E-10	4.46E-06
Tmem123	0.267731	3.71E-05	1
Chd7	0.267642	5.54E-05	1
Tap2	0.267454	0.000179	1
Rai14	0.267362	1.59E-10	5.13E-06
Eif6	0.267213	1.05E-06	0.033993
Cerk	0.267007	1.72E-06	0.055549
Mrap	0.266282	6.04E-08	0.001951
D5Ertd579	0.26618	1.92E-06	0.062101
AI413582	0.266176	0.00034	1
Psme1	0.266108	2.04E-06	0.065705
Junb	0.265603	0.004478	1
Net1	0.26545	1.27E-07	0.004099
Slc52a3	0.265238	9.44E-12	3.05E-07
Ap1s1	0.265225	2.99E-07	0.009653
Larp1	0.26439	2.35E-07	0.007595
Tex264	0.264094	4.01E-11	1.29E-06
Aff1	0.263565	5.61E-08	0.001812
Cd80	0.263527	2.82E-09	9.1E-05
Tubb6	0.263207	4.89E-11	1.58E-06
Klrk1	0.263146	6.51E-06	0.210192
Slc31a1	0.26291	2.24E-07	0.007246
Atp5j2	0.262712	6.47E-05	1
Rps15	0.262655	8.46E-06	0.273094
Ube2j2	0.262429	7.38E-06	0.238184
Batf2	0.261999	2.94E-10	9.5E-06
Psma2	0.261934	4.69E-05	1
Rab5if	0.261901	8.2E-08	0.002647

Akap7	0.26089	6.94E-07	0.02242
Etfb	0.26045	7.41E-05	1
Hk2	0.260406	1.56E-06	0.050468
Ripk3	0.260166	8.63E-11	2.79E-06
Mafb	0.260041	0.000868	1
Uqcc2	0.26004	2.51E-06	0.081094
Txndc5	0.259622	1.46E-09	4.7E-05
Xbp1	0.259612	0.000114	1
Mrpl2	0.259011	6.05E-05	1
mt-Nd4	0.259001	0.000177	1
Nutf2	0.258424	4.37E-09	0.000141
Tspan4	0.258378	9.77E-13	3.16E-08
Pim3	0.258043	1.98E-09	6.39E-05
Echs1	0.256359	3E-07	0.009686
Tmem147	0.255994	2.9E-06	0.093782
Tap1	0.25581	0.000196	1
Eif3c	0.255598	0.000569	1
Gadd45g	0.255512	1.43E-07	0.004619
Csnk2b	0.255384	0.000105	1
Rpl23	0.255191	1.21E-06	0.038987
Rpn1	0.254919	5.29E-06	0.170898
Timm10	0.254832	9.7E-09	0.000313
Itgam	0.254192	0.005232	1
Gm15283	0.253928	8.09E-08	0.002611
Rpl7a	0.253589	3.67E-07	0.011846
Mtch2	0.253019	1.01E-07	0.003258
Timm8b	0.252495	0.000287	1
Slc20a1	0.251829	3.71E-08	0.001198
Nceh1	0.251628	4.66E-09	0.000151
Ralgds	0.251392	3.18E-11	1.03E-06
Slirp	0.251062	3.86E-06	0.124693
Atp5l	0.250481	1.42E-05	0.459326
Tle5	0.250475	0.0001	1
Micos13	0.250304	9.47E-07	0.030572
Nolc1	0.250292	4.25E-05	1
Ftl1-ps1	0.249988	6.04E-08	0.00195
Polr2i	0.249812	5.88E-07	0.018982
Hnrnpab	0.249438	0.000468	1
Gbp9	0.24879	4.8E-05	1
1700017B(	0.248286	1.1E-10	3.56E-06
Rtp4	0.247928	2.29E-06	0.073914
Syngr2	0.247765	0.003961	1
Clta	0.247705	7.06E-06	0.227876
Ybx1	0.247514	7.75E-05	1
Drap1	0.247474	2.02E-06	0.065181
Rgcc	0.247317	7.45E-08	0.002405
Nudt21	0.247209	5.44E-06	0.175514
Tnfaip2	0.247058	0.000182	1

Prtn3	0.246871	3.64E-08	0.001175
Ksr1	0.246714	6.61E-09	0.000213
Myc	0.246087	7.61E-06	0.245763
Fkbp1a	0.245528	4.21E-05	1
Cass4	0.245467	0.000304	1
Klrb1b	0.24546	2.1E-06	0.067774
Pdia4	0.24545	2.53E-10	8.15E-06
Htt	0.245133	1.63E-07	0.005253
Comt	0.245015	5.33E-07	0.01722
Ncf1	0.244648	3.48E-05	1
Slc25a39	0.244584	5.29E-07	0.017068
Mrc1	0.244209	2.58E-11	8.34E-07
Prkca	0.243959	3.52E-07	0.01138
Thyn1	0.243788	1.14E-12	3.69E-08
Aifm2	0.243295	1.01E-10	3.26E-06
Abhd17a	0.243256	2.05E-05	0.663121
Smarcb1	0.243047	5.55E-07	0.017925
Cfl1	0.242919	1.3E-06	0.041855
Ncln	0.242366	8.67E-07	0.027993
Gabarap	0.241994	2.16E-07	0.006978
Gusb	0.241939	0.000139	1
Banf1	0.241364	2.71E-05	0.874577
Phb	0.241338	4.23E-07	0.013641
Svbp	0.241245	1.18E-07	0.0038
Arf5	0.240734	4.3E-06	0.138724
Tbca	0.240679	5.21E-05	1
Ndufa11	0.240409	1.13E-05	0.365805
Hspd1	0.240015	3.24E-05	1
Dapk1	0.23991	6.35E-05	1
Snrpd3	0.239606	1.31E-05	0.422388
Atp5h	0.239529	8.47E-06	0.273557
4930430E1	0.239385	2.99E-13	9.65E-09
Tmem189	0.239384	1.01E-08	0.000327
Gm50237	0.239296	2.62E-13	8.47E-09
Tor3a	0.239023	1.57E-05	0.506139
Hnrnpa1	0.238969	0.000209	1
Eif4ebp1	0.23895	0.005636	1
Rgl1	0.238825	0.000104	1
Ndufb10	0.238704	3.48E-05	1
Mrpl20	0.238517	0.000186	1
Rap2c	0.238431	7.72E-05	1
Pgm1	0.238339	8.71E-10	2.81E-05
Stx4a	0.238014	0.000119	1
Ppil1	0.237579	0.000859	1
Rgs1	0.237188	2.43E-06	0.078372
Ric1	0.237028	1.49E-08	0.000483
H2afy	0.23682	0.000179	1
Mrps33	0.236756	1.12E-05	0.361435

Cfap410	0.236737	2.87E-07	0.009268
Eef1d	0.236458	0.000107	1
Tmem192	0.236437	1.48E-06	0.047711
Spcs1	0.235854	3.36E-05	1
Mrpl11	0.235537	1.71E-07	0.005525
Snap29	0.235391	1.76E-05	0.567447
Rtcb	0.235366	0.000159	1
Pnrc1	0.235251	0.000436	1
Nubp1	0.235159	1.23E-05	0.395772
Sdc3	0.234885	1.42E-05	0.457522
Mrps16	0.234826	1.15E-06	0.036989
Il15ra	0.234729	5.87E-08	0.001894
Arhgap31	0.234555	3.88E-05	1
Plod1	0.234111	9.84E-09	0.000318
Atg5	0.234084	1.6E-06	0.051714
Ranbp2	0.233696	5.49E-05	1
Renbp	0.233694	2.93E-10	9.46E-06
Tlr12	0.233013	4E-12	1.29E-07
Gnl3	0.232785	1.3E-09	4.2E-05
Cacna1d	0.23277	0.000131	1
Creld2	0.231819	1.7E-09	5.5E-05
Vcam1	0.231665	4.01E-06	0.129388
Slc16a6	0.231547	2.53E-10	8.18E-06
Yif1b	0.231246	5.81E-09	0.000188
Pik3r6	0.230867	4.55E-06	0.14681
Cct8	0.230636	0.0008	1
Sulf2	0.23045	1.4E-05	0.451088
Apex1	0.230246	4.95E-09	0.00016
Fbxw17	0.229941	1.55E-09	5E-05
Psmd8	0.229917	0.00139	1
Ndufc2	0.229854	5.35E-07	0.017262
Edf1	0.229794	0.000169	1
Impa2	0.229783	1.99E-08	0.000641
Mrpl54	0.229677	4.08E-07	0.013172
Coro2a	0.229607	0.000396	1
Trim25	0.229575	0.002706	1
mt-Nd3	0.229387	7.42E-05	1
Sys1	0.229382	0.000114	1
Bri3	0.22936	1.51E-07	0.004889
Bcat2	0.228614	4.03E-08	0.0013
Peak1	0.228588	2E-05	0.645478
Pip5k1c	0.228481	2.58E-06	0.083263
Siglec1	0.22826	3.24E-10	1.05E-05
Cdca8	0.228184	1.87E-07	0.006043
Adgb	0.228132	8.29E-07	0.026762
Ndufb2	0.228125	2.51E-05	0.809075
Ergic1	0.228073	7.29E-09	0.000235
Dennd1b	0.228042	0.000418	1

Ybx3	0.227788	1.12E-07	0.003605
Cmc1	0.227761	0.000295	1
Plxnd1	0.227657	1.54E-10	4.98E-06
Slc25a4	0.227539	1.05E-07	0.003374
Fcgrt	0.227523	1.55E-06	0.049971
Gpx3	0.227237	4.36E-08	0.001408
Rpl4	0.227223	1.24E-05	0.399754
Icam1	0.227104	0.005487	1
Galnt7	0.227083	2.02E-09	6.51E-05
Rab7	0.227041	0.000145	1
Plekho1	0.22608	1.7E-08	0.000549
Iscu	0.226024	0.015944	1
Lrrc58	0.225749	6.11E-06	0.19717
Psmb3	0.225648	8.3E-05	1
Anapc11	0.225599	0.000322	1
Actg1	0.225095	4.71E-07	0.015192
Mapk6	0.225074	7.29E-07	0.023521
Itgb1bp1	0.225025	1.34E-07	0.004322
Cenpa	0.224957	0.006964	1
Rps8	0.224935	6.68E-05	1
Eef1b2	0.224505	0.000164	1
9130401M	0.224469	2.89E-05	0.93364
Ubb	0.224403	1.37E-06	0.044324
Herc6	0.224387	0.000352	1
Rmdn3	0.224191	1.02E-09	3.29E-05
Surf4	0.224028	1.02E-05	0.330135
Emg1	0.223999	1.34E-06	0.043225
Hivep3	0.22398	4.83E-06	0.155824
A930007I1	0.223977	8.53E-09	0.000275
Mlkl	0.223281	1.39E-08	0.000449
Rpl27	0.22323	3E-06	0.096702
Slc16a3	0.22271	0.002674	1
Spcs2	0.222703	0.000443	1
Mrpl35	0.222478	2.18E-09	7.05E-05
Hist1h1e	0.222152	0.065817	1
Ndufa2	0.222093	0.000319	1
Kif1b	0.221901	4.63E-06	0.149501
Fdx2	0.221824	1.23E-07	0.003968
1700037H(	0.221364	2.85E-05	0.920396
Abhd11	0.221221	1.1E-07	0.003564
Suclg1	0.221169	1.86E-06	0.059921
Rpl17	0.221034	0.0004	1
Scpep1	0.221028	3.94E-05	1
Snu13	0.220895	8.09E-05	1
Dut	0.220887	8.05E-06	0.259975
Nudcd2	0.220878	1.45E-06	0.046737
Pfdn1	0.220813	1.45E-05	0.466829
Nagk	0.220484	5.02E-07	0.016202

Ttc39c	0.220445	1.04E-06	0.033483
Batf3	0.220407	2.32E-05	0.750374
Mrps23	0.220167	1.45E-07	0.004684
Rabggtb	0.219694	5.86E-05	1
Birc5	0.219625	3.71E-05	1
Zdhhc4	0.219507	0.000293	1
Hsp90ab1	0.219483	2.4E-05	0.774964
Cd302	0.218934	0.000115	1
Rpl24	0.218629	0.000154	1
Fkbp1b	0.218537	0.001936	1
Heatr1	0.218012	6.49E-09	0.000209
Bsg	0.217307	1.41E-05	0.45439
Ifi203	0.217266	4.53E-06	0.146202
Tmed10	0.217145	2.5E-05	0.807863
Anapc15	0.216585	2.4E-08	0.000774
Nudt3	0.216169	1.15E-08	0.000371
Cpne2	0.216	1.31E-12	4.22E-08
Tmem140	0.215796	0.000306	1
Desi2	0.215686	2.02E-07	0.006534
Oxct1	0.215632	6.14E-06	0.198173
Il12rb2	0.215474	1.49E-06	0.048194
Il1rl2	0.215298	1.52E-11	4.9E-07
Mdfic	0.215132	3.51E-07	0.011316
Cd151	0.215092	8.35E-07	0.02695
Mdm2	0.214952	0.001521	1
Prdx1	0.214828	1.21E-05	0.390619
Smc2	0.214688	2.28E-05	0.7372
Fmn1	0.214265	7.82E-07	0.025242
Dmxl2	0.214157	2.11E-08	0.000682
Cnih1	0.214008	2.68E-06	0.086408
Esyt2	0.213665	4.6E-06	0.148527
Rpl28	0.213271	6.56E-05	1
Vat1	0.213218	5.42E-07	0.017502
Gm12865	0.213216	6.23E-10	2.01E-05
Pmvk	0.212944	5.83E-08	0.001881
Esd	0.212664	1.46E-05	0.470954
Usp14	0.212651	4.91E-07	0.015865
Mroh1	0.212238	1.73E-08	0.000558
Cd93	0.212235	0.000434	1
Hadh	0.212219	4.88E-07	0.015743
Kars	0.212209	1.21E-07	0.003894
Spg21	0.211706	2.75E-05	0.887935
Rps5	0.211589	0.000164	1
Aph1a	0.211389	0.000174	1
Stard3nl	0.210884	1.25E-07	0.004024
Cct3	0.210754	1.5E-05	0.485607
Adam9	0.210684	3.57E-06	0.115144
Rab5c	0.210391	8.31E-05	1

Sec13	0.210306	0.000495	1
Prpf31	0.210282	6.83E-08	0.002206
Wwox	0.210091	2.91E-05	0.938418
Aig1	0.210081	4.02E-05	1
Rad23a	0.209873	1.1E-05	0.356499
Osm	0.209315	7.15E-08	0.00231
Mid1	0.209236	2.87E-08	0.000926
Psmd2	0.209211	0.001492	1
1110038B1	0.208935	2.18E-06	0.070383
Abracl	0.208869	0.001421	1
Lsm7	0.208743	5.91E-05	1
Plxna1	0.208412	1.93E-10	6.23E-06
Pla2g4a	0.20834	0.002646	1
Myo7a	0.20822	4.83E-07	0.015581
Gfer	0.207781	2.45E-07	0.007924
Mrps18a	0.207434	0.000124	1
Nup93	0.207379	8.44E-07	0.027262
Usp18	0.207204	2.61E-05	0.843723
Ptpn7	0.207029	5.82E-07	0.018788
Eif2b2	0.206917	0.000129	1
Tcp11l1	0.206739	3.19E-07	0.010288
Paics	0.206668	7.41E-06	0.239102
Gas2l1	0.206165	8.95E-10	2.89E-05
Tusc3	0.205725	1.66E-07	0.00536
Mvb12b	0.205566	3.7E-08	0.001195
Cdk4	0.205207	1.4E-05	0.453571
Egr2	0.205122	2.56E-07	0.008255
Fuom	0.204935	9.18E-10	2.96E-05
Khk	0.204618	8.57E-06	0.276545
Wdr83os	0.204307	9.64E-05	1
Cks1b	0.203928	4.64E-05	1
Gars	0.203486	8.42E-05	1
Cenpw	0.203397	6.33E-08	0.002044
Tes	0.20324	2.91E-05	0.939403
Gpr171	0.203161	2.18E-09	7.04E-05
Lamtor2	0.203127	0.002825	1
Ndufs7	0.202888	1.81E-05	0.585709
Uqcrfs1	0.202846	1.67E-05	0.538668
Rnf141	0.202809	6.52E-08	0.002106
Uqcrc2	0.202676	0.012958	1
Ehd1	0.202553	0.003286	1
Taf10	0.20235	8.31E-06	0.268378
Rplp0	0.202171	8.61E-05	1
Lrpprc	0.201897	3.61E-06	0.116611
Pkig	0.201859	6.31E-07	0.020365
Nrg1	0.201701	1.55E-07	0.004989
Mycbp2	0.201562	0.002509	1
Abcg1	0.201009	3.13E-05	1

Rps7	0.200986	0.000313	1
Dnajb11	0.200984	1.44E-05	0.466459
Impdh1	0.200901	3.35E-08	0.001081
Jdp2	0.200891	1.89E-08	0.00061
Lmnb1	0.20087	7.99E-05	1
Nans	0.200774	7.27E-06	0.234685
Rer1	0.200605	5.51E-05	1
Srp9	0.200573	0.000354	1
B3gnt2	0.200498	4.65E-09	0.00015
Spink2	0.20034	3.27E-07	0.010562
Rgmb	0.200338	2.22E-06	0.07164
Naxe	0.200267	0.000221	1
Acat1	0.200207	2.09E-06	0.067623
Nsd2	0.200156	1.5E-10	4.85E-06
Dnpep	0.200143	5.01E-07	0.01618
Tnfsf12	0.200011	3.99E-05	1
Znrd2	0.199368	5.98E-09	0.000193
Cyb5r3	0.199141	3.44E-06	0.111112
Tmod3	0.198291	0.000284	1
Plau	0.197988	5.27E-09	0.00017
Psmg4	0.197762	1.07E-05	0.346235
Ndufs6	0.197753	8.84E-05	1
Metap2	0.197501	9.13E-07	0.029482
Inpp5j	0.197034	1.79E-08	0.000579
Naa25	0.196964	2.11E-06	0.068239
Plekha1	0.196927	2.29E-07	0.007392
Pgap2	0.196327	0.004757	1
Timp2	0.195925	6.08E-07	0.01962
Sestd1	0.195883	3.18E-08	0.001026
Atg3	0.195869	6.49E-06	0.209414
Sra1	0.195844	0.000928	1
9430038I0	0.195773	9.68E-08	0.003125
Mrpl38	0.19521	5.63E-07	0.018172
Hmbs	0.195098	4.24E-05	1
Gmnn	0.194852	1.93E-06	0.062417
Atp5md	0.194731	0.0002	1
AI662270	0.194712	0.000181	1
Prm1	0.194679	2.77E-13	8.93E-09
Ube2s	0.194147	0.001052	1
Tns1	0.194083	4.45E-07	0.014353
Eif3m	0.19403	0.00061	1
Set	0.193993	0.000457	1
Lman2	0.193885	0.000938	1
Atp13a2	0.1938	2.52E-06	0.081504
Gtf2h5	0.193641	0.000135	1
Rnps1	0.193274	0.001284	1
Kdm3a	0.193081	5.61E-08	0.001811
Rnasel	0.193026	2.6E-06	0.083877

Mrps14	0.192983	0.001325	1
Acp1	0.192959	4.12E-05	1
Rps10	0.192596	0.0001	1
Impdh2	0.192563	7.2E-07	0.023232
Rasa4	0.19247	8.98E-05	1
Naa20	0.192325	4.07E-08	0.001315
Eng	0.192319	3.91E-06	0.126221
Cnpy2	0.192222	0.001017	1
Pygl	0.191899	0.000458	1
Tmbim1	0.191558	1.78E-05	0.573153
Snhg6	0.191555	2.44E-07	0.007865
Slc36a1	0.191457	0.000111	1
Sult1a1	0.191243	2.9E-05	0.937116
2900097C1	0.19124	1.76E-06	0.056982
Insl6	0.191167	5.78E-10	1.87E-05
Coq7	0.190939	8.47E-10	2.73E-05
Uhrf1bp1	0.19093	4.27E-09	0.000138
Gyg	0.190879	0.003523	1
Srpr	0.190738	0.000616	1
Ccnb2	0.190705	2.92E-05	0.943933
Smn1	0.190408	9.95E-07	0.032123
Psmb8	0.190403	2.69E-05	0.86995
Lactb	0.190079	7.19E-07	0.023221
Psmb1	0.190026	0.000456	1
Exoc3l4	0.189787	4.87E-12	1.57E-07
Canx	0.189517	0.001138	1
Bola3	0.189427	7.93E-08	0.002561
Mdn1	0.189302	1.17E-07	0.003783
H2-Q1	0.189057	9.33E-05	1
Gm42962	0.188999	7.98E-09	0.000258
Reep5	0.188891	0.00446	1
Prelid3b	0.188607	3.12E-05	1
Atp5e	0.188508	0.000219	1
Gm46563	0.188458	1.38E-08	0.000447
Sbf2	0.188445	3.89E-08	0.001255
Gar1	0.188331	1.04E-06	0.033534
Eid1	0.188287	1.57E-06	0.050669
Cpped1	0.188139	1.03E-07	0.003328
Ostc	0.188032	0.001706	1
Fgd6	0.187792	9.24E-14	2.98E-09
Cox7c	0.187778	0.001112	1
Idh3g	0.187495	6.94E-05	1
Noc2l	0.187447	3.61E-05	1
Ifi44	0.18739	1.7E-06	0.05479
Galk1	0.187247	4.59E-09	0.000148
Cyth1	0.18718	0.001463	1
Dnajc5	0.18714	0.000305	1
Aarsd1	0.187086	1.04E-05	0.335534

Slc23a2	0.186852	6.98E-06	0.225212
Rps18	0.186613	0.00171	1
Rhoc	0.18642	1.7E-06	0.054937
Marcksl1	0.186398	0.00026	1
Dock1	0.186308	5.15E-05	1
Zfas1	0.186078	0.000801	1
Dohh	0.186055	5.41E-09	0.000175
Psmb7	0.185986	0.000162	1
Sec11a	0.185893	2.12E-07	0.006847
Baiap2	0.185791	7.91E-08	0.002553
Carhsp1	0.185658	2.79E-08	0.0009
Wdfy1	0.185356	0.000261	1
Parvb	0.185295	6.77E-07	0.021868
Eprs	0.185081	3.32E-06	0.107204
Sco2	0.185058	1.58E-12	5.09E-08
Commd1	0.185002	0.000553	1
Eif3g	0.184936	0.000698	1
Ssna1	0.184791	0.000396	1
Kcnq1ot1	0.184722	2.88E-06	0.092912
Slamf6	0.184475	1.01E-11	3.25E-07
Bmp2k	0.184469	6.08E-08	0.001962
Inf2	0.184308	0.000195	1
Tmem268	0.184258	2E-07	0.006459
Rsl1d1	0.184178	2.21E-06	0.071211
Trnau1ap	0.18415	2.98E-06	0.09631
Il18rap	0.184081	3.64E-08	0.001174
Egr1	0.18402	0.000146	1
Phf11d	0.183949	2.15E-07	0.006941
Eif3k	0.18337	0.003918	1
Atp5mpl	0.183295	0.000297	1
Prkcsh	0.183204	3.67E-05	1
Acbd6	0.183028	1.37E-05	0.440712
Tnfrsf14	0.182568	1.23E-09	3.98E-05
Ewsr1	0.182445	0.000683	1
Pira2	0.182199	4.24E-05	1
Tax1bp1	0.182132	0.006119	1
Vkorc1	0.182118	2.96E-06	0.095502
Dhrs3	0.182019	6.29E-09	0.000203
Kctd17	0.181275	3.89E-09	0.000126
Casp9	0.181187	0.000273	1
Ubr4	0.180728	0.000986	1
Eef1a1	0.180685	8.38E-05	1
Rps3	0.180503	0.000285	1
P2rx4	0.180275	1.9E-06	0.0615
Tgif1	0.180031	1.71E-06	0.055166
Lage3	0.179902	1.08E-06	0.034807
Eri3	0.179897	3.77E-06	0.121617
Uqcrc1	0.179863	0.000923	1

Plscr1	0.179388	4.32E-07	0.013955
Rpl8	0.179388	0.00087	1
Selenow	0.179241	0.01417	1
Glrx3	0.179219	0.001681	1
Atp11a	0.179064	3.07E-07	0.009916
Npm3	0.179064	0.000829	1
Rpl10	0.178939	0.000408	1
Rpl19	0.178863	5.29E-06	0.170776
Clybl	0.178818	1.69E-08	0.000546
Prxl2b	0.178789	8.13E-06	0.262387
Smim20	0.17877	0.000416	1
Fndc3b	0.178738	4.39E-05	1
Psmb9	0.178689	0.000774	1
Rsph3a	0.178667	6.93E-05	1
Eif4e2	0.178573	0.000171	1
Nab2	0.178407	3.01E-07	0.009707
Tfg	0.178359	6.78E-05	1
Ndufs8	0.178358	0.000138	1
Tpx2	0.178275	0.000744	1
Mrpl15	0.178226	0.000112	1
Asf1b	0.178138	0.000135	1
Hs6st1	0.17812	1.51E-05	0.487256
Aimp2	0.177954	1.95E-06	0.063057
Notch1	0.177439	1.04E-06	0.033503
Camk1	0.177302	0.000389	1
Tnfsf13	0.17714	0.000762	1
Mcfd2	0.177091	9.2E-06	0.296898
Tomm7	0.177077	0.001434	1
Camk2g	0.176977	4.41E-05	1
Hilpda	0.176877	4.89E-05	1
Atp2a2	0.176746	2.61E-05	0.843646
Rpl36a	0.176652	0.001377	1
Crlf2	0.176563	0.078821	1
Eif3b	0.176546	2.01E-05	0.649538
Stx2	0.176525	1.72E-09	5.55E-05
Ubl4a	0.176469	6.12E-07	0.019772
H2-Q10	0.176165	2.76E-05	0.889621
Sec61b	0.176067	0.028981	1
Hist1h2ae	0.175763	0.480869	1
Slc12a7	0.175486	1.6E-07	0.005167
Atp5c1	0.175378	0.001314	1
Ywhah	0.17528	0.001638	1
Crtc3	0.17508	4.98E-07	0.016091
Gas2l3	0.174804	3.07E-11	9.9E-07
Zcchc24	0.174614	4.03E-05	1
Kdelr2	0.174612	0.000694	1
Pno1	0.174562	3.76E-08	0.001215
Dpy19l1	0.174455	0.000801	1

Ece1	0.174376	5.79E-05	1
Mybbp1a	0.174116	3.14E-06	0.101338
Immt	0.173801	4.75E-05	1
Eea1	0.173594	4.4E-05	1
Tubb2a	0.17348	0.000164	1
Wdr62	0.173252	1.44E-11	4.64E-07
Cst7	0.173178	9.23E-05	1
Gltp	0.173067	0.001411	1
Actn1	0.172935	2.6E-08	0.00084
Morrbid	0.172651	0.052795	1
Naca	0.172576	0.001255	1
Kifc3	0.172317	0.003549	1
Cdt1	0.172028	4.66E-05	1
Treml2	0.172005	1.95E-06	0.062861
Mrps15	0.171787	0.029258	1
Gm4841	0.17175	1.39E-08	0.000449
Plpp2	0.17172	6.55E-08	0.002116
Psap	0.171549	0.000109	1
Hs3st3b1	0.171536	9.45E-10	3.05E-05
Mvd	0.171344	1.52E-06	0.049152
Luzp1	0.171143	9.05E-07	0.029231
Edem1	0.170931	7.16E-06	0.23131
Gm7030	0.170834	0.000815	1
Rbis	0.17081	0.000265	1
Gipc1	0.170683	3.16E-06	0.102128
Snx6	0.170501	5.86E-05	1
Farsa	0.170244	2.15E-06	0.069349
Dhx29	0.170222	4.87E-05	1
Rpl26	0.170041	6.04E-05	1
Ets2	0.169493	2.18E-06	0.070321
Csrnp1	0.169486	1.99E-05	0.642625
Cetn3	0.169437	7.99E-05	1
Dynll1	0.169397	0.001544	1
Hcfc1r1	0.169346	0.000104	1
Bub1b	0.169228	4.58E-07	0.014782
Asah1	0.169121	0.000269	1
Qdpr	0.169059	1.57E-07	0.005065
Slc7a8	0.169038	6.39E-05	1
Nip7	0.168861	0.000181	1
Tpt1	0.168848	5.13E-06	0.165498
Phb2	0.168788	0.001282	1
Hint2	0.16871	0.000489	1
Fam3c	0.168599	5.15E-07	0.016626
Sfxn1	0.168569	3.53E-05	1
Ap2s1	0.168563	0.000109	1
Psmc4	0.168319	0.000624	1
Tcp1	0.168297	0.000287	1
Lpxn	0.16825	8.34E-05	1

Mfsd12	0.168007	2.07E-06	0.06696
Mib1	0.167769	5.99E-06	0.193494
Epb41l1	0.167413	1.37E-07	0.004437
Slfn8	0.167315	0.016986	1
Ffar2	0.167287	3.27E-08	0.001055
Nucks1	0.167019	0.002203	1
Pgam5	0.16683	6.37E-07	0.02057
Urm1	0.166557	0.000113	1
Cnbp	0.166549	0.000687	1
Dynll2	0.166494	3.57E-08	0.001153
Anapc5	0.166429	0.002026	1
Wdr75	0.166415	1.46E-05	0.471574
Anln	0.166254	6.74E-06	0.217736
Dstn	0.166051	0.00198	1
Aars	0.166026	4.59E-07	0.014814
Endog	0.165934	1.8E-08	0.00058
Lxn	0.165766	3.76E-07	0.012149
Tmem106a	0.165728	0.001748	1
Slc27a1	0.165642	6.47E-09	0.000209
Arl1	0.165529	0.003446	1
Chchd6	0.16549	1.78E-06	0.057317
Rab3d	0.165399	5.11E-06	0.165116
Ndufa10	0.165202	7.55E-06	0.243726
Glmp	0.165041	0.003186	1
Pomp	0.164985	0.014261	1
Crat	0.164984	3.6E-07	0.011637
Fh1	0.164875	2.04E-05	0.658584
Wdr41	0.164741	2.29E-05	0.738491
Atp5k	0.164641	0.003386	1
Capn5	0.164429	2.3E-06	0.074098
Mmp19	0.164379	2.06E-07	0.006658
M6pr	0.164147	0.003432	1
Mrps24	0.164051	3.79E-06	0.122431
Mapk7	0.164014	6.13E-06	0.197775
Psmd3	0.16398	7.21E-06	0.232708
Rrm1	0.163907	0.002275	1
Mgst3	0.163845	5.63E-07	0.018177
Bcl2l1	0.163713	0.000197	1
Snhg1	0.163501	3.86E-07	0.012462
Srsf6	0.16346	0.017237	1
Lyz2	0.163363	0.00314	1
Rpl5	0.162856	0.001526	1
Nsun2	0.162717	5.66E-05	1
Ndufa9	0.162358	6.19E-05	1
Ccni	0.162332	0.001607	1
Clpp	0.162305	2.27E-07	0.007344
Chchd3	0.162278	5.17E-05	1
BC004004	0.162151	0.001474	1

Timm50	0.162007	3.57E-05	1
Arl6ip4	0.161757	0.000188	1
B2m	0.161652	0.003393	1
2610001J0	0.16114	3.25E-06	0.105002
Skp1a	0.160848	0.004984	1
Hp1bp3	0.160835	0.001697	1
Uqcr10	0.160682	9.92E-05	1
Mcm5	0.160552	0.01501	1
Id2	0.160374	0.05344	1
Pwp1	0.160315	6.4E-06	0.206525
Isyna1	0.160296	0.000261	1
Mrps12	0.160164	0.00118	1
Slc43a2	0.160047	0.001069	1
Myo10	0.160035	4.36E-06	0.140797
Polr2h	0.159977	9.17E-05	1
Dnajc19	0.159898	0.002045	1
Plin3	0.159656	2.85E-05	0.919659
Mis18a	0.159482	0.000771	1
Rnf103	0.159314	0.002001	1
F11r	0.159313	9.64E-05	1
Zfp560	0.158826	5.44E-06	0.175469
Chchd4	0.158797	3.94E-08	0.001272
Dctpp1	0.158779	2.11E-06	0.068124
Casp4	0.158728	0.001213	1
Pdlim7	0.158459	0.002936	1
Ndufb7	0.158458	0.004554	1
Psmd1	0.158339	0.000771	1
Hsbp1	0.158102	0.001305	1
Foxp4	0.158069	5.03E-08	0.001624
Hspa8	0.157979	0.006473	1
Rab2a	0.157967	0.030278	1
Sh3tc1	0.157923	5.88E-07	0.018985
Rtca	0.15792	3.68E-05	1
Entpd1	0.157736	0.012122	1
Ctdnep1	0.15773	1.6E-05	0.516669
Arap2	0.157439	0.000141	1
Ccl3	0.157205	0.114481	1
Zfp593	0.157164	2.87E-06	0.092638
Smdt1	0.157119	0.012947	1
Plbd2	0.156776	0.000681	1
Tmem160	0.156775	5.06E-07	0.016333
Cmtm7	0.156762	0.085436	1
Rbm7	0.156648	0.003309	1
Mrps17	0.156642	5.39E-06	0.17397
Mcm3	0.156502	0.001201	1
Tmem170b	0.156275	1.54E-06	0.049653
Ormdl2	0.15624	0.000889	1
Ube2l3	0.156067	0.002114	1

Smg9	0.155962	2.16E-06	0.069819
Acvr2a	0.155855	3.86E-05	1
Vapa	0.155632	9.37E-05	1
Vamp8	0.155544	0.00023	1
Utp20	0.155309	1.46E-05	0.470763
Snx4	0.154813	0.000297	1
Cisd1	0.154637	0.000122	1
Snx3	0.154631	0.001128	1
H3f3b	0.154513	0.01258	1
Tmem205	0.154458	7.74E-05	1
Bcap29	0.154374	1.56E-05	0.505221
Esyt1	0.154357	0.001665	1
Ero1l	0.154243	1.86E-05	0.601575
Pmpcb	0.154221	6.56E-06	0.211921
Cysltr1	0.154146	2.41E-09	7.77E-05
Lpar1	0.154057	1.01E-05	0.325312
Cct2	0.153992	0.015354	1
Homer1	0.153786	1.13E-05	0.36411
Alkbh7	0.153204	1.98E-07	0.006391
Tmem258	0.15319	0.075426	1
Hnrnpll	0.153167	0.000157	1
Psmc6	0.153025	0.001043	1
Lamp2	0.152962	0.012633	1
Mllt6	0.152938	2.99E-06	0.096452
Ddhd2	0.152837	2.02E-07	0.006518
Txnrd1	0.152533	7.67E-07	0.024749
Rpl36al	0.152342	0.00375	1
Rilpl2	0.152195	0.000268	1
Ksr2	0.152106	4.02E-07	0.012981
Mrps34	0.152079	5.34E-05	1
Pik3ip1	0.152013	4.08E-05	1
Abce1	0.151904	3.54E-06	0.11435
Snrpe	0.151791	0.092047	1
Ccdc34	0.151411	3.01E-07	0.009721
Pdxk	0.151325	2.91E-05	0.939874
Ssr2	0.151133	0.000219	1
Dennd4c	0.150778	0.002532	1
Galnt6	0.150736	8.24E-08	0.00266
Ebp	0.150698	0.000103	1
Phgdh	0.150295	5.3E-05	1
Pes1	0.150282	1.4E-05	0.450729
Nhlrc3	0.150175	0.000136	1
Dbi	0.149918	0.025472	1
Hacd2	0.149918	3.78E-05	1
Mydgf	0.149892	0.000429	1
Rasgrp1	0.149776	9.1E-06	0.293787
Rnaseh2a	0.149574	6.33E-07	0.02043
Gadd45b	0.149475	0.018397	1

Adh5	0.149364	4.78E-05	1
Tuba1a	0.149254	0.002101	1
Guk1	0.149062	5.09E-09	0.000164
Npepl1	0.149	3.29E-06	0.106159
Yipf1	0.148901	0.000336	1
Fuca2	0.148836	1.29E-05	0.417498
Zbtb8os	0.148811	0.000426	1
Ift43	0.148361	1.41E-08	0.000456
H2-Q7	0.148301	0.007092	1
Adprh	0.148283	0.000989	1
Dip2b	0.148154	7.16E-05	1
Gba	0.148005	5.28E-05	1
Mpp7	0.147775	5.6E-05	1
Ndfip1	0.147749	0.018231	1
Acot13	0.147709	4.06E-05	1
Rpf2	0.147546	4.83E-05	1
Sec24a	0.147343	2.73E-05	0.882331
Siva1	0.147326	0.000652	1
Insig1	0.147054	0.000153	1
Rbx1	0.147016	0.002486	1
Trappc6a	0.146938	1.58E-05	0.508719
Erp29	0.146922	0.014119	1
Lgals8	0.146831	6.51E-05	1
Kif13b	0.146624	0.000106	1
A530040E1	0.146521	1.64E-06	0.053081
Enah	0.146396	2.61E-07	0.008415
Ufm1	0.146384	0.00051	1
Stoml2	0.14633	5.1E-08	0.001645
Ppp1cc	0.146198	6.88E-06	0.222012
Card11	0.146148	0.000644	1
Gm10134	0.146104	5.12E-10	1.65E-05
Dpp9	0.146088	4.79E-06	0.154747
Apobec3	0.145855	0.003841	1
Rtf2	0.145832	0.011267	1
Clec9a	0.145755	4.17E-08	0.001346
Rpl27a	0.14548	0.012762	1
Klf6	0.144674	0.013915	1
Rab22a	0.144437	0.000338	1
Ergic3	0.144395	6.67E-05	1
Gpatch4	0.144286	4.16E-07	0.013443
Cdca3	0.144146	0.000241	1
Gnl1	0.144012	3.01E-06	0.09725
Snrpd1	0.143996	0.001937	1
Slc15a3	0.143959	0.00092	1
Nudt14	0.143551	1.43E-05	0.460313
Mpst	0.1435	4.11E-05	1
Anxa5	0.143427	0.009713	1
Gm36043	0.143186	6.05E-05	1

Nubp2	0.142379	7.65E-05	1
Atp6v1d	0.142171	0.000361	1
Gm32089	0.142128	1.82E-09	5.86E-05
1500011B(	0.142105	1.02E-05	0.329258
Edem2	0.142018	7.95E-05	1
Ddx39b	0.14154	0.012223	1
Hnrnpa0	0.141428	5.82E-05	1
Mtif2	0.141221	2.84E-06	0.091597
Igf2r	0.141213	3.1E-08	0.001001
Acaa1a	0.141211	0.002009	1
Coro1c	0.141177	0.003406	1
Plk2	0.141104	2.32E-05	0.749523
Nudt9	0.141029	1.12E-05	0.361648
Nt5m	0.140955	3.56E-05	1
Mapk13	0.140942	1.31E-07	0.004235
Tmbim4	0.140539	0.106825	1
Ifi211	0.140397	0.000456	1
Plekhf2	0.140346	0.000179	1
Ccdc115	0.140344	0.000332	1
Abcd3	0.140313	9.33E-06	0.301276
Wdr36	0.140311	3.63E-05	1
Atf1	0.140271	0.000106	1
Mbd2	0.140119	2.43E-05	0.783586
Arl11	0.140045	3.25E-05	1
Pop7	0.139997	0.007008	1
C1d	0.139953	0.000249	1
Ap2m1	0.139889	0.007464	1
Rpl41	0.139882	0.008772	1
Itm2c	0.139731	0.000858	1
Nudc	0.139591	0.004688	1
Nelfe	0.139496	0.000862	1
Lamtor3	0.13943	0.00161	1
Srm	0.139373	2.52E-09	8.14E-05
Wsb2	0.139214	1.94E-07	0.006266
Tdg	0.13869	7.81E-06	0.252213
Clic1	0.138603	0.013545	1
Ggt5	0.138413	3.62E-05	1
Dusp23	0.138316	3.05E-09	9.84E-05
Ppp5c	0.137959	0.000714	1
Hibadh	0.137928	3.7E-05	1
Dtymk	0.137774	0.000127	1
Apba3	0.137688	1.06E-05	0.343747
Golt1b	0.13744	3.33E-06	0.107433
Ppp2r1a	0.137422	0.001951	1
Cyb561d2	0.13742	4.53E-06	0.14619
Gab1	0.137317	3.01E-07	0.009717
Rex1bd	0.137189	5.01E-05	1
Ilf3	0.137133	1.5E-06	0.048569

Dhx9	0.137092	0.002628	1
Naglu	0.137026	6.85E-08	0.002212
Mtfr1	0.13699	0.141105	1
Mrpl17	0.136792	1.79E-05	0.578244
Pcmtd1	0.136767	2.57E-07	0.00829
Nfkbia	0.136408	0.152635	1
Ssbp4	0.136116	0.000305	1
Slc28a2	0.136002	0.026592	1
Fkbp8	0.135968	0.005977	1
Lsm2	0.135835	4.03E-05	1
Trmt6	0.135712	0.000152	1
Ndufs4	0.135464	0.000268	1
March5	0.135394	0.000264	1
Tsn	0.135142	0.055425	1
Ptpn9	0.135124	0.000464	1
Aldh1l1	0.135008	0.001384	1
Arid5a	0.134947	2.58E-08	0.000832
mt-Nd6	0.134898	0.000669	1
Tmem104	0.134818	0.000219	1
Rhoh	0.134743	0.000161	1
Psmc1	0.134679	0.000265	1
Bola1	0.134565	0.005093	1
Fbxl2	0.134559	2.21E-05	0.713305
Tbrg1	0.134378	2.32E-07	0.007492
Aen	0.13435	0.000181	1
Calm3	0.134165	0.008318	1
Zcchc17	0.134079	0.000176	1
Mrpl30	0.134076	0.001909	1
Malsu1	0.133898	2.22E-05	0.715352
Gna15	0.133872	2.8E-06	0.090355
Htatsf1	0.133869	0.002393	1
Ndufb11	0.133814	0.001995	1
Med25	0.133766	0.004052	1
Ndufa4	0.133747	0.247269	1
Hebp1	0.133743	0.000123	1
Taf1d	0.133696	0.000188	1
Pirb	0.133692	0.253813	1
Mgat4a	0.133604	2.62E-06	0.084634
Hnrnpa3	0.133564	0.018499	1
Mrpl55	0.133455	4.18E-06	0.134817
Fam78b	0.133391	0.00012	1
Wrap73	0.13328	0.000497	1
Ptgs2os2	0.133207	1.44E-07	0.004663
Nt5c	0.133135	0.003985	1
Vopp1	0.132794	1.96E-05	0.631496
Selenos	0.132766	0.00026	1
Il11ra1	0.132693	1.17E-06	0.037815
Arf1	0.132651	0.018058	1

Copz2	0.13194	2.65E-08	0.000856
Gpn1	0.131899	3.73E-05	1
AU022252	0.131583	1.11E-06	0.035994
Pcbp1	0.131564	0.000435	1
Tmem33	0.131544	0.000425	1
Pusl1	0.131297	1.03E-06	0.033231
Lifr	0.130926	0.0308	1
Casp3	0.130856	0.004997	1
Map1lc3b	0.130777	0.007326	1
Tcf12	0.130621	0.002243	1
Kif13a	0.130435	5.74E-06	0.185338
Ech1	0.13041	0.000224	1
Atic	0.130393	0.000113	1
Atpaf2	0.130309	0.000306	1
Cnot10	0.13019	0.004313	1
Stk11	0.130155	0.001162	1
Alms1	0.129984	0.000577	1
Ube2l6	0.129927	0.042218	1
Plekhn1	0.1299	1.99E-08	0.000643
Fxyd5	0.12964	0.038213	1
Fam174a	0.129444	0.006076	1
Fcor	0.12944	8.97E-07	0.028967
Gm42047	0.129427	0.027853	1
Lamtor5	0.129363	0.013509	1
Lsr	0.129256	1.05E-05	0.338829
Eif1ax	0.129126	4.86E-06	0.156955
Dctn3	0.129113	0.002545	1
Iars	0.128871	6.46E-06	0.208436
St3gal3	0.128742	0.000278	1
Pdcd5	0.128622	0.000892	1
Mettl16	0.12837	0.002199	1
Snrnp25	0.128225	2.94E-05	0.949268
Ift27	0.128172	8.19E-06	0.264502
Arhgdia	0.128157	0.024045	1
Rars	0.128111	3.98E-05	1
Acadm	0.128046	0.000126	1
0610030E2	0.128043	0.001938	1
Sh3bgrl3	0.127984	0.001109	1
Gpr108	0.127954	0.000901	1
Fnbp1	0.127908	0.055513	1
Dtd1	0.127776	1.37E-05	0.443008
Tnp2	0.127611	3.7E-08	0.001195
Anapc1	0.127574	0.021075	1
Slc45a4	0.12744	3.68E-06	0.118877
Slc39a1	0.127327	1.85E-06	0.05981
Sri	0.127285	0.068792	1
Tomm40l	0.127205	0.001543	1
Pald1	0.127191	1.14E-06	0.036799

Sin3b	0.127123	0.003844	1
Nxn	0.127113	0.000506	1
Sdha	0.127097	0.008072	1
Magoh	0.126568	3.33E-05	1
Gm40645	0.126541	9.42E-05	1
Fcho1	0.126527	0.000346	1
Imp4	0.126415	0.001668	1
Cinp	0.12632	0.000174	1
Med9	0.126252	9.53E-07	0.030761
Ncapd2	0.126208	0.000807	1
Tbl1x	0.126195	0.002016	1
Orai1	0.126195	4.1E-05	1
Ntmt1	0.126168	0.000424	1
Atf4	0.126081	0.000171	1
Mrpl51	0.125945	0.000246	1
Aldh1b1	0.125931	3.3E-05	1
Mrps7	0.12593	3.41E-05	1
Ptges3	0.125898	0.002795	1
Nop58	0.125527	0.000493	1
Pi4ka	0.125473	0.000932	1
Bop1	0.125419	0.004456	1
Lyar	0.125284	0.000167	1
Slc41a2	0.12517	6.12E-07	0.019751
Smc4	0.12511	0.030206	1
Ube2n	0.125105	0.003641	1
Tuba4a	0.125063	0.012328	1
Fam136a	0.12505	4.9E-05	1
Ddb1	0.125046	0.006958	1
Nub1	0.124821	0.023224	1
Ssu72	0.124786	0.03863	1
Tmem273	0.12442	8.33E-08	0.00269
Bag1	0.124409	4.5E-05	1
Glul	0.124326	0.010759	1
Mrpl43	0.124297	0.02421	1
Dnajc11	0.124294	3.22E-05	1
Galk2	0.124149	0.030709	1
Gpr146	0.124004	2.21E-05	0.714234
Tigd2	0.123898	0.014164	1
Ghitm	0.123873	0.00622	1
Rnpepl1	0.123873	4.08E-06	0.131648
Caprin1	0.123831	0.019672	1
Mtch1	0.12381	0.000116	1
Slc35b1	0.123723	0.000426	1
Atp8a1	0.123277	0.040456	1
Rpl13a	0.123248	0.004844	1
Gtpbp8	0.123204	3.61E-06	0.116443
Mrpl22	0.123192	8.14E-06	0.262686
Zfp995	0.123174	0.002333	1

Sumo3	0.123045	1.8E-05	0.582019
Klhdc4	0.123035	4.24E-05	1
Gtpbp4	0.122984	1.35E-05	0.436482
Gbgt1	0.122574	6.53E-08	0.002107
Vma21	0.122539	0.000288	1
Ethe1	0.122486	0.000119	1
Alg9	0.122444	0.001405	1
Tcerg1	0.122435	0.014201	1
Cct5	0.122383	0.024911	1
Jpt2	0.122214	1.24E-05	0.400799
Metap1d	0.121982	0.078821	1
Ube2d3	0.121865	0.21706	1
Tsen15	0.121716	9.98E-06	0.322267
Psmd13	0.121687	0.004764	1
Rit1	0.12164	6.75E-05	1
Atad3a	0.121568	1.42E-07	0.004597
Rbms2	0.121528	0.002411	1
Tfdp1	0.121249	2.93E-05	0.94654
Wdr5	0.121186	2.22E-05	0.717632
Eef1akmt4	0.121185	1.32E-08	0.000427
Fos	0.121136	0.01007	1
Ppme1	0.121083	0.000138	1
Synj2	0.121075	8.01E-06	0.258493
Grhpr	0.121034	7.21E-07	0.023276
Tnfaip8l3	0.120934	1.62E-06	0.052399
Ap5s1	0.120889	2.14E-07	0.006905
Vps28	0.120757	0.036592	1
Srprb	0.120754	0.001593	1
Flywch1	0.120707	0.001237	1
Atp13a3	0.120473	0.000202	1
Selenoh	0.120386	0.000474	1
Nlrp3	0.120372	0.078245	1
Rabgef1	0.120315	7.76E-06	0.25069
Ktn1	0.120121	1.97E-05	0.635109
Mgst2	0.120095	0.003352	1
Txndc11	0.119913	3.91E-05	1
Mettl1	0.119558	1.89E-06	0.061166
Gm31718	0.119557	3.71E-06	0.119857
Ndufaf4	0.119025	9.08E-07	0.029321
Adam17	0.118972	0.005812	1
Dhx58	0.118816	0.000164	1
Nfil3	0.118658	4.1E-05	1
Snapc1	0.118641	0.000158	1
Fundc1	0.118595	0.003687	1
Azi2	0.118549	0.008655	1
Tmed9	0.118459	0.00135	1
Ddx31	0.118386	4.96E-06	0.160157
Rrp1	0.118356	0.006877	1

Shmt2	0.118327	5.28E-07	0.017054
Slpi	0.118305	0.004129	1
Atp23	0.118173	0.000124	1
Sar1a	0.118069	0.00034	1
Dnajc3	0.118057	0.0004	1
Tmem208	0.117859	0.010017	1
Ctu2	0.11782	6.03E-05	1
Abcf2	0.117803	0.000262	1
Nr4a2	0.117765	0.005809	1
Usp10	0.117684	0.000489	1
Chchd5	0.11768	6.83E-05	1
Pgs1	0.11767	0.000108	1
AB124611	0.117447	0.02756	1
Secisbp2l	0.11739	0.000298	1
Oat	0.1173	0.000475	1
Nectin2	0.117259	6.7E-08	0.002164
Scamp3	0.117211	0.000526	1
Atp6v1h	0.117211	0.002859	1
Pold2	0.117173	0.000462	1
Mpeg1	0.117013	0.065026	1
Ctnnb1	0.116847	0.060876	1
Hspbp1	0.116831	6.74E-05	1
Hmgn1	0.116747	0.000317	1
Rbm17	0.116743	1.94E-06	0.062722
Mcrip2	0.116592	3.09E-06	0.09979
Eif2s1	0.116551	0.000233	1
Cryl1	0.116504	0.006308	1
Dglucy	0.116425	0.001071	1
Tmem241	0.11642	0.000408	1
Fpr2	0.116329	4.27E-06	0.137881
Acox1	0.116144	0.009802	1
Plin2	0.116052	0.080855	1
Smim13	0.115992	3.26E-05	1
Rnf180	0.115864	2.25E-05	0.724819
Sdhaf4	0.115657	7.6E-06	0.24522
Gabarapl1	0.115551	5.28E-06	0.170608
H2afv	0.115363	0.073339	1
Nisch	0.115345	0.017816	1
Tpst1	0.115182	0.000346	1
Hdac2	0.115134	0.000128	1
C1qtnf6	0.115089	3.1E-08	0.001002
Med23	0.115032	2.08E-06	0.067017
Ephx1	0.114799	0.00059	1
Churc1	0.114674	0.007208	1
Tmem87b	0.114559	0.000348	1
Pafah1b3	0.114551	8.48E-05	1
Dym	0.114422	0.006005	1
Tipin	0.114335	0.016285	1

Iqgap2	0.114183	0.000113	1
Fosl2	0.114141	0.000276	1
Pole4	0.114121	5.64E-05	1
Rab9	0.114092	0.000388	1
Synpo	0.114029	4.59E-07	0.014826
Actr8	0.113816	0.025828	1
Atp6v0d1	0.113765	0.01493	1
Chmp1a	0.1137	0.000667	1
Snhg12	0.113642	8.84E-05	1
Smim3	0.113509	0.011173	1
Spr	0.113432	0.000124	1
Tmem245	0.113304	0.047139	1
Hnrnpdl	0.1133	0.178428	1
Kpna1	0.113281	0.06989	1
Enpp4	0.113201	8.19E-06	0.264437
Ms4a4b	0.11314	0.598785	1
Ddrgk1	0.113086	8.56E-05	1
Gcsh	0.112811	6.06E-05	1
Tmem63a	0.112756	2.29E-07	0.007377
Malt1	0.112514	0.929241	1
Prkar1a	0.112196	0.146538	1
Tomm22	0.112119	0.00436	1
Utp15	0.112085	6.35E-06	0.205162
Park7	0.11201	0.006829	1
Rabl6	0.111957	0.000181	1
Nup85	0.111775	7.94E-05	1
Tmem106b	0.111757	7.29E-06	0.23538
Slc37a2	0.111755	1.22E-06	0.039316
Ctla2b	0.111732	0.000222	1
Anapc4	0.11173	4.53E-05	1
Akr1b10	0.111647	0.000131	1
Nol12	0.111641	1.81E-06	0.058279
Sept8	0.111584	5.87E-05	1
Zswim7	0.111405	3.51E-05	1
Arfgap3	0.111307	1.86E-06	0.060207
Slc25a51	0.111287	0.023259	1
Eif3d	0.111273	0.009297	1
Hbp1	0.111252	0.004367	1
Heatr6	0.111227	5.09E-05	1
Idnk	0.111033	0.037763	1
Tmem9b	0.110981	0.021102	1
Knstrn	0.110723	0.003605	1
Blvrb	0.110616	0.024997	1
Psmd11	0.110509	0.061562	1
Pdcd2	0.110387	0.001197	1
Srp19	0.110347	0.00312	1
Gaa	0.110342	4.61E-05	1
Mknk2	0.110336	0.033464	1

Arrdc3	0.110329	0.00153	1
Mob2	0.110168	0.000704	1
Syvn1	0.110114	0.001008	1
Glod4	0.110108	0.039564	1
Hdgf	0.110056	0.005819	1
2610044O	0.109566	0.012738	1
Rpsa	0.109529	0.058615	1
Gramd3	0.10948	0.022191	1
Acadsb	0.109444	2.96E-05	0.955359
Rrp7a	0.109399	0.010053	1
Peli2	0.109195	2.35E-06	0.075986
Rpl11	0.109011	0.019592	1
Gclm	0.1089	3.75E-08	0.001211
Fads1	0.108788	4E-05	1
Ptbp1	0.10876	0.021426	1
Slc48a1	0.108697	0.001589	1
Exosc8	0.108631	0.000183	1
Elp1	0.108544	3.28E-06	0.106048
Psmd9	0.108419	4.43E-05	1
Ciapin1	0.108415	0.005672	1
Trim30c	0.108385	0.000458	1
Dusp10	0.108101	3.85E-05	1
Mvk	0.108079	0.003262	1
Eif2b1	0.108006	0.000891	1
Eef1e1	0.107802	0.00019	1
Rps6kc1	0.107791	4.11E-05	1
Vill	0.107765	0.131033	1
Rack1	0.107723	0.036008	1
Tor2a	0.107715	5.7E-05	1
Lztfl1	0.107523	0.00285	1
Coro7	0.10729	5.23E-05	1
Ubac1	0.107237	1.09E-05	0.352283
Ndufb4	0.107207	0.012044	1
Asna1	0.107195	1.83E-05	0.590132
Lamtor4	0.107174	0.112093	1
Tuba1c	0.107119	0.026991	1
Vps35	0.107077	0.041752	1
Eif3a	0.107068	0.020974	1
Ints2	0.10681	7.67E-06	0.247651
Sdhaf1	0.106779	0.000174	1
Eif2s2	0.106751	0.021114	1
Mtm1	0.106742	0.001336	1
Gm47283	0.106718	0.014879	1
Atm	0.106706	0.000223	1
Slc7a11	0.106704	2.63E-05	0.848521
Scamp1	0.106626	3.71E-06	0.119916
Pin1	0.106624	1.9E-05	0.612813
Ube2v1	0.106577	0.026173	1

Mrpl48	0.106527	0.004663	1
Tchh	0.106476	1.64E-07	0.005279
B230219D:	0.106455	0.001127	1
Cd69	0.106316	0.000553	1
Polr1d	0.106254	0.01308	1
Gm37240	0.106206	0.000241	1
Tgtp1	0.106155	6.95E-05	1
Cdkn3	0.106148	0.000307	1
Eif3l	0.106059	0.000503	1
Aldh9a1	0.10604	0.008174	1
Flot2	0.105894	0.000971	1
Vti1b	0.105669	0.003468	1
2310022A:	0.105457	0.130413	1
Ccng2	0.105308	0.004358	1
Ctnnd1	0.10527	0.015943	1
Kif23	0.104937	0.147498	1
Dcaf1	0.104937	1.61E-06	0.051839
Ostm1	0.104878	1.45E-05	0.469108
Dctn2	0.10486	0.067072	1
Leprotl1	0.104725	0.000367	1
G3bp1	0.104719	0.031235	1
Ezh2	0.104683	0.020709	1
Dad1	0.104562	0.035139	1
Bin1	0.104508	0.002161	1
Adora3	0.104501	5.75E-05	1
Pik3r1	0.104481	0.014878	1
Casp12	0.104362	2.5E-06	0.080606
Wdr48	0.104352	0.000513	1
Golgb1	0.104322	0.001005	1
Esr1	0.104307	0.003607	1
Zfp1	0.104293	0.000752	1
Abl2	0.104209	2.37E-06	0.076392
Icosl	0.10415	0.000108	1
Rps15a	0.104019	0.106088	1
3830406C:	0.103978	0.005281	1
Src	0.103977	1.5E-05	0.485629
Rnpep	0.103954	0.404501	1
Poglut1	0.103917	0.011556	1
Acd	0.103886	0.000598	1
Tor1b	0.103613	0.002229	1
Vezt	0.10358	0.000373	1
Hspa4l	0.10353	0.000126	1
Atrn	0.103451	0.019265	1
E330020D:	0.103424	4.97E-05	1
Mgrn1	0.103399	0.006444	1
Tnfsf13b	0.103338	0.000886	1
Sec24d	0.103226	1.42E-05	0.459264
Acpp	0.103179	1E-05	0.323229

Ssbp1	0.103118	0.001587	1
Usp5	0.102963	0.000215	1
Bscl2	0.102877	0.015415	1
Rxra	0.102868	0.000993	1
Rcan3	0.102822	0.000327	1
Ccdc122	0.10279	2.06E-05	0.66485
Chst14	0.102714	3.04E-06	0.09807
Lonp1	0.102617	0.001741	1
Mphosph6	0.102567	3.52E-05	1
Cyth2	0.10242	0.000235	1
Galt	0.10218	0.025053	1
Pon3	0.102143	1.5E-05	0.484915
Alkbh2	0.10201	0.000133	1
Rmi2	0.101933	2.14E-08	0.000691
Tmem199	0.101667	0.000651	1
Fez2	0.101596	8.1E-05	1
Wdr13	0.101576	0.000837	1
Osbpl3	0.101449	0.011213	1
Ubxn1	0.101446	0.029117	1
Pacsin1	0.101371	3.68E-06	0.118869
Rps4x	0.10133	0.083042	1
Imp3	0.101312	0.003845	1
Gnb4	0.101266	0.000747	1
Zpr1	0.101178	0.000437	1
Pi4k2a	0.101169	0.000375	1
Fkbp4	0.101167	0.009346	1
Rufy2	0.101114	3.31E-05	1
Chmp6	0.101081	0.001331	1
Hdhd2	0.100985	1.55E-05	0.500623
Mlf2	0.100727	0.030703	1
Otub1	0.100672	0.012132	1
Fam129b	0.10057	0.000196	1
Gpat3	0.100255	4.13E-06	0.133454
Mrps9	0.10016	0.000602	1
Cuta	0.100147	0.015102	1
Sclt1	0.100026	0.038896	1
Mrpl3	0.100022	0.003577	1
Dnmt1	0.099933	0.00449	1
Maff	0.099925	0.000117	1
Vapb	0.099775	0.000141	1
Slc10a3	0.099717	0.000121	1
Glrx2	0.099672	0.00646	1
Brip1	0.099653	0.000136	1
Smarcc1	0.099583	2.68E-05	0.865461
Prdx3	0.099567	0.000166	1
Mcph1	0.099401	0.000753	1
Gpkow	0.09931	0.021829	1
Ubald2	0.09926	0.049136	1

Ptges	0.099156	6.87E-06	0.22183
Cdk16	0.098958	2.4E-05	0.775223
Gm21860	0.09889	2.61E-05	0.842105
Tep1	0.098877	0.021715	1
Mecr	0.098798	0.011235	1
Pcyox1	0.098713	0.002649	1
Akt1	0.0985	0.0001	1
Ruvbl1	0.098392	2.14E-05	0.689693
Lcmt1	0.098288	0.00373	1
Slc38a6	0.098256	0.000163	1
Polr2m	0.098189	0.000792	1
Ifit1	0.098142	5.07E-05	1
Arhgap10	0.098118	1.4E-06	0.045311
Ccdc85b	0.098094	4.37E-05	1
Srp72	0.09799	0.000197	1
Timm10b	0.097917	0.027793	1
Ttc27	0.097864	0.002241	1
Rpia	0.097725	0.000105	1
Pdss1	0.097659	1.18E-05	0.380007
Ccdc32	0.097653	0.007186	1
Xpa	0.097652	7.19E-05	1
Uba2	0.097473	0.003474	1
Dap3	0.097377	8.77E-05	1
Cops9	0.097351	0.022655	1
Parp12	0.097332	0.003224	1
Dync1h1	0.097327	0.06966	1
Carmil1	0.097238	1.44E-05	0.465828
Tmem97	0.09719	0.002225	1
B4galt1	0.097135	0.020674	1
Gosr2	0.097095	0.045952	1
Ints11	0.097027	4.15E-05	1
Zbtb42	0.096999	0.009993	1
Cfap20	0.09696	0.000108	1
Tk1	0.096941	0.000574	1
March3	0.096786	0.000726	1
Usf2	0.096718	0.000735	1
Pip4p2	0.09661	0.00017	1
Tnfsf10	0.096592	8.85E-05	1
Rfc1	0.096571	0.240966	1
Il4ra	0.096469	0.005911	1
Apol9b	0.09644	7.17E-06	0.231527
Srr	0.096425	0.002059	1
Chrac1	0.096413	0.009917	1
Mapk9	0.096376	0.003168	1
Cox10	0.096326	0.03696	1
Enpp5	0.096284	0.000407	1
Zmat2	0.096261	0.001256	1
Tmem106c	0.096204	2.21E-05	0.712935

Anp32e	0.096184	0.025271	1
Micall2	0.095931	3.81E-05	1
Tiparp	0.095862	6.99E-05	1
Trmt11	0.095846	0.060474	1
Nup88	0.095779	0.019236	1
Iars2	0.095716	0.021068	1
Gtf2h4	0.095485	0.015394	1
Hras	0.095393	0.011302	1
Fnip1	0.095382	0.000587	1
Per2	0.095378	3.19E-06	0.102856
Morf4l2	0.095358	0.008969	1
Smyd2	0.095347	9.68E-06	0.312453
Minpp1	0.09533	0.002586	1
Nfkbie	0.095313	0.210749	1
Bhlhe40	0.095175	0.002604	1
Cops6	0.095069	0.004748	1
Mrps18b	0.095062	0.001968	1
Trmt2a	0.094944	0.000179	1
Adgrb1	0.094935	4.23E-07	0.013659
1110012L1	0.094846	0.043305	1
St6galnac4	0.094821	0.000955	1
Tyms	0.094717	2.27E-05	0.734483
Tsta3	0.094627	2.16E-06	0.069693
A930037H(	0.09461	0.023441	1
Wdr43	0.094606	0.000614	1
Optn	0.09457	1.41E-06	0.04555
Bad	0.094479	2.32E-05	0.748872
Txndc15	0.094347	0.015214	1
Nln	0.094273	0.003009	1
Msl3	0.094161	0.000971	1
Hyou1	0.094148	0.014799	1
Tbrg4	0.094119	0.006476	1
Nufip1	0.094024	3.98E-05	1
Mxd4	0.094016	0.000751	1
Tradd	0.093732	0.009386	1
Pinx1	0.093656	0.000246	1
Ruvbl2	0.093605	3.81E-05	1
Prkaa1	0.093503	0.022839	1
Rmc1	0.093483	0.002472	1
Mavs	0.093452	0.000601	1
Neu1	0.093416	0.001375	1
Dmxl1	0.093398	0.000127	1
Mrto4	0.09327	0.002026	1
Prkar2a	0.093216	7.74E-05	1
2310039H(	0.09321	0.000301	1
Tomm5	0.093035	0.01275	1
Klhl2	0.092892	0.129135	1
Lsm4	0.092854	0.056168	1

Stip1	0.092852	0.005633	1
Ankrd40	0.092852	0.000242	1
Psat1	0.092786	0.000221	1
Mepce	0.092717	0.042738	1
Kpna3	0.092704	0.000117	1
Rps23	0.092694	0.139873	1
Dlat	0.092647	0.000111	1
Ppp1r12c	0.092523	0.018136	1
Rps19bp1	0.092457	0.000288	1
Als2cl	0.092435	2.4E-06	0.077471
Cggbp1	0.092393	0.060231	1
Ckap4	0.092232	0.012833	1
Ddx54	0.092223	0.006783	1
Ei24	0.092159	0.000289	1
Slc35f6	0.092098	0.000255	1
Trmt61a	0.092054	0.000227	1
Ell2	0.092032	0.005928	1
Uba5	0.091979	0.000191	1
Tpcn2	0.091968	4E-06	0.129255
Dld	0.091858	0.006352	1
Zfp64	0.091854	1.31E-05	0.423766
Clip2	0.09176	1.64E-05	0.528644
Ado	0.091731	0.000502	1
Fam214a	0.091709	0.163408	1
Cops5	0.091638	7.95E-05	1
Dnlz	0.091566	0.022009	1
Mat2b	0.091539	0.009515	1
Cd52	0.091504	0.227807	1
Trappc3	0.091458	0.001216	1
Pym1	0.091431	0.000302	1
Mta1	0.091406	0.000241	1
Psmc3	0.091363	0.061775	1
Raly	0.091177	0.005681	1
Cox14	0.090975	0.03965	1
Adamtsl4	0.090909	2.45E-06	0.079208
Prps1	0.090895	7.67E-05	1
Maz	0.090698	0.003212	1
Rbck1	0.090679	0.00277	1
Afg1l	0.090632	0.000105	1
Zfp281	0.090498	0.000359	1
Stard9	0.090426	0.001582	1
Cdc16	0.090278	0.000111	1
Gsto1	0.090233	0.005878	1
Rest	0.090227	0.000386	1
Polr2f	0.090125	0.0003	1
Rhou	0.089867	7.02E-05	1
Mdh1	0.089855	0.21577	1
Sik3	0.089855	0.003772	1

Atf2	0.089837	0.001483	1
Cpsf1	0.089837	5.05E-05	1
Trap1	0.089807	8.44E-06	0.272632
Gpr180	0.089766	0.000313	1
Pld2	0.089687	6.08E-05	1
Rpain	0.089684	0.000351	1
Pkib	0.089593	1.01E-06	0.032661
Ncapg2	0.089567	0.003969	1
Tmem183a	0.089437	0.000694	1
Fam120a	0.08943	0.047692	1
Smurf1	0.089375	1.42E-05	0.459481
Hells	0.089332	0.00211	1
Iah1	0.089312	0.000625	1
Itm2b	0.089246	0.034165	1
Pigk	0.089169	1.03E-05	0.332288
Hspb11	0.089096	9.98E-05	1
Ncbp2	0.089046	0.001529	1
Gtf2a2	0.089044	0.039189	1
Uqcc3	0.08904	0.002273	1
Nmi	0.088869	0.008258	1
Ftsj1	0.088861	9.04E-05	1
Pola1	0.088843	0.000297	1
Fggy	0.088813	1.97E-06	0.063647
Diaph3	0.088744	0.027244	1
Ints1	0.088634	0.001622	1
Rpl18a	0.088626	0.031021	1
E130309D(	0.088572	3.36E-05	1
E2f4	0.088467	0.002527	1
Mpg	0.088391	0.014487	1
Slx4ip	0.088302	0.043406	1
Aurkaip1	0.088284	0.119949	1
Gm37233	0.08827	2.33E-06	0.07521
Trnt1	0.088142	0.034492	1
Asl	0.088103	0.000217	1
Hmgb2	0.088068	0.649722	1
Crbn	0.088065	0.005309	1
Hikeshi	0.088023	0.004774	1
Manbal	0.088014	6.39E-06	0.20614
Eif4g1	0.087923	0.013536	1
Slc25a46	0.087827	3.79E-05	1
Fbxo34	0.087739	0.012974	1
Ppt2	0.087642	0.015418	1
Gm4117	0.087591	0.04551	1
Rab3ip	0.087529	1.64E-05	0.528607
Pttg1ip	0.087373	0.000269	1
Ptges2	0.087343	0.000433	1
Arf4	0.087092	0.028059	1
Znhit2	0.087024	9.13E-05	1

Rpa2	0.086958	0.14361	1
Strap	0.086771	0.030736	1
Zfand2a	0.086675	0.001339	1
Mea1	0.086458	0.040654	1
Rbm28	0.086273	0.013728	1
Rusc1	0.08623	0.403541	1
Agap3	0.086209	5.57E-05	1
Itpk1	0.086181	0.002071	1
Ifi35	0.086172	0.01068	1
Spata5	0.086046	0.053688	1
Ubl7	0.085988	0.008189	1
Mrps5	0.085977	1.37E-05	0.441232
Nfu1	0.085873	0.000333	1
Tra2a	0.085869	0.031052	1
Osbpl1a	0.085852	3.78E-06	0.122092
1110051M	0.085806	5.15E-05	1
Polr3d	0.085735	0.000314	1
Mag	0.085707	3.98E-06	0.128467
Rfc5	0.085598	3.64E-05	1
Polr2e	0.085479	0.001017	1
Snx8	0.085424	2.45E-06	0.079224
Rrs1	0.085324	1.5E-05	0.485167
Gla	0.085194	0.000166	1
Tmem101	0.085134	0.009997	1
Lamc1	0.085052	0.00026	1
Hdgfl2	0.084999	0.000251	1
Acvrl1	0.084984	0.002002	1
Ccdc71l	0.084837	0.013854	1
Tagap	0.084741	0.030615	1
Arsg	0.084684	2.26E-05	0.729837
Rab11fip5	0.084654	0.000205	1
Slc35a3	0.084646	0.003264	1
Sgta	0.084609	0.000248	1
Cmtm6	0.084607	0.025704	1
Rcc1	0.084512	0.002788	1
Rcc1l	0.084506	2.41E-05	0.778319
Prps1l3	0.084426	1.21E-05	0.391398
Thap7	0.08439	0.02531	1
Hpf1	0.084352	0.002199	1
Zfp622	0.084337	0.010187	1
Gys1	0.084278	0.00037	1
Sh3glb2	0.084057	0.000393	1
Kat14	0.084023	0.000509	1
Pofut2	0.083962	0.006472	1
H2-M3	0.083942	0.160273	1
1110032A(	0.083937	0.015255	1
Abhd4	0.083926	0.004241	1
Kif1c	0.083885	5.21E-06	0.168259

Maf	0.083879	8.39E-06	0.270814
Mrpl28	0.08387	0.000123	1
Ltbr	0.083834	0.007403	1
Clec4d	0.083805	8.44E-05	1
Chd4	0.083767	0.355206	1
Cox17	0.083723	0.067531	1
Gm49359	0.083705	0.001446	1
Tmem126a	0.083604	0.0006	1
Fxn	0.083506	0.000955	1
Carm1	0.083458	0.010275	1
Hint3	0.083411	6.17E-05	1
Slc7a5	0.083323	5.5E-06	0.177611
Ciao2a	0.083321	0.093986	1
Meaf6	0.083201	0.0005	1
Psma7	0.083194	0.116533	1
Mrrf	0.083156	0.000165	1
Vps18	0.083141	8.71E-05	1
Mbd3	0.08306	0.000237	1
Atp6v1b2	0.082977	0.064374	1
Egln2	0.082878	0.00019	1
Txnl1	0.082858	0.006255	1
Eif1ad	0.08278	0.005683	1
Mfn2	0.082774	0.000277	1
Mcm7	0.082731	0.00468	1
Cep164	0.082685	0.00326	1
Ptrh2	0.082664	0.000569	1
Cluh	0.082662	0.009169	1
Rab6a	0.082657	0.028874	1
Gorasp2	0.082644	0.020017	1
Polr2d	0.082637	0.000444	1
Htatip2	0.08258	0.001003	1
Elof1	0.082565	7.67E-05	1
Cdc34	0.082549	0.000101	1
Rab28	0.08249	4.98E-05	1
Pcm1	0.082461	0.00193	1
Bckdhb	0.082412	0.001736	1
Kdsr	0.082407	0.003559	1
Slc39a3	0.082319	5.65E-05	1
Alg5	0.08229	0.00017	1
Ddx21	0.082232	0.03804	1
Cenpm	0.082227	1.03E-05	0.331641
Eif4e3	0.082199	0.018143	1
Tex14	0.082141	0.000346	1
Mfhas1	0.082084	0.000659	1
Bcl2a1d	0.082083	0.028584	1
Gtf2f1	0.082037	0.002248	1
Npc1	0.081953	0.001809	1
Serbp1	0.08192	0.088288	1

Bcl2a1a	0.081881	0.000126	1
Elp3	0.081818	2.06E-05	0.665684
Mcm4	0.08177	0.077972	1
Rragc	0.081763	0.002914	1
Urb1	0.081719	0.000105	1
Zdhhc1	0.081567	0.003157	1
Sqle	0.081454	0.052074	1
Actr1b	0.081437	0.000396	1
Aco1	0.081417	0.001515	1
Ppm1m	0.081363	0.00295	1
Ubxn6	0.08131	0.000129	1
Pdcd1lg2	0.081281	0.00198	1
Gnai3	0.081268	0.072729	1
Trappc5	0.081184	0.001487	1
Wdr12	0.081074	0.000501	1
Snhg4	0.081044	0.000489	1
Trir	0.081012	0.114147	1
Dnajc25	0.080984	7.23E-05	1
Sav1	0.080945	6.03E-05	1
Exosc5	0.080899	1.87E-05	0.604709
Tma7	0.080732	0.45422	1
Ints4	0.08065	0.037284	1
Ocstamp	0.080501	0.046481	1
Arl8b	0.080452	0.004236	1
Plk3	0.080449	0.001225	1
Ctns	0.080325	0.000268	1
Psme4	0.080321	0.026768	1
Slfn2	0.080098	0.232206	1
Mrpl37	0.080042	0.000418	1
Blvra	0.080006	0.009927	1
Lancl1	0.079959	0.063986	1
Thap4	0.079933	1.47E-05	0.475206
Romo1	0.079923	0.008738	1
Ptpn18	0.079756	0.127455	1
Rab24	0.079756	0.000438	1
Slc9a3r1	0.079678	0.010219	1
B4galt6	0.079609	0.000505	1
Pink1	0.079402	0.016035	1
Dtx3	0.079307	2.45E-06	0.079051
Nedd8	0.079291	0.21107	1
Zcchc2	0.079238	0.038978	1
Wipi1	0.079125	0.000125	1
Tubgcp3	0.079124	0.056241	1
Dag1	0.07904	7.47E-05	1
Uggt1	0.079039	0.018781	1
Gatd3a	0.078989	0.000725	1
Focad	0.078959	0.001274	1
St14	0.07885	5.75E-05	1

Mvp	0.078769	0.016289	1
Mocs2	0.078737	5.68E-05	1
Dis3l2	0.078713	0.000529	1
Acyp2	0.078702	0.001286	1
Wdr4	0.078695	0.000352	1
Mcub	0.078636	0.013475	1
Atxn10	0.078615	0.019878	1
Gmds	0.078538	0.001175	1
Tmem65	0.078414	9E-05	1
Clec4e	0.078388	0.655322	1
Gm13203	0.078356	6.7E-06	0.21625
Hsdl2	0.07818	0.005139	1
Bcs1l	0.078159	0.005768	1
Cltb	0.078089	4.55E-05	1
Gnptab	0.078077	0.008327	1
Ehmt2	0.077978	0.008903	1
Noc4l	0.077954	0.00067	1
Timm17a	0.07788	0.003622	1
Tmx1	0.077871	0.059214	1
Dexi	0.077833	0.000385	1
Ptpn23	0.077822	2.97E-05	0.960224
Ecsit	0.077817	0.001714	1
Dpy30	0.077745	0.006221	1
Ccz1	0.077652	0.020908	1
Eif4h	0.077646	0.045473	1
Hspbap1	0.07761	0.001514	1
Cldnd1	0.077569	5.07E-05	1
Fgfr1op	0.077556	0.015147	1
Ssrp1	0.077499	0.010897	1
Smarca4	0.077488	0.1423	1
Dera	0.077437	0.01297	1
Aacs	0.077434	0.000275	1
Uxt	0.077409	0.001004	1
Ipo5	0.077306	3.5E-05	1
Msantd2	0.077178	0.004119	1
Trp53inp2	0.077165	0.00028	1
BC147527	0.077147	0.397873	1
Slc25a13	0.077122	7.99E-06	0.257975
Rnf213	0.077095	0.274707	1
2410022M	0.077028	0.004954	1
Osbpl7	0.077006	0.007035	1
Psma1	0.077005	0.019305	1
Rps11	0.076962	0.100689	1
Fbh1	0.076949	0.001669	1
Parn	0.076929	0.001151	1
Dcun1d5	0.076859	0.007011	1
Ltv1	0.076799	0.000431	1
Rad17	0.076794	0.05418	1

Commd9	0.076791	0.000248	1
Gm33370	0.076637	0.000843	1
Chpt1	0.076562	0.000637	1
Srgn	0.076433	0.78997	1
Yeats4	0.076315	0.001407	1
Fn1	0.076261	2.41E-05	0.776494
Slc25a12	0.07626	0.002487	1
Glt8d1	0.076242	0.006755	1
Lrrc42	0.076231	0.001756	1
Phf5a	0.076198	0.004107	1
Calm2	0.075704	0.201605	1
Kcnk13	0.075628	0.000846	1
Gspt1	0.075625	0.006797	1
Wdr44	0.075619	0.034197	1
Pfdn4	0.075613	0.012781	1
Tbl3	0.075603	0.006763	1
Tent5c	0.075573	3.87E-05	1
Trim3	0.075512	2.83E-05	0.91319
Sap18	0.075475	0.213628	1
Rnf187	0.075466	0.002056	1
Nudcd1	0.075322	4.56E-06	0.147349
6330562C2	0.075301	6.85E-06	0.221228
Ifi208	0.075289	0.000131	1
Pip4p1	0.075286	0.00252	1
Ten1	0.075268	0.022625	1
Rab12	0.075252	3.59E-05	1
Mrps18c	0.07521	0.010881	1
Zc3h10	0.075197	0.033737	1
Eef1aknmt	0.075162	0.002797	1
Spryd3	0.075131	5.13E-05	1
Zfp787	0.075117	0.001441	1
Asb11	0.075117	1.24E-06	0.040175
Shq1	0.075117	0.001029	1
Setd1a	0.075114	0.020643	1
Faf2	0.075027	0.001627	1
Eny2	0.075003	0.001245	1
Aamdc	0.074961	0.001057	1
Mrpl27	0.074904	2.13E-05	0.686311
Dnajc9	0.074901	0.077247	1
Lman1	0.074851	7.5E-05	1
Nos1ap	0.074788	2.42E-05	0.781332
Dcaf4	0.074777	7.44E-05	1
Ptpn2	0.074749	0.000609	1
Lrrc4	0.074734	2.81E-05	0.907166
Agpat2	0.074651	0.000462	1
Ctdspl	0.074647	0.000621	1
Brap	0.074636	0.032581	1
Ilf2	0.074629	0.011705	1

Ap4b1	0.074623	0.013938	1
Drg1	0.074605	0.033916	1
Gadd45gip	0.074584	9.43E-05	1
Timm8a1	0.074571	9.93E-05	1
Rnf145	0.074481	0.004921	1
Gca	0.074391	4.57E-05	1
Rhbdd2	0.07439	0.005472	1
Psmb5	0.074323	0.072134	1
Ccdc58	0.074268	0.001117	1
Washc1	0.074224	0.003724	1
5430416N(	0.07415	0.002106	1
Ppp1r7	0.074113	0.055763	1
Blcap	0.074098	0.002843	1
Cars	0.074072	0.000557	1
Mrpl50	0.074005	0.000112	1
Rfxank	0.073869	0.004731	1
Galnt11	0.073817	0.004323	1
Fdxr	0.073804	0.025478	1
Twsg1	0.073722	3.41E-05	1
Nipsnap2	0.073707	0.002215	1
Lars	0.073684	0.000654	1
Cep120	0.073637	0.000601	1
Alg8	0.073446	0.007916	1
4930405A:	0.073369	0.004946	1
Tg	0.07335	0.003959	1
Emc6	0.073335	0.015218	1
Ufc1	0.073212	0.023625	1
Mmp8	0.073147	0.093328	1
Tacc3	0.073142	0.036678	1
Arl4a	0.073121	0.000113	1
Mterf4	0.073116	0.000504	1
Kifap3	0.073097	3.59E-05	1
Tmem67	0.073018	0.000281	1
Rnf169	0.072987	0.101931	1
Gm17494	0.072936	0.00623	1
Pld1	0.072853	0.063711	1
Sf3b4	0.072814	0.078989	1
Mrpl47	0.072768	0.000495	1
Fam104a	0.072738	0.009541	1
Foxk2	0.072587	0.004552	1
Zfp691	0.07245	0.01664	1
Pard6a	0.072395	0.000527	1
Adsl	0.072393	0.000122	1
Psmd6	0.072345	0.003987	1
Slc30a7	0.072343	0.000453	1
Poldip2	0.07229	3.81E-05	1
Mov10	0.072202	0.000368	1
Aga	0.071971	0.016425	1

Agfg2	0.071971	0.000286	1
Sdf4	0.071926	0.178172	1
Thoc3	0.071915	0.020962	1
Rcor1	0.071874	0.002482	1
Get4	0.071868	3.02E-05	0.974054
Pik3r2	0.071831	0.002145	1
Zmym6	0.07171	0.022015	1
Zfp810	0.07165	0.000768	1
Gmps	0.071601	0.014171	1
Ankrd37	0.071543	0.002524	1
Clcn5	0.071539	0.00418	1
Tmem134	0.071527	0.139538	1
Dpysl2	0.071478	0.002457	1
Asah2	0.071379	0.001244	1
Psmc5	0.071267	0.035795	1
Irs2	0.071261	0.116271	1
Pmm1	0.071235	0.000146	1
Nup205	0.071098	0.001572	1
Txndc9	0.071045	0.002655	1
Riok3	0.071003	0.095467	1
Plekhm2	0.070974	0.000234	1
Slc52a2	0.070968	0.000122	1
Rdm1	0.07088	0.221267	1
Mul1	0.070869	0.001087	1
Tbc1d14	0.07085	0.011701	1
Zdhhc21	0.070804	4.5E-05	1
Tnfaip8l2	0.070718	0.125543	1
Arl2	0.070685	0.000992	1
Riok1	0.070584	0.081249	1
Rnaseh2b	0.070535	0.000418	1
Nubpl	0.070525	0.00041	1
Nol7	0.070469	0.000536	1
Ext2	0.070369	0.010781	1
Atad5	0.070357	0.008472	1
Ccnd1	0.070303	5.1E-06	0.164519
Ndufs2	0.070291	0.04083	1
Stk19	0.070285	0.001882	1
Stx12	0.070234	0.000564	1
Epg5	0.070219	0.002218	1
Cp	0.070214	0.011245	1
Gmppb	0.070178	0.042448	1
Poc1a	0.07017	0.028306	1
Chka	0.070124	5.39E-05	1
Exosc2	0.07009	0.000638	1
Arl10	0.070054	0.281914	1
Rbbp8	0.069954	0.137851	1
Grwd1	0.069948	0.00012	1
Clmp	0.069914	0.000289	1

Alkbh1	0.069902	0.037661	1
Cebpe	0.069899	0.008918	1
Hsp90aa1	0.069896	0.084608	1
Relt	0.069866	0.116638	1
Fam3a	0.069866	0.008057	1
Washc5	0.069829	0.002331	1
Lsm12	0.069743	0.012468	1
Ndst2	0.069726	6.92E-05	1
Nfkbil1	0.06964	0.025927	1
Nomo1	0.069566	0.000712	1
Rnf220	0.069562	0.014591	1
Pold4	0.069556	0.016944	1
Pias4	0.069513	0.002435	1
Tmed1	0.069414	0.00142	1
Slc6a6	0.069401	0.003905	1
Nmt2	0.06931	0.000226	1
Mrm1	0.069181	0.002514	1
Stard7	0.069118	0.006208	1
Sf3a2	0.069042	0.031078	1
Pml	0.068965	0.00698	1
Znfx1	0.068916	0.000965	1
Slc30a9	0.068736	6.78E-06	0.218903
Tecpr2	0.068661	0.002198	1
Trp53i13	0.068659	0.012053	1
Pop4	0.068585	6.51E-05	1
Bbs9	0.068528	0.090382	1
Hs2st1	0.068527	0.005756	1
Ino80e	0.06851	0.006097	1
Cttnbp2nl	0.068447	3.77E-06	0.12159
Hyal2	0.068436	0.000662	1
Mmadhc	0.068429	0.000485	1
Armc10	0.068425	0.044237	1
Smc5	0.068414	0.007659	1
Rab18	0.068356	0.002299	1
Them6	0.06816	0.010683	1
Tspan32	0.068138	0.00152	1
Cad	0.068103	0.0002	1
Bcar3	0.068058	0.000132	1
Ch25h	0.068019	2.13E-05	0.686504
Psme3	0.067924	0.001275	1
Plppr3	0.06791	0.000144	1
Gm5577	0.067867	0.001103	1
Abtb2	0.067823	0.003888	1
Gfm1	0.067782	5.04E-06	0.162727
Ogfod3	0.067763	5.55E-05	1
Srrd	0.067762	0.006624	1
Mrps11	0.06776	0.011716	1
Pdk3	0.067749	0.002743	1

Tpd52l2	0.067717	0.206004	1
Unc45a	0.067683	0.001429	1
Dip2a	0.067677	0.009408	1
Uba1	0.067622	0.035696	1
Cs	0.06757	0.176642	1
Hnrnpc	0.067485	0.168322	1
Eif3e	0.067347	0.002527	1
Ctbp1	0.067288	0.000706	1
Pbdc1	0.067204	0.01355	1
Mvb12a	0.067163	0.001295	1
Nudt6	0.067098	0.001803	1
Nudt8	0.067065	0.000909	1
Dph1	0.066948	0.032373	1
Zc3h12c	0.066918	0.301146	1
Znhit6	0.066864	6.46E-05	1
Batf	0.06676	0.134948	1
Fam117a	0.066739	0.01134	1
Cd81	0.066684	0.001691	1
Bcl2l2	0.06661	0.000287	1
Ola1	0.066583	0.006205	1
Txnl4a	0.066579	0.008411	1
Ebna1bp2	0.066559	0.065928	1
Fdx1	0.066551	0.001764	1
Apool	0.066523	6.28E-06	0.202821
Castor2	0.066466	2.27E-05	0.732762
Prpf38a	0.066444	0.00287	1
Rala	0.066414	0.003334	1
Ubxn4	0.066408	0.049562	1
Wdr73	0.066363	0.0014	1
Trappc2l	0.066282	0.008951	1
Racgap1	0.066226	0.309931	1
Emc4	0.066191	0.007572	1
Chmp7	0.066104	0.005386	1
2310040G	0.066076	0.001077	1
Dis3	0.066009	9.66E-06	0.31172
Rp9	0.065985	0.083615	1
Rac1	0.065791	0.289108	1
Keap1	0.065767	0.052422	1
Stk39	0.065748	0.000288	1
Rsl24d1	0.065663	0.009727	1
Rps14	0.065611	0.194149	1
Wdr1	0.065592	0.242374	1
Brox	0.065576	0.047503	1
Fbxo33	0.065567	0.000566	1
Iqsec1	0.065546	1.04E-05	0.336681
Dpm3	0.065491	0.109417	1
Sae1	0.065445	0.00948	1
Palm	0.065424	0.003009	1

Tulp4	0.065229	0.001126	1
Plpp5	0.065143	0.000235	1
Mad2l1	0.065137	0.000533	1
Mapkbp1	0.065086	2.71E-05	0.87573
Cby1	0.064939	0.001007	1
Ptger2	0.064907	3.17E-06	0.102409
Fen1	0.064851	0.01202	1
Zswim8	0.064821	0.003636	1
Rpl6	0.064819	0.072401	1
Ik	0.06477	0.043502	1
Kdm4b	0.064694	0.000269	1
Dicer1	0.064615	0.002383	1
Mob3c	0.0646	0.00031	1
Gart	0.064591	3.53E-05	1
Ipo9	0.064583	0.000276	1
Yars	0.064542	0.061682	1
Mcemp1	0.064472	0.006038	1
Dnajc4	0.064352	0.001567	1
Wdr77	0.064309	0.003542	1
Rbm45	0.064264	0.199239	1
Jazf1	0.064232	0.003144	1
AI504432	0.064143	0.006724	1
Ctps	0.064019	8.34E-05	1
Tmco3	0.064018	0.001338	1
Wipi2	0.063949	0.003476	1
Zfp771	0.06393	0.004714	1
Idh1	0.063928	0.035052	1
Tmem248	0.063857	0.003769	1
Klhdc3	0.063849	0.009363	1
Cherp	0.063769	0.016869	1
Ubfd1	0.063673	0.001075	1
Paqr7	0.063661	0.001289	1
Ttpal	0.063632	0.00425	1
Ergic2	0.063617	0.002645	1
Sepsecs	0.063545	5.71E-05	1
Rpl22	0.063539	0.035479	1
E230016K2	0.063531	0.014369	1
Ppih	0.063512	0.001195	1
Fech	0.063507	0.002234	1
Cfap36	0.063504	9.07E-05	1
Kdm2b	0.063399	0.040506	1
Abca1	0.063303	0.001367	1
Bax	0.063245	0.014254	1
Trit1	0.063203	0.001233	1
Gpr160	0.063187	0.013181	1
Dcp2	0.063179	0.003777	1
Mcoln1	0.063113	0.010393	1
Galnt2	0.063088	0.001568	1

Sppl2b	0.06306	0.000374	1
Ttll5	0.062999	0.004688	1
Aptx	0.062903	0.024855	1
Hdac1	0.062872	0.027022	1
Tmem37	0.06287	0.014195	1
Erg28	0.062826	0.006352	1
Man2a2	0.062823	0.010839	1
Mapre2	0.062813	0.173486	1
Nudt2	0.062791	0.000113	1
Ddx18	0.062785	0.00072	1
Kat2a	0.062763	0.003737	1
Dusp19	0.062714	5.48E-05	1
Msh3	0.062624	0.001117	1
Wdr11	0.062519	0.002127	1
Tmem167	0.062511	0.022511	1
Odf2	0.06241	0.000252	1
Ift57	0.062403	0.00538	1
Galm	0.062367	0.000393	1
Tmem260	0.062281	0.000152	1
Gtpbp2	0.06227	0.007031	1
Lemd2	0.06223	0.001181	1
Atpaf1	0.062221	0.04025	1
Tgs1	0.062217	0.01067	1
Ints12	0.062176	0.010927	1
Tnfsf9	0.062063	0.00018	1
Myh10	0.062018	0.000131	1
Mapk1ip1l	0.061921	0.037597	1
Bzw2	0.061919	0.004578	1
Srp68	0.061848	0.001844	1
Nit1	0.061759	0.022276	1
Rab35	0.061708	0.040648	1
Tmtc4	0.061638	5.58E-05	1
Kdelr1	0.061635	0.018022	1
Zfyve19	0.061624	0.001339	1
Nmnat3	0.061593	0.005721	1
Unc93b1	0.061571	0.118827	1
Incenp	0.061434	0.011632	1
Rpp21	0.061411	0.111431	1
Sugt1	0.061392	0.024148	1
Rnf121	0.061347	0.008689	1
Tubg1	0.061346	0.002416	1
Ide	0.061329	0.002398	1
Trpm4	0.061326	0.003022	1
Spryd7	0.061311	0.001035	1
Gas8	0.061295	0.000403	1
Coa6	0.061258	0.020159	1
Fnip2	0.061251	2.75E-05	0.888583
Arfgap1	0.061212	0.019948	1

Dcxr	0.061144	0.045571	1
Agpat5	0.061112	3.63E-05	1
Mfsd7a	0.061096	0.013788	1
Polr1a	0.061047	4.63E-05	1
Natd1	0.061028	0.000677	1
Cd3eap	0.060947	0.003295	1
Nom1	0.060936	0.000561	1
Mcrs1	0.060869	0.020773	1
Dph6	0.060838	0.003542	1
Zcchc9	0.06079	0.002148	1
Uri1	0.06077	0.000171	1
Fam102b	0.060762	0.004031	1
Suv39h1	0.060713	0.000771	1
Oas1g	0.060637	0.019437	1
Zbtb5	0.060609	0.000291	1
Atp6v1c1	0.060552	0.000158	1
As3mt	0.060531	0.002067	1
Megf8	0.060391	0.001397	1
Arpc1a	0.06039	0.050627	1
Ctdp1	0.060284	0.000264	1
Zfp951	0.060215	0.023339	1
Mzt1	0.0602	0.002896	1
Jagn1	0.060177	0.008959	1
Seh1l	0.060132	0.000132	1
Nsmce1	0.060094	0.007248	1
Gtf2i	0.06	0.031508	1
Rce1	0.059977	2.23E-05	0.718943
Emp1	0.059959	0.006078	1
Smim14	0.059922	0.048764	1
Dusp3	0.0599	0.002931	1
Fer	0.05989	0.000239	1
5730480H(	0.059885	0.001383	1
Triobp	0.05982	0.005413	1
Leo1	0.059799	0.027184	1
Adi1	0.059715	2.63E-05	0.849483
Gm14636	0.059696	0.000101	1
Ccl25	0.059673	0.057109	1
Epn1	0.059593	0.116055	1
Sdr39u1	0.059461	0.001894	1
Dnajc2	0.059456	0.002813	1
Pex11a	0.059434	0.000304	1
Rps19	0.059433	0.097374	1
Qrsl1	0.059311	0.048346	1
Entr1	0.059306	0.244066	1
Chid1	0.059187	1.76E-05	0.566885
Etf1	0.059184	0.350421	1
Calu	0.05917	0.00142	1
Elovl1	0.059041	0.048379	1

Fam122a	0.059007	0.00097	1
Thop1	0.059007	0.009342	1
Rrp9	0.058924	0.000175	1
Tmem129	0.058874	0.000155	1
Smim26	0.058818	0.000323	1
Hectd3	0.058761	0.031456	1
Pex16	0.058713	0.001845	1
Ada	0.058702	0.00205	1
Mast2	0.058647	0.001498	1
Rabac1	0.058618	0.060556	1
R3hcc1	0.058553	0.000564	1
Orai3	0.058529	0.019659	1
Kcnk6	0.058511	0.00652	1
Tmem126b	0.058435	0.003216	1
Ccnc	0.058324	0.088418	1
Mipep	0.058298	0.009791	1
Akip1	0.058219	0.003652	1
Farp2	0.058182	0.000175	1
Cdk10	0.058178	0.000844	1
Smap1	0.058166	0.031988	1
Slc4a1ap	0.058056	0.015835	1
Znhit1	0.057971	0.040391	1
Fam110a	0.057944	3.45E-06	0.111391
Cpt2	0.057922	0.070246	1
Mllt3	0.057911	0.015156	1
St6galnac6	0.05791	0.001597	1
Acot7	0.057829	0.000475	1
Dazap1	0.057819	0.01651	1
Lrrc14	0.057784	9.24E-05	1
Parp6	0.057759	7.76E-05	1
Syap1	0.057703	0.230447	1
Rpl7l1	0.057602	0.079261	1
Twf1	0.057595	0.002693	1
Acad8	0.057581	0.045825	1
Trp53rka	0.057553	0.020763	1
Dkc1	0.057486	0.000596	1
Clptm1	0.057467	0.026477	1
Blzf1	0.057337	0.002747	1
Mrpl9	0.05731	0.009921	1
Selenbp1	0.057298	0.023857	1
Pole3	0.057282	0.002287	1
Alg13	0.05725	0.00767	1
Mrpl45	0.057194	0.000665	1
Eef2	0.057191	0.057739	1
Dgcr8	0.057135	0.000678	1
Tlr1	0.056968	0.005509	1
Pafah1b2	0.056957	0.002955	1
Selenon	0.056913	0.017237	1

Snapc2	0.056888	0.001499	1
Taf4b	0.056879	0.000431	1
Rab43	0.056824	0.029322	1
1110059E2	0.056754	0.001	1
Xpo1	0.056621	0.044447	1
Zfp664	0.0566	0.089514	1
Phospho2	0.056595	0.000158	1
Nol6	0.056586	0.00175	1
Slc35e4	0.056585	0.023828	1
Pkd2	0.056537	0.000249	1
Tardbp	0.056445	0.011256	1
Fam78a	0.056408	0.005806	1
Eif4e	0.056343	0.11012	1
Cnot9	0.056343	0.000693	1
Sigmar1	0.056311	0.001664	1
Cebpzos	0.05625	0.000932	1
Dimt1	0.056243	0.004101	1
Ift22	0.056214	0.002025	1
Rusc2	0.056172	0.001979	1
Acy1	0.056165	5.63E-05	1
Pfkfb2	0.056062	0.002289	1
Psmd10	0.056022	2.56E-05	0.826006
Atraid	0.056008	0.013917	1
Dennd1a	0.055984	0.320898	1
Dmap1	0.055957	0.001119	1
H2-Oa	0.055937	0.000518	1
Gas5	0.055922	0.858359	1
Ubl5	0.055915	0.358134	1
H2afx	0.055809	0.322635	1
Apoc2	0.05569	0.568736	1
Tecpr1	0.055685	0.002997	1
Klc4	0.05562	1.32E-06	0.042516
Nvl	0.055604	0.002022	1
Zwint	0.055539	0.00052	1
Polr1b	0.055483	0.005186	1
Clcn6	0.055468	0.010325	1
Kdm1a	0.055414	0.036933	1
Eif2b5	0.055398	0.00126	1
Gmcl1	0.055343	0.006458	1
Zfp202	0.055334	0.000104	1
Dbt	0.0553	0.01573	1
Slc35e1	0.055268	0.005963	1
Med12l	0.055218	0.001013	1
Tm2d2	0.055198	0.008769	1
Glb1	0.055192	0.000676	1
Ptov1	0.05519	0.190184	1
Abcf1	0.055103	0.092574	1
D430042O	0.05501	0.0023	1

Tlr3	0.05494	0.000683	1
Tarbp2	0.054891	0.000128	1
Mpv17	0.054875	0.077384	1
Pgrmc2	0.054838	0.000255	1
Mzt2	0.054798	0.014663	1
Mrps2	0.054792	0.002597	1
Ift88	0.054766	3.52E-05	1
Farsb	0.05473	0.005163	1
Ttc13	0.054675	0.001507	1
Shb	0.054666	0.010105	1
Ftsj3	0.054647	0.000165	1
Rcbtb2	0.054601	0.071329	1
Bud23	0.054591	0.006325	1
Pigq	0.054579	0.005276	1
Grpel1	0.05455	0.007641	1
Napa	0.0545	0.011577	1
Slc22a18	0.054486	0.000154	1
Ccdc90b	0.054477	0.001273	1
Coa7	0.054474	2.75E-05	0.88795
Tjp2	0.054429	0.009838	1
Cables2	0.054429	0.072081	1
Birc2	0.05442	0.002561	1
Mrpl21	0.054383	0.006735	1
Rabepk	0.054341	0.00085	1
Gins2	0.054266	0.001288	1
Tmem63b	0.054265	0.001043	1
Pprc1	0.054092	0.000772	1
Coro1b	0.054053	0.282282	1
Eif2b4	0.054019	0.008202	1
Hmg20b	0.053983	0.062055	1
Dusp22	0.05398	0.032638	1
Stoml1	0.053972	0.007385	1
Wdr91	0.053892	0.00028	1
Atp6v0a2	0.053874	0.02199	1
Coq6	0.053839	0.18525	1
Cmtm3	0.053807	0.007274	1
Gm11361	0.053746	0.000892	1
Tmco4	0.053665	0.00071	1
Mfsd8	0.053632	0.110142	1
Gga2	0.053525	0.002053	1
Cnep1r1	0.053492	0.006257	1
Rxrb	0.053491	0.001539	1
Nudt5	0.053471	6.03E-05	1
5031425F1	0.053469	0.005911	1
Sgsm3	0.053424	0.000384	1
Tasp1	0.053362	0.000246	1
Guf1	0.053291	4.19E-05	1
Prmt7	0.053174	0.00032	1

Ndrg1	0.053173	0.062417	1
BC029722	0.053146	0.013841	1
Emc7	0.05314	0.038692	1
Pdap1	0.053122	0.033756	1
Ppcs	0.053096	0.005225	1
F5	0.053068	0.113697	1
Elp5	0.05305	0.021796	1
Exoc5	0.052925	0.001023	1
Mir22hg	0.052793	0.01978	1
Katnb1	0.052759	7.08E-06	0.228738
Nup62	0.052692	0.00287	1
Gm17018	0.052687	0.001501	1
Ahctf1	0.05264	0.000962	1
Ctdsp2	0.052628	0.046187	1
Cdc37	0.052626	0.183011	1
Bloc1s6	0.052578	0.001858	1
G6pc3	0.052552	0.010245	1
Adat2	0.052511	0.009219	1
Sf3a3	0.05232	0.010324	1
Mbnl2	0.052192	0.377724	1
Samd1	0.052171	0.034503	1
Cds1	0.052058	0.000725	1
Oasl2	0.051969	0.095406	1
Lyrm9	0.051956	0.009378	1
Slc12a4	0.051907	0.000257	1
Ndufa7	0.051889	0.092151	1
Tbc1d15	0.051888	0.015736	1
March8	0.051843	0.000819	1
Dnajc12	0.051827	0.002097	1
Josd2	0.051765	0.077539	1
Mtrf1l	0.051713	0.033028	1
Hagh	0.051697	0.002478	1
Pced1b	0.051647	0.000374	1
Ciao1	0.051566	0.014621	1
Gm46224	0.051552	0.054357	1
Prdx2	0.051492	0.30939	1
B020010K1	0.05146	0.002011	1
Dnaja1	0.05136	0.029652	1
Golph3	0.051306	0.056325	1
Rab44	0.051145	0.005771	1
Zzz3	0.051106	0.010714	1
Mier2	0.051074	0.386392	1
Rps9	0.051074	0.553004	1
Pfdn2	0.051071	0.008684	1
Gpatch1	0.050997	0.003733	1
Xrcc6	0.050975	0.010339	1
Taf12	0.050894	0.009388	1
Rps16	0.050834	0.30359	1

Tsnax	0.050783	0.006459	1
Zfp384	0.05069	0.000112	1
Oxa1l	0.050676	0.008893	1
Taf11	0.050631	0.005785	1
Ercc6l2	0.05056	0.002172	1
Snx25	0.050544	0.000731	1
Polr1e	0.050495	0.001327	1
Rtl5	0.050422	0.004374	1
Fnbp1l	0.050402	0.002052	1
Usp31	0.050377	0.004286	1
P2rx7	0.050346	0.133835	1
2210016L2	0.050314	0.005793	1
Psmg2	0.050301	8.17E-05	1
Cep162	0.050294	0.01343	1
Mrpl18	0.050286	0.014014	1
Mob1b	0.050219	0.020452	1
Crtap	0.050188	0.012512	1
Nmd3	0.050086	0.025185	1
Tsg101	0.050082	0.031576	1
Ptdss2	0.050059	0.044076	1
Dnajc16	0.050054	0.000333	1
Cul9	0.050043	0.002899	1
Znhit3	0.050003	0.000178	1
Trim47	0.05	0.004803	1
Slc44a1	0.049996	0.067516	1
Tns3	0.049993	0.169104	1
Tsfm	0.049991	0.002342	1
Tnfsf13os	0.049982	0.166033	1
Rnmt	0.04993	0.005642	1
Fmr1	0.049863	0.002565	1
Pcbp2	0.04986	0.28687	1
Chsy1	0.04984	0.003712	1
Rsph3b	0.049807	0.031263	1
Tbc1d9	0.049771	0.069691	1
H13	0.049768	0.542162	1
Taf1	0.049654	0.032484	1
Mthfr	0.049631	0.0344	1
Tmem165	0.049613	0.013927	1
Cables1	0.049585	0.000909	1
Rnd1	0.049524	0.000906	1
Rfx5	0.049513	0.000604	1
Tmem250-ı	0.049434	0.0292	1
Llph	0.049397	0.063945	1
Bcl2a1c	0.049373	0.001162	1
Usp28	0.049324	0.000173	1
Gcdh	0.049295	0.001264	1
Pi4k2b	0.049257	0.004454	1
Mapk1ip1	0.049205	0.01474	1

2300009A(	0.049155	0.023034	1
Capn10	0.049136	0.003768	1
Mtfmt	0.049108	5.56E-07	0.017953
Anapc13	0.049081	0.177046	1
Fahd2a	0.049048	0.027724	1
Ganab	0.04901	0.025956	1
Pstpip2	0.048923	0.090527	1
Adora2b	0.048916	0.201249	1
Mprip	0.048906	0.06893	1
Gpr137	0.048838	0.005175	1
Abcc3	0.048821	0.002676	1
Arrb2	0.048771	0.10289	1
Zfp358	0.048706	0.003603	1
Adk	0.048491	0.01471	1
Fam173a	0.048443	0.012343	1
Phc2	0.048438	0.000225	1
Ddx56	0.048425	0.00055	1
Btg1	0.048411	0.589025	1
P3h1	0.048408	0.004028	1
Prkrip1	0.048394	0.019806	1
Glo1	0.048377	0.00083	1
Hspa14	0.04834	0.027896	1
Zbtb38	0.048316	0.003105	1
Atp6v1f	0.048302	0.375545	1
Tars2	0.048227	0.000222	1
Zfp319	0.048221	0.000805	1
Med4	0.048216	0.011268	1
Ankzf1	0.048213	0.284734	1
Kti12	0.048142	0.042615	1
Cenpx	0.048134	0.097494	1
Nit2	0.048109	0.003625	1
Shld2	0.048098	0.040058	1
Tmem42	0.048076	0.005482	1
Snrnp27	0.047978	0.032406	1
H6pd	0.047917	0.008439	1
Sap18b	0.047878	0.01674	1
Gadd45a	0.04781	0.008546	1
Pdcl3	0.047744	0.013413	1
Ipo13	0.047735	0.031099	1
Rpap3	0.047682	0.001086	1
Dhrs7	0.047664	0.592143	1
Mcee	0.047657	0.004759	1
Pcna	0.047609	0.205676	1
Kank3	0.0476	0.001324	1
Ube2a	0.047576	0.32428	1
Slc11a2	0.047531	0.004476	1
Vps33a	0.047486	0.010547	1
Dtnbp1	0.047453	0.010748	1

Osgin1	0.047447	0.006021	1
Ptpn4	0.047435	0.031799	1
Mycbp	0.047428	0.019568	1
Ilvbl	0.047416	0.000804	1
Mrpl41	0.047399	0.001909	1
Decr2	0.047286	0.012857	1
Mrpl36	0.047253	0.039133	1
Tsc1	0.047078	0.021044	1
Fntb	0.046943	0.004449	1
Zfp472	0.046866	0.091288	1
Sco1	0.046836	0.004826	1
Adck2	0.046723	0.196825	1
Atp8b2	0.046715	0.152625	1
Tnfsf14	0.04662	0.003369	1
Sac3d1	0.046507	0.020594	1
Ube2i	0.046485	0.316591	1
Gid4	0.04645	0.002868	1
Gtf2f2	0.046444	0.008	1
Ppfia1	0.046402	0.012084	1
Pex19	0.046347	0.077773	1
Heatr5a	0.046311	0.08835	1
Cct7	0.046311	0.208099	1
Pla2g15	0.046268	0.001625	1
Ndc1	0.046227	0.006607	1
Commd3	0.046215	0.052956	1
Champ1	0.046144	0.031835	1
Tmem9	0.046132	0.00752	1
Chtop	0.046114	0.001881	1
Fam76a	0.046106	2.6E-05	0.838891
Trim35	0.046043	0.005422	1
Wiz	0.046028	0.000285	1
Fastk	0.046025	0.000353	1
Sypl	0.046016	0.267421	1
Nasp	0.045992	0.050679	1
Khdrbs1	0.045981	0.54887	1
Mtg1	0.045966	0.030905	1
Trio	0.045918	0.043472	1
Max	0.045888	0.021628	1
Lpgat1	0.045835	0.000402	1
Rpl18	0.045757	0.207232	1
Ube4b	0.045745	0.031611	1
Map3k4	0.045732	0.001522	1
Wrnip1	0.045697	0.000657	1
Cdk6	0.045653	0.074668	1
Rpa3	0.045618	0.007575	1
Proser1	0.045612	0.002854	1
Gtf3c5	0.045575	0.006241	1
Wdr92	0.045571	0.033788	1

Irf7	0.045513	0.291459	1
Gm47882	0.045498	0.007868	1
Tceanc2	0.045481	0.01341	1
Rps3a1	0.045452	0.469966	1
Hps6	0.045388	0.001731	1
Tent2	0.045368	0.386474	1
Ccdc127	0.045238	0.026928	1
Scap	0.045163	0.007692	1
Zfyve21	0.045127	0.000956	1
Rbm43	0.045097	0.007624	1
Vmp1	0.045088	0.301141	1
Cd99l2	0.044941	0.158044	1
Fbxl14	0.04493	0.054503	1
Xpot	0.044918	0.003207	1
Zdhhc5	0.044868	0.014045	1
Khsrp	0.044866	0.331448	1
Rad54l2	0.044793	0.007006	1
Galc	0.044771	0.006821	1
Tnfrsf12a	0.044689	0.000741	1
0610010F(	0.044656	0.002113	1
Nup160	0.044633	0.082532	1
Nup37	0.044535	0.001087	1
Nat10	0.044502	0.001856	1
Tvp23b	0.044444	0.052939	1
Nqo2	0.044346	0.001401	1
Uqcc1	0.044251	0.000854	1
Sptssa	0.04417	0.013943	1
Rexo4	0.044098	0.04672	1
Rnf19b	0.044091	0.031982	1
Gypc	0.044018	0.011463	1
Plp2	0.043984	0.010366	1
Arhgap5	0.043951	0.06104	1
Ccs	0.043934	0.001554	1
Tmem138	0.043893	0.003819	1
Tpp1	0.043886	0.001368	1
Cox19	0.043872	0.004054	1
Uros	0.043754	0.017371	1
Vps72	0.043629	0.183798	1
Eif4ebp3	0.043609	0.10525	1
B3gntl1	0.043575	0.005122	1
Rpp14	0.043574	0.009872	1
Mesd	0.043482	0.00175	1
Ahsa1	0.043404	0.025527	1
Cabin1	0.043376	0.005699	1
Runx1	0.04336	0.291082	1
Rfxap	0.043298	0.002337	1
Abhd5	0.043258	0.002285	1
Itpripl1	0.043227	0.002827	1

Prmt1	0.043224	0.022102	1
Mrps10	0.043204	0.047486	1
Cav2	0.04319	0.007771	1
Qsox1	0.043148	0.041705	1
Agk	0.043132	0.000226	1
Acaca	0.043109	0.04183	1
Cuedc2	0.042966	0.06971	1
Prr14l	0.04296	0.091145	1
Mrps26	0.042959	0.027693	1
Ifrd1	0.04294	0.001137	1
Chchd7	0.042915	0.009102	1
Spout1	0.042871	0.001064	1
Cdpf1	0.042864	0.000238	1
Snhg17	0.042811	0.001423	1
Atp10a	0.042805	0.063416	1
Rbbp4	0.042675	0.044814	1
Zfp768	0.042607	0.012654	1
Fam207a	0.042597	0.00224	1
Ccdc107	0.042557	0.003128	1
Ppp2ca	0.042529	0.029097	1
2510009E(	0.04237	0.012149	1
Tmem161b	0.042232	0.326908	1
Nfix	0.042214	0.029957	1
Alg11	0.042093	0.001253	1
Pde4d	0.042069	0.016034	1
Ost4	0.041993	0.384622	1
Paox	0.041989	0.000888	1
Habp4	0.041952	0.034259	1
Traf6	0.041913	0.023809	1
Sc5d	0.041864	0.048126	1
Mrps6	0.041847	0.00236	1
Hrh2	0.041839	0.001332	1
Tram2	0.041796	0.01652	1
Sfmbt1	0.041744	0.00095	1
Bckdk	0.0417	0.003779	1
Stxbp1	0.041616	0.000304	1
Stx17	0.041596	0.178788	1
Chaf1a	0.041325	0.004607	1
Rps13	0.041267	0.406759	1
Foxred1	0.041264	0.184366	1
Ankmy2	0.041232	0.163102	1
Errfi1	0.041224	0.008932	1
Trmt61b	0.041197	0.028188	1
Agap1	0.041156	0.040764	1
Nup214	0.041121	0.038373	1
Pycr2	0.0411	0.026146	1
Alkbh6	0.041015	0.01189	1
Pin4	0.041014	0.030202	1

Tmem161a	0.040915	0.024353	1
Akr7a5	0.040908	0.217282	1
Kif3b	0.040877	0.001886	1
Cops7a	0.04082	0.016153	1
Isoc1	0.040818	0.011277	1
Thra	0.040748	0.01625	1
Snrpa1	0.040731	0.014168	1
Tsr1	0.040644	0.01137	1
Ptbp2	0.040592	0.009654	1
Flad1	0.040472	0.000417	1
Slc39a7	0.040451	0.023085	1
Tpmt	0.04043	0.044125	1
Tlr11	0.040374	0.008792	1
Letmd1	0.040373	0.046877	1
Mboat1	0.040367	0.015103	1
Ccdc22	0.040252	0.061505	1
Mogs	0.040222	0.043884	1
Ppp1r21	0.040132	0.248217	1
Clec10a	0.040044	0.000123	1
Eml4	0.039996	0.282711	1
Ccnk	0.039994	0.004999	1
Ldb1	0.039983	0.009298	1
Bak1	0.039967	0.328578	1
Nudt16l1	0.039936	0.00051	1
Pdhx	0.039874	0.000953	1
Zc3h6	0.039746	0.075816	1
AI837181	0.039694	0.008963	1
Bub3	0.039674	0.034609	1
Srek1	0.039672	0.003915	1
Pskh1	0.039631	0.0442	1
Nol8	0.039546	0.013304	1
Utp18	0.039494	0.012953	1
3110040N1	0.039478	0.005941	1
Zfp36l1	0.039383	0.228048	1
Prr12	0.03926	0.013698	1
Dhx32	0.039128	0.012997	1
Faap100	0.039094	0.021421	1
Samm50	0.039047	0.270271	1
Cluap1	0.038863	0.002764	1
Tmem237	0.038826	9.58E-05	1
Mettl6	0.038825	0.000802	1
Babam1	0.038801	0.002905	1
Tent5a	0.038756	0.031806	1
Ofd1	0.038665	0.000993	1
Sqstm1	0.038637	0.051032	1
Sfxn3	0.038635	0.117953	1
Ube2q2	0.038621	0.044044	1
2310057M	0.038562	0.029589	1

Snf8	0.03852	0.123401	1
Gabpa	0.038487	0.024005	1
Prpf19	0.038485	0.078065	1
Prmt5	0.038428	0.005616	1
Cbr1	0.038424	0.049066	1
Bpnt1	0.038396	0.011895	1
Hook2	0.038388	0.035401	1
Unc119b	0.038354	0.003565	1
Ercc3	0.038348	0.021713	1
Wdr74	0.038319	0.003939	1
Tmem80	0.038275	0.007324	1
Zfp800	0.03803	0.330566	1
Acp6	0.037904	0.150513	1
Tpm3	0.037833	0.176042	1
Sertad1	0.037785	0.003623	1
Gstk1	0.037743	0.00114	1
D530033B1	0.037742	0.027702	1
Dennd3	0.037733	0.003198	1
Tmco6	0.03769	0.012879	1
Tut4	0.037676	0.557991	1
Haus3	0.037632	0.029212	1
Scarb2	0.037606	0.048016	1
Sdhaf2	0.037602	0.016047	1
Dnaja2	0.037573	0.062633	1
Fbxw9	0.03752	0.021192	1
Gm10131	0.037495	0.011144	1
Uap1	0.037491	0.00406	1
Il3ra	0.037404	0.032849	1
Mfsd11	0.0374	0.019684	1
2010320M	0.037306	0.000407	1
Acadvl	0.037229	0.000808	1
Nek1	0.037195	0.014525	1
Hsd17b4	0.037171	0.630196	1
Map1lc3a	0.037149	0.017197	1
Ehbp1	0.037143	0.010099	1
Ift172	0.037139	0.00121	1
Snap47	0.037127	0.015236	1
Slc30a1	0.037117	0.013893	1
Actr1a	0.037072	0.228946	1
BC002059	0.037063	0.011735	1
Brf1	0.03702	0.046258	1
Abitram	0.037016	0.007029	1
Tada2a	0.036945	0.095206	1
Spns1	0.036898	0.009125	1
Cbx1	0.036896	0.005063	1
Polr3e	0.036841	0.005031	1
Marc2	0.036838	0.031113	1
Fasn	0.036738	0.023206	1

Tpra1	0.036549	0.01502	1
Lipo3	0.036478	0.003904	1
Smg8	0.036469	0.02591	1
Eefsec	0.036392	0.17132	1
Nupl2	0.03637	0.102526	1
Cib2	0.036357	0.21135	1
Tlr2	0.036344	0.006042	1
Dmac1	0.036303	0.026415	1
Zfp456	0.036233	0.001625	1
Dbr1	0.036227	0.003469	1
Rft1	0.036214	0.00799	1
Dpp7	0.0362	0.000453	1
Zc3h7b	0.036165	0.039482	1
Tgm1	0.03616	0.00021	1
Txnip	0.036125	0.673102	1
Usb1	0.036046	0.002942	1
Ppp1r11	0.035992	0.134026	1
Pnpt1	0.035987	0.063991	1
Nudt18	0.035982	0.005335	1
Tubgcp5	0.035972	0.005857	1
Bccip	0.035954	0.004964	1
Tmem39a	0.035904	0.016637	1
Thoc6	0.035901	0.007462	1
Necap2	0.035816	0.226073	1
Zfp444	0.035807	0.023838	1
Ascc2	0.035799	0.022727	1
Yif1a	0.035736	4.33E-06	0.13995
Apoo	0.035726	0.04688	1
Gatd1	0.035725	0.004427	1
Tom1l2	0.035677	0.017143	1
Top3b	0.035653	0.183613	1
Praf2	0.035649	3.35E-05	1
Rbm15b	0.03563	0.008815	1
Atp6ap1	0.035623	0.4563	1
Dbf4	0.035529	0.004108	1
Yipf4	0.0355	0.189153	1
Rabif	0.035488	0.680769	1
Zxdc	0.035451	0.011474	1
Alg14	0.035435	0.001615	1
Pef1	0.035433	0.002064	1
Pcgf5	0.035401	0.017382	1
Asf1a	0.035368	0.060572	1
Pdcl	0.035366	0.57133	1
Mmp25	0.035336	0.000639	1
Sub1	0.035295	0.148563	1
Slfn3	0.03528	0.01976	1
Mrpl32	0.035275	0.01919	1
Glrx5	0.035246	0.066872	1

Rpl30	0.035243	0.482217	1
Clcc1	0.035239	0.044297	1
Taf4	0.035172	0.069605	1
Maip1	0.035166	0.010534	1
Fgd2	0.03509	0.400688	1
Dnajb4	0.035086	0.056986	1
Stt3a	0.035083	0.021379	1
Mcm6	0.035066	0.013623	1
Psme2b	0.03506	0.015134	1
Tyk2	0.035027	0.306882	1
Pdhb	0.035012	0.000439	1
Cstf1	0.034881	0.0013	1
Uckl1	0.03486	0.004781	1
Eftud2	0.03484	0.115229	1
Nars2	0.034811	0.030091	1
Adam15	0.034676	0.052413	1
Pwwp2b	0.034613	0.002515	1
Ddx46	0.034608	0.0431	1
Usp46	0.03451	0.00414	1
Inpp5b	0.034505	0.001413	1
Pkp4	0.034336	0.003653	1
Caml	0.034323	0.000146	1
Arsk	0.034322	0.004708	1
Cmc2	0.034314	0.003639	1
Ogfr	0.034309	0.262902	1
Ypel5	0.0343	0.026751	1
Vps26a	0.034279	0.082101	1
Zbtb25	0.034221	0.021246	1
Zfp318	0.034195	0.006916	1
Rgs10	0.034163	0.008857	1
Rnf157	0.034154	0.003502	1
Dus1l	0.034141	0.004728	1
Hnrnpa2b1	0.034127	0.368186	1
Pde2a	0.03412	0.153803	1
Arl2bp	0.034078	0.020636	1
Gm14325	0.034021	0.074572	1
Ndufa3	0.033997	0.16662	1
Tmem185b	0.033985	0.004469	1
Atp6v0b	0.033934	0.202415	1
Ttyh3	0.033874	0.075495	1
Csf1	0.033873	0.021339	1
Slc25a19	0.033842	0.028096	1
Telo2	0.033807	0.003305	1
Ap2a1	0.033806	0.012768	1
Cdk5rap3	0.033776	0.0006	1
Hmox2	0.033733	0.137462	1
Czib	0.033715	0.001883	1
Abt1	0.033656	0.006397	1

Srxn1	0.033643	0.005363	1
Ucp2	0.033639	0.734661	1
Galnt3	0.03361	0.060949	1
Upf1	0.033564	0.000636	1
Ginm1	0.033455	0.005038	1
Uck1	0.033384	0.007459	1
Rwdd4a	0.033374	0.003649	1
Scrn3	0.033342	0.476356	1
Gtf3c3	0.033322	0.03456	1
Brix1	0.033316	0.005922	1
Srebf1	0.033285	0.022226	1
Ciz1	0.033241	0.056671	1
Ndufaf2	0.033227	0.004622	1
Abcd4	0.033208	0.045761	1
Gm11131	0.033112	0.053939	1
Csnk2a2	0.033107	0.002281	1
Lpp	0.033104	0.201419	1
Srbd1	0.033099	0.156291	1
Rnf10	0.032968	0.046804	1
Taf1c	0.032918	0.010361	1
Atp11b	0.032838	0.032495	1
Styx	0.032817	0.025714	1
Cfl2	0.032802	0.034238	1
Use1	0.032775	0.011597	1
Eif4b	0.032752	0.064641	1
Cln6	0.032722	0.004763	1
Etfrf1	0.032707	0.015687	1
Bnip2	0.032687	0.197387	1
Nsfl1c	0.032601	0.003971	1
Fermt3	0.032598	0.301705	1
Nup155	0.032582	0.021285	1
Tasor2	0.03257	0.133996	1
6330418K(	0.03255	0.027903	1
6530409C:	0.032511	0.019136	1
Kif19a	0.032481	0.000911	1
Wnk1	0.032425	0.606432	1
Atg4b	0.032393	0.051661	1
Fars2	0.032393	0.005086	1
Fam53a	0.032378	0.028506	1
Cep78	0.032376	0.100855	1
Rnf185	0.032368	0.004175	1
Ccdc25	0.032316	0.006931	1
Efcab14	0.032311	0.015793	1
Golim4	0.032263	0.016211	1
Dffb	0.032258	0.105778	1
Rps6ka4	0.032182	0.000717	1
Sirt6	0.032122	0.049657	1
Supv3l1	0.032059	0.023439	1

Abcb8	0.032009	0.0022	1
Aasdhppt	0.031978	0.021551	1
Sf3b6	0.031869	0.205481	1
Mpi	0.031818	0.015417	1
Ascc1	0.031808	0.872755	1
Ckap5	0.031805	0.005577	1
Ciao3	0.031775	0.000217	1
Elp4	0.031757	0.001755	1
Med21	0.031751	0.023226	1
Oprm1	0.031749	0.016976	1
Zc3hc1	0.031745	0.096538	1
Arhgef7	0.031711	0.002922	1
Lin54	0.031664	0.003355	1
Zkscan6	0.031629	0.010989	1
Fcf1	0.031614	0.069116	1
Ncoa6	0.031597	0.002383	1
Pacs2	0.031593	0.000737	1
Rffl	0.031584	0.011434	1
Slc36a4	0.03156	0.197944	1
Ddah1	0.031523	0.004964	1
Pgm2	0.03145	0.006223	1
Ash2l	0.031254	0.01702	1
Ddx10	0.031214	0.009697	1
Rbm8a	0.031193	0.308279	1
Nprl3	0.031161	0.040345	1
Adprhl2	0.031155	0.075611	1
Ciao2b	0.031008	0.039766	1
2610020C(	0.030996	0.030332	1
Zfand1	0.030976	0.042151	1
Spata5l1	0.030933	0.039353	1
Vps9d1	0.030845	0.098296	1
Tmem141	0.030822	0.012198	1
Saal1	0.030811	0.041015	1
Rdh14	0.030775	0.000648	1
Rbfa	0.030773	0.015374	1
Dhdh	0.030772	0.009809	1
Mgat4b	0.030761	0.00044	1
Egln1	0.030729	0.009313	1
Ivd	0.030669	0.001395	1
Mtmr9	0.030572	0.016438	1
Setd3	0.030559	0.020584	1
Srd5a3	0.030509	0.011466	1
Ift46	0.030489	0.033528	1
Slc25a16	0.030462	0.001223	1
Mms22l	0.030461	0.040961	1
Psmd14	0.030379	0.006237	1
Cisd3	0.030363	0.00153	1
Tceal9	0.030355	0.104409	1

Fbxl15	0.030293	0.005533	1
Traf2	0.030234	0.014431	1
Lrch1	0.030191	0.091509	1
Hpcal1	0.03016	0.246881	1
Dnajc21	0.030052	0.006466	1
Tent4a	0.030013	0.015151	1
Dusp28	0.030006	0.008755	1
Myl12a	0.030005	0.125597	1
Flot1	0.029903	0.093033	1
Fastkd1	0.029897	0.040406	1
Snapc4	0.029829	0.046696	1
E430024P1	0.029727	0.0715	1
Fbxw7	0.029727	0.001715	1
Nsmf	0.029721	0.033028	1
Map2k3	0.029696	0.037604	1
Tmem185a	0.029562	0.021104	1
Hspa4	0.029512	0.18141	1
Mks1	0.02951	0.017919	1
Aamp	0.029371	0.003743	1
Prorp	0.029357	0.013071	1
Eif2ak1	0.029252	0.445742	1
Cep152	0.029224	0.487239	1
Polr3a	0.029174	0.088209	1
Zswim4	0.029131	0.046833	1
Gm16229	0.029121	0.012832	1
Yju2	0.029104	0.011055	1
Dock7	0.029053	0.002248	1
Arhgef11	0.028999	0.008123	1
Rnf5	0.028997	0.016332	1
Exo5	0.028968	0.003027	1
Cep70	0.028962	0.10057	1
Rab11fip1	0.028961	0.036867	1
Pcid2	0.028943	0.122654	1
Tysnd1	0.028917	0.000323	1
Narf	0.02889	0.038898	1
Sirt2	0.028874	0.004127	1
Abhd17c	0.02883	0.018864	1
Tmem184c	0.028771	0.293849	1
Hadha	0.028747	0.120864	1
Smim24	0.028666	0.015499	1
Dgcr6	0.02862	0.03215	1
2310061I0	0.028583	0.029199	1
Jmjd1c	0.028582	0.107856	1
Nap1l4	0.028527	0.187702	1
Dars	0.028518	0.170914	1
D10Wsu10	0.028517	0.111815	1
Ocrl	0.028499	0.126469	1
Cstf2	0.028496	0.044245	1

Etfbkmt	0.028444	0.130317	1
Tgds	0.028414	0.004822	1
Thap12	0.028402	0.007077	1
Gm9725	0.028383	0.055295	1
9530082P2	0.028279	0.000393	1
Gna12	0.028264	0.005566	1
Gm15821	0.028253	0.046733	1
Szt2	0.02824	0.018062	1
Tmbim6	0.028226	0.171675	1
Amz2	0.028184	0.016529	1
Zbtb11os1	0.028114	0.021758	1
Mfge8	0.028035	0.051432	1
Tmed8	0.028034	0.015369	1
Fam149b	0.028029	0.003355	1
Gmppa	0.028023	0.093977	1
Pnpla6	0.028003	0.057317	1
2700062C0	0.027983	0.029937	1
Nsdhl	0.027958	0.001393	1
Dynlt3	0.027952	0.0432	1
Rcbtb1	0.027921	0.004203	1
Pex3	0.027872	0.472539	1
Ykt6	0.027854	0.040302	1
1600002K0	0.027827	0.001521	1
Mpdu1	0.027724	0.040297	1
Rab1b	0.027722	0.162407	1
Ap2a2	0.027709	0.109413	1
Atrip	0.027637	0.02043	1
Dtx3l	0.027535	0.029955	1
Gbe1	0.027432	0.013597	1
Tmem219	0.027428	0.03967	1
Cog7	0.027398	0.357457	1
Ube2e1	0.027394	0.021004	1
Zfp157	0.02738	0.129905	1
Itpa	0.027376	0.060712	1
Zmpste24	0.027375	0.029638	1
Rps6ka5	0.027357	0.012249	1
Nepro	0.027356	0.026277	1
Tlr6	0.027306	0.016902	1
Gm4316	0.027285	0.069436	1
Osbpl11	0.027265	0.072134	1
Reep4	0.027221	0.003457	1
Ccdc167	0.027214	0.024027	1
Zscan21	0.027142	0.035916	1
Rab5a	0.02711	0.034713	1
Nacc2	0.027084	0.018026	1
Abcb10	0.027078	0.028729	1
Mief1	0.026915	0.174505	1
Casz1	0.026904	0.055927	1

Atr	0.026864	0.00388	1
Csnk1g3	0.026863	0.047968	1
Vamp7	0.026813	0.001223	1
Slc22a4	0.026809	0.01722	1
Zfp654	0.026702	0.042414	1
Eogt	0.026667	0.155853	1
Gstz1	0.026662	0.001293	1
Gnptg	0.026588	0.04453	1
Gnl3l	0.026565	0.051052	1
Prdm1	0.026558	0.030457	1
Med20	0.02653	0.148258	1
Zbtb17	0.026514	0.023953	1
Kyat3	0.026508	0.073541	1
Coq10a	0.026383	0.020484	1
Katna1	0.026335	0.211444	1
Sf3b5	0.026259	0.110324	1
2610002M	0.026215	0.000374	1
Dnaja3	0.02621	0.002048	1
Scfd1	0.026199	0.064207	1
Rhbdd3	0.026153	0.054249	1
Nt5dc3	0.026149	0.015659	1
Dhx34	0.026108	0.113095	1
Tmx2	0.026095	0.004271	1
Trub2	0.026048	0.050702	1
Lrrc47	0.026023	0.004052	1
1110065P2	0.025938	0.006818	1
Gemin7	0.025861	0.128208	1
Pank4	0.025837	0.015015	1
Ndufb3	0.025707	0.173799	1
Ulbp1	0.025694	0.00305	1
Btrc	0.025647	0.153243	1
Ubqln1	0.025617	0.02541	1
Vac14	0.025613	0.011458	1
Sh2b1	0.02558	0.069305	1
Polr1c	0.025561	0.001135	1
Xpnpep1	0.025531	0.068693	1
Ikbke	0.025405	3.18E-05	1
Dpagt1	0.025384	0.001973	1
Pign	0.025369	0.052281	1
Gdap2	0.025341	0.343346	1
Serhl	0.025221	0.029951	1
Usp24	0.025182	0.030227	1
Zfp993	0.025169	0.009373	1
Cpne8	0.025088	0.008677	1
Nemp1	0.025074	0.153986	1
Bcl2l12	0.025045	0.339391	1
Neil1	0.025041	0.021139	1
Ccdc6	0.02504	0.004945	1

Tsr3	0.024974	0.004614	1
Bcl9l	0.02496	0.10805	1
Tpgs1	0.024948	0.105258	1
Zfp934	0.024907	0.102017	1
Stk35	0.024862	0.837285	1
Creld1	0.024856	0.042492	1
Git1	0.024811	0.019116	1
Ss18l2	0.024811	0.003811	1
Trmt10a	0.024796	0.082352	1
Gm8739	0.024753	0.030691	1
P4ha1	0.024728	0.246772	1
Fbxl8	0.024689	0.377312	1
Exoc8	0.024681	0.014076	1
BC005561	0.024625	0.033126	1
Panx1	0.024595	0.027198	1
Stom	0.024541	0.088495	1
Snd1	0.024528	0.181488	1
Naa40	0.024516	0.001981	1
Rmnd5a	0.024494	0.046095	1
Lnpk	0.024447	0.098684	1
Ubxn2b	0.024355	0.087185	1
Thumpd2	0.024344	0.018003	1
Epm2aip1	0.024212	0.03267	1
Manba	0.024112	0.048499	1
Tirap	0.024111	0.101311	1
Samd8	0.024102	0.229555	1
Fance	0.024095	0.005163	1
Desi1	0.024091	0.272717	1
Poc5	0.024031	0.116012	1
Tpgs2	0.024021	0.013394	1
Xpo4	0.024007	0.010316	1
Atn1	0.023953	0.033179	1
Lpcat1	0.023953	0.005534	1
Ssh3	0.02384	0.013302	1
Cflar	0.023837	0.042014	1
Exd2	0.023746	0.018696	1
Slc37a4	0.023727	0.152387	1
Rprd1b	0.023611	0.021021	1
Nle1	0.02356	0.021332	1
Maea	0.023435	0.101185	1
Ahcy	0.023402	0.029244	1
Zfp24	0.02339	0.043498	1
Nup50	0.023338	0.005586	1
Copz1	0.023336	0.422222	1
Trip10	0.023311	0.023848	1
Rpl21	0.023305	0.707055	1
Platr25	0.023218	0.205988	1
A330023F2	0.023199	0.009129	1

Spaca9	0.023191	0.001403	1
Tiprl	0.0231	0.046243	1
Slc2a6	0.023083	0.128422	1
Nras	0.023063	0.263414	1
Ubap2l	0.022987	0.291203	1
Cul4a	0.022919	0.005066	1
1700021F(	0.022897	0.025401	1
Utp25	0.022896	0.002714	1
Fem1b	0.022896	0.038266	1
Fbxo42	0.022823	0.276644	1
Gpat4	0.022755	0.059656	1
Fam114a1	0.022718	0.019492	1
Tmem214	0.022654	0.008949	1
Adipor2	0.022592	0.537839	1
Ecd	0.022583	0.011529	1
Mphosph9	0.022576	0.028147	1
Kansl2	0.022525	0.041229	1
Eif5b	0.022481	0.091955	1
Gstt2	0.022474	0.190539	1
Cops2	0.022428	0.111057	1
Armh3	0.022409	0.027938	1
Tbk1	0.022322	0.006684	1
Golga5	0.022243	0.002151	1
Mmut	0.022228	0.135723	1
1190007I0	0.022213	0.001564	1
Aven	0.022159	0.049689	1
St13	0.022137	0.015713	1
Tnip3	0.022135	0.229504	1
Dcaf15	0.022131	0.011195	1
Clock	0.022091	0.033767	1
Myef2	0.022071	0.005301	1
Gcc1	0.022051	0.122167	1
Ap3s1	0.02204	0.060298	1
Prpf40a	0.022039	0.10752	1
Vps35l	0.022036	0.097239	1
Pip5k1a	0.022007	0.052434	1
Ppip5k1	0.021973	0.015051	1
Lig1	0.021968	0.094175	1
0610010K:	0.021872	0.00292	1
Hist1h1c	0.021868	0.483643	1
G3bp2	0.021841	0.363364	1
Erf	0.021841	0.008292	1
Spg20	0.021817	0.035392	1
Snrnp35	0.021783	0.009023	1
Prmt6	0.021776	0.074412	1
Tbcd	0.021722	0.00677	1
Acp2	0.021659	0.01941	1
Arl5b	0.021641	0.031622	1

Tomm70a	0.021609	0.001676	1
2310022B(	0.021581	0.03911	1
Mtmr14	0.021566	0.177997	1
Sh3bp5l	0.021502	0.027087	1
Tusc2	0.021464	0.007016	1
Nacc1	0.021348	0.231934	1
Nudt1	0.021268	0.092234	1
Abhd14b	0.021259	0.096918	1
Psmc2	0.021254	0.003628	1
Timm29	0.021252	0.239967	1
Ccnl1	0.021235	0.127955	1
Eaf1	0.021231	0.012073	1
Smim8	0.021222	0.00107	1
Sec22a	0.021221	0.021295	1
4930402H:	0.021212	0.042952	1
Pmpca	0.021164	0.025906	1
Arhgap22	0.021099	0.604873	1
Taf1a	0.021077	0.003427	1
Vdac3	0.020909	0.02865	1
Rapgef2	0.020899	0.082842	1
Fam219a	0.020892	0.005112	1
Eif4a3	0.020803	0.012018	1
Trmt2b	0.020745	0.000567	1
Lmf2	0.020736	0.010162	1
Dnttip2	0.020731	0.054629	1
Med10	0.020688	0.004289	1
Gm16286	0.020636	0.178602	1
Mnat1	0.020597	0.069791	1
Commd2	0.02057	0.159096	1
Ercc8	0.020488	0.001048	1
Smim4	0.020471	0.076914	1
Sars2	0.020456	0.001046	1
Sft2d3	0.020453	0.093702	1
Ttf2	0.020402	0.004072	1
Kdm6a	0.020383	0.010711	1
Prkra	0.020377	0.571096	1
Fam217b	0.02029	0.059908	1
Eif2s3x	0.020281	0.001766	1
Nipa2	0.020263	0.258425	1
Usp19	0.020259	0.104128	1
Mnt	0.020195	0.061296	1
Borcs7	0.020194	0.061383	1
Twf2	0.02019	0.057643	1
Tm9sf1	0.020185	0.003972	1
Scyl1	0.020105	0.028461	1
Lsm3	0.020082	0.025727	1
Gm12216	0.019897	0.009149	1
Cbwd1	0.019863	0.167047	1

Cpsf2	0.019842	0.108634	1
Gatad1	0.019814	0.03665	1
Mrpl49	0.019785	0.01446	1
Msto1	0.019652	0.054184	1
Pfkfb3	0.019632	0.071036	1
Dhcr24	0.019621	0.697295	1
Nfs1	0.019549	0.014431	1
Ogg1	0.019441	0.016537	1
Stx6	0.01938	0.313998	1
Zfat	0.019362	0.000681	1
Acsf2	0.019354	0.033213	1
Nucb2	0.019321	0.009983	1
Crtc2	0.019299	0.032478	1
Ilkap	0.019294	0.042967	1
Aida	0.019268	0.02411	1
Mccc1	0.019247	0.217626	1
Kdm4a	0.019243	0.019753	1
Trp53inp1	0.019102	0.136136	1
Flnb	0.019074	0.003601	1
Rbmx	0.018991	0.050616	1
2410002F2	0.018926	0.182631	1
Hirip3	0.018923	0.002873	1
Kctd5	0.01884	0.051934	1
Rpl36	0.018837	0.896432	1
Paxip1	0.018821	0.011053	1
Fam114a2	0.018794	0.015501	1
Dnal4	0.018744	0.003788	1
Wdr61	0.018672	0.034258	1
Nabp2	0.018669	0.404484	1
Galns	0.018668	0.094266	1
Rheb	0.018657	0.068781	1
Nosip	0.018652	0.011945	1
Ube2k	0.018585	0.090887	1
Ing2	0.018467	0.954231	1
Top3a	0.018459	0.020678	1
Washc2	0.01844	0.055179	1
Afg3l2	0.018431	0.007868	1
Il1rap	0.018377	0.054307	1
Zdhhc13	0.01832	0.017131	1
Tbcel	0.018195	0.004121	1
Lacc1	0.018127	0.028598	1
Nr2c2	0.01812	0.045007	1
Vps13d	0.018034	0.090239	1
Plaa	0.017921	0.041532	1
Xab2	0.017881	0.744691	1
Camsap1	0.017831	0.070779	1
Noa1	0.017823	0.126314	1
Msh6	0.017809	0.128308	1

Syncrip	0.017713	0.125217	1
Pramef8	0.017697	0.059011	1
Isg20l2	0.017687	0.012018	1
Pola2	0.017654	0.019282	1
Itprid2	0.017625	0.095102	1
Tmem29	0.017624	0.028843	1
Ago3	0.017578	0.265121	1
Hddc2	0.017552	0.000968	1
Cenpt	0.017544	0.009252	1
Mrps25	0.017537	0.001407	1
Gm2629	0.017523	0.003026	1
4932438A1	0.01752	0.123931	1
Sesn2	0.017516	0.008821	1
Decr1	0.017435	0.071349	1
Ppp2cb	0.017406	0.072959	1
Nop9	0.017372	0.011573	1
Tcf3	0.017344	0.078771	1
Tmf1	0.017329	0.240931	1
Pabpc4	0.017318	0.028511	1
Tnpo3	0.017311	0.089989	1
Maco1	0.017301	0.157397	1
Csnk2a1	0.017287	0.38644	1
Cul4b	0.017266	0.020185	1
Llgl1	0.017262	0.006857	1
Bud13	0.01723	0.021577	1
Gpr84	0.017117	0.007531	1
Nop16	0.017116	0.01118	1
Pisd	0.017104	0.188772	1
Srsf2	0.017067	0.582789	1
Klhdc10	0.01706	0.252045	1
Fzd7	0.017042	0.184668	1
Mrps31	0.01703	0.005367	1
Syce2	0.017018	0.003977	1
Nkapd1	0.016995	0.050752	1
Cyp20a1	0.016985	0.01598	1
Bloc1s5	0.016928	0.008755	1
E2f1	0.01692	0.063153	1
Mcm3ap	0.016891	0.100786	1
G2e3	0.016878	0.027637	1
Mtfr1l	0.01681	0.012629	1
Gm9844	0.016786	0.132366	1
Ccdc47	0.016736	0.32974	1
Hsd17b7	0.016734	0.134745	1
Nanp	0.016649	0.005921	1
Zc3h14	0.016576	0.012973	1
Slc25a10	0.016541	0.040582	1
Asnsd1	0.016356	0.021738	1
Osbp	0.016344	0.024336	1

Phf10	0.016303	0.002119	1
Rad18	0.016233	0.004341	1
Denr	0.016212	0.058987	1
4931406C(	0.016179	0.014696	1
Pip4k2c	0.016114	0.114702	1
Fkrp	0.016079	0.029505	1
Rpusd1	0.016072	0.007259	1
Mrpl10	0.016069	0.160304	1
Chtf8	0.016056	0.445521	1
Cast	0.016053	0.174055	1
Cask	0.016042	0.384057	1
Plcb1	0.016027	0.187228	1
Ap3s2	0.015976	0.036655	1
Fmc1	0.015918	0.007512	1
Kctd3	0.015893	0.01634	1
Nup35	0.015871	0.003369	1
Bahd1	0.015806	0.119486	1
Zfp598	0.015794	0.004758	1
Sertad3	0.015789	0.010313	1
Ampd2	0.015739	0.040447	1
Chd2	0.01572	0.240072	1
Sertad2	0.015674	0.000772	1
Arhgef6	0.015653	0.042867	1
Tatdn2	0.015535	0.002423	1
Jmjd7	0.015509	0.008919	1
Med15	0.015503	0.063992	1
Ywhae	0.015451	0.255936	1
Mcm2	0.015442	0.052714	1
Atp6v1e1	0.015435	0.246512	1
Surf6	0.015433	0.282166	1
Tbc1d20	0.015335	0.029862	1
Sec23b	0.015281	0.009286	1
Mthfd1	0.015261	0.011918	1
Slc25a26	0.015219	0.007567	1
Fbxo25	0.01519	0.032248	1
Akap11	0.015136	0.032088	1
Atg14	0.015116	0.438368	1
Cplane1	0.015097	0.059206	1
Tyw5	0.015062	0.018977	1
Ndufaf6	0.01504	0.071464	1
Coasy	0.015034	0.042131	1
Nme7	0.015001	0.04489	1
Crem	0.014975	0.090049	1
Zdhhc16	0.014971	0.008746	1
Ube2v2	0.014956	0.217628	1
Asb8	0.01495	0.008184	1
Deaf1	0.014942	0.005459	1
Rars2	0.014916	0.123658	1

Ints7	0.014827	0.106149	1
Inpp5a	0.01481	0.061622	1
Mtf1	0.014724	0.082772	1
Cbll1	0.014702	0.038502	1
Car9	0.014681	0.11889	1
Phactr4	0.014677	0.000842	1
Prcc	0.01466	0.075121	1
Pdpr	0.014644	0.005167	1
Mrpl58	0.014643	0.016529	1
Lancl2	0.014637	0.068832	1
Creb3	0.014574	0.200889	1
Kat5	0.014564	0.931028	1
1810030O	0.014542	0.330239	1
Smyd5	0.014504	0.000241	1
Ell	0.01448	0.022984	1
Hax1	0.014435	0.06695	1
Mtus1	0.014388	0.045843	1
Ddx20	0.01437	0.010905	1
Tax1bp3	0.014334	0.006315	1
Gale	0.014252	0.025248	1
Pdcd6ip	0.014247	0.371166	1
Bmi1	0.014227	0.008918	1
Tex2	0.014213	0.031668	1
Mars	0.014128	0.089552	1
Mif4gd	0.014117	0.051278	1
Mtor	0.014089	0.015092	1
Tti2	0.014081	0.229867	1
Oxld1	0.014078	0.060703	1
Rae1	0.014029	0.276512	1
Uchl5	0.013973	0.010089	1
Atad2	0.013971	0.031414	1
Pigf	0.013915	0.023398	1
Tmem109	0.013879	0.352827	1
Hotairm1	0.013876	0.00547	1
Dapk3	0.013872	0.093943	1
Otud6b	0.013849	0.003194	1
Ttc19	0.013838	0.030987	1
Gm8797	0.013791	0.029731	1
Kxd1	0.013778	0.120181	1
Eef1akmt1	0.013696	0.04448	1
Uqcrh	0.01369	0.990188	1
Elp2	0.013674	0.025647	1
Tex30	0.013624	0.029539	1
Vkorc1l1	0.01357	0.105899	1
Inppl1	0.013533	0.000184	1
Mtx2	0.013403	0.001635	1
Slc1a5	0.013401	0.003651	1
Ccdc43	0.013299	0.266156	1

Pms2	0.013268	0.020042	1
Sec63	0.013265	0.004687	1
Virma	0.013258	0.015701	1
Rnf38	0.013244	0.291323	1
Wdr19	0.01321	0.022488	1
Bnip1	0.013199	0.003609	1
Sfxn5	0.013162	0.010618	1
Ap1ar	0.013141	0.02477	1
Pdk1	0.013138	0.029118	1
Tcn2	0.013103	0.019013	1
Aph1c	0.013102	0.197325	1
Heatr3	0.013077	0.036521	1
Zfpl1	0.013039	0.00408	1
Cxxc1	0.013034	0.277242	1
Map2k4	0.012994	0.074846	1
Cops8	0.012963	0.032284	1
Ppie	0.012933	0.108496	1
Stk25	0.012901	0.038811	1
Tars	0.012853	0.080613	1
Slc25a43	0.012803	0.036099	1
Ctu1	0.012727	0.062985	1
Tmem251	0.012719	0.006425	1
Med16	0.012712	0.07493	1
Iffo2	0.012703	0.165369	1
Psmg3	0.012697	0.123616	1
Hs1bp3	0.012656	0.001951	1
Sptlc1	0.012567	0.001377	1
Pnp2	0.012501	0.007032	1
Ubap2	0.012404	0.147957	1
1110004F1	0.012402	0.449227	1
Rbsn	0.012364	0.033998	1
Mafk	0.012335	0.131965	1
Necap1	0.012259	0.002225	1
Rnf8	0.012236	0.009183	1
Zfp266	0.012191	0.730797	1
Hacd4	0.012015	0.149759	1
Sdhaf3	0.01198	0.00872	1
Alyref2	0.011946	0.045958	1
Dnaaf3	0.011907	0.012793	1
Eral1	0.011879	0.244335	1
Eif2b3	0.011857	0.005046	1
Ccar1	0.01185	0.044132	1
Rfc4	0.011846	0.073589	1
Myl6	0.01171	0.599453	1
Alg3	0.011675	0.050003	1
Srpk2	0.011611	0.070747	1
Ghdc	0.011589	0.028878	1
Med6	0.011546	0.014846	1

Dennd2d	0.011501	0.011677	1
Irf2	0.011478	0.667223	1
Alad	0.01142	0.002431	1
Dync2h1	0.011377	0.053798	1
Armc8	0.011308	0.01566	1
Sephs1	0.011302	0.072284	1
Pus10	0.011281	0.137869	1
Gnpda2	0.011255	0.018511	1
Adrm1	0.011247	0.027624	1
Cand1	0.0112	0.029255	1
Zfp236	0.011198	0.140314	1
Klf7	0.011193	9.66E-05	1
Polrmt	0.01119	0.010968	1
Usp36	0.011156	0.00121	1
2700049A(	0.011149	0.069436	1
Dcps	0.011116	0.094322	1
Eef2kmt	0.011078	0.014747	1
Dhdds	0.011025	0.039835	1
Ppif	0.011023	0.25527	1
Tmx4	0.010903	0.089489	1
Rpusd4	0.010893	0.014151	1
Pcca	0.010841	0.025894	1
Ddx19a	0.010782	0.005496	1
Cdc37l1	0.010776	0.004823	1
Haus7	0.010737	0.020069	1
Sec23ip	0.010718	0.107478	1
Fbxo9	0.01068	0.039033	1
Mrtfb	0.010646	0.497446	1
Coa4	0.010608	0.003087	1
Ccdc71	0.010602	0.071586	1
D16Ertd47	0.010581	0.019313	1
Srpk1	0.010561	0.004961	1
Msh2	0.01055	0.051476	1
Zcchc10	0.010505	0.117127	1
Tor1aip2	0.010493	0.102398	1
2310015A:	0.010475	0.164768	1
Naf1	0.010433	0.031019	1
Zfp451	0.010429	0.150252	1
Mast3	0.010428	0.044846	1
Sfswap	0.010372	0.161309	1
Siah2	0.010359	0.020721	1
Taf6l	0.010356	0.085626	1
Rasal3	0.010349	0.030113	1
Ly9	0.010337	0.062267	1
Hnrnpm	0.01032	0.126966	1
Snx11	0.010239	0.050606	1
Bag4	0.010213	0.136901	1
Gnpat	0.010096	0.328723	1

Mctp2	0.010073	0.031012	1
Ankrd52	0.010039	0.003359	1
Aph1b	0.010011	0.194709	1
Strn4	-0.01006	0.001055	1
Pde8a	-0.01007	0.050173	1
Jpt1	-0.01011	0.915481	1
Zfp932	-0.01013	0.010176	1
Cpd	-0.01017	0.062478	1
Polr2k	-0.01019	0.014656	1
Tbkbp1	-0.01019	1.61E-05	0.520158
Cdk2	-0.01025	0.595488	1
AC154200.	-0.01026	0.333719	1
Exosc7	-0.0103	0.230315	1
Ahsa2	-0.0103	0.500683	1
Tbc1d10b	-0.01034	0.343634	1
Coq9	-0.01036	0.187996	1
Cog2	-0.01039	0.308028	1
Mmgt2	-0.01043	0.454059	1
Thap11	-0.01046	0.106784	1
Cul2	-0.01048	0.045412	1
Ndufb1-ps	-0.01048	0.94187	1
Dop1b	-0.01049	0.024241	1
Cep19	-0.0105	0.599431	1
Sdf2	-0.01062	0.108152	1
Zkscan8	-0.01066	0.09306	1
Tspyl2	-0.01067	0.039969	1
Pstpip1	-0.01079	0.57646	1
Tmem94	-0.01085	0.101498	1
Tk2	-0.01088	0.181692	1
Aldh4a1	-0.01091	0.919499	1
Cyb5a	-0.01098	0.004455	1
Slc25a44	-0.01101	0.102916	1
C9orf72	-0.01101	0.08749	1
Sept10	-0.01103	0.1154	1
Recql	-0.01105	0.00777	1
Cog6	-0.01106	0.440766	1
Actb	-0.01108	0.645018	1
Mrps30	-0.01109	0.1004	1
Glmn	-0.01111	0.003903	1
Zfp280d	-0.01116	0.244525	1
Cpsf4	-0.01117	0.024928	1
Ube2q1	-0.01118	0.452673	1
Ap1s3	-0.01119	0.467093	1
Tbc1d22a	-0.01119	0.036831	1
Polk	-0.01121	0.277075	1
Arl8a	-0.01125	0.051542	1
Yrdc	-0.0113	0.041045	1
Cyren	-0.01135	0.755546	1

F730043M	-0.01139	0.131494	1
Dis3l	-0.0114	0.397529	1
Bcr	-0.01141	0.958582	1
Mbd1	-0.01141	0.107186	1
B9d2	-0.01144	0.477547	1
Mbip	-0.01153	0.05283	1
Zfp90	-0.01154	0.152306	1
Blmh	-0.01157	0.057669	1
Rpl34	-0.01157	0.662546	1
Tmem11	-0.01158	0.00172	1
Arl3	-0.01164	0.583906	1
Nsmce3	-0.01164	0.09106	1
Pam	-0.01165	0.237835	1
Abhd10	-0.01169	0.195693	1
Chp1	-0.01169	0.079902	1
U2af2	-0.01178	0.019924	1
Cdc25a	-0.01179	0.021612	1
Akap9	-0.0118	0.100026	1
Met	-0.01188	0.027871	1
Depdc5	-0.01188	0.170697	1
BC003965	-0.01198	0.192115	1
Apex2	-0.01198	0.478139	1
Erap1	-0.01198	0.06949	1
Hipk3	-0.01201	0.192527	1
Tmem43	-0.01202	0.331936	1
Nup54	-0.01202	0.10669	1
Lcmt2	-0.01208	0.231319	1
Zyg11b	-0.01209	0.948418	1
Cnot8	-0.0121	0.541462	1
Rps24	-0.01213	0.428816	1
Prim2	-0.01217	0.178604	1
Stard3	-0.01218	0.163469	1
Gtf3a	-0.0122	0.150799	1
Ddit3	-0.01223	0.020842	1
Arhgef10	-0.01227	0.555592	1
Smarcd1	-0.01231	0.009212	1
Zbtb33	-0.01238	0.48769	1
Cwf19l1	-0.01244	0.282166	1
Chmp2a	-0.01245	0.939293	1
Gatb	-0.01247	0.019933	1
Pus7l	-0.01254	0.134421	1
Uevld	-0.01257	0.308882	1
Setd7	-0.01258	0.258492	1
Aaas	-0.01263	0.149182	1
Ppat	-0.01265	0.084857	1
Ankrd49	-0.01272	0.199454	1
Capn7	-0.01273	0.058321	1
Esf1	-0.01277	0.018307	1

Lsm8	-0.01283	0.006064	1
1110059G:	-0.01283	0.284037	1
Slc37a3	-0.01287	0.121926	1
Dusp7	-0.01289	0.033602	1
Aggf1	-0.01291	0.066102	1
Pogz	-0.01291	0.282637	1
C87436	-0.01297	0.141003	1
Upf3a	-0.01299	0.067334	1
Tjap1	-0.01311	0.438084	1
Deptor	-0.01313	0.142876	1
Atxn7l3	-0.01315	0.055756	1
Rap2b	-0.01318	0.173594	1
Qser1	-0.01319	0.320449	1
Trim65	-0.01324	0.007733	1
Pdcd10	-0.0133	0.632195	1
Atxn3	-0.01331	0.203823	1
Prpf18	-0.01343	0.32619	1
Det1	-0.01344	0.453254	1
Pilra	-0.01349	0.368042	1
Sumf1	-0.0135	0.094157	1
Slfn9	-0.01354	0.07326	1
Zfp53	-0.01354	0.042117	1
Kcmf1	-0.01355	0.054859	1
Srrt	-0.01366	0.15213	1
Tfpt	-0.01366	0.011603	1
Ddx41	-0.01366	0.096062	1
Yars2	-0.01368	0.059969	1
Ube2z	-0.01375	0.053495	1
Tnfaip1	-0.01376	0.113112	1
Zfp386	-0.0138	0.187515	1
Il15	-0.01381	0.045122	1
Prrc2b	-0.01384	0.850628	1
Ddx52	-0.01386	0.086008	1
Map2k6	-0.01388	0.081239	1
Emc8	-0.01392	0.09392	1
Pigp	-0.01392	0.198167	1
Mib2	-0.01392	0.346606	1
Wdsub1	-0.01393	0.182508	1
Alox5ap	-0.01397	0.91568	1
Gtf2h2	-0.01399	0.127013	1
Alkbh8	-0.01403	0.17551	1
Taf8	-0.01405	0.083042	1
Pacc1	-0.01408	0.47296	1
Bclaf1	-0.01412	0.146881	1
B4gat1	-0.01413	0.415011	1
Mad2l1bp	-0.01413	0.092796	1
Actl6a	-0.01413	0.056316	1
Isca2	-0.01414	0.052601	1

Dnajc10	-0.01416	0.03365	1
Mrpl46	-0.01419	0.092267	1
Casp7	-0.01421	0.030351	1
Parp10	-0.01423	0.072993	1
Golph3l	-0.01426	0.186878	1
Pex11b	-0.0143	0.060147	1
Rnf135	-0.01431	0.072391	1
Mfsd5	-0.01431	0.285039	1
Plekha2	-0.01434	0.138808	1
Spata2	-0.01437	0.231765	1
Skiv2l	-0.01437	0.275169	1
Foxj2	-0.01437	0.031164	1
Mrps35	-0.01437	0.119744	1
Mrpl34	-0.01444	0.091195	1
Acsf3	-0.01452	0.553304	1
Cdca4	-0.01467	0.050076	1
Chmp5	-0.01478	0.33462	1
Med31	-0.01479	0.566505	1
Klhl24	-0.0148	0.31648	1
Eif2d	-0.01485	0.055464	1
Thada	-0.01486	0.023355	1
Ppm1g	-0.01489	0.159888	1
Fbxw5	-0.01497	0.135977	1
Klhl7	-0.01503	0.549696	1
Tmem41b	-0.01506	0.010854	1
Zmat3	-0.01507	0.032445	1
Zfp830	-0.01508	0.254459	1
Runx3	-0.0151	0.16308	1
Ahcyl1	-0.01512	0.000621	1
Pex14	-0.01514	0.160805	1
Dcun1d3	-0.01517	0.085038	1
Aar2	-0.01519	0.041675	1
Rrp1b	-0.01525	0.072617	1
Rnf214	-0.01527	0.152222	1
Zfp692	-0.01531	0.630458	1
Pik3ca	-0.01538	0.070367	1
Dop1a	-0.0154	0.027074	1
Drosha	-0.01542	0.38702	1
Erc1	-0.01546	0.098975	1
Ctnnbip1	-0.01552	0.148595	1
Copb2	-0.01554	0.068148	1
Yeats2	-0.01555	0.034261	1
Ttc4	-0.01559	0.071868	1
1810037I1	-0.01561	0.07083	1
Brk1	-0.01564	0.386856	1
Spag7	-0.01568	0.32422	1
Rnf13	-0.01572	0.784803	1
Gdi2	-0.01575	0.695589	1

Hsh2d	-0.01576	0.815963	1
Gpn3	-0.01584	0.24766	1
Las1l	-0.01592	0.311733	1
Rhot1	-0.01598	0.204023	1
Lrrfip2	-0.01601	0.506332	1
Casd1	-0.01606	0.25537	1
Alg12	-0.01609	0.387801	1
Jmjd6	-0.01611	0.196385	1
Was	-0.01613	0.806143	1
Hjurp	-0.01615	0.285647	1
Idua	-0.01617	0.017058	1
Slc7a6os	-0.01618	0.354192	1
Lrp6	-0.01624	0.174179	1
Coq8b	-0.01626	0.291351	1
Oas1c	-0.01635	0.17378	1
Pde6d	-0.01636	0.524844	1
Tbccd1	-0.01637	0.056795	1
Polr2g	-0.01638	0.379655	1
Zfp639	-0.01645	0.011697	1
Sp140	-0.01648	0.429989	1
Nxt1	-0.01652	0.902372	1
Ing5	-0.01652	0.048573	1
Casp8	-0.01658	0.290336	1
Commd8	-0.01662	0.109847	1
Cmas	-0.01667	0.177934	1
Yipf6	-0.01668	0.027063	1
Hp	-0.01669	0.220066	1
Tet3	-0.01672	0.119665	1
St3gal5	-0.01675	0.135926	1
Exoc7	-0.01684	0.008368	1
Acot2	-0.01684	0.375494	1
Coq10b	-0.01685	0.092987	1
Clp1	-0.01686	0.496929	1
Ddx27	-0.01693	0.033356	1
Glb1l	-0.01694	0.786015	1
Afmid	-0.01697	0.077322	1
Dmac2	-0.01703	0.544477	1
Mcur1	-0.01709	0.123078	1
Hdhd5	-0.0171	0.579466	1
Ddx55	-0.01713	0.182021	1
Trpv2	-0.01726	0.901539	1
Adar	-0.01737	0.247207	1
D2hgdh	-0.01742	0.626169	1
Rufy3	-0.01742	0.022424	1
Sars	-0.01744	0.253252	1
Serpinb9	-0.01746	0.073416	1
Ddx24	-0.01746	0.743218	1
Mboat7	-0.01751	0.132846	1

Zfp516	-0.01757	0.101916	1
Stk11ip	-0.01759	0.523816	1
Ankrd16	-0.0176	0.638941	1
Epb41l4ao:	-0.01767	0.057919	1
Mapre1	-0.01767	0.070343	1
Lrrc41	-0.01773	0.015893	1
Nedd1	-0.01778	0.022514	1
Sec22b	-0.01786	0.076497	1
Rbmx2	-0.01788	0.664836	1
Flii	-0.01791	0.226144	1
Cnpy4	-0.01792	0.050151	1
Gpd2	-0.01793	0.10202	1
2410004B:	-0.018	0.061419	1
Gtf3c6	-0.01801	0.238624	1
Prkacb	-0.01804	0.471006	1
Myg1	-0.0181	0.019793	1
Ccdc117	-0.0181	0.4798	1
Jund	-0.01818	0.990417	1
Tdp2	-0.01819	0.27385	1
Phf23	-0.01821	0.094555	1
Fkbp3	-0.01825	0.142304	1
Pias2	-0.0184	0.167037	1
Fkbpl	-0.01842	0.798567	1
Dnase1l1	-0.01844	0.247973	1
Oxsm	-0.01845	0.100838	1
Endod1	-0.01845	0.541772	1
Ropn1l	-0.01847	0.104346	1
Fnbp4	-0.0185	0.813495	1
Shisa5	-0.01852	0.719082	1
Emc10	-0.01856	0.051759	1
Mterf3	-0.01862	0.221233	1
Crkl	-0.01872	0.135298	1
Pdik1l	-0.01876	0.310457	1
Slc22a5	-0.01877	0.291928	1
Ice1	-0.01879	0.276696	1
Fas	-0.01884	0.03542	1
Dhcr7	-0.01885	0.583162	1
Brcc3	-0.01886	0.022046	1
Rbm34	-0.0189	0.059902	1
Pfn1	-0.01894	0.855772	1
Ankrd39	-0.01902	0.084237	1
Nckipsd	-0.01907	0.220264	1
Ptcd1	-0.01909	0.492366	1
Gm8909	-0.01912	0.190006	1
Zfp617	-0.01913	0.457749	1
Smarcal1	-0.01936	0.135292	1
Cars2	-0.01938	0.001309	1
Zfp729b	-0.0195	0.098748	1

Tnip2	-0.01955	0.012975	1
Atf6b	-0.01957	0.350802	1
Ppp2r3c	-0.01958	0.301051	1
S100a13	-0.01962	0.717978	1
Tmem115	-0.01963	0.416366	1
Smpd1	-0.01976	0.176638	1
1700025G(	-0.01983	0.322187	1
Gmfg	-0.01995	0.864209	1
Srsf7	-0.02005	0.357241	1
Ufd1	-0.0201	0.015328	1
Cwc15	-0.02027	0.347045	1
Txnrd2	-0.02027	0.043366	1
Ano10	-0.02027	0.507185	1
Pja1	-0.02034	0.691915	1
Fdxacb1	-0.02047	0.074547	1
Rbm19	-0.02057	0.115188	1
Carnmt1	-0.02057	0.562964	1
Gm26917	-0.0206	0.530292	1
Kctd12	-0.02064	0.246261	1
Ice2	-0.02068	0.841716	1
Supt7l	-0.0207	0.084584	1
Brd3	-0.02071	0.08328	1
Gtf2h1	-0.02073	0.070488	1
Ulk1	-0.02075	0.766074	1
Tns4	-0.02076	0.383354	1
Cdk7	-0.02082	0.054697	1
Myo9b	-0.02094	0.273551	1
Arl15	-0.02099	0.073915	1
Tamm41	-0.02103	0.215224	1
Ptrhd1	-0.02103	0.253424	1
Gosr1	-0.02103	0.124281	1
Acap3	-0.02103	0.322747	1
Sfr1	-0.02106	0.664072	1
Slc25a37	-0.02107	0.005654	1
Zfp668	-0.0211	0.07669	1
Plrg1	-0.02112	0.13575	1
Idh2	-0.02115	0.000955	1
Sbf1	-0.02135	0.188672	1
Zfp868	-0.02135	0.091124	1
2810402E2	-0.02136	0.439585	1
Babam2	-0.02142	0.570131	1
Trmt5	-0.02143	0.060252	1
Tcta	-0.02147	0.260625	1
Slf1	-0.02147	0.011307	1
Fam193a	-0.0215	0.097691	1
Mettl5	-0.02153	0.239914	1
Kbtbd3	-0.02155	0.106239	1
Mmgt1	-0.02164	0.29502	1

Oser1	-0.02171	0.180849	1
Klc1	-0.02174	0.194636	1
Dffa	-0.02175	0.382908	1
Ormdl3	-0.02175	0.738295	1
Mthfsd	-0.02175	0.127842	1
Wdr24	-0.0218	0.217198	1
Ppp4r1	-0.02181	0.059803	1
Hist2h2ac	-0.02187	0.425678	1
Unk	-0.02197	0.253911	1
Neurl3	-0.02206	0.383893	1
Pias3	-0.02206	0.204615	1
Isoc2b	-0.02208	0.612221	1
Senp5	-0.02214	0.047104	1
Orc5	-0.0222	0.106925	1
H2-Q4	-0.02226	0.860714	1
Il21r	-0.02226	0.045554	1
Togaram1	-0.0223	0.005781	1
Topors	-0.02235	0.161971	1
Cep250	-0.02239	0.246786	1
Zfp518a	-0.02241	0.025035	1
Arl13b	-0.02245	0.454159	1
Mospd3	-0.02245	0.288698	1
Enoph1	-0.02255	0.155375	1
Slc11a1	-0.02257	0.244513	1
Zfp330	-0.0226	0.020513	1
Mocos	-0.02266	0.371667	1
Uty	-0.02267	0.295049	1
Cutc	-0.02272	0.679491	1
Rnaset2b	-0.02272	0.602632	1
Mrpl24	-0.02276	0.257071	1
Polr3k	-0.02278	0.085515	1
Scly	-0.02281	0.000219	1
Memo1	-0.02287	0.002505	1
Cipc	-0.02293	0.439969	1
Agap2	-0.023	0.510562	1
Gm14548	-0.02306	0.048472	1
Mettl25	-0.02312	0.272952	1
Pxmp4	-0.02313	0.233856	1
Zfp160	-0.02316	0.419351	1
Smim15	-0.02319	0.204094	1
Pick1	-0.0232	0.386034	1
Zfp943	-0.0232	0.161923	1
Atg2a	-0.0232	0.44586	1
Rab37	-0.02327	0.191696	1
Nadk2	-0.02329	0.867926	1
Zfp653	-0.02342	0.23388	1
Ppp6r2	-0.02342	0.82726	1
Fto	-0.02343	0.023665	1

Mettl17	-0.02348	0.254847	1
Pdzd11	-0.02351	0.108193	1
Itpripl2	-0.02352	0.016191	1
Matk	-0.02357	0.283173	1
Ppa2	-0.0236	0.228685	1
Axin1	-0.02363	0.456561	1
BC023719	-0.02364	0.091908	1
Ncapd3	-0.02364	0.012048	1
Ube2f	-0.02366	0.904697	1
Cep57	-0.02377	0.094126	1
Cep104	-0.02378	0.005988	1
Bicd2	-0.02378	0.126155	1
Pja2	-0.0238	0.679957	1
Stxbp2	-0.02382	0.37086	1
Nr1h3	-0.02388	0.744118	1
Kctd9	-0.0239	0.388975	1
Gm39469	-0.02392	0.498122	1
Ccdc88b	-0.02393	0.780668	1
Acbd3	-0.02394	0.231239	1
Ercc4	-0.02398	0.22189	1
Zfp512	-0.02402	0.046882	1
Padi4	-0.02403	0.743366	1
Tnpo2	-0.02405	0.400284	1
Smg5	-0.02412	0.124588	1
2810403D:	-0.02412	0.944754	1
Crls1	-0.02423	0.052522	1
Dolk	-0.02425	0.502706	1
Hmox1	-0.02429	0.001304	1
Slc35a1	-0.02433	0.364785	1
Surf1	-0.0244	0.059825	1
Zfp429	-0.02449	0.279609	1
Fig4	-0.02468	0.09835	1
Hmgxb4	-0.02469	0.341064	1
Arel1	-0.02469	0.063029	1
Agtpbp1	-0.02477	0.003345	1
Znrf1	-0.02481	0.096013	1
Fut11	-0.02484	0.027741	1
Ly6e	-0.02491	0.25116	1
Rnf113a2	-0.02494	0.753636	1
Trim37	-0.02498	0.902982	1
Arhgap23	-0.02503	0.441899	1
Larp4	-0.02508	0.211822	1
Tor4a	-0.02508	0.976193	1
Brms1l	-0.02517	0.969999	1
Aldh18a1	-0.02521	0.395419	1
Tmem259	-0.02523	0.227592	1
Gapt	-0.02544	0.545045	1
Cpox	-0.02544	0.085711	1

Ctps2	-0.02544	0.194988	1
Usp16	-0.02546	0.004921	1
Selenoo	-0.02549	0.358071	1
Ints10	-0.0255	0.127532	1
Dus3l	-0.02553	0.471359	1
Ikzf5	-0.02556	0.166012	1
Strip1	-0.02557	0.11162	1
Zscan26	-0.02564	0.333517	1
Erlin2	-0.02567	0.131264	1
Pgpep1	-0.02568	0.06753	1
Patl1	-0.02573	0.193279	1
Ccdc77	-0.02576	0.100464	1
2610507I0	-0.02576	0.997677	1
Elk3	-0.02584	0.063334	1
Clpb	-0.02585	0.274941	1
Scd2	-0.02587	0.15987	1
Gm49336	-0.0259	0.120646	1
Polr3f	-0.02593	0.458206	1
Vamp4	-0.02593	0.058856	1
Cox18	-0.02604	0.556306	1
Rev3l	-0.02608	0.148778	1
Polr2j	-0.02608	0.225756	1
Ddi2	-0.02608	0.21332	1
Trmt44	-0.02613	0.216386	1
Fbxo8	-0.02617	0.092863	1
Slc25a24	-0.02618	0.285205	1
Abhd18	-0.02626	0.436342	1
Dock6	-0.02629	0.066178	1
Rhot2	-0.02631	0.634082	1
Cops3	-0.02639	0.272701	1
Dhodh	-0.02639	0.139308	1
Crnkl1	-0.0264	0.425822	1
5830432E(	-0.02641	0.851957	1
Phpt1	-0.02647	0.753572	1
Ankrd54	-0.02648	0.501775	1
Fra10ac1	-0.02652	0.038128	1
Rnf146	-0.02652	0.089521	1
Gan	-0.02654	0.382537	1
Sec31a	-0.02654	0.212613	1
Ppp2r1b	-0.02655	0.222766	1
Cenpc1	-0.02659	0.135828	1
Rab11a	-0.02661	0.327483	1
Rpap1	-0.02663	0.236932	1
Zfp91	-0.02665	0.053611	1
Ggh	-0.02666	0.669459	1
Zbtb45	-0.02671	0.368892	1
Orc2	-0.02675	0.222142	1
Trrap	-0.02675	0.075887	1

Ubxn8	-0.02676	0.131552	1
Prkaca	-0.02682	0.167023	1
2210016F1	-0.02691	0.076055	1
Mtmr3	-0.02692	0.245112	1
Vps51	-0.02694	0.045077	1
Ttll4	-0.02696	0.374535	1
Igbp1	-0.02703	0.334195	1
5730455P1	-0.0271	0.570517	1
Ppan	-0.02711	0.49711	1
Dcun1d2	-0.02711	0.101708	1
Akt1s1	-0.02711	0.068872	1
Pcmtd2	-0.02722	0.547952	1
1700084C0	-0.02726	0.416587	1
Zfand4	-0.0273	0.185882	1
Sec61g	-0.02734	0.464558	1
Brf2	-0.02738	0.009304	1
Clpx	-0.02743	0.014537	1
Rttn	-0.02745	0.662723	1
Phrf1	-0.02748	0.030223	1
Psph	-0.02751	0.501215	1
Noc3l	-0.02752	0.00719	1
Tm2d1	-0.02752	0.539368	1
Mak16	-0.02755	0.090403	1
Gm31597	-0.02756	0.124702	1
Ppp2r2d	-0.02756	0.027227	1
Phtf1	-0.02758	0.120431	1
Dcp1b	-0.02759	0.223712	1
Mgat1	-0.02766	0.159071	1
Gcn1	-0.02767	0.211683	1
Dnajc30	-0.02769	0.024635	1
Nars	-0.0277	0.221058	1
Spcs3	-0.02775	0.49722	1
Ccdc130	-0.02776	0.24103	1
Scyl2	-0.02779	0.016127	1
Ndrg3	-0.02779	0.248359	1
Zdhhc9	-0.02779	0.211638	1
Lyrm2	-0.02781	0.405225	1
Ptar1	-0.02782	0.475936	1
Stub1	-0.02783	0.301402	1
Actr5	-0.02785	0.371162	1
Ttc38	-0.02787	0.370336	1
Gmfb	-0.02788	0.222821	1
Ptdss1	-0.02801	0.18402	1
Wdr46	-0.02803	0.017011	1
Usp21	-0.02805	0.23305	1
Gm36738	-0.02805	0.358741	1
Med24	-0.02808	0.159713	1
Itpr3	-0.02822	0.10249	1

Nudt22	-0.02822	0.554256	1
Trem1	-0.02823	0.382192	1
Topbp1	-0.02823	0.200411	1
Trappc11	-0.02829	0.089955	1
Pank3	-0.02829	0.076434	1
Apip	-0.0283	0.232189	1
Scmh1	-0.02835	0.38412	1
Nmral1	-0.02836	0.451036	1
Edrf1	-0.02843	0.886892	1
Ndufaf1	-0.02844	0.817573	1
Usp39	-0.02851	0.116237	1
Odr4	-0.02853	0.194098	1
Surf2	-0.02856	0.09982	1
Cops7b	-0.02856	0.185564	1
Prpsap1	-0.02858	0.174067	1
Parp3	-0.02863	0.121649	1
Higd2a	-0.02863	0.588865	1
Bag5	-0.02865	0.518773	1
Mga	-0.02865	0.025073	1
Mex3c	-0.02866	0.185467	1
AU040320	-0.02868	0.181628	1
Fem1a	-0.02875	0.750872	1
Gclc	-0.02887	0.041351	1
Daxx	-0.02891	0.232554	1
Med8	-0.02893	0.051678	1
Rinl	-0.02897	0.978643	1
Sap30bp	-0.02899	0.086511	1
Dnm2	-0.02904	0.684016	1
Crebzf	-0.02907	0.249213	1
Pde12	-0.0291	0.478649	1
Smad5	-0.0292	0.862142	1
Zfp655	-0.02921	0.067953	1
Wdyhv1	-0.02927	0.166815	1
Gne	-0.02927	0.912984	1
Mrfap1	-0.02936	0.806434	1
Stard4	-0.0294	0.275583	1
Phf6	-0.02948	0.689636	1
Tcp11l2	-0.02951	0.894242	1
Slamf9	-0.02962	0.036688	1
Mettl26	-0.02962	0.847564	1
Atg16l1	-0.02974	0.027799	1
Micu3	-0.02974	0.449149	1
Zdhhc18	-0.02982	0.005578	1
Emc1	-0.02987	0.121198	1
Psmf1	-0.02988	0.323431	1
Rnf114	-0.02992	0.904021	1
Mtmr10	-0.02997	0.841885	1
Fbxo46	-0.03007	0.813834	1

Lsm14b	-0.0302	0.564582	1
Snx12	-0.0303	0.478205	1
Rnf4	-0.03031	0.585544	1
Taf1b	-0.03039	0.779861	1
Hist1h2bc	-0.03062	0.051948	1
Ogdh	-0.03068	0.049633	1
Gnpda1	-0.03069	0.033895	1
Mitd1	-0.03071	0.092555	1
Pcmt1	-0.03071	0.373467	1
Nfyb	-0.03074	0.344514	1
Prorsd1	-0.03078	0.123963	1
Mrpl19	-0.03087	0.026679	1
Adnp2	-0.0309	0.082566	1
Dnajc24	-0.03091	0.379015	1
Wipf1	-0.03091	0.752894	1
Pds5a	-0.03092	0.036943	1
Arfip1	-0.031	0.032314	1
Avpi1	-0.031	0.101185	1
Dctn6	-0.03102	0.529791	1
Rab3il1	-0.03114	0.062753	1
Cetn2	-0.03116	0.050419	1
Gemin5	-0.03124	0.309854	1
Dus2	-0.03135	0.193685	1
Acsl4	-0.03136	0.119976	1
Zfp738	-0.03139	0.108411	1
Acox3	-0.0314	0.659317	1
1600020E(	-0.03141	0.065376	1
Pstk	-0.03147	0.817894	1
Wrap53	-0.03148	0.791307	1
Qtrt1	-0.03148	0.590022	1
Bap1	-0.03148	0.560247	1
Rtel1	-0.03156	0.307405	1
Arpp19	-0.03156	0.512116	1
Mon1b	-0.03157	0.370187	1
Ddx60	-0.03167	0.066283	1
Asb13	-0.03174	0.738589	1
Lsm1	-0.03176	0.020903	1
Zfp687	-0.03178	0.892043	1
Ap4e1	-0.03178	0.076329	1
Wdr7	-0.0318	0.14099	1
Paf1	-0.0319	0.263199	1
Cds2	-0.03215	0.33266	1
Borcs5	-0.03218	0.09585	1
Lbr	-0.03221	0.567045	1
Ocel1	-0.03223	0.05151	1
Taf6	-0.03241	0.105636	1
Zbtb9	-0.03242	0.155007	1
Aplp2	-0.03244	0.970967	1

Anks1	-0.03245	0.040845	1
Pou2f1	-0.03248	0.201767	1
Fzr1	-0.03249	0.139876	1
Slc35a2	-0.03255	0.594337	1
Osgep	-0.03255	0.316608	1
Etv3	-0.03257	0.198789	1
Mrpl57	-0.03263	0.095781	1
Rangap1	-0.03265	0.043183	1
Anapc7	-0.03275	0.033473	1
Rpp30	-0.03278	0.301529	1
Zswim6	-0.03289	0.08788	1
Zfp688	-0.03289	0.179379	1
Cln5	-0.03309	0.048855	1
Daam1	-0.0331	0.412924	1
Accs	-0.03313	0.457203	1
Manea	-0.03319	0.8345	1
Psmg1	-0.03319	0.152044	1
Rab1a	-0.03329	0.282863	1
Mettl2	-0.03335	0.833361	1
Sgpl1	-0.0334	0.002214	1
Mpzl3	-0.03342	0.575152	1
Nt5c3	-0.03344	0.231394	1
Csrp2	-0.03348	0.317576	1
Ercc6	-0.03354	0.493041	1
Snrpb2	-0.03355	0.009672	1
Cnp	-0.03355	0.043876	1
Helz	-0.0336	0.036387	1
Dgcr2	-0.03362	0.184798	1
Fbxw8	-0.03364	0.019881	1
3110056K(	-0.03367	0.60094	1
Sucla2	-0.03375	0.239412	1
Elk4	-0.03376	0.319141	1
Gt(ROSA)26	-0.03376	0.771333	1
Zfp709	-0.03377	0.254002	1
Rpl7	-0.03377	0.389673	1
Sergef	-0.03378	0.770586	1
Stk38l	-0.03382	0.864797	1
Cul5	-0.03388	0.630791	1
Hinfp	-0.03391	0.567562	1
Polr2c	-0.03394	0.584345	1
Fam222b	-0.03397	0.186784	1
Lactb2	-0.034	0.269048	1
Mcrip1	-0.03402	0.578962	1
Pdrg1	-0.03406	0.6971	1
Ppig	-0.03411	0.047399	1
Fam118b	-0.03419	0.441889	1
Pspc1	-0.03419	0.171344	1
Mbd6	-0.03423	0.107058	1

Gm36486	-0.03426	0.195006	1
Fibp	-0.03428	0.231053	1
Ctbp2	-0.03428	0.064836	1
Npat	-0.0343	0.988567	1
Sirt3	-0.03437	0.338003	1
Klhl22	-0.03438	0.018408	1
Rdh5	-0.03443	0.341655	1
Rai1	-0.03448	0.795562	1
Borcs8	-0.03452	0.134091	1
2010315B(	-0.03454	0.351105	1
Rbm22	-0.03457	0.468288	1
Klhl26	-0.03457	0.321048	1
Zkscan1	-0.03457	0.300178	1
Rfk	-0.03458	0.541203	1
Uvssa	-0.03464	0.099168	1
Hbs1l	-0.03466	0.441111	1
Rhno1	-0.03467	0.799422	1
Ppp2r5d	-0.03478	0.299169	1
Gm10076	-0.0348	0.05646	1
Thoc7	-0.03489	0.020873	1
Aldh3b1	-0.03492	0.422586	1
Fbxw4	-0.03493	0.756108	1
Ipo11	-0.03494	0.723669	1
Tbp	-0.03499	0.255067	1
Cnot11	-0.03505	0.043192	1
Trim41	-0.0351	0.18078	1
Zfp260	-0.03512	0.645233	1
Zfp35	-0.03517	0.850288	1
Zfp865	-0.03522	0.16339	1
Ranbp9	-0.03523	0.306111	1
Slc2a9	-0.03524	0.088161	1
Bclaf3	-0.03534	0.037856	1
Gak	-0.03536	0.1104	1
Pon2	-0.03537	0.727005	1
Fhod1	-0.03544	0.57138	1
Cep290	-0.03549	0.130412	1
Golga3	-0.0355	0.382663	1
Dubr	-0.03555	0.022813	1
Cmtr1	-0.03556	0.104487	1
Sptlc2	-0.03557	0.537819	1
Zfp574	-0.03559	0.442048	1
Mlxip	-0.03559	0.173188	1
Abl1	-0.0356	0.932515	1
Pigb	-0.03562	0.23287	1
Rnf41	-0.03563	0.251497	1
Tent4b	-0.03581	0.026601	1
Ap1b1	-0.03591	0.169923	1
Braf	-0.03593	0.033884	1

Uhmk1	-0.03593	0.054242	1
G430095P1	-0.03601	0.1676	1
Etfa	-0.03605	0.112482	1
Cdk2ap1	-0.03607	0.395158	1
Faap24	-0.03613	0.139588	1
Frmd4a	-0.03615	0.6078	1
Map7d1	-0.03617	0.220939	1
Xaf1	-0.03617	0.40462	1
Grsf1	-0.0362	0.060185	1
Galnt9	-0.03625	0.592625	1
Cks2	-0.03632	0.368593	1
Srp14	-0.03635	0.514277	1
Taf15	-0.03636	0.222213	1
Cant1	-0.03642	0.02102	1
Pip4k2b	-0.03646	0.042861	1
Ncdn	-0.03651	0.282898	1
Dpf2	-0.03651	0.854002	1
Map11	-0.03652	0.019428	1
Tbc1d22b	-0.03654	0.204137	1
Ufl1	-0.03655	0.114547	1
Kansl1l	-0.03658	0.120855	1
Arhgap1	-0.03661	0.401552	1
Rpap2	-0.03662	0.128805	1
Arl6ip1	-0.03664	0.607649	1
Larp4b	-0.03664	0.307219	1
Tufm	-0.03667	0.052689	1
Cyhr1	-0.03668	0.196788	1
Trappc12	-0.03669	0.302702	1
Plscr3	-0.03672	0.318585	1
Arfip2	-0.03673	0.037799	1
Eif5	-0.03678	0.360721	1
Ppp1r13b	-0.03679	0.744833	1
Mindy3	-0.03686	0.072861	1
Yipf5	-0.03695	0.035981	1
Tbc1d17	-0.03696	0.263245	1
Orc3	-0.03696	0.647587	1
Trmt12	-0.037	0.622085	1
Ero1lb	-0.03709	0.177025	1
Ttc32	-0.0371	0.259368	1
Ints13	-0.03713	0.34959	1
Usp2	-0.03721	0.60915	1
Phkg2	-0.03721	0.566079	1
Setd5	-0.03724	0.737316	1
Pggt1b	-0.03725	0.760723	1
Gle1	-0.03732	0.618159	1
Mettl23	-0.03735	0.38498	1
Vps4a	-0.03737	0.508675	1
Camsap2	-0.03742	0.023338	1

Tm7sf3	-0.03745	0.158092	1
Dync1li2	-0.03745	0.287527	1
Pex1	-0.03746	0.10313	1
Jak2	-0.0375	0.347294	1
Rpp25l	-0.03751	0.096792	1
Ramac	-0.03753	0.327067	1
Dnttip1	-0.03755	0.013346	1
Synrg	-0.03759	0.589546	1
Trpm7	-0.03761	0.102923	1
Hprt	-0.03771	0.228668	1
Klhl36	-0.03785	0.255609	1
Pik3c2a	-0.03793	0.100106	1
Prrg2	-0.03794	0.102926	1
Zfp146	-0.03799	0.955583	1
Irf8	-0.03801	0.298342	1
Synj2bp	-0.03807	0.063561	1
Msantd3	-0.03813	0.221458	1
Rbl2	-0.03814	0.910868	1
Immp1l	-0.03816	0.10702	1
Apmap	-0.03817	0.017688	1
Arpc3	-0.03818	0.98947	1
Elovl6	-0.03821	0.721326	1
Haus5	-0.03823	0.151954	1
Sil1	-0.03828	0.025852	1
Zbtb34	-0.03831	0.625308	1
Prdm4	-0.03831	0.4127	1
Ftx	-0.03833	0.015329	1
Ube2g2	-0.03839	0.377313	1
Dpm1	-0.03845	0.660129	1
Ptpa	-0.03862	0.463569	1
Ctse	-0.03863	0.700583	1
Wdr45b	-0.03868	0.525555	1
Atxn1l	-0.03869	0.647631	1
Ric8a	-0.03873	0.376201	1
Arl5a	-0.03873	0.05047	1
Dtd2	-0.03874	0.097814	1
Qrich1	-0.03881	0.070482	1
Srf	-0.03881	0.452882	1
Trim33	-0.03882	0.118736	1
Bcas3	-0.03883	0.128948	1
Bcap31	-0.03884	0.707269	1
Trmt10c	-0.039	0.326769	1
Dnajc17	-0.03902	0.31586	1
Rnaseh2c	-0.03912	0.446007	1
Zfp282	-0.03915	0.040131	1
Clk2	-0.03933	0.283408	1
Hexa	-0.03941	0.309259	1
Serinc1	-0.03941	0.02619	1

Rbm14	-0.03945	0.270304	1
Lats1	-0.03948	0.434552	1
Sgcb	-0.03953	0.286434	1
Prr3	-0.03958	0.063822	1
Oma1	-0.03959	0.498363	1
Tmub2	-0.0396	0.710004	1
Rtn4ip1	-0.03961	0.42193	1
Slc38a10	-0.03962	0.164957	1
Ighmbp2	-0.03964	0.71174	1
Vipas39	-0.03966	0.015099	1
Pigyl	-0.03968	0.375002	1
Tmem209	-0.03973	0.063405	1
Eif2s3y	-0.03973	0.063921	1
Cdkn2aipnl	-0.03976	0.456548	1
Tmed7	-0.03978	0.53619	1
Hadhb	-0.03982	0.081019	1
Fdps	-0.03984	0.10623	1
Vps37c	-0.03987	0.573626	1
Nkap	-0.03987	0.496487	1
Rif1	-0.03992	0.07233	1
Fam20b	-0.03998	0.639224	1
Mkrn2	-0.04004	0.673203	1
Med12	-0.04005	0.033379	1
Slc30a5	-0.04013	0.039717	1
Zcchc4	-0.04018	0.302888	1
Sumo2	-0.0402	0.968611	1
Cep85	-0.04027	0.207658	1
2510002D:	-0.04028	0.746995	1
Zfp326	-0.04029	0.157468	1
Man2c1	-0.04029	0.347671	1
Wdr81	-0.04032	0.283541	1
Edc4	-0.04038	0.228014	1
Wbp1	-0.04038	0.098554	1
Snapin	-0.04038	0.030201	1
Pbrm1	-0.04041	0.748481	1
Cog1	-0.04055	0.089399	1
Rtn3	-0.04057	0.211577	1
Slc25a1	-0.04058	0.079821	1
Dctn4	-0.04058	0.124691	1
Stambp	-0.04059	0.131233	1
Tbcc	-0.04063	0.746717	1
Kbtbd11	-0.04072	0.929755	1
Krr1	-0.04082	0.555185	1
Taf2	-0.04084	0.168159	1
Zfp385a	-0.04085	0.087425	1
Irak2	-0.0409	0.158204	1
Thoc1	-0.04093	0.054062	1
Hcfc1	-0.04098	0.235124	1

Dyrk2	-0.04102	0.18869	1
Asb7	-0.04109	0.346356	1
Nsun4	-0.04111	0.813292	1
Stimate	-0.04111	0.311334	1
Xpo5	-0.04116	0.173947	1
Ank	-0.04116	0.94235	1
Zbtb6	-0.04119	0.574841	1
Rap2a	-0.0412	0.204829	1
Dnajb1	-0.04122	0.287831	1
Slc33a1	-0.04126	0.321118	1
Man2a1	-0.04132	0.054568	1
Fam241a	-0.04151	0.136671	1
Slc35b4	-0.04152	0.209848	1
Ndfip2	-0.04152	0.263005	1
Uvrag	-0.04155	0.534246	1
S1pr2	-0.04158	0.050374	1
Ing3	-0.04167	0.095269	1
Gm11944	-0.04173	0.900525	1
Zfp422	-0.04189	0.960962	1
Snhg20	-0.04193	0.406006	1
Cfdp1	-0.04195	0.070599	1
Thrap3	-0.04196	0.836182	1
Pdzd8	-0.04198	0.180047	1
Fpr1	-0.04202	0.009959	1
Ints9	-0.04203	0.74526	1
Stk16	-0.04219	0.043026	1
Ndufs1	-0.04222	0.11129	1
Rnf138	-0.04223	0.114238	1
Fam210a	-0.04224	0.37844	1
Wdfy2	-0.04225	0.438497	1
Zbtb43	-0.04239	0.134571	1
Zfp983	-0.04239	0.783661	1
Nagpa	-0.0424	0.173025	1
Gpn2	-0.04241	0.045796	1
Ap1g2	-0.04244	0.279832	1
Miip	-0.04246	0.362442	1
Gpr89	-0.04252	0.392252	1
Prxl2c	-0.04254	0.053733	1
Smu1	-0.04258	0.221802	1
Usp4	-0.0426	0.60873	1
Dr1	-0.0426	0.224065	1
Rrn3	-0.04262	0.956921	1
Zfp212	-0.04263	0.156484	1
Trappc13	-0.04265	0.471734	1
Rab4b	-0.04267	0.209231	1
Sun2	-0.04268	0.005232	1
Zfp346	-0.04269	0.292149	1
Rad9a	-0.04269	0.638253	1

Brd9	-0.0427	0.284426	1
Tmem128	-0.04271	0.271804	1
Ppil4	-0.04275	0.320929	1
Mtrex	-0.04279	0.138546	1
Gm13012	-0.0428	0.505736	1
Emsy	-0.04285	0.525592	1
Pcyox1l	-0.04287	0.002567	1
Pde4a	-0.04287	0.464596	1
Atp9b	-0.04289	0.332014	1
CAAA0111{	-0.0429	0.246501	1
Faap20	-0.04296	0.395841	1
Wtap	-0.04303	0.182338	1
Armcx3	-0.04304	0.861579	1
Srek1ip1	-0.04314	0.028658	1
Suclg2	-0.04315	0.157027	1
Zfp148	-0.04315	0.92682	1
Ap3m1	-0.04316	0.241929	1
Tle3	-0.04317	0.050545	1
Tbl1xr1	-0.0432	0.208356	1
Gtpbp1	-0.0432	0.724729	1
Eif4enif1	-0.04322	0.17235	1
Srfbp1	-0.04325	0.037314	1
Tnks	-0.04327	0.039814	1
Tctex1d2	-0.04332	0.490569	1
Eif2ak4	-0.04334	0.000139	1
Stat2	-0.0434	0.169611	1
Sacm1l	-0.04341	0.588897	1
Trappc6b	-0.04348	0.109412	1
Pfdn6	-0.04354	0.088285	1
4931406P:	-0.04355	0.320488	1
Rlf	-0.04376	0.130931	1
Pctp	-0.04377	0.954017	1
Tefm	-0.0438	0.98101	1
Rab21	-0.04381	0.412106	1
Usp37	-0.04383	0.15874	1
Ngrn	-0.04385	0.362326	1
Pdcd2l	-0.04389	0.141485	1
Sass6	-0.04398	0.097402	1
Tfcp2	-0.04405	0.404482	1
Fam160b2	-0.04406	0.230856	1
Slc25a17	-0.0441	0.081793	1
Ss18	-0.04413	0.132721	1
Rnasek	-0.04413	0.431857	1
Dbndd2	-0.04413	0.099411	1
Fdft1	-0.04421	0.692675	1
Phf20	-0.04426	0.066406	1
Top1	-0.04435	0.65126	1
Cspp1	-0.04443	0.297203	1

Selenoi	-0.04448	0.642948	1
Trp53bp2	-0.0445	0.307776	1
Cenpq	-0.04452	0.482678	1
Hectd4	-0.04456	0.006517	1
Csnk1a1	-0.04463	0.958733	1
Mon1a	-0.04464	0.452123	1
Hdac9	-0.04467	0.914424	1
Toe1	-0.04469	0.125937	1
L3mbtl2	-0.04474	0.540138	1
Cir1	-0.04475	0.21572	1
Pex7	-0.04485	0.247554	1
Ivns1abp	-0.04496	0.193422	1
Arid3b	-0.04498	0.975298	1
Dnajb9	-0.04499	0.697415	1
Hif1an	-0.0451	0.455966	1
Gm16599	-0.04511	0.653408	1
Wdr25	-0.04511	0.330391	1
Vrk2	-0.04512	0.357467	1
Fam160b1	-0.04516	0.402788	1
Nprl2	-0.0452	0.027081	1
Rgp1	-0.04521	0.182427	1
Dhx57	-0.04526	0.679395	1
Usp22	-0.04531	0.398925	1
5031439G(	-0.04533	0.652078	1
Abca2	-0.04534	0.175933	1
Prrc1	-0.04536	0.165834	1
Arglu1	-0.04536	0.19788	1
Fcho2	-0.04539	0.820059	1
Zfp780b	-0.04545	0.972009	1
Anapc2	-0.04554	0.284694	1
Kiz	-0.04554	0.114741	1
Ripk1	-0.04561	0.089954	1
Plekhg1	-0.04563	0.323747	1
Dgat1	-0.04576	7.4E-05	1
Thumpd3	-0.04584	0.061393	1
Arf2	-0.04585	0.332885	1
Btf3l4	-0.04587	0.455166	1
Cactin	-0.04593	0.041795	1
Cnppd1	-0.04594	0.492372	1
Rnf139	-0.0461	0.970962	1
Ccdc134	-0.0461	0.15251	1
Trdmt1	-0.04613	0.126021	1
Appl2	-0.04613	0.009306	1
Dele1	-0.04619	0.283043	1
Kmt5c	-0.04626	0.370105	1
Slc50a1	-0.04626	0.70987	1
Parp1	-0.04627	0.41635	1
Tspan17	-0.04629	0.011018	1

Tada1	-0.0463	0.323913	1
Scoc	-0.04631	0.755305	1
Atg12	-0.04632	0.391395	1
Golga7	-0.04633	0.152442	1
Ufsp2	-0.04635	0.39263	1
Nup188	-0.04636	0.730095	1
Ncbp3	-0.04639	0.117897	1
Gskip	-0.04644	0.461818	1
Tcea1	-0.04654	0.489979	1
Acads	-0.04655	0.379806	1
Fadd	-0.04669	0.505101	1
Ubr2	-0.0467	0.146059	1
Snx13	-0.04674	0.481607	1
Pcgf1	-0.04694	0.312433	1
Trappc10	-0.04695	0.15578	1
Dcaf13	-0.04697	0.182172	1
Ubqln4	-0.04707	0.858089	1
Sec23a	-0.04726	0.795261	1
Dcaf8	-0.04726	0.51531	1
Zfp994	-0.04727	0.778342	1
Ccdc174	-0.04744	0.721906	1
Lpcat3	-0.04748	0.203595	1
Cd2ap	-0.04748	0.810815	1
Ddit4	-0.04758	0.422824	1
Plcb2	-0.04762	0.707996	1
Rnf126	-0.04765	0.3938	1
Tmem19	-0.04771	0.290696	1
Clptm1l	-0.04778	0.059661	1
Kdm1b	-0.04779	0.979284	1
Xylt2	-0.04782	0.479192	1
Rassf2	-0.04785	0.560529	1
Ddx19b	-0.04793	0.469714	1
Me2	-0.04797	0.901094	1
Selenof	-0.048	0.507057	1
Alg10b	-0.04803	0.228532	1
Gpatch3	-0.04804	0.293853	1
Ier3ip1	-0.0481	0.334614	1
Tmem222	-0.04821	0.116479	1
Sar1b	-0.04823	0.0599	1
Mdc1	-0.04831	0.746483	1
Polr2l	-0.04846	0.340076	1
Siglece	-0.04855	0.150486	1
Xpnpep3	-0.04857	0.289084	1
Dcaf11	-0.04858	0.263534	1
Mettl14	-0.04859	0.346542	1
Eif2ak2	-0.04866	0.324537	1
Elac2	-0.04869	0.001332	1
Nkiras1	-0.04875	0.386221	1

Slc35a5	-0.04876	0.100134	1
Ptcd2	-0.04877	0.345868	1
Lmo2	-0.04881	0.612064	1
Vps45	-0.049	0.224546	1
Ythdf2	-0.04904	0.311738	1
March7	-0.04907	0.136576	1
Dhrs4	-0.04927	0.629545	1
Erp44	-0.04935	0.353021	1
Clint1	-0.04936	0.865669	1
Epb41	-0.04939	0.151643	1
Rfc2	-0.0494	0.259218	1
Snrnp200	-0.04941	0.32947	1
Tmed3	-0.04941	0.686539	1
Rnf34	-0.04951	0.210806	1
Strbp	-0.04956	0.496168	1
Ccnt2	-0.04965	0.277506	1
Hcst	-0.04967	0.582771	1
1700037C1	-0.04971	0.509184	1
Zbed4	-0.04971	0.802119	1
Itpkb	-0.04976	0.76368	1
Kctd2	-0.04978	0.60659	1
Sirpb1c	-0.04979	0.884504	1
Arfgap2	-0.04982	0.221671	1
Prpf4	-0.04988	0.269168	1
Gm48678	-0.04989	0.860632	1
Tcf25	-0.04993	0.900346	1
Rab2b	-0.04996	0.832699	1
Ddx42	-0.04996	0.693917	1
Calcoco1	-0.05003	0.703179	1
Ankrd10	-0.05005	0.738917	1
Ipmk	-0.05006	0.361684	1
Trim14	-0.05008	0.240742	1
Nudt16	-0.05008	0.0886	1
Elmod2	-0.05012	0.585941	1
Atg2b	-0.05014	0.107841	1
Med28	-0.05015	0.367985	1
Lat2	-0.05023	0.373932	1
Hsdl1	-0.05024	0.067897	1
C2cd3	-0.05038	0.854683	1
Ppard	-0.0504	0.011495	1
Supt4a	-0.05042	0.924585	1
Wbp11	-0.05046	0.406159	1
Invs	-0.05047	0.329344	1
A430005L1	-0.05048	0.337475	1
Slx1b	-0.05053	0.127969	1
Utp6	-0.05061	0.217152	1
Tmem175	-0.05076	0.568231	1
Rab3gap2	-0.05076	0.295132	1

Bet1l	-0.05078	0.752838	1
Aste1	-0.05079	0.287775	1
Ssh1	-0.0508	0.109995	1
Aak1	-0.05083	0.06979	1
Lrrc8b	-0.05086	0.701155	1
Lysmd3	-0.05095	0.650692	1
Tec	-0.051	0.311695	1
Cdc73	-0.05103	0.318639	1
Usp6nl	-0.05106	0.190883	1
Gm20513	-0.05106	0.17482	1
Polr3c	-0.05106	0.564837	1
Gm16845	-0.05114	0.831636	1
Stt3b	-0.05117	0.499522	1
Dnajb14	-0.05118	0.065071	1
Ap4m1	-0.05118	0.087222	1
Exoc1	-0.05118	0.021644	1
Pdcd11	-0.05122	0.256721	1
Dvl3	-0.05122	0.363039	1
Lrrc57	-0.05122	0.238306	1
Usp38	-0.05123	0.542062	1
Sft2d2	-0.05134	0.050496	1
Nde1	-0.05136	0.0385	1
Kctd13	-0.05138	0.228239	1
Atf7	-0.05141	0.869999	1
Pak1	-0.05141	0.106085	1
Ireb2	-0.05154	0.485342	1
Ttf1	-0.05163	0.036148	1
Zfp672	-0.0517	0.445011	1
Mark3	-0.05175	0.293077	1
Ap5b1	-0.05184	0.571466	1
Morf4l1	-0.05192	0.967919	1
Mtr	-0.05192	0.245929	1
Pdss2	-0.05195	0.190275	1
Tpst2	-0.05195	0.906395	1
Mtf2	-0.05196	0.145267	1
Traf7	-0.05204	0.9319	1
Recql5	-0.05205	0.840799	1
Otud4	-0.05207	0.050765	1
Cd2bp2	-0.05208	0.300649	1
Hsph1	-0.05213	0.404389	1
Cry1	-0.05218	0.277706	1
Idi1	-0.05222	0.282892	1
Cfap97	-0.05226	0.291228	1
Ehmt1	-0.05228	0.497313	1
Spty2d1	-0.05231	0.368055	1
Xrcc4	-0.05233	0.430132	1
Gopc	-0.05233	0.453377	1
Dedd	-0.05234	0.729289	1

Ring1	-0.05241	0.320771	1
Arih2	-0.05245	0.986275	1
Hmg20a	-0.05249	0.233955	1
LTO1	-0.0525	0.536253	1
4930523C(	-0.05255	0.903636	1
Entpd6	-0.05259	0.570412	1
Senp3	-0.05259	0.308447	1
Sumf2	-0.05261	0.144403	1
Sh3bp4	-0.05263	0.391503	1
Golga4	-0.05267	0.019087	1
Gigyf1	-0.05268	0.270558	1
Rab14	-0.05271	0.760056	1
Cog4	-0.05279	0.332146	1
Txndc16	-0.05282	0.454538	1
Rps28	-0.0529	0.217345	1
Gm20732	-0.05295	0.635168	1
Rnf40	-0.05296	0.207544	1
Pnkd	-0.05308	0.191128	1
Rassf1	-0.05312	0.293	1
Ube3b	-0.05316	0.128511	1
Lrrc61	-0.05317	0.955361	1
Nr1d2	-0.05324	0.737536	1
Mlh3	-0.05335	0.713397	1
Lrch4	-0.05336	0.542759	1
Prpsap2	-0.05339	0.414141	1
Iqce	-0.05345	0.122211	1
Fuz	-0.05354	0.884131	1
Disc1	-0.0536	0.395241	1
Lrrc40	-0.05361	0.394994	1
Rab10os	-0.05372	0.338629	1
Rpl39	-0.05386	0.169912	1
Xpo7	-0.05388	0.687318	1
Trappc4	-0.05394	0.357627	1
Tssc4	-0.05398	0.159758	1
Mosmo	-0.05409	0.262502	1
Vhl	-0.05411	0.049777	1
Tmed4	-0.05421	0.90084	1
Cyb5b	-0.05422	0.187471	1
Dctn1	-0.05423	0.341748	1
Yy1	-0.05425	0.060634	1
Vav2	-0.05434	0.411775	1
Btbd10	-0.05435	0.636778	1
Ywhab	-0.0544	0.813977	1
Ubr5	-0.05445	0.07189	1
Lrp10	-0.05451	0.48325	1
Cdk9	-0.05456	0.102438	1
Ston2	-0.05457	0.233077	1
Bphl	-0.05468	0.981597	1

Orai2	-0.05472	0.371469	1
Ppil2	-0.05475	0.535621	1
Nectin1	-0.05479	0.705665	1
Pwwp2a	-0.05485	0.354686	1
Scnm1	-0.0549	0.389214	1
Mtmr4	-0.05494	0.808561	1
Ubr3	-0.05499	0.255582	1
Nktr	-0.055	0.10812	1
Ifit1bl1	-0.05504	0.529945	1
Snip1	-0.05506	0.301948	1
Aup1	-0.05509	0.940043	1
Gsk3a	-0.05509	0.066246	1
Cnot4	-0.0551	0.895858	1
Reps1	-0.05518	0.269562	1
Rabgap1	-0.05518	0.535008	1
Dhx35	-0.05521	0.152821	1
Pigc	-0.05527	0.09921	1
4732471J0	-0.0553	0.451617	1
Lyrm1	-0.05532	0.528332	1
Zbtb1	-0.05533	0.212533	1
Rnf123	-0.05546	0.886746	1
Dhx30	-0.05557	0.052955	1
Cnnm4	-0.05557	0.727229	1
Exoc6	-0.05559	0.775803	1
Caap1	-0.05562	0.406046	1
Ankrd27	-0.05568	0.305501	1
Spsb2	-0.05582	0.867356	1
Sprtn	-0.05583	0.389325	1
U2af1l4	-0.05583	0.863915	1
Man1c1	-0.05584	0.285886	1
Irgc1	-0.05588	0.528915	1
Cstf3	-0.05588	0.175126	1
Rxylt1	-0.05599	0.057532	1
Uba3	-0.05601	0.295144	1
Col4a3bp	-0.05601	0.735484	1
Slc4a2	-0.05605	0.116338	1
Peli1	-0.05611	0.696033	1
Map2k7	-0.05614	0.06886	1
Slc39a11	-0.05621	0.327517	1
Cd300lg	-0.0564	0.765019	1
Nrde2	-0.05646	0.694823	1
Hdac4	-0.05649	0.230301	1
Eci1	-0.05649	0.106608	1
Lipa	-0.05652	0.195794	1
Wasl	-0.05655	0.879296	1
Smox	-0.05661	0.422898	1
Adck1	-0.05663	0.620483	1
Tmem120a	-0.05666	0.112458	1

Impa1	-0.05667	0.72241	1
Pde3b	-0.05672	0.056584	1
Gmeb1	-0.05674	0.516103	1
Sike1	-0.05675	0.913653	1
Hdac3	-0.05681	0.860186	1
Uba7	-0.05685	0.51027	1
Fam120b	-0.05698	0.460922	1
Slc41a3	-0.05699	0.298519	1
Ccdc102a	-0.05704	0.805753	1
Mgme1	-0.05707	0.141592	1
Nxt2	-0.05709	0.845301	1
Setdb1	-0.05709	0.068466	1
Ang	-0.05718	0.202657	1
Ino80b	-0.05718	0.068414	1
Otulin	-0.05723	0.723261	1
Rps6kb1	-0.05726	0.204444	1
Csnk1d	-0.05737	0.637083	1
Nlrc5	-0.05738	0.276434	1
Srsf1	-0.05743	0.028187	1
Gm14326	-0.05745	0.500439	1
Lonp2	-0.05748	0.75339	1
Exoc2	-0.05753	0.770281	1
Morc2a	-0.05755	0.229671	1
Slc39a6	-0.05755	0.282273	1
Bcas2	-0.05764	0.257275	1
Kptn	-0.0578	0.828493	1
Capza2	-0.05784	0.436495	1
Ssbp3	-0.05788	0.253913	1
Rab29	-0.0579	0.576495	1
Ddx1	-0.05791	0.136557	1
Pmm2	-0.05806	0.977568	1
Sfpq	-0.05807	0.644129	1
Ppp2r3d	-0.05812	0.463457	1
Nbr1	-0.05812	0.071462	1
Atp6v1a	-0.05814	0.77775	1
Ppp1r15b	-0.05827	0.400413	1
Trim56	-0.05827	0.288597	1
Spg7	-0.05833	0.345782	1
Ap5m1	-0.05834	0.861267	1
Mrpl39	-0.05837	0.53904	1
Dtx2	-0.05842	0.490495	1
Bcl9	-0.05849	0.209446	1
Rbm47	-0.05852	0.466729	1
Ccdc84	-0.05854	0.870493	1
Nrp1	-0.05856	0.751416	1
Pak4	-0.05857	0.527984	1
Ern1	-0.05857	0.174755	1
Lpcat2	-0.05864	0.79959	1

Tmem159	-0.05865	0.556428	1
Igf1r	-0.05866	0.753938	1
Mbtps2	-0.05869	0.392223	1
Izumo4	-0.0587	0.393961	1
Derl1	-0.05887	0.910216	1
Prpf3	-0.05888	0.088429	1
Tmem223	-0.05888	0.079095	1
Fam98b	-0.0589	0.790253	1
Upf3b	-0.05892	0.114938	1
Cbfa2t2	-0.05894	0.48146	1
Nsmaf	-0.05894	0.491368	1
Rreb1	-0.05903	0.871822	1
Gtpbp3	-0.05904	0.230853	1
Raet1e	-0.05912	0.965346	1
Nob1	-0.05914	0.374352	1
Saysd1	-0.05922	0.137472	1
Msl1	-0.05928	0.261773	1
Zer1	-0.05934	0.67398	1
Eml2	-0.05936	0.400599	1
Zkscan14	-0.05943	0.719474	1
Atl2	-0.05948	0.747641	1
Commd6	-0.05948	0.160595	1
Klhl11	-0.05956	0.324105	1
Khdc4	-0.05957	0.523897	1
Aimp1	-0.0596	0.769412	1
D11Wsu47	-0.05971	0.22237	1
Herc3	-0.05973	0.598261	1
Dda1	-0.05982	0.008482	1
Rfwd3	-0.05987	0.942851	1
Asb6	-0.05999	0.769104	1
Wbp1l	-0.06004	0.829246	1
Zfp36	-0.06007	0.452443	1
Metap1	-0.06007	0.275984	1
Pop5	-0.06012	0.634583	1
Tmtc3	-0.06035	0.819156	1
Pigv	-0.06047	0.678592	1
Pnpla7	-0.0605	0.974308	1
Zfp280c	-0.06056	0.924039	1
Lsg1	-0.06066	0.06949	1
Ercc5	-0.0607	0.712748	1
Zc3h18	-0.0607	0.408657	1
Efl1	-0.06074	0.932935	1
Traf3ip2	-0.06081	0.295097	1
Nat2	-0.06085	0.72189	1
Ctcf	-0.06088	0.074843	1
Nono	-0.06088	0.599669	1
Fam76b	-0.06089	0.273103	1
Ctsb	-0.06094	0.051154	1

Srsf4	-0.06094	0.212684	1
Rsad2	-0.06102	0.616601	1
Vgll4	-0.06114	0.020777	1
Pam16	-0.06119	0.060845	1
Tbce	-0.06123	0.722414	1
Paip2	-0.06125	0.79294	1
Hipk1	-0.06127	0.124551	1
Avl9	-0.06128	0.480588	1
Haghl	-0.06131	0.419654	1
Snx19	-0.06137	0.652249	1
Vwa5a	-0.06142	0.461835	1
Pithd1	-0.06146	0.397923	1
Fxr1	-0.06157	0.277154	1
Ddx49	-0.06157	0.069661	1
Ikbip	-0.06158	0.5631	1
Mtap	-0.06177	0.676714	1
Vps13a	-0.06179	0.766125	1
Ndufa6	-0.06179	0.359159	1
Slc9a1	-0.06183	0.116717	1
Cwc27	-0.06194	0.031325	1
Sf3b2	-0.06196	0.739267	1
St7l	-0.06205	0.485502	1
Rtn4	-0.06208	0.487716	1
Vps11	-0.06208	0.006869	1
Shprh	-0.06217	0.352885	1
Cerkl	-0.0622	0.706411	1
Ogfod2	-0.06224	0.946968	1
B3galt4	-0.06226	0.953903	1
Smad1	-0.06227	0.114337	1
Rrnad1	-0.06228	0.728767	1
Mrs2	-0.06233	0.280725	1
Pold3	-0.06239	0.126786	1
Plekha3	-0.06253	0.60295	1
Fopnl	-0.06272	0.902549	1
Ppp1r37	-0.06282	0.619204	1
Crot	-0.06288	0.448668	1
Fam50a	-0.06309	0.806485	1
Lpin1	-0.06311	0.266239	1
Appbp2	-0.06316	0.334236	1
Xpr1	-0.06322	0.367976	1
Pigm	-0.06328	0.846839	1
Cdk5	-0.0633	0.259623	1
Snx27	-0.0633	0.178219	1
Bag6	-0.06351	0.049143	1
Commd10	-0.06355	0.087982	1
Cisd2	-0.06358	0.206571	1
Exosc10	-0.0637	0.70043	1
Fxr2	-0.06373	0.219013	1

Arap3	-0.06378	0.03884	1
Snap23	-0.06384	0.782834	1
Diaph1	-0.06385	0.544283	1
Pptc7	-0.06396	0.178162	1
Coq2	-0.06396	0.839286	1
Arpc1b	-0.06406	0.334605	1
Cyp4f17	-0.06406	0.940306	1
Npepps	-0.06408	0.14197	1
Relb	-0.06411	0.418409	1
Ldlrad4	-0.06417	0.070689	1
Suds3	-0.06427	0.117059	1
R3hcc1l	-0.06437	0.319647	1
Gm45894	-0.06448	0.764478	1
Alg1	-0.06454	0.280397	1
Ap3m2	-0.06454	0.609148	1
Mcu	-0.06456	0.989129	1
Acad9	-0.06467	0.814611	1
Emc3	-0.06468	0.157289	1
Mrpl53	-0.06468	0.044514	1
Slc25a36	-0.06478	0.683906	1
Mtmr2	-0.06481	0.10496	1
Prr14	-0.06486	0.133025	1
Gm2000	-0.0649	0.816967	1
Fam204a	-0.06498	0.349732	1
Ncoa5	-0.06502	0.253851	1
Pts	-0.06503	0.891743	1
Pum1	-0.06508	0.42787	1
C2cd2	-0.06508	0.623285	1
Gga1	-0.06512	0.131828	1
Bms1	-0.06514	0.143136	1
Agpat1	-0.06521	0.422436	1
Fancl	-0.06531	0.713755	1
Mtmr12	-0.06531	0.252592	1
Lrrc45	-0.06537	0.805215	1
Haus6	-0.06544	0.153092	1
Dnajb12	-0.0655	0.474906	1
Nf1	-0.06552	0.228409	1
Ptpn11	-0.0656	0.51396	1
Ccdc186	-0.06562	0.392412	1
Spg11	-0.0657	0.478627	1
Tapbpl	-0.06575	0.234207	1
Med14	-0.06584	0.154645	1
Prps2	-0.06584	0.475188	1
Lin7c	-0.06591	0.180879	1
Eif3h	-0.06596	0.725983	1
Utp3	-0.06596	0.943975	1
Gm43813	-0.06597	0.620293	1
Vps39	-0.066	0.24486	1

Selenok	-0.06601	0.900305	1
Dcaf17	-0.06614	0.392325	1
Acvr1	-0.06615	0.719388	1
Ifi206	-0.06615	0.044221	1
Zfp839	-0.0662	0.119197	1
Usp1	-0.06621	0.468722	1
Cacul1	-0.06624	0.479468	1
Rev1	-0.0663	0.719473	1
Csnk1g1	-0.06636	0.487285	1
Ago1	-0.06646	0.285184	1
Cstf2t	-0.06647	0.596988	1
Hps1	-0.06649	0.489834	1
B230307C2	-0.06652	0.06069	1
Whamm	-0.06655	0.180129	1
Mfn1	-0.06655	0.316275	1
Sel1l	-0.06661	0.304419	1
Gm38394	-0.06662	0.563398	1
Plcl1	-0.06686	0.511746	1
Sphk2	-0.06688	0.640716	1
Stam	-0.06706	0.601642	1
Hsd11b1	-0.06711	0.383894	1
Ulk2	-0.06713	0.072053	1
Gsdme	-0.06724	0.665112	1
Mfsd10	-0.06728	0.363432	1
Plekhg2	-0.0673	0.885271	1
Gse1	-0.06731	0.702944	1
Ip6k2	-0.06732	0.150817	1
Blm	-0.06733	0.701194	1
Map4	-0.06735	0.829959	1
Tab3	-0.06736	0.551575	1
4833438C0	-0.06749	0.223438	1
Prepl	-0.06751	0.600158	1
Ppp1r8	-0.06753	0.684168	1
Sbno1	-0.06754	0.571366	1
Gm15448	-0.06761	0.335213	1
Sde2	-0.06772	0.919239	1
Rbm41	-0.06772	0.763473	1
Map4k1	-0.06774	0.692329	1
Ssb	-0.06774	0.86631	1
Slc35c1	-0.06779	0.467924	1
Man2b1	-0.06783	0.216152	1
Scyl3	-0.06784	0.259934	1
Zscan29	-0.06789	0.205904	1
Zfp131	-0.06797	0.673429	1
Tmem150b	-0.06801	0.80367	1
Ptcd3	-0.06802	0.440172	1
Ppp1r12b	-0.06828	0.005593	1
Mre11a	-0.06835	0.231058	1

Nfatc2ip	-0.06837	0.875715	1
Gatc	-0.06839	0.600158	1
Riox2	-0.06841	0.623564	1
Ranbp3	-0.06841	0.264911	1
Hexim1	-0.06841	0.661253	1
Pcyt2	-0.06847	0.620384	1
Diablo	-0.06847	0.979843	1
Cklf	-0.06851	0.869707	1
Cog3	-0.06859	0.240015	1
Lnpep	-0.06862	0.577839	1
Smad7	-0.06873	0.793167	1
Cers6	-0.0688	0.555071	1
Ulk3	-0.06882	0.243046	1
Timm17b	-0.06886	0.077248	1
Wdr53	-0.069	0.781843	1
Mrpl4	-0.06904	0.961835	1
Fbxo7	-0.06905	0.218072	1
Cryzl1	-0.06909	0.341223	1
Cyb561d1	-0.06926	0.377828	1
Uimc1	-0.06932	0.410465	1
Gm44686	-0.06932	0.648628	1
Trak1	-0.06942	0.13133	1
Rbm48	-0.06954	0.781912	1
Cep128	-0.06958	0.952759	1
Lias	-0.06971	0.363849	1
Esrra	-0.06976	0.052862	1
Ercc1	-0.06977	0.475478	1
Ugcg	-0.0698	0.498037	1
Naa60	-0.06984	0.706421	1
Ggps1	-0.06986	0.193472	1
Kidins220	-0.06994	0.179636	1
9030025P2	-0.07004	0.898495	1
Rabggta	-0.07004	0.938645	1
Pcnt	-0.0702	0.375951	1
Rnf144b	-0.07026	0.012887	1
Zbtb44	-0.07034	0.834498	1
Lclat1	-0.07037	0.916795	1
Zfp715	-0.07043	0.456686	1
Gm16675	-0.07044	0.529808	1
Clec16a	-0.07048	0.906474	1
Pepd	-0.07056	0.727236	1
Srrm1	-0.07057	0.30343	1
Hectd1	-0.07058	0.903346	1
Capn15	-0.07058	0.708072	1
Slc35f5	-0.07066	0.009008	1
P2ry10b	-0.07067	0.962076	1
Rogdi	-0.07068	0.420205	1
Xpc	-0.07072	0.426149	1

Zfp729a	-0.07075	0.881284	1
Ncoa1	-0.07081	0.501561	1
Flvcr1	-0.07082	0.242263	1
Rab32	-0.07086	0.729319	1
Mios	-0.0709	0.234685	1
Milr1	-0.07096	0.820254	1
Zfp87	-0.071	0.59794	1
Zdhhc7	-0.07105	0.077255	1
Mrps27	-0.0711	0.50774	1
Plpbp	-0.0711	0.505381	1
Plec	-0.07118	0.940768	1
Psmd5	-0.07125	0.419554	1
Jkamp	-0.07129	0.093566	1
Limk1	-0.07147	0.927013	1
Lemd3	-0.07156	0.187863	1
Eri1	-0.07156	0.443266	1
Nol10	-0.0716	0.588364	1
Rmnd1	-0.07161	0.190103	1
Snx21	-0.07161	0.590009	1
Aebp2	-0.07178	0.146633	1
Gpalpp1	-0.07179	0.373724	1
Emp3	-0.07183	0.621594	1
Gm6034	-0.07188	0.874861	1
Ebag9	-0.07193	0.498213	1
Snhg3	-0.07197	0.069426	1
Cep44	-0.07197	0.612561	1
Rock1	-0.07198	0.579661	1
Nifk	-0.07199	0.056755	1
Cbx5	-0.07199	0.235173	1
March6	-0.07204	0.17608	1
Fubp1	-0.07205	0.755393	1
Cdk5rap1	-0.07209	0.853896	1
Mbtps1	-0.07209	0.135078	1
Mto1	-0.07225	0.798694	1
Mfap1a	-0.07226	0.325602	1
Sumo1	-0.07227	0.359881	1
Ankrd17	-0.07236	0.727754	1
Csnk1g2	-0.07241	0.375607	1
Ankib1	-0.0725	0.180687	1
Mapk8ip3	-0.07256	0.405197	1
Gamt	-0.07257	0.457897	1
Pum3	-0.07259	0.324712	1
Lrif1	-0.0726	0.888756	1
Scrib	-0.07268	0.912339	1
Uba6	-0.07271	0.566755	1
Nup133	-0.07277	0.178748	1
Numb	-0.0728	0.13054	1
AI987944	-0.07282	0.624144	1

Coq5	-0.07311	0.032104	1
Pkd1	-0.07316	0.707637	1
Ifi209	-0.0732	0.314939	1
Ak3	-0.07323	0.981248	1
Cyfip1	-0.07326	0.873545	1
Tsc2	-0.07326	0.737629	1
AW549877	-0.07326	0.136504	1
Ids	-0.07347	0.57319	1
Pcyt1a	-0.07355	0.555472	1
Vrk3	-0.07356	0.466399	1
Coq3	-0.07357	0.980273	1
Leprot	-0.07364	0.234666	1
Prkab1	-0.07367	0.40611	1
Rundc1	-0.0737	0.13333	1
Tnpo1	-0.07371	0.875789	1
Btbd1	-0.07377	0.637953	1
Wwc2	-0.07401	0.477911	1
Lcorl	-0.07402	0.281173	1
Angel2	-0.07432	0.901055	1
Cnbd2	-0.07446	0.390359	1
Acsl5	-0.07448	0.931423	1
BC031181	-0.07452	0.443107	1
Flt3l	-0.07452	0.931161	1
Ppfibp1	-0.07454	0.191471	1
Ints5	-0.07455	0.344904	1
Rlim	-0.0747	0.888588	1
Casp6	-0.07474	0.031833	1
Dot1l	-0.07476	0.185216	1
Cd53	-0.07477	0.521238	1
Ranbp10	-0.07477	0.678828	1
Tbck	-0.07478	0.314886	1
Nup98	-0.07482	0.532229	1
Pibf1	-0.0749	0.197355	1
Hibch	-0.0749	0.956849	1
Slc35b2	-0.075	0.552788	1
Hes6	-0.07503	0.203776	1
Rfesd	-0.07505	0.990205	1
Retreg1	-0.07508	0.133395	1
Gpatch8	-0.07509	0.283565	1
Cnot6	-0.07512	0.184694	1
Kbtbd2	-0.07515	0.716471	1
Lypla1	-0.07523	0.731211	1
Dck	-0.07529	0.561728	1
Dpm2	-0.07535	0.317872	1
Ino80c	-0.0754	0.391209	1
Rpl9	-0.0754	0.160951	1
Oxsr1	-0.0754	0.793659	1
Mtmr1	-0.07544	0.808667	1

Lzic	-0.07545	0.223808	1
Zfyve1	-0.07549	0.023642	1
Tusc1	-0.07561	0.970132	1
Gm32036	-0.07566	0.447814	1
Ankrd13c	-0.07577	0.153153	1
Fiz1	-0.07577	0.787066	1
Atg101	-0.07577	0.438351	1
Dhps	-0.07585	0.256468	1
Zfp637	-0.0759	0.449906	1
Timmdc1	-0.0759	0.660342	1
Papolg	-0.07592	0.691215	1
Pak1ip1	-0.07603	0.392865	1
Cltc	-0.07604	0.639993	1
Cdkal1	-0.07605	0.276422	1
Gtf2b	-0.07605	0.929092	1
Bptf	-0.07606	0.445085	1
Atxn7l3b	-0.07613	0.910179	1
Mpp6	-0.07614	0.034267	1
Nelfcd	-0.07615	0.358637	1
Tdrd3	-0.07619	0.711746	1
Tmem184b	-0.0762	0.190526	1
Cdadc1	-0.07628	0.700318	1
Abcf3	-0.07636	0.91711	1
Mthfs	-0.07637	0.27715	1
Ccdc93	-0.07647	0.565713	1
Ammecr1l	-0.07651	0.088855	1
Rabep2	-0.07657	0.842598	1
Primpol	-0.07668	0.905633	1
9930111J2	-0.07669	0.328148	1
Aff4	-0.07675	0.399236	1
Rgs19	-0.07685	0.417548	1
Spata6	-0.0769	0.608152	1
Tatdn1	-0.07691	0.539832	1
Crebrf	-0.07719	0.561123	1
Hmgn5	-0.0772	0.897946	1
Hhex	-0.07721	0.433487	1
Bnip3l	-0.07721	0.777483	1
Mllt10	-0.07723	0.975659	1
Trim5	-0.07726	0.753437	1
Psmd12	-0.07733	0.872722	1
Ccdc9	-0.07735	0.484831	1
Rcl1	-0.07746	0.877676	1
B4galt4	-0.0775	0.156376	1
Cd72	-0.07753	0.011745	1
Zfp322a	-0.07753	0.574188	1
Med17	-0.0776	0.543014	1
Brd8	-0.0776	0.281193	1
Sgf29	-0.07763	0.505472	1

Flcn	-0.07764	0.580425	1
Vasp	-0.07768	0.939919	1
Wdfy3	-0.07769	0.350813	1
Sap130	-0.07769	0.226515	1
Vars	-0.07771	0.852318	1
2310011J0	-0.07771	0.923969	1
Stk26	-0.07772	0.093727	1
Krcc1	-0.07776	0.471012	1
Akirin1	-0.07778	0.707774	1
Eya3	-0.07781	0.008831	1
Dnaaf2	-0.07788	0.356246	1
Zbtb11	-0.07789	0.311239	1
Pan2	-0.07797	0.964961	1
Fbxo4	-0.07799	0.922562	1
Rnf115	-0.07802	0.590195	1
Fbxl20	-0.07813	0.315607	1
Naa30	-0.07843	0.332015	1
Rmi1	-0.07843	0.667183	1
Szrd1	-0.07871	0.500244	1
Edc3	-0.07874	0.863728	1
Ccdc124	-0.07874	0.386896	1
Parp2	-0.07874	0.300669	1
Phf2	-0.07882	0.752829	1
Map4k5	-0.0789	0.619564	1
Sord	-0.07898	0.439115	1
Rbm15	-0.07901	0.695072	1
Zfp646	-0.07903	0.258775	1
Snx14	-0.07906	0.281697	1
Trappc9	-0.07915	0.120202	1
Smyd3	-0.07935	0.104498	1
Scamp2	-0.07935	0.24757	1
Smc3	-0.07944	0.708081	1
Foxo1	-0.07946	0.577352	1
Cxcr4	-0.07954	0.584526	1
Arhgap12	-0.07974	0.032852	1
Usp12	-0.07978	0.697078	1
Ppwd1	-0.07979	0.251697	1
Ralb	-0.07995	0.259496	1
Map3k20	-0.08001	0.512018	1
Zfp397	-0.08005	0.735007	1
Ywhaq	-0.08014	0.74502	1
Snx18	-0.08014	0.709615	1
Zfp597	-0.08017	0.956849	1
Zranb2	-0.08017	0.219783	1
Iws1	-0.08035	0.418118	1
Rbmxl1	-0.08039	0.393347	1
Polr3gl	-0.08039	0.74832	1
Trim23	-0.0804	0.327078	1

Smarcad1	-0.08043	0.734508	1
Tecr	-0.08044	0.870187	1
Zfp106	-0.08049	0.696652	1
Mlx	-0.0805	0.388102	1
Larp7	-0.0805	0.228014	1
Senp6	-0.08051	0.639819	1
Lonrf1	-0.08052	0.880421	1
Fam45a	-0.08069	0.461603	1
Vps26c	-0.08075	0.22264	1
Gm15417	-0.08077	0.735643	1
Pdgfb	-0.08083	0.230073	1
Gpatch11	-0.08091	0.602717	1
Fam219b	-0.08092	0.767753	1
Mynn	-0.08101	0.06457	1
Snapc3	-0.08105	0.599812	1
Ecpas	-0.08114	0.317203	1
Fam193b	-0.08116	0.897628	1
Irak4	-0.08116	0.747337	1
Becn1	-0.08118	0.94502	1
Hsf2	-0.08119	0.70207	1
Wwp1	-0.08132	0.631455	1
N6amt1	-0.08134	0.565101	1
6530402F1	-0.08135	0.234484	1
Banp	-0.08139	0.923308	1
4930453N1	-0.0814	0.333748	1
Mbtd1	-0.08142	0.206373	1
Sgsh	-0.08165	0.343789	1
Snrnp40	-0.08166	0.684095	1
Vps52	-0.08194	0.957788	1
Zbtb18	-0.08199	0.591787	1
Dnajc13	-0.08199	0.101103	1
Terf2ip	-0.08207	0.918891	1
Gtf2e2	-0.08214	0.44232	1
Erlec1	-0.08215	0.22206	1
Smarcc2	-0.0823	0.818297	1
Vps37a	-0.08232	0.402544	1
Timm44	-0.08233	0.847447	1
Exosc3	-0.08239	0.826975	1
Inpp4a	-0.08248	0.818687	1
Sirt7	-0.08252	0.545779	1
Apopt1	-0.08257	0.748045	1
Rhbdd1	-0.08266	0.774445	1
Fam160a2	-0.08271	0.626272	1
Smad4	-0.08272	0.801806	1
Mien1	-0.08275	0.870384	1
Rad50	-0.08281	0.204999	1
Slc25a32	-0.08282	0.875993	1
Rb1cc1	-0.08296	0.933174	1

Zfp329	-0.08306	0.967476	1
Dynlt1c	-0.08308	0.834915	1
Cd68	-0.08311	0.571428	1
Hspa1a	-0.08316	0.154523	1
Pex2	-0.08325	0.018959	1
Cnot7	-0.08336	0.622811	1
Tut1	-0.08342	0.988051	1
Polm	-0.08342	0.587356	1
Mindy1	-0.08342	0.27288	1
Terf2	-0.08344	0.556461	1
Nup153	-0.08373	0.333482	1
Gpbp1l1	-0.08375	0.537488	1
Chuk	-0.08375	0.96695	1
Nol11	-0.08384	0.549465	1
Gm5165	-0.08386	0.660088	1
Dedd2	-0.08399	0.112476	1
Wbp4	-0.08402	0.462321	1
Trim34a	-0.08405	0.628822	1
Trafd1	-0.08408	0.851482	1
Plod3	-0.08417	0.717623	1
Snx17	-0.08423	0.574008	1
Yipf2	-0.08425	0.178674	1
Skap2	-0.08427	0.128913	1
Pot1a	-0.0843	0.492149	1
Cpne3	-0.08434	0.670044	1
Clk3	-0.08438	0.795208	1
Retreg3	-0.0844	0.814559	1
Bloc1s3	-0.08443	0.2624	1
Nsun3	-0.08451	0.559775	1
Chic2	-0.08463	0.748912	1
Naa80	-0.08465	0.561757	1
Ctdspl2	-0.08466	0.405057	1
1810013L2	-0.08473	0.582977	1
Kif2a	-0.08495	0.527197	1
Ankfy1	-0.08496	0.673653	1
Rptor	-0.08496	0.711216	1
Erh	-0.08499	0.941651	1
Poc1b	-0.08513	0.44635	1
Cpsf6	-0.08518	0.070195	1
Fosb	-0.0852	0.54087	1
Akirin2	-0.08526	0.612289	1
Trip12	-0.08527	0.783255	1
Xrn1	-0.08527	0.597571	1
Bcl2l13	-0.08537	0.938363	1
Zfp961	-0.08545	0.617969	1
Wdr82	-0.08551	0.835056	1
Ap4s1	-0.08552	0.942603	1
Tgfbrap1	-0.08554	0.959051	1

Prkag1	-0.08554	0.311495	1
5530601H(	-0.08555	0.521658	1
Dgat2	-0.08555	0.161717	1
Dhx37	-0.08556	0.754301	1
Cers2	-0.08577	0.94953	1
Pphln1	-0.0858	0.56008	1
Trim26	-0.08582	0.02129	1
Ubn2	-0.08583	0.213014	1
Hnrnpk	-0.08585	0.343652	1
Bag3	-0.08585	0.292186	1
Grb2	-0.08589	0.813254	1
Ube2d2a	-0.08598	0.372663	1
Ociad1	-0.0862	0.69528	1
Rspo1	-0.08622	0.381646	1
Stambpl1	-0.08622	0.067032	1
Ccdc82	-0.08623	0.709447	1
Snrpa	-0.08631	0.516781	1
Gpaa1	-0.08631	0.872391	1
Pitrm1	-0.08636	0.939781	1
Srsf9	-0.08638	0.422708	1
Zdhhc14	-0.08644	0.578113	1
Yme1l1	-0.08648	0.521554	1
Raph1	-0.08651	0.267567	1
Ppp1r9b	-0.08652	0.723699	1
Zc3h11a	-0.08654	0.121212	1
Txlng	-0.08657	0.825022	1
Adnp	-0.08658	0.175605	1
Klhl18	-0.08661	0.558993	1
Nae1	-0.08669	0.25838	1
Mtmr6	-0.08681	0.529203	1
Rdx	-0.0871	0.492958	1
Rbm25	-0.08712	0.33934	1
E2f3	-0.08716	0.502292	1
Anks1b	-0.08717	0.979479	1
Myo5a	-0.08721	0.37787	1
Trak2	-0.08722	0.587526	1
Tpm1	-0.08722	0.983225	1
Kat6b	-0.08723	0.16145	1
Cdc27	-0.08723	0.047957	1
Gzf1	-0.08734	0.896172	1
Tsga10	-0.08741	0.831366	1
Irgm2	-0.08749	0.286216	1
Cic	-0.08756	0.870105	1
Faim	-0.0876	0.207141	1
Gm20528	-0.08762	0.760825	1
Nudt4	-0.08763	0.481288	1
Dcaf12	-0.08766	0.921236	1
Rad51d	-0.08777	0.632983	1

Elavl1	-0.08782	0.456042	1
Parp14	-0.08788	0.826421	1
Trmt1l	-0.0879	0.993438	1
Cdk11b	-0.08796	0.660282	1
Aspscr1	-0.08796	0.497356	1
Ppp6c	-0.08803	0.970123	1
Rnf11	-0.08804	0.35883	1
Vamp2	-0.08806	0.13508	1
Nfxl1	-0.08808	0.697219	1
Atp2b4	-0.0882	0.058398	1
Cnot1	-0.08825	0.766671	1
Ube2g1	-0.08826	0.585405	1
Zfhx3	-0.08827	0.043635	1
Hsf1	-0.08827	0.541564	1
Abhd13	-0.08835	0.476204	1
Cdc23	-0.08849	0.797526	1
Cradd	-0.08851	0.263901	1
Rraga	-0.08857	0.343097	1
Abhd17b	-0.08862	0.473569	1
Epc2	-0.08866	0.328937	1
Mau2	-0.08869	0.776219	1
Kdm2a	-0.08871	0.634864	1
Ppp3r1	-0.08874	0.722878	1
Nipsnap3b	-0.08877	0.080918	1
Vps53	-0.08878	0.73376	1
Pnpla8	-0.08878	0.73144	1
Nfrkb	-0.08881	0.715304	1
Impad1	-0.08883	0.762182	1
Sf3b3	-0.08883	0.642039	1
Rnf219	-0.089	0.542801	1
Mthfsl	-0.08901	0.697246	1
2010013B2	-0.08902	0.022621	1
Nploc4	-0.08909	0.049973	1
Tango2	-0.08915	0.106417	1
Icmt	-0.08916	0.078942	1
U2surp	-0.0892	0.718778	1
Kif16b	-0.08923	0.572082	1
Ppil3	-0.08923	0.121469	1
Naa15	-0.08928	0.624547	1
Smim27	-0.0893	0.769637	1
Prep	-0.08939	0.541877	1
Golga2	-0.08957	0.370562	1
Stat5a	-0.08963	0.736823	1
Usp7	-0.08969	0.370115	1
Pde7a	-0.08973	0.536871	1
Sesn1	-0.0898	0.606519	1
Polr3b	-0.08986	0.897008	1
Mfng	-0.08986	0.347259	1

Pcgf3	-0.08988	0.297688	1
Cdkl4	-0.08989	0.542727	1
Ccng1	-0.08999	0.462667	1
Prkd2	-0.09005	0.254882	1
Rfx7	-0.09008	0.77514	1
AI467606	-0.09011	0.18517	1
Gm6377	-0.09012	0.771284	1
Copa	-0.09033	0.943755	1
Sh2b2	-0.09035	0.24134	1
Mrps21	-0.09046	0.640253	1
4921524J1	-0.09046	0.513955	1
Sos1	-0.09048	0.513888	1
Nkiras2	-0.09056	0.104163	1
Ly96	-0.09059	0.649445	1
Tspan31	-0.0906	0.221409	1
Ccdc28b	-0.09061	0.398734	1
Dync1li1	-0.09065	0.409013	1
Terf1	-0.09078	0.45936	1
Stard5	-0.09082	0.460346	1
Ptpra	-0.09082	0.328744	1
Sh3gl1	-0.09098	0.446031	1
Acot8	-0.09114	0.675532	1
Utp11	-0.09118	0.109734	1
Ilk	-0.09118	0.662621	1
Rnft1	-0.09121	0.724047	1
Eloa	-0.09123	0.991598	1
Sipa1l2	-0.09125	0.878374	1
Tubgcp4	-0.09145	0.907057	1
Srcap	-0.09148	0.514597	1
Xxylt1	-0.09149	0.674361	1
Cers5	-0.09149	0.647874	1
Clns1a	-0.0915	0.239596	1
Arsa	-0.09158	0.437449	1
Hlx	-0.09163	0.917108	1
Mcts1	-0.09165	0.652183	1
Rin3	-0.09187	0.57053	1
Soat1	-0.09194	0.513267	1
Gm26510	-0.09196	0.123944	1
Prpf38b	-0.09206	0.686032	1
Zmym5	-0.09206	0.315137	1
Dhx38	-0.09211	0.446147	1
Ccndbp1	-0.09212	0.447658	1
Letm1	-0.09216	0.950918	1
Zdhhc20	-0.09219	0.889417	1
1700123O	-0.09221	0.149889	1
Mfap1b	-0.0923	0.345763	1
Dclre1c	-0.09233	0.449906	1
Ssbp2	-0.09233	0.23175	1

Usp42	-0.09234	0.697726	1
Tifa	-0.09237	0.978304	1
Tprn	-0.09237	0.385015	1
Slc26a2	-0.09239	0.737702	1
Ttc1	-0.09241	0.608481	1
Tex261	-0.09246	0.530077	1
Chst15	-0.09247	0.121422	1
Tesk2	-0.0925	0.318642	1
Samd4b	-0.09254	0.227392	1
Ncbp1	-0.09277	0.266481	1
A930015D(	-0.09289	0.977698	1
Zfand2b	-0.09291	0.427027	1
Zfp592	-0.09292	0.976763	1
Dusp12	-0.09296	0.722347	1
Exosc1	-0.09303	0.532936	1
Frs2	-0.09305	0.58321	1
Commd7	-0.09308	0.773825	1
Thoc5	-0.09314	0.890486	1
N4bp2	-0.09315	0.899942	1
Exosc4	-0.09318	0.06214	1
Ctso	-0.09318	0.362048	1
Rubcn	-0.0932	0.411237	1
Srp54a	-0.09328	0.47741	1
Actr6	-0.09332	0.82609	1
Gabpb1	-0.09336	0.772356	1
Ube2d1	-0.09338	0.693046	1
Rad21	-0.09349	0.858583	1
Ifi27	-0.09349	0.008008	1
Bcl7b	-0.0935	0.523124	1
Ywhag	-0.09351	0.76299	1
Brat1	-0.09354	0.895627	1
5330438D1	-0.09357	0.489897	1
Zfp207	-0.09357	0.944792	1
D130009I1	-0.09365	0.531651	1
Bcdin3d	-0.09374	0.273554	1
5430405H(	-0.0938	0.698433	1
Polg	-0.09391	0.690955	1
Trip4	-0.09393	0.343499	1
Ddx39	-0.09397	0.994283	1
Strn	-0.09398	0.72877	1
Dcun1d1	-0.09402	0.659272	1
Ift20	-0.09403	0.617671	1
Dcaf10	-0.0941	0.656319	1
Edem3	-0.09421	0.231824	1
Ythdf1	-0.09433	0.956047	1
Mgat5	-0.09438	0.94649	1
Agfg1	-0.0944	0.939031	1
Snrnp70	-0.09441	0.602835	1

H1f0	-0.09443	0.381359	1
Impact	-0.09443	0.641739	1
Lasp1	-0.09457	0.702501	1
Clasrp	-0.09467	0.584873	1
Zfp410	-0.09469	0.795814	1
Rbm18	-0.09475	0.895616	1
Cog8	-0.09483	0.496101	1
Rngtt	-0.09484	0.450564	1
Cdc42bpb	-0.09485	0.576464	1
Nr2c1	-0.09497	0.897628	1
Zfp277	-0.0953	0.480461	1
Rnaseh1	-0.09531	0.286554	1
Hypk	-0.09537	0.784551	1
Dnajc7	-0.09542	0.727517	1
Dynlt1a	-0.09549	0.712685	1
Fam210b	-0.0955	0.133299	1
Hdac8	-0.09552	0.649064	1
Atp11c	-0.09566	0.508842	1
Bsdc1	-0.09567	0.595675	1
Chd8	-0.09567	0.536712	1
Nbeal1	-0.09583	0.285277	1
Sirt1	-0.09584	0.593645	1
Dock4	-0.09588	0.516777	1
Kdm5b	-0.09589	0.076379	1
Pde1b	-0.09591	0.157241	1
Lmf1	-0.09597	0.563144	1
Csnk1e	-0.09607	0.444895	1
Irf1	-0.09609	0.919447	1
Rgl2	-0.09615	0.753124	1
Fam234a	-0.09616	0.2256	1
Isy1	-0.09619	0.121602	1
Naxd	-0.0962	0.911638	1
Supt16	-0.09621	0.926156	1
Abraxas2	-0.09631	0.890561	1
BC005624	-0.09634	0.557027	1
Zbtb24	-0.09636	0.950642	1
Tm6sf1	-0.09645	0.296979	1
Il6st	-0.09649	0.303197	1
Khnyn	-0.09649	0.716587	1
Ip6k1	-0.09658	0.171399	1
St3gal2	-0.09659	0.045177	1
Pck2	-0.09661	0.989584	1
Dnajc8	-0.09672	0.787803	1
Otud5	-0.09673	0.70928	1
Parp9	-0.09678	0.652729	1
Rpe	-0.09682	0.342009	1
Kctd20	-0.09692	0.921718	1
Acin1	-0.09706	0.765661	1

Mospd2	-0.0971	0.723496	1
Cntln	-0.09712	0.81296	1
Sept11	-0.09717	0.875911	1
Ralgps1	-0.09724	0.981969	1
Chst12	-0.09725	0.625976	1
Tmem60	-0.09729	0.00433	1
Tdrd7	-0.0974	0.323768	1
Gba2	-0.09742	0.501096	1
Tmem234	-0.09743	0.31546	1
Snx30	-0.09751	0.331924	1
Gcc2	-0.09764	0.480207	1
Ssr1	-0.09772	0.760889	1
Scai	-0.09773	0.042654	1
Naa16	-0.09777	0.152505	1
Lypla2	-0.09792	0.674658	1
Rnd3	-0.098	0.83618	1
Taf7	-0.09807	0.879812	1
Slc25a11	-0.09808	0.695464	1
Numa1	-0.09812	0.874398	1
Fis1	-0.09821	0.453135	1
Hmgcr	-0.09826	0.474557	1
Trim8	-0.09838	0.311528	1
Parl	-0.09841	0.837655	1
Itprip	-0.09847	0.48386	1
Asxl1	-0.09849	0.56444	1
Ubn1	-0.09849	0.728806	1
Wars2	-0.09862	0.780021	1
Fbxo11	-0.09867	0.831371	1
Hnrnpu	-0.09871	0.061953	1
Pcnx3	-0.09879	0.981615	1
Ctc1	-0.09889	0.340545	1
Casp1	-0.09894	0.985503	1
Vps8	-0.09895	0.671795	1
Cgas	-0.09898	0.720082	1
Itsn1	-0.099	0.339341	1
Lrig2	-0.099	0.829783	1
Arhgef18	-0.09901	0.583603	1
Mark4	-0.09901	0.861177	1
Slf2	-0.09914	0.624339	1
Sntb2	-0.09927	0.420234	1
Actn4	-0.09929	0.592423	1
Smim12	-0.0993	0.280344	1
Cwc25	-0.09941	0.416325	1
Men1	-0.09948	0.419075	1
Bbip1	-0.09953	0.404282	1
Vps4b	-0.09964	0.779926	1
Evi2	-0.09977	0.009186	1
Zfp984	-0.09992	0.687736	1

Casc3	-0.09994	0.793405	1
Dnajc14	-0.09996	0.08065	1
Tor1a	-0.1	0.562839	1
B4galt3	-0.10004	0.682634	1
Gapvd1	-0.10027	0.323491	1
Tmem181a	-0.10037	0.279843	1
Calcrl	-0.10038	0.673522	1
Slc17a5	-0.10038	0.74684	1
C330007P(	-0.10039	0.918155	1
Ap1g1	-0.1006	0.51584	1
Arnt	-0.1006	0.779046	1
Fkbp15	-0.10065	0.621351	1
Cyth4	-0.1007	0.572669	1
Brd7	-0.10073	0.905517	1
Atg9a	-0.10082	0.244789	1
Armc1	-0.1009	0.050507	1
Tnf	-0.10091	0.918937	1
Ube3c	-0.10142	0.474283	1
Pex6	-0.10143	0.294821	1
Med13l	-0.10153	0.79435	1
Rybp	-0.10153	0.074614	1
Zfp809	-0.1017	0.511517	1
Tpcn1	-0.1017	0.647397	1
Auh	-0.10204	0.175522	1
Bach2	-0.10205	0.166065	1
Bmf	-0.10217	0.956779	1
Ttc9c	-0.10223	0.849121	1
Rps6kb2	-0.10226	0.717337	1
Btaf1	-0.10227	0.179823	1
Tgtp2	-0.10249	0.52911	1
Sarnp	-0.10257	0.892493	1
Ugt1a7c	-0.10258	0.624351	1
Pygo2	-0.10265	0.263393	1
Casp8ap2	-0.10278	0.899431	1
Slc35b3	-0.10281	0.618354	1
Tbc1d13	-0.10287	0.394534	1
Med1	-0.10293	0.166875	1
Smim11	-0.10297	0.964133	1
Efr3a	-0.10298	0.144518	1
Gm13212	-0.10319	0.942275	1
Acvr1b	-0.10327	0.884879	1
Sntb1	-0.10328	0.013021	1
Gm47162	-0.10331	0.01125	1
Nf2	-0.10339	0.578255	1
Gm27017	-0.10345	0.542265	1
Hcfc2	-0.10349	0.937112	1
Tmem229b	-0.10353	0.892159	1
Acot11	-0.10357	0.131989	1

Rasa1	-0.10362	0.27436	1
Supt3	-0.10364	0.69035	1
Marf1	-0.10364	0.979028	1
Snrnp48	-0.10368	0.581174	1
Wdr3	-0.10373	0.660789	1
Zfp991	-0.10376	0.132076	1
Syk	-0.10384	0.37182	1
Zfp110	-0.104	0.512034	1
Safb	-0.1041	0.928017	1
Exoc6b	-0.10412	0.498205	1
Spin1	-0.10412	0.522493	1
Oas2	-0.10416	0.917337	1
Psip1	-0.10416	0.484008	1
Cep63	-0.10421	0.630204	1
Brpf3	-0.10433	0.201831	1
Utp23	-0.10448	0.450897	1
Sowahc	-0.10448	0.831334	1
Nelfb	-0.1045	0.813477	1
Frg1	-0.10461	0.432876	1
Pik3r4	-0.10473	0.915806	1
Zfx	-0.10476	0.607306	1
Zfp644	-0.10488	0.806824	1
Zfp945	-0.10494	0.717597	1
Hpse	-0.1051	0.868449	1
Btk	-0.10514	0.887224	1
B430306N(	-0.10528	0.309574	1
Ncstn	-0.10541	0.828568	1
Opa3	-0.1055	0.463813	1
Stam2	-0.1055	0.491867	1
Znrf2	-0.10558	0.783963	1
Isg20	-0.10573	0.325575	1
Acaa2	-0.10574	0.303939	1
Bckdha	-0.10585	0.243641	1
Ticam1	-0.10591	0.520687	1
Nfyc	-0.10593	0.204381	1
Man1b1	-0.10593	0.206104	1
Cep350	-0.10595	0.485373	1
Zfp866	-0.10598	0.955663	1
Rpa1	-0.10599	0.583386	1
4833439L1	-0.10612	0.6793	1
Fam98a	-0.10613	0.832409	1
Gm5617	-0.10617	0.456551	1
Lilra6	-0.10619	0.435064	1
Disp1	-0.10623	0.481294	1
Hoxb4	-0.10627	0.116849	1
Arih1	-0.10646	0.757586	1
Trpc4ap	-0.10649	0.587124	1
Rnf25	-0.10656	0.986697	1

Vps36	-0.10661	0.68411	1
Sp4	-0.1067	0.399296	1
Itpr2	-0.10674	0.983795	1
Prrc2c	-0.10676	0.583402	1
Ezh1	-0.1068	0.74936	1
Wbp2	-0.1068	0.997232	1
Rbm27	-0.1069	0.759593	1
Leng9	-0.10697	0.716167	1
Chmp1b	-0.10698	0.983705	1
Hnrnpf	-0.10698	0.079682	1
Ndufaf3	-0.10699	0.409284	1
Camta2	-0.10707	0.909883	1
Hars2	-0.10711	0.581506	1
Pdcd4	-0.10724	0.22184	1
Chordc1	-0.10734	0.346678	1
Rpl37	-0.10741	0.020587	1
Prcp	-0.10766	0.303873	1
Lin37	-0.10776	0.702615	1
Pqlc3	-0.1078	0.469326	1
Dclre1a	-0.1079	0.654134	1
Mob4	-0.1079	0.958871	1
Tra2b	-0.10798	0.39909	1
Uba52	-0.10813	0.347325	1
Pxk	-0.10818	0.988734	1
Hmbox1	-0.10818	0.656726	1
Ano6	-0.1082	0.468969	1
Skil	-0.1083	0.259261	1
Pih1d1	-0.10835	0.78118	1
Abtb1	-0.10836	0.999105	1
Gpatch2	-0.10841	0.830064	1
Ugdh	-0.10843	0.565788	1
Bcl10	-0.10843	0.116027	1
Map3k7	-0.10846	0.626041	1
Rnf44	-0.10865	0.887937	1
Aldh16a1	-0.10874	0.524971	1
Fgd3	-0.10874	0.953469	1
Mylip	-0.1088	0.717509	1
Akr1b3	-0.10882	0.619224	1
Rbbp7	-0.10892	0.600448	1
Lmbr1l	-0.10895	0.365094	1
Zkscan3	-0.10896	0.155163	1
Ifi213	-0.10904	0.027146	1
Mff	-0.10928	0.938981	1
Tmem167b	-0.10941	0.976903	1
Adss	-0.10946	0.658437	1
Naa10	-0.10948	0.491961	1
Ppp1r35	-0.10955	0.735531	1
Kpna6	-0.10958	0.833404	1

Abr	-0.10959	0.698229	1
Amfr	-0.10961	0.103767	1
Utp14a	-0.10968	0.76264	1
Ubap1	-0.10969	0.332265	1
Twistnb	-0.10976	0.673284	1
Frrs1	-0.10987	0.794876	1
Uxs1	-0.10997	0.991654	1
Hps4	-0.11003	0.715418	1
AW011738	-0.11018	0.032056	1
4930581F2	-0.11019	0.053186	1
Gmpr2	-0.11024	0.452622	1
Gm15964	-0.11025	0.131923	1
Dync1i2	-0.11028	0.339606	1
Tmem59	-0.11033	0.246735	1
Snhg15	-0.11042	0.984773	1
Emilin2	-0.11045	0.375709	1
Rab5b	-0.11048	0.548272	1
Elf2	-0.11051	0.325915	1
Zmym4	-0.11053	0.683103	1
Usp45	-0.11065	0.473421	1
Eif4ebp2	-0.11066	0.411562	1
A730081D0	-0.11073	0.268123	1
Eef1akmt2	-0.11078	0.224428	1
Tgoln1	-0.11084	0.78545	1
Mafg	-0.11109	0.709467	1
Irf2bpl	-0.11113	0.925894	1
Plekhm1	-0.1113	0.840028	1
Stim1	-0.1113	0.388925	1
Faf1	-0.11142	0.127044	1
Tnks2	-0.11142	0.853851	1
Lsm10	-0.11145	0.683201	1
Cyb561a3	-0.11147	0.573769	1
Grcc10	-0.11152	0.259372	1
Rab8b	-0.11159	0.817682	1
Bicra	-0.11162	0.589235	1
Gm15832	-0.11162	0.081166	1
Atp1a1	-0.11188	0.626283	1
D6Wsu163	-0.11194	0.70391	1
Plcl2	-0.11195	0.821257	1
Trim24	-0.11202	0.12267	1
Nelfa	-0.11205	0.606249	1
Entpd7	-0.1122	0.699125	1
Gm3448	-0.11224	0.255426	1
Herpud2	-0.11248	0.45849	1
Dhx40	-0.11272	0.907928	1
Gm16556	-0.11282	0.166484	1
Ttc14	-0.11293	0.575701	1
Peg13	-0.11293	0.006626	1

Zgpat	-0.11297	0.922726	1
Hgs	-0.11298	0.423559	1
Cbx3	-0.113	0.004772	1
Ifi207	-0.11306	0.664153	1
Slc7a1	-0.11319	0.700544	1
Dhx16	-0.1132	0.690915	1
Capns1	-0.11339	0.90522	1
Smndc1	-0.11368	0.385132	1
Mms19	-0.11373	0.820881	1
Rnf20	-0.11386	0.702343	1
Kmt5a	-0.11388	0.330603	1
Gsdmd	-0.11393	0.029004	1
Lsm6	-0.11399	0.728713	1
Zfp607a	-0.11417	0.074401	1
Pitpnc1	-0.11423	0.965646	1
Phkb	-0.11424	0.556916	1
Ikbkg	-0.11429	0.475821	1
Rmnd5b	-0.1143	0.92123	1
Nek7	-0.11435	0.454741	1
Ogfod1	-0.11435	0.108023	1
Brms1	-0.11456	0.865127	1
2610507B1	-0.11463	0.781462	1
Zc3h3	-0.11464	0.618205	1
Fbxl6	-0.11473	0.51313	1
Sf3a1	-0.11474	0.56921	1
Tm9sf2	-0.11481	0.825026	1
Zbtb22	-0.11492	0.395711	1
C1galt1c1	-0.11492	0.905324	1
Dcaf6	-0.11512	0.63892	1
Socs5	-0.1152	0.087043	1
Nbeal2	-0.11521	0.579637	1
BC005537	-0.11524	0.322894	1
Zfp26	-0.11527	0.583971	1
Zfp652	-0.11531	0.24983	1
Zfp524	-0.11534	0.472869	1
Ppp4r2	-0.11548	0.868815	1
Matr3	-0.11549	0.64635	1
Borcs6	-0.1156	0.683691	1
Nfya	-0.11563	0.80504	1
Lrwd1	-0.11587	0.604894	1
Zc3h15	-0.11588	0.65028	1
Oaz2	-0.11599	0.256077	1
Crybg3	-0.11616	0.077954	1
Ppp6r3	-0.11625	0.840732	1
Mapkapk3	-0.11627	0.985028	1
Rrp36	-0.11644	0.484022	1
Zfp335	-0.11649	0.825339	1
Ythdc2	-0.11658	0.767727	1

Lrrc20	-0.1168	0.119413	1
Chkb	-0.11688	0.731371	1
Appl1	-0.11692	0.698785	1
Itgb2	-0.11729	0.037758	1
Ndor1	-0.11737	0.721828	1
Wdr26	-0.11743	0.556547	1
Ralgapb	-0.11744	0.117935	1
Ttc37	-0.1175	0.996556	1
Slc9a8	-0.11759	0.271431	1
Specc1l	-0.1176	0.733056	1
Serf2	-0.11764	0.005786	1
Hira	-0.11777	0.147609	1
Ilrun	-0.11781	0.720097	1
Ints3	-0.11797	0.93979	1
Scaf1	-0.11802	0.51552	1
Adora2a	-0.11808	0.030184	1
1600012H(	-0.11821	0.772744	1
Morc3	-0.11826	0.761887	1
Sap25	-0.11842	0.590965	1
Ltn1	-0.11849	0.301954	1
Rc3h2	-0.11851	0.415845	1
Ralbp1	-0.11853	0.535379	1
Ubqln2	-0.11854	0.574869	1
Prr5l	-0.11883	0.741455	1
Creg1	-0.11891	0.770663	1
Taf5	-0.11892	0.053051	1
Prpf6	-0.11899	0.756094	1
Cwf19l2	-0.11911	0.707688	1
Pigs	-0.11938	0.320136	1
Ppp1r10	-0.1194	0.494476	1
Supt20	-0.11949	0.961872	1
Clk4	-0.11949	0.948496	1
Agl	-0.11951	0.767195	1
Yipf3	-0.1196	0.861629	1
Arfrp1	-0.11962	0.350572	1
Elovl5	-0.11966	0.458064	1
Gga3	-0.11976	0.505818	1
Os9	-0.11987	0.711799	1
Pitpnm1	-0.1199	0.987485	1
Fbxl4	-0.11992	0.214894	1
Fam168b	-0.11996	0.813564	1
Bid	-0.12	0.467294	1
Rela	-0.12003	0.175917	1
Haus8	-0.12034	0.941322	1
Nlrp1b	-0.12035	0.993584	1
Tanc2	-0.12035	0.620251	1
Jak1	-0.12042	0.674981	1
Ankrd11	-0.12053	0.324852	1

Ctr9	-0.12055	0.344192	1
Azin1	-0.12059	0.500684	1
Ctnna1	-0.12063	0.303477	1
Tmem168	-0.12066	0.12209	1
Emc2	-0.12074	0.956624	1
Tmtc2	-0.12079	0.037347	1
Insr	-0.12086	0.482952	1
Pds5b	-0.12089	0.594231	1
Dclre1b	-0.12089	0.146845	1
Tial1	-0.1209	0.865472	1
Zfp398	-0.12102	0.537301	1
Gucd1	-0.12105	0.742755	1
Tti1	-0.12105	0.921101	1
Trappc1	-0.12105	0.825696	1
Asxl2	-0.12107	0.838939	1
Commd4	-0.12116	0.705443	1
Iqsec2	-0.12122	0.106765	1
Atmin	-0.12125	0.795281	1
Cirbp	-0.12126	0.714029	1
Ppp1r18	-0.1213	0.099606	1
Mccc2	-0.1214	0.122096	1
Dcp1a	-0.12147	0.694674	1
Vrk1	-0.12148	0.306569	1
Ddx58	-0.12149	0.658902	1
Luc7l3	-0.1216	0.292653	1
Gxylt1	-0.12161	0.227408	1
Zmiz2	-0.12178	0.80226	1
Dcaf7	-0.12184	0.587452	1
Prickle3	-0.12191	0.184935	1
Nbas	-0.12191	0.146843	1
Tia1	-0.12196	0.318893	1
Cd177	-0.12201	0.21166	1
Smarca5	-0.12208	0.982391	1
Rbm42	-0.12211	0.3897	1
Umad1	-0.12216	0.970622	1
Rgs14	-0.12219	0.79619	1
Dhx15	-0.12227	0.841864	1
4933412E1	-0.12229	0.3656	1
Gpr65	-0.1224	0.558077	1
Map3k15	-0.12253	0.995959	1
Sppl3	-0.12255	0.880183	1
Smad2	-0.12273	0.617019	1
Rab3gap1	-0.12288	0.579529	1
Hdac5	-0.12288	0.181879	1
Mid1ip1	-0.12305	0.966728	1
Gm49797	-0.12312	0.583702	1
Ormdl1	-0.12315	0.406612	1
Psd4	-0.12318	0.821631	1

Chd6	-0.12324	0.995455	1
Gmeb2	-0.12325	0.644593	1
Slu7	-0.12326	0.635074	1
Pdlim2	-0.12342	0.759609	1
Eif3f	-0.12345	0.135758	1
Kat7	-0.12352	0.516919	1
Oas1a	-0.12354	0.641934	1
Cab39	-0.12362	0.444963	1
Zfp871	-0.12362	0.939293	1
Ddx3y	-0.12394	0.751011	1
Wapl	-0.12407	0.9311	1
Ltc4s	-0.12408	0.087915	1
Zfp942	-0.1241	0.996782	1
Btbd7	-0.1241	0.539707	1
Zc3h4	-0.12426	0.845291	1
Susd6	-0.12434	0.382317	1
Tyw1	-0.12435	0.503199	1
Rgs18	-0.12436	0.002335	1
Mrpl1	-0.12442	0.520907	1
Ric8b	-0.12443	0.851154	1
Cnr2	-0.12448	0.803289	1
Uhrf2	-0.1245	0.77274	1
Mtdh	-0.12457	0.10213	1
Rprd2	-0.1246	0.991432	1
Zfr	-0.12462	0.551498	1
Pkn2	-0.12465	0.583443	1
Nsmce4a	-0.12466	0.901333	1
Dusp11	-0.12467	0.937296	1
Agpat3	-0.1247	0.473156	1
Lrp1	-0.12471	0.353872	1
Vcpip1	-0.12481	0.155566	1
Shld1	-0.12488	0.233776	1
Tcof1	-0.12489	0.425783	1
Zfand3	-0.12501	0.737937	1
Cpsf3	-0.12517	0.424882	1
Top2b	-0.12517	0.265551	1
Tfam	-0.12525	0.939138	1
Ppm1d	-0.12539	0.259043	1
Nt5c2	-0.12539	0.663473	1
Arl6ip6	-0.12564	0.404472	1
Arid2	-0.12565	0.184336	1
Setdb2	-0.12577	0.648121	1
Ppp4r3a	-0.1259	0.723622	1
Ramp1	-0.12606	0.587799	1
Cramp1l	-0.12608	0.985528	1
Zfp511	-0.12613	0.216808	1
Swt1	-0.12614	0.204956	1
Mettl3	-0.12617	0.74408	1

Dnajc1	-0.12632	0.611364	1
Fanca	-0.12635	0.920008	1
Rpl35a	-0.12644	0.034089	1
Copb1	-0.12646	0.51501	1
Inip	-0.1265	0.27804	1
Tmem179b	-0.12693	0.316504	1
Slbp	-0.12703	0.949841	1
Tmem243	-0.12703	0.357626	1
Usp48	-0.1271	0.330135	1
Kansl3	-0.12721	0.483531	1
Nap1l1	-0.12744	0.116087	1
Ddx28	-0.12747	0.624484	1
Zfyve26	-0.1275	0.93903	1
Slc38a1	-0.12756	0.792517	1
Srrm2	-0.12759	0.283701	1
Sharpin	-0.12781	0.743353	1
Slc22a15	-0.12791	0.286298	1
Ticam2	-0.12791	0.821806	1
Elac1	-0.12793	0.142419	1
1810026B(	-0.12804	0.665024	1
Uhrf1bp1l	-0.12808	0.804955	1
Myd88	-0.1281	0.50573	1
Nsrp1	-0.12811	0.891393	1
Pan3	-0.12814	0.179365	1
Mocs1	-0.12818	0.248498	1
Kpnb1	-0.12819	0.375758	1
Ube2e3	-0.12835	0.91294	1
Tprgl	-0.12842	0.675298	1
Gtf3c1	-0.12843	0.218348	1
Gbf1	-0.12847	0.890599	1
Brpf1	-0.12851	0.847691	1
2310033P(	-0.12857	0.803376	1
Nlrx1	-0.1287	0.129929	1
Prdm10	-0.12893	0.152995	1
Nfic	-0.12897	0.449344	1
Smg6	-0.12898	0.713438	1
Smc1a	-0.12902	0.44233	1
Mdp1	-0.12926	0.265401	1
Dhx8	-0.1294	0.983486	1
Golm1	-0.12943	0.259215	1
Thoc2	-0.12947	0.586084	1
Fbxw2	-0.12954	0.680997	1
Spast	-0.12957	0.421672	1
Micu2	-0.12962	0.585306	1
Mri1	-0.12965	0.847938	1
Plbd1	-0.12968	0.510048	1
Oip5os1	-0.12974	0.358854	1
Parg	-0.12989	0.614021	1

Sympk	-0.13007	0.262851	1
Bud31	-0.13009	0.831681	1
Araf	-0.13012	0.919325	1
Midn	-0.13028	0.455971	1
Mon2	-0.1305	0.624092	1
Hmgcl	-0.13063	0.289404	1
Pcf11	-0.13066	0.623451	1
Rnf215	-0.13074	0.116849	1
Snhg8	-0.13082	0.510729	1
Tlr8	-0.13084	0.767876	1
Serpinb8	-0.13097	0.124253	1
Nsd1	-0.13112	0.312127	1
Kmt2b	-0.13115	0.472627	1
Evi5	-0.13116	0.951325	1
Afdn	-0.13122	0.860329	1
Dph5	-0.13125	0.081421	1
Dgkz	-0.13131	0.562517	1
Emd	-0.1314	0.527053	1
Plxdc1	-0.13158	0.024733	1
Suz12	-0.1316	0.47668	1
Tpk1	-0.1317	0.139379	1
Slc25a28	-0.13174	0.995728	1
Cpt1a	-0.13187	0.892293	1
Rab33b	-0.1319	0.189412	1
Cab39l	-0.13206	0.82257	1
Nedd4l	-0.13213	0.112799	1
Shoc2	-0.13223	0.932453	1
Ube4a	-0.13226	0.483537	1
Chfr	-0.13233	0.149251	1
Prkag2	-0.13235	0.156034	1
Arfgef1	-0.13238	0.585391	1
Spag9	-0.13242	0.713213	1
Wdr20	-0.13249	0.363828	1
Klf10	-0.13273	0.403386	1
Rufy1	-0.13275	0.991651	1
Ppp6r1	-0.13289	0.82321	1
Prpf8	-0.13324	0.412776	1
Paxbp1	-0.13325	0.941324	1
Rsu1	-0.13327	0.418185	1
Zmym2	-0.13343	0.457401	1
Mbd5	-0.13345	0.963663	1
Cfh	-0.13347	0.121401	1
2610035D:	-0.13349	0.00917	1
Dynlt1b	-0.13355	0.097288	1
Pltp	-0.13359	0.010503	1
Hnrnpr	-0.13373	0.706815	1
Setx	-0.13373	0.94868	1
Gps1	-0.1341	0.802808	1

Ppm1a	-0.13415	0.398359	1
Fyttd1	-0.13433	0.342375	1
Lmbrd1	-0.13434	0.919759	1
Mcm9	-0.13441	0.487327	1
Arntl	-0.13442	0.126673	1
Apc	-0.13453	0.797955	1
Atg4a	-0.13454	0.50646	1
Glyr1	-0.13457	0.42953	1
Spop	-0.13479	0.22207	1
Exoc3	-0.13483	0.98273	1
Ehd4	-0.13512	0.399946	1
Phf1	-0.13514	0.206115	1
Fam98c	-0.13534	0.841798	1
Supt5	-0.13537	0.847056	1
Pqlc2	-0.1354	0.58898	1
Gls	-0.13542	0.755836	1
Med7	-0.13554	0.53171	1
Filip1l	-0.13555	4.69E-07	0.015152
Rps27a	-0.13573	0.003023	1
Ppp4c	-0.13577	0.093647	1
Klhl28	-0.13585	0.394033	1
Rab27a	-0.13628	0.339757	1
Taf13	-0.1363	0.923262	1
Hgf	-0.13632	0.897355	1
Vav1	-0.13637	0.523897	1
Fam117b	-0.13655	0.212048	1
Nemp2	-0.13659	0.004998	1
Vps41	-0.13663	0.068227	1
Tmem50a	-0.13667	0.022282	1
Acyp1	-0.13697	0.102766	1
Immp2l	-0.137	0.64676	1
Ifnar1	-0.1371	0.694759	1
Cnpy3	-0.13714	0.443604	1
Rnpc3	-0.13726	0.891012	1
Lgmn	-0.1374	0.165685	1
Jtb	-0.13743	0.528152	1
Arpc5l	-0.13747	0.422863	1
Ubr1	-0.1376	0.740135	1
Osgin2	-0.13771	0.038478	1
Nek9	-0.13793	0.682409	1
Tomm34	-0.138	0.473958	1
Casp2	-0.13809	0.299493	1
Aagab	-0.13816	0.599401	1
Zfyve27	-0.13833	0.55319	1
Gng5	-0.13847	0.031082	1
Akap8l	-0.13856	0.732054	1
Med30	-0.13859	0.792739	1
Map3k2	-0.13863	0.328124	1

Nsun6	-0.13867	0.838018	1
AI839979	-0.13889	0.003606	1
Gm35154	-0.13901	0.120336	1
Nrbf2	-0.13913	0.912228	1
Eps15l1	-0.13915	0.663047	1
Atrx	-0.13916	0.161494	1
Prpf39	-0.13917	0.978885	1
Cep85l	-0.13924	0.191859	1
3300002I0	-0.13925	0.084436	1
March1	-0.13928	0.076985	1
Hsd17b10	-0.13934	0.271047	1
Chm	-0.13958	0.842068	1
Osbpl2	-0.13966	0.866232	1
Dapp1	-0.13977	0.135715	1
Naa35	-0.13978	0.739214	1
Dyrk1a	-0.13979	0.314473	1
Stn1	-0.13991	0.272	1
Sik1	-0.13992	0.900801	1
Nhlrc2	-0.13995	0.739902	1
Paip1	-0.14018	0.77632	1
Thap3	-0.14022	0.767646	1
Dennd1c	-0.14023	0.941056	1
Mtss1	-0.14024	0.610375	1
Ubl3	-0.14025	0.350448	1
Wdr37	-0.14027	0.238645	1
Gfm2	-0.14036	0.152099	1
Zfp84	-0.14045	0.115943	1
Zfp120	-0.14046	0.274738	1
Mphosph8	-0.14066	0.69915	1
Tmem127	-0.14076	0.493606	1
Rbbp5	-0.14098	0.220699	1
Bfar	-0.14134	0.464588	1
Ubxn2a	-0.14139	0.337893	1
Plekhb2	-0.14149	0.759446	1
Anxa1	-0.14166	0.870984	1
Rbks	-0.1417	0.606763	1
Amz1	-0.14174	0.036301	1
Ccnt1	-0.1418	0.48096	1
Pigx	-0.14187	0.555944	1
Cops4	-0.14214	0.801857	1
Ppp2r5e	-0.14225	0.515785	1
Gripap1	-0.14229	0.050792	1
Ube2b	-0.1423	0.400184	1
Ccdc61	-0.14231	0.037945	1
Oga	-0.14234	0.766565	1
Clasp1	-0.14244	0.63687	1
Nfkbib	-0.14247	0.455645	1
Tmco1	-0.14256	0.602939	1

Ankrd12	-0.14265	0.589959	1
Mpc2	-0.14271	0.366969	1
Gid8	-0.14284	0.988609	1
Bcor	-0.14296	0.857583	1
Stau1	-0.14296	0.928215	1
Hnrnpl	-0.14298	0.333511	1
Snrk	-0.14302	0.354652	1
Rbm10	-0.14332	0.220599	1
Sema4b	-0.14333	0.336996	1
Trappc8	-0.14346	0.786993	1
Upf2	-0.14356	0.305036	1
Zbtb16	-0.14359	0.008585	1
Grap	-0.14371	0.168192	1
Parp4	-0.14371	0.426906	1
Tbc1d8b	-0.14392	0.038493	1
2610037D(	-0.14408	0.381569	1
H2-T23	-0.14417	0.463282	1
Safb2	-0.14419	0.78758	1
6030458C1	-0.1442	0.483678	1
Fchsd2	-0.14429	0.970312	1
Nsmce2	-0.14435	0.895731	1
Mthfd2	-0.1445	0.503701	1
Vamp1	-0.1445	0.109262	1
Gpr35	-0.14461	0.083775	1
Lipe	-0.14463	0.127961	1
Zmat5	-0.14466	0.508399	1
Jup	-0.14467	0.00386	1
Ubtf	-0.14474	0.128524	1
Txlna	-0.14482	0.728458	1
Ccpg1	-0.14483	0.218486	1
Sec24c	-0.14484	0.984107	1
Epb41l2	-0.14492	0.517967	1
Usp33	-0.145	0.662518	1
Sbds	-0.14503	0.246509	1
E130307A1	-0.14504	0.307946	1
Arpc2	-0.14516	0.011418	1
Cyp51	-0.14535	0.301886	1
Lta4h	-0.14548	0.489162	1
Sh3glb1	-0.14561	0.209721	1
Fbrsl1	-0.14586	0.585217	1
Zfp513	-0.14592	0.340346	1
Patz1	-0.14601	0.783387	1
Amd1	-0.14612	0.719247	1
Eed	-0.14652	0.621617	1
Camkmt	-0.14654	0.33706	1
Zfp971	-0.14654	0.872487	1
Rarg	-0.14664	0.027755	1
Cnst	-0.1467	0.059609	1

Zdhhc17	-0.14679	0.663396	1
Rnf111	-0.14683	0.331096	1
Pdpk1	-0.14688	0.803154	1
Slain2	-0.147	0.4669	1
Rpl10-ps3	-0.147	0.734833	1
Kdm4c	-0.14716	0.25485	1
Bicral	-0.14726	0.31099	1
Brip1os	-0.14728	0.645848	1
Vta1	-0.14735	0.346644	1
Oas3	-0.14745	0.896788	1
Cse1l	-0.14747	0.68171	1
Atp2b1	-0.14751	0.207764	1
Tmcc3	-0.14765	0.003294	1
Hip1r	-0.14778	0.108013	1
Gng10	-0.14779	0.332974	1
Slc7a7	-0.14785	0.644613	1
Brwd3	-0.14811	0.506378	1
Tkt	-0.14814	0.060563	1
D230025D	-0.14819	0.513248	1
Lfng	-0.14828	0.580364	1
Zc3h13	-0.14836	0.703025	1
Cep135	-0.1486	0.760781	1
Rbbp6	-0.14863	0.859184	1
Fbxo3	-0.14868	0.481391	1
Tnfrsf1a	-0.14879	0.374176	1
Tbc1d1	-0.14906	0.362736	1
P2ry6	-0.1491	0.529071	1
Ube2h	-0.14919	0.881627	1
St6galnac3	-0.14925	0.001917	1
Pex13	-0.14931	0.296908	1
Rab31	-0.14932	0.018996	1
Bmyc	-0.14935	0.053721	1
Mplkip	-0.14948	0.231816	1
Pnrc2	-0.14971	0.047225	1
Eif3j1	-0.14975	0.859068	1
Xrn2	-0.14983	0.121309	1
Rtf1	-0.14986	0.866355	1
Vamp3	-0.15001	0.787173	1
Zfp790	-0.15008	0.01689	1
Sec62	-0.15021	0.082571	1
Rhbdf2	-0.15022	0.706299	1
Mppe1	-0.15033	0.51674	1
Ensa	-0.1504	0.553009	1
Znrd1	-0.15043	0.191717	1
Ammecr1	-0.15068	0.193918	1
Tsen34	-0.15076	0.991704	1
Nfx1	-0.15088	0.593123	1
Kctd10	-0.15102	0.247489	1

Atxn2l	-0.15102	0.211026	1
Samhd1	-0.1511	0.055983	1
Dram2	-0.15129	0.63293	1
Zmynd11	-0.1513	0.811225	1
Tbc1d9b	-0.15134	0.1442	1
Adgrg5	-0.1514	0.367556	1
Ppp1r2	-0.15159	0.668905	1
Fgfr1op2	-0.1516	0.189553	1
Slc36a3os	-0.15171	0.11938	1
Arid4b	-0.15199	0.570193	1
Ppp1r15a	-0.15201	0.838422	1
Brd1	-0.15226	0.914725	1
Chmp2b	-0.15246	0.334351	1
Gm20658	-0.15246	0.853155	1
Eipr1	-0.15254	0.368221	1
Cnot3	-0.15263	0.478817	1
2810013P(	-0.15265	0.643811	1
Arhgap25	-0.15268	0.845904	1
Nsf	-0.15273	0.356806	1
Wac	-0.15284	0.313299	1
Gm39556	-0.15316	0.207016	1
Glg1	-0.15333	0.792665	1
Golga1	-0.15341	0.199479	1
Adap1	-0.15344	0.570134	1
Kcnj2	-0.15385	1.43E-06	0.046011
Inpp5f	-0.15404	0.844133	1
Nck2	-0.15413	0.022278	1
Ppip5k2	-0.15431	0.602624	1
Rnf2	-0.15434	0.040106	1
Rnf166	-0.1544	0.955923	1
Sgpp1	-0.15443	0.286775	1
Msmo1	-0.1545	0.752004	1
Socs4	-0.15489	0.605454	1
Zfp770	-0.15495	0.001766	1
Socs6	-0.15503	0.815599	1
Asb3	-0.15508	0.485051	1
Pbx3	-0.15516	0.789516	1
Cul3	-0.15524	0.786796	1
Shkbp1	-0.15538	0.216295	1
Bloc1s4	-0.15546	0.9047	1
Cdkn2d	-0.1555	0.269232	1
Gm5431	-0.15563	0.690758	1
Nrbp1	-0.15571	0.234364	1
Il10rb	-0.15577	0.775127	1
Cdc42se2	-0.15578	0.184896	1
Nlrp1a	-0.15598	0.738209	1
Suco	-0.15608	0.739186	1
Dnmt3a	-0.1561	0.979968	1

Cd79b	-0.15622	0.000727	1
Dcaf5	-0.15625	0.550527	1
Chd9	-0.15638	0.173838	1
Pdlim1	-0.15646	0.051314	1
Rcsd1	-0.15653	0.739215	1
Vbp1	-0.15668	0.992209	1
Med27	-0.15671	0.396324	1
Slc16a7	-0.15671	0.069328	1
Kmt2d	-0.15683	0.55034	1
Cpeb4	-0.15686	0.888424	1
N4bp2l2	-0.15688	0.284101	1
Mfsd14a	-0.157	0.66932	1
Tob2	-0.1574	0.768688	1
Gnpnat1	-0.15764	0.067747	1
Cr1l	-0.15802	0.488792	1
R3hdm4	-0.15817	0.116114	1
Rprd1a	-0.15828	0.12102	1
Cog5	-0.15839	0.641669	1
Sik2	-0.15855	0.392045	1
Zmynd8	-0.15893	0.46899	1
Vps16	-0.15906	0.166182	1
Fus	-0.15907	0.177135	1
Gm47754	-0.15932	0.111484	1
Fbxo38	-0.15934	0.47045	1
Syne3	-0.15939	0.017047	1
Pea15a	-0.15947	0.118576	1
Fbxl12	-0.15956	0.857521	1
Brwd1	-0.15989	0.32404	1
Zhx1	-0.15989	0.089395	1
Osbpl9	-0.15993	0.317026	1
Phka2	-0.16	0.721696	1
Spopl	-0.16013	0.943582	1
Oard1	-0.1602	0.814876	1
Hgsnat	-0.16025	0.285419	1
E230029C(	-0.16028	0.997133	1
Pwwp3a	-0.16046	0.009391	1
Nufip2	-0.16053	0.313454	1
Cox20	-0.16071	0.363815	1
Stat1	-0.16122	0.04384	1
Dna2	-0.16143	0.563754	1
Dynlt1f	-0.16172	0.187657	1
Tmem30a	-0.16184	0.563955	1
Dennd4b	-0.16197	0.334303	1
Cep192	-0.16206	0.591094	1
Smcr8	-0.16209	0.071849	1
C1galt1	-0.16219	0.817541	1
Phlpp1	-0.16225	0.171967	1
Tmem230	-0.16236	0.6198	1

Anapc16	-0.1624	0.539579	1
Eif1b	-0.16242	0.199077	1
Zrsr2	-0.16253	0.4214	1
Rapgef6	-0.16258	0.474575	1
Ahcyl2	-0.16278	0.278999	1
Ate1	-0.16286	0.916439	1
Gtf3c2	-0.16298	0.491275	1
Erich1	-0.16307	0.096498	1
Cyp4f13	-0.1632	0.573173	1
Nr1h2	-0.16332	0.653937	1
Gpank1	-0.16336	0.281751	1
Fip1l1	-0.16339	0.289361	1
Gatad2b	-0.1634	0.89761	1
Kin	-0.16341	0.478804	1
Pcsk7	-0.16346	0.811637	1
Ascc3	-0.16352	0.430184	1
Ngdn	-0.16359	0.532753	1
Man1a2	-0.1636	0.881642	1
Pycrl	-0.16362	0.470586	1
Dbnl	-0.16373	0.041216	1
Pgap1	-0.16395	0.64812	1
Nemf	-0.16409	0.463642	1
Eml3	-0.16416	0.101645	1
Cntrl	-0.16435	0.575574	1
Gdpd3	-0.16441	0.216914	1
Nlk	-0.16445	0.88127	1
Poldip3	-0.1645	0.26547	1
Wipf2	-0.16454	0.529057	1
Herc2	-0.16506	0.440049	1
Hnrnpul2	-0.16507	0.087939	1
Pqlc1	-0.16519	0.979505	1
Crybg1	-0.16521	0.858345	1
Ints6l	-0.16522	0.925254	1
Lrp5	-0.16525	0.388373	1
Rhog	-0.16529	0.008047	1
Ptp4a3	-0.16537	0.6058	1
Brd4	-0.16537	0.360405	1
Arpc4	-0.16542	0.007592	1
Mettl9	-0.16544	0.29008	1
Acss2	-0.16575	0.136524	1
Cyb5r4	-0.16579	0.280148	1
Ssr3	-0.16585	0.184527	1
H2-Ke6	-0.16612	0.309583	1
Pknox1	-0.1662	0.088491	1
Ddx23	-0.16621	0.288384	1
Prkn	-0.16621	0.152971	1
Vsir	-0.16627	0.264213	1
Chd3	-0.16666	0.660884	1

Arrdc2	-0.16673	0.017537	1
Ryr1	-0.16703	0.302966	1
Fbxo28	-0.16706	0.305827	1
Gm15564	-0.16707	0.010761	1
Rsrc2	-0.16744	0.247545	1
Gm7072	-0.16747	0.213987	1
Fmnl3	-0.16749	0.481543	1
Senp2	-0.16754	0.408686	1
Traf5	-0.16759	0.495527	1
Tspyl1	-0.16759	0.352343	1
Zfp182	-0.16759	0.085279	1
Tbc1d23	-0.16778	0.310448	1
Atp6v0e	-0.16789	0.017731	1
Zfp467	-0.16792	0.18052	1
Pura	-0.16793	0.380913	1
Myo9a	-0.16809	0.919368	1
Washc4	-0.16812	0.635741	1
Srsf10	-0.16814	0.502736	1
Gm35769	-0.16817	0.451706	1
Chchd2	-0.1682	0.011322	1
Kcnip1	-0.16859	0.033538	1
Arhgap11a	-0.1686	0.347919	1
Prkdc	-0.16866	0.728402	1
Hnrnph1	-0.16868	0.284403	1
Cpsf7	-0.16869	0.295818	1
Parp11	-0.1687	0.406153	1
Ints6	-0.16871	0.489912	1
Plekhg3	-0.16912	0.04566	1
Stx18	-0.16933	0.579333	1
Icam2	-0.16939	0.210913	1
Phip	-0.16953	0.444448	1
Pak2	-0.16961	0.040562	1
Lcor	-0.16966	0.856232	1
Fam43a	-0.16973	0.00817	1
Itgb5	-0.16976	0.02505	1
Fam53c	-0.16991	0.172633	1
Pnn	-0.16994	0.189481	1
Atg10	-0.17003	0.324817	1
Pcif1	-0.17018	0.9714	1
Lrch3	-0.17019	0.71536	1
Mtpap	-0.1702	0.777849	1
Prdm2	-0.17025	0.393076	1
Ifih1	-0.17038	0.631534	1
Sec11c	-0.17041	0.182401	1
Ganc	-0.17049	0.104533	1
Bmpr2	-0.17057	0.511853	1
Arhgef2	-0.17075	0.587767	1
Rnf31	-0.17077	0.879013	1

Eif2a	-0.17104	0.300207	1
Uspl1	-0.17115	0.835246	1
Tlr9	-0.17134	0.1437	1
Sf1	-0.17143	0.190135	1
Kcnab2	-0.17144	0.911977	1
Ing4	-0.17167	0.58254	1
Ankle2	-0.1717	0.6603	1
Cln8	-0.17184	0.235191	1
Abca3	-0.17191	0.161156	1
Gtf2a1	-0.17212	0.529546	1
Atp1a3	-0.17214	0.210947	1
N4bp1	-0.17229	0.999515	1
Sephs2	-0.1724	0.228009	1
Ythdc1	-0.17242	0.973132	1
Dlg1	-0.1725	0.484066	1
Nck1	-0.17265	0.908535	1
Gps2	-0.17266	0.464743	1
Tmem87a	-0.1729	0.84753	1
Rasa2	-0.17308	0.549	1
Anxa11	-0.1731	0.513393	1
Spred2	-0.17312	0.647509	1
Ophn1	-0.17321	0.243983	1
Mob3a	-0.17325	0.453656	1
Ermard	-0.17332	0.191961	1
Ankrd13a	-0.17338	0.272381	1
Fbxw11	-0.17352	0.516154	1
Zfp143	-0.1736	0.263296	1
Pfdn5	-0.1736	0.004709	1
Sin3a	-0.17403	0.86677	1
Ube3a	-0.17417	0.503301	1
Dguok	-0.17421	0.339588	1
Ublcp1	-0.17422	0.867332	1
Abcb7	-0.17427	0.004463	1
Pyroxd1	-0.17451	0.016054	1
Kmt2a	-0.17453	0.077974	1
Cox16	-0.17467	0.712473	1
Slfn5	-0.1747	0.065917	1
Dhrs7b	-0.17475	0.955405	1
Pnkp	-0.17481	0.159797	1
Sh3pxd2a	-0.17483	0.02471	1
Kri1	-0.17483	0.444449	1
Rictor	-0.17486	0.300289	1
Setd1b	-0.17495	0.445696	1
Tram1	-0.17504	0.132947	1
9930021J0	-0.1751	0.139773	1
Tinf2	-0.17513	0.318628	1
Gm9949	-0.17533	0.000634	1
Nxf1	-0.17586	0.766411	1

Hltf	-0.1759	0.263065	1
Sqor	-0.17598	0.581884	1
Svip	-0.17601	0.000723	1
Pdxdc1	-0.17603	0.820953	1
Pde4dip	-0.17625	0.307863	1
Pde4b	-0.17629	0.371397	1
Strada	-0.17648	0.285601	1
Polr2b	-0.17689	0.108354	1
Sptan1	-0.17696	0.227619	1
Gpr155	-0.17717	0.00405	1
Psen1	-0.17721	0.941481	1
Ankra2	-0.17722	0.849615	1
Cdk12	-0.17739	0.639239	1
St6gal1	-0.1777	0.073722	1
P2ry13	-0.17795	0.153438	1
Hivep2	-0.17805	0.160069	1
Gigyf2	-0.17812	0.309651	1
Stab1	-0.17836	3.23E-05	1
Pgghg	-0.17843	0.133751	1
Sp2	-0.17847	0.233051	1
Tspyl3	-0.17896	0.003737	1
Ipcef1	-0.17923	0.506961	1
Rftn1	-0.17928	0.158034	1
Usp34	-0.17932	0.366147	1
Clec2i	-0.17936	0.243954	1
Tsc22d2	-0.17942	0.262989	1
Hmgxb3	-0.17946	0.94215	1
Nfkbid	-0.17947	0.760435	1
Usf3	-0.17955	0.05705	1
R3hdm2	-0.17955	0.689767	1
Slc25a20	-0.17985	0.564334	1
Ubac2	-0.17987	0.340245	1
Nod2	-0.18009	0.854803	1
Gphn	-0.18031	0.002993	1
Micu1	-0.1804	0.548688	1
Maf1	-0.18069	0.276722	1
Armc3	-0.18078	0.000754	1
Zfp950	-0.18108	0.107761	1
Sp3	-0.18113	0.703251	1
2810004N	-0.18117	0.9045	1
Trim21	-0.18145	0.621738	1
Pik3cb	-0.18154	0.721563	1
Smarcd2	-0.18161	0.029645	1
Eif4g2	-0.18178	0.100433	1
Rnf6	-0.18203	0.49139	1
Lsm14a	-0.1821	0.993047	1
Zcrb1	-0.18216	0.271341	1
Med13	-0.18219	0.183355	1

Spic	-0.18233	0.031198	1
Pi4kb	-0.18262	0.408741	1
Stard8	-0.18282	0.96482	1
Cybc1	-0.18295	0.095725	1
Tmem71	-0.18318	0.120439	1
Slc8b1	-0.18397	0.3656	1
Sod1	-0.18403	0.814153	1
Pdha1	-0.18405	0.529603	1
Snx29	-0.1844	0.824776	1
Mpp1	-0.18441	0.862176	1
Ppfibp2	-0.18455	0.000222	1
Heca	-0.18465	0.766843	1
Gm20559	-0.18466	0.044366	1
Mta2	-0.1852	0.073536	1
Lrba	-0.18554	0.059175	1
Ndufaf7	-0.1857	0.042737	1
Nrf1	-0.1858	0.497246	1
Ncoa3	-0.18585	0.033844	1
Fbrs	-0.18587	0.294419	1
Ldlrap1	-0.18609	0.052875	1
Mia3	-0.18646	0.159931	1
Fnta	-0.18697	0.500437	1
Copg1	-0.18719	0.577552	1
Fam133b	-0.18733	0.091366	1
Gon4l	-0.18748	0.433543	1
Creb1	-0.18771	0.740172	1
Smg1	-0.18776	0.1505	1
Rcan1	-0.18778	0.332234	1
Pabpc1	-0.18779	0.024401	1
A530064D(	-0.18781	0.001378	1
Ggct	-0.18783	0.428054	1
Zfp263	-0.18795	0.189612	1
Ifi27l2a	-0.18809	0.267213	1
Scaf4	-0.1881	0.316019	1
Fli1	-0.18837	0.086136	1
Senp1	-0.18864	0.357893	1
Tmpo	-0.18886	0.066925	1
Aqr	-0.18888	0.670688	1
Wls	-0.18911	0.299431	1
Bod1l	-0.18918	0.178737	1
Nup107	-0.18949	0.040901	1
Plpp1	-0.18952	0.090488	1
Arhgap27o	-0.18965	0.002784	1
Paxx	-0.18973	0.038929	1
Galnt10	-0.18978	0.803988	1
Mindy2	-0.18992	0.67161	1
Ralgps2	-0.19004	0.194046	1
Nrm	-0.19019	0.08704	1

Baz2a	-0.19028	0.329683	1
Tmsb10	-0.19031	0.231647	1
Phf12	-0.19038	0.269571	1
Adipor1	-0.19084	0.10336	1
S100a4	-0.19096	0.030862	1
Rock2	-0.19097	0.1666	1
Tmc8	-0.19109	0.002849	1
Gss	-0.19115	0.717232	1
L3mbtl3	-0.1912	0.61443	1
Rab8a	-0.19133	0.07536	1
Maml2	-0.19141	0.618257	1
4833420G1	-0.19144	0.620482	1
Mefv	-0.19157	0.202611	1
Tug1	-0.19158	0.142415	1
Lmbrd2	-0.19158	0.957066	1
Agps	-0.19169	0.887734	1
Ubash3b	-0.19218	0.1432	1
Shc1	-0.19238	0.654589	1
Cdk19	-0.19238	0.891661	1
Plxnc1	-0.19248	0.051405	1
Ahr	-0.1927	0.37029	1
Phc3	-0.19339	0.10159	1
Cdc26	-0.19366	0.344935	1
Gfpt1	-0.19391	0.377681	1
Rassf3	-0.19431	0.888994	1
Herpud1	-0.19439	0.518046	1
Zfp62	-0.19461	0.051899	1
Rbm26	-0.19472	0.115108	1
Triap1	-0.19507	0.144741	1
AW554918	-0.19512	0.588668	1
Irf3	-0.19514	0.437204	1
Jrkl	-0.19548	0.048705	1
Cdipt	-0.19551	0.935246	1
Slc38a2	-0.19553	0.400299	1
Huwe1	-0.19562	0.910801	1
Bbc3	-0.19572	0.054751	1
Fcgr4	-0.19584	0.298308	1
Pdlim5	-0.19589	0.114581	1
Gm17767	-0.19597	0.000588	1
Lman2l	-0.19606	0.779699	1
Serpinb6a	-0.19657	0.566135	1
Scaf8	-0.19657	0.270659	1
Il27	-0.19722	0.935334	1
Serinc3	-0.19724	0.127096	1
Il18	-0.19739	0.195384	1
Dpp8	-0.19739	0.205926	1
Prrc2a	-0.19757	0.408772	1
Osbpl8	-0.19773	0.511688	1

Pygb	-0.19816	0.13786	1
Kdm5c	-0.19852	0.067272	1
Sft2d1	-0.19869	0.154044	1
Usp8	-0.19869	0.328166	1
Arf6	-0.19871	0.242897	1
Anxa7	-0.19902	0.885677	1
Ogfrl1	-0.19938	0.947813	1
Selenot	-0.19948	0.050759	1
Def6	-0.19958	0.209798	1
Purb	-0.19998	0.127505	1
Slc29a1	-0.20008	0.441867	1
Cln3	-0.201	0.242152	1
4930444A1	-0.20107	0.001275	1
Dock10	-0.20112	0.288968	1
Abcd2	-0.20137	0.230341	1
Mark2	-0.20138	0.06006	1
Gpr18	-0.20151	0.501426	1
Hnrnpd	-0.20156	0.017103	1
Zup1	-0.20163	0.13138	1
Ube2r2	-0.20168	0.518915	1
Tle4	-0.20183	0.528234	1
Tspan5	-0.20192	0.373452	1
Kdm7a	-0.20216	0.051324	1
Senp7	-0.20227	0.326212	1
BE692007	-0.2023	0.32614	1
Laptm4a	-0.20241	0.04839	1
Ago2	-0.20243	0.307457	1
Irf9	-0.20243	0.01532	1
Amn1	-0.20255	0.846808	1
Crebl2	-0.20259	4.8E-05	1
Hps5	-0.2027	0.644044	1
Tut7	-0.2028	0.028827	1
Abi1	-0.20282	0.002839	1
Slmap	-0.20284	0.523972	1
Trp53	-0.20292	0.275213	1
Gnb1	-0.203	0.231757	1
Tor1aip1	-0.203	0.011778	1
Sppl2a	-0.20311	0.096908	1
Wdr33	-0.20324	0.068367	1
N4bp2l1	-0.20337	0.210997	1
Nudcd3	-0.20347	0.789642	1
Baz1b	-0.20356	0.160209	1
Ube2w	-0.20363	0.155577	1
Spen	-0.20364	0.357487	1
Phf8	-0.20373	0.083924	1
Ncoa7	-0.20381	0.061331	1
Nlrc4	-0.2039	0.008841	1
Rbm6	-0.20396	0.219986	1

Apaf1	-0.20397	0.52258	1
Prkx	-0.20407	0.086881	1
Mrpl14	-0.20413	0.459626	1
Hpgds	-0.2043	0.114463	1
0610040J0	-0.20438	0.082512	1
Rfx3	-0.20467	0.024077	1
Hivep1	-0.20473	0.726977	1
Trim30b	-0.20477	0.323536	1
Map2k5	-0.20488	0.915176	1
Ccdc69	-0.20502	0.010055	1
Relch	-0.20505	0.173312	1
Atxn7l1	-0.20505	0.364542	1
Prkd3	-0.20516	0.19888	1
Bloc1s2	-0.20542	0.151358	1
Mical1	-0.2057	0.004554	1
Pikfyve	-0.2058	0.151154	1
Pxn	-0.20602	0.008469	1
Map4k3	-0.20637	0.196846	1
Atad2b	-0.20645	0.02471	1
Gpbp1	-0.20655	0.291803	1
Atp6v0a1	-0.2067	0.750115	1
Nsa2	-0.20706	0.018756	1
Abcc4	-0.20714	0.309371	1
Fam13b	-0.20734	0.539095	1
Stim2	-0.20745	0.008428	1
Ccdc171	-0.20765	0.047841	1
Ralgapa1	-0.20794	0.707497	1
Maml1	-0.20795	0.017266	1
Exoc4	-0.20821	0.989238	1
Gm20663	-0.20831	0.016355	1
Capzb	-0.20847	1.62E-05	0.521867
3110082I1	-0.20859	0.200925	1
Gpr137b	-0.20867	0.259827	1
Bdp1	-0.2096	0.811654	1
Nostrin	-0.20961	0.111033	1
Tpp2	-0.2097	0.083556	1
Cdyl	-0.20991	0.354169	1
4632427E1	-0.20993	0.056608	1
Tbc1d5	-0.21053	0.126457	1
Cyb5r1	-0.21057	0.597288	1
Gm32401	-0.2106	0.000124	1
Rtn1	-0.21121	1.62E-06	0.052353
Sptbn1	-0.21126	0.598396	1
Cbfb	-0.21128	0.560407	1
Map4k2	-0.21133	0.341144	1
Cux1	-0.21157	0.170516	1
Aip	-0.2117	0.188045	1
Wwp2	-0.2119	0.090948	1

Unc13d	-0.21193	0.222679	1
Rbm33	-0.21194	0.116101	1
Arid1b	-0.21201	0.104876	1
Ubxn7	-0.21203	0.037853	1
Akt3	-0.2121	0.306875	1
Rpl38	-0.21215	5.41E-05	1
Prkab2	-0.21219	0.005371	1
Cop1	-0.21224	0.147056	1
Pnisr	-0.21244	0.304584	1
Tmx3	-0.21253	0.145182	1
Atg7	-0.21263	0.14399	1
5430427O	-0.21272	0.004977	1
Leng8	-0.21279	0.054559	1
Pofut1	-0.21298	0.665705	1
Rnf168	-0.21303	0.223757	1
Slc12a9	-0.21314	0.42024	1
Sept7	-0.21317	0.063588	1
Zcchc8	-0.21353	0.026149	1
Sp110	-0.21374	0.018326	1
Sos2	-0.21377	0.102336	1
Tmem50b	-0.21382	0.082448	1
4921511I1	-0.21433	0.000376	1
Eapp	-0.21435	0.86171	1
Fyco1	-0.21439	0.470714	1
Cpne1	-0.21445	0.105031	1
Glud1	-0.21467	0.000662	1
H2-D1	-0.2147	0.014689	1
Tnrc6c	-0.21506	0.52342	1
Cbx4	-0.21512	0.025466	1
Nmt1	-0.21514	0.750334	1
Slc4a7	-0.21521	0.780378	1
Taf3	-0.21527	0.080416	1
Puf60	-0.21561	0.341369	1
Kat2b	-0.21587	0.398867	1
Zhx2	-0.21592	6.36E-05	1
Gm37494	-0.21603	0.04693	1
Gabarapl2	-0.21617	0.353644	1
Kmt5b	-0.21619	0.193615	1
Tbc1d32	-0.21622	0.443237	1
Adgre1	-0.21631	0.912721	1
Rsbn1l	-0.21631	0.460766	1
Gm13562	-0.21653	0.072049	1
Trmt112	-0.21677	0.058809	1
Lrrk1	-0.21692	0.12719	1
Scaper	-0.21732	0.967809	1
Rp2	-0.21747	0.045032	1
Mfap3	-0.21773	0.115296	1
Itpr1	-0.21829	0.20489	1

Ap2b1	-0.21846	0.326256	1
Preb	-0.2185	0.012324	1
Adamdec1	-0.21852	0.123395	1
Atp2c1	-0.21878	0.222686	1
Mta3	-0.21894	0.38175	1
Chd1	-0.21904	0.557027	1
Ugp2	-0.21905	0.246411	1
Cdk2ap2	-0.2194	0.010845	1
Hook3	-0.21961	0.174796	1
4930469K1	-0.21962	0.359614	1
Iffo1	-0.21963	0.190593	1
Rmdn1	-0.21973	0.080446	1
Cebpd	-0.21994	0.248376	1
Pld4	-0.22058	0.006685	1
Zfp944	-0.22151	0.040733	1
Akap8	-0.22154	0.462339	1
Cyld	-0.22155	0.035548	1
Zeb2	-0.22156	0.009291	1
Mkln1	-0.22195	0.104654	1
Mapkapk2	-0.22215	0.003475	1
Tnrc6b	-0.22269	0.028806	1
Arhgdib	-0.22315	0.000427	1
Tfe3	-0.22319	0.104347	1
Elmsan1	-0.22327	0.604259	1
Tstd3	-0.22347	0.059009	1
Armc7	-0.22366	0.024599	1
Hdac7	-0.22368	0.107109	1
F630028O1	-0.22377	0.003537	1
Rpl3l	-0.22388	0.055614	1
Arpc5	-0.22408	0.00081	1
Mrpl52	-0.22409	0.007555	1
Snx1	-0.22426	0.035107	1
Tpr	-0.22452	0.013266	1
Dgka	-0.22474	0.001569	1
Rhob	-0.22482	0.853153	1
Ist1	-0.22486	0.121568	1
Ldah	-0.22508	0.020626	1
Traf3ip3	-0.22521	0.046331	1
Rspry1	-0.22533	0.531095	1
Gm10130	-0.22593	0.032869	1
Piezo1	-0.22597	0.254472	1
Gm16120	-0.22607	1.89E-06	0.061095
Rrm2b	-0.22608	0.113705	1
Zranb1	-0.2262	0.158098	1
Gab3	-0.22646	0.022371	1
Tmsb4x	-0.22685	2.5E-08	0.000807
Arhgap9	-0.22733	0.009237	1
Gch1	-0.22743	0.090863	1

Prex1	-0.22748	0.041339	1
Baz1a	-0.22778	0.034318	1
Lrmp	-0.22788	0.618347	1
Kif21b	-0.22791	0.145474	1
Herc1	-0.22808	0.154215	1
Sh3bp2	-0.22812	0.037247	1
Abhd2	-0.22849	0.321119	1
Rsrc1	-0.22906	0.481912	1
9030404E1	-0.22921	7.6E-07	0.024528
Gdi1	-0.22934	0.139844	1
Ntng2	-0.22943	0.268723	1
Trip11	-0.22958	0.13845	1
Etnk1	-0.22976	0.451595	1
Gm4070	-0.22977	0.211115	1
Vegfc	-0.23148	1.29E-08	0.000417
Mettl7a1	-0.23164	0.000341	1
Bola2	-0.23172	0.13798	1
Gtdc1	-0.23221	0.013081	1
Ppp3ca	-0.23229	0.045021	1
Rac2	-0.23291	0.004299	1
Card9	-0.23303	0.081036	1
Stk40	-0.23306	0.017969	1
Tsc22d3	-0.2334	0.048029	1
Smagp	-0.2335	0.001298	1
Ptpn1	-0.23361	0.000512	1
Ccdc180	-0.23362	0.036896	1
Gm11651	-0.23381	1.37E-08	0.000443
Ifit3b	-0.23383	7.36E-05	1
Gm42418	-0.23448	0.037331	1
Far1	-0.23452	0.002256	1
Cdc42ep2	-0.23456	0.430475	1
Bin3	-0.23463	0.11586	1
Mink1	-0.23485	0.568706	1
Capza1	-0.23487	0.002281	1
Stat6	-0.2351	0.167531	1
Ccdc97	-0.23525	0.106946	1
Ppfia4	-0.23537	0.026488	1
Mad1l1	-0.23547	0.299226	1
Zfp52	-0.23548	0.002893	1
Dazap2	-0.23575	7.52E-05	1
Ambra1	-0.23592	0.042054	1
Rab11b	-0.23643	0.14883	1
Phf3	-0.23655	0.935767	1
2310009A(	-0.2369	0.12966	1
Rnf167	-0.23721	0.082502	1
Dap	-0.23769	0.114667	1
Nudt13	-0.23794	0.028919	1
Mbnl1	-0.23797	0.001101	1

Myo1c	-0.23805	0.163648	1
Csf2ra	-0.23815	0.000274	1
Lats2	-0.2382	0.235694	1
Arrb1	-0.23844	0.338662	1
Bloc1s1	-0.2385	0.054582	1
Slc25a38	-0.23891	0.087038	1
Rere	-0.23923	0.21055	1
Pik3cg	-0.23927	0.027397	1
Slc38a9	-0.2394	0.112467	1
Srsf5	-0.23958	0.014685	1
Phyh	-0.23961	0.484088	1
Gabpb2	-0.23963	0.030096	1
Fryl	-0.2398	0.033576	1
Nrd1	-0.23984	0.031947	1
Fndc3a	-0.24032	0.03111	1
Ampd3	-0.24054	0.321615	1
Fut8	-0.24056	0.559189	1
Slc39a13	-0.24059	0.018277	1
Parp8	-0.24066	0.022839	1
Gm37401	-0.24082	0.005498	1
Gm7644	-0.24111	0.000464	1
Tcf4	-0.24303	0.34159	1
Zfand6	-0.24307	0.026732	1
Slc35c2	-0.24314	0.005718	1
Nsd3	-0.24328	0.00237	1
Adamts10	-0.24329	0.144535	1
Tmem156	-0.24334	0.00163	1
Rbm38	-0.24345	0.011456	1
Cd82	-0.24349	0.164352	1
Gab2	-0.2435	0.19848	1
Dmtf1	-0.24353	0.048312	1
Tppp3	-0.24365	0.084216	1
Hnrnph3	-0.24369	0.284064	1
Hmgcs1	-0.2437	0.424455	1
Zfp407	-0.2437	0.549487	1
Ip6k3	-0.24398	1.47E-06	0.04751
Ptger4	-0.24419	0.109069	1
Setd2	-0.24475	0.069518	1
Zbtb7b	-0.24503	0.001874	1
Helz2	-0.24506	0.041216	1
Rchy1	-0.24514	0.720443	1
Lima1	-0.24515	0.047859	1
Sipa1	-0.24518	0.051805	1
Gpsm3	-0.24518	0.000747	1
Vps13c	-0.24531	0.077955	1
Gpr183	-0.24553	0.008473	1
Supt6	-0.24554	0.269508	1
Mapk1	-0.24602	0.110186	1

Cdc40	-0.24618	0.078236	1
Pten	-0.24672	0.077172	1
Mcmbp	-0.2474	0.04455	1
Rbms1	-0.24745	0.015301	1
Itch	-0.24811	0.003734	1
Cdc5l	-0.24839	0.046239	1
Srgap2	-0.2484	0.293994	1
Atxn7	-0.24855	0.060854	1
5031425E2	-0.24856	0.012106	1
Usf1	-0.24857	0.067083	1
Zfp608	-0.24878	0.004248	1
Enox2	-0.24882	0.028618	1
Tmed5	-0.24882	0.023928	1
AC149090.	-0.24897	0.118386	1
Erlin1	-0.24926	0.225655	1
Setbp1	-0.24986	0.000383	1
Macf1	-0.25032	0.00108	1
Erbin	-0.25057	0.027361	1
Arhgap15	-0.25072	0.002742	1
A130014A(	-0.25074	0.000115	1
Clcn3	-0.25088	0.404395	1
H2-T24	-0.2509	0.006071	1
Mrtfa	-0.2509	0.017132	1
Pias1	-0.251	0.018088	1
Phf20l1	-0.2511	0.401934	1
Wrn	-0.25229	0.352943	1
Plekhj1	-0.25273	0.070581	1
Plek	-0.25281	0.027992	1
Rc3h1	-0.2529	0.274284	1
Nckap1l	-0.25331	0.069843	1
Hsd17b11	-0.25343	0.057294	1
Acbd5	-0.2535	0.102839	1
Hnrnpul1	-0.25355	0.018135	1
Zfp869	-0.25356	0.017419	1
Lysmd4	-0.25401	0.080799	1
Msra	-0.25406	0.089686	1
Sgk1	-0.25422	0.012402	1
Rsbn1	-0.25479	0.044149	1
Map3k3	-0.25489	0.071039	1
Mdm4	-0.25489	0.107012	1
Ppm1b	-0.25497	0.153473	1
Camk1d	-0.25527	0.002313	1
Clcn4	-0.25608	0.427525	1
Kdm5a	-0.25609	0.541488	1
Luc7l2	-0.25623	0.005873	1
Map3k11	-0.25633	0.00949	1
BC028528	-0.25653	0.046092	1
Pik3r5	-0.25655	0.0478	1

Zfp445	-0.25659	0.154316	1
Syf2	-0.25667	0.019857	1
Wasf2	-0.2567	0.000981	1
Tmc6	-0.25673	0.000228	1
G6pdx	-0.25674	0.01816	1
Vezf1	-0.25675	0.005441	1
Extl3	-0.25678	0.148047	1
Msl2	-0.25683	0.010147	1
Alpk1	-0.25704	0.169611	1
Atp2a3	-0.25748	0.002992	1
Nav2	-0.25766	0.013495	1
Arl6ip5	-0.2579	0.001419	1
Zfyve16	-0.25851	0.216335	1
Nfkb2	-0.25855	0.213058	1
Capn2	-0.25895	0.011744	1
Cept1	-0.25926	0.194161	1
Ms4a7	-0.25947	0.153132	1
R3hdm1	-0.26074	0.050228	1
Ctdsp1	-0.26084	0.815724	1
Msrb1	-0.26157	0.000546	1
Csk	-0.26159	0.003848	1
Actr10	-0.26179	0.121122	1
Asph	-0.26196	0.001801	1
Cat	-0.26209	0.425439	1
Secisbp2	-0.26243	0.061595	1
Ezr	-0.26252	0.104989	1
Ifngr2	-0.26276	0.00084	1
Gmip	-0.26301	0.000908	1
Phtf2	-0.26317	0.049983	1
Mknk1	-0.26376	0.030692	1
Vps37b	-0.26417	0.027568	1
Klf13	-0.26417	0.001207	1
Rps21	-0.26437	1.26E-06	0.040608
Api5	-0.26451	0.030126	1
Cdk13	-0.26457	0.433054	1
Arrdc1	-0.26473	0.145198	1
Tsc22d4	-0.26507	0.000999	1
Usp15	-0.26553	0.078637	1
Calm1	-0.26569	0.000107	1
Sms	-0.26596	0.087522	1
H2-K1	-0.26606	2.55E-06	0.082313
Dok1	-0.26606	0.134192	1
Gm26877	-0.26646	5.62E-06	0.181503
Dido1	-0.26662	0.013224	1
Dhx36	-0.26665	0.068242	1
A330040F1	-0.26688	0.098853	1
Git2	-0.26711	0.002628	1
Cd83	-0.26726	0.988335	1

Phactr2	-0.26734	0.405602	1
Ccl12	-0.2675	0.049263	1
Taz	-0.26754	0.105717	1
Pum2	-0.26772	0.080636	1
Scaf11	-0.26832	0.687798	1
Papola	-0.26843	0.018406	1
Zeb2os	-0.26848	0.024057	1
Kansl1	-0.26854	0.008963	1
Haao	-0.26926	0.001128	1
Syne1	-0.26937	0.122465	1
Sh3bgrl	-0.26985	0.003321	1
B4galnt1	-0.27096	0.02658	1
Nbn	-0.27107	0.02463	1
Dcakd	-0.27183	0.035492	1
Ostf1	-0.27197	8.97E-05	1
Sec14l1	-0.27206	0.185193	1
Tmem135	-0.2723	0.056168	1
Dusp2	-0.27237	0.10608	1
Cdc42	-0.27238	2.67E-07	0.008615
Stat3	-0.27242	0.003359	1
Ino80d	-0.27244	0.00678	1
Fam214b	-0.27269	0.011105	1
Fam172a	-0.2735	0.084344	1
Smchd1	-0.27419	0.004696	1
Bank1	-0.27459	0.014617	1
Usp47	-0.27469	0.120343	1
Ylpm1	-0.27484	0.056258	1
Phf14	-0.27523	0.016818	1
Evi2a	-0.27558	0.083557	1
Fam126a	-0.27574	0.040813	1
Nop10	-0.27685	0.030604	1
B3galnt2	-0.27706	0.000734	1
Sh3bp1	-0.27746	0.016638	1
Prpf4b	-0.27784	0.070598	1
Kat6a	-0.27827	0.00343	1
Cmpk2	-0.27882	0.01682	1
Pet100	-0.27884	0.029146	1
Msi2	-0.27886	0.273754	1
Sp1	-0.27917	0.177293	1
Btbd9	-0.27934	0.062141	1
E2f2	-0.27966	0.050109	1
Ldlr	-0.27969	0.019497	1
Gvin1	-0.28001	0.031505	1
Frmd8	-0.28028	0.008564	1
Cd44	-0.28068	6.43E-05	1
Dennd4a	-0.2807	0.266193	1
Gm17749	-0.28092	9.6E-08	0.0031
Copg2	-0.2826	0.097644	1

Mier1	-0.28286	0.004128	1
Srsf11	-0.28328	0.026717	1
Anxa2	-0.2835	0.00052	1
Cd47	-0.28381	0.000126	1
Cnot2	-0.28396	0.198716	1
1600010M	-0.28396	0.00281	1
Rnf216	-0.28419	0.073875	1
Zfand5	-0.28488	0.011755	1
C2cd5	-0.28492	0.206959	1
Nrarp	-0.28515	8.18E-07	0.026414
Pros1	-0.2856	0.003813	1
Mia2	-0.28562	0.034222	1
Arid4a	-0.28611	0.000214	1
Rab10	-0.28632	0.002082	1
Trim30d	-0.28637	0.188537	1
Apobr	-0.28701	0.081264	1
Crk	-0.28726	0.101805	1
Srebf2	-0.28745	0.046101	1
Bcl11a	-0.28835	5.02E-06	0.162056
Snx20	-0.28836	0.045256	1
Swap70	-0.28873	0.000376	1
Efcab11	-0.28873	0.027134	1
Gm13919	-0.28876	1.24E-07	0.003996
St3gal6	-0.28877	1.83E-05	0.592337
Zdhhc6	-0.28899	0.005261	1
Klf3	-0.28917	0.019059	1
Lpin2	-0.28919	0.027474	1
Fcer1g	-0.28969	4.32E-05	1
Pik3cd	-0.29014	0.000462	1
Rps6ka1	-0.29017	0.000362	1
Tnfrsf21	-0.29036	0.022226	1
Zfc3h1	-0.29044	0.000852	1
Nt5dc1	-0.29142	0.101771	1
Thbd	-0.29153	1.07E-05	0.344166
8030462N1	-0.29184	0.000303	1
Cbfa2t3	-0.29313	0.001847	1
Magt1	-0.29364	0.002767	1
2210408F2	-0.29366	0.048226	1
Zc3h12d	-0.29386	1.14E-05	0.36934
Tom1	-0.29448	0.015825	1
Tnfrsf13b	-0.2945	0.057258	1
Lilra5	-0.29465	0.042996	1
Tmem38b	-0.29471	0.016397	1
Tnfaip8	-0.29539	0.001197	1
Jun	-0.29568	0.164686	1
Gm2245	-0.29581	0.00771	1
Celf1	-0.29587	0.231711	1
Zfp217	-0.29621	0.009382	1

Degs1	-0.29635	0.000639	1
Trpm2	-0.29649	0.618104	1
Tnrc18	-0.29651	0.00728	1
Itgb7	-0.29664	0.001864	1
Foxk1	-0.29668	0.003128	1
Bmt2	-0.29668	0.024539	1
Vamp5	-0.29728	0.034095	1
Rexo1	-0.29729	0.007595	1
Abca9	-0.29734	0.00561	1
Mdm1	-0.29819	9.1E-07	0.029375
Usp9x	-0.2982	0.013636	1
Sfi1	-0.29827	0.083465	1
Spidr	-0.29898	0.058657	1
Trim12c	-0.29906	0.016231	1
Slc16a10	-0.29922	0.008691	1
Cib1	-0.29934	0.021869	1
Ptgir	-0.2998	0.000161	1
Madd	-0.30002	0.001904	1
Ndel1	-0.30005	0.068275	1
H2afj	-0.30072	0.004674	1
St3gal1	-0.30074	0.000285	1
Lpcat4	-0.30078	0.000933	1
Actr2	-0.30119	1.11E-05	0.358301
Mxi1	-0.30122	0.130207	1
Rnf149	-0.30129	0.004137	1
B4galt5	-0.30164	0.042539	1
Fam168a	-0.3022	0.135226	1
Tasor	-0.30267	0.036422	1
Zeb1	-0.3037	0.000452	1
Samd9l	-0.30372	0.016509	1
Phf21a	-0.30391	0.004737	1
Ccnl2	-0.30408	0.012453	1
Hcls1	-0.30435	0.000249	1
Mcl1	-0.30436	4.83E-05	1
Acot9	-0.30603	0.005809	1
Stxbp6	-0.30666	2.62E-05	0.845464
Cmpk1	-0.30696	0.000903	1
Cyp27a1	-0.30734	0.002609	1
Dennd5a	-0.30748	0.000314	1
Ifnar2	-0.30749	0.012118	1
Clk1	-0.30768	0.001807	1
Ralgapa2	-0.30777	0.061722	1
Sept9	-0.30785	0.001161	1
Kif5b	-0.3079	0.000578	1
Apobec1	-0.30813	0.002902	1
Rabgap1l	-0.30851	0.084629	1
Apba1	-0.30853	0.00333	1
Myl12b	-0.3092	1.4E-07	0.004515

Oxr1	-0.3094	0.004143	1
Mapk14	-0.3097	0.002566	1
Arid1a	-0.30986	0.005053	1
Rps6ka2	-0.31006	1.76E-06	0.056725
Gm32633	-0.3103	6.43E-06	0.207623
Ep300	-0.31056	0.016754	1
Naip2	-0.31062	0.004802	1
Galnt1	-0.3108	0.098095	1
Ythdf3	-0.31096	0.011363	1
Irf5	-0.31159	1.96E-05	0.633403
Dgkd	-0.31182	0.000979	1
Synj1	-0.31231	0.000525	1
Ddx6	-0.31233	0.001547	1
Zcchc7	-0.31253	0.052465	1
Mapkap1	-0.31281	0.020877	1
Zfp638	-0.31304	0.006834	1
Tmem131l	-0.31336	0.00302	1
Ccr2	-0.31338	0.392748	1
Tpm4	-0.31348	0.001334	1
Ngly1	-0.31359	0.107789	1
Tmem51	-0.31456	0.000649	1
Anxa6	-0.31472	0.013914	1
Rnase4	-0.31546	0.968243	1
Itsn2	-0.31567	0.046016	1
Nop53	-0.31597	0.075826	1
Spred1	-0.316	0.278647	1
Prkcd	-0.31601	2.37E-06	0.076438
Polr2a	-0.31629	0.000478	1
Sltm	-0.31655	0.064651	1
Inpp4b	-0.31664	6.14E-05	1
Ddx5	-0.3177	1.95E-06	0.06289
Limd1	-0.31771	0.00153	1
Stx16	-0.31807	0.001129	1
Hes1	-0.31809	4.94E-05	1
Vcl	-0.31947	0.000347	1
Foxo3	-0.31982	0.005378	1
Sdccag8	-0.31993	0.029061	1
Smpdl3a	-0.32	0.000229	1
Tfeb	-0.32054	0.006833	1
Eif4a2	-0.32108	0.01522	1
Sipa1l3	-0.32134	0.008604	1
Lims1	-0.32306	0.000189	1
Pkn1	-0.32484	0.002281	1
Ash1l	-0.32487	0.00192	1
Fam32a	-0.32543	0.000703	1
Zzef1	-0.32655	0.056282	1
Tank	-0.32661	0.025956	1
Cnot6l	-0.32666	0.003044	1

Ckb	-0.32675	0.015612	1
Trim12a	-0.32696	0.042434	1
Sh2b3	-0.32724	0.004184	1
Tiam1	-0.32881	0.190825	1
Ncor1	-0.32887	0.003172	1
Cebpb	-0.3293	0.002069	1
Pid1	-0.3294	0.000103	1
Mef2d	-0.32969	0.01772	1
Rbm39	-0.33006	2.65E-06	0.085455
Ext1	-0.33238	0.107743	1
Ttc17	-0.33264	0.014028	1
Rnf130	-0.33322	0.005348	1
Arsb	-0.3336	0.00164	1
Naga	-0.33362	0.000115	1
Tbc1d2b	-0.33397	0.134733	1
Abcd1	-0.33412	0.034584	1
Stag1	-0.33474	0.000629	1
Ddx3x	-0.33477	0.000446	1
Evl	-0.33479	0.000779	1
Specc1	-0.33495	0.001008	1
Gstm1	-0.33525	0.108602	1
Gsk3b	-0.33564	0.002301	1
Myh9	-0.33637	1.55E-05	0.501375
A630001G1	-0.33655	0.025766	1
Crlf3	-0.33684	0.018567	1
Ggta1	-0.33694	0.00617	1
Fau	-0.33727	4.69E-13	1.51E-08
Birc6	-0.33746	0.003892	1
Cep170	-0.3375	0.000423	1
Epc1	-0.33753	0.008349	1
Krit1	-0.33811	0.012357	1
Mfsd6	-0.33833	4.68E-08	0.001512
Ncoa2	-0.33935	0.096612	1
4833407H1	-0.34004	2E-08	0.000647
Tlr13	-0.34082	0.003002	1
Fes	-0.34162	0.001212	1
Cd300ld3	-0.34163	5.63E-11	1.82E-06
Tspan14	-0.34243	0.000156	1
Pilrb1	-0.34265	0.002098	1
Ebi3	-0.34295	0.006147	1
Rab19	-0.34345	0.000374	1
Cbl	-0.3435	0.001057	1
Tgm3	-0.34468	1.42E-08	0.000458
Kdm3b	-0.34508	0.004385	1
Elf1	-0.34532	0.000203	1
Tmem119	-0.3455	0.089042	1
Gpr34	-0.34578	0.636778	1
Stx11	-0.34654	0.074242	1

Atp8b4	-0.34708	0.002561	1
Sash3	-0.34739	2.58E-05	0.833174
Fcrl1	-0.34786	3.45E-08	0.001115
Nhsl2	-0.34834	0.005358	1
Ppdpf	-0.34849	9.94E-05	1
Ppp4r3b	-0.34888	0.003338	1
Eif1	-0.34941	4.85E-09	0.000156
Itgb1	-0.35053	0.001704	1
Snrpb	-0.35086	4.06E-06	0.130945
Ifit3	-0.35125	0.077896	1
Dpp4	-0.35136	6.14E-07	0.019814
Mecp2	-0.35154	0.002347	1
Trib1	-0.35154	0.08754	1
Gstp1	-0.35217	0.000136	1
Ogt	-0.35252	0.001689	1
Dstyk	-0.35382	0.000224	1
Lbh	-0.35397	0.002527	1
Sgms1	-0.3546	0.010484	1
Nptn	-0.3547	1.85E-05	0.598187
Hspa1b	-0.35478	0.026073	1
Unc119	-0.35478	0.105625	1
Megf9	-0.35498	5.72E-05	1
Zc3hav1	-0.35512	0.000301	1
Glipr1	-0.35515	0.010882	1
Kdm6b	-0.35521	0.008921	1
Strn3	-0.35566	0.205187	1
Cyp4f18	-0.35754	0.000312	1
Gcnt1	-0.35844	0.074913	1
Daglb	-0.3595	0.001177	1
Gramd4	-0.35964	6.3E-05	1
Taldo1	-0.3607	2.58E-05	0.832366
Atf7ip	-0.36087	0.012641	1
Tnrc6a	-0.36115	0.088433	1
Lyst	-0.36158	0.034014	1
Polb	-0.36174	0.000572	1
Dock11	-0.36175	0.003028	1
Usp25	-0.36179	0.050847	1
Inpp1	-0.36296	8.58E-05	1
Padi2	-0.36518	7.66E-08	0.002474
Slc15a4	-0.36539	0.000597	1
Rras	-0.36548	0.001651	1
Rpl37a	-0.36601	5.69E-12	1.84E-07
Lncpint	-0.36659	0.017158	1
Gm5547	-0.367	3.76E-09	0.000121
Cdc14a	-0.36803	0.000408	1
Nipbl	-0.36867	0.000548	1
Nin	-0.36924	0.003573	1
Rassf4	-0.36972	1.13E-05	0.364205

Adssl1	-0.37053	0.000402	1
Crebbp	-0.37077	0.001574	1
Lrrc25	-0.37112	3.23E-07	0.010413
Rgs3	-0.37127	8.78E-05	1
Taok3	-0.37202	5.95E-06	0.191991
Sh3bp5	-0.37204	0.000274	1
Cd37	-0.37241	0.015502	1
Lars2	-0.37267	1.58E-08	0.00051
Ccdc125	-0.3729	0.006235	1
Smad3	-0.37307	0.008314	1
Cap1	-0.3737	3.84E-10	1.24E-05
Rhoq	-0.37377	0.431048	1
Rps29	-0.37379	1.23E-12	3.97E-08
Grk5	-0.37495	0.023132	1
Sept6	-0.37658	3E-05	0.968123
3110021N1	-0.37835	1.31E-07	0.004225
Rhoa	-0.37837	1.77E-10	5.71E-06
Rbpms	-0.37853	0.001629	1
Sort1	-0.37878	0.002841	1
Acap2	-0.37912	4.06E-06	0.131051
Arl4c	-0.38004	0.00636	1
Cmss1	-0.38163	2.83E-10	9.15E-06
Trps1	-0.38193	0.001759	1
Atg4d	-0.38198	4.61E-05	1
Il10ra	-0.38349	1.85E-06	0.059664
Ccnd2	-0.38364	0.671513	1
Stk3	-0.38378	0.007725	1
Wdfy4	-0.38384	0.037932	1
Ptbp3	-0.38401	1.66E-08	0.000534
Tgfbr2	-0.38403	0.00276	1
Nfat5	-0.38438	0.072773	1
Myo18a	-0.38465	0.000165	1
Iqgap1	-0.38477	5.36E-08	0.00173
Birc3	-0.3849	8.67E-05	1
Stag2	-0.38529	0.000398	1
Arhgap17	-0.38585	1.25E-05	0.403493
Actr3	-0.38598	7.47E-11	2.41E-06
Ankhd1	-0.38605	0.000192	1
Rin2	-0.38644	0.000332	1
Acer3	-0.38652	0.000211	1
Map2k1	-0.38735	1.12E-06	0.0361
Rsf1	-0.38792	0.003796	1
Igsf6	-0.38806	3.86E-06	0.124585
Abcc5	-0.38826	0.085705	1
Inpp5d	-0.38838	0.00012	1
Abca7	-0.38871	0.000404	1
St8sia4	-0.38889	0.001205	1
Tgfb1	-0.38895	1.41E-06	0.045509

Bri3bp	-0.38911	8.22E-06	0.265343
Psenen	-0.39061	4.86E-06	0.157051
Baz2b	-0.39105	3.11E-06	0.100251
Havcr2	-0.39113	0.000887	1
Nfatc1	-0.39115	7.74E-05	1
Map3k1	-0.39171	0.000355	1
D1Ertd622	-0.39195	0.000362	1
Tifab	-0.39207	1.86E-08	0.000599
Plac8	-0.39218	1.21E-06	0.039154
9930111J2	-0.39245	5.09E-06	0.16424
Ncor2	-0.39293	0.001051	1
Akap13	-0.39345	7.18E-06	0.231827
Fam49b	-0.39409	1.44E-06	0.046435
Gnb2	-0.39441	1.75E-07	0.005653
Kras	-0.39453	0.000933	1
Pfkfb4	-0.39597	5.06E-05	1
Rnf144a	-0.39874	2.07E-12	6.67E-08
Cmip	-0.40044	0.000414	1
Bach1	-0.40067	4.83E-07	0.01558
Rps6ka3	-0.40155	0.000169	1
Tacc1	-0.40161	9.73E-05	1
Selenop	-0.40177	0.017728	1
Kmt2c	-0.40258	3.22E-05	1
Scarb1	-0.40276	1.29E-06	0.041496
Myadm	-0.40348	0.000288	1
Rps27rt	-0.40379	4.02E-06	0.129782
6430548M	-0.40383	0.000189	1
Lmo4	-0.40431	0.022835	1
Pcnx	-0.40548	2.8E-06	0.090312
Nup210	-0.40554	2.23E-05	0.720204
Ggnbp2	-0.40563	3.21E-06	0.103549
Qk	-0.4063	0.000746	1
Zmiz1	-0.40672	0.001365	1
Ccnd3	-0.40725	4.57E-07	0.014749
Rel	-0.40813	0.000212	1
Gpr132	-0.40986	9.74E-05	1
Fbxl17	-0.41065	0.000581	1
Lrrc8c	-0.4113	0.002493	1
Mrpl33	-0.41301	1.96E-06	0.063281
Mob1a	-0.41327	3.49E-07	0.011273
Alcam	-0.41371	0.001661	1
Irak3	-0.41468	0.008231	1
Nuak2	-0.41479	0.000668	1
Ptp4a2	-0.41528	2.8E-06	0.090383
Ncf2	-0.41548	1.88E-07	0.006073
Eps15	-0.41701	0.000647	1
Cybb	-0.42022	2.62E-11	8.47E-07
Dse	-0.42046	0.171014	1

Tm9sf4	-0.4205	1.89E-05	0.611431
Pafah1b1	-0.42055	1.04E-05	0.336379
Dusp5	-0.42075	2.55E-07	0.008236
I830077J0:	-0.42101	0.001777	1
Ifitm3	-0.42165	2.22E-11	7.16E-07
Cdc42se1	-0.42179	1.98E-06	0.063861
Insyn2b	-0.42235	4.04E-05	1
Ypel3	-0.42325	9.54E-07	0.030802
Usp32	-0.42437	4.46E-05	1
Gpatch2l	-0.42503	0.000233	1
Ahnak	-0.42631	0.251593	1
Ptpn6	-0.42777	1.31E-09	4.24E-05
Ncf4	-0.42816	6.35E-05	1
Rsrp1	-0.42849	1.24E-07	0.004007
Htra2	-0.4288	0.001939	1
Tnip1	-0.42884	0.008722	1
Traf3	-0.42895	0.001934	1
Rara	-0.429	2.64E-06	0.0852
Ptpn22	-0.43006	0.000713	1
Map3k8	-0.43007	0.00066	1
Ddx17	-0.43041	2.93E-05	0.94648
Ier2	-0.43063	1.21E-06	0.03892
Vps13b	-0.43166	8.87E-05	1
Fam111a	-0.43188	1.96E-05	0.633814
Clip1	-0.43194	2.62E-05	0.845817
Nfe2	-0.43345	2.76E-07	0.008906
Gpx1	-0.4336	5.88E-12	1.9E-07
Tmem176a	-0.43378	0.038691	1
Rbm5	-0.43402	1.11E-06	0.035947
Stk4	-0.43515	2.58E-06	0.083424
Scp2	-0.43578	2.27E-08	0.000732
Kpna4	-0.43626	3.8E-05	1
Abcg3	-0.43693	0.043661	1
Tyrobp	-0.43719	2.59E-19	8.35E-15
Taok1	-0.4375	0.000327	1
Slk	-0.438	4.72E-06	0.152299
Plxnb2	-0.43804	1.05E-08	0.000338
Mfsd14b	-0.43848	0.00118	1
Elf4	-0.43901	9.4E-07	0.030336
Nfatc3	-0.43966	0.000483	1
Ms4a4c	-0.44107	9.86E-05	1
Gm48099	-0.44126	0.000509	1
Tcf7l2	-0.44182	0.039165	1
Zfp36l2	-0.44391	5.68E-06	0.183335
Ikbkb	-0.44423	6.68E-09	0.000216
Diaph2	-0.44515	9.5E-05	1
Btg2	-0.44517	0.000319	1
Dgkh	-0.44654	1.05E-05	0.338342

Arhgap39	-0.44692	0.000434	1
Pmaip1	-0.44829	1.08E-05	0.350115
Zfp706	-0.44843	1.86E-06	0.06007
Atf6	-0.45198	8.69E-06	0.280434
Napg	-0.45233	9.5E-06	0.306749
Csgalnact2	-0.45254	5.12E-08	0.001654
Mapk3	-0.45264	3.2E-05	1
2310001H1	-0.45285	2.01E-06	0.064971
Aftph	-0.453	1.48E-05	0.477035
Sh2d1b1	-0.45337	0.00407	1
Cdkn1b	-0.45441	3.64E-05	1
Xylt1	-0.45445	8.5E-08	0.002744
Gm2a	-0.45457	6E-08	0.001936
Stk17b	-0.45568	5.88E-05	1
Kmt2e	-0.45659	9.55E-09	0.000308
Gm12185	-0.45746	6.92E-07	0.02234
Ccdc192	-0.45825	2.79E-09	9.02E-05
Gm34084	-0.45949	0.004611	1
Tmem176b	-0.4596	0.475555	1
Nab1	-0.45982	0.000196	1
Cd9	-0.46005	0.005274	1
Rapgef1	-0.46049	2.07E-06	0.066762
Il16	-0.46258	0.000138	1
Lrrk2	-0.46266	0.000134	1
Ripk2	-0.46283	0.000704	1
Smarca2	-0.46481	0.00017	1
Xiap	-0.46511	6.03E-06	0.194602
Eml6	-0.46553	1.83E-06	0.059204
Akap10	-0.46643	2.52E-05	0.813624
Arhgap27	-0.46921	5.63E-05	1
Il6ra	-0.46956	2.23E-09	7.2E-05
Gm11808	-0.47046	1.48E-08	0.000478
Arhgap4	-0.4709	2.27E-07	0.007321
Son	-0.47126	3.74E-09	0.000121
Nr3c1	-0.47163	1.07E-05	0.34703
Ppp1r12a	-0.47199	8.83E-10	2.85E-05
Prkce	-0.47269	1.35E-06	0.043683
Sf3b1	-0.47279	2.41E-11	7.78E-07
Arhgap45	-0.47309	6.93E-08	0.002238
Gk	-0.47392	0.026247	1
Naip5	-0.47408	2.2E-06	0.071105
Cd300lb	-0.47439	8.41E-08	0.002716
Epsti1	-0.47664	3.56E-10	1.15E-05
Sp100	-0.47703	2.74E-09	8.86E-05
BC035044	-0.47722	5.15E-11	1.66E-06
Ttc7b	-0.47759	2.52E-05	0.815102
Adrb2	-0.4781	0.000246	1
Fry	-0.47955	2.68E-05	0.865888

Susd3	-0.48289	5.3E-08	0.001711
Gnas	-0.48418	6.18E-09	0.0002
Ptpro	-0.48444	3.16E-07	0.010211
Wsb1	-0.48458	2.14E-07	0.006896
Plekhm3	-0.48918	1.4E-06	0.045319
Klhl5	-0.48931	2.98E-08	0.000962
Cdc42ep3	-0.4907	2.61E-09	8.43E-05
Arap1	-0.49252	1.86E-07	0.00602
Tgfbr3	-0.49315	1.82E-12	5.87E-08
Neat1	-0.4934	1.73E-05	0.559677
Ehbp1l1	-0.4938	1.49E-07	0.004817
Sh2d3c	-0.49381	1.7E-07	0.005491
Scnn1a	-0.49597	7.39E-09	0.000238
Cst3	-0.49697	3.65E-16	1.18E-11
Tab2	-0.4972	4.16E-09	0.000134
Foxn2	-0.4983	1.3E-05	0.418864
Pacs1	-0.49859	5.14E-06	0.165794
Lyl1	-0.4988	4.69E-08	0.001514
Zbtb7a	-0.49893	1.33E-07	0.0043
C130050O1	-0.50133	5.08E-06	0.163998
Xdh	-0.50267	6.85E-10	2.21E-05
Tln1	-0.50275	2.74E-13	8.83E-09
Zbtb20	-0.50346	3.09E-05	0.998837
Csf3r	-0.50415	1.04E-05	0.334588
Sorl1	-0.50641	3.79E-06	0.122319
Atg4c	-0.50826	1.22E-07	0.00393
Cd180	-0.50925	2.88E-06	0.09294
Cd48	-0.50975	2.28E-13	7.36E-09
Laptm5	-0.51005	3.03E-22	9.78E-18
Man1a	-0.51045	1.11E-05	0.359023
Mob3b	-0.51127	1.32E-05	0.425513
Pbxip1	-0.51128	5.69E-07	0.018378
Ccdc12	-0.51253	1.29E-09	4.17E-05
Adgre5	-0.51306	7.44E-10	2.4E-05
Aim2	-0.51555	4.23E-06	0.136551
Utrn	-0.5158	6.05E-08	0.001952
Hexb	-0.51582	0.213836	1
Arid3a	-0.51608	2.77E-05	0.894167
Clec2g	-0.51879	2.41E-12	7.8E-08
Tbc1d8	-0.52038	0.000386	1
Cdk17	-0.52153	2.88E-09	9.31E-05
Sipa1l1	-0.52228	4.2E-05	1
Calhm2	-0.5223	4.99E-08	0.001612
Arhgap30	-0.52286	2.47E-11	7.97E-07
2410006H1	-0.5251	1.42E-07	0.004597
Rubcnl	-0.52651	1.45E-07	0.004665
Sirpa	-0.52828	2.22E-12	7.18E-08
Itgax	-0.52971	3E-05	0.968813

Gm35853	-0.53067	2.41E-13	7.79E-09
Camkk2	-0.53255	2.94E-10	9.49E-06
Mtpn	-0.53283	6.52E-12	2.11E-07
Cebpz	-0.53491	0.001098	1
Fgfr2	-0.5359	3.3E-09	0.000106
Traf1	-0.53604	0.002724	1
Gsr	-0.53626	1.85E-09	5.98E-05
Cyp2ab1	-0.53839	2.91E-14	9.41E-10
Stk24	-0.53947	2.15E-07	0.006945
Spi1	-0.54072	2.6E-17	8.38E-13
Lyz1	-0.54092	0.120595	1
Rap1gds1	-0.54248	8.78E-06	0.283609
Limd2	-0.54372	5.98E-08	0.00193
Selplg	-0.54609	1.03E-08	0.000334
Etv6	-0.54629	7.28E-10	2.35E-05
Atp7a	-0.54635	6.79E-05	1
Notch2	-0.54915	1.57E-07	0.00506
Nfkb1	-0.54972	7.62E-09	0.000246
Elmo2	-0.54973	9.19E-07	0.029679
Slc8a1	-0.5526	1.6E-06	0.051698
Arhgap26	-0.55302	3.36E-06	0.10833
Ccdc50	-0.55453	3.71E-07	0.011976
Grk2	-0.55625	6.33E-07	0.02043
Aldh2	-0.55649	1.03E-07	0.003315
Lrrc8d	-0.55838	3.38E-08	0.00109
Pilrb2	-0.55895	1.38E-05	0.444717
Map4k4	-0.5598	3.23E-06	0.104312
Tbpl1	-0.56027	0.000155	1
Lair1	-0.56209	9.12E-06	0.294427
Nrros	-0.56287	1.71E-12	5.53E-08
Slc9a9	-0.56739	1.63E-05	0.526257
Lcp2	-0.56957	4.4E-10	1.42E-05
Ier5	-0.57136	3.88E-08	0.001252
H3f3a	-0.5716	7E-22	2.26E-17
Gpr141	-0.57311	2.64E-09	8.51E-05
Arhgef3	-0.57479	2.34E-06	0.075518
Hps3	-0.57882	9.22E-08	0.002978
Hlcs	-0.58325	4.37E-06	0.141061
Cnn2	-0.58406	1.52E-09	4.91E-05
Rnase6	-0.58443	4.71E-09	0.000152
Adcy7	-0.58739	1.03E-08	0.000332
Asap1	-0.58801	1.98E-09	6.41E-05
Lpar6	-0.58898	1.07E-08	0.000347
Ms4a6b	-0.58978	1.15E-10	3.72E-06
Jarid2	-0.59177	1.17E-13	3.79E-09
Celf2	-0.59272	1.94E-11	6.26E-07
Heg1	-0.59478	1.34E-08	0.000434
Lrrfip1	-0.5953	1.39E-08	0.000449

Sparc	-0.59635	0.206848	1
Marcks	-0.59753	3.74E-09	0.000121
Vav3	-0.59879	2.63E-07	0.008492
Tspan13	-0.59891	3.31E-08	0.001068
Slc12a6	-0.6005	3.58E-07	0.011572
Ptpn12	-0.60139	1.4E-05	0.452569
Plekho2	-0.60398	1.75E-10	5.65E-06
L1cam	-0.60409	3.73E-14	1.2E-09
Ppm1h	-0.60514	1.34E-06	0.043399
Themis2	-0.6057	6.06E-09	0.000196
Eif4g3	-0.60608	1.76E-11	5.69E-07
Lcp1	-0.60657	7.29E-21	2.35E-16
Fabp4	-0.60935	7.09E-12	2.29E-07
Ap1s2	-0.60956	1.91E-11	6.16E-07
Cdk8	-0.61158	5.75E-10	1.86E-05
Ppp2r5c	-0.61169	4.48E-08	0.001445
Herc4	-0.61279	1.3E-09	4.2E-05
Mctp1	-0.61944	7.1E-10	2.29E-05
Gm31814	-0.62056	1E-09	3.23E-05
Klf4	-0.62158	5.57E-08	0.001797
Ifngr1	-0.62184	3.77E-15	1.22E-10
Grap2	-0.62244	4.4E-08	0.00142
Gpcpd1	-0.62283	1.98E-07	0.006402
Smurf2	-0.6256	0.001222	1
Sh3kbp1	-0.62582	5.73E-11	1.85E-06
Dipk1a	-0.62808	8.92E-09	0.000288
Sgk3	-0.62827	1.89E-14	6.12E-10
Ms4a6c	-0.63096	4.04E-19	1.31E-14
Cyp4f16	-0.63109	2.19E-09	7.06E-05
Plcg2	-0.63193	4.01E-15	1.29E-10
P2ry12	-0.63402	0.574125	1
Akna	-0.63566	1.05E-12	3.38E-08
Abhd12	-0.63736	2.2E-11	7.1E-07
Svil	-0.6418	5.73E-06	0.18497
Rasgrp2	-0.64565	1.28E-11	4.13E-07
Ttr	-0.64611	4.31E-09	0.000139
Tpd52	-0.64776	1.39E-11	4.49E-07
Agpat4	-0.65131	2.15E-06	0.069469
St3gal4	-0.6527	2.92E-07	0.009419
Plagl2	-0.65422	1.07E-11	3.45E-07
Cblb	-0.65451	2.89E-05	0.932659
Ccdc88a	-0.65549	4.04E-11	1.3E-06
Il31ra	-0.65867	1.5E-06	0.048441
Rb1	-0.65967	1.27E-06	0.040884
Arhgef37	-0.66384	1.11E-08	0.000359
Klra2	-0.66705	2.4E-15	7.75E-11
Nfe2l2	-0.66886	4.82E-16	1.56E-11
Lyn	-0.67068	1.49E-22	4.8E-18

Tiam2	-0.6709	3.3E-10	1.07E-05
Rasgrp4	-0.67167	8.91E-15	2.88E-10
Nod1	-0.67201	1.11E-07	0.003581
Anp32a	-0.67201	7.14E-15	2.31E-10
Tet2	-0.67299	4.38E-08	0.001413
Ttc7	-0.68216	5.63E-14	1.82E-09
Rap1b	-0.68255	1.7E-12	5.47E-08
Acss1	-0.68541	9.34E-17	3.02E-12
Foxp1	-0.6877	6.59E-13	2.13E-08
Nav1	-0.69273	6.55E-14	2.11E-09
Ccm2	-0.69667	1.64E-07	0.005295
Rps27	-0.69912	3.28E-28	1.06E-23
Fgd4	-0.70104	2.71E-09	8.75E-05
Tgfbr1	-0.70172	6.8E-08	0.002194
Raf1	-0.7034	2.27E-11	7.32E-07
Smc6	-0.70532	6.12E-08	0.001976
Mirt1	-0.70781	9.57E-16	3.09E-11
Tmem131	-0.71052	1.28E-11	4.13E-07
Gcnt2	-0.71225	9.42E-14	3.04E-09
Smpdl3b	-0.71419	9E-06	0.290656
Metrnl	-0.71734	6.18E-15	1.99E-10
Ppp1cb	-0.71744	1.52E-11	4.9E-07
Rasa3	-0.71851	6.72E-15	2.17E-10
Tmem164	-0.71975	1.48E-12	4.77E-08
Slc46a3	-0.72205	2.52E-19	8.12E-15
Gm21188	-0.72289	5.53E-17	1.79E-12
Vti1a	-0.72632	1.61E-12	5.21E-08
Ifitm2	-0.72709	2.28E-13	7.36E-09
Dusp6	-0.7318	8.43E-12	2.72E-07
Itgav	-0.73231	3.41E-10	1.1E-05
Arl5c	-0.73411	1.31E-11	4.22E-07
Naip6	-0.73434	5.68E-10	1.84E-05
Ppp2r5a	-0.73475	1.21E-16	3.92E-12
Gsap	-0.73665	7.08E-10	2.29E-05
Tlr7	-0.73795	1.08E-12	3.49E-08
Stat5b	-0.74347	5.16E-11	1.67E-06
Sirpb1b	-0.7445	2.08E-14	6.73E-10
Ywhaz	-0.74687	5.86E-26	1.89E-21
Otulinl	-0.74875	4.99E-19	1.61E-14
Pecam1	-0.74984	3.17E-10	1.02E-05
Flna	-0.75063	1.37E-20	4.43E-16
Nfam1	-0.75145	1.7E-22	5.48E-18
Msn	-0.75227	7.73E-25	2.5E-20
Gm9733	-0.75381	1.42E-05	0.458003
Cdk14	-0.75515	1.56E-12	5.05E-08
F13a1	-0.75876	0.595464	1
Fyn	-0.76013	1.78E-09	5.75E-05
Add3	-0.76088	8.16E-14	2.63E-09

Trem3	-0.76143	2.45E-11	7.9E-07
Rassf5	-0.76337	4.91E-14	1.59E-09
Mef2a	-0.76514	8.24E-17	2.66E-12
Csf1r	-0.76975	3.17E-20	1.02E-15
Nupr1	-0.77176	1.69E-12	5.45E-08
Dok3	-0.77188	3.21E-16	1.04E-11
Dock8	-0.77798	2.69E-21	8.69E-17
Elmo1	-0.77899	2.6E-16	8.4E-12
Fmnl1	-0.78105	1.38E-23	4.44E-19
Dgkg	-0.78393	9.65E-18	3.11E-13
Lsp1	-0.7874	6.84E-27	2.21E-22
Dnah12	-0.78871	3.2E-15	1.03E-10
Sirpb1a	-0.7892	1.96E-10	6.32E-06
Runx2	-0.79115	1.12E-15	3.62E-11
Map3k14	-0.79159	6.29E-15	2.03E-10
Clec4a1	-0.79468	1.55E-18	5.02E-14
Gm5150	-0.79504	1.45E-11	4.69E-07
Hip1	-0.80381	1.99E-10	6.41E-06
Napsa	-0.80959	2.18E-24	7.05E-20
Gm36161	-0.81009	2.82E-17	9.12E-13
Ltb	-0.82933	2.43E-11	7.85E-07
Ighm	-0.83057	5.51E-17	1.78E-12
Arhgef1	-0.83492	7.33E-21	2.37E-16
Nabp1	-0.83569	1.96E-14	6.34E-10
Tmcc1	-0.83584	4.22E-13	1.36E-08
Cd300c2	-0.83585	3.74E-15	1.21E-10
Clec4a3	-0.83711	8.93E-17	2.88E-12
Dleu2	-0.8458	5.18E-20	1.67E-15
Prr13	-0.85378	3.68E-19	1.19E-14
Rgs2	-0.85468	6.1E-16	1.97E-11
Id3	-0.8553	1.35E-17	4.36E-13
Clec2d	-0.86192	2.8E-17	9.03E-13
Fam107b	-0.86279	5.72E-24	1.85E-19
Gm26740	-0.87096	7.34E-20	2.37E-15
Malat1	-0.8759	2.79E-23	9E-19
Map3k5	-0.87862	8.71E-16	2.81E-11
Nkain2	-0.88321	4.37E-29	1.41E-24
Mef2c	-0.88449	2.94E-15	9.5E-11
Itga4	-0.88458	1.83E-17	5.92E-13
Mir142hg	-0.88612	7.32E-24	2.36E-19
Coro1a	-0.88703	2.62E-38	8.46E-34
Zfp710	-0.88752	8.29E-20	2.68E-15
Gm19951	-0.89014	2.43E-16	7.84E-12
Ccdc88c	-0.8903	2.49E-18	8.05E-14
Ikzf1	-0.89322	7.92E-17	2.56E-12
Bin2	-0.90201	1.41E-23	4.55E-19
Hfe	-0.90202	5.92E-16	1.91E-11
Fam129a	-0.90858	2.14E-19	6.91E-15

Stk38	-0.91672	1.99E-25	6.43E-21
Hck	-0.92346	8.77E-25	2.83E-20
Nxpe4	-0.93278	4.41E-24	1.42E-19
Skint3	-0.93482	1.19E-15	3.84E-11
Camk2d	-0.93907	1.78E-18	5.75E-14
Ptprc	-0.94995	4.74E-33	1.53E-28
Nfkbiz	-0.95325	1.8E-12	5.81E-08
Sema4d	-0.96043	7.18E-23	2.32E-18
Serpinb2	-0.96339	7.18E-23	2.32E-18
Arhgef10l	-0.9642	1.25E-21	4.04E-17
Prkcb	-0.96538	1.95E-18	6.29E-14
Cyth3	-0.96893	3.09E-17	9.96E-13
Krt80	-0.97187	9.04E-17	2.92E-12
Rap1a	-0.97846	1.78E-30	5.73E-26
Pip4k2a	-0.98232	1.99E-22	6.41E-18
Myo1f	-0.99971	1.97E-33	6.38E-29
Ptk2b	-1.00172	1.08E-20	3.48E-16
Spata13	-1.00891	4.6E-16	1.48E-11
Dock2	-1.01103	1.77E-27	5.72E-23
Foxn3	-1.01208	1.48E-18	4.79E-14
Ms4a4a	-1.01578	2.18E-16	7.05E-12
Slc44a2	-1.01626	2.54E-29	8.19E-25
Pik3ap1	-1.01729	3.34E-20	1.08E-15
Maml3	-1.02775	2.74E-17	8.85E-13
Tnfrsf1b	-1.03809	7.31E-26	2.36E-21
Ear2	-1.04483	5.21E-11	1.68E-06
Dock5	-1.04836	5.83E-20	1.88E-15
Ripor2	-1.0496	8.96E-12	2.89E-07
Samsn1	-1.05546	9.16E-23	2.96E-18
Ceacam1	-1.05701	1.23E-22	3.96E-18
Zfyve9	-1.05902	1.62E-23	5.23E-19
Lst1	-1.0627	1.69E-35	5.46E-31
S1pr5	-1.06466	5.88E-24	1.9E-19
Ankrd44	-1.0666	2.11E-23	6.81E-19
Fgr	-1.07056	1.92E-27	6.2E-23
Bcl6	-1.07857	1E-21	3.23E-17
Mbp	-1.07982	5.02E-22	1.62E-17
Klf2	-1.08464	9.21E-24	2.97E-19
Pag1	-1.10772	3.18E-12	1.03E-07
Cd244a	-1.1143	3.73E-28	1.2E-23
Spn	-1.12777	7.98E-19	2.58E-14
Prkch	-1.13812	1.02E-24	3.31E-20
Sat1	-1.1441	5.36E-23	1.73E-18
Ifitm6	-1.1468	1.71E-26	5.53E-22
Nadk	-1.1473	3.68E-34	1.19E-29
Ptprj	-1.15706	2.86E-23	9.23E-19
Ldlrad3	-1.1606	4.62E-27	1.49E-22
Pparg	-1.16734	1.02E-23	3.28E-19

Cd300a	-1.17538	2.19E-34	7.07E-30
Myo1g	-1.20861	6.11E-27	1.97E-22
Fyb	-1.21373	1.88E-36	6.06E-32
Ssh2	-1.22099	3.05E-28	9.84E-24
Abi3	-1.22647	8.43E-31	2.72E-26
Apbb1ip	-1.23368	9.49E-35	3.06E-30
Fam49a	-1.24109	3.3E-27	1.07E-22
Il17ra	-1.24203	8.46E-37	2.73E-32
Ptpre	-1.24842	9.36E-26	3.02E-21
Gm15987	-1.26678	1.57E-30	5.07E-26
Cytip	-1.29882	2.05E-33	6.61E-29
Pot1b	-1.30744	5.85E-24	1.89E-19
Cyfip2	-1.34281	2.25E-18	7.27E-14
Itgal	-1.34611	1.88E-30	6.08E-26
Treml4	-1.34634	1.04E-23	3.36E-19
Slc12a2	-1.37068	9.81E-27	3.17E-22
Stap1	-1.40906	2.44E-38	7.87E-34
Cd36	-1.43029	6.15E-17	1.99E-12
Rap1gap2	-1.4423	2.61E-19	8.41E-15
Cd300e	-1.45568	2.21E-26	7.12E-22
Grk3	-1.48962	1.04E-28	3.35E-24
Dusp16	-1.49659	1.52E-17	4.91E-13
Nedd9	-1.51741	8.78E-27	2.84E-22
Stk10	-1.5274	2.7E-37	8.73E-33
Cd300ld	-1.54779	3.82E-23	1.23E-18
Gngt2	-1.6334	2.11E-31	6.8E-27
Pglyrp1	-1.65274	1.8E-27	5.82E-23
Eno3	-1.68341	9.97E-27	3.22E-22
Bcl2	-1.69121	1.53E-20	4.93E-16
Cx3cr1	-1.72454	4.64E-42	1.5E-37
Adgre4	-1.79619	5.91E-30	1.91E-25
Nr4a1	-1.8488	9.41E-39	3.04E-34
Ace	-1.89424	1.08E-33	3.49E-29
Fah	-2.07008	6.64E-65	2.14E-60
Pou2f2	-2.20573	1.89E-50	6.11E-46

SUPPLEMENTAL TABLE 3. Differentially Regulated Genes in GVHD versus BM Microglia

Gene	avg_log2FC	p_val	p_val_adj
Cd74	4.631676	0	0
H2-Ab1	3.315752	0	0
H2-Aa	3.308687	0	0
H2-Eb1	3.032956	0	0
Apoe	2.954148	2.2E-259	7E-255
Ccl2	2.36626	4.1E-221	1.3E-216
Cxcl10	2.245663	7.3E-80	2.36E-75
Cxcl9	2.115001	5.41E-76	1.75E-71
Ifitm3	2.013149	7E-299	2.3E-294
Ccl5	1.985545	1.64E-77	5.31E-73
Gbp2	1.962679	5.9E-297	1.9E-292
Ccl7	1.883627	9.5E-147	3.1E-142
Ccl12	1.875255	4.9E-144	1.6E-139
Ly6a	1.699295	2.9E-169	9.4E-165
H2-Q7	1.680514	2.9E-280	9.5E-276
Cd300lf	1.672374	2.9E-289	9.4E-285
H2-K1	1.669221	0	0
Iigp1	1.627557	5.8E-148	1.9E-143
H2-T23	1.500082	6.3E-291	2E-286
Fcgr4	1.4871	3.9E-248	1.2E-243
H2-Q4	1.469189	1.2E-247	3.8E-243
Stat1	1.460111	2.6E-224	8.5E-220
Gbp7	1.450896	3.6E-246	1.2E-241
Ccl4	1.446982	5.46E-64	1.76E-59
H2-D1	1.436948	0	0
Fgl2	1.412627	2.8E-259	9.1E-255
Gbp5	1.411745	1.5E-175	5E-171
Ifi27l2a	1.400547	5.3E-161	1.7E-156
Ddit4	1.391013	2.42E-46	7.82E-42
Hcar2	1.379885	1.8E-211	5.9E-207
Cd274	1.371381	1.4E-164	4.5E-160
Lgals3bp	1.368695	8.6E-251	2.8E-246
H2-Q6	1.367432	1.7E-218	5.3E-214
Cd52	1.301768	6.2E-199	2E-194
Il2rg	1.29776	2.8E-134	8.9E-130
Ifi207	1.294544	9.1E-210	2.9E-205
Bst2	1.289813	3.8E-197	1.2E-192
Oasl2	1.279411	2E-194	6.6E-190
Cst7	1.271549	6.3E-114	2E-109
Zbp1	1.263794	5.7E-235	1.8E-230
Ifi30	1.254503	1.2E-202	3.8E-198
Isg15	1.245352	7.1E-149	2.3E-144
Ifi204	1.235391	3.1E-217	1E-212
Irf1	1.229177	2E-125	6.4E-121

Ctsc	1.196438	5.8E-199	1.9E-194
Nlrc5	1.183851	1.3E-195	4.2E-191
Lilrb4a	1.183265	6.4E-111	2.1E-106
Cybb	1.170243	2E-140	6.6E-136
C4b	1.168057	1.1E-188	3.6E-184
Gm4951	1.152232	2.7E-161	8.6E-157
Srgn	1.141687	1.1E-145	3.6E-141
Rpl32	1.137283	4E-248	1.3E-243
Rps20	1.122064	1.3E-257	4.1E-253
B2m	1.119388	3.1E-297	1E-292
Tap1	1.117568	1.1E-183	3.5E-179
Tspo	1.1104	1.1E-139	3.7E-135
Rpsa	1.095168	1.7E-219	5.4E-215
Ccl3	1.064149	2.51E-30	8.11E-26
Rnf213	1.055791	8.5E-123	2.7E-118
Irf7	1.048852	1.9E-151	6.3E-147
AW11201C	1.048045	3.5E-120	1.1E-115
Fkbp5	1.040891	4.9E-100	1.6E-95
Samhd1	1.040362	2.6E-119	8.3E-115
Clec2d	1.026459	4.9E-143	1.6E-138
Rps2	1.026277	4.8E-209	1.5E-204
Cd36	1.010312	6.2E-151	2E-146
Gbp4	1.009687	9.1E-170	2.9E-165
Stat2	0.990243	3.4E-148	1.1E-143
Spint1	0.980676	2.4E-152	7.7E-148
Rps18	0.974699	9.5E-183	3.1E-178
Ifit2	0.973332	7.68E-97	2.48E-92
Slfn2	0.97086	1.3E-138	4.2E-134
Fth1	0.955782	1.4E-185	4.4E-181
Cebpb	0.953734	1.7E-125	5.6E-121
Plaur	0.953622	2.72E-84	8.77E-80
Psme1	0.952682	1.8E-154	5.8E-150
Axl	0.952592	5.5E-140	1.8E-135
Irgm1	0.944915	6.6E-127	2.1E-122
AU020206	0.939862	6.1E-128	2E-123
Nfkbia	0.933597	4.19E-75	1.35E-70
Rps12	0.931308	8.2E-192	2.6E-187
Arhgap15	0.927296	1.83E-32	5.92E-28
Lyz2	0.919916	2.5E-126	8.2E-122
Rps24	0.919428	9.3E-211	3E-206
Rplp0	0.917267	1.7E-184	5.5E-180
Il18bp	0.912049	1.2E-168	4E-164
Gbp8	0.900554	7.9E-170	2.6E-165
Rps5	0.895888	4.7E-184	1.5E-179
Tnf	0.89455	1.54E-43	4.97E-39
Gbp6	0.886408	7.7E-138	2.5E-133
Sp100	0.876176	4.8E-128	1.5E-123
Rps7	0.875812	3.4E-179	1.1E-174

mt-Atp8	0.872948	1.9E-147	6E-143
Ms4a6c	0.86972	1.4E-76	4.52E-72
Rpl19	0.863279	2.3E-185	7.3E-181
Cxcl16	0.857896	1.02E-69	3.29E-65
Rpl10a	0.854533	6.5E-166	2.1E-161
Fxyd5	0.847605	3.5E-104	1.1E-99
Il15ra	0.845558	9.3E-130	3E-125
Psap	0.834524	1.8E-228	5.9E-224
Rps11	0.833966	6.9E-177	2.2E-172
H2-DMa	0.833954	2.5E-116	8.2E-112
Pim1	0.822244	1.5E-123	4.9E-119
Csf1	0.813464	6.65E-83	2.15E-78
Gbp9	0.810874	4E-114	1.3E-109
Cd83	0.807978	3.76E-48	1.21E-43
H2-T22	0.804287	1.4E-107	4.7E-103
Rpl13	0.800608	4.3E-184	1.4E-179
Rps26	0.797089	3.7E-132	1.2E-127
Rpl27a	0.796682	4.8E-162	1.6E-157
Socs1	0.795728	2.61E-86	8.43E-82
Psmb8	0.7929	1.9E-108	6.2E-104
Prdx5	0.787641	1.3E-88	4.21E-84
Rps8	0.786649	2E-160	6.6E-156
Rpl12	0.783156	8.7E-126	2.8E-121
Rps15a	0.781702	8.2E-155	2.6E-150
Rpl23	0.778918	2.5E-152	7.9E-148
Rpl17	0.777323	6.1E-142	2E-137
Rpl14	0.768931	4.6E-113	1.5E-108
Ifit3	0.764504	1.94E-90	6.25E-86
mt-Co1	0.761493	2.3E-136	7.5E-132
Rps27a	0.759381	8E-164	2.6E-159
Rpl24	0.758691	1.4E-124	4.6E-120
Rps13	0.755327	4.2E-140	1.4E-135
Arid5b	0.740637	3.54E-44	1.14E-39
Ifi211	0.740093	1.5E-125	4.7E-121
Psme2	0.738871	5.45E-87	1.76E-82
Ucp2	0.737073	4.14E-68	1.34E-63
Rps16	0.735663	4.7E-137	1.5E-132
Rtp4	0.735518	5.54E-90	1.79E-85
Ccdc86	0.734403	7.7E-100	2.47E-95
Atp8a1	0.730706	1.41E-86	4.57E-82
Ctss	0.726126	6E-221	1.9E-216
Rpl35	0.725271	4E-118	1.3E-113
Rack1	0.723405	7.5E-107	2.4E-102
Rps6	0.721232	1.25E-95	4.05E-91
Rps19	0.719262	2.7E-129	8.8E-125
Gas5	0.71803	1.52E-87	4.9E-83
Rps10	0.716517	2.5E-138	8.2E-134
Tor3a	0.713377	7.53E-83	2.43E-78

Vcam1	0.710246	8.44E-82	2.72E-77
Rbm3	0.710211	4.37E-93	1.41E-88
Bcl2a1a	0.707149	6.17E-90	1.99E-85
Rpl26	0.705508	3.1E-121	1E-116
Parp14	0.704187	6.98E-70	2.25E-65
Tpt1	0.701956	2.4E-135	7.9E-131
Igtp	0.701926	7.82E-64	2.53E-59
Rpl28	0.701777	6.5E-118	2.1E-113
Nme2	0.7013	1.43E-84	4.62E-80
Bcl2a1b	0.698919	2.17E-76	7E-72
Slfn5	0.695787	5.03E-85	1.62E-80
Rpl18a	0.695488	1.1E-137	3.5E-133
Siglecf	0.695396	3.73E-67	1.2E-62
Ly86	0.693107	6.3E-121	2E-116
mt-Co2	0.6931	3.2E-112	1E-107
Bcl2a1d	0.689157	6.3E-103	2.02E-98
Rpl22l1	0.688197	1.76E-77	5.67E-73
Gbp3	0.684802	7.7E-114	2.5E-109
Id2	0.684458	2.1E-57	6.78E-53
Ccnd3	0.683708	1.13E-32	3.64E-28
Lap3	0.683495	1.51E-72	4.87E-68
Pkm	0.678331	3.89E-50	1.26E-45
Rpl27	0.677376	2.1E-100	6.63E-96
Rpl29	0.676322	1.3E-104	4.3E-100
Eif2ak2	0.673275	2.27E-74	7.34E-70
Herc6	0.672027	3E-53	9.68E-49
Mif	0.672007	2.75E-43	8.87E-39
Slamf8	0.667789	1.7E-84	5.48E-80
Rpl11	0.666305	1.7E-121	5.6E-117
Rpl15	0.665657	6.8E-102	2.2E-97
Sgip1	0.664556	8.02E-77	2.59E-72
Socs2	0.662734	1.1E-116	3.6E-112
Rpl13a	0.659035	2E-100	6.44E-96
Ier3	0.658685	2.97E-29	9.59E-25
Ifi213	0.655639	1.35E-86	4.36E-82
Rps23	0.652719	1.1E-113	3.7E-109
Xaf1	0.651004	1.11E-88	3.57E-84
Il1b	0.648757	1.16E-32	3.76E-28
Rplp2	0.6466	3.4E-103	1.08E-98
Ly6e	0.645646	8.5E-125	2.7E-120
Rpl37	0.645536	3E-112	9.5E-108
Il1a	0.641159	2.67E-31	8.61E-27
Phf11b	0.63923	2.01E-72	6.49E-68
Dna2	0.638955	2.95E-95	9.53E-91
Csf2rb	0.635706	3.51E-60	1.13E-55
Slc11a1	0.6337	4.96E-71	1.6E-66
Rpl9	0.629875	5.4E-103	1.75E-98
Npc2	0.629181	7.2E-109	2.3E-104

Oas1a	0.625228	5.9E-74	1.91E-69
Rpl39	0.624107	2.2E-105	7.2E-101
Selenow	0.624022	3.54E-72	1.14E-67
Rpl36	0.623959	4.04E-92	1.3E-87
Naaa	0.623671	2.15E-62	6.94E-58
Oasl1	0.622786	3.43E-79	1.11E-74
Rps4x	0.622494	3.8E-107	1.2E-102
Rpl3	0.620644	2.2E-106	7.2E-102
Tgtp2	0.615951	8.4E-100	2.7E-95
Trim30a	0.612562	4.23E-66	1.37E-61
Rpl41	0.611855	2.4E-100	7.73E-96
Rpl6	0.611618	1.7E-104	5.5E-100
H2-DMb1	0.610001	9.09E-35	2.93E-30
Ifi206	0.608203	4.56E-93	1.47E-88
mt-Co3	0.604885	1.49E-93	4.83E-89
Ifi47	0.602366	9.56E-78	3.09E-73
Psmb9	0.595356	1.53E-69	4.93E-65
Rpl35a	0.595152	9.9E-104	3.2E-99
Rtcb	0.594767	9.17E-58	2.96E-53
Rpl34	0.594724	1.36E-96	4.39E-92
Dusp1	0.594444	1.58E-18	5.09E-14
Rpl36a	0.594228	3.92E-70	1.26E-65
Cd72	0.59264	1.3E-43	4.21E-39
Rpl30	0.591658	1.5E-102	4.75E-98
Rpl18	0.590741	1.18E-95	3.82E-91
Eef1b2	0.587563	2.57E-72	8.29E-68
Coro2a	0.585648	1.22E-54	3.94E-50
Rps9	0.583611	1.2E-102	3.74E-98
Csf2ra	0.583051	1.34E-58	4.32E-54
Tsc22d3	0.577174	1.62E-24	5.23E-20
Efhd2	0.576475	1.87E-66	6.05E-62
Gm26917	0.573047	1.58E-61	5.12E-57
Eef1a1	0.570923	3.8E-127	1.2E-122
Gpr65	0.570493	1.09E-67	3.51E-63
Rps14	0.564747	1.4E-84	4.51E-80
Rgs1	0.563393	2.66E-16	8.6E-12
Nampt	0.563249	6.86E-62	2.21E-57
Fau	0.561115	1.2E-111	3.9E-107
Socs3	0.560591	2.73E-29	8.83E-25
2410006H	0.553591	6E-47	1.94E-42
Rpl8	0.551779	1.08E-74	3.49E-70
Ms4a4c	0.54919	5.77E-86	1.86E-81
Hacd2	0.548755	3.59E-47	1.16E-42
Gm43305	0.545413	1.77E-11	5.72E-07
Rps3	0.543702	2.22E-83	7.18E-79
Epsti1	0.542933	1.49E-43	4.81E-39
Itgax	0.542787	7.77E-72	2.51E-67
Rpl7a	0.542633	1.42E-82	4.57E-78

Ldha	0.54197	2.33E-39	7.52E-35
Cd69	0.541957	3.81E-40	1.23E-35
Psmb10	0.540451	1.18E-51	3.8E-47
C3ar1	0.539311	3.61E-50	1.16E-45
Tlr2	0.537563	1.22E-22	3.95E-18
Gm42601	0.537036	1.31E-51	4.22E-47
Etv6	0.535861	8.64E-51	2.79E-46
Ifih1	0.533715	4.87E-38	1.57E-33
Rps28	0.531313	2.73E-72	8.82E-68
Rad51b	0.530893	9.69E-39	3.13E-34
Zbtb16	0.528596	2.27E-47	7.32E-43
Apobec3	0.527082	1.18E-55	3.8E-51
Rps25	0.526598	7.03E-57	2.27E-52
Parp9	0.526545	6.83E-60	2.2E-55
Rplp1	0.525387	1.51E-95	4.86E-91
Gatm	0.52226	5.07E-33	1.64E-28
Rpl31	0.518145	3.38E-53	1.09E-48
Ier2	0.517808	5.77E-30	1.86E-25
Rab43	0.514514	5.32E-53	1.72E-48
Sp110	0.514218	8.59E-51	2.77E-46
Rpl21	0.513965	2.81E-88	9.07E-84
Usp18	0.513041	3.79E-48	1.22E-43
Egr1	0.510878	1.68E-24	5.43E-20
Rps3a1	0.510734	9.29E-82	3E-77
Gm46224	0.509773	4.22E-43	1.36E-38
Cyp4f18	0.508032	2.13E-88	6.89E-84
Myc	0.506748	1.03E-31	3.32E-27
Zfas1	0.502367	1.37E-69	4.44E-65
Chka	0.502202	1.03E-42	3.32E-38
Rpl23a	0.501613	5.48E-51	1.77E-46
Grn	0.500038	4.47E-95	1.44E-90
Eif3f	0.499968	1.15E-51	3.71E-47
Per1	0.496775	1.45E-42	4.68E-38
Cfb	0.495975	1.18E-68	3.81E-64
Apobec1	0.49489	2.01E-37	6.48E-33
Tnfsf13b	0.493836	1.99E-45	6.41E-41
Cstb	0.489776	3.2E-37	1.03E-32
Fmnl2	0.489549	5.99E-43	1.94E-38
Rpl37a	0.488962	1.47E-69	4.74E-65
Ifi209	0.488665	2.32E-66	7.51E-62
Capg	0.488381	1.44E-45	4.64E-41
Rnf169	0.486985	5.9E-23	1.9E-18
Pitpna	0.484431	8.05E-45	2.6E-40
Slc2a6	0.482406	8.95E-76	2.89E-71
mt-Atp6	0.481501	5.68E-53	1.84E-48
Ranbp2	0.477771	1.03E-38	3.34E-34
Fcgr2b	0.475718	2.94E-39	9.49E-35
Rpl10	0.475526	1.44E-73	4.65E-69

Znfx1	0.474485	1.3E-44	4.19E-40
Treml2	0.472048	1.58E-77	5.1E-73
Rpl5	0.469765	3.36E-46	1.09E-41
Ccnl1	0.469136	4.71E-43	1.52E-38
Tagln2	0.461906	6.57E-36	2.12E-31
Gm2a	0.460983	2.01E-43	6.5E-39
Cpd	0.459876	8.88E-31	2.87E-26
Clic4	0.45981	1.86E-44	6E-40
Icam1	0.459378	4.81E-19	1.55E-14
Gpi1	0.458718	1.21E-36	3.91E-32
Lat2	0.458001	1.12E-44	3.6E-40
Ccrl2	0.456222	7.48E-34	2.42E-29
Slc31a2	0.456196	8.27E-39	2.67E-34
Npm1	0.454669	1.12E-41	3.62E-37
Lpp	0.451481	1E-34	3.24E-30
Fcgr1	0.450788	2.4E-48	7.75E-44
Gm33370	0.449228	8.53E-53	2.75E-48
Rps15	0.448111	3.77E-45	1.22E-40
Atp6v0c	0.447225	1.23E-41	3.96E-37
Cox8a	0.446042	3.58E-44	1.16E-39
Ctsh	0.445537	1.73E-55	5.58E-51
Rpl7	0.445177	1.01E-53	3.25E-49
Scimp	0.44364	4.91E-61	1.59E-56
Pik3ip1	0.439971	2.01E-33	6.5E-29
Rpl22	0.439343	3.02E-50	9.75E-46
Ifi35	0.435763	6.02E-38	1.94E-33
Pml	0.434485	5.2E-38	1.68E-33
Hsp90ab1	0.431976	2.02E-34	6.51E-30
Trpm2	0.431429	2.55E-39	8.24E-35
Tap2	0.428301	1.03E-35	3.33E-31
Ripor2	0.428198	4.61E-39	1.49E-34
Plaat3	0.427339	1.59E-54	5.13E-50
2210408F2	0.426988	8.71E-42	2.81E-37
Birc3	0.42629	2.94E-34	9.48E-30
Cyba	0.426	4.82E-66	1.56E-61
Zfp36	0.424861	3.54E-10	1.14E-05
Ctsb	0.423436	1.27E-53	4.11E-49
Cep85	0.423357	1.26E-11	4.05E-07
Ssr4	0.420408	6.64E-40	2.14E-35
Vps54	0.41567	1.72E-30	5.56E-26
Rpl36al	0.415559	3.38E-38	1.09E-33
Rpl38	0.415154	1.98E-43	6.38E-39
B4galt1	0.41413	8.32E-20	2.69E-15
Tpst1	0.41181	3.21E-60	1.04E-55
Serp1	0.411707	3.42E-24	1.1E-19
Gcnt2	0.411271	3.75E-29	1.21E-24
Phf11d	0.410474	3.47E-41	1.12E-36
H2-M3	0.407967	1.66E-40	5.37E-36

Cited2	0.405673	4.2E-20	1.36E-15
Sdc3	0.405385	5.96E-47	1.92E-42
Creb5	0.399635	4.6E-26	1.49E-21
1110038B:	0.398445	5.38E-41	1.74E-36
Gpr84	0.397897	3.38E-06	0.109133
Smim3	0.395422	1.83E-43	5.91E-39
Eif5a	0.393564	1.56E-28	5.03E-24
Eef1g	0.390073	2.07E-30	6.68E-26
Zup1	0.389259	4.69E-27	1.51E-22
Ifit1	0.388288	2.1E-39	6.79E-35
Hspe1	0.388027	1.48E-32	4.77E-28
Gnl3	0.387654	9.16E-34	2.96E-29
Vim	0.387108	2.84E-23	9.18E-19
Gm12216	0.386652	3.74E-51	1.21E-46
Tpi1	0.386471	5.81E-18	1.88E-13
Pvt1	0.386179	2.81E-33	9.06E-29
Itgal	0.384791	4.96E-58	1.6E-53
Ctsz	0.383125	6.24E-62	2.01E-57
Tapbpl	0.382223	2.38E-43	7.69E-39
Nuak2	0.382105	2.59E-41	8.35E-37
Rsad2	0.380068	3.41E-39	1.1E-34
Rasgef1b	0.379057	1.5E-19	4.85E-15
Man2a1	0.378906	3.47E-34	1.12E-29
Rps17	0.374468	8.97E-31	2.9E-26
Akr1a1	0.374182	6.73E-28	2.17E-23
Eif4a1	0.37409	4.08E-32	1.32E-27
Zc3hav1	0.373315	6.04E-27	1.95E-22
Cd244a	0.373156	6.55E-60	2.12E-55
Morrbid	0.372832	1.49E-34	4.81E-30
Iqgap1	0.372434	5.56E-18	1.8E-13
Chd7	0.370817	6.22E-28	2.01E-23
Lgals3	0.370574	1.75E-29	5.64E-25
Arhgap24	0.368598	6.78E-16	2.19E-11
Rpl4	0.368358	1.4E-27	4.52E-23
Ybx1	0.367938	3.44E-31	1.11E-26
Slc31a1	0.366908	1.38E-32	4.46E-28
Trafd1	0.365877	4.38E-24	1.41E-19
Ube2l6	0.365365	3.25E-46	1.05E-41
Gnptab	0.365292	3.22E-37	1.04E-32
Ccr1	0.365148	5.49E-19	1.77E-14
Klf6	0.364729	4.38E-15	1.42E-10
Coro7	0.364691	3.37E-40	1.09E-35
Atp5g2	0.363726	2.11E-32	6.82E-28
Rabgap1l	0.363487	0.25711	1
Fbxw17	0.363124	6.46E-48	2.09E-43
Tapbp	0.363087	1.12E-26	3.62E-22
Sorl1	0.362343	5.64E-25	1.82E-20
Cept1	0.362167	2.51E-27	8.11E-23

Slc25a3	0.361867	1.53E-26	4.95E-22
Mmp14	0.360308	3.2E-32	1.03E-27
Klf9	0.360109	3E-40	9.67E-36
Map3k8	0.35957	2.55E-36	8.23E-32
Bcl2	0.359426	0.001874	1
Sema4a	0.359263	4.61E-50	1.49E-45
Plac8	0.357987	1.56E-40	5.03E-36
mt-Nd1	0.357634	9.82E-24	3.17E-19
Lpl	0.357473	3.33E-19	1.07E-14
Camk1d	0.35738	2.09E-28	6.75E-24
Msr1	0.357072	1.01E-57	3.27E-53
Cd63	0.355991	2.48E-19	8.01E-15
Eif3h	0.355182	2E-22	6.45E-18
Ehd1	0.35489	5.45E-48	1.76E-43
Siglec1	0.35202	4.46E-53	1.44E-48
Klf13	0.351948	5.76E-21	1.86E-16
Rbm47	0.35127	7.01E-14	2.26E-09
Psma5	0.351201	1.62E-23	5.23E-19
Acsl4	0.350132	9.11E-28	2.94E-23
Ly9	0.349293	1.77E-37	5.73E-33
Lacc1	0.349214	8.11E-26	2.62E-21
Rab5if	0.348381	2.56E-36	8.26E-32
Rps21	0.347884	1.64E-40	5.28E-36
Glrx	0.347676	8.79E-35	2.84E-30
AB124611	0.345486	1.41E-50	4.55E-46
Gm15964	0.344374	9.1E-48	2.94E-43
Prr5l	0.344356	1.13E-37	3.65E-33
Dhx58	0.343818	3.32E-38	1.07E-33
Ccnd2	0.342518	2.16E-62	6.98E-58
Prdx1	0.342226	1.73E-24	5.57E-20
Cd40	0.342135	1.71E-37	5.52E-33
Sh3bgrl3	0.341876	6.61E-30	2.13E-25
Uqcrh	0.340557	2.47E-24	7.97E-20
Trim30d	0.340551	3.05E-28	9.84E-24
Parp12	0.338097	1.02E-32	3.28E-28
Mllt3	0.337379	3.4E-19	1.1E-14
Zc3h7a	0.336937	6.17E-15	1.99E-10
C1qa	0.335173	4.72E-71	1.52E-66
Lrrfip1	0.335014	4.42E-21	1.43E-16
1600014C1	0.334348	2.41E-26	7.78E-22
Cblb	0.334297	0.000146	1
Txn1	0.333494	8.08E-26	2.61E-21
Abr	0.33277	6.13E-21	1.98E-16
Ppp1r15a	0.332723	7.32E-22	2.36E-17
Ppp1r21	0.332705	6.21E-17	2.01E-12
Hsp90aa1	0.331434	6.87E-12	2.22E-07
Rassf3	0.331057	7.91E-39	2.55E-34
Wwox	0.32986	7.02E-16	2.27E-11

Npnt	0.329601	2.03E-30	6.54E-26
Dgkz	0.32957	1.72E-22	5.56E-18
B3gnt2	0.329422	1.02E-24	3.28E-20
Xdh	0.328736	7.33E-47	2.37E-42
Hnrnpa1	0.328446	1.84E-26	5.95E-22
Sat1	0.328198	1.1E-15	3.55E-11
Csf2rb2	0.327223	2.87E-24	9.26E-20
Chmp4b	0.32713	1.69E-31	5.46E-27
Snhg12	0.324271	1.63E-27	5.27E-23
Oas2	0.323573	1.19E-50	3.85E-46
Arid5a	0.323036	7.24E-32	2.34E-27
Cd48	0.322795	9.9E-25	3.2E-20
Aldoa	0.322286	5.79E-14	1.87E-09
Slfn8	0.320265	1.05E-21	3.37E-17
C1qb	0.320169	2.24E-68	7.24E-64
March5	0.31995	1.09E-33	3.51E-29
Itpr1	0.319352	2.34E-20	7.55E-16
Casp4	0.319301	3.28E-27	1.06E-22
Pilra	0.318337	5.15E-23	1.66E-18
Phf11a	0.318277	5.73E-43	1.85E-38
Naca	0.318001	9.76E-29	3.15E-24
Snhg1	0.31701	1.33E-31	4.28E-27
Uqcrq	0.316558	1.22E-22	3.95E-18
Rps29	0.316128	9.49E-37	3.06E-32
Kcnk6	0.31541	3.51E-23	1.13E-18
Tmem106a	0.314781	3.41E-29	1.1E-24
Trim14	0.313399	4.79E-22	1.55E-17
Scpep1	0.313252	5.71E-23	1.84E-18
Echs1	0.313061	1.51E-22	4.86E-18
Ddx60	0.312372	6.07E-20	1.96E-15
Tmem104	0.310864	1.58E-17	5.09E-13
Siglecg	0.310046	1.44E-35	4.65E-31
F830016B(	0.308998	4.36E-34	1.41E-29
Rnase6	0.308528	6.03E-32	1.95E-27
Tns1	0.30703	1.27E-25	4.1E-21
Ston1	0.30702	1.97E-40	6.37E-36
Tagap	0.306632	1.93E-12	6.24E-08
Fcgr3	0.306058	5.23E-21	1.69E-16
Dhx9	0.305282	1.33E-18	4.3E-14
Tkt	0.304726	2.07E-21	6.67E-17
P2rx4	0.304022	1.21E-21	3.9E-17
Ifit3b	0.30378	2.35E-32	7.59E-28
Lrmda	0.303551	4.47E-31	1.44E-26
Coro1c	0.303079	1.11E-17	3.6E-13
Btf3	0.302279	3.57E-23	1.15E-18
Slc2a1	0.302223	4.09E-23	1.32E-18
Hck	0.300141	3.15E-21	1.02E-16
Zfand5	0.299582	5.59E-12	1.81E-07

Snx2	0.299353	6.51E-19	2.1E-14
Taf1d	0.298828	8.63E-22	2.79E-17
C1qbp	0.298303	9.48E-23	3.06E-18
Myo5a	0.298105	1.17E-24	3.79E-20
Trim25	0.298015	2.05E-23	6.62E-19
Cox5b	0.297471	5.27E-22	1.7E-17
Pip4p1	0.297323	1.79E-23	5.78E-19
Slc1a3	0.296496	4.96E-16	1.6E-11
Gsap	0.296127	1.97E-17	6.36E-13
Cox7b	0.295477	1.95E-25	6.29E-21
Calm1	0.294974	3.06E-15	9.87E-11
Minpp1	0.293602	6.54E-30	2.11E-25
Atp5e	0.292448	4.2E-22	1.36E-17
C3	0.292061	1.85E-32	5.97E-28
Dtx3l	0.291337	5.87E-28	1.9E-23
Milr1	0.291197	7.09E-32	2.29E-27
Lncpint	0.290575	1.17E-16	3.79E-12
Neat1	0.290388	2.25E-19	7.26E-15
Naa25	0.289832	2.67E-26	8.61E-22
Utrn	0.288572	4.47E-05	1
Aprt	0.28743	8.53E-27	2.75E-22
CAAA0114[illegible]	0.286888	2.95E-18	9.53E-14
Kpna4	0.286305	6.89E-15	2.22E-10
Sp140	0.285167	2.77E-25	8.95E-21
mt-Nd4l	0.284356	2.09E-21	6.74E-17
Fnbp4	0.284161	4.16E-18	1.34E-13
Pgs1	0.283865	1.48E-27	4.78E-23
Gns	0.283258	5.05E-18	1.63E-13
Eif3i	0.283062	1.55E-17	5E-13
Xrn2	0.281953	7.21E-24	2.33E-19
Pomp	0.281719	9.57E-17	3.09E-12
Mapkapk2	0.281685	1.8E-17	5.8E-13
Ripk2	0.280237	5.56E-17	1.8E-12
Prpf38b	0.279727	1.2E-20	3.88E-16
Cd86	0.279242	2.13E-13	6.89E-09
Ncl	0.278622	3.85E-14	1.24E-09
Ftl1	0.277964	9.39E-16	3.03E-11
Gm47283	0.277822	2.12E-12	6.85E-08
Atp5mpl	0.277609	2.27E-18	7.33E-14
Hspa8	0.276592	5.06E-22	1.63E-17
Btaf1	0.275739	7.72E-18	2.49E-13
Uba7	0.274475	2.37E-18	7.65E-14
Calhm6	0.274336	9.26E-23	2.99E-18
Ttc39b	0.27408	5.34E-15	1.72E-10
Tnfaip3	0.273991	3.59E-09	0.000116
Ifrd1	0.272954	1.91E-18	6.18E-14
B4galnt1	0.271763	4.69E-20	1.51E-15
Rhbdf2	0.271073	2.77E-25	8.94E-21

Hint1	0.270491	1.24E-18	4E-14
AI506816	0.26966	7.87E-20	2.54E-15
Pgam1	0.2688	3.85E-10	1.24E-05
Gls	0.268729	1.35E-21	4.35E-17
Daxx	0.268605	4.85E-21	1.56E-16
Tbc1d1	0.268006	7.36E-23	2.38E-18
Sh3bp2	0.267938	5.24E-20	1.69E-15
Nod1	0.267879	4.79E-23	1.55E-18
Abcg3	0.26777	9.7E-22	3.13E-17
Cox4i1	0.267299	3.91E-21	1.26E-16
Ppargc1b	0.26713	7.36E-34	2.38E-29
Slc25a22	0.266731	6.75E-30	2.18E-25
Ppan	0.266157	5.2E-23	1.68E-18
Nsd2	0.266045	6.17E-16	1.99E-11
Slc2a3	0.266001	6.47E-18	2.09E-13
Igsf6	0.26551	1.46E-18	4.7E-14
Osbpl9	0.265489	2.83E-14	9.15E-10
Cox5a	0.265397	6.61E-20	2.13E-15
Arhgef3	0.265265	7E-12	2.26E-07
Adrb1	0.264446	3.76E-16	1.21E-11
Tmod3	0.263605	2.46E-15	7.93E-11
Lgals9	0.263516	3.18E-22	1.03E-17
Plin2	0.263448	1.63E-20	5.25E-16
Fyn	0.263016	0.543318	1
Diaph1	0.262387	8.67E-27	2.8E-22
Atp1a3	0.262262	1.81E-29	5.85E-25
Rtraf	0.262058	2.75E-13	8.88E-09
Eepd1	0.261867	8.93E-16	2.88E-11
Myo1e	0.261814	3.91E-12	1.26E-07
Arpc1b	0.260173	6.22E-22	2.01E-17
Pla2g7	0.259887	1.62E-34	5.24E-30
Cmtr1	0.259808	1.16E-15	3.74E-11
Gnl2	0.259611	2.01E-17	6.49E-13
Coq10b	0.259154	7.03E-18	2.27E-13
Pnpla2	0.258293	1.5E-13	4.83E-09
Ppm1h	0.258204	2.31E-12	7.46E-08
Rmdn3	0.257806	8.95E-23	2.89E-18
Foxn2	0.257805	1.33E-18	4.28E-14
Atp1a1	0.257316	2.69E-15	8.68E-11
AY036118	0.257191	0.02155	1
Ncoa1	0.257165	8.07E-15	2.61E-10
Adar	0.256628	5.22E-23	1.69E-18
H2-Oa	0.256335	1.67E-10	5.39E-06
Eif3e	0.256171	2.53E-17	8.16E-13
Pstpip1	0.2558	3.53E-25	1.14E-20
Anxa5	0.255348	1.08E-17	3.47E-13
Pgk1	0.255254	7E-18	2.26E-13
Hivep2	0.255228	3.17E-11	1.02E-06

Tank	0.255108	9.17E-17	2.96E-12
Il27	0.254847	7.1E-29	2.29E-24
Colgalt1	0.253937	1.77E-14	5.72E-10
Eif3k	0.253859	8.86E-19	2.86E-14
Ptma	0.25245	4.35E-13	1.4E-08
Dst	0.252412	1.38E-21	4.46E-17
Dnajc13	0.252376	4.6E-13	1.49E-08
Rbpj	0.252374	2.39E-18	7.7E-14
Rftn1	0.252078	6.37E-20	2.06E-15
Atp5l	0.25203	1.69E-16	5.46E-12
Tomm20	0.251324	3.15E-16	1.02E-11
Cd47	0.249563	1.18E-11	3.81E-07
Plekho2	0.249436	1.49E-22	4.8E-18
Helz2	0.248246	8.93E-33	2.88E-28
Ldlr	0.247047	9.98E-26	3.22E-21
Relb	0.246988	4.29E-18	1.38E-13
Ifitm2	0.246081	5.98E-38	1.93E-33
Eif4ebp1	0.245837	1.13E-21	3.65E-17
Mlxip	0.245133	4.74E-15	1.53E-10
Sharpin	0.244492	5.21E-19	1.68E-14
Hs3st3b1	0.244207	2.46E-29	7.95E-25
Nenf	0.243995	6.84E-22	2.21E-17
Hspa1a	0.243621	9.46E-18	3.05E-13
Cep152	0.243521	2.66E-10	8.6E-06
Taldo1	0.242477	2.81E-12	9.08E-08
Pnpt1	0.242337	2E-22	6.46E-18
Ly6i	0.241929	2.8E-38	9.05E-34
Got1	0.241928	1.58E-18	5.11E-14
Gpx1	0.241883	1.23E-18	3.98E-14
Gramd1b	0.241699	0.000126	1
Max	0.241195	1.82E-18	5.88E-14
Pik3ap1	0.241026	1.74E-14	5.62E-10
E230001N(	0.241016	2.75E-33	8.88E-29
Ms4a6b	0.240205	3.26E-10	1.05E-05
Ddx39	0.240031	1.06E-14	3.42E-10
Oas1g	0.239975	3.4E-39	1.1E-34
Tmed2	0.239733	1.12E-15	3.6E-11
Atp5g3	0.23914	3.15E-12	1.02E-07
C030034L1	0.238826	1.88E-26	6.08E-22
A330040F1	0.238586	1.68E-16	5.42E-12
Junb	0.238205	3.07E-10	9.91E-06
Cpq	0.237808	2.2E-19	7.11E-15
Cd44	0.237639	6.85E-15	2.21E-10
C1galt1c1	0.237559	3.16E-14	1.02E-09
Tmsb10	0.236345	6.18E-07	0.019939
Anxa3	0.236307	3.23E-15	1.04E-10
Nmi	0.235219	2.5E-15	8.07E-11
Rab37	0.235107	2.23E-39	7.2E-35

Slc25a25	0.235085	2.85E-26	9.19E-22
H2-T24	0.234815	6.57E-17	2.12E-12
Rab20	0.234803	2.08E-11	6.73E-07
Tmem140	0.234738	1.54E-13	4.98E-09
Clcn7	0.234438	5.82E-15	1.88E-10
Slfn9	0.234247	2.71E-24	8.75E-20
Hspd1	0.233959	4.2E-12	1.35E-07
Sdf4	0.233922	2.65E-18	8.55E-14
Mbd2	0.233707	6.18E-15	2E-10
Parp10	0.233523	2.21E-21	7.13E-17
Icosl	0.233195	2.55E-16	8.24E-12
Tgif1	0.232562	1.81E-17	5.84E-13
Ccdc102a	0.232251	1.24E-32	3.99E-28
Eif3a	0.231997	1.18E-12	3.82E-08
mt-Cytb	0.231492	2.07E-21	6.7E-17
St14	0.231422	1.11E-23	3.57E-19
Nceh1	0.2303	7.14E-27	2.31E-22
Acot9	0.229894	2.15E-23	6.96E-19
Zc3h12a	0.229843	5.46E-13	1.76E-08
B430306N(	0.229727	7.36E-28	2.38E-23
Eif1a	0.228796	5.78E-19	1.87E-14
Rcan1	0.228395	0.031109	1
Flna	0.228323	2.17E-09	7.01E-05
Lyst	0.227817	1.7E-07	0.005486
Mt1	0.227237	3.27E-08	0.001057
Gars	0.227072	6.61E-14	2.13E-09
Mdfic	0.227004	1.07E-11	3.46E-07
Etv3	0.22654	6.16E-14	1.99E-09
Alkbh1	0.226516	2.14E-20	6.9E-16
Tent5a	0.226506	1.25E-13	4.04E-09
Lrp10	0.226461	2.2E-17	7.11E-13
Mat2a	0.22635	1.04E-09	3.36E-05
Herc4	0.2255	1.3E-12	4.19E-08
Uvrag	0.225264	2.8E-14	9.05E-10
Fcgrt	0.224993	2.51E-14	8.11E-10
Zdhhc21	0.224959	7.92E-21	2.56E-16
Atp6v0e	0.22472	1.9E-13	6.15E-09
Il10ra	0.224662	2.6E-10	8.39E-06
Rnf114	0.224481	8.37E-15	2.7E-10
Srm	0.224423	1.07E-27	3.46E-23
Tor1aip1	0.223688	5.86E-13	1.89E-08
Ddhd1	0.223555	1.54E-21	4.96E-17
Rasa4	0.222828	8.46E-12	2.73E-07
Cfl1	0.222371	1.01E-13	3.28E-09
Morc3	0.222168	4.8E-13	1.55E-08
Dram2	0.221921	1.87E-08	0.000603
Gm50237	0.221855	2.36E-34	7.63E-30
Vmp1	0.221531	1.73E-11	5.58E-07

H2afy	0.221386	1.13E-20	3.64E-16
Rbms1	0.220867	2.61E-14	8.44E-10
Cox6c	0.220646	3.12E-14	1.01E-09
Slc9a3r1	0.220528	3.51E-14	1.13E-09
Dek	0.220365	2.87E-14	9.27E-10
Anxa6	0.219615	3.69E-15	1.19E-10
Slc1a5	0.219605	2.86E-21	9.24E-17
Fam174a	0.219416	3.33E-17	1.07E-12
Mrpl30	0.218988	5.78E-15	1.87E-10
Gapdh	0.218789	1.44E-10	4.66E-06
Ulbp1	0.218648	1.56E-15	5.02E-11
Tor1aip2	0.218264	5.69E-12	1.84E-07
Crybg1	0.218124	1.6E-23	5.16E-19
Bbx	0.217788	6.72E-11	2.17E-06
Dbnl	0.217777	1.2E-10	3.88E-06
Wdr43	0.217459	6E-17	1.94E-12
Gpr18	0.21674	3.08E-28	9.94E-24
Mapre2	0.216524	1.06E-09	3.41E-05
Coa5	0.215601	2.25E-19	7.26E-15
Tmem154	0.215338	3.44E-25	1.11E-20
Atf3	0.214777	1.69E-07	0.005471
Gas7	0.213299	2.59E-31	8.35E-27
Tent2	0.212779	2.34E-09	7.56E-05
Wars	0.212489	2.98E-14	9.63E-10
Asah1	0.212363	6.44E-17	2.08E-12
Lmln	0.211779	5.93E-18	1.91E-13
Abhd11	0.211566	9.56E-19	3.09E-14
Actr3	0.211469	8.63E-12	2.78E-07
Nfkbiz	0.211438	3.76E-10	1.21E-05
Ogfr	0.210944	8.17E-15	2.64E-10
Cox6a1	0.210663	1.53E-12	4.93E-08
Acer3	0.210238	6.12E-13	1.98E-08
Cd180	0.209869	4.42E-06	0.142767
Galc	0.209568	1E-12	3.24E-08
Cct2	0.209336	1.29E-11	4.15E-07
Akr1b3	0.208773	1.42E-11	4.58E-07
Fcer1g	0.208472	1.24E-18	3.99E-14
Sla	0.207905	7.9E-06	0.255137
Atp5c1	0.207545	3.79E-13	1.22E-08
Tlr12	0.207372	2.33E-13	7.52E-09
Shisa5	0.206751	4.66E-15	1.51E-10
Gsto1	0.206415	1.83E-23	5.91E-19
Prkch	0.206163	7.07E-07	0.022816
Samd9l	0.205485	2.22E-22	7.17E-18
Tlr1	0.20548	5.38E-16	1.74E-11
Map2k2	0.205273	2.76E-13	8.89E-09
Nsa2	0.205198	1.1E-10	3.55E-06
Atp5b	0.204143	1.22E-09	3.94E-05

Psma4	0.203964	1.77E-11	5.72E-07
Gvin1	0.203413	1.36E-13	4.39E-09
Trim12a	0.203268	7.57E-12	2.44E-07
Nsmce2	0.202933	1.53E-09	4.95E-05
A930007I1	0.202767	8.41E-18	2.71E-13
Hpcal1	0.202452	3.52E-18	1.14E-13
Uba52	0.202191	1.25E-17	4.05E-13
Timm23	0.201975	1.65E-13	5.33E-09
Rac2	0.201945	2.01E-09	6.5E-05
Gsdmd	0.2019	3.02E-14	9.74E-10
Isg20	0.20169	9.2E-29	2.97E-24
Tnfsf12	0.201658	2.73E-15	8.82E-11
Lrrk1	0.201609	9.66E-11	3.12E-06
Eef1d	0.20151	1.67E-13	5.4E-09
H2-DMb2	0.2012	2.13E-21	6.88E-17
Ms4a6d	0.201179	2.23E-09	7.19E-05
Tmem251	0.200994	2.22E-17	7.17E-13
Art3	0.200693	4.66E-20	1.5E-15
Pfdn5	0.200517	9.8E-14	3.16E-09
Itgb2	0.200517	4.42E-11	1.43E-06
Prelid1	0.200494	2.08E-14	6.7E-10
Myo9b	0.200473	6.15E-13	1.99E-08
Hnrnpdl	0.200403	1.6E-08	0.000516
Dclre1c	0.200087	1.66E-10	5.37E-06
Rab2a	0.199555	1.98E-13	6.39E-09
Slc7a1	0.199437	3.33E-18	1.08E-13
Atxn1	0.198986	0.004559	1
Mapkapk3	0.198823	3.41E-11	1.1E-06
Ndst2	0.198493	3.02E-15	9.74E-11
Psma7	0.198255	1.52E-13	4.91E-09
Hsd17b12	0.197978	3.42E-16	1.11E-11
Rab27a	0.197807	5.27E-14	1.7E-09
Znrf3	0.197603	2.81E-16	9.07E-12
Nfe2l2	0.197551	4.16E-11	1.34E-06
Cers6	0.197354	5.81E-07	0.01877
Ndufa13	0.196679	3.49E-10	1.13E-05
Snrpg	0.196485	5.09E-10	1.64E-05
Otud4	0.196017	1.32E-11	4.25E-07
Tbc1d8	0.195481	1.03E-16	3.34E-12
Rspo1	0.195374	8.57E-41	2.77E-36
Plek	0.19479	9.09E-06	0.2936
Gm4070	0.194661	4.06E-13	1.31E-08
Arl5c	0.194092	1.95E-15	6.3E-11
Nhp2	0.19408	1.21E-15	3.91E-11
Slc25a5	0.193552	1.4E-10	4.51E-06
Srsf2	0.193345	5.48E-10	1.77E-05
Cycs	0.19328	4.88E-09	0.000158
Usp25	0.19297	4E-11	1.29E-06

Luzp1	0.192356	4.64E-16	1.5E-11
Wdfy1	0.192354	5.74E-11	1.85E-06
Ap3b1	0.192272	1.01E-10	3.26E-06
Slc30a7	0.192053	1.23E-10	3.98E-06
Tnfsf8	0.19197	4.45E-16	1.44E-11
Rnf19b	0.191671	2.49E-16	8.04E-12
Nudt9	0.191283	7.17E-15	2.32E-10
Kif3b	0.191123	9.02E-13	2.91E-08
Scfd2	0.191065	3.59E-09	0.000116
Ran	0.190563	9.19E-06	0.296573
Snx10	0.190179	4.51E-09	0.000146
Idnk	0.190085	4.65E-14	1.5E-09
Tpr	0.189542	6.53E-14	2.11E-09
Irgm2	0.189443	7.96E-08	0.002571
Rilpl2	0.189382	1.34E-16	4.31E-12
Mfsd12	0.189304	3.67E-21	1.18E-16
Golgb1	0.189113	4.84E-13	1.56E-08
Etf1	0.188649	1.92E-11	6.19E-07
Prkca	0.188597	7.18E-05	1
Uri1	0.188119	1.05E-13	3.37E-09
C1qc	0.187954	1.95E-28	6.3E-24
Xpo6	0.187743	1.92E-12	6.19E-08
Pgd	0.18745	1.31E-12	4.23E-08
Hk3	0.187248	1.53E-07	0.004949
Ndufa1	0.187181	4.71E-14	1.52E-09
Vps13c	0.186861	1.18E-13	3.82E-09
Nt5c	0.186775	4.37E-17	1.41E-12
Sdad1	0.18668	6.03E-13	1.95E-08
Alkbh2	0.186054	1.4E-26	4.51E-22
Epb41l4ao	0.185941	1.94E-15	6.26E-11
Setdb2	0.185743	1.28E-14	4.15E-10
Mfsd1	0.184774	5.45E-14	1.76E-09
Gpr160	0.184179	3.34E-09	0.000108
Ndufb1-ps	0.183989	7.16E-12	2.31E-07
Ncf1	0.18391	1.97E-09	6.34E-05
A930015D(	0.183836	2.65E-13	8.57E-09
Pabpc1	0.183798	6.47E-09	0.000209
Sap30	0.183785	1.44E-17	4.64E-13
Spop	0.183759	8.61E-12	2.78E-07
Slc49a4	0.183219	6.14E-09	0.000198
Msrb1	0.182862	4.59E-15	1.48E-10
2810013P(	0.182781	7.52E-09	0.000243
Hebp1	0.182525	3.24E-14	1.04E-09
Ppa1	0.18241	5.43E-21	1.75E-16
Ak2	0.182324	3E-09	9.68E-05
Il18	0.181986	3.85E-10	1.24E-05
Lfng	0.181463	1.39E-11	4.48E-07
Pde7a	0.181231	2.33E-05	0.75097

Slc43a2	0.181194	0.000204	1
D16Ertd47	0.180945	2.29E-15	7.41E-11
Gm7030	0.18012	6.42E-25	2.07E-20
Bola2	0.18001	4.66E-14	1.51E-09
Hivep1	0.17996	1.96E-13	6.32E-09
Bri3bp	0.179769	1.22E-18	3.94E-14
Pirb	0.17938	2.13E-13	6.88E-09
Mphosph1(	0.178513	2.72E-13	8.79E-09
A930037H(	0.178312	2.35E-22	7.58E-18
Nop56	0.178215	5.34E-09	0.000173
Sgcb	0.177894	1.28E-18	4.13E-14
Atp5h	0.177625	3.38E-11	1.09E-06
Tubb5	0.177153	1.79E-07	0.005793
Sub1	0.177006	1.31E-07	0.004233
Pi4ka	0.176995	6.26E-13	2.02E-08
Fam241a	0.176885	1.57E-29	5.07E-25
Cox6b1	0.176871	5.08E-11	1.64E-06
Mrpl54	0.176768	2.06E-11	6.65E-07
Dennd1b	0.176767	2.68E-08	0.000865
Slc38a6	0.17628	5.61E-10	1.81E-05
Phf20l1	0.176105	3.65E-10	1.18E-05
Raly	0.175718	2.77E-14	8.94E-10
Hspa5	0.175706	2.33E-08	0.000751
Pced1b	0.175423	1.54E-13	4.96E-09
Dop1b	0.175362	7.28E-13	2.35E-08
Trpv4	0.175222	1.87E-23	6.05E-19
Parp11	0.175081	3.71E-14	1.2E-09
Crybb1	0.174982	0.001431	1
Grina	0.174205	6.18E-12	2E-07
Psme4	0.174182	4.39E-11	1.42E-06
Anp32b	0.173773	1.38E-14	4.45E-10
Atf6	0.173127	2.06E-10	6.66E-06
Pdk3	0.173026	5.55E-12	1.79E-07
Sfxn1	0.172362	5.37E-14	1.73E-09
Atg3	0.172198	5.07E-13	1.64E-08
Tyk2	0.171969	3.43E-11	1.11E-06
Psmb2	0.171651	6.48E-08	0.002093
Nfkbib	0.17119	1.33E-10	4.28E-06
Casp1	0.170947	4.19E-12	1.35E-07
Plbd2	0.170278	2.25E-11	7.26E-07
Rps27	0.170266	6.27E-11	2.02E-06
Rbm17	0.169636	1.03E-18	3.32E-14
Pfkfb3	0.169385	1.85E-08	0.000598
Slc15a4	0.169301	1.69E-15	5.45E-11
Relt	0.16915	1.94E-18	6.25E-14
Eef2	0.168918	4.86E-10	1.57E-05
Cox7a2	0.168547	3.69E-10	1.19E-05
Trim34a	0.168479	4.09E-13	1.32E-08

Arf4	0.168452	6.16E-07	0.019893
Nktr	0.167778	9.03E-11	2.92E-06
Mvp	0.167463	8.2E-12	2.65E-07
Snhg6	0.167393	7.78E-21	2.51E-16
Pld4	0.167007	6.94E-11	2.24E-06
Gm17268	0.166881	3.94E-15	1.27E-10
Lcp2	0.1668	5.92E-06	0.190992
M6pr	0.166509	1.1E-08	0.000355
Pfkl	0.166327	2.52E-11	8.13E-07
Ctsl	0.166201	6.37E-09	0.000206
Coq7	0.166091	5.92E-18	1.91E-13
Taok3	0.165687	1.78E-10	5.73E-06
Hmgb2	0.16557	0.000384	1
Dnm1l	0.165288	1.37E-12	4.42E-08
Hnrnpll	0.164926	3.49E-08	0.001127
Higd1a	0.164873	9.67E-10	3.12E-05
Svbp	0.164283	2.18E-09	7.03E-05
Mcl1	0.164024	4.51E-07	0.014553
Atp5o	0.163406	7.6E-09	0.000246
Cep128	0.163141	3.69E-11	1.19E-06
Haao	0.162053	4.11E-19	1.33E-14
Vars	0.162013	3.61E-08	0.001167
Adipor2	0.162004	2.29E-13	7.38E-09
Lrpap1	0.161916	2.18E-08	0.000704
Ppfia4	0.161568	9.36E-07	0.030222
Gm37240	0.161357	2.59E-07	0.008361
Slc12a9	0.161345	2.41E-09	7.78E-05
Nmd3	0.161038	1.67E-09	5.38E-05
Cct5	0.160899	1.81E-09	5.85E-05
Uqcc2	0.160592	2.55E-08	0.000824
Bak1	0.16059	3.05E-12	9.84E-08
Ptrhd1	0.160582	1.44E-11	4.66E-07
Eps8	0.160394	1.06E-10	3.41E-06
Atrnl1	0.160285	3.33E-18	1.08E-13
Vps13a	0.160083	1.69E-10	5.47E-06
Dynll1	0.160025	1.64E-06	0.052949
Dnajb14	0.159912	4.03E-09	0.00013
0610012G(	0.159658	1.37E-10	4.42E-06
Tpd52	0.15934	1.29E-08	0.000417
Osm	0.158773	1.12E-08	0.000363
Hcst	0.158112	5.51E-12	1.78E-07
Mthfr	0.157767	1.29E-11	4.17E-07
Tex2	0.157227	3.05E-08	0.000984
Plekhf2	0.157046	2.48E-15	8.01E-11
Ppib	0.156924	2.27E-06	0.073242
Rnh1	0.156898	9.58E-08	0.003092
Fbl	0.156755	2.62E-07	0.008462
Gusb	0.156738	1.17E-08	0.000378

Abl2	0.156594	8.56E-09	0.000276
Atp5j2	0.156487	3.21E-06	0.103608
Man2b1	0.155841	8.17E-12	2.64E-07
Ola1	0.155603	1.3E-10	4.2E-06
Capn1	0.155157	1.31E-14	4.24E-10
Psma2	0.155155	9.93E-08	0.003205
Rras2	0.154956	1.18E-13	3.8E-09
2410002F2	0.154841	1.89E-05	0.611695
Aatf	0.154798	5.42E-12	1.75E-07
Ndufv3	0.154753	2.08E-08	0.000673
Plekha1	0.154714	5.14E-11	1.66E-06
Pde4b	0.154452	0.266336	1
Kxd1	0.154442	3.59E-08	0.00116
C5ar1	0.15437	5.26E-06	0.169758
Lrp12	0.154313	1.23E-08	0.000397
Spata5	0.154037	1.14E-09	3.68E-05
Ftl1-ps1	0.153922	2.64E-17	8.52E-13
Tmem184b	0.153816	9.66E-12	3.12E-07
Vapa	0.153809	1.58E-05	0.51024
Ankle2	0.153791	9.25E-09	0.000299
Snd1	0.153733	1.81E-09	5.84E-05
Gyg	0.152838	1.13E-07	0.003656
Cp	0.152642	7.89E-11	2.55E-06
Gm14023	0.152539	1.22E-10	3.94E-06
Synj1	0.152532	3.77E-08	0.001216
Nop14	0.152243	5.3E-08	0.001711
Htt	0.152219	5.99E-10	1.93E-05
Exoc6	0.152011	4.99E-10	1.61E-05
Anapc5	0.151817	4.49E-08	0.00145
Tubb6	0.151791	1.83E-11	5.89E-07
Limd2	0.151617	4.91E-06	0.158566
Prmt3	0.151549	4.66E-16	1.5E-11
Larp1	0.151361	2.09E-11	6.75E-07
Neu1	0.151297	1.05E-08	0.000339
Pik3r1	0.150924	0.000713	1
Aoah	0.15087	3.69E-20	1.19E-15
Ecpas	0.150318	2.95E-09	9.52E-05
Hat1	0.150177	3.48E-09	0.000112
Sys1	0.15011	4.54E-07	0.014653
Tpm4	0.1501	8.17E-09	0.000264
Cyb5r3	0.149626	4.04E-09	0.00013
Tlr7	0.149444	0.004613	1
Psmg4	0.149178	2.76E-11	8.9E-07
Dnttip1	0.149024	1.27E-16	4.09E-12
Cntrl	0.148642	7.09E-08	0.00229
Txndc17	0.148417	4.65E-08	0.001503
Sri	0.148403	7.53E-11	2.43E-06
Elovl1	0.148107	1.44E-06	0.046625

Nvl	0.147426	1.69E-11	5.46E-07
Ndufb8	0.147341	3.61E-09	0.000117
mt-Nd2	0.146885	5.4E-07	0.017436
Eea1	0.14687	1.35E-09	4.37E-05
Ndufb9	0.146846	5.82E-07	0.018781
Ccdc134	0.14684	2.26E-12	7.29E-08
Slc3a2	0.146686	8.53E-08	0.002755
Hmox1	0.146671	8.12E-05	1
Gm40645	0.146638	1.51E-13	4.87E-09
Gm31718	0.146178	1.15E-15	3.71E-11
Anapc16	0.145651	3.36E-10	1.09E-05
Ptk2b	0.145485	1.9E-05	0.614944
Zfp106	0.145381	2.01E-11	6.5E-07
Acadl	0.144812	5.58E-06	0.180284
4930469K1	0.144671	1.83E-07	0.005907
Hps5	0.144623	1.22E-10	3.94E-06
Cct8	0.144415	2.41E-07	0.007784
Micu2	0.144415	1.46E-09	4.73E-05
Atp5g1	0.144413	7.04E-08	0.002272
Creld2	0.144393	6.05E-07	0.01952
Sdhb	0.144302	7.5E-07	0.02423
Snrpe	0.144272	3.59E-06	0.115837
Srsf7	0.144193	0.000286	1
Rbm26	0.144072	4.11E-07	0.013283
Lrch3	0.143868	1.46E-06	0.047135
Psmb4	0.14361	7.4E-08	0.002388
Gpr35	0.143571	2.78E-20	8.98E-16
Stx16	0.143433	1.48E-07	0.004779
Dennd4a	0.143166	0.347554	1
Frrs1	0.143084	3.64E-05	1
Jdp2	0.142981	6.36E-17	2.05E-12
Irak2	0.14279	0.005285	1
Fryl	0.142743	0.000111	1
Gng5	0.142615	5.36E-07	0.017308
2310001H1	0.142583	2.56E-16	8.25E-12
Ipcef1	0.14251	8.61E-10	2.78E-05
Hspa4	0.142435	1.37E-09	4.42E-05
Rbm25	0.142426	1.5E-10	4.86E-06
Lrrc59	0.142281	3.96E-12	1.28E-07
Tnfsf10	0.142258	7.05E-14	2.28E-09
Coro1a	0.142214	4.29E-06	0.138342
Il6	0.141883	1.46E-10	4.71E-06
Naip2	0.141709	2E-08	0.000646
Pcna	0.141629	2.58E-05	0.833
Ppia	0.141569	3.32E-05	1
Cfap410	0.141337	4.04E-11	1.31E-06
Nob1	0.140895	4.32E-08	0.001395
Sod2	0.140802	2.31E-10	7.47E-06

Cd81	0.140743	2.22E-08	0.000716
Psmd8	0.14073	4.91E-06	0.158548
Tmbim1	0.140607	4.89E-12	1.58E-07
Eif2s1	0.140232	2.16E-08	0.000697
Nedd4l	0.140219	3.58E-11	1.16E-06
Mrps24	0.139942	1.46E-09	4.7E-05
H2afj	0.139645	6.49E-06	0.209588
Gtf2f1	0.139336	2.77E-07	0.008932
Eif4e3	0.139266	5.47E-10	1.77E-05
Rufy3	0.139166	5.46E-13	1.76E-08
Slamf6	0.138931	3.55E-10	1.15E-05
Nucb2	0.138766	3.65E-13	1.18E-08
Eva1b	0.138678	1.32E-12	4.26E-08
Alpk1	0.13859	1.68E-11	5.42E-07
Inpp1	0.138465	8.53E-15	2.75E-10
Trim21	0.138353	3.8E-12	1.23E-07
Litaf	0.138275	8.36E-07	0.027004
Nab1	0.138225	1.8E-09	5.82E-05
C9orf72	0.138152	5.95E-09	0.000192
Csnk1g1	0.137745	2.9E-06	0.093655
Snrpd2	0.137504	8.46E-10	2.73E-05
Nudt19	0.137291	7.1E-20	2.29E-15
Lair1	0.13716	4.84E-09	0.000156
Arhgef7	0.136901	2.83E-07	0.009147
Kntc1	0.136861	1.7E-09	5.5E-05
Sptlc2	0.136807	6.1E-06	0.196958
Farsa	0.136592	4.14E-10	1.34E-05
Gde1	0.136507	2.09E-10	6.76E-06
Snhg16	0.136116	6.67E-12	2.15E-07
Rassf4	0.135918	1.74E-07	0.005614
Sass6	0.135813	3.49E-11	1.13E-06
Aim2	0.135582	8.14E-06	0.26274
B4galt5	0.135354	3.62E-06	0.116976
Dapk1	0.135337	1.87E-07	0.006029
Rapgef2	0.135123	3.35E-08	0.00108
Rnf149	0.134943	5.65E-08	0.001823
Slirp	0.134817	7.93E-11	2.56E-06
Psma3	0.134742	5.45E-07	0.017608
Pmm2	0.134546	8.77E-09	0.000283
Apmap	0.134417	4.61E-10	1.49E-05
Bclaf1	0.134078	1.41E-06	0.045594
Lifr	0.133888	0.005844	1
Aopep	0.133821	0.000352	1
Cox7a2l	0.13374	2.48E-08	0.000799
Uqcr11	0.133707	4.15E-10	1.34E-05
Ilrun	0.13353	3.21E-11	1.04E-06
Atp13a1	0.13335	3.92E-12	1.27E-07
Tomm40	0.133025	4.08E-07	0.013171

Peli1	0.132999	0.000283	1
Bnip2	0.132642	4.53E-06	0.14614
Nsd3	0.132603	0.00041	1
Slc6a6	0.132598	1.1E-08	0.000355
Synj2	0.132197	1.99E-11	6.42E-07
Alg5	0.132123	2.55E-05	0.821869
Prpf40a	0.13174	1.28E-07	0.004126
Heatr5a	0.131525	4.86E-06	0.157035
Eed	0.131474	2.71E-06	0.087446
Chd1	0.131351	6.04E-08	0.001951
Hsph1	0.131225	1.59E-08	0.000512
Psen2	0.131224	9.23E-08	0.002981
Slc16a3	0.131008	0.000136	1
Mrto4	0.130653	1.56E-08	0.000503
Ripk1	0.130651	3.01E-10	9.71E-06
Srsf6	0.130636	3.48E-07	0.011229
Eif3m	0.130612	0.000175	1
Zdhhc3	0.130569	1.33E-07	0.004278
Ranbp1	0.130553	0.000107	1
Acvrl1	0.130486	2.83E-07	0.009145
Txn2	0.130479	1.02E-06	0.032793
Asb13	0.130285	4.19E-09	0.000135
Nap1l1	0.130198	3.98E-05	1
Ddx58	0.1301	3.19E-07	0.010299
Mrps18b	0.12991	4.99E-09	0.000161
Susd6	0.129749	9.46E-05	1
mt-Nd5	0.129669	3.97E-07	0.012824
Psmd12	0.129197	1.38E-05	0.445528
Sept9	0.129108	0.000289	1
Gm2000	0.128958	6.33E-14	2.04E-09
Cryba4	0.128901	1.67E-07	0.00539
Il4ra	0.128896	3.85E-07	0.012425
Ctla2b	0.128891	3.26E-11	1.05E-06
Chuk	0.128753	5.62E-08	0.001815
Emc4	0.12874	4.42E-06	0.142722
Ndufs3	0.128287	3.36E-08	0.001084
Pola1	0.128176	0.006905	1
Cbwd1	0.127946	3.03E-09	9.8E-05
Gla	0.127579	2.15E-17	6.95E-13
Pfn1	0.127495	0.000673	1
Usp30	0.127384	1.5E-17	4.84E-13
Pign	0.127178	2.64E-09	8.54E-05
Mcm6	0.126744	0.000964	1
Slc7a6	0.126712	1.16E-10	3.74E-06
Atp11b	0.126682	2.1E-07	0.006778
mt-Nd4	0.126425	2.52E-07	0.008146
Tbc1d10a	0.126405	1.13E-06	0.036608
Rpia	0.126403	4.4E-08	0.00142

Ube2d3	0.126353	2.62E-05	0.844889
Top1	0.126196	0.000133	1
Ubr4	0.126083	5.74E-07	0.018534
Zfp810	0.126061	6.81E-06	0.219879
Zfp593	0.125918	3.15E-10	1.02E-05
Ets2	0.125835	2.57E-05	0.829106
Sqstm1	0.125563	0.000915	1
Btg2	0.125392	0.054724	1
Rabggtb	0.125141	8.31E-09	0.000268
Aig1	0.124897	9.06E-09	0.000292
Gstcd	0.124711	9.05E-10	2.92E-05
Myh10	0.124686	8.88E-15	2.87E-10
Pi4k2a	0.124551	3.85E-08	0.001244
Cmpk2	0.124383	2.63E-22	8.5E-18
Stim2	0.124332	2.95E-10	9.54E-06
Calr	0.1241	0.000661	1
Tmem87b	0.124084	2.32E-05	0.749602
Pdia5	0.124073	3.58E-08	0.001155
Ndufa6	0.124067	2.43E-06	0.078591
Notch4	0.124021	7.12E-10	2.3E-05
Tmem67	0.124007	6.06E-12	1.96E-07
Mark2	0.123985	2.52E-05	0.813081
Zfp330	0.123796	1.43E-10	4.62E-06
Irs2	0.123598	1.61E-08	0.000521
Zc3h12c	0.123493	5.88E-09	0.00019
Zeb2	0.123355	3.11E-05	1
Rassf1	0.122898	2.42E-06	0.078225
Kctd5	0.122857	1.98E-09	6.4E-05
Snhg15	0.122821	1.08E-08	0.000348
Wdr12	0.122477	6.2E-07	0.02001
Mov10	0.122427	1.77E-07	0.005701
Tpcn2	0.122247	8.64E-13	2.79E-08
Impdh2	0.122148	5.48E-08	0.001768
Zfp622	0.122101	2.13E-05	0.68712
Dnajc15	0.121761	5.14E-06	0.165805
Rnasel	0.121365	5.56E-08	0.001794
Kdm7a	0.121286	5.25E-07	0.016965
Gtf2f2	0.121277	2.1E-07	0.006782
Orai1	0.121219	4.84E-06	0.156289
Safb2	0.121176	1.48E-06	0.047628
Snu13	0.121136	1.83E-05	0.590303
Erap1	0.121051	2.42E-06	0.078221
Reep4	0.120983	2.04E-05	0.658367
Atp6v1a	0.120828	0.000136	1
Utp14a	0.120745	4.46E-06	0.144131
Slc22a17	0.120567	7.42E-07	0.023961
Fgfr1op	0.120521	1.93E-09	6.24E-05
Cmc1	0.120503	5.55E-09	0.000179

Wdr55	0.120466	1.14E-07	0.003679
Dnajc3	0.120382	1.6E-07	0.005152
Jak3	0.120222	9.65E-10	3.11E-05
Keap1	0.119958	1.75E-05	0.565505
Abhd15	0.119882	0.261343	1
Elob	0.119842	0.000116	1
Sdf2l1	0.119841	5.39E-07	0.017397
Psmd14	0.119836	4.31E-06	0.139133
Taf10	0.119649	1.97E-07	0.006345
Pacsin2	0.119616	1.94E-07	0.006255
Serf2	0.119112	2.08E-06	0.06723
Daam1	0.1191	1.2E-12	3.88E-08
Nop16	0.119061	6.78E-10	2.19E-05
Abhd16a	0.119006	0.000177	1
Trim56	0.118951	3.07E-06	0.099131
BC147527	0.118804	6.02E-16	1.94E-11
St7	0.118777	3.97E-05	1
Tnfrsf14	0.118488	1.66E-09	5.35E-05
Cisd2	0.118228	5.67E-08	0.001831
Sec61b	0.118073	7.56E-08	0.002441
Abracl	0.117988	1.31E-05	0.421741
Resf1	0.117826	1.15E-06	0.037058
Batf3	0.117816	3.37E-07	0.010891
Zfyve26	0.11772	2.91E-07	0.009389
Plin3	0.117578	1.38E-12	4.44E-08
Tra2b	0.11757	1.29E-06	0.041649
Bag3	0.1175	3.51E-06	0.113405
Tomm70a	0.117499	3.51E-08	0.001132
Csnk2b	0.117377	0.00061	1
Masp1	0.117263	3.59E-15	1.16E-10
Ppp1r14b	0.117215	6.89E-09	0.000223
Gch1	0.117179	4.42E-12	1.43E-07
Abhd17a	0.117172	1.49E-07	0.004808
Sec61g	0.117093	8.42E-06	0.271857
Rrs1	0.117053	9.73E-07	0.031401
Nubp1	0.116947	3.52E-07	0.011357
Sidt2	0.116914	9.72E-08	0.003139
Tspan32	0.116729	1.53E-10	4.95E-06
Polr1d	0.116724	9.57E-07	0.030886
Rcn1	0.116686	1.75E-08	0.000565
Gmppb	0.116546	6.29E-09	0.000203
Nfkbid	0.116474	2.97E-05	0.958291
Rbbp8	0.116244	1.22E-06	0.039287
Atad2	0.116074	1.19E-05	0.385013
Noc4l	0.116028	7.35E-13	2.37E-08
Scarf2	0.115963	3.75E-10	1.21E-05
Ccdc25	0.115681	1.08E-08	0.000348
Bzw2	0.11535	8.31E-10	2.68E-05

Slc35b1	0.115272	5.48E-05	1
Eftud2	0.115163	4.68E-07	0.015117
Mrpl32	0.115139	7.85E-08	0.002534
Elf4	0.115134	0.000566	1
Bach2os	0.115109	5.25E-12	1.7E-07
AC113595.	0.115064	1.76E-11	5.68E-07
Cacybp	0.114979	1.07E-07	0.003453
Ist1	0.114839	6.7E-07	0.021639
Rap2b	0.114723	3.2E-10	1.03E-05
Gbgt1	0.114658	5.36E-14	1.73E-09
Pdss1	0.11459	1.09E-10	3.53E-06
Rab24	0.114589	7.39E-09	0.000239
Cyp51	0.114583	2.18E-07	0.007028
Tpp2	0.114173	4.01E-07	0.012949
Akr1b10	0.113985	0.000207	1
Snhg4	0.113839	1.1E-13	3.54E-09
Ccnl2	0.113598	8.3E-06	0.268091
Qars	0.113417	2.77E-07	0.008944
Incenp	0.113246	2.08E-05	0.671738
Adam9	0.113093	1.9E-07	0.006144
Tmco4	0.113077	1.27E-06	0.040946
Pcgf5	0.113011	2.74E-09	8.84E-05
Sf3b1	0.112951	7.66E-07	0.02474
Nfxl1	0.112706	1.27E-07	0.004115
Mafk	0.11262	2.81E-09	9.08E-05
Pdcd2	0.112556	1.07E-09	3.46E-05
Cnr2	0.112511	1.81E-09	5.84E-05
Dcp2	0.112448	3.99E-11	1.29E-06
Eno1b	0.112431	1.32E-08	0.000425
Ciao2a	0.112263	2.01E-05	0.648861
Elac2	0.112172	2.57E-07	0.008297
Mif4gd	0.111985	9.15E-06	0.295303
Pgap2	0.111979	1E-10	3.24E-06
Ndufb5	0.111951	7.19E-07	0.023203
Psmb1	0.111827	1.39E-06	0.044868
Arf6	0.111525	8.48E-06	0.273632
Txnrd1	0.111417	0.000406	1
Gtpbp4	0.111182	3.99E-06	0.128839
Anxa4	0.111138	7.44E-08	0.002402
Arih1	0.111121	1.56E-05	0.503955
Gm11808	0.111115	3.16E-06	0.101884
Tuba1c	0.111001	1.11E-05	0.357284
Azi2	0.11097	6.65E-07	0.02148
Tes	0.110898	1.4E-06	0.04519
Timm10b	0.110716	1.54E-07	0.00496
Arpc3	0.11043	0.000194	1
Rfc2	0.110411	1.88E-05	0.60539
Atox1	0.110355	1.3E-06	0.041816

Tcp1	0.110313	2.13E-05	0.686885
Vamp8	0.110286	0.000152	1
Jpt1	0.110199	2.93E-06	0.094512
B4galt7	0.110177	1.86E-07	0.005997
Dph5	0.109887	1.77E-07	0.005706
Gm11084	0.109886	5.91E-09	0.000191
Snrnp48	0.109856	0.000487	1
Get4	0.109833	2.29E-08	0.00074
Atp2b1	0.109715	0.000126	1
Xrn1	0.109539	1.95E-06	0.063088
Myo1c	0.109346	5.01E-06	0.161793
Mpi	0.109184	1.42E-05	0.458351
Atp5d	0.108989	6.26E-06	0.202263
Mospd2	0.108551	5.32E-08	0.001719
Irf2bp2	0.108512	0.000512	1
Afg3l2	0.108361	9.71E-06	0.313578
Ube2j2	0.108296	1.64E-07	0.005292
Irf8	0.108138	0.002038	1
Nipa2	0.108061	0.000101	1
Atp7a	0.107751	6.04E-06	0.194852
Gk	0.10765	7.14E-11	2.3E-06
Rabepk	0.107619	1.01E-10	3.27E-06
Pgap1	0.107618	2.79E-07	0.008995
Tbc1d14	0.107546	3.5E-06	0.112891
Cdk14	0.107532	2.06E-05	0.665788
Naa50	0.107441	4.57E-07	0.014767
Ddx50	0.107417	9.18E-05	1
Mthfd1l	0.107405	9.3E-06	0.300244
Themis2	0.107284	0.000645	1
Csrnp1	0.10728	0.000239	1
Sin3b	0.107235	0.000346	1
Mfsd10	0.10666	4.41E-07	0.014222
Ndufa12	0.106603	5.4E-09	0.000174
Atm	0.10654	1.07E-07	0.003456
Ncoa7	0.106226	1.88E-05	0.607113
Ddi2	0.106146	6.34E-07	0.020483
Ptpn1	0.106028	0.000465	1
Hcfc1r1	0.105984	0.000794	1
Sf3b3	0.105931	2.63E-05	0.849549
Tasor2	0.105771	9.56E-06	0.308676
Hspa9	0.105618	1.85E-05	0.598577
Pa2g4	0.10559	5.49E-05	1
Esyt2	0.105541	0.011207	1
Mia2	0.105523	1.92E-05	0.621326
Igbp1	0.105387	9.56E-10	3.08E-05
Stxbp2	0.105279	1.53E-09	4.92E-05
A330023F2	0.104999	3.28E-08	0.00106
Tmpo	0.104714	1.23E-06	0.039786

Elk4	0.104603	1.37E-05	0.441219
Fam49a	0.104508	5.74E-05	1
Psmc4	0.104372	1.4E-05	0.451504
1810013L2	0.104284	4.69E-05	1
Tra2a	0.104081	0.000283	1
Parl	0.10385	4.54E-06	0.146693
Srpk2	0.103834	3.14E-05	1
Sra1	0.103699	0.001334	1
Mre11a	0.103528	1E-06	0.032279
Tmbim6	0.103527	3.92E-06	0.126426
Dck	0.103435	3.18E-10	1.03E-05
Ppig	0.103434	7.73E-08	0.002497
Lclat1	0.103416	0.000259	1
Pld3	0.103227	0.002136	1
Ankrd16	0.103192	3.4E-08	0.001097
Mettl17	0.103093	1.92E-05	0.618517
Aimp1	0.102791	9.49E-09	0.000306
Rars	0.102734	5.22E-09	0.000168
Chd4	0.102664	0.000198	1
Mdn1	0.10254	4.62E-07	0.0149
Ndufa8	0.102433	3.19E-06	0.103136
Hnrnpab	0.102391	0.000986	1
Mocs1	0.102236	3.25E-07	0.010486
Ggnbp2	0.102037	2.34E-07	0.007539
Sdhd	0.101782	3.51E-07	0.011326
Uqcr10	0.101714	5.9E-06	0.190361
Cgas	0.101563	6.58E-05	1
Eif5b	0.101461	1.86E-08	0.000599
Tmbim4	0.101412	0.00044	1
Nufip2	0.101239	3.32E-05	1
Fam219a	0.101222	1.66E-05	0.535361
Ndufc1	0.101072	1.66E-07	0.005371
Runx1	0.100845	0.000156	1
Mrpl12	0.100761	3.26E-07	0.010528
Nfs1	0.100757	1.24E-05	0.399352
Mdh2	0.100751	2.56E-05	0.82766
Nemf	0.100584	7.93E-07	0.025595
Gtf2h1	0.100558	2.9E-08	0.000936
Sergef	0.100445	2.41E-06	0.077693
Mybbp1a	0.100328	0.000135	1
St5	0.100226	2.03E-06	0.065524
Birc2	0.100188	2.15E-06	0.069572
Supt16	0.100105	7.5E-05	1
Tubb4b	0.100036	9.25E-06	0.298606
Serbp1	0.099987	1.51E-05	0.485973
Rab22a	0.099736	2.7E-05	0.872881
Hmga1	0.099618	1.7E-10	5.48E-06
Myd88	0.099466	0.000124	1

Phb2	0.099446	5.88E-06	0.189866
Lypla2	0.099351	3.11E-05	1
Suco	0.099314	0.000421	1
Clint1	0.099286	8.17E-05	1
Dcun1d5	0.099229	2.06E-06	0.066653
Arhgef18	0.099195	0.01508	1
Eva1a	0.099147	0.000163	1
Smchd1	0.099076	0.00228	1
Farsb	0.098924	1.5E-07	0.004838
Fen1	0.098777	4.68E-05	1
Srek1	0.098678	0.001437	1
Cyb5b	0.098661	6.76E-08	0.002183
Cpeb2	0.09848	4.88E-05	1
Sppl2a	0.098446	0.000182	1
Gng10	0.098421	0.002212	1
Washc2	0.098377	4.55E-06	0.146745
Snhg17	0.098081	1.55E-09	5.01E-05
Calm3	0.098041	1.27E-05	0.410441
4933421O	0.098018	8.81E-10	2.84E-05
Fundc2	0.097874	2.1E-08	0.000676
Atp6v1b2	0.097845	3.33E-05	1
Adcy7	0.097796	1.25E-05	0.402898
Mecr	0.097693	6.57E-08	0.002122
Tomm7	0.097659	8.45E-06	0.272902
Stk24	0.097605	0.021622	1
Atg16l1	0.09736	0.000426	1
Tcerg1	0.097351	4.24E-05	1
Usp16	0.097246	6.99E-06	0.225784
Ston2	0.097135	8.23E-07	0.026582
Nlrp1b	0.096909	7.39E-08	0.002386
Gm12185	0.096706	1.01E-05	0.327645
Ddx21	0.096615	2.18E-05	0.703793
Banf1	0.096614	1.38E-06	0.044588
Slc30a6	0.096602	3.79E-05	1
Rnps1	0.096582	1.74E-05	0.563091
Rnf34	0.096031	5.25E-06	0.16962
Rlim	0.096007	0.000152	1
Llph	0.095887	4.62E-06	0.149277
Wtap	0.095877	2.91E-06	0.094084
Dnaja1	0.095476	1.76E-06	0.056839
Hdac2	0.095397	1.89E-08	0.000612
Hipk1	0.09537	7.51E-05	1
Slc11a2	0.0953	4.87E-06	0.157207
Nfrkb	0.095112	1.08E-05	0.347284
Gatad2a	0.094935	5.21E-06	0.168228
Apaf1	0.094852	4.66E-05	1
Ptpn2	0.094636	1.14E-05	0.367568
Rrp7a	0.094607	1.09E-06	0.035341

Ube2l3	0.094582	0.01725	1
Arf3	0.094557	8.21E-06	0.265188
Trim27	0.094365	9.38E-06	0.302859
Nedd8	0.094352	3.59E-05	1
Elovl5	0.094274	5.67E-05	1
Ndel1	0.094254	5.42E-05	1
Stx7	0.094254	9.04E-05	1
Hscb	0.094223	3.4E-08	0.001099
Lrrfip2	0.094183	5.66E-06	0.182794
Nme1	0.094092	5.72E-06	0.184778
Atad2b	0.094073	1.01E-06	0.032575
Srrd	0.09364	1.49E-08	0.000482
Ndufb7	0.093168	0.002058	1
Pus10	0.093099	6.63E-05	1
Cmklr1	0.093085	1.32E-05	0.426655
Psma1	0.09306	0.000307	1
Hps3	0.093041	0.000574	1
Ptpn11	0.092792	9.67E-07	0.031234
1500011B(	0.092738	1.12E-07	0.003624
Tecpr1	0.092692	0.029639	1
Pts	0.092576	0.000465	1
Pcsk7	0.092507	2.72E-07	0.008794
Shmt2	0.092404	1.71E-07	0.005523
Aif1	0.092251	0.00423	1
Insig1	0.092237	4.6E-09	0.000149
Cdk9	0.092198	1.28E-06	0.041321
Plk3	0.092096	4.12E-07	0.013299
Crybg3	0.092082	9.51E-06	0.306985
Phf5a	0.091862	0.004143	1
Snrpd1	0.091832	3.87E-07	0.012502
Rhoh	0.091569	0.009278	1
Parp3	0.091519	1.01E-08	0.000327
Psmb6	0.091391	0.014148	1
Mrpl57	0.091233	0.00086	1
Esd	0.091076	1.87E-05	0.602822
Bcl2l1	0.091044	0.000415	1
Maco1	0.090923	4.57E-06	0.147593
Zbtb11	0.09089	1.75E-06	0.056617
Tsta3	0.090801	2.58E-06	0.083236
Ndufs6	0.090748	6.13E-06	0.197848
Insyn2b	0.090566	0.000133	1
Slc7a5	0.09033	3.7E-09	0.00012
Sbno2	0.090315	3.68E-05	1
Prdx6	0.090265	2.97E-07	0.009593
Cotl1	0.090186	0.001493	1
Ormdl2	0.090106	1.1E-06	0.035387
Ccdc9	0.090086	1.83E-06	0.059067
Olfr111	0.090053	0.004501	1

Limd1	0.089946	1.84E-07	0.005955
Setd7	0.08993	2.4E-08	0.000776
Ddx27	0.08982	1.6E-05	0.517086
Ubfd1	0.089812	1.96E-08	0.000634
Galns	0.089734	5.58E-10	1.8E-05
Trnau1ap	0.089633	1.21E-07	0.003908
Glcci1	0.089616	0.000328	1
Nars2	0.089495	3.87E-06	0.12504
Timm13	0.089279	7.23E-05	1
Nol10	0.08908	4.92E-05	1
Ifngr2	0.089064	0.002485	1
Fars2	0.088602	3.3E-06	0.106584
Ppt2	0.08849	0.000115	1
Ndufaf6	0.088437	1.34E-08	0.000432
Fndc3a	0.088378	0.006213	1
Mapk1ip1l	0.088369	2.45E-05	0.790047
Mrps9	0.088151	1.46E-05	0.470639
Mvb12a	0.088098	2.98E-07	0.00961
Cdk6	0.088053	0.01018	1
Wdr41	0.087999	1.91E-07	0.00617
Riok3	0.08784	2.91E-05	0.938131
Wdr74	0.087619	5.11E-06	0.164923
Nt5m	0.087595	1.59E-05	0.513859
Ino80	0.087475	1.29E-09	4.16E-05
Pdhb	0.087439	0.000196	1
Smg5	0.087361	4.78E-10	1.54E-05
Nr2c2ap	0.08727	1.27E-07	0.004101
Sept6	0.087265	0.000449	1
Limk1	0.087107	0.000189	1
Heatr1	0.08699	1.07E-05	0.345777
Sltm	0.086957	4.21E-05	1
S100a1	0.086956	1.15E-08	0.000371
Thoc1	0.086869	0.00041	1
Hhat	0.086805	3.51E-06	0.113216
Pdia3	0.086753	0.000183	1
Magohb	0.086727	2.14E-09	6.9E-05
Lsm4	0.086633	2.52E-05	0.814015
Scand1	0.086631	3.54E-05	1
4932438A1	0.086622	4.51E-06	0.145574
Slc15a3	0.08662	6.46E-06	0.208481
Rp9	0.086506	0.000516	1
Anxa11	0.086499	0.004867	1
Selenoh	0.086443	3.04E-06	0.098089
Tle5	0.086391	2.06E-05	0.666593
Eef1akmt4	0.086316	3.15E-11	1.02E-06
Mpeg1	0.086284	0.000154	1
Dnaaf3	0.086259	2.92E-11	9.44E-07
Pex16	0.086139	2.74E-07	0.008856

Nin	0.086066	5.73E-06	0.184974
Cluh	0.085917	2.66E-11	8.59E-07
Pgp	0.085733	1.19E-05	0.383497
Kif5b	0.085722	0.000651	1
Dlst	0.085651	6.23E-05	1
Zfp367	0.085641	7.52E-07	0.024275
Ftsj3	0.085519	1.45E-07	0.004677
Ddost	0.085489	0.001489	1
Thoc6	0.085417	5.04E-10	1.63E-05
Trit1	0.085401	2.35E-05	0.759996
Rabl6	0.085353	0.000348	1
Coq2	0.085323	1.72E-07	0.005567
Ndufa2	0.085312	9.34E-05	1
Gm17106	0.085306	3.47E-06	0.112105
Rnf115	0.085271	0.000633	1
Riox1	0.085102	4.04E-06	0.130328
Rad50	0.08488	1.35E-05	0.435114
Spata6	0.084826	0.000407	1
Srsf10	0.084809	0.000143	1
Relch	0.084765	4.33E-05	1
Sertad1	0.084762	8.05E-06	0.259889
Cdk11b	0.084716	1.45E-06	0.046962
Psat1	0.084568	1.36E-05	0.440656
Setd3	0.084409	0.000125	1
Txndc5	0.084336	1.91E-07	0.006167
Naa20	0.084292	5.01E-05	1
Upf3b	0.084287	0.000421	1
Mrpl52	0.084178	0.000278	1
Bmyc	0.08409	0.013777	1
Peli2	0.083944	0.000538	1
Cdv3	0.083914	0.003492	1
Scly	0.083858	1.4E-05	0.453154
Creb3	0.083795	0.001365	1
Tlk1	0.08362	8.52E-06	0.275227
Pes1	0.083557	3.79E-07	0.012241
Ap2s1	0.083388	5.78E-06	0.18651
Tmem128	0.083364	8.49E-05	1
Sem1	0.08317	0.000178	1
Ube2w	0.083158	0.000136	1
Mrpl33	0.083157	7.85E-05	1
Eno1	0.083018	0.001024	1
Psmb7	0.082937	0.000636	1
Zfp945	0.08293	2.03E-07	0.006544
Tomm6	0.082743	0.000296	1
4933412E1	0.082652	4.15E-09	0.000134
Wdr36	0.082647	6.86E-05	1
Huwe1	0.082555	0.000627	1
Atad3a	0.082549	6.21E-07	0.020037

Med1	0.08246	0.000117	1
Tiparp	0.082254	7.77E-06	0.250758
Lamtor5	0.082242	9.78E-06	0.315859
Mak16	0.082238	0.000127	1
Rev3l	0.081974	9.72E-06	0.313732
Uimc1	0.081946	0.003028	1
Ndufb6	0.081936	0.000563	1
Ddx10	0.081842	5.78E-06	0.186632
Tmed1	0.081133	1.66E-08	0.000537
Fkbp1a	0.080932	3.01E-05	0.973387
Birc6	0.080915	0.000806	1
Ndufs5	0.080877	0.000718	1
Mrps16	0.080869	9.99E-05	1
Tiam1	0.080852	0.000478	1
Prrc2c	0.080732	0.002657	1
Grwd1	0.080713	1.65E-06	0.053374
Tut7	0.080706	0.00012	1
Atg2a	0.080679	0.003045	1
Tec	0.080421	0.000677	1
Szrd1	0.080344	2.99E-05	0.964195
Batf	0.080264	1.9E-10	6.14E-06
Igf2bp3	0.080252	1.99E-11	6.43E-07
Kri1	0.080242	3.87E-07	0.012492
Mydgf	0.080121	0.000428	1
Ubp1	0.08007	4.62E-08	0.001492
Trib1	0.08007	0.000521	1
Cast	0.080034	0.001142	1
Arf5	0.079981	2.27E-05	0.73239
Pum3	0.079875	1.08E-06	0.034842
Psma6	0.079871	4.11E-05	1
Nrd1	0.079776	2.55E-06	0.082372
Gm43813	0.079768	8.41E-08	0.002716
Sertad2	0.079642	1.9E-06	0.06142
Tfpi	0.079625	0.003383	1
Swt1	0.07961	0.000308	1
Stx8	0.07948	0.000414	1
Ttll5	0.079476	2.54E-06	0.082131
Cops9	0.079021	1.03E-06	0.033206
Tmem97	0.079001	1.43E-06	0.046318
Srsf11	0.078992	0.000325	1
Med21	0.078964	6.82E-05	1
Fdps	0.078863	0.000236	1
Abca7	0.078837	0.000226	1
Gar1	0.078792	3.35E-06	0.108062
Rabgef1	0.078753	0.000309	1
Parp4	0.078629	0.000346	1
Rps27l	0.07858	0.000198	1
Slc20a1	0.078555	0.000491	1

Myl6	0.078411	0.000177	1
Tent5c	0.078355	0.045067	1
Mob3c	0.078327	0.000146	1
Snrpd3	0.078223	0.000181	1
Fos	0.078133	1.74E-06	0.056131
Tyms	0.07809	0.028414	1
Lsm14a	0.07807	0.000298	1
Gm15283	0.07803	0.001498	1
Slc25a38	0.077999	7.31E-07	0.023613
Mta3	0.077884	0.002341	1
Chd8	0.077827	0.015921	1
Cops7a	0.077795	0.000132	1
Cog4	0.077641	0.000148	1
Ipo7	0.077639	0.008315	1
Cox17	0.077256	0.001586	1
Arel1	0.07718	2.92E-06	0.094171
Psmc6	0.077105	0.00229	1
Tbcd	0.077027	0.00013	1
Wrn	0.077019	7.79E-05	1
Rnf168	0.076971	0.000423	1
Mycbp2	0.076958	0.002633	1
Acy1	0.076926	1.65E-06	0.053252
Vdac2	0.076898	0.004128	1
Usp21	0.076826	0.000311	1
Eif2ak4	0.076821	1.4E-05	0.451387
Klhl2	0.07682	4.85E-09	0.000157
Myo1g	0.076786	7.44E-09	0.00024
Ten1	0.076712	0.00013	1
Thrap3	0.076616	0.001026	1
Lztfl1	0.076532	0.001804	1
Supt20	0.076354	0.002334	1
Tmem181a	0.076353	0.002368	1
Htatip2	0.076172	1.06E-10	3.42E-06
Ccar1	0.076042	9.69E-05	1
Sept11	0.076036	0.121353	1
Cd34	0.075983	2.5E-06	0.080866
Cdt1	0.075918	0.001665	1
Ywhag	0.075676	0.012214	1
Mapk6	0.075662	0.000182	1
Fhad1	0.075632	7.46E-06	0.240964
Atrx	0.07547	9.65E-06	0.311419
0610030E2	0.075424	0.0005	1
Timm17a	0.075393	0.0037	1
Tmem242	0.075367	2.92E-07	0.009421
Nop58	0.075164	2.06E-05	0.663547
Myo9a	0.075136	0.000118	1
Cyth1	0.075088	4.23E-05	1
Ltv1	0.074986	8.39E-07	0.027099

Slc38a1	0.07497	0.001265	1
Prune2	0.074872	3.1E-06	0.099997
Lmbr1l	0.074866	0.000105	1
Phb	0.074833	0.000166	1
Arl6ip4	0.074817	0.000277	1
Kdm4b	0.074807	1.17E-05	0.378684
Atg7	0.074701	1.68E-06	0.054127
Epb41	0.07467	0.003593	1
Wdr75	0.074632	0.000446	1
Dera	0.074406	0.000224	1
Zkscan6	0.074305	1.83E-05	0.59147
Fdx2	0.074277	3.56E-07	0.011485
Cyp2r1	0.074205	9.74E-07	0.031435
Ccdc58	0.074093	0.002876	1
Myl12a	0.074017	0.000809	1
Vti1a	0.073973	0.000497	1
Pbdc1	0.073871	6.91E-07	0.022322
B3gntl1	0.073746	0.00097	1
Inpp5b	0.073686	0.000473	1
Mad2l2	0.073654	5.19E-05	1
Fbrs	0.073597	0.000134	1
Meis3	0.073484	0.000467	1
Prpf38a	0.073396	5.34E-07	0.017226
Urm1	0.073394	4.05E-06	0.130877
Dctpp1	0.073387	9.98E-05	1
Nup153	0.073328	3.07E-06	0.099238
Kmt2a	0.073285	0.000846	1
Jak2	0.073211	5.12E-06	0.165245
Cracr2a	0.073156	0.004334	1
Chchd1	0.072851	5.7E-06	0.183869
Itpk1	0.072766	0.000331	1
Stat3	0.072675	0.002833	1
Arrdc4	0.072618	7.24E-07	0.023386
Snrpa1	0.072596	2.79E-06	0.089918
Srp9	0.072451	0.025417	1
Ugcg	0.072372	5.36E-05	1
Gltp	0.072297	0.003356	1
Nle1	0.07223	1.52E-05	0.489728
Becn1	0.072223	0.000215	1
5430427O	0.072179	1.29E-05	0.415564
Ikzf2	0.072123	5.16E-05	1
Gadd45gip	0.072011	0.00011	1
Fam89a	0.071957	3.65E-08	0.00118
Rab8a	0.07189	0.01175	1
Stx12	0.071786	2.69E-05	0.868103
Gm42031	0.071578	0.000171	1
Mettl6	0.071425	9.61E-06	0.310358
Nasp	0.071415	0.002947	1

Tmem39a	0.071348	1.85E-05	0.598154
H2afz	0.071277	0.028301	1
Uqcrb	0.071277	0.000167	1
Ssr3	0.071252	0.006064	1
Mcph1	0.071185	0.002704	1
Notch1	0.071125	0.001023	1
Rpf2	0.071049	1.5E-06	0.048322
Ell2	0.071011	3.22E-05	1
Lsm12	0.070919	0.002744	1
Ctdnep1	0.070649	0.000306	1
B230219D:	0.070603	2.8E-05	0.903113
Srsf3	0.070576	0.064053	1
Rnf4	0.070573	0.014397	1
Bcat2	0.070534	7.9E-07	0.025516
Srpk1	0.07032	0.008412	1
Pik3r6	0.070242	0.000175	1
Ccdc84	0.070209	6.01E-07	0.019417
Rsl24d1	0.070188	3.4E-05	1
Ei24	0.070173	2.15E-05	0.694419
Manba	0.070166	0.000788	1
Agpat5	0.070066	4.7E-06	0.151804
Bola3	0.069971	5.28E-11	1.71E-06
Mrpl20	0.069783	1.31E-05	0.422401
Fam222b	0.069693	4.34E-05	1
Phc3	0.069642	0.062515	1
Ndor1	0.069617	0.00012	1
Ywhab	0.069512	0.014835	1
Plaa	0.069465	1.25E-06	0.040269
Aurkaip1	0.069333	1.1E-05	0.355838
Cacnb2	0.069323	0.000332	1
Eps15	0.069276	0.001117	1
Exosc1	0.069251	7.73E-07	0.024945
Lsg1	0.069192	0.000158	1
Igf1r	0.06915	0.030218	1
Hipk2	0.069138	5.99E-06	0.193268
Tmem123	0.069053	2.78E-08	0.000899
Tma7	0.06894	0.003988	1
Tecpr2	0.068885	5.2E-10	1.68E-05
Atic	0.068848	0.009816	1
Nol12	0.068637	1.95E-06	0.062893
Ube2s	0.068424	0.000247	1
Aco2	0.068398	4.14E-05	1
Msh3	0.068393	9.23E-06	0.298087
Aldh18a1	0.068226	1.48E-05	0.477415
Dhps	0.068099	9.73E-08	0.003142
Il13ra1	0.068014	0.001119	1
Utp3	0.067873	0.000293	1
Strn4	0.06784	8.04E-05	1

Osbpl8	0.06783	0.007786	1
Vipas39	0.067817	0.000255	1
Agfg2	0.067549	4.95E-05	1
Utp4	0.06753	8.1E-09	0.000261
Slc25a39	0.067503	0.001046	1
Ctu2	0.067416	4.59E-05	1
Nolc1	0.067321	0.000245	1
Tcirg1	0.067189	0.000658	1
Arap2	0.067148	0.001109	1
Med15	0.067121	1.11E-05	0.359932
Aak1	0.067075	0.555672	1
Cmas	0.066959	0.000106	1
Morf4l2	0.066908	0.035502	1
Nip7	0.066838	0.000114	1
Rpe	0.066838	2.06E-05	0.663472
Cyb5r4	0.06676	0.001208	1
Eif1ad	0.066733	2.04E-05	0.659964
Tnip1	0.066726	0.000114	1
Ddx46	0.066645	0.000622	1
Rlf	0.066558	0.019241	1
Kdm6b	0.066479	0.101761	1
Trap1	0.066417	0.00522	1
Eif2b5	0.066332	5.42E-05	1
Mical1	0.06631	0.005295	1
Foxo1	0.066251	0.392785	1
Cndp2	0.066169	0.000647	1
Ints9	0.066087	8.35E-06	0.269683
Serpinb9	0.065928	0.000104	1
Dnajc11	0.065806	5.59E-05	1
Cdc37	0.065793	0.004246	1
Rngtt	0.065792	0.007838	1
Baiap2	0.065712	0.000945	1
Paox	0.065708	8.1E-06	0.261537
Nubp2	0.065706	1.03E-06	0.033225
Dusp11	0.065628	0.000289	1
Nup98	0.065616	0.000237	1
Psmb3	0.065607	0.000901	1
Rab29	0.065596	1.35E-05	0.436895
Sult1a1	0.065474	0.609741	1
Nop9	0.065445	0.000313	1
Ap1g2	0.065202	0.000162	1
Gfer	0.065188	0.002063	1
Prdx2	0.065174	0.00011	1
Tgs1	0.065087	3.69E-05	1
Dhodh	0.065081	4.34E-07	0.014004
Ap1s1	0.064904	0.004877	1
Osgin2	0.064868	2.66E-05	0.858292
Tg	0.064835	0.015173	1

Ankrd17	0.064753	0.00313	1
Aff1	0.064711	0.002995	1
Bri3	0.064644	4.77E-08	0.00154
Ndufa7	0.064616	0.000784	1
Slc9a8	0.06455	6.01E-05	1
Rbx1	0.064527	0.012899	1
Nupl2	0.064522	2.43E-06	0.078402
Ddx24	0.064456	9.55E-05	1
Atg4a	0.064421	0.000214	1
Msto1	0.064397	0.000196	1
Snhg8	0.064191	3.91E-05	1
Tbk1	0.064186	0.000981	1
Tsr3	0.064141	0.002939	1
Ethe1	0.064037	5.32E-06	0.171808
Abcf1	0.063993	0.000456	1
D530033B1	0.063992	3.04E-06	0.098296
Jade2	0.063902	0.017427	1
Ahctf1	0.063874	0.000733	1
Dhdh	0.063661	1.23E-05	0.395738
Lsm5	0.063646	0.013436	1
Gmeb2	0.063532	0.006014	1
Mrpl21	0.063462	2.51E-05	0.811508
Zfp655	0.063409	0.002577	1
Map2k7	0.063382	1.09E-05	0.350441
Kat2b	0.063244	0.000156	1
Wdr18	0.063185	3.63E-05	1
Aph1a	0.063087	0.00032	1
Ndufa11	0.063073	0.001085	1
Foxj3	0.06306	0.000112	1
CAAA0111	0.063038	2.58E-05	0.832074
Ogfrl1	0.063027	0.004316	1
Ears2	0.062931	0.017947	1
Nat10	0.062921	1.12E-05	0.361867
Srp14	0.062839	0.002758	1
Tmem268	0.062705	0.00072	1
Slc10a7	0.062655	0.030846	1
Ankrd12	0.062629	0.001545	1
Timm9	0.062432	0.00579	1
Rrp15	0.062424	5.56E-06	0.179519
Ppm1g	0.062235	0.00939	1
Mrpl18	0.062196	3.21E-06	0.103547
Ptges2	0.062194	7.96E-05	1
N4bp1	0.062108	0.001704	1
Hnrnpa3	0.062087	0.000515	1
Arfgef1	0.062085	0.001585	1
Tmem189	0.06206	0.004717	1
Ascc3	0.06191	0.003762	1
Gm20275	0.061695	6.93E-05	1

Acd	0.061682	4.83E-05	1
Dnaja2	0.061643	0.001783	1
Med14	0.061582	0.002976	1
Tmem164	0.061484	0.000436	1
Nufip1	0.061478	0.000239	1
Zfp36l2	0.061376	0.743397	1
Vwa8	0.06135	2.22E-05	0.718317
Ptprj	0.061311	0.002481	1
Ivd	0.061083	0.000974	1
Mthfd2	0.061069	8.82E-06	0.284614
Klf2	0.060896	7.11E-07	0.02297
Ero1l	0.060896	4.88E-06	0.157392
Nsun6	0.060884	0.074854	1
Pms2	0.060878	0.009094	1
Tsr1	0.060788	4.11E-05	1
Bop1	0.060698	1.99E-05	0.643017
Mafb	0.060548	0.269344	1
Pold4	0.06051	0.000713	1
Ipo4	0.060454	0.004925	1
Lsp1	0.060211	0.141901	1
Manf	0.060069	0.004041	1
Mppe1	0.059885	0.000797	1
Slc39a14	0.05973	1.32E-05	0.426517
Etnk1	0.059687	0.005301	1
Ppp1r12c	0.059674	7.05E-05	1
Inpp4a	0.059641	8.17E-06	0.263849
Zc3h13	0.059639	6.54E-07	0.021109
Mpp6	0.059465	0.000135	1
Cep78	0.059336	0.000669	1
Snrnp35	0.059326	0.000104	1
Pno1	0.059299	2.39E-05	0.772989
Fam3c	0.059294	0.001174	1
Mdm2	0.059281	0.060758	1
Mfsd5	0.059153	0.002206	1
Slc35b3	0.059139	0.000173	1
Nckipsd	0.058788	0.002399	1
Src	0.058656	2.26E-06	0.073017
Ascc2	0.058655	4.14E-06	0.133737
Mrpl2	0.058622	0.000268	1
Stx18	0.058614	0.000152	1
Fyb	0.05857	0.019247	1
Lrrc4	0.05855	0.000149	1
Tmed8	0.058546	0.00016	1
Prkx	0.058453	0.011631	1
Rer1	0.058336	0.000183	1
Mrpl23	0.058258	0.000378	1
Gm16286	0.058195	0.004699	1
Fam162a	0.058193	7.41E-07	0.023922

Tet2	0.058157	0.004037	1
Ppif	0.058154	3.51E-05	1
Itpr3	0.058093	8.8E-05	1
Rnft1	0.057982	0.001302	1
Dazap1	0.057868	0.003373	1
Erp44	0.057867	0.000514	1
Slc35d2	0.057819	8.73E-05	1
Ints6	0.057792	5.12E-05	1
Slc12a7	0.057754	6.26E-05	1
Wapl	0.057724	0.002296	1
2610507B:	0.057666	0.00018	1
Rnaseh2b	0.05765	9.87E-06	0.318751
Snap23	0.057636	0.001058	1
Zmiz2	0.057611	0.00133	1
Tspan13	0.057582	0.374455	1
March2	0.057546	2.95E-05	0.953624
Rab8b	0.057507	0.051483	1
Rbck1	0.057344	0.003138	1
Nipsnap3b	0.057326	0.0002	1
Pphln1	0.057325	0.000216	1
Fbxw2	0.057233	0.004733	1
Ncf4	0.057215	0.019867	1
Snx1	0.057194	0.000679	1
Uhrf1bp1	0.057154	1.31E-09	4.24E-05
Golga4	0.057133	0.000123	1
Pgls	0.057075	0.001514	1
Commd4	0.057038	0.002124	1
Trim12c	0.057009	0.000389	1
Dhcr7	0.056948	3.41E-06	0.110004
Pycard	0.05688	0.056705	1
Mink1	0.056768	0.013618	1
Stk19	0.056729	0.000277	1
Akt3	0.056681	2.6E-05	0.839509
Hmbs	0.056492	0.001266	1
Trmt61a	0.056465	0.000433	1
Tmsb4x	0.056432	0.41423	1
Trappc6a	0.056397	0.000308	1
Mob1b	0.05639	0.000779	1
Dnajb1	0.05637	0.004448	1
Dkc1	0.056348	8.3E-05	1
Tmem167	0.056323	0.010761	1
Ambra1	0.056246	0.00509	1
Pde4a	0.056159	7.19E-05	1
Nus1	0.05614	4.57E-05	1
Mtmr14	0.05612	0.00038	1
Rab32	0.056083	0.010123	1
Ttc27	0.05603	0.000506	1
Mrpl45	0.055741	0.008591	1

Hgsnat	0.055716	0.000285	1
Cyp4v3	0.055631	0.019302	1
Tox4	0.055622	0.000138	1
Eif3j1	0.055603	0.001008	1
Ppm1b	0.055573	0.355813	1
Gipc1	0.055559	0.000428	1
Arv1	0.055515	3.88E-07	0.012543
Plekha5	0.055477	0.000535	1
Cct6a	0.055335	0.086295	1
Trub2	0.05521	0.000814	1
Tor1b	0.055059	0.011674	1
Rrp9	0.05493	1.57E-05	0.505286
Lmnb1	0.054875	0.000118	1
Odf2	0.054781	0.006758	1
Nol9	0.054737	0.000952	1
Churc1	0.054713	0.000417	1
Trmt10c	0.054652	0.003414	1
Sik3	0.054635	7.63E-05	1
Snx6	0.054632	0.010369	1
Bcl6	0.054615	0.031822	1
Spout1	0.054551	8.71E-05	1
Mri1	0.054512	5.73E-06	0.185013
St8sia1	0.054411	1.59E-06	0.051249
Micos10	0.054315	0.005342	1
Utp18	0.054255	2.37E-06	0.076519
Elof1	0.05422	0.000119	1
Gdap2	0.05413	0.000275	1
Rnf180	0.054034	0.002051	1
Serhl	0.053959	0.000538	1
Triobp	0.053909	0.001075	1
Smdt1	0.053849	0.000199	1
Tent4b	0.053842	0.000485	1
Cox14	0.053801	0.000995	1
Ddx39b	0.053542	0.001731	1
Guk1	0.05349	0.000139	1
Mbp	0.053406	0.000411	1
Rras	0.053358	3.96E-07	0.012773
Polr3c	0.053346	0.005439	1
Acaca	0.053078	1.96E-05	0.631857
Lrrc8d	0.053035	4.89E-05	1
Nt5c3	0.052991	0.000607	1
Sart3	0.052833	0.001649	1
Ints12	0.052799	0.001646	1
Iars2	0.052736	0.002046	1
Zc3h15	0.052728	0.034512	1
Jtb	0.052659	0.017005	1
Cope	0.052626	0.292504	1
Mrps10	0.052615	0.001124	1

Ccz1	0.052566	0.003286	1
Baz1a	0.052507	0.000343	1
Pdcd5	0.052452	0.012167	1
Pitpnm1	0.052343	0.000789	1
Heatr6	0.052318	0.001579	1
3110082I1	0.052241	0.007235	1
Tfdp1	0.052241	0.004388	1
Selenoo	0.052112	0.001526	1
Dnajc17	0.052016	0.00076	1
Alg3	0.051945	0.000153	1
Agpat2	0.05188	2.05E-06	0.066262
Raet1e	0.051865	0.00021	1
Ece1	0.051863	0.00112	1
Mrpl35	0.051737	0.000228	1
Lnpep	0.051644	0.020021	1
Arhgdib	0.051637	0.058643	1
Fam111a	0.051605	0.000168	1
Cyld	0.051499	0.001909	1
Nln	0.051448	5.49E-05	1
Ewsr1	0.051438	0.002544	1
Ppm1m	0.05134	0.000435	1
Brms1	0.051336	0.000124	1
Nsun2	0.05116	0.003632	1
Ube2v1	0.051084	0.015345	1
Esyt1	0.051053	0.029408	1
Adpgk	0.050916	2.44E-05	0.788346
Cbx5	0.050884	0.000637	1
Cdyl2	0.050783	0.000521	1
C1rl	0.050724	6.04E-05	1
Ube2f	0.050537	0.003903	1
Rnpep	0.050487	0.012965	1
Hlx	0.05048	2.2E-05	0.711406
Elf2	0.050439	0.006104	1
Herpud1	0.050333	0.202961	1
Yars2	0.050104	0.000862	1
Nploc4	0.050027	6.3E-05	1
Taf13	0.049885	4.01E-06	0.129362
Plagl2	0.049826	0.00101	1
Bloc1s2	0.04982	0.008454	1
Cdk12	0.049646	0.021624	1
Bin3	0.049634	0.000106	1
Tars2	0.049476	0.000521	1
Cd2bp2	0.049459	0.000972	1
Prpf39	0.049438	0.000994	1
Tmem126a	0.049337	0.019907	1
Pcyt2	0.049322	0.012313	1
Fam104a	0.049302	0.002694	1
Mxd1	0.049295	0.005227	1

Lrpprc	0.04918	0.018566	1
Pgm1	0.049118	0.000604	1
Smc2	0.049108	2.31E-05	0.747323
Ube2d1	0.049091	0.000571	1
Cwc27	0.049066	2.23E-05	0.720518
Ptprc	0.049012	0.381522	1
Mms19	0.048892	0.001545	1
Spryd7	0.048841	0.034775	1
Prpf4	0.048729	3.93E-05	1
Os9	0.048631	0.006568	1
Usp3	0.048608	0.061485	1
Nudt21	0.048551	0.002502	1
Ube2g2	0.048524	0.000891	1
Soat1	0.048516	0.016799	1
Pola2	0.048505	0.00144	1
Mier1	0.048428	0.00124	1
Arid4b	0.048349	0.019726	1
Ern1	0.048269	0.002912	1
Kars	0.04822	0.000202	1
Mcoln1	0.047915	6.25E-05	1
Ttc7b	0.047884	0.095799	1
Prelid3b	0.04788	0.013112	1
Sp4	0.047839	0.019237	1
Pgam5	0.047825	0.002523	1
Mapk9	0.047811	5.41E-07	0.017456
Rbm28	0.047789	0.008606	1
Rap1gap2	0.047693	0.070225	1
Mrpl15	0.047636	0.001158	1
Mitf	0.047587	0.057552	1
Eef1e1	0.047567	0.00659	1
Dap3	0.047561	0.000637	1
Jmjd6	0.047502	0.002774	1
Ncor1	0.047472	0.055344	1
Taf3	0.047414	0.014824	1
Chchd4	0.047365	0.000643	1
Ildr1	0.047235	0.006788	1
Tmem87a	0.047213	0.000223	1
Vapb	0.04721	0.001203	1
Psmd1	0.047186	0.000258	1
Eif1ax	0.047147	0.000673	1
Def6	0.047139	0.038964	1
Htatsf1	0.047101	0.000527	1
Trim30c	0.046947	0.014514	1
Vps50	0.046894	0.007377	1
Hook2	0.046827	0.00013	1
Slamf9	0.046725	0.000585	1
Acsl5	0.046714	0.009263	1
Pkig	0.046575	0.011445	1

Cenpx	0.046479	0.004743	1
Brix1	0.046434	0.000408	1
Glb1	0.046393	0.006002	1
Znhit6	0.046361	0.005032	1
Mrpl11	0.046204	0.016089	1
Acox3	0.046202	0.200774	1
Zdhhc12	0.046191	0.000228	1
Cmip	0.046113	2.8E-05	0.905447
Afg1l	0.045927	0.00798	1
Pkn2	0.045926	0.0015	1
Glyr1	0.045909	0.137056	1
Acin1	0.045894	0.014237	1
Ndufaf4	0.045792	0.016351	1
Traf3	0.045726	0.000484	1
Irak1	0.045558	0.000331	1
Limk2	0.045545	0.04766	1
Acbd6	0.045542	0.002978	1
Akap9	0.045452	0.005359	1
Gm17018	0.045434	0.000214	1
Nadk	0.045405	0.032619	1
Shfl	0.045363	0.021087	1
Hpn	0.045339	6.23E-06	0.201097
Psmd9	0.045283	0.000389	1
Scaf4	0.045201	0.000463	1
Gins1	0.045188	8.82E-05	1
Eif4e2	0.04518	0.000394	1
Heatr3	0.045132	0.000879	1
Mtif2	0.045058	0.026139	1
Dtymk	0.045032	0.019642	1
Grsf1	0.044738	3.54E-06	0.114187
Sdhaf2	0.044644	0.000705	1
Cct7	0.044639	0.03922	1
Sh3bgrl	0.044584	0.00104	1
Pitrm1	0.044565	0.004517	1
Zcchc7	0.044527	0.019438	1
Snrpf	0.044458	0.001687	1
Naa38	0.044438	0.00058	1
Gab3	0.044438	0.27786	1
Wdr3	0.044212	0.003526	1
Polr2f	0.044168	0.001225	1
Secisbp2	0.044137	0.055323	1
Fam217b	0.044032	0.002286	1
Rnf121	0.04401	0.001391	1
C2cd5	0.044001	0.102674	1
Fam13b	0.043989	0.002771	1
D130040H	0.043874	0.00033	1
Gabpb2	0.043861	0.200355	1
Pnpla7	0.043821	0.691004	1

Riox2	0.043758	0.000433	1
Usp7	0.043727	0.000496	1
Nif3l1	0.043636	0.000905	1
Chfr	0.043562	0.006632	1
Zfand2a	0.043542	0.00056	1
Inafm1	0.043501	0.266603	1
Pdia4	0.043465	0.03604	1
Nfkb2	0.043331	0.000176	1
Gtf2h5	0.043307	0.000563	1
Cep164	0.043298	0.00074	1
Ep400	0.043288	0.000463	1
Prmt1	0.043238	0.01357	1
Fermt3	0.043212	0.093431	1
Hltf	0.043209	0.004717	1
Zfp64	0.043182	0.051321	1
Mtch2	0.043153	0.005851	1
Trak2	0.04305	0.000902	1
Twf1	0.043029	0.007517	1
Fbxo6	0.043008	0.011939	1
Ppid	0.042888	0.004818	1
Tcf20	0.042868	0.05343	1
Nifk	0.042761	0.003664	1
Uchl5	0.042716	0.00731	1
Rnf19a	0.042689	0.388682	1
Mea1	0.042672	0.001087	1
Tex10	0.042491	0.000325	1
Fbxo34	0.042485	0.000307	1
Speg	0.042476	0.00016	1
Slc36a4	0.042475	0.009963	1
Tbl3	0.04242	0.006147	1
Enpp4	0.042275	0.000276	1
Zdhhc13	0.042267	0.011433	1
Pon2	0.042189	0.215669	1
Dynll2	0.042167	0.031447	1
Mtfmt	0.042121	0.000528	1
Msra	0.041993	0.000366	1
Trmt6	0.041943	0.057034	1
Eif2b3	0.041801	0.004385	1
Poli	0.041785	7.6E-05	1
Cep162	0.041732	0.00812	1
Stoml1	0.041727	0.000599	1
Tomm5	0.041707	0.000185	1
Slc25a19	0.041677	0.00038	1
Pkib	0.041671	0.001149	1
Adora3	0.041669	0.012382	1
Rae1	0.041659	0.019893	1
Dtnbp1	0.041589	0.000195	1
Prpf31	0.041579	0.000966	1

Zpr1	0.041336	0.002136	1
Zfp992	0.041277	2.63E-05	0.849385
Btbd19	0.041261	0.021967	1
Pinx1	0.041257	2.86E-05	0.921776
Trmo	0.041242	0.001615	1
Sco2	0.041162	0.010533	1
Clec5a	0.041137	0.062432	1
Dus2	0.041029	0.241813	1
Paf1	0.040968	0.004672	1
Dmxl2	0.040878	0.000267	1
Sae1	0.040833	0.142243	1
Tmub1	0.040829	0.00019	1
Elp1	0.040809	0.006846	1
Trip12	0.040715	0.011593	1
Ccdc82	0.040623	0.049523	1
Adprm	0.040617	0.004841	1
Fam120a	0.040565	0.005358	1
Katnbl1	0.040516	0.001504	1
Slk	0.040461	0.000103	1
Ints2	0.040442	0.012861	1
Ptcd1	0.040409	0.019083	1
Srfbp1	0.040252	0.006116	1
Rexo4	0.040232	0.001781	1
Pwp1	0.040122	0.003014	1
Wdr83os	0.040117	0.004539	1
Plekhn1	0.040093	0.001453	1
Taf15	0.040001	0.015662	1
Ufm1	0.039976	0.000229	1
9430038I0	0.039952	0.000187	1
Sertad3	0.039881	0.000337	1
Gpr89	0.039839	0.000918	1
Dtd1	0.039795	0.035959	1
Isy1	0.039794	0.012631	1
Ago2	0.039784	0.004552	1
Phf6	0.03971	0.004266	1
Acot8	0.039692	0.005759	1
Cyc1	0.039677	0.062509	1
Uqcrfs1	0.039567	0.000883	1
Galk1	0.039494	0.058745	1
Mrps28	0.03947	0.000249	1
Yeats2	0.039463	0.000135	1
Hmgcl	0.039373	0.028126	1
Ppfia1	0.039291	0.250789	1
Nudt3	0.039256	2.05E-05	0.661848
Cisd1	0.039254	0.045546	1
Spata5l1	0.039225	0.000792	1
Fnbp1	0.039102	0.035206	1
Arpc5	0.0391	0.13718	1

Asxl2	0.039052	0.479285	1
Dctn3	0.03898	0.015439	1
Pank2	0.038968	0.001287	1
Haus8	0.038955	0.000131	1
Gart	0.038924	0.002743	1
Cyb561d2	0.038917	0.00011	1
Alkbh6	0.038857	0.043701	1
Fblim1	0.038856	5.12E-06	0.16522
Zmat3	0.038772	0.017891	1
Dcaf13	0.038716	0.033612	1
Itgb1bp1	0.038693	0.240533	1
Arfip2	0.03861	0.006477	1
Rint1	0.038578	0.019938	1
Tmem219	0.038518	0.023449	1
Taf11	0.038491	0.057483	1
Qrsl1	0.038484	0.000995	1
Atp5md	0.038484	0.006112	1
Bag1	0.038413	3.39E-06	0.109412
Rabep1	0.038406	0.001626	1
Gab2	0.038379	0.006768	1
Ppme1	0.03832	0.208001	1
Eci2	0.038315	0.065162	1
Mrps35	0.038269	6.51E-05	1
Aimp2	0.038158	0.000125	1
Cs	0.038157	0.009672	1
Ppp1r13b	0.038145	0.019055	1
Zrsr2	0.03813	0.000236	1
Chd3	0.038073	0.004939	1
Atl3	0.038026	0.007795	1
Sugp1	0.038012	0.000729	1
Dnajc2	0.038006	2.68E-05	0.865062
Ikbkb	0.038002	0.009088	1
Gm16118	0.037977	0.000339	1
Micos13	0.037926	0.001931	1
Rbpms	0.037853	0.000379	1
Snap29	0.037769	0.0012	1
2310061I0	0.037765	0.079436	1
Fam214b	0.037688	0.000358	1
Klhdc4	0.037621	0.046323	1
Exosc8	0.037486	0.032283	1
Fnip1	0.03741	0.059165	1
Ncapd3	0.037388	0.024118	1
Smarca4	0.037347	0.001304	1
Pprc1	0.037311	0.007245	1
Tmem161b	0.037223	0.027401	1
Scfd1	0.037182	0.015015	1
Eif3l	0.037164	0.072188	1
Ankib1	0.037135	0.077253	1

Fcf1	0.036986	0.027169	1
Hinfp	0.036858	0.000136	1
Ube2g1	0.03684	0.331553	1
Gmds	0.036827	0.000955	1
Nsmaf	0.036805	0.003194	1
Trip11	0.036775	0.001153	1
Eipr1	0.036762	0.000131	1
Atp6v1d	0.036623	0.012067	1
Pik3cb	0.036542	0.00385	1
Marc2	0.036505	0.054275	1
Golga5	0.036477	0.000818	1
Eri2	0.036468	0.00231	1
Caprin1	0.036457	0.007103	1
mt-Nd3	0.036401	0.001774	1
Mier2	0.036338	0.001521	1
Ankrd11	0.036296	0.001094	1
Nup205	0.036263	0.000318	1
Pttg1	0.036233	0.003477	1
Mdm4	0.036225	0.003005	1
Prkar2a	0.036204	0.132102	1
Tdrd7	0.036151	2.43E-05	0.78438
Tango2	0.035994	0.002582	1
Esf1	0.035968	0.000591	1
Osbpl2	0.035733	0.123649	1
Eri1	0.035571	0.001788	1
Ints7	0.035505	0.024211	1
Arl5a	0.035482	0.002826	1
G3bp1	0.035437	0.003505	1
Gm9993	0.035366	0.006656	1
A630089N	0.03535	0.004691	1
Med8	0.035334	0.032966	1
Stimate	0.035141	0.001025	1
Arfgap1	0.035117	0.017226	1
March8	0.035084	1.27E-05	0.409258
Pold3	0.035039	0.001839	1
Cbfb	0.034965	0.000983	1
Rwdd4a	0.034954	0.380758	1
Atad5	0.034884	0.000447	1
Trim30b	0.034816	0.001003	1
Ecsit	0.034782	0.014459	1
Smurf2	0.034748	0.048219	1
Ssr2	0.034717	0.031388	1
Rnf123	0.034556	0.099499	1
Phlpp1	0.034518	0.39833	1
Dnmt1	0.034459	0.885098	1
Rnf214	0.034406	0.004658	1
Ndufb2	0.034335	0.017598	1
Slc13a3	0.034185	0.014903	1

Gin1	0.034129	0.114885	1
Tigd2	0.033966	0.001749	1
Ifnar1	0.033926	0.023206	1
Lin54	0.033924	0.021968	1
Golt1b	0.03379	0.000482	1
Gnaq	0.033776	1.6E-05	0.517907
Sec24b	0.03373	0.001359	1
Coq5	0.033689	0.004854	1
Tnip2	0.033649	0.002754	1
Pop7	0.033527	0.000244	1
Rab5c	0.033455	0.109222	1
U2af1	0.033453	0.017308	1
Mrps17	0.033363	0.044469	1
Noc3l	0.033352	0.029253	1
Uqcc1	0.03323	0.003623	1
Arid4a	0.033221	0.129072	1
Guca1a	0.033207	0.003794	1
Ndufb10	0.033201	0.000839	1
Eif4b	0.033153	0.004505	1
6720427I0	0.032994	0.008324	1
Mrps31	0.032851	0.020102	1
Ampd2	0.032831	0.032402	1
Osbpl11	0.032821	0.147643	1
Inpp5f	0.03281	0.000493	1
Erg28	0.03278	0.000923	1
Xbp1	0.032769	0.065116	1
Mrps15	0.032752	0.003532	1
Metap1d	0.032666	0.01492	1
Msn	0.032624	0.211352	1
Timm10	0.032598	0.001123	1
Rcbtb1	0.032432	0.012428	1
Traf2	0.032427	0.035291	1
Heca	0.032339	0.066862	1
Tm9sf1	0.032333	0.007275	1
Ptrh2	0.032299	0.022106	1
Ppdpf	0.032298	0.104076	1
Mfge8	0.032283	0.00073	1
Uchl3	0.032271	0.10028	1
Mroh1	0.032255	0.010202	1
Siva1	0.032203	0.007282	1
Rala	0.032166	0.000118	1
Exosc4	0.032086	0.011684	1
Phip	0.032057	0.077334	1
Lamp1	0.031996	0.14652	1
Prmt7	0.031944	0.041288	1
Ercc8	0.03191	0.003068	1
Gnai2	0.031867	0.316571	1
Fbxw11	0.031748	0.000498	1

Abce1	0.031716	0.002041	1
Cyp20a1	0.03168	0.012997	1
Cebpg	0.03168	0.004778	1
Lars	0.031572	0.007841	1
Nup93	0.031548	0.015193	1
Adss	0.031516	0.354987	1
Senp2	0.03149	0.000494	1
Seh1l	0.031454	0.000332	1
Ndufs4	0.031314	0.012946	1
Mettl1	0.031271	0.004646	1
Slc35e2	0.03112	0.114663	1
Slc30a5	0.031082	0.015655	1
Mrpl41	0.031056	0.000144	1
Gm16599	0.030836	0.00172	1
4930402H	0.030815	0.198591	1
Ythdc1	0.030693	0.010463	1
Umps	0.030676	0.028383	1
Recql5	0.03064	0.011271	1
Mrpl27	0.0306	0.007178	1
Me2	0.030547	0.102236	1
Surf4	0.030545	0.034547	1
Mien1	0.030503	0.005208	1
Psmf1	0.030438	0.006829	1
Etfb	0.030339	0.005484	1
B3galt6	0.030329	0.008051	1
Psmb5	0.030303	0.154642	1
Dlg1	0.030274	0.003039	1
Lman2	0.030222	0.009602	1
1110032A	0.030221	0.021299	1
Arfgap3	0.030209	0.246202	1
Ubr5	0.030044	0.05404	1
Cdk7	0.03003	0.011497	1
Pofut2	0.029991	0.031433	1
Abhd18	0.029967	0.048569	1
Cog5	0.029958	0.021689	1
Pym1	0.029892	0.016439	1
Bnip1	0.029871	0.000122	1
Eps15l1	0.029772	0.050867	1
Rab4b	0.029711	0.019282	1
Golph3	0.029681	0.028144	1
Itga5	0.029567	0.00629	1
Dtd2	0.029524	0.200742	1
Dis3l2	0.029483	0.008462	1
Rpp21	0.029433	0.035414	1
Ubl4a	0.029316	0.170642	1
Ythdc2	0.029307	0.050612	1
Rgs14	0.029307	0.076909	1
Ppp2r2a	0.029305	0.051793	1

Lyar	0.029301	0.010512	1
Tfam	0.029285	0.569087	1
H13	0.029203	0.024282	1
Dpp3	0.029054	0.005415	1
Inip	0.029043	0.000975	1
Opa1	0.028957	0.010602	1
Tbca	0.028944	8.59E-05	1
Ddx41	0.028932	0.015809	1
Slf1	0.0289	0.000813	1
Naa15	0.028894	0.003651	1
Atp6v1f	0.028866	0.019426	1
Etfa	0.028822	0.023276	1
Necap2	0.028757	0.009672	1
Rnpc3	0.028751	0.211142	1
Cpsf4	0.028712	0.030194	1
Gmps	0.02871	0.029801	1
Use1	0.028602	0.010059	1
Usp14	0.028561	0.008114	1
Yif1b	0.028557	0.031082	1
U2af2	0.02853	0.127026	1
Nrbf2	0.028523	0.002143	1
Park7	0.028506	0.07076	1
Lig3	0.028473	0.003773	1
Lonp1	0.028428	7.69E-05	1
Pigs	0.028417	0.005713	1
Trnt1	0.028407	0.08828	1
Cul4b	0.028283	0.02288	1
Dyrk4	0.028166	0.170363	1
B230307C2	0.028161	0.004882	1
Ddt	0.028085	0.001414	1
Rce1	0.028074	0.005708	1
Fis1	0.027956	0.039566	1
Vps29	0.02788	0.008498	1
Nub1	0.027666	0.029082	1
Tmem11	0.027625	0.050019	1
Uap1	0.027624	0.010429	1
Ssbp4	0.027435	0.064561	1
Thyn1	0.027427	0.006306	1
Knop1	0.02724	0.008735	1
Havcr2	0.027239	0.683486	1
Srsf4	0.027225	0.011631	1
Ndufa10	0.027157	0.018992	1
Ap3m2	0.02713	0.090072	1
Sirt3	0.027107	0.000889	1
Zfp131	0.027058	0.004566	1
Hectd3	0.02703	0.011745	1
Clk2	0.027028	0.015253	1
Gba	0.026979	0.004411	1

Rsrc1	0.026969	0.05009	1
Cnot10	0.026863	0.012013	1
Wnk1	0.026824	0.014757	1
Ints13	0.026777	0.017053	1
Tatdn1	0.026763	0.005481	1
Ero1lb	0.02667	0.003089	1
Mgst3	0.026574	0.001453	1
Sms	0.026553	0.041988	1
Gm26510	0.026491	0.008489	1
Ppp1cc	0.026449	0.030226	1
Utp6	0.026158	0.017033	1
Rfxap	0.026071	0.040327	1
Diaph2	0.026034	0.014318	1
Rsph3a	0.026006	0.018442	1
Sugt1	0.025952	0.070985	1
Stk38	0.025938	0.004432	1
Baz2a	0.025927	0.006012	1
2210016L2	0.025886	0.000841	1
Tubgcp4	0.025841	0.05452	1
D230025D	0.025827	0.214003	1
Mbd3	0.025809	0.000648	1
Ppp1r3d	0.025807	0.000915	1
Ing5	0.025789	0.009132	1
Nras	0.025703	0.03005	1
Bms1	0.025699	0.037764	1
Rnf166	0.025696	0.002857	1
Fubp1	0.025591	0.036821	1
Sgf29	0.025558	0.023683	1
Ndufv2	0.025476	0.016393	1
Gm16337	0.025428	0.162336	1
Acaa1a	0.025376	0.112556	1
Mrpl19	0.025363	0.034925	1
Tep1	0.025348	0.001908	1
Plekhm2	0.025339	8.73E-05	1
Pctp	0.025333	0.004814	1
Tceanc2	0.025269	0.013731	1
Smn1	0.025236	6.05E-06	0.195352
Slc25a12	0.025181	0.019025	1
Nkapd1	0.025149	0.000949	1
Xrcc6	0.025106	0.253248	1
Psd4	0.02507	0.10593	1
Crlf2	0.025065	0.011411	1
Zfp346	0.024992	0.021231	1
Crk	0.024943	0.00449	1
Nhlrc2	0.024864	0.031689	1
Slc39a6	0.024839	0.003827	1
Mpp1	0.024828	0.110282	1
Ndufab1	0.024811	0.035234	1

Dnajc5	0.024798	0.010022	1
Tsen2	0.024753	0.080671	1
Pdcd4	0.024733	0.835457	1
Stmn1	0.024686	0.005704	1
Rbl1	0.02468	0.070045	1
Zdhhc4	0.024551	0.00444	1
Qtrt1	0.024445	0.022988	1
Tsc1	0.024414	0.138175	1
3830406C1	0.024318	0.014803	1
Gmppa	0.024228	0.079297	1
Smg7	0.024165	0.0211	1
Eef2kmt	0.024051	0.00071	1
9130401M	0.024018	0.011248	1
Mrpl46	0.02399	0.005241	1
Fiz1	0.023984	0.070026	1
Meaf6	0.023966	0.019581	1
Ube2k	0.023897	0.00145	1
Ric1	0.023886	0.000863	1
Ilkap	0.023875	0.01841	1
Toe1	0.023866	0.244694	1
Polr3h	0.023739	0.053539	1
Denr	0.023637	0.000397	1
Dph6	0.023635	0.0186	1
Plekhb2	0.023552	0.002452	1
Sars2	0.023516	0.002599	1
Eif3g	0.023499	0.000564	1
4921524J1	0.023434	0.010603	1
Hnrnpm	0.023422	0.069816	1
Ccnh	0.023419	0.047688	1
Safb	0.023382	0.012067	1
Ip6k1	0.023351	0.000843	1
Camk2d	0.023312	0.065657	1
Snupn	0.023281	0.002371	1
AI413582	0.023209	0.043349	1
Mzt2	0.02317	0.04804	1
Hdac4	0.023117	0.045984	1
Adipor1	0.022952	0.585147	1
Miga1	0.022847	0.05387	1
AU022793	0.022805	0.029333	1
Simc1	0.022792	0.038169	1
Set	0.022617	0.000573	1
Flad1	0.022611	0.011977	1
Fam98c	0.022602	0.055066	1
Trpm7	0.022579	0.025647	1
Kitl	0.022423	0.011407	1
Mrpl17	0.022402	0.024949	1
Prkrip1	0.022381	0.010501	1
Fam114a1	0.022272	0.000952	1

Cars2	0.022239	0.001093	1
Nup54	0.022125	0.033126	1
N4bp2l2	0.022088	0.184107	1
Rtca	0.022057	0.01612	1
Sumf1	0.022057	0.00485	1
Tmem41b	0.021996	0.1157	1
Cdc40	0.021981	0.118487	1
Tbce	0.021954	0.042471	1
Tmem161a	0.021946	0.023899	1
Cd2ap	0.021823	0.037413	1
Cdk17	0.021711	0.251226	1
Rap1b	0.021569	0.011816	1
Hmgcr	0.021478	0.003159	1
Polr3d	0.021379	0.000637	1
Gpatch3	0.021271	0.018663	1
Ralgapa2	0.021198	0.42819	1
Gemin7	0.021145	0.046008	1
Ddx23	0.02114	0.060068	1
Ak6	0.021132	0.05271	1
Cog3	0.021041	0.076269	1
Ccm2	0.020998	0.040212	1
Atp5a1	0.02099	0.340931	1
Pex13	0.020978	0.285558	1
Bdp1	0.020945	0.000605	1
Pin4	0.020941	0.04784	1
Sart1	0.020936	0.038091	1
Nectin2	0.020822	0.000332	1
Mbtd1	0.020819	0.016353	1
Mrps26	0.020812	0.042107	1
Rrbp1	0.020811	0.022277	1
Ppm1k	0.020776	0.137078	1
Ubxn4	0.020776	0.027292	1
Mrtfb	0.020732	0.082108	1
Tmf1	0.020708	0.235757	1
Ccdc186	0.0207	0.013963	1
Pds5b	0.020627	0.689942	1
Gm17066	0.020607	0.008177	1
Kif13b	0.020599	0.031094	1
Prep	0.020567	0.087158	1
Pcyox1	0.020554	0.030806	1
Nom1	0.020538	0.008293	1
Jund	0.020517	0.356759	1
Fbxw7	0.02051	0.002217	1
Sun1	0.02051	0.100039	1
Tctex1d2	0.020482	0.019985	1
Rpp30	0.020411	0.13951	1
Zfp654	0.020371	0.073608	1
H2afx	0.020363	0.000806	1

Atf4	0.020353	0.012622	1
Ciapin1	0.020344	0.047033	1
Scyl3	0.020296	0.032453	1
Prim2	0.020284	0.87842	1
Csnk1g3	0.020225	0.040093	1
Hspbap1	0.020149	0.016804	1
Nsun4	0.02009	0.003887	1
Tars	0.019932	0.022015	1
Ttll4	0.019913	0.000822	1
Selenof	0.01985	0.307657	1
Brd2	0.019787	0.401472	1
March1	0.019757	0.433764	1
Notch2	0.019752	0.079385	1
Naip6	0.019746	0.001113	1
Ddx56	0.01973	0.031646	1
Mtap	0.019696	0.042662	1
Eif4enif1	0.019678	0.00399	1
Ndufa4	0.019652	0.058918	1
Fam160a2	0.019639	0.486392	1
Coq9	0.019496	0.017502	1
Supv3l1	0.019378	0.406302	1
Gcsh	0.019158	0.018723	1
Gm27017	0.019144	0.024921	1
Rpa1	0.019143	0.1251	1
Fastkd2	0.019075	0.001966	1
Dbi	0.019056	0.00889	1
Cox10	0.018925	0.019748	1
Cox18	0.018903	0.002819	1
Znhit1	0.018834	0.004524	1
Tipin	0.01881	0.073372	1
Eif2b2	0.018776	0.144964	1
Btbd7	0.018774	0.01138	1
Wdr70	0.018754	0.003423	1
Csnk2a2	0.018669	0.011142	1
Sumo3	0.018655	0.117905	1
Epn1	0.01857	0.004036	1
Chst14	0.018521	0.261594	1
Dars	0.018484	0.170168	1
Telo2	0.018476	0.000109	1
Ubb	0.018456	0.945108	1
Zfp53	0.018451	0.086577	1
Dpy19l4	0.01843	0.051289	1
Sec61a2	0.018391	0.023384	1
Wdr83	0.018386	0.001309	1
Ftx	0.018295	0.281391	1
Psmd13	0.018202	0.119959	1
Rbm39	0.018156	0.836377	1
Polg	0.018107	0.055998	1

Tex14	0.018044	0.228082	1
Tysnd1	0.018022	0.25748	1
Xpa	0.018002	0.112268	1
Cnot4	0.017991	0.015071	1
Wdr73	0.017954	0.023141	1
Eprs	0.017866	0.039995	1
Tradd	0.017837	0.059254	1
Ints11	0.01782	0.25218	1
Tsfm	0.017682	0.001325	1
Rusc1	0.017628	0.001247	1
Pip5k1c	0.017627	0.04338	1
Psme3	0.017623	0.019315	1
Lsm1	0.017606	0.006986	1
Gm48678	0.017508	0.146949	1
Atxn7	0.017485	0.195348	1
Stk40	0.017445	0.043751	1
Spryd3	0.017381	0.003414	1
Ergic2	0.017306	0.035641	1
Nudt7	0.017263	0.035767	1
Msmo1	0.017247	0.28081	1
Cul2	0.01724	0.173732	1
Trir	0.017195	0.159308	1
Dgcr8	0.017144	0.098411	1
Prr3	0.017059	0.000282	1
Atp5j	0.017046	0.009915	1
Qpctl	0.016911	0.022068	1
Mrpl10	0.016889	0.173557	1
Hmgn2	0.016761	0.192845	1
Cpeb3	0.016744	0.541764	1
Emc8	0.016683	0.011887	1
Ranbp9	0.016655	0.180148	1
Gna13	0.016596	0.088991	1
Exosc9	0.016585	0.012112	1
Slbp	0.016582	0.276495	1
Ust	0.016309	0.993677	1
Dedd	0.016222	0.163284	1
Nudcd2	0.016204	0.004923	1
Htra3	0.016152	0.001728	1
Med26	0.016107	0.017705	1
Hmg20b	0.016078	0.071829	1
Katna1	0.016072	0.002761	1
Npm3	0.016072	0.009082	1
Epb41l3	0.016067	0.029856	1
Napg	0.016024	0.032074	1
Rragc	0.016019	0.425308	1
Tbcel	0.015953	0.308805	1
Rab12	0.015924	0.011451	1
Tnfaip8	0.015904	0.009393	1

Utp15	0.015899	0.04705	1
Zfp958	0.015855	0.023253	1
Mrps34	0.015845	0.331486	1
Vac14	0.015798	0.000677	1
Asf1a	0.015704	0.073624	1
Glrx3	0.015631	0.064004	1
Agtrap	0.015627	0.004601	1
Prxl2c	0.015543	0.03527	1
Atp6v1h	0.015516	0.342466	1
Tomm22	0.015496	0.075605	1
Smarce1	0.015459	0.041607	1
Borcs5	0.01536	0.105207	1
Azin1	0.015314	0.005211	1
Sugp2	0.015291	0.022923	1
Slc27a1	0.015264	0.000538	1
Pnn	0.015243	0.048293	1
Bad	0.015166	0.019441	1
Fem1b	0.015059	0.21499	1
Mon1a	0.015005	0.149938	1
Mad1l1	0.014922	0.208188	1
Mlh3	0.014863	0.031336	1
Isca2	0.014845	0.039466	1
Rps27rt	0.014757	0.007547	1
Cryzl2	0.014743	0.010579	1
Rtf1	0.014662	0.021815	1
Acly	0.014625	0.163342	1
Zfp516	0.014618	7.16E-06	0.231287
Polr3k	0.014601	0.163009	1
Map3k7	0.014587	0.002181	1
Copg2	0.014551	0.153176	1
Skiv2l	0.014503	0.052368	1
Polr1c	0.014485	0.039064	1
Fastkd1	0.01443	0.00696	1
Nkiras2	0.014421	0.002718	1
Timmdc1	0.01435	0.016889	1
Zdhhc16	0.014277	0.073961	1
Sfxn2	0.014222	0.054445	1
Mpst	0.014211	0.007045	1
Prkag2	0.014189	0.070194	1
Ndufa3	0.014183	0.000458	1
Ggh	0.014172	0.096341	1
Usf3	0.014067	0.013008	1
Ddx54	0.014064	0.008014	1
Hddc2	0.013984	0.038828	1
Snhg3	0.01392	0.002442	1
Ppp1r12a	0.013814	0.888964	1
Tmem192	0.013809	0.010853	1
Trappc2l	0.013753	0.026068	1

Tmem131	0.013652	0.139312	1
Fxr1	0.013611	0.014603	1
Rhbdd1	0.013604	0.296417	1
Fbxo7	0.01352	0.00802	1
Sos1	0.013497	0.079797	1
Glud1	0.013454	0.077277	1
Zfp609	0.013407	0.018154	1
Hrh2	0.01339	0.001005	1
Pdrg1	0.01338	0.024671	1
Plgrkt	0.013361	0.457207	1
Rnf24	0.013259	0.046218	1
Rad9a	0.013218	0.102748	1
Il1rl2	0.013159	0.005645	1
Acp1	0.013148	0.004462	1
Tsen15	0.013123	0.055337	1
1810026B(	0.013062	0.052221	1
Mrpl28	0.013054	0.008668	1
Pold2	0.012987	0.005381	1
Syncrip	0.012945	0.137822	1
Slc39a11	0.012929	0.019987	1
Mrpl51	0.012865	0.009374	1
Spred2	0.012807	0.145153	1
Taf7	0.012759	0.610831	1
Ufc1	0.012729	0.008606	1
1700021F(	0.012652	0.015964	1
Smc5	0.012601	0.184225	1
Mfsd8	0.012591	0.036538	1
Fbxo33	0.012545	0.111299	1
Kif1b	0.012499	0.043904	1
Klhdc10	0.012459	0.070067	1
Cd101	0.012456	0.189828	1
Dimt1	0.012425	0.002488	1
Ddx49	0.012414	0.001895	1
Ergic1	0.012384	0.026048	1
Ric3	0.012317	0.089631	1
Med11	0.012216	0.000439	1
Mir142hg	0.0122	0.278862	1
Nbdy	0.01213	0.09994	1
Atpaf2	0.012129	0.10313	1
Nup155	0.012098	0.002835	1
Traf5	0.012095	0.143363	1
Zfp40	0.011923	0.302257	1
Mob2	0.011917	0.152212	1
Mief1	0.011909	0.158496	1
Eif2a	0.011778	0.004443	1
Dnajc4	0.011776	0.691638	1
Parvb	0.011634	0.005953	1
Rpusd4	0.011588	0.006939	1

Flnb	0.011522	0.403083	1
BC004004	0.011516	0.182435	1
Phtf1	0.011449	0.000326	1
Metap1	0.011408	0.012093	1
Rrm1	0.01139	0.309066	1
Rev1	0.01138	0.12381	1
Cuedc2	0.011349	0.067344	1
Flvcr1	0.011325	0.014238	1
Crlf3	0.01131	0.367245	1
Wdr91	0.011243	0.001732	1
Rnaseh1	0.011237	0.01752	1
Nono	0.011228	0.350158	1
Tusc2	0.011185	0.053294	1
Wdr5	0.011152	0.166876	1
L3mbtl3	0.01115	0.018498	1
Atr	0.011146	0.000735	1
Gng12	0.01114	0.022353	1
Mrps27	0.011082	0.018389	1
Tma16	0.011028	0.001687	1
Ubl5	0.011009	0.008537	1
Synrg	0.011004	0.023449	1
Ankrd39	0.010982	0.011254	1
Zfp281	0.01094	0.032692	1
Zswim8	0.010816	0.300602	1
Hs2st1	0.010791	0.012082	1
Cabin1	0.010782	0.018609	1
Ilf2	0.010733	0.038478	1
Eif4e	0.010731	0.041422	1
Ppp2r5e	0.010706	0.01583	1
Arl6ip6	0.01056	0.219732	1
Washc1	0.010457	0.111031	1
Ubac2	0.010456	0.980841	1
Sdc4	0.010428	0.016958	1
Rpain	0.010384	0.015317	1
Tut1	0.010383	0.075226	1
Zfp472	0.010382	0.097375	1
Ehbp1l1	0.010325	0.000307	1
Txndc16	0.010263	0.000269	1
Akap8	0.010202	0.091153	1
Traf6	0.010171	0.002032	1
Sec24a	0.010147	0.039848	1
Hsd3b7	0.010129	0.25942	1
Setd5	0.01012	0.30045	1
Fdx1	0.010099	0.002806	1
Kat2a	0.010058	0.030785	1
Ptar1	0.010041	0.006203	1
Chd2	0.010029	0.092083	1
Rab7	-0.01003	0.127083	1

Rnf8	-0.01005	0.057545	1
Nprl3	-0.01006	0.098269	1
Nab2	-0.01017	0.038815	1
Mag	-0.01022	0.064738	1
Tefm	-0.01025	0.039575	1
Gpkow	-0.01029	0.185185	1
Actl6a	-0.01031	0.191202	1
Hif1an	-0.01041	0.852002	1
Rnf31	-0.01043	0.005781	1
Gnptg	-0.01043	0.163103	1
Ufd1	-0.01044	0.185259	1
Ing3	-0.01044	0.972826	1
Mospd3	-0.01046	0.140457	1
Zfp429	-0.01047	0.099748	1
Zfp91	-0.01053	0.006925	1
Mrpl37	-0.01055	0.119139	1
Rufy1	-0.01067	0.262268	1
Zfp326	-0.0107	0.339841	1
Rnf215	-0.01073	0.14739	1
Socs4	-0.01074	0.037607	1
Usp47	-0.01075	0.331748	1
Rars2	-0.01077	0.660113	1
Nelfe	-0.0108	0.515136	1
Pgrmc2	-0.01088	0.826686	1
Tarbp2	-0.01089	0.754355	1
Vrk2	-0.0109	0.290366	1
Fam207a	-0.01094	0.036631	1
Ubr7	-0.01096	0.471425	1
Stk11ip	-0.01098	0.305917	1
Uxs1	-0.01107	0.043423	1
Supt6	-0.01114	0.311012	1
Ankrd52	-0.01115	0.544138	1
Mrpl3	-0.01117	0.066259	1
Zcchc9	-0.0112	0.065749	1
Las1l	-0.01131	0.574518	1
Ppp2r3c	-0.01134	0.022829	1
Mysm1	-0.01137	0.983832	1
Ndufaf7	-0.01142	0.582193	1
Hif1a	-0.01145	0.524419	1
Zc3h18	-0.01145	0.149573	1
Slc12a4	-0.0115	0.014821	1
Ccdc34	-0.0115	0.146689	1
Tmem209	-0.01152	0.012288	1
Rad51d	-0.01152	0.280457	1
Bccip	-0.01153	0.439038	1
Mrps18a	-0.01154	0.164359	1
Tmem68	-0.01155	0.096603	1
Nbn	-0.01161	0.361604	1

Gphn	-0.01164	0.05709	1
Oaz1	-0.01168	0.89465	1
Armc5	-0.01168	0.341693	1
Tmem9b	-0.01168	0.333622	1
Ntmt1	-0.01174	0.034087	1
Abcc4	-0.01177	0.109883	1
Faim	-0.01179	0.124464	1
Ddx42	-0.01187	0.045747	1
Galnt7	-0.01197	0.030515	1
Exog	-0.01203	0.205413	1
Ogfod2	-0.01206	0.505561	1
Asb6	-0.0121	0.814009	1
Zfp410	-0.01211	0.330903	1
Zfp141	-0.0122	0.407546	1
Spns2	-0.01221	0.001711	1
Nomo1	-0.01222	0.352898	1
Map3k2	-0.01225	0.994177	1
Dda1	-0.01232	0.110257	1
Gle1	-0.01235	0.038727	1
Washc3	-0.01237	0.124463	1
Rpap1	-0.0124	0.550782	1
Smap1	-0.01241	0.203678	1
Wdyhv1	-0.01243	0.3626	1
Epg5	-0.01244	0.069016	1
Nup50	-0.01247	0.047342	1
Galnt12	-0.0125	0.111608	1
Rabggta	-0.01261	0.017585	1
Ddx55	-0.01264	0.383439	1
Galnt11	-0.01267	0.06163	1
Trmu	-0.01275	0.974772	1
Ripor1	-0.01277	0.185424	1
Fastk	-0.01278	0.641259	1
Cbfa2t2	-0.01284	0.156747	1
Tpd52l2	-0.01287	0.303997	1
Snx15	-0.01287	0.204836	1
BC017158	-0.01292	0.41178	1
Tada2a	-0.01297	0.509531	1
Bckdhb	-0.01298	0.023299	1
Poglut2	-0.01299	0.596915	1
Kif2a	-0.013	0.417941	1
Foxred1	-0.01301	0.197236	1
Terf2	-0.01306	0.344346	1
Luc7l	-0.01312	0.181527	1
Rnf220	-0.01317	0.885094	1
Sdhaf1	-0.01317	0.00734	1
Xpnpep3	-0.01319	0.235518	1
Utp20	-0.01319	0.024267	1
P4hb	-0.01319	0.43053	1

Xpnpep1	-0.0132	0.109891	1
Trub1	-0.01322	0.113982	1
Sec22a	-0.01325	0.451921	1
Ccnk	-0.01326	0.71732	1
Cox7c	-0.01332	0.304007	1
Laptm4a	-0.01347	0.814665	1
Gstt2	-0.0135	0.178918	1
Pcyt1a	-0.01356	0.431045	1
Ftsj1	-0.01361	0.661167	1
Fnbp1l	-0.01363	0.022798	1
Parp2	-0.01371	0.141671	1
Cdk5rap2	-0.01385	0.417163	1
Scaf8	-0.01385	0.249729	1
Fbxo38	-0.01388	0.111264	1
Dhrs1	-0.01401	0.198836	1
5730480H(	-0.01404	0.323846	1
Rab10	-0.01405	0.929051	1
Flot1	-0.01405	0.402162	1
Ldlrap1	-0.01406	0.182214	1
Gm2245	-0.01412	0.364085	1
Prkd2	-0.01414	0.124393	1
Trmt1	-0.01414	0.127187	1
Cds1	-0.01417	0.275299	1
Ppil2	-0.01418	0.538364	1
Rbm7	-0.01418	0.46752	1
Ubxn1	-0.01425	0.036313	1
Xpr1	-0.01432	0.870625	1
Thoc5	-0.01433	0.073389	1
Otud6b	-0.01435	0.512948	1
Fzr1	-0.01439	0.797438	1
Fuca2	-0.01441	0.094126	1
Naa40	-0.01445	0.143754	1
Rpf1	-0.01446	0.151464	1
Psmd6	-0.01449	0.184514	1
Tvp23b	-0.01455	0.588985	1
Esr1	-0.0146	0.124544	1
Ift88	-0.0147	0.171654	1
Polr3a	-0.01477	0.754181	1
Zfp160	-0.01478	0.382598	1
Sephs2	-0.01481	0.648727	1
Zgpat	-0.01481	0.713771	1
Exosc5	-0.01484	0.531023	1
Nup160	-0.01488	0.544062	1
Fam53b	-0.01489	0.162863	1
Cluap1	-0.01491	0.826089	1
Zkscan14	-0.01496	0.965508	1
Pfdn1	-0.01497	0.092349	1
Atg4d	-0.01501	0.307412	1

Ctif	-0.01501	0.511112	1
Braf	-0.01501	0.101896	1
Ankrd27	-0.01508	0.777055	1
Tmem41a	-0.01508	0.460613	1
Sh3bp5	-0.0151	0.674198	1
Dop1a	-0.01512	0.235964	1
Psmd2	-0.01519	0.702263	1
Osbp	-0.01521	0.293759	1
Aggf1	-0.01527	0.292201	1
Gdpd3	-0.01539	0.24321	1
Hectd1	-0.01543	0.362914	1
Trrap	-0.01545	0.070406	1
Rsph3b	-0.0156	0.044353	1
Paxbp1	-0.0156	0.039265	1
Ndufs8	-0.01571	0.304408	1
Tbpl1	-0.01572	0.450917	1
Sumo2	-0.01582	0.611181	1
Usp12	-0.01583	0.000849	1
Mdp1	-0.01584	0.223069	1
Dcaf10	-0.01587	0.150605	1
Dpy19l1	-0.01596	0.090248	1
Ccdc32	-0.01598	0.670996	1
Cep350	-0.01607	0.164295	1
Wdr77	-0.01609	0.599276	1
Zfp36l1	-0.0161	0.011569	1
Vkorc1l1	-0.0161	0.068452	1
Ldah	-0.01614	0.808437	1
Dlat	-0.01618	0.201	1
6030458C1	-0.01622	0.577748	1
Ino80c	-0.01627	0.465886	1
Zfp292	-0.01628	0.306556	1
Gtpbp1	-0.01636	0.163205	1
Sod1	-0.01637	0.045472	1
Crbn	-0.0164	0.75381	1
Nagk	-0.01643	0.366815	1
Cers5	-0.01649	0.416458	1
Lmbrd1	-0.01654	0.319917	1
Erlin1	-0.01657	0.945885	1
Itsn2	-0.01659	0.273618	1
Dctn4	-0.01661	0.545596	1
Fbxw8	-0.01664	0.313528	1
Mpg	-0.0167	0.428332	1
Gosr1	-0.01674	0.560355	1
Gabarap	-0.01684	0.43591	1
Tubg1	-0.01687	0.757942	1
Esco1	-0.01694	0.179952	1
Rab6a	-0.01698	0.232833	1
Ccdc115	-0.01698	0.387442	1

Cct3	-0.01698	0.062852	1
Ess2	-0.01713	0.448806	1
Rps19bp1	-0.01714	0.620114	1
Zfp809	-0.01714	0.08386	1
Rnf138	-0.01719	0.846606	1
Fh1	-0.01721	0.255593	1
mt-Nd6	-0.01723	0.259989	1
Glipr1	-0.01725	0.396892	1
Ermard	-0.01728	0.580987	1
Tjap1	-0.01729	0.179	1
Casp7	-0.01731	0.009687	1
Polr2i	-0.01732	0.102984	1
Slc23a2	-0.01734	0.305402	1
Lsm6	-0.01741	0.075026	1
Uspl1	-0.01746	0.758477	1
Acbd5	-0.01748	0.121926	1
Selenoi	-0.0175	0.89141	1
Ppm1a	-0.01751	0.191775	1
Lbh	-0.01754	0.59429	1
Anxa7	-0.01755	0.22305	1
Tspyl2	-0.01766	0.368945	1
Kdm2a	-0.01767	0.697399	1
Btg1	-0.0177	0.701953	1
Sf3b5	-0.0177	0.281833	1
Thap4	-0.01782	0.292559	1
Rbm19	-0.01782	0.204351	1
Pfdn4	-0.01788	0.262309	1
Iws1	-0.0179	0.121755	1
Scamp1	-0.01791	0.397211	1
Tmem234	-0.01791	0.155717	1
Cds2	-0.01791	0.246677	1
Faap20	-0.01793	0.17752	1
Cops8	-0.018	0.768007	1
Erp29	-0.01808	0.89137	1
Siae	-0.01811	0.231911	1
Clpp	-0.01826	0.185509	1
Ammecr1l	-0.01829	0.143956	1
Rtf2	-0.01831	0.846283	1
Fam98b	-0.01833	0.763044	1
Chic2	-0.01834	0.717974	1
Isoc2a	-0.01834	0.622393	1
Hdac8	-0.01837	0.098919	1
Ndufb4	-0.01837	0.284704	1
Ccdc88a	-0.01839	0.00382	1
Nmt2	-0.01844	0.082114	1
Pdhx	-0.01844	0.019692	1
A430005L1	-0.01848	0.163463	1
Fdft1	-0.01851	0.400246	1

Kpna2	-0.01858	0.789387	1
Traf7	-0.01859	0.170243	1
Khk	-0.01861	0.476068	1
Uvssa	-0.01869	0.135526	1
Gnb1	-0.01869	0.050927	1
Tmcc1	-0.01875	0.032216	1
Uba3	-0.01881	0.638656	1
Zwint	-0.01881	0.321231	1
Arntl	-0.01883	0.633854	1
Smarca5	-0.01887	0.009615	1
Tmem208	-0.01888	0.371491	1
Pcgf3	-0.01894	0.291667	1
Eml3	-0.01896	0.975324	1
Aspscr1	-0.01896	0.164809	1
Mrps30	-0.01903	0.154212	1
Pole4	-0.01907	0.177743	1
Dnttip2	-0.0191	0.731475	1
Txndc11	-0.01914	0.529253	1
Timm8b	-0.0192	0.266974	1
Fam20b	-0.01924	0.11475	1
Smg1	-0.0193	0.061804	1
Dmap1	-0.01932	0.203825	1
Pmpca	-0.01933	0.701015	1
Shc1	-0.01933	0.147761	1
C2cd2l	-0.01934	0.599056	1
Rhbdd3	-0.01934	0.333026	1
Sc5d	-0.01942	0.280946	1
Maip1	-0.01943	0.220191	1
Fam49b	-0.01945	0.310199	1
Nudt4	-0.01953	0.106468	1
Asnsd1	-0.01954	0.32224	1
Mapk7	-0.01955	0.942765	1
Gskip	-0.01956	0.931353	1
Slc8b1	-0.01958	0.133108	1
Ksr1	-0.01958	0.549829	1
Fbxo11	-0.01959	0.022898	1
Zfp780b	-0.01959	0.455342	1
Alg11	-0.01963	0.467031	1
Rsrc2	-0.01964	0.378393	1
Agfg1	-0.01965	0.870938	1
Asl	-0.01965	0.137822	1
Pdcd7	-0.01967	0.403761	1
Ccl25	-0.0197	0.087535	1
Zbtb8os	-0.01974	0.263555	1
Eif2s2	-0.01975	0.48389	1
Mrps7	-0.01977	0.687317	1
Nipsnap2	-0.0198	0.125386	1
Rmnd5b	-0.01982	0.707076	1

Dxo	-0.01985	0.880462	1
Ptms	-0.01986	0.431747	1
Tmem184c	-0.01988	0.138403	1
Brms1l	-0.01995	0.479588	1
Atxn7l1	-0.01997	0.586119	1
Acsf2	-0.01999	0.324694	1
Rnf20	-0.02001	0.569334	1
Naa16	-0.02004	0.135364	1
Hnrnpul2	-0.02006	0.709154	1
Cul4a	-0.02012	0.233039	1
Opa3	-0.02012	0.591257	1
Ppt1	-0.02013	0.497272	1
E330020D:	-0.02017	0.24179	1
Abhd10	-0.02018	0.569917	1
U2surp	-0.02031	0.115236	1
Cenpo	-0.02034	0.131173	1
Sp3os	-0.02035	0.261814	1
Rcor1	-0.02038	0.029545	1
Atg2b	-0.02045	0.545467	1
Mettl2	-0.0205	0.165918	1
Hgs	-0.0205	0.5038	1
Rfx5	-0.02052	0.087955	1
Rrad	-0.02058	0.783249	1
Zfp335	-0.0207	0.608668	1
Samd4b	-0.02075	0.457595	1
Mrpl49	-0.02075	0.981493	1
Bnip3	-0.02075	0.826533	1
Fam126a	-0.02076	0.75662	1
Ranbp3	-0.02077	0.605561	1
Stim1	-0.0208	0.324538	1
Rrp8	-0.02085	0.658197	1
Slc35a2	-0.02094	0.232209	1
Als2	-0.02096	0.338447	1
Fam133b	-0.02099	0.118177	1
Med29	-0.02106	0.174945	1
Usp5	-0.02108	0.294113	1
Mrpl24	-0.02108	0.224328	1
Cnot1	-0.02114	0.256277	1
Trp53inp1	-0.02114	0.391233	1
Nans	-0.02117	0.123305	1
Stk38l	-0.02118	0.156403	1
Pebp1	-0.02127	0.146888	1
Psmg1	-0.02133	0.115451	1
Dbt	-0.02137	0.736798	1
Lsm3	-0.02138	0.338059	1
Aida	-0.02138	0.202141	1
Rab28	-0.02141	0.432983	1
Myl12b	-0.02143	0.694284	1

Thumpd1	-0.02144	0.779295	1
Cab39l	-0.02152	0.401022	1
Ankrd13a	-0.02154	0.199676	1
Trim26	-0.02163	0.9601	1
Hipk3	-0.02163	0.678889	1
Mtx1	-0.02164	0.319529	1
Pan2	-0.02167	0.460321	1
Drap1	-0.02168	0.631179	1
Zscan29	-0.02174	0.493746	1
Usp6nl	-0.0218	0.350772	1
Chpf2	-0.02181	0.89228	1
Pnkp	-0.02183	0.154806	1
Snf8	-0.02186	0.710293	1
Lias	-0.02188	0.311425	1
Nsun3	-0.02193	0.110432	1
Usp42	-0.02208	0.448122	1
Znhit2	-0.02209	0.510386	1
Mcu	-0.0221	0.237013	1
Iffo2	-0.02211	0.41795	1
Tmem179b	-0.02212	0.160112	1
Stt3b	-0.02215	0.141713	1
S100a13	-0.02216	0.041118	1
Zfp574	-0.0222	0.502261	1
Aldh3b1	-0.02224	0.515266	1
S100pbp	-0.02226	0.998776	1
Natd1	-0.02226	0.766119	1
Hist1h1c	-0.02228	0.404868	1
Gtf2ird2	-0.02228	0.815937	1
Ttc1	-0.02232	0.457677	1
Pik3cd	-0.02234	0.264796	1
Snrpc	-0.02246	0.17799	1
Lsm2	-0.0225	0.23506	1
Ap5b1	-0.0225	0.533766	1
Gm7072	-0.02252	0.859999	1
Zfp282	-0.02253	0.927065	1
Rrp1	-0.02266	0.126403	1
Vps16	-0.02266	0.344873	1
Ahsa1	-0.02272	0.402465	1
2700097O0	-0.02275	0.301424	1
Mkrn2	-0.0228	0.182713	1
Clasrp	-0.02282	0.478516	1
Edf1	-0.02285	0.10345	1
Eef1akmt1	-0.02287	0.514044	1
Ctdp1	-0.02292	0.335358	1
Rfwd3	-0.02297	0.349379	1
Map4	-0.02299	0.240331	1
Rnf41	-0.02305	0.222852	1
Ttc37	-0.02313	0.027768	1

Fancc	-0.02313	0.170872	1
Nfyc	-0.02314	0.845625	1
Fbh1	-0.02321	0.08043	1
Ino80b	-0.02325	0.648803	1
Btbd1	-0.02348	0.224617	1
Pkd1	-0.0235	0.696003	1
Lrp6	-0.0235	0.876285	1
Bclaf3	-0.02359	0.880008	1
Cdc14a	-0.02361	0.16867	1
Mtpap	-0.02363	0.417452	1
Snrpb2	-0.02363	0.237912	1
Mib1	-0.02364	0.210107	1
Sv2a	-0.02368	0.263972	1
Usp15	-0.02374	0.234111	1
Auh	-0.02374	0.940679	1
Setx	-0.02375	0.590305	1
Pcnt	-0.02377	0.726298	1
Riok1	-0.02391	0.082419	1
Gucd1	-0.02392	0.816531	1
Fuca1	-0.02397	0.738195	1
Spopl	-0.024	0.183548	1
Irf3	-0.02403	0.08724	1
Med12	-0.02403	0.227853	1
Akap11	-0.02405	0.239109	1
Mrgbp	-0.02406	0.14225	1
Rel	-0.02406	0.936261	1
Slc39a3	-0.02414	0.583532	1
Srcap	-0.02417	0.924427	1
Ints1	-0.02431	0.284022	1
1110038F1	-0.02435	0.378462	1
Akap10	-0.02436	0.28958	1
Strn3	-0.02442	0.122357	1
Chchd5	-0.02444	0.345427	1
Nacc1	-0.02446	0.707533	1
Camta1	-0.02447	0.042338	1
Kpna1	-0.02449	0.927758	1
Plcg2	-0.02453	0.618354	1
Kin	-0.02455	0.401457	1
Parg	-0.02457	0.366562	1
Enc1	-0.02458	0.775626	1
Ubxn6	-0.02461	0.766952	1
Rffl	-0.02468	0.178832	1
Ddx47	-0.02472	0.530658	1
Rps6kb1	-0.02478	0.124197	1
Prkaa1	-0.0248	0.305601	1
Hsbp1	-0.02483	0.54065	1
Fance	-0.02484	0.463962	1
Cwc25	-0.02486	0.155899	1

Psmc5	-0.0249	0.616025	1
Tlnrd1	-0.02491	0.663126	1
Mllt10	-0.02491	0.979087	1
Ttc33	-0.02491	0.920001	1
Sipa1l3	-0.02494	0.066618	1
Snx20	-0.02495	0.23261	1
Hint2	-0.02499	0.07711	1
Phka2	-0.02509	0.116142	1
Taf1	-0.02511	0.252004	1
Rttn	-0.02513	0.321439	1
Ppp5c	-0.02513	0.429045	1
Snx24	-0.02514	0.528669	1
Zfp407	-0.02519	0.035506	1
Tmem222	-0.0252	0.41394	1
Lsm7	-0.02526	0.055525	1
Rex1bd	-0.0253	0.125836	1
Slc35a1	-0.02533	0.861007	1
Dcaf6	-0.02536	0.951241	1
Zfp800	-0.02539	0.831342	1
Zfp983	-0.02546	0.937644	1
Taf2	-0.0255	0.54728	1
Araf	-0.02551	0.95309	1
Sde2	-0.02551	0.476881	1
Psmd4	-0.02553	0.197156	1
Fam234a	-0.02558	0.351069	1
Ppp1r37	-0.02561	0.141331	1
Ube2n	-0.02566	0.201708	1
Ncbp2	-0.02566	0.491451	1
Rexo2	-0.0257	0.724136	1
Anks1	-0.02572	0.076753	1
Elp5	-0.02579	0.348857	1
Spty2d1	-0.02583	0.941756	1
Mettl9	-0.02588	0.265174	1
Crtc2	-0.0259	0.910308	1
Gbe1	-0.02591	0.792724	1
Rdm1	-0.02593	0.257936	1
Atp13a2	-0.02599	0.027466	1
Taf6	-0.02599	0.622729	1
Bag4	-0.02602	0.998995	1
Lpgat1	-0.02608	0.326386	1
Zfp771	-0.02608	0.249137	1
Klc2	-0.02622	0.318603	1
Gpbp1l1	-0.02625	0.613619	1
Primpol	-0.02629	0.144811	1
Mtln	-0.02641	0.968707	1
Uqcrc2	-0.02647	0.933606	1
Thumpd3	-0.02648	0.230455	1
Kras	-0.02649	0.792928	1

Kmt2d	-0.02653	0.452855	1
Trpv2	-0.02655	0.327276	1
Gga3	-0.02657	0.60624	1
Saal1	-0.0266	0.784304	1
Bcl2l12	-0.02663	0.947734	1
Cmpk1	-0.02665	0.342239	1
Uty	-0.02665	0.251682	1
Rapsn	-0.02666	0.0445	1
Grpel1	-0.02666	0.911807	1
Mast4	-0.02674	0.053623	1
Eif2s3y	-0.02678	0.251207	1
Vps33a	-0.02683	0.293563	1
Sprtn	-0.02689	0.781253	1
Pdzd11	-0.0269	0.403181	1
Hmgxb3	-0.02694	0.168234	1
Stk16	-0.02698	0.095486	1
Ciao2b	-0.02702	0.333804	1
Dhx29	-0.02702	0.808038	1
Mphosph6	-0.02702	0.079681	1
Vti1b	-0.02703	0.285917	1
Rc3h1	-0.02705	0.221376	1
Galnt1	-0.02707	0.289745	1
Jmjd8	-0.02707	0.370999	1
Ric8a	-0.02713	0.355265	1
Atp5f1	-0.02719	0.912908	1
Zhx1	-0.02719	0.113304	1
Tmtc3	-0.02723	0.559044	1
Anapc15	-0.02724	0.002264	1
Ncoa6	-0.02738	0.867456	1
Grpel2	-0.02743	0.971787	1
Gigyf2	-0.02755	0.283146	1
Mtrex	-0.02759	0.924502	1
Amz1	-0.02762	0.059164	1
Selenot	-0.02763	0.395183	1
Zfp146	-0.02764	0.569188	1
Tmem243	-0.02765	0.455189	1
Paxx	-0.02778	0.429101	1
Akap13	-0.02779	0.263649	1
Pitpnc1	-0.02785	0.126373	1
Nudt5	-0.02788	0.354459	1
Stk17b	-0.02791	0.190845	1
Setd2	-0.02792	0.69115	1
Hspa14	-0.02794	0.364774	1
Phyh	-0.02795	0.243373	1
Aifm1	-0.02798	0.149506	1
Nutf2	-0.028	0.459373	1
Imp3	-0.02802	0.941194	1
Abt1	-0.02802	0.764769	1

Sac3d1	-0.02812	0.49694	1
Tcp11l1	-0.02818	0.307622	1
Fra10ac1	-0.02825	0.854799	1
Pdcd2l	-0.02826	0.99319	1
Ints5	-0.02831	0.987184	1
Them6	-0.02832	0.708956	1
Amn1	-0.02843	0.664285	1
Dhx33	-0.02853	0.255328	1
Ptbp2	-0.02857	0.430562	1
Imp4	-0.0286	0.107967	1
Wdr82	-0.02862	0.798653	1
Klhl26	-0.02863	0.704967	1
Hpf1	-0.02865	0.262522	1
Hsf1	-0.02865	0.963294	1
Chchd7	-0.02866	0.52297	1
Usp40	-0.02866	0.441172	1
Dctn6	-0.02866	0.64827	1
Tasp1	-0.02867	0.110234	1
Snx8	-0.02868	0.79979	1
Cep120	-0.02881	0.612936	1
Pqlc2	-0.02884	0.339488	1
Commd1	-0.02885	0.34986	1
Golph3l	-0.02894	0.594745	1
C330018D:	-0.02896	0.93425	1
Ptdss1	-0.02904	0.100382	1
Rnmt	-0.02905	0.199317	1
Dph7	-0.02905	0.844474	1
Brwd3	-0.0291	0.370823	1
Phrf1	-0.02915	0.555908	1
Depdc5	-0.02922	0.472149	1
Nmnat3	-0.02923	0.391069	1
Lrrc57	-0.02926	0.688292	1
Pdpk1	-0.02937	0.513077	1
Pfdn2	-0.02942	0.52482	1
Kansl3	-0.02944	0.008007	1
Smarcad1	-0.0295	0.882222	1
Nxt1	-0.02951	0.576159	1
Tmem18	-0.02953	0.999543	1
Nectin4	-0.02954	0.030392	1
Rnf40	-0.0296	0.482572	1
Tti2	-0.02961	0.940846	1
Yif1a	-0.02964	0.485935	1
Mul1	-0.02966	0.326569	1
Ndufs7	-0.02968	0.613375	1
Anks3	-0.02968	0.701787	1
Slc41a3	-0.02973	0.951413	1
Fgfr1op2	-0.02977	0.510536	1
Pop5	-0.02978	0.42087	1

Asah2	-0.02987	0.184764	1
Fip1l1	-0.02996	0.841503	1
Pafah1b2	-0.02997	0.400235	1
Smim4	-0.03003	0.257604	1
Acadvl	-0.03003	0.45452	1
Copg1	-0.0301	0.065212	1
Tmem185a	-0.03018	0.411881	1
Ibtk	-0.03019	0.206225	1
Nlrp1a	-0.03021	0.008865	1
Cct4	-0.03022	0.881924	1
Rnf10	-0.03023	0.144497	1
Med4	-0.03031	0.546597	1
Ptcd2	-0.03043	0.792512	1
Fam53a	-0.03043	0.676847	1
Pdlim2	-0.03044	0.631068	1
Rnf185	-0.03044	0.157785	1
Haus3	-0.03051	0.459101	1
Paip1	-0.03053	0.508116	1
Cep135	-0.03054	0.27789	1
E2f4	-0.03062	0.990154	1
Rrn3	-0.03063	0.168273	1
Cwc22	-0.03068	0.610168	1
Prr14l	-0.03069	0.653615	1
Dhx36	-0.03071	0.362011	1
Uggt1	-0.03071	0.709025	1
Ecd	-0.03073	0.500057	1
Osbpl7	-0.03078	0.325998	1
Fcho2	-0.0308	0.747549	1
Ino80e	-0.03082	0.292642	1
Gm1976	-0.03089	0.547934	1
1810030O0	-0.0309	0.526959	1
Zfp87	-0.0309	0.937166	1
Hdac3	-0.03095	0.863552	1
Decr1	-0.03095	0.92163	1
9330159M	-0.03101	0.106071	1
Wdr61	-0.03105	0.101105	1
Chst12	-0.03106	0.40006	1
Sccpdh	-0.0311	0.540043	1
Cactin	-0.03117	0.594884	1
Arfrp1	-0.03119	0.176942	1
Ddx3y	-0.03121	0.674035	1
Nudt13	-0.03122	0.303878	1
Nde1	-0.03122	0.322891	1
Pias4	-0.0313	0.265522	1
Rnasek	-0.03133	0.902755	1
Ash2l	-0.03136	0.962731	1
BC005561	-0.03139	0.133804	1
Dnajb9	-0.03142	0.548513	1

Npepps	-0.03144	0.314038	1
Zfp597	-0.03147	0.926436	1
Pigf	-0.0315	0.074159	1
Rgs3	-0.03153	0.004127	1
Mettl16	-0.03157	0.537536	1
Ap2m1	-0.03158	0.875303	1
2510039O	-0.03162	0.711545	1
Cox19	-0.03167	0.897827	1
Actr5	-0.03168	0.911789	1
Cdc16	-0.0317	0.448291	1
Eci1	-0.03171	0.730761	1
Miip	-0.03174	0.28826	1
Mrpl53	-0.03179	0.667651	1
Cfap36	-0.03183	0.514904	1
Taf1b	-0.03185	0.329084	1
Slc36a1	-0.03187	0.102478	1
Pycrl	-0.0319	0.174931	1
Nfe2l1	-0.03193	0.460931	1
Lamtor2	-0.03196	0.176782	1
Phykpl	-0.03199	0.975018	1
Sbf1	-0.032	0.463037	1
Fyco1	-0.03212	0.533045	1
Cuta	-0.03216	0.412044	1
Rbm45	-0.03218	0.735753	1
Gpr157	-0.03219	0.854055	1
Gfpt1	-0.03221	0.146576	1
Hspbp1	-0.03222	0.14776	1
Slx4ip	-0.03231	0.083309	1
Mnat1	-0.03248	0.053038	1
Cdc37l1	-0.03248	0.716223	1
Mrps14	-0.03249	0.448742	1
Nup107	-0.0325	0.639029	1
Recql	-0.03253	0.456607	1
Trim33	-0.03255	0.967854	1
Gng2	-0.03257	0.920307	1
Preb	-0.03279	0.434762	1
Caap1	-0.03279	0.814512	1
Tbc1d32	-0.03283	0.954678	1
Brd4	-0.03285	0.283228	1
Pptc7	-0.03292	0.206876	1
Adprhl2	-0.03297	0.953585	1
Dpm1	-0.03297	0.320009	1
Nsf	-0.03302	0.458833	1
Cpt1a	-0.03308	0.382884	1
Scai	-0.0331	0.005894	1
Dhx16	-0.03311	0.954131	1
Eif2b1	-0.03319	0.791784	1
Snrpb	-0.03319	0.843717	1

Zfp26	-0.0332	0.256412	1
Wdr11	-0.03322	0.802409	1
Sec24d	-0.03324	0.637783	1
Mrps6	-0.03327	0.529703	1
Vps53	-0.03339	0.120638	1
Dynlt1c	-0.03344	0.213741	1
8030462N1	-0.03344	0.985303	1
Mtmr6	-0.03345	0.445579	1
Rinl	-0.03353	0.033606	1
Taf8	-0.03356	0.695373	1
Yipf6	-0.03365	0.998357	1
Mtch1	-0.03366	0.246315	1
Zfp709	-0.03371	0.160132	1
Fam98a	-0.03374	0.93335	1
Zfp943	-0.03378	0.703985	1
Zfand1	-0.03381	0.912265	1
Tomm34	-0.03383	0.425188	1
Trappc1	-0.03383	0.330465	1
Rufy2	-0.03384	0.945244	1
Cnih1	-0.03384	0.196083	1
Zdhhc9	-0.03388	0.975584	1
Trappc5	-0.03389	0.599325	1
Cyth2	-0.03393	0.755832	1
Nr1d2	-0.03395	0.107696	1
Necap1	-0.03397	0.952363	1
Spata1	-0.03399	0.677605	1
Rictor	-0.03399	0.377919	1
Kat6b	-0.03409	0.102098	1
Cdk4	-0.0341	0.663327	1
Polr2g	-0.0341	0.364278	1
Stk25	-0.03415	0.982567	1
Fkrp	-0.03421	0.799198	1
Ift140	-0.03427	0.806587	1
Nol7	-0.03429	0.225088	1
Agtpbp1	-0.0343	0.108178	1
Rad54l2	-0.03432	0.874756	1
Ap5m1	-0.03433	0.74873	1
Rfk	-0.03434	0.960128	1
Zfp991	-0.03436	0.513818	1
Trim44	-0.03442	0.222767	1
Ghitm	-0.03442	0.312764	1
Setd1a	-0.03442	0.161921	1
Tmem19	-0.03444	0.923477	1
Chtop	-0.03449	0.700656	1
Chst1	-0.03449	0.403395	1
Arhgap30	-0.03454	0.043607	1
Foxj2	-0.03458	0.748982	1
Tsga10	-0.0346	0.459442	1

Bfar	-0.0346	0.02833	1
Efr3a	-0.03461	0.853542	1
Tcf25	-0.03463	0.760406	1
Clk1	-0.0347	0.657802	1
Orc2	-0.03471	0.482629	1
Furin	-0.03474	0.112643	1
Cant1	-0.03488	0.685204	1
Mfn2	-0.03491	0.586512	1
Rasa1	-0.03505	0.362618	1
Pex7	-0.03507	0.706345	1
Cpt2	-0.03507	0.378506	1
Tbc1d13	-0.03513	0.386224	1
Mettl25	-0.03513	0.366377	1
Chmp2a	-0.03514	0.915219	1
Foxk1	-0.03515	0.844483	1
Mpdu1	-0.03515	0.981422	1
Rnf181	-0.03518	0.205781	1
Gtpbp8	-0.03519	0.541526	1
Hes6	-0.03521	0.862413	1
Fkbp3	-0.03525	0.800594	1
Cbr4	-0.03526	0.637373	1
Zfp207	-0.03532	0.498175	1
Zfyve1	-0.03533	0.895444	1
Sgpl1	-0.03535	0.27551	1
Stxbp1	-0.03543	0.946714	1
Khnyn	-0.03548	0.6128	1
Cdc42bpb	-0.0355	0.007914	1
Malt1	-0.03551	0.089364	1
Terf2ip	-0.03552	0.068571	1
Sap30bp	-0.03556	0.221133	1
Anp32e	-0.03556	0.832914	1
Gpr108	-0.03556	0.138548	1
Pigx	-0.03561	0.658736	1
Zfp688	-0.03569	0.618821	1
Zfp182	-0.03573	0.514776	1
Krit1	-0.03578	0.135753	1
Hdac1	-0.0358	0.502162	1
Stat5a	-0.03581	0.407148	1
Zfp444	-0.03581	0.733384	1
Cep192	-0.03587	0.322337	1
Mrpl44	-0.03588	0.525088	1
Ddx52	-0.03598	0.75497	1
Med27	-0.03601	0.284117	1
Nacc2	-0.03607	0.515418	1
Coa6	-0.0361	0.858156	1
Uck1	-0.03615	0.675686	1
Dtx2	-0.03623	0.215563	1
Mus81	-0.03632	0.142061	1

Sf3a3	-0.03633	0.826991	1
Eaf1	-0.03637	0.406607	1
Trmt2b	-0.03639	0.69075	1
Psmg2	-0.03641	0.474452	1
Foxk2	-0.03642	0.798191	1
Abca3	-0.03644	0.319915	1
Vps45	-0.03647	0.323806	1
Bet1	-0.03651	0.996914	1
Mettl3	-0.03659	0.955878	1
1810037I1	-0.03661	0.94263	1
Ttc14	-0.03668	0.638689	1
Gmcl1	-0.03671	0.882484	1
4931406P1	-0.03672	0.439276	1
Lin7c	-0.03676	0.731297	1
Dicer1	-0.03676	0.21864	1
Zfyve16	-0.03687	0.919141	1
Ipo5	-0.03692	0.329341	1
Gapvd1	-0.03694	0.83843	1
Poglut1	-0.03698	0.518859	1
Osbpl1a	-0.037	0.788635	1
Eya3	-0.03703	0.342089	1
Phactr4	-0.03716	0.508632	1
Ppp6r3	-0.03722	0.664686	1
Usp48	-0.03727	0.274129	1
Map2k5	-0.03728	0.503164	1
Slc4a1ap	-0.03728	0.837728	1
Eny2	-0.03729	0.462175	1
Mtmr9	-0.03735	0.876313	1
Ptp4a2	-0.03736	0.285061	1
Brpf1	-0.03741	0.206809	1
Klhl18	-0.03742	0.370125	1
Malat1	-0.03743	0.00096	1
Tmem147	-0.03746	0.221437	1
Slc35b4	-0.03747	0.882514	1
Dnajc30	-0.03752	0.468307	1
2900097C1	-0.03754	0.322904	1
Rcl1	-0.0376	0.2934	1
1700037C1	-0.03762	0.551166	1
Mllt11	-0.03766	0.570463	1
Tmem127	-0.03768	0.792246	1
Slc35a4	-0.0378	0.302852	1
Med10	-0.03789	0.481119	1
Clns1a	-0.03791	0.658103	1
Ap4e1	-0.03793	0.682856	1
Cops5	-0.03794	0.289257	1
Pelp1	-0.03796	0.210479	1
Ngdn	-0.03799	0.799701	1
Lsm8	-0.038	0.827232	1

Rbm8a	-0.03803	0.796482	1
Mrps5	-0.03811	0.528809	1
Man2c1	-0.03822	0.870173	1
4833420G:	-0.03827	0.380815	1
Atf1	-0.03828	0.168952	1
Fbxw9	-0.03831	0.801231	1
Pdpr	-0.03833	0.588324	1
Gtf3c6	-0.03836	0.130368	1
Zbtb43	-0.0385	0.587572	1
Tmem260	-0.03854	0.034059	1
Rdh14	-0.03863	0.288206	1
Actr10	-0.03864	0.892259	1
Zfp738	-0.03865	0.834395	1
Ttc9c	-0.03866	0.718417	1
Pum1	-0.03868	0.971831	1
Zfp592	-0.03869	0.625038	1
Srsf1	-0.03869	0.251018	1
Lrrc8a	-0.0387	0.508591	1
Ercc6l2	-0.03875	0.75152	1
Cox11	-0.03882	0.393034	1
Snhg20	-0.03885	0.141125	1
Zfp512	-0.03885	0.728855	1
Nlrx1	-0.03889	0.780999	1
Nr2c1	-0.03892	0.276584	1
Tlr4	-0.03892	0.210167	1
Alas1	-0.03892	0.846595	1
Zfp692	-0.03902	0.706895	1
Lcor	-0.03906	0.228654	1
Leng1	-0.03907	0.444258	1
Exoc2	-0.03911	0.573994	1
Smyd5	-0.03919	0.624287	1
Gm9725	-0.03921	0.917648	1
Abcd4	-0.03925	0.602	1
Cspp1	-0.03927	0.073746	1
Usp45	-0.03928	0.775898	1
Tab1	-0.03931	0.484726	1
Sar1b	-0.03932	0.88767	1
Dcaf1	-0.03942	0.775956	1
Pds5a	-0.03946	0.490105	1
Rnf25	-0.03946	0.345939	1
Tmem14c	-0.03948	0.729208	1
Wipi2	-0.03953	0.733086	1
Sptlc1	-0.03962	0.795242	1
Nfkbie	-0.03964	0.303972	1
Tbl1x	-0.03965	0.828922	1
Mrps18c	-0.03965	0.199777	1
Zfp839	-0.03967	0.877009	1
3110040N:	-0.03967	0.943035	1

Zranb1	-0.03968	0.668382	1
Cstf2	-0.03969	0.357702	1
Nupr1	-0.0397	0.307487	1
Rps6kc1	-0.03971	0.058147	1
Tmem131l	-0.03971	0.310926	1
Terf1	-0.03975	0.481402	1
Uhmk1	-0.03985	0.702064	1
Eri3	-0.03991	0.072235	1
Ostm1	-0.03997	0.31646	1
Desi1	-0.03998	0.858798	1
Rapgef6	-0.04002	0.255194	1
Fbxl6	-0.04011	0.90001	1
Mrtfa	-0.04016	0.846619	1
Chmp7	-0.04017	0.074417	1
Nubpl	-0.04017	0.842547	1
Serpinb6a	-0.04018	0.877562	1
Cep83os	-0.04025	0.943041	1
Pfdn6	-0.04026	0.173821	1
St3gal3	-0.04028	0.165282	1
Eif2d	-0.04029	0.889345	1
Scnm1	-0.04032	0.502443	1
Lcp1	-0.04043	0.092585	1
Ap4b1	-0.04044	0.366287	1
Cops3	-0.04045	0.873132	1
Nup133	-0.04051	0.805137	1
Dpp9	-0.04053	0.463564	1
Gm34280	-0.04055	0.327842	1
Mtx2	-0.04057	0.653679	1
Ube2q1	-0.04063	0.555097	1
Acaa2	-0.04064	0.785932	1
Wdr92	-0.0407	0.290648	1
Desi2	-0.0407	0.210799	1
Cops6	-0.04071	0.437186	1
Gpd1l	-0.04077	0.291912	1
Mdh1	-0.04079	0.527487	1
Lrrc3	-0.0408	0.621493	1
Shld1	-0.04081	0.975551	1
1600010M	-0.04082	0.658563	1
Lcorl	-0.04085	0.318014	1
Ankrd40	-0.04088	0.890688	1
Sh2b1	-0.04092	0.515841	1
Zfp944	-0.04096	0.574169	1
Zyx	-0.04099	0.196868	1
Hadhb	-0.04102	0.421085	1
Tpm1	-0.04111	0.182042	1
Timm44	-0.04125	0.121233	1
Zzz3	-0.04125	0.831561	1
Nt5dc1	-0.04127	0.772111	1

Golim4	-0.04131	0.23306	1
Xpo4	-0.04138	0.860698	1
Trp53i13	-0.04149	0.406592	1
Bmpr1a	-0.0415	0.156813	1
Smim11	-0.0415	0.719643	1
Jagn1	-0.04151	0.300043	1
5730455P1	-0.04152	0.860681	1
5530601H0	-0.04153	0.656537	1
Ubald2	-0.04155	0.951047	1
Med25	-0.04155	0.369704	1
Ppp4r3b	-0.0416	0.983813	1
Tpgs1	-0.04169	0.709573	1
Zfp639	-0.04169	0.963973	1
Btbd10	-0.04172	0.728527	1
Tifa	-0.04173	0.577052	1
Trabd	-0.04179	0.586776	1
Arhgap1	-0.04181	0.843574	1
Uqcrc1	-0.04186	0.860675	1
Thoc7	-0.04188	0.703354	1
Map2k3	-0.04191	0.392252	1
Vcpip1	-0.04195	0.848032	1
Rmnd1	-0.04204	0.194052	1
Ppil4	-0.04209	0.51981	1
Ypel5	-0.04212	0.760578	1
Apopt1	-0.04214	0.949744	1
Plcb2	-0.0422	0.622306	1
Pck2	-0.04222	0.493071	1
1600020E0	-0.04227	0.070257	1
Pigg	-0.04229	0.750066	1
Mrpl39	-0.04238	0.409604	1
Dld	-0.04241	0.735521	1
Mbip	-0.04242	0.836991	1
Nrp2	-0.04244	0.550006	1
Marcksl1	-0.04245	0.865128	1
Ltn1	-0.04249	0.829119	1
1110059E2	-0.04263	0.410136	1
Alox5	-0.04285	0.3893	1
Gcc2	-0.04289	0.107174	1
Sgpp1	-0.04294	0.497573	1
Dynlt3	-0.04296	0.371953	1
Atpsckmt	-0.04309	0.981641	1
Lemd3	-0.04313	0.482098	1
Tdrd3	-0.04314	0.729463	1
Crtc3	-0.04315	0.086442	1
Mrpl22	-0.04326	0.810261	1
Malsu1	-0.04328	0.295977	1
Herpud2	-0.04333	0.170157	1
Wbp1l	-0.04333	0.58256	1

St7l	-0.04334	0.209216	1
Yipf2	-0.04337	0.345304	1
Acad8	-0.04342	0.862083	1
Larp7	-0.04348	0.175085	1
Cacul1	-0.0435	0.74896	1
Med30	-0.04354	0.751089	1
Vamp7	-0.04355	0.991857	1
Lin52	-0.04356	0.895798	1
Znrd1	-0.04357	0.948188	1
Invs	-0.04361	0.870347	1
Mindy3	-0.04363	0.678753	1
Vps51	-0.04372	0.808364	1
Orc4	-0.04383	0.959011	1
Mthfd1	-0.04384	0.891891	1
Cetn3	-0.0439	0.829536	1
Nsmce3	-0.04394	0.559686	1
Ccdc28b	-0.04395	0.955855	1
Trappc10	-0.04396	0.794958	1
Lipo3	-0.04399	0.464983	1
Rbbp5	-0.04407	0.874534	1
BC052040	-0.04409	0.952875	1
Ncbp1	-0.04417	0.927232	1
Eya4	-0.04423	0.112628	1
Erlec1	-0.04424	0.771109	1
Hdgfl2	-0.04425	0.746661	1
Ehmt1	-0.0443	0.046982	1
Vdac1	-0.04431	0.520494	1
Firre	-0.04431	0.237837	1
Dnajc10	-0.04436	0.754169	1
Kdm3a	-0.04442	0.856982	1
Mnt	-0.04443	0.439419	1
Psmd10	-0.04451	0.967463	1
Specc1l	-0.04453	0.061116	1
Ncstn	-0.04463	0.817696	1
Hadh	-0.04464	0.775834	1
Mprip	-0.04471	0.347371	1
Ostc	-0.04478	0.517674	1
2310033P(	-0.04479	0.626042	1
Zfp74	-0.04484	0.075877	1
Nudt6	-0.04484	0.438931	1
Brd1	-0.04488	0.947865	1
Bud13	-0.04488	0.975462	1
Snx25	-0.0449	0.996533	1
Actb	-0.0449	0.000187	1
Ift172	-0.04491	0.359562	1
Atxn10	-0.04494	0.866995	1
Gm14325	-0.04497	0.994871	1
Pias2	-0.04498	0.066556	1

Adck5	-0.04505	0.633254	1
Ube2q2	-0.04506	0.265689	1
Atf2	-0.04506	0.887271	1
Hmces	-0.04507	0.666374	1
Tmem241	-0.04512	0.828716	1
Piezo1	-0.04512	0.958947	1
Stam2	-0.04522	0.448347	1
Wrap73	-0.04523	0.098004	1
Herc1	-0.04523	0.488334	1
Neurl1a	-0.04527	0.583627	1
Cnp	-0.04529	0.572831	1
Tnpo3	-0.04531	0.308225	1
Exosc3	-0.04531	0.739263	1
Ctnnbl1	-0.04534	0.332022	1
Hccs	-0.04536	0.285663	1
5430405H	-0.04538	0.860713	1
Ndufa9	-0.04539	0.267992	1
Socs6	-0.0454	0.758571	1
Psmg3	-0.04541	0.601394	1
Med6	-0.04542	0.194885	1
Smim26	-0.04545	0.821169	1
Kbtbd4	-0.04545	0.106374	1
Arhgap9	-0.04546	0.827888	1
Uckl1	-0.04547	0.992895	1
Mis12	-0.04549	0.239614	1
Pspc1	-0.04555	0.435923	1
Snx9	-0.04557	0.166197	1
Apex2	-0.04558	0.84692	1
Pxdc1	-0.04565	0.902885	1
Ccdc124	-0.0457	0.386679	1
Tom1	-0.04576	0.712606	1
Klc1	-0.04584	0.021313	1
Ap2a2	-0.04589	0.3194	1
Cc2d1a	-0.04591	0.425469	1
Rmdn1	-0.04591	0.853974	1
Ubxn8	-0.04594	0.874349	1
Emd	-0.04595	0.236548	1
Ino80d	-0.04598	0.947335	1
Pcnx	-0.04599	0.962686	1
Cse1l	-0.04599	0.441017	1
2810004N	-0.04602	0.55238	1
Mcm3ap	-0.04608	0.723623	1
Wdr37	-0.04612	0.638672	1
Arl2	-0.04613	0.607569	1
App	-0.04618	0.763837	1
Sbds	-0.04622	0.605796	1
Edem3	-0.04625	0.506588	1
Clcn6	-0.04627	0.519577	1

Klhl28	-0.04629	0.597613	1
Xrcc1	-0.04631	0.662049	1
Proser1	-0.04632	0.77251	1
Ehmt2	-0.04634	0.649185	1
Thada	-0.04634	0.641073	1
Adh5	-0.04635	0.894773	1
Prr13	-0.04637	0.849675	1
Dock7	-0.0465	0.198758	1
Prmt5	-0.04661	0.204293	1
Sepsecs	-0.04663	0.496393	1
Tcf12	-0.04666	0.698599	1
Ezh1	-0.04668	0.915019	1
Rnf146	-0.04671	0.553004	1
Yeats4	-0.04672	0.415586	1
Mpv17l2	-0.04677	0.566708	1
Dag1	-0.04682	0.540067	1
Klhl20	-0.04685	0.421773	1
Dnajc1	-0.04689	0.964806	1
Cherp	-0.04694	0.20284	1
Zbtb45	-0.04694	0.549255	1
Ccdc91	-0.04698	0.577508	1
Hnrnpl	-0.04699	0.190339	1
Zyg11b	-0.04707	0.394945	1
Sike1	-0.04709	0.350722	1
Ascc1	-0.0471	0.857969	1
Ddx19a	-0.04714	0.949014	1
Zfp276	-0.0472	0.690909	1
Ubn2	-0.04721	0.878143	1
Snw1	-0.04728	0.493373	1
Dusp12	-0.04733	0.839499	1
Zfp619	-0.04736	0.276481	1
Pank3	-0.04737	0.773573	1
Smg8	-0.04738	0.584975	1
Dnajc19	-0.04741	0.181854	1
Cwf19l2	-0.04743	0.072762	1
Rhbdd2	-0.04744	0.281417	1
Ogdh	-0.04745	0.995625	1
Mrpl58	-0.04746	0.192148	1
Ubl7	-0.04754	0.711681	1
Arrdc1	-0.0476	0.563855	1
Dus1l	-0.04768	0.592011	1
Clpb	-0.0477	0.703115	1
Fbxl5	-0.04777	0.533197	1
Dap	-0.04777	0.711397	1
Arfgef2	-0.04782	0.196552	1
Nmral1	-0.04783	0.904486	1
G6pdx	-0.04788	0.807375	1
Washc5	-0.04794	0.172015	1

Taf1c	-0.04796	0.926453	1
Ipo9	-0.04797	0.488209	1
Pacs2	-0.048	0.093274	1
Zfp445	-0.04801	0.890557	1
Nudt22	-0.04806	0.974669	1
Ppm1d	-0.04807	0.547684	1
Skap2	-0.04808	0.36724	1
Ppp3cb	-0.04818	0.742598	1
Rps6ka3	-0.04818	0.336858	1
Zmat2	-0.04822	0.445947	1
Asb7	-0.04829	0.77071	1
Cep250	-0.04832	0.840584	1
Hdgf	-0.04835	0.519981	1
A530088E(	-0.04843	0.808926	1
Naglu	-0.04848	0.776543	1
Arpc2	-0.04848	0.110813	1
Gabpa	-0.0485	0.771971	1
Akt2	-0.04852	0.772637	1
Apc	-0.04856	0.528945	1
Adk	-0.04857	0.588904	1
Tmem214	-0.04866	0.533688	1
Arhgap21	-0.04869	0.983884	1
Bcap31	-0.04872	0.394945	1
Smad7	-0.04874	0.293349	1
Ralgapa1	-0.04876	0.70295	1
Spen	-0.04877	0.967711	1
Zfp524	-0.04878	0.891982	1
Vrk3	-0.04878	0.859409	1
Fbxo9	-0.04879	0.642901	1
Srsf9	-0.04888	0.721261	1
Atp9b	-0.0489	0.561014	1
Prdm1	-0.04891	0.205787	1
Eif4a2	-0.04897	0.762068	1
Elavl4	-0.04897	0.551374	1
Sarnp	-0.04898	0.841116	1
Gm42962	-0.049	0.584463	1
Afap1l1	-0.049	0.852984	1
Ndufs1	-0.04909	0.715817	1
Ap1m1	-0.0491	0.503592	1
Sec23ip	-0.04911	0.932348	1
Leo1	-0.04911	0.948695	1
Edc3	-0.04916	0.542717	1
Synj2bp	-0.04917	0.67235	1
Stx5a	-0.0492	0.710622	1
D8Ertd738	-0.04921	0.636795	1
Mtmr4	-0.04928	0.888443	1
Ccnt1	-0.04929	0.867261	1
Ptprs	-0.0493	0.868875	1

Elf1	-0.04932	0.231368	1
Arhgap18	-0.04933	0.558515	1
Nt5c2	-0.04933	0.781972	1
Abhd17b	-0.04936	0.71093	1
Ppp1r15b	-0.04941	0.827927	1
Armc1	-0.04954	0.757345	1
Smim12	-0.04954	0.042939	1
Ppp1r12b	-0.04955	0.952791	1
Zfp942	-0.04961	0.774082	1
Cd9	-0.04962	0.158904	1
Ddx31	-0.04963	0.054556	1
Gm13562	-0.04966	0.866171	1
Gsg1	-0.04971	0.657639	1
B3gat3	-0.04975	0.814853	1
Ints8	-0.04978	0.779665	1
Scyl1	-0.04979	0.664911	1
Phtf2	-0.04981	0.831589	1
Mgat4b	-0.04983	0.98424	1
Kcnq1ot1	-0.04995	0.036093	1
Fam149b	-0.05006	0.822393	1
Alg6	-0.05013	0.620792	1
Hsd17b10	-0.05013	0.820778	1
Cetn2	-0.05015	0.904234	1
Nup214	-0.05027	0.748676	1
Ssrp1	-0.0503	0.629242	1
Atg10	-0.05032	0.552235	1
Usp8	-0.05033	0.550724	1
Inppl1	-0.05034	0.450268	1
2610020C(	-0.05038	0.164318	1
Zdhhc24	-0.05038	0.71234	1
Zc3h14	-0.05041	0.345668	1
Fam76a	-0.05043	0.583042	1
Ube2b	-0.05044	0.521357	1
Zscan25	-0.05056	0.155655	1
Capn7	-0.05059	0.652803	1
Irf9	-0.05062	0.98241	1
Nipbl	-0.05067	0.834461	1
Cir1	-0.05071	0.506659	1
Klc4	-0.05071	0.794628	1
Gm5617	-0.05074	0.362851	1
Smc1a	-0.05077	0.249822	1
Pef1	-0.05083	0.477109	1
Mtmr2	-0.05087	0.76065	1
Rheb	-0.05102	0.482135	1
Uevld	-0.05103	0.665475	1
Atg13	-0.05119	0.409496	1
Cdk5	-0.05126	0.697631	1
Prkcd	-0.0513	0.983128	1

Lnpk	-0.05132	0.615981	1
2410022M	-0.05139	0.270212	1
Ntan1	-0.0514	0.537054	1
Lyn	-0.05144	0.492377	1
Zfp511	-0.05153	0.865272	1
Ndufaf3	-0.05155	0.420994	1
Impact	-0.0517	0.040844	1
Cyth3	-0.05172	0.256325	1
Nkap	-0.05173	0.683843	1
Tceal9	-0.05173	0.824894	1
Lemd2	-0.05175	0.299687	1
Gtf3a	-0.05183	0.392497	1
Rab11fip5	-0.05187	0.903195	1
Ppp1r10	-0.05189	0.851328	1
D330023K	-0.05192	0.231909	1
Dmac2	-0.05193	0.290267	1
Cfl2	-0.05194	0.910701	1
Alkbh3	-0.05199	0.909713	1
Ttpal	-0.05203	0.759324	1
Nhlrc3	-0.05205	0.873555	1
Zzef1	-0.05207	0.623096	1
Mcee	-0.05207	0.964312	1
Pex19	-0.05208	0.783382	1
Pcgf1	-0.05208	0.782003	1
Rcan3	-0.0521	0.647257	1
Rspry1	-0.05211	0.616082	1
Slc7a6os	-0.05217	0.670197	1
Gpbp1	-0.05219	0.712907	1
Nek9	-0.05225	0.882568	1
Zdhhc6	-0.05225	0.995652	1
Cnbp	-0.05225	0.177198	1
Ccs	-0.05225	0.338593	1
Dpagt1	-0.05231	0.960234	1
Wasl	-0.05233	0.718856	1
Akr1e1	-0.05234	0.314534	1
Irf5	-0.05238	0.199956	1
Rbm15	-0.0524	0.620988	1
Ice2	-0.05247	0.791258	1
Phf8	-0.05254	0.944609	1
Slc17a9	-0.05255	0.207369	1
Gm49336	-0.05268	0.528815	1
Actg1	-0.05275	0.023683	1
Myo6	-0.05286	0.682115	1
Copb2	-0.0529	0.699695	1
Chst11	-0.05291	0.002865	1
Sfxn3	-0.05293	0.479561	1
Frs2	-0.05297	0.687955	1
Lrif1	-0.05298	0.890766	1

Ttc32	-0.0531	0.506968	1
Slc30a9	-0.0531	0.757025	1
Prpsap1	-0.05313	0.235028	1
Mynn	-0.05321	0.20459	1
Hsdl1	-0.05324	0.302303	1
Dnajc8	-0.05332	0.665212	1
Orc5	-0.05333	0.550856	1
Ormdl3	-0.05333	0.196885	1
Crcp	-0.05338	0.87369	1
Acat1	-0.05341	0.656364	1
Shkbp1	-0.0535	0.611329	1
Ttc8	-0.05351	0.276727	1
Strada	-0.05353	0.860771	1
Ccar2	-0.05357	0.533187	1
Arhgap35	-0.05365	0.863798	1
Ttc4	-0.05366	0.684459	1
Trim28	-0.0537	0.929188	1
Cdc27	-0.05373	0.736822	1
Hnrnpa2b1	-0.05375	0.410665	1
Rnf2	-0.05389	0.108258	1
Zscan21	-0.0539	0.224933	1
Mccc2	-0.05391	0.726144	1
Mtg2	-0.05392	0.678876	1
Aqr	-0.05393	0.813602	1
Sumf2	-0.05397	0.917599	1
Cltb	-0.05398	0.289673	1
Polr3f	-0.05399	0.156041	1
Tirap	-0.05405	0.167932	1
Vdac3	-0.05406	0.778584	1
Sec61a1	-0.05409	0.497753	1
Galk2	-0.05416	0.83561	1
Tmem229b	-0.05417	0.658633	1
Pitpnb	-0.05419	0.845461	1
Abhd2	-0.0542	0.393387	1
Smad2	-0.05431	0.823006	1
Rpp25l	-0.05436	0.805605	1
Tbrg1	-0.05437	0.194779	1
Vps52	-0.05438	0.637195	1
Aco1	-0.05445	0.781314	1
Atp13a3	-0.05449	0.974433	1
Elavl1	-0.05452	0.713382	1
Rmi1	-0.05453	0.471575	1
Spag9	-0.05453	0.933618	1
Pex3	-0.05455	0.96389	1
Lrrc41	-0.05456	0.578384	1
Uap1l1	-0.05459	0.938421	1
Suz12	-0.05459	0.566997	1
Gpr146	-0.0546	0.91243	1

2700062C(	-0.05461	0.993696	1
Camsap2	-0.05466	0.423623	1
Cnih4	-0.05469	0.864202	1
Top3a	-0.05473	0.180243	1
Rogdi	-0.05476	0.487011	1
Rnf38	-0.05477	0.255129	1
Chp1	-0.05477	0.568554	1
Gstz1	-0.0548	0.480286	1
St3gal4	-0.05483	0.66004	1
Lrrc58	-0.05486	0.778767	1
Fam192a	-0.05486	0.624463	1
Ckap5	-0.05491	0.601132	1
Txnrd2	-0.05496	0.57783	1
Cpsf7	-0.05498	0.452507	1
Slc39a9	-0.05499	0.469012	1
Higd2a	-0.05503	0.653977	1
Abcb7	-0.05505	0.939013	1
Cdkn1a	-0.05506	0.732592	1
Fbxw4	-0.05509	0.928597	1
Ttc5	-0.0551	0.818763	1
Rnf14	-0.05512	0.996804	1
Gm20528	-0.05512	0.373622	1
Gatd1	-0.05512	0.86517	1
Kdm5d	-0.05513	0.531718	1
Hdac11	-0.05517	0.19588	1
Ndufaf8	-0.05519	0.32501	1
Calu	-0.05524	0.839674	1
Pigm	-0.05525	0.82121	1
Ncaph2	-0.05527	0.919604	1
Hcfc1	-0.05544	0.386533	1
Usp37	-0.05548	0.670963	1
Mmadhc	-0.05555	0.830449	1
Stk4	-0.05555	0.069835	1
Zfp322a	-0.05557	0.924208	1
Hars2	-0.05559	0.133588	1
Fam126b	-0.05566	0.318573	1
Mfap1a	-0.0557	0.128257	1
Zfp868	-0.05572	0.853723	1
Homer3	-0.05573	0.426421	1
Mettl15	-0.05576	0.668195	1
Ap4s1	-0.05577	0.558579	1
Eif2s3x	-0.05583	0.911988	1
Wdr13	-0.05584	0.874398	1
Trappc6b	-0.05585	0.211362	1
Fggy	-0.05587	0.105176	1
Ube3b	-0.05589	0.396617	1
Chid1	-0.05601	0.793759	1
Zfp438	-0.05604	0.701282	1

Urod	-0.05606	0.300846	1
9930111J2	-0.05611	0.157063	1
Ik	-0.05612	0.488004	1
Phka1	-0.05614	0.369261	1
Itm2b	-0.05615	0.000205	1
Pde12	-0.05618	0.671966	1
Dolpp1	-0.05622	0.434977	1
Ugdh	-0.05626	0.570337	1
Dhx30	-0.05628	0.832017	1
Bid	-0.05629	0.780357	1
Rgmb	-0.05632	0.253353	1
Klhl5	-0.05641	0.347127	1
Plcg1	-0.05642	0.39466	1
Rbm14	-0.05642	0.91746	1
Fbxl12	-0.05643	0.794131	1
Eid1	-0.05645	0.596659	1
Gtf3c1	-0.05655	0.837128	1
Trp53bp2	-0.05666	0.710632	1
Tbc1d20	-0.05667	0.523373	1
Rnf103	-0.05678	0.30223	1
Ghdc	-0.05679	0.260669	1
Apex1	-0.05681	0.823438	1
Mtmr10	-0.0569	0.836353	1
Zfp280d	-0.05691	0.430628	1
Ninj1	-0.05691	0.511312	1
Map2k4	-0.05694	0.609047	1
E130307A1	-0.05694	0.72706	1
Rpp14	-0.05695	0.622327	1
Tmc6	-0.05697	0.721121	1
Rexo1	-0.05698	0.554491	1
Adck1	-0.05706	0.606468	1
Arid2	-0.05707	0.463129	1
Pmpcb	-0.05709	0.672005	1
Emc2	-0.05712	0.231736	1
Kif1bp	-0.05713	0.262175	1
Tmem199	-0.05722	0.749661	1
Ttc17	-0.05725	0.619445	1
Snrk	-0.05725	0.369243	1
Golga7	-0.0573	0.700143	1
C1d	-0.0573	0.659782	1
Pde4dip	-0.05732	0.177804	1
Tnrc6a	-0.05736	0.669113	1
Tom1l2	-0.0574	0.308125	1
Papolg	-0.0575	0.442103	1
Med16	-0.05752	0.957489	1
Rtl8c	-0.05759	0.167798	1
Dym	-0.05761	0.785558	1
Strap	-0.05767	0.112555	1

Rnf187	-0.05768	0.306791	1
Echdc1	-0.05769	0.450806	1
Pcm1	-0.05769	0.28485	1
Kctd12	-0.0578	0.006931	1
Fktn	-0.05781	0.832988	1
Tnfaip1	-0.05793	0.481359	1
Catspere2	-0.05796	0.918633	1
Gxylt1	-0.05796	0.803468	1
Trappc3	-0.05796	0.931946	1
Gm26782	-0.05797	0.642114	1
Gtf3c3	-0.05801	0.928069	1
Triap1	-0.05802	0.408745	1
Vps9d1	-0.05803	0.711268	1
Pip5k1a	-0.05811	0.952494	1
Rrp36	-0.05814	0.541729	1
Zfp951	-0.05814	0.991525	1
Snrnp40	-0.05814	0.47804	1
Sephs1	-0.05823	0.950914	1
Psmc3	-0.05837	0.470542	1
Ube2h	-0.05847	0.371959	1
Mark3	-0.05847	0.273994	1
Ndufb3	-0.05847	0.918112	1
Ube2j1	-0.05847	0.349364	1
Cpped1	-0.05854	0.479664	1
Maz	-0.05861	0.738113	1
Map3k4	-0.05866	0.676663	1
Clec16a	-0.05868	0.066582	1
Aen	-0.05874	0.721532	1
Pet100	-0.05881	0.467428	1
Twistnb	-0.05885	0.949611	1
Zfr	-0.05897	0.691401	1
Thumpd2	-0.05898	0.663692	1
C130050O	-0.05899	0.353657	1
Tef	-0.05904	0.860468	1
Cep70	-0.05905	0.420324	1
Mark4	-0.05915	0.561136	1
Hnrnpa0	-0.05924	0.531047	1
Prpf3	-0.05924	0.542891	1
Myg1	-0.05925	0.708849	1
Tfpt	-0.05926	0.944224	1
Plekho1	-0.05931	0.976227	1
Tcaim	-0.05933	0.228344	1
Acad9	-0.05933	0.295308	1
Entpd7	-0.05937	0.788772	1
Lamp2	-0.05939	0.241303	1
9530068E(	-0.0594	0.429366	1
Drosha	-0.05941	0.96423	1
Bub3	-0.05943	0.177702	1

Fbxo3	-0.05943	0.066297	1
Ppp4c	-0.05945	0.935416	1
Mettl21a	-0.05949	0.495797	1
Usp32	-0.05954	0.802223	1
Mlf2	-0.05958	0.774902	1
Pisd	-0.05961	0.575779	1
Smarcal1	-0.05963	0.969925	1
Spin1	-0.05968	0.407699	1
Uso1	-0.05971	0.857848	1
Slc17a5	-0.05974	0.737101	1
Pex14	-0.05975	0.802435	1
Nedd1	-0.05986	0.618223	1
Cdc42se1	-0.05991	0.168694	1
Lipe	-0.05993	0.791145	1
Tor2a	-0.05995	0.883872	1
Atg5	-0.05996	0.593955	1
Actr1b	-0.06005	0.715548	1
Nbas	-0.06008	0.209126	1
Pigb	-0.06017	0.756157	1
Nr2c2	-0.06019	0.557071	1
Med20	-0.06021	0.476674	1
Tyrobp	-0.06022	0.033198	1
Zfp799	-0.06023	0.250579	1
Ube2v2	-0.06023	0.939348	1
Psmd5	-0.06024	0.640889	1
Stub1	-0.06024	0.515071	1
Acbd3	-0.06034	0.546585	1
Armt1	-0.06035	0.035218	1
Axin1	-0.06036	0.899596	1
Dcaf4	-0.06036	0.200614	1
Kdm5c	-0.06046	0.707398	1
Vamp5	-0.06047	0.677693	1
Ankmy2	-0.06052	0.191989	1
Gkap1	-0.06053	0.854557	1
Snapc3	-0.06054	0.819547	1
Sgta	-0.06055	0.422824	1
Magoh	-0.06058	0.654653	1
Uba1	-0.06062	0.990039	1
Cpox	-0.06067	0.588626	1
Mphosph9	-0.06069	0.112279	1
Arnt	-0.06071	0.342397	1
Zfpl1	-0.06077	0.929119	1
Dhx35	-0.0608	0.123766	1
Rfc1	-0.06093	0.290236	1
Eef1akmt2	-0.06095	0.349044	1
Tsg101	-0.06097	0.897886	1
Evi2	-0.06105	0.039712	1
Aar2	-0.06111	0.163716	1

Thoc3	-0.06116	0.115238	1
Arl11	-0.06121	0.977701	1
Ubald1	-0.06121	0.172584	1
Ell	-0.06126	0.349619	1
Snrnp27	-0.06127	0.615563	1
Ankzf1	-0.06129	0.087679	1
Slc38a2	-0.06131	0.2888	1
Sh3tc1	-0.06138	0.18728	1
Arl14ep	-0.06139	0.55709	1
Zbtb2	-0.06139	0.262456	1
Adap2os	-0.06141	0.618419	1
Tmem9	-0.06141	0.741725	1
Golga1	-0.06141	0.97001	1
Tm9sf4	-0.06153	0.623519	1
Mks1	-0.06163	0.463202	1
Sigmar1	-0.06177	0.137815	1
Nudt18	-0.06184	0.075059	1
Scarb1	-0.0619	0.347389	1
Klhl6	-0.06191	0.823543	1
B430010I2	-0.06195	0.53245	1
Strbp	-0.06197	0.875939	1
Ccdc127	-0.06198	0.633799	1
Mrps33	-0.06206	0.809568	1
Elac1	-0.06209	0.215949	1
Ago3	-0.06212	0.392242	1
Kctd13	-0.06214	0.475197	1
Nfatc3	-0.06214	0.273388	1
Dhx15	-0.06215	0.620432	1
Tmem65	-0.06218	0.801055	1
Yme1l1	-0.06221	0.590067	1
Blvra	-0.06224	0.253387	1
Pibf1	-0.06225	0.627533	1
Tcf3	-0.06225	0.813279	1
Stambp	-0.06227	0.838421	1
Rbmxl1	-0.0623	0.524211	1
BC031181	-0.06235	0.845496	1
Sbno1	-0.06256	0.79919	1
Ifnar2	-0.06263	0.417268	1
Ccdc62	-0.06267	0.591355	1
Klhl22	-0.06269	0.036769	1
Pcid2	-0.0627	0.634294	1
Cbll1	-0.0627	0.898553	1
Ciao1	-0.06271	0.34014	1
Fndc3b	-0.06275	0.016683	1
Hyal2	-0.06279	0.244527	1
Hnrnpc	-0.06279	0.802992	1
Slc4a2	-0.06279	0.592669	1
Luc7l3	-0.06282	0.267733	1

Utp11	-0.06289	0.70252	1
Polr2e	-0.06291	0.846761	1
Tnfrsf11a	-0.06293	0.223978	1
Lancl1	-0.06294	0.981421	1
Adssl1	-0.06296	0.687398	1
Smcr8	-0.06304	0.475097	1
Gga1	-0.06304	0.903074	1
Ccdc85b	-0.06305	0.306456	1
Cdkl3	-0.06311	0.915629	1
Dad1	-0.06313	0.618358	1
Chmp5	-0.06317	0.484524	1
Flii	-0.06331	0.424384	1
Sf3a2	-0.06336	0.581242	1
Pdik1l	-0.06337	0.497318	1
Gdi2	-0.06343	0.033815	1
Cog6	-0.06348	0.022451	1
1110059G1	-0.06357	0.50765	1
Lypla1	-0.06361	0.191965	1
Asb8	-0.06364	0.544479	1
Cops7b	-0.06366	0.045318	1
Letm1	-0.0637	0.91906	1
Emc1	-0.0637	0.916923	1
Nelfa	-0.06373	0.88619	1
Rap2a	-0.06378	0.375909	1
Prxl2b	-0.06381	0.900916	1
Abcb1b	-0.06386	0.279919	1
Ubqln1	-0.06391	0.460439	1
Rab9	-0.06397	0.740737	1
Slc16a10	-0.06408	0.693327	1
Rnf141	-0.06409	0.922211	1
Sord	-0.0641	0.286796	1
Tbc1d9	-0.06413	0.59329	1
Chrac1	-0.06418	0.252125	1
Fam193b	-0.06421	0.022927	1
Rpap2	-0.06427	0.201094	1
Ptpn4	-0.06431	0.565073	1
Med31	-0.06435	0.079243	1
Rab11a	-0.06438	0.84686	1
Pbx3	-0.06439	0.683573	1
Tmem258	-0.06442	0.477759	1
Rprd1a	-0.06452	0.994601	1
Drg1	-0.06453	0.325636	1
Ctps2	-0.06453	0.283949	1
Mtpn	-0.06455	0.439935	1
2610507I0	-0.06458	0.236936	1
Mgme1	-0.06459	0.174721	1
Fut11	-0.06459	0.728582	1
Mau2	-0.06461	0.943288	1

Sacm1l	-0.06464	0.983499	1
Tecr	-0.06467	0.299084	1
Ankrd46	-0.0647	0.072616	1
Rnf113a2	-0.06471	0.140373	1
Dab2	-0.06471	0.647049	1
Trappc11	-0.06472	0.819778	1
Exosc7	-0.06483	0.859574	1
Vps37c	-0.06484	0.931456	1
Sirt7	-0.06484	0.802497	1
Clic1	-0.0649	0.024321	1
Trmt11	-0.065	0.948112	1
Ostf1	-0.06506	0.394186	1
Mapk8	-0.06509	0.970665	1
Rbm18	-0.06512	0.80188	1
Eloa	-0.06517	0.809119	1
Atp2a2	-0.06519	0.568394	1
Rest	-0.06525	0.765305	1
Trmt13	-0.06533	0.481404	1
Psip1	-0.06536	0.117088	1
Gon4l	-0.06539	0.937709	1
Sdhaf4	-0.06546	0.318172	1
Vps13b	-0.06548	0.550245	1
Dennd4b	-0.06551	0.709247	1
Rab11fip2	-0.06552	0.676673	1
Dnlz	-0.06555	0.24152	1
Zbtb9	-0.06556	0.381546	1
Dynlrb1	-0.06558	0.615274	1
Tlr6	-0.0656	0.836817	1
Fkbp2	-0.06566	0.875649	1
Egln2	-0.06567	0.287469	1
Arl15	-0.06568	0.029354	1
Lats2	-0.06572	0.174107	1
Phf3	-0.06584	0.971489	1
Edc4	-0.06585	0.139572	1
Mrps11	-0.06593	0.406513	1
E4f1	-0.06598	0.993305	1
Usp28	-0.06599	0.361676	1
Rbbp6	-0.066	0.342436	1
Efcab14	-0.06603	0.566485	1
Vps8	-0.06607	0.649565	1
Slc35c1	-0.06607	0.337859	1
Pld2	-0.06615	0.150445	1
Dmac1	-0.06621	0.680412	1
Dgcr2	-0.06625	0.536544	1
Commd10	-0.06626	0.987427	1
Zfp994	-0.0663	0.821727	1
Xrcc4	-0.06631	0.541829	1
Trmt2a	-0.06634	0.295883	1

Hagh	-0.06637	0.288735	1
Afg3l1	-0.0665	0.627661	1
Ssna1	-0.06653	0.559832	1
AU041133	-0.0666	0.229519	1
Aga	-0.06667	0.907088	1
Gtf2b	-0.0667	0.753035	1
BC002059	-0.06671	0.096566	1
Nup188	-0.06672	0.728596	1
Rock1	-0.06675	0.964655	1
Vgll4	-0.06685	0.241959	1
Derl2	-0.06692	0.704193	1
Rbm6	-0.06692	0.955816	1
Tada1	-0.06699	0.711869	1
Smarcb1	-0.067	0.982957	1
Sclt1	-0.06707	0.784157	1
Dcaf5	-0.06714	0.628618	1
Tm2d1	-0.06716	0.771785	1
Slc35f5	-0.06717	0.414603	1
Dcp1a	-0.0672	0.786171	1
Ercc5	-0.06721	0.828861	1
Actn4	-0.06722	0.226578	1
Ppp2ca	-0.06728	0.757789	1
Zscan22	-0.0673	0.085631	1
Cpeb4	-0.06741	0.565645	1
Cramp1l	-0.06742	0.950218	1
Ppa2	-0.0675	0.304714	1
Il21r	-0.06762	0.344711	1
Gimap1	-0.06764	0.009856	1
Ninl	-0.06767	0.734362	1
Nfkbil1	-0.06768	0.246357	1
Pofut1	-0.06771	0.752791	1
Srd5a3	-0.06777	0.74216	1
Gps1	-0.06788	0.244238	1
C330007P(	-0.06794	0.447881	1
Smad4	-0.06794	0.762093	1
Tmem170b	-0.06801	0.773353	1
Eogt	-0.06807	0.020948	1
Rab10os	-0.06811	0.408043	1
Wdr7	-0.06822	0.88364	1
Blvrb	-0.06822	0.177152	1
Actr6	-0.06832	0.097502	1
Oser1	-0.06838	0.314982	1
Ap3m1	-0.06848	0.988581	1
Naa80	-0.0685	0.231778	1
Ngly1	-0.06859	0.924476	1
Scap	-0.06861	0.389369	1
Larp4	-0.06863	0.846042	1
Fgd4	-0.06864	0.966504	1

Ccdc59	-0.0687	0.736372	1
Mesd	-0.0687	0.776439	1
Edrf1	-0.06872	0.609042	1
Ctcf	-0.06879	0.858972	1
Ccpg1	-0.06882	0.591554	1
Capza2	-0.06885	0.052711	1
Gpalpp1	-0.06885	0.178897	1
Bloc1s1	-0.06887	0.731394	1
Usp35	-0.0689	0.07623	1
Smarcd2	-0.06892	0.001749	1
Snapc5	-0.06894	0.358717	1
Rft1	-0.06895	0.924074	1
Cdkn2aipnl	-0.06898	0.729446	1
Hadha	-0.06899	0.448511	1
Ccndbp1	-0.06905	0.247745	1
B3galt4	-0.06912	0.609316	1
Slc9a1	-0.06913	0.209803	1
Tspan3	-0.06913	0.933221	1
Atxn3	-0.06917	0.170691	1
Psmd3	-0.06919	0.496748	1
Dus3l	-0.06919	0.2252	1
Pom121	-0.06923	0.269357	1
Fam193a	-0.06928	0.809106	1
Sec16a	-0.06929	0.043111	1
Msi2	-0.0694	0.591428	1
Tm9sf3	-0.0694	0.702146	1
Tsnax	-0.06943	0.546414	1
Prpf18	-0.06945	0.051052	1
Mapk1ip1	-0.06948	0.11539	1
Med28	-0.0695	0.307839	1
Uros	-0.06951	0.39177	1
Kdm5a	-0.06953	0.376619	1
Cebpz	-0.06954	0.831137	1
2610037D(	-0.06956	0.27372	1
Aasdhppt	-0.06961	0.875805	1
Ptcd3	-0.06965	0.872717	1
Sap25	-0.06967	0.727822	1
2210408I2	-0.06969	0.561274	1
Rbbp7	-0.0697	0.138744	1
Dusp22	-0.0697	0.417241	1
H2-Ob	-0.06971	0.6012	1
Aktip	-0.06974	0.124262	1
Mfsd14a	-0.06976	0.500228	1
Rbm41	-0.06982	0.303096	1
Coasy	-0.06991	0.549988	1
Rab35	-0.06992	0.461918	1
Cnot7	-0.07	0.616206	1
Mbd6	-0.07005	0.021313	1

Ice1	-0.07008	0.819128	1
Spast	-0.0701	0.652424	1
Ccdc191	-0.0701	0.528367	1
Tmco3	-0.07014	0.471885	1
Tbccd1	-0.07014	0.294361	1
Pcbd2	-0.07015	0.170254	1
Prmt9	-0.07017	0.154772	1
Cfap20	-0.0702	0.02158	1
Clcn3	-0.0702	0.231712	1
Polr3b	-0.07025	0.69279	1
Yipf5	-0.07029	0.806984	1
AI987944	-0.0703	0.564504	1
Dync2h1	-0.07065	0.717839	1
Pygo2	-0.07082	0.113634	1
Dync1li2	-0.07087	0.06139	1
Pih1d1	-0.0709	0.617685	1
Mcrip1	-0.07095	0.442674	1
Glmp	-0.07096	0.521389	1
Gimap6	-0.07097	2.18E-05	0.704928
Akt1s1	-0.07098	0.252178	1
Eif4ebp2	-0.07103	0.167608	1
Phf7	-0.07108	0.0395	1
Atpaf1	-0.07121	0.909203	1
Virma	-0.07124	0.687036	1
Zfp143	-0.07128	0.821841	1
Znrf1	-0.07132	0.124553	1
Lgmn	-0.07135	5.59E-05	1
Fopnl	-0.07139	0.563026	1
Mttp	-0.07142	0.040727	1
Pten	-0.07144	0.266237	1
Map4k5	-0.07153	0.271848	1
Sar1a	-0.07153	0.117032	1
Abcf3	-0.07154	0.35658	1
Lyrm2	-0.07156	0.140977	1
Fam120c	-0.07156	0.33074	1
Gtf2a1	-0.07165	0.943337	1
Zfp386	-0.07177	0.695322	1
Mtmr12	-0.07177	0.059794	1
Pip4k2c	-0.07181	0.051756	1
Dmac2l	-0.07186	0.008896	1
Oat	-0.07193	0.958493	1
Gigyf1	-0.07193	0.202664	1
Baz2b	-0.07194	0.297951	1
Arfip1	-0.072	0.092149	1
Trappc2	-0.07202	0.022557	1
Alg12	-0.07206	0.758134	1
Mmut	-0.07208	0.962793	1
Prdx4	-0.07208	0.83657	1

Ercc1	-0.07211	0.032656	1
Rpn2	-0.07216	0.017438	1
Fbxo42	-0.07218	0.087723	1
Cdyl	-0.07222	0.903076	1
Prkd3	-0.07227	0.957841	1
Pkd2	-0.0723	0.381629	1
Fads1	-0.07233	0.288821	1
Stip1	-0.07235	0.963493	1
BC029722	-0.0724	0.180451	1
Sel1l	-0.07241	0.329874	1
Kdm6a	-0.07247	0.605789	1
Pdap1	-0.07247	0.357033	1
Rnf135	-0.0725	0.805349	1
Fmn1	-0.07251	0.410636	1
Sec22b	-0.07256	0.528413	1
Slc10a3	-0.07262	0.222323	1
Scaf1	-0.07275	0.343084	1
Hibadh	-0.07287	0.505465	1
Syvn1	-0.07289	0.935034	1
Abtb1	-0.07299	0.008634	1
Mef2d	-0.07304	0.796052	1
Krba1	-0.07308	0.040305	1
Zfp846	-0.0731	0.102848	1
Lmo2	-0.07318	0.834422	1
Rnf6	-0.0732	0.848063	1
Spsb3	-0.07327	0.693124	1
Gm47664	-0.07328	0.145125	1
Tcta	-0.07328	0.539988	1
Mettl23	-0.07329	0.107311	1
Mtf2	-0.07332	0.147255	1
Grb2	-0.07336	0.018107	1
Cog7	-0.07336	0.354769	1
Mto1	-0.07349	0.626342	1
Ctbs	-0.07363	0.663137	1
Inpp5k	-0.07364	0.643113	1
Vamp3	-0.07367	0.363069	1
Hbs1l	-0.07368	0.477829	1
Cers2	-0.07369	0.212851	1
Sirt2	-0.07372	0.210122	1
Hacd4	-0.0739	0.260022	1
Zfp84	-0.07395	0.767621	1
Abcc5	-0.07402	0.386289	1
Supt4a	-0.07403	0.249448	1
Stoml2	-0.07405	0.795612	1
Zfp24	-0.07408	0.11811	1
Fbxl20	-0.0742	0.516442	1
Mrps12	-0.07421	0.857373	1
Neurl3	-0.07434	0.458081	1

Scaf11	-0.07435	0.153953	1
Coq8b	-0.07438	0.41395	1
Hmbox1	-0.07451	0.651932	1
Pcca	-0.07453	0.777569	1
Wdr33	-0.07455	0.09238	1
Crebzf	-0.07457	0.191949	1
Enoph1	-0.07459	0.176404	1
Syt11	-0.0746	0.853988	1
Psmc1	-0.07461	0.70807	1
Ugp2	-0.07463	0.185947	1
Lrrc14	-0.07466	0.285423	1
Zbtb24	-0.07468	0.232846	1
Trim24	-0.07468	0.429901	1
Vamp1	-0.07476	0.083653	1
Dexi	-0.07484	0.522767	1
Iqce	-0.07487	0.892794	1
Pdgfa	-0.07493	0.343345	1
Strn	-0.07497	0.19656	1
Ric8b	-0.07503	0.282917	1
Ltbr	-0.07507	0.156148	1
Odr4	-0.07508	0.835914	1
Lmo4	-0.07521	0.252013	1
Nek1	-0.07523	0.291547	1
Ssb	-0.0753	0.84784	1
1500004A1	-0.07534	0.712603	1
Mterf3	-0.07537	0.105284	1
Vash1	-0.07543	0.059372	1
Gm5165	-0.07546	0.011384	1
BC024978	-0.07552	0.046586	1
Ulk3	-0.07553	0.226405	1
Cnot11	-0.07555	0.091764	1
Zmat5	-0.07556	0.334602	1
Pacs1	-0.07559	0.520653	1
Acp6	-0.07561	0.104698	1
Glt8d1	-0.07562	0.061099	1
Rbm22	-0.07567	0.446349	1
Gatc	-0.07568	0.056081	1
Hprt	-0.07579	0.344238	1
Ciao3	-0.07584	0.658197	1
Ccdc61	-0.07585	0.098448	1
Hax1	-0.07586	0.723236	1
Snrnp70	-0.07592	0.019624	1
Bcl10	-0.07596	0.686616	1
N4bp2l1	-0.07596	0.314937	1
Plcd1	-0.07603	0.456437	1
Tnfrsf1a	-0.07607	0.163143	1
Borcs7	-0.07608	0.686232	1
Slc48a1	-0.07619	0.310032	1

Nrf1	-0.07621	0.260501	1
Pnrc2	-0.07624	0.151489	1
Fam204a	-0.07624	0.152569	1
Timm50	-0.07625	0.678212	1
Zfp719	-0.0763	0.085869	1
Rab1b	-0.0763	0.278774	1
Lats1	-0.07633	0.271709	1
Gps2	-0.07634	0.189048	1
Rrm2b	-0.07636	0.111887	1
Pqbp1	-0.07639	0.022948	1
Zfp704	-0.0764	0.892242	1
Anapc1	-0.07651	0.192676	1
Ubr1	-0.07654	0.559271	1
Atp6v0a2	-0.07659	0.164424	1
Fibp	-0.07665	0.953454	1
Ccdc130	-0.07668	0.356948	1
Tmem50b	-0.07677	0.740464	1
Iqcb1	-0.07678	0.00133	1
Agpat1	-0.07678	0.080366	1
Gripap1	-0.0768	0.732541	1
Emsy	-0.07687	0.326141	1
Fhl3	-0.07688	0.002625	1
2610001JC	-0.07692	0.804916	1
Cwc15	-0.07695	0.766957	1
Creld1	-0.07702	0.06943	1
Far1	-0.07703	0.55497	1
Zfp317	-0.07706	0.105491	1
Etfdh	-0.07709	0.161725	1
Ube4b	-0.07711	0.865813	1
Dock2	-0.07712	3.1E-06	0.100069
Dnajb12	-0.07712	0.334417	1
Rsf1	-0.07729	0.684495	1
Kdsr	-0.07729	0.193078	1
Elmsan1	-0.0773	0.677583	1
Khsrp	-0.07733	0.287711	1
Ppard	-0.07737	0.784732	1
Erbin	-0.07738	0.871289	1
Cep95	-0.07742	0.880732	1
Msl3	-0.07744	0.094308	1
Alg14	-0.07751	0.926323	1
Xlr	-0.07753	0.5188	1
Gm49359	-0.07756	0.183736	1
Slc38a7	-0.07757	0.765449	1
Armc7	-0.07762	0.401978	1
Prpf6	-0.07762	0.127645	1
Setd1b	-0.0777	0.444431	1
Taok2	-0.07791	0.899	1
Stx6	-0.07793	0.880785	1

Smg6	-0.07795	0.216017	1
Ufsp2	-0.07802	0.698368	1
Zscan26	-0.07804	0.973804	1
Pbxip1	-0.07805	0.886859	1
Gpn1	-0.07811	0.618831	1
Ipmk	-0.07811	0.971396	1
As3mt	-0.07814	0.344597	1
5031425E2	-0.0783	0.225416	1
Mapk8ip3	-0.07833	0.995706	1
Pgm2	-0.07836	0.132727	1
Abcd1	-0.07837	0.074041	1
Derl1	-0.07843	0.425082	1
Vps41	-0.07844	0.831654	1
Fam219b	-0.07847	0.06582	1
Slc33a1	-0.07847	0.265964	1
Krtcap2	-0.07848	0.743435	1
Nae1	-0.0785	0.556354	1
Ruvbl1	-0.07852	0.246521	1
Pcnp	-0.07854	0.761357	1
Trappc12	-0.0786	0.117906	1
Slu7	-0.07864	0.947746	1
Ncor2	-0.07867	0.378085	1
Micu1	-0.07868	0.317077	1
Entr1	-0.07871	0.795841	1
Supt7l	-0.07873	0.090752	1
Setdb1	-0.07877	0.501292	1
Retreg3	-0.07882	0.126797	1
Phf20	-0.07886	0.959666	1
Ypel2	-0.07892	0.011279	1
Cmtm4	-0.07893	0.57815	1
Ash1l	-0.07898	0.938557	1
Klhdc3	-0.07901	0.283089	1
Brd7	-0.07905	0.988686	1
Ubap2l	-0.07912	0.480411	1
E230029C0	-0.07916	0.385032	1
Scarb2	-0.07919	0.121786	1
Eif2b4	-0.0792	0.417817	1
Pstk	-0.07921	0.118161	1
Prkdc	-0.07924	0.962671	1
Casp6	-0.07924	0.193324	1
Cdca4	-0.07928	0.101927	1
Retsat	-0.07936	0.233938	1
Zfp644	-0.07939	0.807735	1
Tardbp	-0.0795	0.771339	1
Smu1	-0.07956	0.088539	1
Cdc73	-0.07956	0.788437	1
3110056K0	-0.07963	0.048721	1
Nup88	-0.07967	0.044076	1

Plpp5	-0.07968	0.016687	1
Prdx3	-0.07972	0.724452	1
Pigt	-0.07973	0.684879	1
Ap3d1	-0.07974	0.485375	1
Npepl1	-0.07977	0.866474	1
Ints14	-0.07984	0.149111	1
Cux1	-0.07985	0.592675	1
Apip	-0.07992	0.609207	1
Atg4c	-0.07992	0.600892	1
4930453N	-0.07994	0.132496	1
Znrf2	-0.07995	0.803438	1
Zc3h3	-0.07999	0.503802	1
C2cd3	-0.08011	0.730447	1
Gaa	-0.08011	0.735194	1
Rab18	-0.08015	0.40174	1
Nova1	-0.08018	0.132654	1
Trim23	-0.08018	0.065236	1
Nlrp3	-0.08018	0.491313	1
Hps1	-0.08021	0.135173	1
Zfp414	-0.08023	0.368082	1
Zfp865	-0.08023	0.135168	1
Kdelr2	-0.08026	0.426158	1
Ublcp1	-0.08034	0.186403	1
Bzw1	-0.08035	0.034313	1
Ofd1	-0.08041	0.571956	1
Pabpn1	-0.08041	0.692833	1
Sec23a	-0.08051	0.034903	1
Acvr2a	-0.08055	0.222939	1
Ddx20	-0.08055	0.151829	1
Pot1a	-0.08058	0.131088	1
Reep3	-0.08061	0.635795	1
Gtpbp2	-0.08062	0.794865	1
Spg11	-0.08063	0.952867	1
Pam16	-0.08069	0.827236	1
Tmem134	-0.08072	0.616535	1
Ptdss2	-0.08076	0.202208	1
Ppp1r35	-0.08086	0.101161	1
Fyttd1	-0.08088	0.212145	1
Rbsn	-0.0809	0.179749	1
Mpnd	-0.08091	0.457418	1
Smarcc2	-0.08098	0.448055	1
Arrdc3	-0.08098	0.131453	1
Bmt2	-0.08103	0.892772	1
Fam168b	-0.08104	0.442668	1
Mettl5	-0.08104	0.013713	1
Commd2	-0.08106	0.019148	1
Lefty1	-0.08108	0.023824	1
Dcun1d2	-0.08109	0.13402	1

Fam135a	-0.08112	0.134669	1
Rnf11	-0.08119	0.681305	1
Nup62	-0.08128	0.958251	1
Tmem256	-0.08133	0.807296	1
Aplp2	-0.08164	0.817784	1
Prpf40b	-0.08165	0.289007	1
Fancl	-0.08167	0.17059	1
Tmem63b	-0.0817	0.24954	1
Tm2d3	-0.08175	0.373868	1
Gpr107	-0.08176	0.899967	1
Zfp35	-0.08177	0.061573	1
Ebp	-0.08189	0.352303	1
Sap130	-0.08189	0.814307	1
Kdm4c	-0.0819	0.947076	1
Trappc13	-0.0819	0.008045	1
Scd2	-0.0819	0.013254	1
Zfp703	-0.08194	2.51E-05	0.809722
Nsrp1	-0.08196	0.565659	1
Gpcpd1	-0.08199	0.022239	1
Tspan9	-0.08203	0.523845	1
Marf1	-0.08205	0.461515	1
Dcps	-0.08209	0.092	1
Wac	-0.08217	0.885257	1
Prrc2b	-0.08218	0.143292	1
Dhx34	-0.08223	0.211226	1
Cul9	-0.08238	0.260165	1
Smarcc1	-0.08248	0.600996	1
Tmem203	-0.08249	0.005166	1
Rad23b	-0.08257	0.628051	1
Srp54a	-0.08258	0.002921	1
Mpp5	-0.08271	0.010289	1
Dhrs4	-0.08276	0.43167	1
Ap2b1	-0.0828	0.531685	1
Gm20703	-0.08282	0.014052	1
Smg9	-0.0829	0.390231	1
Tnks2	-0.08293	0.866569	1
Tjp2	-0.08303	0.176581	1
Akap7	-0.08311	0.884604	1
Gm49797	-0.08322	0.163872	1
Rab40c	-0.08324	0.318611	1
Zdhhc17	-0.08331	0.287242	1
Sesn2	-0.08343	0.160955	1
Arhgef11	-0.08344	0.555735	1
Ogt	-0.08362	0.020866	1
Tug1	-0.08364	0.111515	1
Pde6d	-0.08376	0.103577	1
Vps18	-0.08377	0.254974	1
Zmpste24	-0.08379	0.999055	1

Anapc4	-0.0838	0.383858	1
Ganab	-0.08389	0.518991	1
Il7r	-0.0839	0.050811	1
Srp19	-0.08393	0.32573	1
Dr1	-0.08399	0.360417	1
Brwd1	-0.08399	0.455196	1
Cxxc1	-0.084	0.01006	1
Ssu72	-0.08403	0.316392	1
Rnaseh2c	-0.08406	0.146723	1
Med23	-0.08406	0.695977	1
Cgrrf1	-0.08414	0.306604	1
Pogz	-0.08427	0.333042	1
Ctdsp1	-0.08429	0.017806	1
Snrnp200	-0.08432	0.411208	1
Srprb	-0.08432	0.148267	1
Elp3	-0.08435	0.038237	1
Gm6712	-0.08437	0.120103	1
Mrpl48	-0.08437	0.211919	1
Bloc1s6	-0.0846	0.127814	1
Elmo2	-0.08461	0.060251	1
Xab2	-0.08462	0.417027	1
Mcm3	-0.08466	0.452954	1
Ankrd10	-0.08469	0.039797	1
Aldh6a1	-0.08473	0.032142	1
Zfp68	-0.08483	0.037269	1
Gm10138	-0.08489	0.082026	1
Dcun1d1	-0.08491	0.267036	1
Prcc	-0.08493	0.167289	1
Myo7a	-0.085	0.992025	1
Disp1	-0.08506	0.012404	1
Nabp2	-0.08511	0.23126	1
1190007I0	-0.08513	0.082673	1
Msh2	-0.08515	0.371625	1
Fam50a	-0.08515	0.181911	1
Gm39469	-0.0852	0.090891	1
Nudcd3	-0.08524	0.427667	1
Capn3	-0.08536	0.155937	1
Zfp955b	-0.08548	0.005452	1
Cpsf2	-0.08549	0.377636	1
Senp3	-0.08556	0.004259	1
Kat7	-0.08557	0.225095	1
Jmjd1c	-0.08565	0.286786	1
Shoc2	-0.08572	0.273023	1
Zmym6	-0.08577	0.032317	1
D1Ertd622	-0.08592	0.276964	1
Sulf2	-0.08595	0.87205	1
Ccdc47	-0.08604	0.628085	1
Prr12	-0.08617	0.021897	1

Dapp1	-0.08617	0.117032	1
Tgfa	-0.0862	0.166569	1
Nit1	-0.08623	0.091146	1
Hectd4	-0.08628	0.813568	1
Smndc1	-0.08629	0.427873	1
Vps26c	-0.08636	0.225869	1
Faap100	-0.08638	0.081333	1
Ubr3	-0.08646	0.640302	1
Tmem106b	-0.08647	0.002364	1
Ipo13	-0.08652	0.002663	1
Mbtps1	-0.08652	0.187417	1
Txlna	-0.08661	0.909726	1
Wipf2	-0.08666	0.177182	1
Cdc23	-0.08671	0.484146	1
Zfp629	-0.08672	0.002936	1
Senp6	-0.08686	0.057292	1
Hmgn1	-0.0869	0.233396	1
Zfp217	-0.08694	0.652247	1
Pex5	-0.08697	0.308981	1
Alyref2	-0.08702	0.106334	1
Ubtf	-0.08704	0.036589	1
Fem1a	-0.08705	0.009596	1
Cdk10	-0.08707	0.299428	1
Kbtbd3	-0.08711	0.044139	1
Ipo8	-0.08724	0.891373	1
Bsg	-0.08724	0.027497	1
Dbndd2	-0.0873	0.755991	1
Renbp	-0.08732	0.645505	1
Ssbp1	-0.08737	0.259404	1
Rb1cc1	-0.0874	0.267884	1
Sh3gl1	-0.08743	0.160983	1
Atg12	-0.08747	0.257872	1
Hnrnpf	-0.08753	0.150495	1
Lrig2	-0.08756	0.326712	1
Klhl25	-0.08757	0.014131	1
Itgb3bp	-0.08758	0.018278	1
Pggt1b	-0.08768	0.307145	1
Phf23	-0.0878	0.111628	1
Stx4a	-0.08782	0.601112	1
Tbc1d22a	-0.08789	0.387182	1
Pogk	-0.08792	0.058853	1
Map11	-0.08803	0.024481	1
Slc25a51	-0.08809	0.034125	1
Arpc5l	-0.08809	0.912702	1
Ggps1	-0.0881	0.348557	1
Tnpo2	-0.08814	0.288621	1
Lpcat2	-0.08816	0.000831	1
Ddhd2	-0.08817	0.436018	1

Maea	-0.08819	0.537673	1
Hdhd2	-0.0882	0.087996	1
Dnmbp	-0.08821	0.108836	1
Eapp	-0.08828	0.402289	1
Stard5	-0.08832	0.030792	1
Taf12	-0.08837	0.396981	1
Slc25a17	-0.08839	0.303653	1
Gsdme	-0.08842	0.020318	1
Zfp729b	-0.08848	0.975912	1
Kansl2	-0.08852	0.094152	1
AU040320	-0.08853	0.001815	1
Fut8	-0.0886	0.050486	1
Npl	-0.08863	0.09573	1
Zfp384	-0.0887	0.001266	1
Trim41	-0.08877	0.60986	1
Top3b	-0.08879	0.306437	1
Pigyl	-0.0888	0.074718	1
Tex264	-0.08882	0.186135	1
Snx19	-0.08883	0.892745	1
Slc38a10	-0.08888	0.08073	1
Gm37233	-0.08902	0.028279	1
Gtf2e2	-0.08909	0.026047	1
1700028E1	-0.08912	0.092167	1
Rrnad1	-0.08919	0.762731	1
Pygb	-0.08924	0.019651	1
Chmp6	-0.08924	0.118178	1
Ginm1	-0.08935	0.226557	1
Rmnd5a	-0.08936	0.083848	1
Ate1	-0.08941	0.228022	1
Clk3	-0.08958	0.606416	1
Gtf3c2	-0.08959	0.02514	1
Pskh1	-0.08976	0.549128	1
Il17ra	-0.08979	0.082725	1
Alg9	-0.08983	0.307275	1
Ap1s2	-0.08984	0.047776	1
Napa	-0.0899	0.130158	1
Fam129a	-0.08996	0.136964	1
Akirin2	-0.09001	0.054753	1
2210016F1	-0.09004	0.395378	1
Ap2a1	-0.09009	0.600231	1
Sh2d3c	-0.09013	0.016761	1
Arcn1	-0.09022	0.217339	1
Faf2	-0.09022	0.301407	1
Mtif3	-0.09027	0.009613	1
Hk2	-0.09034	0.855285	1
Ro60	-0.09039	0.023756	1
Acox1	-0.09047	0.25241	1
Galnt2	-0.09049	0.065402	1

Ylpm1	-0.09054	0.650867	1
Mrpl43	-0.09055	0.088713	1
Clock	-0.09064	0.896359	1
Hk1	-0.09067	0.000234	1
Angel2	-0.09077	0.881164	1
Por	-0.0908	0.106435	1
Sf3a1	-0.0908	0.558983	1
Mmd	-0.09081	0.429049	1
Kpna6	-0.09085	0.117298	1
Rpl7l1	-0.09091	0.339602	1
Appl1	-0.09091	0.032621	1
Pip4p2	-0.09096	0.114019	1
Myh9	-0.09097	0.003208	1
Rcn2	-0.09097	0.163345	1
Rcc2	-0.09112	0.407184	1
Kiz	-0.09113	0.092001	1
Tnni2	-0.09122	0.002121	1
Slc45a4	-0.09123	0.193455	1
Fbxo22	-0.0913	0.58932	1
Alg2	-0.09135	0.200475	1
Ndufs2	-0.09136	0.252612	1
Pigu	-0.09138	0.456408	1
Brox	-0.09145	0.144752	1
Arpc1a	-0.09149	0.335079	1
Dennd1a	-0.09155	0.809454	1
Smim1	-0.09161	0.001284	1
Fech	-0.09165	0.264917	1
Unk	-0.09189	0.034063	1
Zfyve21	-0.09193	0.016913	1
Eif4g3	-0.09195	0.127209	1
Rprd1b	-0.09203	0.270398	1
Cradd	-0.09208	0.552726	1
Ptpn9	-0.09217	0.469951	1
Rbm4	-0.09223	0.128087	1
Iars	-0.09225	0.871495	1
F8a	-0.09226	0.002245	1
Tyw1	-0.09228	0.591094	1
Tmc7	-0.0923	0.002238	1
Acsl1	-0.09231	0.453122	1
Rbm5	-0.09234	0.228545	1
Zhx3	-0.09234	0.047579	1
Pdcd6ip	-0.09248	0.467036	1
Tubgcp5	-0.09258	0.158833	1
Hnrnpd	-0.09264	0.242058	1
Mfap1b	-0.09265	0.001056	1
Ndfip1	-0.09273	0.022526	1
Fto	-0.0928	0.995495	1
Gm29291	-0.0928	8.83E-05	1

Gm15564	-0.09285	0.141078	1
Trmt12	-0.09286	0.032495	1
Lrrcc1	-0.09296	0.197798	1
Ebpl	-0.09303	0.023995	1
Itprid2	-0.09324	0.504057	1
Gm41764	-0.09327	0.005803	1
Ip6k2	-0.09328	0.034077	1
Camkk2	-0.09329	0.789206	1
Vasp	-0.09333	0.437751	1
Sympk	-0.09333	0.162166	1
Orai2	-0.09339	0.549589	1
Ank2	-0.09344	0.910878	1
Ppp3ca	-0.09351	0.117189	1
Ankra2	-0.09354	0.043171	1
Ctns	-0.09357	0.206754	1
Msl1	-0.0936	0.432002	1
Pdcl3	-0.09363	0.272009	1
Josd2	-0.09368	0.69987	1
Senp8	-0.09373	0.009498	1
Nxf1	-0.09376	0.59381	1
Tmem185b	-0.09388	0.047642	1
Eif2ak1	-0.09393	0.080955	1
Sgsm3	-0.09393	0.006637	1
Selenos	-0.09401	0.080769	1
Clcc1	-0.09402	0.182195	1
Cdc42se2	-0.09405	0.036021	1
Tmem101	-0.09407	0.159289	1
Prrg2	-0.09412	0.004962	1
Smpd2	-0.09412	0.009385	1
Bcl2l11	-0.09415	5.99E-07	0.019335
Ppm1f	-0.09426	0.423326	1
Ganc	-0.0943	0.488151	1
Smpdl3a	-0.0943	0.038757	1
Idh3g	-0.09431	0.106149	1
Tpra1	-0.09446	0.243444	1
Ctdspl2	-0.09459	0.036563	1
Foxo3	-0.09461	0.021695	1
Polr1a	-0.09465	0.684021	1
Ppp2r5a	-0.09467	0.083311	1
Fuz	-0.0947	0.007552	1
Fkbp7	-0.09478	0.015598	1
Poldip2	-0.09483	0.746311	1
Srr	-0.09488	0.093081	1
Cdc5l	-0.09488	0.083769	1
Atp6v1e1	-0.09493	0.172692	1
Ddx18	-0.09499	0.341629	1
Cul3	-0.095	0.35059	1
Hace1	-0.0951	0.039431	1

Crls1	-0.0952	0.00761	1
Phax	-0.0953	0.602179	1
Sap18	-0.09531	0.094006	1
Ppp4r1	-0.09536	0.459592	1
Elp4	-0.09541	0.464046	1
Bud31	-0.09552	0.457193	1
Abraxas2	-0.09561	0.134909	1
Zfp263	-0.09574	0.250542	1
Ndufaf2	-0.0958	0.017624	1
Zcchc2	-0.0958	0.018847	1
Kif16b	-0.09585	0.107343	1
Ppp6r2	-0.09593	0.033395	1
Shprh	-0.09596	0.106091	1
Atxn2	-0.09599	0.588249	1
Mob3a	-0.096	0.25199	1
Tnrc6c	-0.09608	0.930052	1
Sowahc	-0.0961	0.032951	1
Vps37a	-0.09612	0.107682	1
Otulin	-0.09617	0.739828	1
Ilvbl	-0.0962	0.700214	1
Mpc2	-0.09624	0.339438	1
Cdk2ap1	-0.09631	0.002791	1
Tlr13	-0.09632	0.136199	1
Stam	-0.09634	0.097057	1
Hint3	-0.09642	0.236802	1
Pdss2	-0.0965	0.396409	1
Myo1f	-0.09655	0.042408	1
Map4k3	-0.09656	0.391692	1
Nudt16	-0.09657	0.174312	1
Dolk	-0.0966	0.004457	1
Leng8	-0.09664	0.380149	1
Psmc2	-0.09672	0.25947	1
Zfp672	-0.09685	0.128979	1
Zfp933	-0.09685	0.019515	1
Zbed3	-0.09685	0.176955	1
Rbm27	-0.09692	0.326271	1
Pkp4	-0.09694	0.73752	1
Suclg1	-0.09694	0.165794	1
Tfip11	-0.09701	0.006692	1
Epc2	-0.09709	0.105833	1
Zfp120	-0.09711	0.186951	1
Nrbp1	-0.09712	0.851239	1
Il31ra	-0.09718	0.000846	1
Ticam2	-0.09721	0.010155	1
Pigq	-0.09724	0.080002	1
Mocs2	-0.09727	0.257699	1
Clptm1l	-0.0973	0.485198	1
Gins4	-0.09739	0.001765	1

Vamp4	-0.09742	0.160911	1
Tmco6	-0.09742	0.003751	1
Lztr1	-0.09746	0.121133	1
Ndrg3	-0.09747	0.028064	1
Slc37a4	-0.09756	0.149869	1
Dync1h1	-0.0976	0.372437	1
Stk11	-0.09762	0.395821	1
Polr2j	-0.09765	0.378063	1
Cstf2t	-0.09769	2.11E-05	0.682096
Smc3	-0.0977	0.081941	1
Dgat1	-0.09775	0.019957	1
Romo1	-0.09786	0.581167	1
Cript	-0.09828	0.165689	1
Fam214a	-0.09836	0.089458	1
Kctd21	-0.09841	0.000483	1
Pik3r2	-0.09843	0.046679	1
Pigh	-0.0985	0.002067	1
Apeh	-0.09852	0.542041	1
Zkscan1	-0.09856	0.319308	1
Csnk1d	-0.09859	0.208344	1
Aarsd1	-0.09867	0.071012	1
Fubp3	-0.09878	0.119224	1
Sdf2	-0.09881	0.250346	1
Rab3gap2	-0.0989	0.702905	1
Ncoa2	-0.09892	0.077642	1
Lysmd3	-0.09899	0.029488	1
Ehbp1	-0.09908	0.226967	1
Ppwd1	-0.09914	0.265694	1
Rbm43	-0.09916	0.005513	1
Gopc	-0.09917	0.199782	1
Gm15726	-0.0992	0.02259	1
Ciz1	-0.0992	0.017002	1
Nelfb	-0.09924	0.016184	1
Hvcn1	-0.09933	0.017922	1
Tti1	-0.09941	0.482217	1
Casp3	-0.09942	0.069729	1
Cul5	-0.09942	0.921286	1
Hikeshi	-0.09946	0.036392	1
Stat5b	-0.09953	0.011409	1
Nf2	-0.09956	0.474114	1
Hmgcs1	-0.09958	0.196234	1
Cog1	-0.09959	0.107281	1
Atg101	-0.09963	0.000823	1
Pcmtd1	-0.09967	0.4396	1
Eloc	-0.09979	0.193992	1
Tfg	-0.0998	0.868517	1
Vkorc1	-0.09981	0.279622	1
Nudt16l1	-0.09982	0.001163	1

Cdc42bpa	-0.09985	0.598521	1
Qdpr	-0.09991	0.18435	1
Uqcc3	-0.10011	0.039733	1
Dpy30	-0.10015	0.411989	1
Ccnd1	-0.10019	0.464249	1
Zfp740	-0.10029	0.006101	1
Crnkl1	-0.10033	0.144753	1
Gatb	-0.10039	0.663275	1
Rap2c	-0.10044	0.238226	1
Oaf	-0.10054	0.549584	1
Anapc11	-0.10057	0.102653	1
Zfp212	-0.1006	0.009672	1
Cdkn2aip	-0.10061	0.036751	1
Iqsec2	-0.10069	0.682909	1
Hnrnpu	-0.1007	0.017404	1
Orai3	-0.10075	0.036428	1
Errfi1	-0.10101	0.006358	1
Ing1	-0.10108	0.017851	1
Ssr1	-0.10113	0.244775	1
Smpd5	-0.10126	0.029839	1
Slc39a1	-0.1013	0.328922	1
Ppp4r2	-0.10135	0.675445	1
Rc3h2	-0.10138	0.464682	1
Nsd1	-0.10141	0.101617	1
Med19	-0.10148	0.013639	1
Gm15537	-0.10152	0.004698	1
Polr2b	-0.10168	0.160985	1
Snx3	-0.1018	0.106007	1
Dnajc7	-0.10192	0.373123	1
Zmym3	-0.10194	0.007583	1
Ptgs1	-0.10201	1.25E-05	0.404035
Mrpl36	-0.10205	0.048439	1
Agap1	-0.10221	0.17646	1
Hspa12a	-0.10225	0.112078	1
Bola1	-0.10229	0.197415	1
Appl2	-0.10237	0.028968	1
Ktn1	-0.10243	0.35405	1
Ankrd13c	-0.1025	0.129247	1
Filip1l	-0.10253	0.086868	1
Ubr2	-0.10261	0.130267	1
Rab33b	-0.10262	0.001088	1
Amz2	-0.10266	0.107777	1
Api5	-0.10267	0.053726	1
Syap1	-0.10278	0.148664	1
Nagpa	-0.1029	0.452172	1
Ring1	-0.10297	0.0988	1
Vezt	-0.10297	0.106307	1
Prkra	-0.10298	0.074166	1

March7	-0.10302	0.022855	1
Ube2r2	-0.10305	0.779996	1
Med22	-0.10307	0.083325	1
Arf2	-0.10308	0.073926	1
Cacna1d	-0.1031	0.135114	1
Cyb5r1	-0.10311	0.319957	1
Smc6	-0.10312	0.263262	1
Spsb2	-0.1032	0.150853	1
Mbd1	-0.10323	0.137733	1
Phospho2	-0.10325	0.00198	1
E2f3	-0.10325	0.550448	1
Fadd	-0.10327	0.003883	1
Ost4	-0.10335	0.361443	1
Otub1	-0.10336	0.687317	1
1600012H(	-0.10342	0.328078	1
Map1lc3a	-0.10351	0.003822	1
Rps6ka4	-0.10352	0.160367	1
Slx1b	-0.10357	0.007229	1
Wdr26	-0.10368	0.105382	1
Snrpa	-0.10371	0.158017	1
Nol4l	-0.10372	0.711946	1
Dapk3	-0.10376	0.144947	1
Smarcd1	-0.10378	0.025387	1
Psmd11	-0.10382	0.055647	1
Xpo5	-0.10383	0.047919	1
Sucla2	-0.10389	0.137911	1
Nisch	-0.1039	0.420278	1
Txndc12	-0.10391	0.039724	1
Chmp2b	-0.10392	0.0044	1
Bcas2	-0.10401	0.149737	1
Dhrs7b	-0.10411	0.680224	1
Eefsec	-0.10416	0.424939	1
Zmym5	-0.10417	0.236435	1
Tfeb	-0.10422	0.58176	1
Dync1li1	-0.10428	0.850168	1
Slc2a9	-0.10431	0.737374	1
Alkbh5	-0.10434	0.05627	1
Yipf4	-0.10435	0.389975	1
Vamp2	-0.10441	0.034906	1
Smpdl3b	-0.10468	0.000282	1
Snx4	-0.10471	0.053661	1
Tmem159	-0.10473	0.031836	1
Polr2k	-0.10474	0.289953	1
Lpar5	-0.10483	0.013962	1
Ints4	-0.10487	0.068637	1
Ppp1r9b	-0.1049	0.085238	1
Map3k14	-0.10492	0.259949	1
Ppp6c	-0.10495	0.137114	1

Spag7	-0.10497	0.02412	1
Platr25	-0.10508	0.053924	1
Brip1os	-0.10508	0.164214	1
Sec23b	-0.10511	0.089243	1
Vps26a	-0.1052	0.126029	1
Cstf3	-0.10524	0.273122	1
Smim8	-0.10529	0.001485	1
2610528A1	-0.10532	0.18734	1
Tcf7l2	-0.10537	0.608695	1
Dlgap4	-0.1054	0.859529	1
Galnt10	-0.10541	0.870801	1
Bap1	-0.10547	0.041685	1
Zc3h4	-0.10548	0.056532	1
Brd9	-0.10551	0.128851	1
Wbp4	-0.10552	0.590634	1
Zfp617	-0.10553	0.004649	1
Lzic	-0.10553	0.008859	1
Stard3nl	-0.10561	0.099708	1
Bcas3	-0.10562	0.568881	1
Atn1	-0.1057	0.000745	1
Celf2	-0.10573	0.000205	1
Kat8	-0.1058	0.012158	1
Layn	-0.10584	0.008168	1
Gak	-0.10585	0.7853	1
Dusp3	-0.10589	0.043539	1
Midn	-0.10611	0.100053	1
Ccdc22	-0.10616	0.023687	1
Pdxdc1	-0.10618	0.704909	1
Gatd3a	-0.1062	0.039202	1
Zc3h6	-0.10626	0.009104	1
Slc7a7	-0.10635	0.384322	1
Dzip3	-0.10641	0.013225	1
Tbc1d12	-0.10645	0.013349	1
Map3k5	-0.10646	0.04924	1
Yars	-0.10646	0.072368	1
Dcp1b	-0.10648	0.036651	1
Btf3l4	-0.10652	0.047979	1
Prdm2	-0.10659	0.312959	1
Ppp3r1	-0.10662	0.016524	1
Kansl1	-0.10662	0.387508	1
Spire1	-0.1067	0.564946	1
Usf1	-0.10687	0.000849	1
2010013B2	-0.10691	0.002571	1
Lman2l	-0.10693	0.442446	1
Pomt1	-0.10697	0.072853	1
Strip1	-0.10699	0.401518	1
Nelfcd	-0.10703	0.001819	1
Pecam1	-0.10704	4.44E-08	0.001435

Usp11	-0.10709	0.00039	1
Fbxl14	-0.1071	0.022251	1
Wdsub1	-0.10713	0.000406	1
Zbtb37	-0.10715	0.014654	1
Dnpep	-0.10731	0.053228	1
Bphl	-0.10741	0.000785	1
Spns1	-0.10775	0.23053	1
Pgpep1	-0.10786	0.00386	1
B4galt3	-0.10792	0.021151	1
Tollip	-0.10794	0.000501	1
Pdk1	-0.10799	0.16835	1
Ick	-0.10801	0.000461	1
Bsdc1	-0.10806	0.007598	1
A530072M	-0.1081	0.169847	1
Tmed4	-0.10812	0.052842	1
Oga	-0.10813	0.124846	1
Pigc	-0.10814	0.064062	1
Idua	-0.10817	0.217563	1
Nxt2	-0.10821	0.029663	1
Mrpl34	-0.10832	0.284392	1
Cand1	-0.10835	0.03968	1
Cnot6l	-0.10842	0.026478	1
Srrm1	-0.10842	0.024608	1
Trappc4	-0.10844	0.008333	1
Ncf2	-0.10847	0.074932	1
Aaas	-0.10868	0.178559	1
Ubtd1	-0.10869	0.40725	1
Man2a2	-0.10873	0.01979	1
Agpat3	-0.1089	0.221406	1
Tmem115	-0.10898	0.033872	1
Ppp2r3a	-0.10899	0.002481	1
Tbck	-0.10903	0.612312	1
Zbtb18	-0.10909	0.039423	1
Zfp277	-0.1091	0.33148	1
Zfp422	-0.10917	0.003544	1
Rraga	-0.10933	0.487153	1
Med13	-0.10946	0.308985	1
Zfp866	-0.10951	0.002262	1
Impa1	-0.10953	0.030027	1
Hibch	-0.10961	0.004223	1
Tm7sf3	-0.10968	0.034925	1
Wdfy4	-0.10973	0.420743	1
Ikzf1	-0.10979	0.000477	1
Gnpda2	-0.10988	0.035598	1
Fig4	-0.10988	0.13773	1
Maml2	-0.10991	0.445726	1
Sec24c	-0.10993	0.028485	1
Fam118b	-0.11002	0.009344	1

Gatad2b	-0.11005	0.752825	1
Zfp236	-0.11006	0.475314	1
Mast2	-0.11015	0.402508	1
Nckap5l	-0.11016	0.160563	1
Ppp1r11	-0.11019	0.352538	1
Dstyk	-0.11021	0.051076	1
Dhx57	-0.11025	0.04784	1
Ago1	-0.11035	0.288923	1
Kctd20	-0.1104	0.024523	1
Dpf2	-0.1105	0.232649	1
Dcakd	-0.11056	0.561142	1
Plxna4os1	-0.11059	0.034156	1
Apbb2	-0.11064	0.354326	1
Sufu	-0.11069	0.275769	1
Dalrd3	-0.11075	0.000496	1
D030028A(	-0.11081	0.144256	1
Elp2	-0.11087	0.246337	1
Anapc7	-0.11088	0.039324	1
Dleu2	-0.11092	0.000402	1
Camk2g	-0.11118	0.552373	1
Qser1	-0.11127	0.158463	1
Bhlhe41	-0.11135	0.496919	1
Gm15635	-0.11141	0.013421	1
Bag5	-0.11145	0.067363	1
1110051M	-0.1115	0.6021	1
Tmem230	-0.11152	0.018801	1
Oxsm	-0.11153	0.037182	1
Copb1	-0.11154	0.015554	1
Fbxl3	-0.11162	0.063675	1
Wdr48	-0.11165	0.012457	1
Rybp	-0.11169	0.001328	1
Ldb1	-0.11169	0.04141	1
Cbr1	-0.1117	0.055379	1
Lactb	-0.11173	0.025128	1
Lmf1	-0.11189	0.152666	1
0610010K1	-0.11193	0.045718	1
Ctnnal1	-0.11207	0.016423	1
Trappc9	-0.11207	0.44091	1
Cep85l	-0.11208	0.271456	1
Cyhr1	-0.11218	0.020158	1
Prkag1	-0.1122	0.121863	1
Poldip3	-0.11235	0.02563	1
Caml	-0.11243	0.084547	1
Ormdl1	-0.11244	0.005028	1
Pkn1	-0.11245	0.492056	1
Cmtm3	-0.11246	0.345988	1
Apobr	-0.11251	0.021409	1
Dnajc24	-0.11255	0.124301	1

Zfx	-0.11256	0.110454	1
Spata13	-0.11259	0.115859	1
Jkamp	-0.11271	0.06702	1
Crkl	-0.11274	0.095277	1
Hypk	-0.11279	0.064386	1
Cdip1	-0.11285	0.073626	1
Acadm	-0.11287	0.030019	1
Smim7	-0.11292	0.630079	1
Vbp1	-0.11306	0.000638	1
Man1b1	-0.11312	0.007783	1
Narf	-0.11312	0.005215	1
Nat9	-0.11318	0.078721	1
Gnpat	-0.1132	0.165882	1
Armcx3	-0.11324	0.050724	1
Med17	-0.11327	0.225784	1
Ppp2r5d	-0.11329	0.0005	1
Tmed5	-0.11337	0.009999	1
Itfg1	-0.11339	0.049456	1
Gramd1a	-0.1134	0.024201	1
Nfkb1	-0.11345	0.032337	1
Coro1b	-0.11348	0.01498	1
Tcp11l2	-0.11371	1.03E-08	0.000334
Oxld1	-0.11376	0.004162	1
Ift20	-0.11387	0.049223	1
Cdipt	-0.11391	0.023442	1
Srrm2	-0.11396	0.022319	1
Rbm10	-0.114	0.000329	1
Lonp2	-0.11401	0.043839	1
Gclc	-0.11402	0.001098	1
Cebpzos	-0.11406	0.229123	1
2510009E(	-0.11406	0.213966	1
Ext1	-0.11407	0.627701	1
Yaf2	-0.11409	0.324659	1
Rprd2	-0.11412	0.031125	1
Usp10	-0.11412	0.03962	1
Lpin1	-0.11413	0.002015	1
Eif4h	-0.11413	0.01106	1
St6galnac4	-0.11417	0.063556	1
Tmed9	-0.11421	0.001209	1
Rubcnl	-0.11429	0.074211	1
Blcap	-0.1144	0.097631	1
Acot13	-0.11445	0.245301	1
Zfp266	-0.11446	0.090586	1
Vma21	-0.11451	0.019276	1
Supt5	-0.11455	0.085253	1
Rhoc	-0.11458	0.01214	1
Slc39a13	-0.11458	0.061554	1
Vps35l	-0.11461	0.137523	1

Ppp1r7	-0.11464	0.037909	1
Vps39	-0.11466	0.000324	1
Ahcyl2	-0.1147	0.116146	1
Aip	-0.11471	0.343582	1
Ccdc71	-0.11478	0.003604	1
Nosip	-0.11485	0.022047	1
Pxmp4	-0.11494	0.003495	1
Ube3c	-0.11497	0.426788	1
5330438D1	-0.11501	0.009505	1
Trmt1l	-0.11503	0.005343	1
Arid3b	-0.11505	0.342172	1
P2rx7	-0.11515	0.01504	1
Snap47	-0.11527	0.009264	1
Rbl2	-0.11533	0.000145	1
Dnajb6	-0.11544	0.040149	1
Gnpda1	-0.11563	0.059466	1
Vps26b	-0.11564	0.002644	1
Pias3	-0.11575	0.004698	1
Rassf2	-0.11579	0.005322	1
Ppox	-0.11581	0.043006	1
Smyd4	-0.11586	0.14547	1
Atp6ap1	-0.11587	0.016064	1
Dnase2a	-0.11592	0.016562	1
Cnot3	-0.11594	0.028997	1
Atp6v0d1	-0.11594	0.135962	1
Isca1	-0.11599	0.050179	1
Dis3l	-0.11599	0.135286	1
Card9	-0.11611	0.010778	1
Dock9	-0.11611	0.001672	1
Sema4c	-0.11618	0.477213	1
Armh3	-0.11624	0.1718	1
Gpat4	-0.11627	0.086177	1
Mcur1	-0.11637	0.675772	1
Arhgef12	-0.11644	0.693049	1
Pik3ca	-0.1168	0.210235	1
Fam160b1	-0.11684	0.007771	1
Zfp658	-0.11686	0.126193	1
Zcchc14	-0.11686	0.001344	1
Rrp1b	-0.11687	0.129715	1
Pyroxd2	-0.11692	0.00015	1
Taco1	-0.11692	0.530159	1
Cog8	-0.11705	0.002996	1
Metap2	-0.11708	0.017451	1
Pycr2	-0.11708	0.152239	1
Fam107b	-0.11712	2.07E-09	6.67E-05
Glod4	-0.1172	0.046779	1
Camta2	-0.11722	0.43965	1
Adrm1	-0.11723	0.225991	1

Hs1bp3	-0.11733	0.061165	1
Cops2	-0.11734	0.192837	1
Vezf1	-0.11735	0.004284	1
Ubap1	-0.11735	0.08186	1
G6pc3	-0.11765	0.016671	1
Asxl1	-0.11768	0.102665	1
Vwa5a	-0.11769	0.123511	1
Vps11	-0.11776	0.060892	1
Lrrc28	-0.11785	0.003922	1
Rhog	-0.11787	0.031867	1
Gpsm1	-0.11791	3.35E-06	0.108257
Prr14	-0.11792	0.018657	1
Usf2	-0.11795	0.171668	1
Tgfbr2	-0.11801	0.008984	1
Ube2z	-0.11808	0.132751	1
Naalad2	-0.11808	0.092889	1
Mlph	-0.11811	0.000208	1
Lrp5	-0.11812	0.29403	1
Adck2	-0.11816	0.004551	1
Atpif1	-0.11822	0.034076	1
Tet3	-0.11825	0.176181	1
Fam120b	-0.11829	0.017259	1
Btbd9	-0.11837	0.460837	1
Kmt2e	-0.11838	0.067533	1
Trp53bp1	-0.11853	0.231369	1
Cd82	-0.11857	0.012657	1
Tgm2	-0.1186	0.018902	1
Ireb2	-0.11877	0.343361	1
Commd6	-0.11879	0.31232	1
Vta1	-0.11885	0.064457	1
Mettl7a1	-0.1189	0.375508	1
Fbxw5	-0.1189	0.139862	1
Miga2	-0.11891	0.001075	1
Sf3b4	-0.11892	0.056183	1
B4galt6	-0.11908	0.057596	1
Samm50	-0.11908	0.063231	1
Tusc3	-0.11913	0.256825	1
Eif4a3	-0.1192	0.008236	1
R3hdm4	-0.11924	0.112161	1
Atg4b	-0.11928	0.27252	1
Cklf	-0.11931	0.040048	1
Ccdc107	-0.11946	0.141427	1
Tbc1d23	-0.1195	0.503487	1
5033421B(	-0.11954	0.020049	1
Sun2	-0.11966	0.634692	1
Rbfa	-0.11967	0.173212	1
Oip5os1	-0.11985	0.122812	1
Ccdc122	-0.11986	0.241799	1

Fbxo8	-0.11986	0.000269	1
Bicra	-0.1199	0.384865	1
Arid1b	-0.11994	0.014599	1
Tmed3	-0.11999	0.050186	1
Ppp2r3d	-0.12002	0.001241	1
Xrcc5	-0.12003	9.07E-06	0.292755
Babam2	-0.12004	0.132988	1
Kmt5c	-0.12011	9.05E-05	1
Gtdc1	-0.12032	0.492197	1
Rhoq	-0.12051	0.388564	1
Ppp1cb	-0.12054	0.015585	1
Pik3r4	-0.12057	0.002069	1
Memo1	-0.12065	0.517076	1
Spcs2	-0.1207	0.003002	1
Zfp637	-0.12071	0.001954	1
Actr2	-0.12076	0.00355	1
Card19	-0.12076	0.019441	1
Btd	-0.12082	0.000434	1
Dock10	-0.121	4.59E-10	1.48E-05
Kbtbd2	-0.12109	0.000509	1
Smim14	-0.1211	0.056457	1
Gm15441	-0.12113	1.02E-05	0.329815
Borcs6	-0.12125	0.001371	1
Lgals8	-0.12131	0.065184	1
Zbtb22	-0.12134	2.17E-06	0.070067
Zfp787	-0.12134	0.21305	1
Pip4k2b	-0.12135	0.265101	1
Srbd1	-0.12139	0.075625	1
Spred1	-0.12147	0.00107	1
Cyp27a1	-0.12162	0.017669	1
Rgl3	-0.12183	9.48E-05	1
Dennd6a	-0.12184	0.028146	1
Glo1	-0.12189	0.002039	1
Atp11c	-0.12197	0.102242	1
Pcyox1l	-0.122	0.000367	1
Prrc1	-0.122	0.085144	1
Rnf150	-0.12208	0.069462	1
Tsc22d2	-0.12208	0.258828	1
Fgf13	-0.12217	0.539261	1
Ntpcr	-0.12217	0.000387	1
Umad1	-0.1222	0.110866	1
Usp4	-0.12223	0.230252	1
Ppcdc	-0.1223	0.009685	1
Tppp	-0.12247	9.95E-06	0.321137
Nfix	-0.12251	0.51745	1
Lman1	-0.12254	0.012752	1
Cdk19	-0.12255	0.177304	1
Gpatch2l	-0.1226	0.19818	1

Ebi3	-0.12266	0.388155	1
Qrich1	-0.12274	0.00855	1
Mtr	-0.12283	0.007805	1
Fkbp4	-0.12284	0.023985	1
Cplane1	-0.1229	0.325632	1
Pecr	-0.12307	0.097561	1
Chmp1a	-0.1231	0.044261	1
Tubgcp2	-0.12316	0.000493	1
Cfap97	-0.12319	0.000415	1
Nop2	-0.12322	0.103025	1
Rbbp9	-0.12332	1.69E-06	0.054723
Wbp11	-0.12352	0.024524	1
Hyou1	-0.12354	0.056092	1
Scamp3	-0.12354	0.011217	1
Jun	-0.12357	2.67E-05	0.863293
Cpne1	-0.12358	0.246071	1
Btrc	-0.12361	0.045592	1
Slc29a3	-0.12362	0.00874	1
Insig2	-0.12366	0.011194	1
Cldnd1	-0.12372	0.158047	1
Uba5	-0.12394	0.021239	1
Shld2	-0.12394	0.005563	1
Sept10	-0.12396	0.017928	1
Cfdp1	-0.12406	0.634107	1
Ap1g1	-0.12411	0.205815	1
Pik3c3	-0.12416	0.013325	1
Ggcx	-0.12416	0.000855	1
Slco4a1	-0.12418	1.32E-05	0.425543
0610009B2	-0.1243	0.005377	1
Sphk2	-0.12434	0.038992	1
Mgat4a	-0.12438	0.041869	1
Tmx2	-0.1244	0.040975	1
Impad1	-0.12449	0.113753	1
Blmh	-0.1246	0.081887	1
Bmf	-0.12462	0.001578	1
Vps4a	-0.12472	0.03757	1
Cdca7	-0.12488	0.000285	1
Cd200r1	-0.12494	0.000752	1
Mapre1	-0.12499	0.035904	1
Trpm4	-0.12514	0.0453	1
Vps72	-0.12516	0.002497	1
Tbc1d31	-0.12522	0.051967	1
Gspt1	-0.12532	0.013683	1
Coa3	-0.12533	0.133208	1
Fbxl17	-0.12536	0.23431	1
Dhrs7	-0.12538	0.019241	1
Usp9x	-0.12544	0.136796	1
Xpot	-0.12548	0.000144	1

Zfp113	-0.12559	0.001514	1
D3Ertd751	-0.12574	8.75E-06	0.282439
Gnai3	-0.12577	0.018289	1
Akr7a5	-0.1258	0.023057	1
Mmaa	-0.12586	0.000194	1
Surf1	-0.12597	0.022355	1
Gm33858	-0.12602	0.003366	1
Pcf11	-0.12614	0.326181	1
Cracr2b	-0.12617	0.000363	1
Dcaf8	-0.12618	0.012952	1
Stau1	-0.12619	0.238945	1
Ikbip	-0.12628	0.00428	1
Wwp1	-0.12632	0.020552	1
Naa10	-0.12632	0.021349	1
Tfcp2	-0.12641	0.012279	1
AW146154	-0.12654	0.000309	1
Tssc4	-0.12658	0.0025	1
Trim5	-0.12678	0.001275	1
Xiap	-0.12681	0.004531	1
Naxe	-0.12687	0.035905	1
Gm15523	-0.12693	0.000487	1
Pak1	-0.12705	0.225589	1
Pdcl	-0.12712	0.000141	1
March6	-0.12714	0.010171	1
Cdk13	-0.12716	0.02667	1
Tmx3	-0.12733	0.009727	1
Tob2	-0.12751	0.016208	1
Wbp1	-0.12756	0.016259	1
Tmx4	-0.12765	8.98E-07	0.028988
Map4k2	-0.12772	0.001947	1
Tmem245	-0.12778	0.022783	1
Topors	-0.1279	0.0567	1
B3galnt1	-0.12794	0.326766	1
Tmem198b	-0.12798	0.000246	1
Banp	-0.12802	0.022208	1
Gne	-0.12802	0.000161	1
Ndufc2	-0.12803	0.006689	1
Wdpcp	-0.12805	0.872901	1
Brd8	-0.12808	0.100835	1
Phf21a	-0.12816	0.045248	1
Mars	-0.12819	0.031867	1
Pex6	-0.12832	5.38E-05	1
Gse1	-0.12838	0.186814	1
Tdp1	-0.12838	0.068736	1
Emc10	-0.1284	0.001692	1
Ahdc1	-0.12845	0.014145	1
Zcchc8	-0.12858	0.030006	1
Rhot1	-0.1286	0.082529	1

Cdk8	-0.12874	1.35E-05	0.436492
Tmem120a	-0.12875	0.087594	1
Arsk	-0.12877	0.001798	1
Ccnq	-0.12883	0.00226	1
Pou2f1	-0.12888	0.016617	1
Dse	-0.12893	0.004089	1
Prmt2	-0.12894	0.027849	1
Rabgap1	-0.12901	0.024628	1
Tsn	-0.12909	0.004958	1
Yipf3	-0.12915	0.016003	1
Copa	-0.12944	8.02E-05	1
Uba6	-0.12944	0.003619	1
Arhgdia	-0.12947	0.003919	1
Pias1	-0.12961	0.000258	1
Smim27	-0.12965	0.000356	1
Gm26520	-0.12969	0.019808	1
Ergic3	-0.1297	0.013839	1
Zfp397	-0.12978	0.022682	1
Ap1b1	-0.12979	0.010538	1
Nlk	-0.12987	0.127357	1
Pcbp1	-0.12998	0.044643	1
Exosc10	-0.13	0.141439	1
Bcdin3d	-0.13019	0.003112	1
Yae1d1	-0.13023	0.001754	1
Wdr20	-0.13028	0.273364	1
Ogg1	-0.13028	0.000792	1
Gpatch8	-0.13034	0.861454	1
Slc39a7	-0.13034	0.01468	1
Sgk3	-0.13044	0.151047	1
Rgl1	-0.13046	0.013002	1
Gm38843	-0.13054	0.012517	1
Daglb	-0.13064	0.000261	1
Epha2	-0.13069	0.011931	1
Yes1	-0.1307	0.007937	1
Hspa13	-0.13075	0.026348	1
Bcl2l13	-0.131	0.000445	1
Ctdsp2	-0.131	2.66E-05	0.858724
Clcf1	-0.13102	0.000197	1
Zmynd11	-0.13104	0.019446	1
Zfp157	-0.13117	0.005131	1
Hcls1	-0.13122	0.038266	1
Tnks	-0.13124	0.688229	1
Snx30	-0.13125	0.351041	1
Pag1	-0.13126	1.85E-05	0.597255
Phkb	-0.13135	0.104374	1
Cpsf1	-0.13143	0.021045	1
Brk1	-0.13146	0.0299	1
Glrp1	-0.1315	0.004151	1

Gm29488	-0.13153	0.001524	1
Gm15503	-0.13163	0.003183	1
Gm16023	-0.13164	4.41E-05	1
Ralbp1	-0.13169	0.17081	1
Phf12	-0.1317	0.252344	1
Lrrc39	-0.1319	1.72E-08	0.000556
Aldh4a1	-0.1321	0.000129	1
Tmed7	-0.13213	0.027822	1
Morc2a	-0.13214	0.011201	1
Spsb1	-0.13221	0.001445	1
Rubcn	-0.13222	0.014365	1
Smurf1	-0.13222	0.247673	1
Unc93b1	-0.13236	1.29E-10	4.15E-06
Hmgxb4	-0.13238	0.031759	1
Tpk1	-0.13241	0.035053	1
Ppp4r3a	-0.13242	0.395283	1
Pigk	-0.13258	0.085791	1
Pcif1	-0.13263	0.025456	1
Zbtb7a	-0.13272	0.0131	1
Ccdc97	-0.13272	7.85E-05	1
Nsmce1	-0.13279	0.016218	1
Mplkip	-0.1328	0.017169	1
H2-Ke6	-0.1328	0.003591	1
Brd3	-0.13282	0.000593	1
Abi2	-0.13283	0.000414	1
Arfgap2	-0.13286	0.014737	1
Jpx	-0.13294	0.002147	1
Ube2m	-0.13294	0.026604	1
Madd	-0.13302	0.003387	1
Ddrgk1	-0.13317	0.085049	1
Kif13a	-0.13317	0.239685	1
Scaper	-0.13318	0.146381	1
Vav1	-0.13324	0.007193	1
AI467606	-0.13329	0.01912	1
Map3k20	-0.13333	0.004742	1
Fes	-0.13338	0.049936	1
Ift52	-0.13351	0.098647	1
Ppp2r5c	-0.13352	0.274937	1
Haghl	-0.13365	0.002705	1
Ank	-0.13375	0.001665	1
Luc7l2	-0.13375	0.002864	1
Tada3	-0.13399	0.001517	1
Wdr45b	-0.134	0.01272	1
Fkbp15	-0.13407	0.019483	1
Slain2	-0.13413	0.016367	1
Mdc1	-0.13418	0.001124	1
Epc1	-0.13437	0.000319	1
Scrn3	-0.13438	0.002822	1

Oxct1	-0.13446	0.001421	1
Sfi1	-0.13447	0.007352	1
Dennd1c	-0.1345	0.036878	1
Cdk2ap2	-0.13464	0.018442	1
Foxp1	-0.13466	0.008115	1
Txnl1	-0.13467	0.004213	1
Zkscan3	-0.13469	0.010707	1
Bptf	-0.13471	0.076796	1
Mafg	-0.13471	0.087468	1
Dhx32	-0.13481	0.000932	1
Fam210a	-0.13481	0.003087	1
Tbc1d16	-0.13489	0.022603	1
Calcoco1	-0.1349	7.48E-05	1
Fam168a	-0.13493	0.256677	1
Tbcc	-0.13497	1.86E-05	0.601168
Aldh9a1	-0.13511	0.009057	1
Timm17b	-0.13512	0.0082	1
Sdha	-0.13514	0.023524	1
Stradb	-0.13515	3.49E-07	0.011283
Tial1	-0.13532	0.072638	1
Tle4	-0.13533	0.008999	1
Dusp16	-0.13556	0.076827	1
Mbtps2	-0.13572	0.098722	1
Lrwd1	-0.13573	4.31E-05	1
Dhx8	-0.13577	0.127723	1
Trim8	-0.13585	0.001871	1
Ppp2r1b	-0.13589	0.003008	1
Cd3eap	-0.13591	0.060857	1
Pdlim5	-0.13593	0.010581	1
Kdm4a	-0.13604	0.007224	1
Dtx3	-0.13611	0.05321	1
Tmub2	-0.13613	0.019562	1
Pot1b	-0.13622	0.045063	1
Arap1	-0.13626	0.110404	1
D6Wsu163	-0.13645	0.016117	1
Nucks1	-0.13648	0.006315	1
Mta2	-0.13652	0.026253	1
Gm41071	-0.13655	0.000228	1
Gtf3c4	-0.13659	1.19E-05	0.384866
Parn	-0.1367	0.018831	1
Ttyh3	-0.1367	0.002006	1
Tnfaip8l2	-0.13678	0.000629	1
Tsc2	-0.1368	0.035208	1
Gm16124	-0.13682	0.00148	1
Ss18	-0.13685	0.000954	1
Csnk2a1	-0.13695	0.024009	1
Ppp2r1a	-0.13696	0.000751	1
Prkcb	-0.13697	0.700786	1

Zfp646	-0.13697	3.29E-05	1
Fgd3	-0.13703	0.243495	1
Sipa1l2	-0.13718	0.52231	1
Cab39	-0.13753	0.012077	1
Pdzd8	-0.13759	0.010932	1
Wwc2	-0.13762	0.134888	1
Dok1	-0.13762	0.041258	1
Ccng1	-0.13762	0.015356	1
Nmt1	-0.13765	0.007651	1
Vps13d	-0.138	0.827197	1
D630023F1	-0.13803	9.37E-05	1
Cdc123	-0.13804	0.003099	1
Ddb1	-0.1381	0.000839	1
Msl2	-0.1381	0.009143	1
Ywhaq	-0.1382	0.002134	1
Ier5	-0.13844	8.12E-06	0.262161
Appbp2	-0.13844	0.026875	1
Akt1	-0.13873	0.006705	1
Anapc13	-0.13875	0.09276	1
Rab5b	-0.13886	0.0026	1
Mpv17	-0.13897	0.000497	1
Osgep	-0.13898	0.017679	1
Sema4g	-0.1391	6.19E-08	0.001998
Lpxn	-0.13912	0.000775	1
Mcrs1	-0.1392	0.004391	1
Cib2	-0.13921	1.72E-06	0.055445
Aldh3a2	-0.13924	3.42E-05	1
Tmx1	-0.13935	0.000314	1
Slc35b2	-0.13947	0.001233	1
Insr	-0.13948	0.092153	1
Gorasp2	-0.13969	0.012754	1
Mon2	-0.13976	0.0214	1
Slc50a1	-0.13981	0.026366	1
Pabpc4	-0.13985	0.00318	1
Ilk	-0.14008	0.001477	1
Gpx4	-0.14012	0.016313	1
Zbtb1	-0.14012	0.022522	1
Lsm10	-0.14032	0.034107	1
Pcnx3	-0.14044	1.2E-05	0.387024
St3gal2	-0.14045	0.002711	1
Ech1	-0.14057	0.002277	1
Champ1	-0.14057	0.024763	1
Mpp7	-0.1407	0.003207	1
Zfp426	-0.14071	0.018895	1
5031439G0	-0.14071	0.000674	1
Mgat1	-0.14098	0.010494	1
Smim15	-0.14114	0.000653	1
Zfp90	-0.14119	0.003364	1

Dcaf17	-0.14124	0.035312	1
Sh3bp1	-0.14153	0.156541	1
Rb1	-0.14157	0.408844	1
Vps4b	-0.14161	0.005574	1
Isoc2b	-0.14174	4.49E-05	1
Sec31a	-0.14179	0.005444	1
Actr1a	-0.1418	0.00312	1
Zkscan8	-0.14182	0.002906	1
Commd3	-0.14219	0.015607	1
Gm16867	-0.14237	5.34E-07	0.017241
Plcb3	-0.14239	0.00029	1
Erh	-0.14251	0.007937	1
D130009I1	-0.14262	3.29E-08	0.001062
Ier3ip1	-0.14264	0.026415	1
Bckdk	-0.14265	0.003436	1
Ptpa	-0.14265	0.013419	1
Zkscan17	-0.14269	0.006206	1
Srek1ip1	-0.14272	0.128989	1
Thap11	-0.14277	9.1E-05	1
Ndfip2	-0.14282	0.001492	1
Mob4	-0.14304	0.009797	1
Actr8	-0.14356	0.000835	1
Mfsd11	-0.14358	6.78E-05	1
Sec13	-0.14359	0.016696	1
Nbeal2	-0.14376	2.42E-09	7.8E-05
Atf6b	-0.14376	0.032073	1
Ngrn	-0.14381	1.32E-05	0.426572
Uba2	-0.14399	0.008697	1
Arhgap32	-0.14401	0.000699	1
Zfp110	-0.14405	0.000165	1
Parp1	-0.14413	0.007333	1
Calcrl	-0.1442	9.44E-05	1
Puf60	-0.14432	0.001994	1
Plau	-0.14434	0.000202	1
Agbl3	-0.14437	1.37E-07	0.004416
Ankrd44	-0.14442	3.78E-10	1.22E-05
Rnf44	-0.14474	0.002422	1
Csk	-0.14476	0.018102	1
Stxbp3	-0.14477	0.046142	1
Snapin	-0.14488	0.003192	1
Nucb1	-0.14489	0.033423	1
AW549877	-0.14496	0.012501	1
Capzb	-0.14502	4.34E-05	1
Khdrbs1	-0.14514	0.003079	1
Tlr9	-0.14537	0.149034	1
Ubxn7	-0.14541	0.007908	1
Rin3	-0.14544	0.001617	1
1110019D	-0.1456	6.47E-05	1

Chmp3	-0.14575	0.007106	1
Cyp4f13	-0.14595	0.017828	1
St13	-0.14615	0.01862	1
Gm15489	-0.1462	3.16E-05	1
Hnrnpr	-0.14621	0.00279	1
Ralgapb	-0.14622	0.005667	1
Mthfs	-0.14625	0.02523	1
Chm	-0.14625	0.008071	1
Zfp518a	-0.14626	0.001445	1
Plekhm3	-0.14629	0.013125	1
Chd6	-0.1463	0.136531	1
Golga2	-0.14631	0.003067	1
Lima1	-0.14632	0.001685	1
Arglu1	-0.14632	0.285057	1
Secisbp2l	-0.14645	0.021735	1
Dstn	-0.14678	0.000432	1
Kdm5b	-0.14678	1.76E-05	0.568283
Sfswap	-0.14693	0.569884	1
Brat1	-0.14704	0.00322	1
Tax1bp3	-0.14724	0.001305	1
Pnisr	-0.14732	0.15268	1
Sp2	-0.14739	0.0002	1
Tns3	-0.14758	0.054788	1
Itgav	-0.14763	0.000232	1
Lrrc61	-0.1477	2.41E-07	0.007787
Rsbn1	-0.1477	0.005978	1
Rasal2	-0.14773	0.138779	1
Mtfr1l	-0.1479	0.001099	1
2310009A(	-0.14792	0.003797	1
Timm21	-0.14795	1.28E-05	0.413129
Oard1	-0.14803	0.000139	1
Dipk1a	-0.14804	2.83E-07	0.009124
5830432E(	-0.14808	3.28E-05	1
Nbeal1	-0.14811	0.002281	1
Srp68	-0.14816	0.016276	1
Pgrmc1	-0.14817	1.25E-05	0.40246
Atf7ip	-0.1485	0.000949	1
Hacd3	-0.14856	0.002571	1
Rab3gap1	-0.14862	0.013711	1
Thap3	-0.14862	0.001143	1
Unc50	-0.14888	0.00094	1
Rai1	-0.14894	0.363351	1
Ano10	-0.14896	0.154291	1
Gbf1	-0.14897	0.155409	1
Map2k6	-0.14898	0.000811	1
Was	-0.14904	0.002521	1
Lamtor1	-0.14914	0.000996	1
Paqr7	-0.14935	0.023639	1

Naxd	-0.14941	0.001818	1
5830444B(	-0.14943	2.54E-06	0.082075
H3f3b	-0.14945	3.3E-07	0.010663
Cyb5d2	-0.1496	7.45E-06	0.240675
Gmfg	-0.14985	0.201336	1
Amd1	-0.15011	2.97E-06	0.095951
Enox2	-0.15016	0.314254	1
Zfp579	-0.15034	4.24E-05	1
Pxk	-0.15041	0.003692	1
Adnp	-0.15048	0.223931	1
2610008E1	-0.15052	5.83E-06	0.188359
Sptssa	-0.15068	0.00097	1
Capns1	-0.15074	0.007642	1
4930481A1	-0.15089	2.42E-08	0.000781
Polr2a	-0.15095	0.001852	1
Ube4a	-0.15097	0.075252	1
Engase	-0.15099	4.27E-07	0.013777
Usp19	-0.15102	0.005657	1
Laptm4b	-0.15121	0.010424	1
Degs1	-0.15127	0.001652	1
Trf	-0.15143	2.03E-07	0.006555
Stard9	-0.15155	0.024112	1
Exoc7	-0.15162	0.012046	1
Snx5	-0.15169	2.99E-06	0.096669
Hist1h2bc	-0.15193	0.005621	1
Zmym2	-0.15199	0.026399	1
Pja1	-0.152	8.52E-06	0.275135
Cd79b	-0.15228	0.003769	1
Otud7b	-0.15234	0.081099	1
Immp1l	-0.15242	0.091049	1
Ccdc106	-0.15253	1.35E-05	0.436735
Mmgt2	-0.15254	5.51E-05	1
Cybc1	-0.15266	0.001568	1
Abi1	-0.15272	0.00439	1
Dyrk1a	-0.1528	0.067517	1
Gpsm3	-0.15289	0.000497	1
Abcc1	-0.15298	0.001325	1
Zfp606	-0.15306	8.83E-07	0.028519
Mpc1	-0.15317	0.000493	1
Ncbp3	-0.15318	0.003013	1
Gmeb1	-0.15339	0.015405	1
Naa60	-0.15341	0.00666	1
Ccni	-0.15343	0.001856	1
Rad23a	-0.15345	0.002024	1
Dctn1	-0.15346	2.54E-05	0.819084
4930517O1	-0.15353	0.001691	1
Nek7	-0.15353	0.008751	1
Frg1	-0.15365	0.004845	1

Ssbp2	-0.15368	0.153384	1
Cdkn1b	-0.15371	0.000176	1
A630001G:	-0.15371	0.013291	1
Padi2	-0.15377	7.02E-05	1
Nsfl1c	-0.15378	0.033194	1
Ampd3	-0.15402	5.07E-06	0.163843
Bcl7a	-0.15407	1.52E-06	0.049203
2010315B(	-0.15419	0.001887	1
Cic	-0.15432	0.000301	1
Tmem35b	-0.15434	3.84E-05	1
Top2b	-0.15443	0.006857	1
Cc2d1b	-0.15474	0.000189	1
Lrmp	-0.15504	0.00278	1
Hbp1	-0.1552	0.000633	1
Rcbtb2	-0.15521	0.000601	1
Map3k1	-0.15525	0.000104	1
Cnnm2	-0.15541	7.56E-08	0.002441
Arsa	-0.15543	0.001943	1
Hjurp	-0.1556	0.002372	1
Lamtor4	-0.15572	0.002789	1
Pigv	-0.15577	2.05E-05	0.662899
AW554918	-0.15607	0.013635	1
Yy1	-0.1561	0.029947	1
Plekha2	-0.15611	0.001617	1
Ccdc192	-0.1562	2.85E-05	0.921383
Mat2b	-0.15622	0.003934	1
Cdc26	-0.15629	0.113407	1
Trim35	-0.15661	0.000314	1
Atg14	-0.15689	1.93E-06	0.062167
Hsd17b4	-0.15696	0.001875	1
Dcaf7	-0.15697	0.024181	1
Cnnm3	-0.15698	2.04E-05	0.657519
Kcnk13	-0.15708	0.001795	1
Ufl1	-0.15722	0.012598	1
Bbip1	-0.15723	0.012128	1
Tmlhe	-0.15727	0.001462	1
Gmpr2	-0.15727	0.020404	1
Pak2	-0.15734	3.29E-06	0.106153
Rad21	-0.15736	7.08E-05	1
Sh3glb1	-0.15736	0.000588	1
Maml1	-0.15749	0.000345	1
Prex1	-0.15762	0.002399	1
Ophn1	-0.15763	0.001328	1
Chmp1b	-0.1577	9.06E-06	0.292477
Edem1	-0.15773	0.002696	1
Fkbp9	-0.15779	1.69E-05	0.545321
Erlin2	-0.15803	0.013037	1
Calhm2	-0.15806	0.001034	1

Bco2	-0.15808	0.002802	1
Trmt112	-0.15829	0.016213	1
Hnrnph2	-0.15842	0.013755	1
Zc3h12b	-0.15862	0.000511	1
Mapkap1	-0.15867	0.001384	1
Nfat5	-0.15877	0.012566	1
2810006K2	-0.15878	0.001159	1
Eml4	-0.15903	0.076118	1
Letm2	-0.15931	4.42E-06	0.142651
Il15	-0.15973	0.000358	1
4931406C0	-0.15986	0.002997	1
Stk3	-0.15995	0.150065	1
Phgdh	-0.16004	0.01499	1
Hist1h4d	-0.16016	9.83E-05	1
Ano6	-0.16019	0.000923	1
Dcxr	-0.16022	0.003549	1
Zfp329	-0.16025	0.000112	1
Gid8	-0.16027	0.000843	1
Xpo7	-0.1603	0.025798	1
Ctnna1	-0.16032	0.002053	1
Slc26a2	-0.16039	5.49E-05	1
Trim65	-0.16062	3.97E-05	1
4732471J0	-0.16071	0.005163	1
Plxdc1	-0.16097	0.069164	1
Lilra5	-0.1611	0.003258	1
Rab5a	-0.16113	0.13399	1
Lhfpl2	-0.16121	0.017659	1
Smim10l1	-0.16127	0.0103	1
Ptgr2	-0.16128	0.000423	1
Mfsd14b	-0.16131	0.00035	1
Vxn	-0.16154	2.72E-08	0.00088
Riok2	-0.16169	0.026107	1
Fundc1	-0.16172	0.000181	1
Smad5	-0.16174	3.04E-06	0.098131
Mecp2	-0.16178	0.043483	1
Slc38a9	-0.16183	0.001764	1
Gm14455	-0.16184	2.09E-11	6.74E-07
Ints10	-0.16203	0.001683	1
Clpx	-0.16213	0.001108	1
Polr3gl	-0.16224	4.54E-06	0.146512
Sort1	-0.16234	0.000346	1
Cpsf3	-0.16247	0.008099	1
H2afv	-0.16253	0.000889	1
Atp6v1c1	-0.16253	0.007444	1
Dctn2	-0.16257	0.00704	1
Bax	-0.16262	0.01093	1
Smo	-0.16263	0.001175	1
Fem1c	-0.1629	2.65E-05	0.854773

Pnkd	-0.16305	0.001006	1
Tmem33	-0.1636	0.02365	1
Dennd5a	-0.16376	0.062639	1
Mff	-0.16377	0.005623	1
Bcl7b	-0.16378	0.007409	1
Fbxl4	-0.16389	0.003765	1
Tln1	-0.16408	0.00818	1
Gmip	-0.16413	0.000777	1
Dguok	-0.16427	0.000819	1
Gm47754	-0.1645	2.6E-05	0.838974
Cebpa	-0.16451	0.000543	1
Rdx	-0.16452	0.002613	1
Tia1	-0.1647	0.00104	1
Dnajc18	-0.16476	0.00089	1
Spcs3	-0.16483	0.000411	1
Cebpd	-0.1651	2.91E-06	0.094014
Agps	-0.16517	0.009169	1
1110004F1	-0.16526	0.006534	1
Chtf8	-0.16565	5.84E-05	1
6330418K(	-0.16573	3.73E-09	0.00012
Tcea1	-0.16584	0.002046	1
Fnip2	-0.16593	0.069505	1
Cox20	-0.16605	0.000313	1
Cox16	-0.16606	0.003713	1
Camsap1	-0.16609	0.000981	1
Ptpn6	-0.16627	7.31E-06	0.235856
Bcl9	-0.16631	0.001829	1
Tbc1d17	-0.16645	9.65E-06	0.311634
Map3k11	-0.16648	0.000229	1
Dock11	-0.16652	0.024007	1
Ccdc88b	-0.16655	0.00029	1
Amfr	-0.16665	0.000113	1
Dazap2	-0.1667	0.001768	1
Fam167b	-0.1668	0.001275	1
Cd300c2	-0.16686	1.77E-06	0.057088
Patz1	-0.16706	0.000354	1
Cdkal1	-0.16708	0.152709	1
Fzd4	-0.16708	2.3E-08	0.000744
Gm37401	-0.16708	1.54E-07	0.004963
Asb3	-0.16714	0.001555	1
Ulk4	-0.16723	0.001119	1
Tle3	-0.16725	6.9E-05	1
Abhd17c	-0.1673	0.000323	1
Ankfy1	-0.16749	0.007323	1
Czib	-0.16755	0.001651	1
Spr	-0.16769	5.58E-05	1
Cnep1r1	-0.16776	8.37E-06	0.270359
Ccl9	-0.16776	0.030923	1

Abhd4	-0.16789	0.000105	1
Matr3	-0.16792	0.000156	1
Tulp4	-0.16802	0.003664	1
Stxbp5	-0.16803	0.010212	1
Jak1	-0.16806	1.29E-07	0.004173
Tgoln1	-0.16808	0.007445	1
Skp1a	-0.16808	1.98E-05	0.640564
Gsk3b	-0.16815	1.17E-05	0.37902
Gm28417	-0.16821	0.000426	1
Letmd1	-0.16822	0.000203	1
Mepce	-0.16824	2.74E-05	0.883814
Cacna1a	-0.16839	0.014938	1
Slf2	-0.16841	0.001962	1
Mtmr3	-0.1685	0.005083	1
Rxylt1	-0.16863	0.000252	1
Asap3	-0.16863	0.001857	1
1700012D1	-0.16875	7.81E-06	0.252004
Cep112	-0.16878	0.083787	1
Vcp	-0.16887	0.003009	1
Zfp148	-0.16898	0.011438	1
Rbm33	-0.16931	0.01192	1
Kmt2c	-0.16941	0.000131	1
B4galt4	-0.1695	0.004019	1
Sfmbt1	-0.16956	0.056079	1
St8sia4	-0.16964	3.33E-10	1.07E-05
Camk2n1	-0.1697	0.001284	1
Tmem60	-0.16978	0.000506	1
Washc4	-0.16991	0.000161	1
Taok1	-0.16993	0.001916	1
Gm13710	-0.16994	0.001096	1
Bnip3l	-0.17024	0.000223	1
Hs6st1	-0.17036	1.17E-05	0.378015
Dido1	-0.17074	0.000245	1
Pon3	-0.17074	0.000759	1
Lmf2	-0.17085	1.63E-05	0.526206
Zfp770	-0.17105	0.006776	1
Cdk5rap3	-0.17109	0.000466	1
Fam76b	-0.17109	0.00424	1
Slc2a8	-0.1712	0.000277	1
Exoc5	-0.17134	0.002095	1
Mrc2	-0.17138	6.76E-05	1
Sf1	-0.17175	1.72E-05	0.555149
Map7d1	-0.17183	0.000129	1
Prorsd1	-0.17195	0.000192	1
Ctr9	-0.17201	0.000664	1
Scp2	-0.17202	0.000228	1
Zbtb38	-0.17207	0.000233	1
Snx27	-0.1722	0.002272	1

Gdi1	-0.1722	2.27E-05	0.732029
Fam45a	-0.17221	8.13E-05	1
Reep5	-0.17228	1.01E-05	0.324644
Rps6ka5	-0.17228	0.005707	1
Espl1	-0.17229	2.38E-05	0.769123
Ube3a	-0.1724	0.001086	1
Maged2	-0.17271	5.08E-08	0.001641
4833439L1	-0.1728	0.002503	1
Nr6a1	-0.17285	0.046079	1
Gpr137	-0.17295	0.000344	1
C87436	-0.173	0.000896	1
Bcl9l	-0.17301	4.2E-06	0.135682
Dyrk2	-0.17323	1.05E-05	0.337457
Cflar	-0.17333	0.001627	1
Ccny	-0.1734	0.017314	1
Atat1	-0.1735	2.44E-06	0.078838
Magi3	-0.17374	8.14E-05	1
Ugt1a7c	-0.1738	0.000264	1
Lbr	-0.17412	4.32E-08	0.001394
H3f3a	-0.17413	3.38E-05	1
Ly96	-0.17421	0.001191	1
Zfp260	-0.17439	1.7E-05	0.549629
2810403D:	-0.17458	0.00127	1
Mxi1	-0.1746	0.000731	1
Gabpb1	-0.17499	0.000199	1
Ptafr	-0.17518	2.73E-05	0.882637
Pan3	-0.1752	0.056906	1
Clip1	-0.17532	0.006357	1
Upp2	-0.17536	0.008489	1
Pik3c2a	-0.17545	0.001285	1
Scn1b	-0.17554	0.000128	1
Ppp1ca	-0.17563	0.000126	1
Nbr1	-0.17572	2.31E-05	0.747371
Tbc1d4	-0.1761	0.000267	1
Cd14	-0.17611	1.05E-10	3.39E-06
Ubqln2	-0.17618	3.43E-08	0.001108
Prcp	-0.17651	0.000247	1
Armc3	-0.17653	7.65E-11	2.47E-06
Mertk	-0.17663	2.11E-07	0.006805
Tpst2	-0.17666	2.27E-06	0.073192
Tmem63a	-0.17671	0.005444	1
Hdlbp	-0.17684	0.006958	1
Sfpq	-0.17687	0.002755	1
Pnpla8	-0.17721	0.000135	1
Gm32036	-0.17741	3.23E-06	0.104334
Chsy1	-0.17744	1.74E-06	0.05606
Kcnj2	-0.17753	5.18E-05	1
Zmym4	-0.17771	0.015687	1

Map3k3	-0.17778	0.002289	1
Manea	-0.17837	0.000544	1
Ppm1e	-0.17871	8.89E-05	1
Mbd5	-0.17878	0.010758	1
Zmynd8	-0.17913	0.039348	1
Dmtf1	-0.17934	0.002274	1
Sppl3	-0.17945	0.051564	1
Cd151	-0.1795	0.007056	1
Prpf4b	-0.17965	0.056066	1
Arl3	-0.17988	8.3E-08	0.00268
Zmat1	-0.18013	9.3E-08	0.003003
Slc25a4	-0.18038	0.000748	1
Zfp638	-0.18039	0.002157	1
Il11ra1	-0.18039	7.68E-07	0.02481
Peg13	-0.18044	7.59E-15	2.45E-10
Cfap74	-0.18059	0.04202	1
Sf3b2	-0.18066	0.006548	1
Ranbp10	-0.18073	0.001406	1
Grk3	-0.18124	0.002431	1
Tspan14	-0.18172	9.38E-05	1
Urgcp	-0.18183	0.002974	1
Anapc2	-0.18197	0.000206	1
Supt3	-0.18199	0.000858	1
Bbc3	-0.18201	2.35E-05	0.757654
Itpripl1	-0.18208	4.69E-05	1
Sdccag8	-0.18216	0.001369	1
Pnp	-0.18249	6.43E-06	0.207683
Phactr2	-0.1826	0.063984	1
Fam173a	-0.18278	0.000996	1
Irf2	-0.18294	3.14E-06	0.101275
Tax1bp1	-0.18307	0.00073	1
Tubb2a	-0.18329	0.000655	1
Ceacam1	-0.18348	3.82E-07	0.012324
Trim36	-0.18348	6.77E-05	1
Magt1	-0.18349	0.008886	1
Zfp871	-0.1835	0.00054	1
Syngr2	-0.18372	3.05E-07	0.009848
Wdfy3	-0.18396	0.001588	1
Tmem141	-0.18405	6.8E-05	1
Cpsf6	-0.18412	7.84E-05	1
Tmtc2	-0.18431	9.33E-11	3.01E-06
Spg20	-0.18436	2.34E-06	0.075537
Rbm42	-0.18437	0.000229	1
Klhl36	-0.18457	5.35E-06	0.172673
Pnrc1	-0.18475	6.57E-07	0.021209
Celf1	-0.18476	0.003622	1
Hmox2	-0.1848	1.23E-05	0.395598
Ppfibp2	-0.18492	0.000342	1

Wdr44	-0.18498	0.01728	1
Trim2	-0.18512	3.71E-05	1
Ramac	-0.18517	1.15E-05	0.371998
Tnpo1	-0.18517	0.029917	1
Nck1	-0.1858	8.88E-05	1
Casd1	-0.18594	6.53E-05	1
Sema4d	-0.18611	8.68E-07	0.028039
Crebrf	-0.1862	3.96E-05	1
B3galt5	-0.18626	0.000427	1
Ssh2	-0.1864	5.69E-13	1.84E-08
Iscu	-0.18644	0.01202	1
Slc39a8	-0.18646	9.57E-08	0.003088
Rbbp4	-0.18671	1.75E-05	0.564754
Clta	-0.18672	8.34E-10	2.69E-05
Abcg2	-0.18672	0.00518	1
Slc35a5	-0.18712	1.81E-05	0.584219
Tgfbi	-0.18732	0.000159	1
Crim1	-0.18732	1.24E-07	0.003989
Pura	-0.1874	0.006203	1
Ing4	-0.18756	5.8E-05	1
Zfp467	-0.18759	0.00058	1
Zdhhc20	-0.18784	0.000474	1
Pcmtd2	-0.18817	9.32E-09	0.000301
Dagla	-0.18823	0.000346	1
Trp53	-0.18834	0.013127	1
Vav2	-0.18835	0.010258	1
Itm2c	-0.18845	1.56E-12	5.02E-08
Pepd	-0.18869	0.000913	1
Syf2	-0.18875	8.04E-06	0.259719
Ralgps1	-0.18892	0.019144	1
Rnf144b	-0.18956	1.39E-06	0.044958
Fam89b	-0.18969	0.000622	1
Rab1a	-0.1899	5.35E-05	1
Sept7	-0.19011	0.000112	1
Clptm1	-0.19019	0.00022	1
R3hdm2	-0.1902	0.027145	1
Dip2c	-0.1906	0.002496	1
Ncoa3	-0.19078	0.001356	1
Gtf2ird1	-0.19084	2.89E-08	0.000934
Hnrnph3	-0.19087	0.000499	1
Zfand6	-0.19089	0.000604	1
Gngt2	-0.19111	1.77E-05	0.570262
Senp7	-0.19119	0.000766	1
Tnrc18	-0.19123	0.000836	1
Creb1	-0.1913	0.000792	1
Arrb2	-0.19132	5.64E-09	0.000182
Emc3	-0.19158	2.61E-05	0.8427
Ncaph	-0.19165	3.7E-10	1.19E-05

Rassf5	-0.19177	0.000175	1
Tab2	-0.1918	2.11E-08	0.000683
Nrros	-0.19181	1.17E-09	3.76E-05
Dpm3	-0.19204	2.49E-06	0.080392
Herc2	-0.19212	2.51E-05	0.8105
Liph	-0.19213	0.010438	1
Rchy1	-0.19218	2.89E-05	0.932359
Usp33	-0.19238	0.000476	1
Ralb	-0.19243	2.24E-07	0.007242
Arhgap17	-0.19256	7.6E-06	0.245238
Slc9a7	-0.19294	1.8E-06	0.058133
Cd37	-0.19299	1.09E-11	3.52E-07
Cd276	-0.19318	3.44E-12	1.11E-07
Sp1	-0.19372	1.53E-07	0.004929
Bicral	-0.19409	0.000247	1
Frmd8	-0.19443	0.000268	1
Papola	-0.19449	1.85E-05	0.596688
Stard3	-0.1947	1.73E-05	0.559355
Dctn5	-0.19474	9.42E-05	1
Acvr1	-0.19475	0.000606	1
Aagab	-0.19553	6.93E-05	1
Parp8	-0.19554	0.022142	1
Prps2	-0.19569	6.82E-07	0.022024
Oma1	-0.19583	1.74E-05	0.560171
Skil	-0.19656	0.000354	1
Gm3252	-0.19668	4.21E-19	1.36E-14
Mfhas1	-0.19699	0.022467	1
Bcor	-0.19709	8.84E-06	0.285451
Bckdha	-0.19739	5.04E-05	1
Itch	-0.19779	0.001121	1
Eif1b	-0.19783	7.18E-05	1
Zfp715	-0.19797	3.37E-06	0.108827
Fam91a1	-0.198	8.1E-07	0.026149
Sp3	-0.19825	0.000602	1
Tcn2	-0.19836	3.34E-08	0.001077
Ak1	-0.19837	0.000729	1
Ube2d2a	-0.1985	5.04E-06	0.162689
Maf1	-0.19862	3.2E-07	0.010347
Tbxas1	-0.19917	2.24E-06	0.072178
Fhod1	-0.19918	2.78E-05	0.897
Slc25a45	-0.19924	0.000297	1
Mcfd2	-0.19931	5.73E-05	1
Commd7	-0.19942	0.0077	1
Stt3a	-0.19961	1.16E-05	0.375602
G3bp2	-0.19966	1.57E-06	0.050529
Phlda3	-0.19984	5.84E-08	0.001885
Arpc4	-0.20012	9.6E-10	3.1E-05
Nf1	-0.20035	0.001819	1

Ctso	-0.20088	0.000194	1
Lrch1	-0.20103	1.39E-05	0.448063
Lpcat1	-0.20103	0.000463	1
Trio	-0.20115	0.079318	1
Zfp950	-0.20124	0.000496	1
Idh1	-0.2014	2E-07	0.006473
Evi5	-0.20165	0.012841	1
Zbtb4	-0.20166	5.38E-06	0.173676
Crebbp	-0.20185	2.39E-05	0.77149
Rgs7bp	-0.20207	0.003044	1
Ppp1r2	-0.20245	4.28E-07	0.013828
Kcnd1	-0.20249	6.2E-06	0.200299
Cat	-0.2027	7.96E-06	0.257006
Tbl1xr1	-0.203	0.000973	1
Pbx2	-0.20307	2.23E-08	0.000719
Mgmt	-0.20319	0.011722	1
Fam3a	-0.20355	2.34E-07	0.007547
Ythdf2	-0.20393	1.53E-05	0.492946
Tceal1	-0.20407	6.05E-18	1.95E-13
Ube2e3	-0.20436	3.55E-07	0.011454
Tasor	-0.20437	0.000183	1
Tmem223	-0.20438	1.41E-06	0.045431
Galnt4	-0.20445	6.55E-09	0.000211
Ids	-0.20463	1.28E-05	0.414661
Flcn	-0.20474	0.000172	1
Stag2	-0.20479	7.86E-05	1
Ppp1r3b	-0.20494	7.14E-08	0.002305
Tmem106c	-0.20513	1.06E-06	0.034378
Nek6	-0.2052	0.000384	1
Rnf5	-0.2053	1.56E-05	0.502566
Pum2	-0.20538	3.05E-05	0.98317
Tm2d2	-0.2058	8.42E-06	0.271812
Hdac5	-0.20587	1.36E-05	0.438565
Tut4	-0.206	5.13E-09	0.000166
Ebf3	-0.20623	4.92E-06	0.158862
Elmod2	-0.2064	7.1E-07	0.022934
Nrm	-0.2065	4.51E-09	0.000146
Selenok	-0.20693	2.63E-08	0.000849
Klhl24	-0.20717	3.28E-07	0.010576
Rfx3	-0.20735	8.32E-07	0.026874
Mkln1	-0.20738	0.001055	1
Stag1	-0.20746	0.002116	1
Itpr2	-0.20758	5E-05	1
Erf	-0.20814	6.09E-06	0.196711
Trpc4ap	-0.2088	2.57E-06	0.083016
Kpnb1	-0.20884	1.52E-05	0.491187
Arsg	-0.20887	0.000443	1
Gm3264	-0.2092	1.21E-18	3.9E-14

Aup1	-0.20921	2.22E-08	0.000717
Wdr1	-0.20922	1.61E-07	0.005206
Rela	-0.20933	0.000155	1
Kdm3b	-0.20977	1.67E-05	0.539696
Sesn1	-0.21013	4.96E-08	0.001601
Nars	-0.21024	4.58E-06	0.14772
Mvb12b	-0.21033	0.000406	1
Kifap3	-0.21034	1.63E-06	0.052774
Mbnl2	-0.21036	1.55E-06	0.050178
Rac1	-0.21054	1.81E-05	0.585472
Canx	-0.21092	1.3E-07	0.004185
Atxn2l	-0.21122	0.000197	1
Anp32a	-0.21133	9.04E-07	0.029179
Rbmx	-0.21136	1.14E-05	0.367911
Cbx1	-0.21155	9.66E-05	1
Spi1	-0.21163	8.8E-12	2.84E-07
Ociad1	-0.21191	9.32E-08	0.003008
Prkacb	-0.21205	1.53E-06	0.049359
Pex2	-0.21207	3.41E-06	0.110062
Slc39a10	-0.2123	2.75E-07	0.008888
Hist1h2be	-0.2128	1.15E-09	3.7E-05
Creb3l2	-0.21293	1.16E-05	0.375806
Tmem43	-0.21296	1.29E-08	0.000417
Foxn3	-0.21302	2.67E-11	8.62E-07
Zfp62	-0.21312	1.22E-05	0.394052
Add1	-0.21323	1.28E-07	0.004118
4933434E2	-0.21332	5.09E-06	0.164488
Lpin2	-0.21406	1.79E-08	0.000577
Cmss1	-0.21413	9.72E-10	3.14E-05
Btk	-0.21425	4.07E-06	0.131331
Isyna1	-0.2143	5.57E-06	0.179682
Igfbp4	-0.21466	1.3E-06	0.041973
Arl6ip1	-0.21466	5E-07	0.016132
Tubb2b	-0.2147	1.18E-06	0.038076
Stambpl1	-0.21488	1.69E-06	0.054414
Cpne3	-0.21498	0.000177	1
Hnrnph1	-0.2152	7.73E-05	1
Cops4	-0.21531	1.8E-06	0.058048
Cysltr1	-0.21535	3.39E-07	0.010961
Srpk3	-0.21552	1.68E-17	5.43E-13
Zswim6	-0.21568	0.002446	1
Nfatc2	-0.21591	3.52E-05	1
Clec4a2	-0.21621	7.49E-10	2.42E-05
Fnta	-0.21627	9.26E-08	0.00299
Scmh1	-0.21639	0.000843	1
Grk2	-0.21651	0.005758	1
Rpn1	-0.21655	7.62E-08	0.002461
Cmya5	-0.21715	1.54E-12	4.98E-08

Adap2	-0.21719	7.21E-08	0.002326
Ext2	-0.21764	0.000408	1
Ykt6	-0.21781	1.99E-06	0.064339
Vps28	-0.21828	0.000742	1
Rab11b	-0.21843	2.44E-09	7.87E-05
Mknk1	-0.21848	7.88E-09	0.000254
Zfp691	-0.21864	7.31E-09	0.000236
Slc39a12	-0.21866	2.7E-16	8.71E-12
Myo18a	-0.21868	0.026012	1
Tmem86a	-0.21948	7.05E-08	0.002275
Ipo11	-0.21962	3.02E-07	0.009746
Bicd2	-0.21998	4.62E-06	0.149011
Apba1	-0.22031	0.000565	1
Sash3	-0.22092	4.86E-08	0.001569
Helz	-0.22098	3.83E-05	1
Krcc1	-0.2213	6.37E-06	0.205549
Eif1	-0.22158	2.12E-12	6.84E-08
Tnrc6b	-0.22196	2.09E-09	6.75E-05
Nfya	-0.22258	0.000336	1
Ccdc90b	-0.22278	1.09E-08	0.000353
Pigp	-0.22279	3.07E-06	0.099197
Sh2b3	-0.2235	2.09E-06	0.067514
Vhl	-0.22363	4.22E-05	1
Inpp5d	-0.22376	2.49E-14	8.04E-10
Bmp2k	-0.22404	1.78E-07	0.00575
Tmed10	-0.22411	7.16E-11	2.31E-06
Adam17	-0.22432	1.15E-06	0.037012
Csnk1a1	-0.22448	6.54E-08	0.002111
Ddx3x	-0.22464	1.81E-05	0.584143
Cnrip1	-0.22478	9.17E-09	0.000296
Tmem52	-0.2249	1.27E-10	4.11E-06
Gadd45g	-0.22512	2.47E-07	0.007983
Ints3	-0.22521	8.17E-08	0.002639
Gm35188	-0.22528	8.8E-07	0.028406
Poc1b	-0.22531	1.78E-06	0.057382
Nfic	-0.22533	1.28E-06	0.04123
Hnrnpk	-0.2254	1.84E-11	5.94E-07
Tcf4	-0.22566	4.16E-06	0.134396
Gm46367	-0.22568	1.1E-08	0.000355
Stau2	-0.22637	0.000165	1
Dele1	-0.22638	1.36E-06	0.043905
Clcn5	-0.22653	2.33E-06	0.075317
Trim47	-0.22679	4.57E-05	1
Purb	-0.22679	1.53E-05	0.49342
Slc4a7	-0.22718	0.000186	1
Tmem175	-0.22771	3.09E-06	0.099911
Slc18b1	-0.22772	3E-13	9.69E-09
Ang	-0.22782	6.32E-05	1

Syk	-0.22789	5.8E-07	0.018717
Papss1	-0.2281	7.16E-07	0.023105
Adgrl2	-0.22811	4.74E-07	0.015292
Arhgef10l	-0.22819	3.87E-12	1.25E-07
Add3	-0.22835	8.65E-08	0.002794
Ptbp1	-0.2285	7.5E-07	0.024198
Lasp1	-0.22864	6.38E-10	2.06E-05
Tspan4	-0.22879	8.47E-08	0.002735
Smad1	-0.22914	2.21E-05	0.714245
Tor4a	-0.2296	2.87E-06	0.092743
Clstn1	-0.22966	4.92E-08	0.00159
Rasal3	-0.22971	1.23E-09	3.98E-05
Nhsl2	-0.23001	5.93E-18	1.92E-13
Dcaf12	-0.23003	3.34E-08	0.00108
Kpna3	-0.23013	1.25E-07	0.004045
Cyren	-0.23019	8.63E-10	2.79E-05
Smyd3	-0.23028	0.000356	1
Txndc15	-0.23036	3.69E-07	0.011922
Gm31243	-0.23043	3.16E-13	1.02E-08
Zfp869	-0.23067	2.13E-08	0.000689
Ddx17	-0.23089	0.000435	1
Fez2	-0.23121	1.79E-06	0.057715
Aftph	-0.23126	0.000693	1
Rtn3	-0.23136	4.18E-11	1.35E-06
Man2b2	-0.23207	8.99E-06	0.290292
Rsu1	-0.23225	8.78E-07	0.028335
Tiprl	-0.2324	9.29E-06	0.299974
Stard8	-0.233	3.32E-07	0.010725
Kansl1l	-0.23365	1.68E-05	0.541345
Mkrn1	-0.23375	2.09E-06	0.067409
1600022D1	-0.23423	1.05E-15	3.37E-11
Gm3164	-0.23448	1.61E-23	5.19E-19
Mast3	-0.23566	1.27E-06	0.041102
Tspyl1	-0.23576	4.5E-10	1.45E-05
Snx14	-0.23586	1.67E-09	5.4E-05
Arrb1	-0.23609	5.24E-10	1.69E-05
Pafah1b1	-0.23682	5.6E-08	0.001807
Prkar1a	-0.23709	7.32E-12	2.36E-07
Mgrn1	-0.2371	6.2E-08	0.002001
Gm9949	-0.23713	5.48E-10	1.77E-05
Bend6	-0.23749	6.84E-16	2.21E-11
Aars	-0.23759	3.62E-08	0.001167
Man1a2	-0.23824	1.01E-06	0.032513
Il10rb	-0.23845	1.65E-09	5.32E-05
Cib1	-0.23888	9.15E-09	0.000295
Slc12a6	-0.23896	5.75E-05	1
Sall3	-0.23914	1.72E-06	0.055521
BC035044	-0.23974	2.99E-06	0.096527

Gm3411	-0.23998	3.64E-20	1.17E-15
Fam117b	-0.24017	0.000278	1
Rnf130	-0.2402	8.5E-07	0.027429
Vrk1	-0.24062	1.74E-06	0.056111
Exoc3	-0.24074	1.18E-05	0.382509
Gm43821	-0.24075	5.2E-12	1.68E-07
2900052L1	-0.24111	2.4E-15	7.74E-11
Clip2	-0.24138	1.94E-05	0.626084
Bag6	-0.24143	7.64E-11	2.47E-06
Fam114a2	-0.24228	1.08E-05	0.349827
Wdr81	-0.24232	1.6E-10	5.16E-06
Cbx3	-0.24252	1.84E-07	0.005939
Cerk	-0.24259	2.11E-08	0.000682
Cul1	-0.24299	1.75E-05	0.564769
Mindy2	-0.24309	2.19E-06	0.070795
Gm30329	-0.24338	5.2E-20	1.68E-15
Gabarapl2	-0.24358	1.11E-05	0.357075
Cbfa2t3	-0.24373	4.15E-10	1.34E-05
Arhgef6	-0.24383	2.7E-07	0.00872
Arhgap25	-0.24429	5.78E-07	0.018667
Gm35154	-0.24446	5.34E-06	0.172434
Plscr3	-0.2449	8.12E-09	0.000262
Phf1	-0.24493	3.66E-13	1.18E-08
Itgb3	-0.24517	9.84E-10	3.18E-05
Swap70	-0.24604	9.86E-08	0.003183
Rp2	-0.24638	5.62E-06	0.181306
Hist1h1e	-0.24638	1.26E-05	0.405696
Cnot2	-0.24643	1.74E-05	0.561223
Zfp934	-0.24653	1.09E-09	3.51E-05
Gm3173	-0.2468	3.23E-28	1.04E-23
Rhoa	-0.24762	3.52E-14	1.14E-09
Rgs19	-0.24803	1.4E-10	4.53E-06
Hmgb1	-0.24829	1.31E-09	4.23E-05
Wbp2	-0.24844	1.34E-06	0.043315
Htra2	-0.24861	8.53E-08	0.002755
4632427E1	-0.24865	5.93E-07	0.01915
Ddx5	-0.24918	3.22E-23	1.04E-18
Abcd3	-0.24928	7.1E-10	2.29E-05
Glg1	-0.24936	5.67E-07	0.018295
Fbrsl1	-0.24942	3.82E-06	0.123312
3222401L1	-0.24991	4.7E-14	1.52E-09
Arl4c	-0.24994	3.13E-09	0.000101
Gstp1	-0.25025	3.17E-08	0.001023
Arf1	-0.25043	6.63E-13	2.14E-08
Tom1l1	-0.25146	2.23E-14	7.19E-10
Gprasp1	-0.25148	2.67E-14	8.62E-10
Nrp1	-0.25153	1.67E-06	0.053888
Dtx4	-0.2517	3.66E-11	1.18E-06

Rap1a	-0.25192	1.86E-08	0.0006
Ttc7	-0.25207	9.2E-07	0.029701
Ywhah	-0.25327	1.96E-10	6.31E-06
Crat	-0.25348	5.73E-09	0.000185
Tifab	-0.25349	1.82E-07	0.005872
Snx13	-0.25354	4.13E-07	0.013323
Ywhaz	-0.2536	3.01E-13	9.71E-09
Lrch4	-0.25363	1.5E-09	4.85E-05
Prpf8	-0.25373	3.32E-08	0.001071
Entpd1	-0.2539	2.22E-22	7.17E-18
Grcc10	-0.25403	5.53E-08	0.001786
Ets1	-0.25467	4.59E-27	1.48E-22
Pknox1	-0.25497	8.84E-08	0.002854
Inka2	-0.25511	8.98E-08	0.002899
Arvcf	-0.25518	1.35E-14	4.35E-10
Tsen34	-0.25521	3.74E-08	0.001208
Arl10	-0.25539	2.47E-11	7.97E-07
Rnaset2a	-0.25624	1.03E-06	0.033256
Rgl2	-0.25689	4.18E-11	1.35E-06
Hmg20a	-0.2571	7.66E-09	0.000247
Dnmt3a	-0.25729	0.004584	1
Dync1i2	-0.25757	1.5E-08	0.000483
Smpd1	-0.2581	8.01E-13	2.58E-08
Pik3r5	-0.25826	6.51E-07	0.02101
Tpm3	-0.25843	1.57E-14	5.08E-10
Slc37a2	-0.2586	4.23E-09	0.000137
Immp2l	-0.25877	0.004567	1
Usp24	-0.25879	1.53E-07	0.004929
Klk8	-0.25888	6.4E-09	0.000206
Ttc3	-0.25923	1.34E-06	0.043178
Accs	-0.25938	1.85E-09	5.96E-05
Ctnnb1	-0.25944	1.27E-09	4.11E-05
Ppp6r1	-0.25961	1.26E-08	0.000407
Kmt5b	-0.25973	3.16E-06	0.101955
Cnpy2	-0.26009	2.24E-07	0.007223
I830077J0	-0.26044	1.27E-08	0.000411
Cdc42	-0.26097	1.2E-21	3.86E-17
Saysd1	-0.2613	3.92E-10	1.26E-05
Retreg1	-0.2616	6.07E-07	0.01961
Acads	-0.26162	3.79E-09	0.000122
Zfp608	-0.26196	4.06E-07	0.013092
Arid1a	-0.26229	5.72E-09	0.000185
Adam10	-0.26234	1.52E-09	4.9E-05
Arhgap4	-0.26254	1.19E-10	3.84E-06
Morf4l1	-0.26289	7.77E-10	2.51E-05
Ctbp2	-0.26349	9.52E-11	3.08E-06
Zfp385a	-0.2638	1.16E-08	0.000373
Prag1	-0.26426	2.92E-12	9.42E-08

Arhgef2	-0.26436	6.67E-08	0.002154
Slc25a11	-0.26454	6.27E-09	0.000202
9130015G1	-0.26464	5.48E-14	1.77E-09
Tpbgl	-0.26494	1.05E-06	0.033938
Tmem50a	-0.26527	8.36E-17	2.7E-12
Cap1	-0.26589	1.75E-15	5.65E-11
Clcn4	-0.26597	4.99E-07	0.016097
Atp6v0a1	-0.2662	1.02E-09	3.3E-05
Arl1	-0.2662	8.29E-09	0.000268
Efnb1	-0.26666	7.56E-21	2.44E-16
Sik2	-0.26692	1.51E-07	0.004869
Tk2	-0.26704	1.65E-09	5.34E-05
Akna	-0.26721	5.96E-10	1.93E-05
Arhgap12	-0.26735	3.71E-07	0.011973
Gpr137b	-0.26752	1.71E-09	5.52E-05
Gm37494	-0.26792	2.25E-10	7.26E-06
Sec14l1	-0.2682	4.01E-09	0.00013
Cln8	-0.26841	1.28E-08	0.000413
Kat6a	-0.26857	4.32E-07	0.013943
Kdelr1	-0.26927	2.92E-09	9.44E-05
Ep300	-0.26944	5.97E-10	1.93E-05
Sgms1	-0.26991	6.54E-07	0.021119
Taz	-0.26992	4.92E-11	1.59E-06
Rasa3	-0.26992	3.91E-08	0.001263
Tmem30a	-0.27026	1.12E-10	3.6E-06
2610035D1	-0.27101	3.33E-21	1.08E-16
Tgfbrap1	-0.27118	6.48E-11	2.09E-06
Mapk14	-0.27119	1.32E-07	0.00425
Smox	-0.27131	6.4E-11	2.07E-06
Vps35	-0.2716	3.8E-08	0.001227
Arl8b	-0.27177	1.36E-09	4.39E-05
Sh3pxd2a	-0.27194	0.000836	1
Tmem37	-0.27207	2.36E-09	7.6E-05
Zbtb20	-0.27247	2.67E-07	0.008607
Tmem173	-0.27254	3.7E-14	1.19E-09
Hspb3	-0.27371	7.28E-15	2.35E-10
Plpp1	-0.27423	4.1E-09	0.000132
Chchd2	-0.27427	1.35E-08	0.000435
Adam3	-0.27434	5.54E-11	1.79E-06
Endod1	-0.27434	3.7E-10	1.2E-05
Stat6	-0.27505	1.62E-09	5.25E-05
Slc16a7	-0.27575	7.56E-08	0.00244
S1pr1	-0.2763	3.53E-24	1.14E-19
Aamp	-0.27631	2.33E-13	7.51E-09
Abcd2	-0.27688	1.88E-08	0.000607
Slmap	-0.27747	2.48E-08	0.000801
Phc2	-0.27756	3.36E-07	0.010856
Pttg1ip	-0.27821	3.38E-11	1.09E-06

Creg1	-0.27996	2.28E-13	7.35E-09
Chst8	-0.28047	7.1E-20	2.29E-15
Paip2	-0.28052	3.92E-10	1.26E-05
Smad3	-0.28062	2.88E-07	0.009311
Rhobtb1	-0.28079	1.37E-11	4.44E-07
Dpysl2	-0.28118	4.4E-09	0.000142
Casp8	-0.28127	1.81E-08	0.000585
Mob1a	-0.28137	1.16E-10	3.75E-06
Ccdc50	-0.28162	5.04E-08	0.001626
Nr6a1os	-0.28168	2.16E-09	6.97E-05
Pxn	-0.28197	1.44E-09	4.66E-05
Rab31	-0.28214	1.09E-13	3.53E-09
Son	-0.2824	5.73E-22	1.85E-17
Etv1	-0.28291	2.57E-09	8.28E-05
Usp22	-0.28306	1.87E-13	6.02E-09
Eml6	-0.28308	1.02E-06	0.033084
Dusp7	-0.28341	2.59E-13	8.35E-09
Gnas	-0.28367	8.75E-10	2.82E-05
Cnpy3	-0.28382	6.58E-12	2.12E-07
Clasp2	-0.2839	3.46E-13	1.12E-08
Hps4	-0.28401	5.06E-11	1.63E-06
Ski	-0.28574	8.92E-07	0.028795
Lactb2	-0.28594	6.16E-10	1.99E-05
Stk10	-0.286	1.35E-13	4.35E-09
Tbc1d9b	-0.28613	1.15E-07	0.003714
Rab39	-0.28614	1.69E-11	5.45E-07
Tmem109	-0.28635	1.83E-11	5.9E-07
Arhgef1	-0.28745	5.24E-15	1.69E-10
Gcnt1	-0.28751	7.63E-11	2.46E-06
Slc25a37	-0.28761	4.49E-14	1.45E-09
Cd302	-0.28785	7.89E-11	2.55E-06
B4gat1	-0.2879	1.2E-13	3.86E-09
Arid3a	-0.28831	1.53E-12	4.95E-08
Pja2	-0.28904	5.24E-11	1.69E-06
Zranb2	-0.28906	2.97E-06	0.095944
Adcy9	-0.28917	8.15E-07	0.026317
Inka1	-0.28968	5.37E-09	0.000173
Map1s	-0.28991	2.29E-10	7.4E-06
Pdlim4	-0.29018	6.12E-10	1.98E-05
Lims1	-0.29024	1.98E-13	6.38E-09
Disc1	-0.29025	1.65E-05	0.531107
Slc8a1	-0.29276	4.12E-12	1.33E-07
Ctsa	-0.29363	2.19E-25	7.07E-21
Mboat7	-0.29391	1.06E-10	3.42E-06
Nfatc1	-0.2942	6.62E-11	2.14E-06
H1f0	-0.29442	3.19E-12	1.03E-07
Kdm2b	-0.29447	9.1E-13	2.94E-08
6030468B:	-0.29547	5.75E-19	1.86E-14

Ccdc12	-0.29598	3.2E-13	1.03E-08
Ccdc162	-0.29619	1.6E-27	5.17E-23
Wdfy2	-0.29666	3.34E-08	0.001078
Atp6v0b	-0.29667	8.55E-25	2.76E-20
Maged1	-0.29676	1.35E-22	4.37E-18
Scamp2	-0.29682	7.49E-26	2.42E-21
Adgre1	-0.29728	1.84E-10	5.93E-06
Hivep3	-0.2973	3.34E-06	0.107709
Hnrnpul1	-0.29734	2.09E-14	6.75E-10
Ramp1	-0.29811	7.97E-09	0.000257
Cirbp	-0.29822	1.49E-13	4.8E-09
Sh2b2	-0.29849	2.19E-11	7.08E-07
P4ha1	-0.2986	3.35E-11	1.08E-06
Epn2	-0.29893	9.34E-15	3.01E-10
Klf7	-0.29916	4.05E-11	1.31E-06
Arhgap39	-0.29929	5.49E-06	0.177157
Rhob	-0.29951	5.79E-33	1.87E-28
Tuba1b	-0.29969	5.55E-09	0.000179
P2ry10b	-0.30038	7.27E-20	2.35E-15
Cmtm7	-0.30087	1.63E-16	5.25E-12
Gna12	-0.30091	8.43E-06	0.27216
Lars2	-0.3014	1.73E-13	5.58E-09
Tmem59	-0.30158	1.97E-19	6.36E-15
Tnfrsf1b	-0.30167	1.24E-13	4E-09
Macf1	-0.30172	7.78E-13	2.51E-08
Samsn1	-0.30199	1.26E-15	4.06E-11
Ywhae	-0.30258	1.71E-14	5.52E-10
Smarca2	-0.3027	6.19E-09	0.0002
1700011L2	-0.30321	4.5E-23	1.45E-18
Ppp1r18	-0.30327	1.31E-14	4.24E-10
Dock4	-0.3035	1.75E-15	5.65E-11
Sema4b	-0.30398	1.42E-25	4.59E-21
Cables1	-0.30511	4.29E-06	0.138517
Itgam	-0.30524	4.96E-25	1.6E-20
Glis3	-0.30533	4.53E-18	1.46E-13
Arhgap22	-0.30569	2.19E-08	0.000706
Matk	-0.30569	1.4E-21	4.53E-17
Laptm5	-0.30584	4.09E-58	1.32E-53
Med13l	-0.30596	1.53E-09	4.95E-05
Ddx6	-0.30625	7.05E-11	2.28E-06
Cass4	-0.30841	8.4E-17	2.71E-12
Jup	-0.30844	1.26E-14	4.07E-10
Rere	-0.3087	1.46E-11	4.73E-07
Ccdc93	-0.30874	1.67E-11	5.39E-07
Atraid	-0.30913	2.49E-14	8.05E-10
Crot	-0.30915	1.34E-20	4.33E-16
Wsb1	-0.30938	1.71E-13	5.51E-09
Ehd4	-0.30949	2.05E-14	6.62E-10

Gm3002	-0.30974	1.99E-34	6.41E-30
Mfap3	-0.31019	4.94E-11	1.59E-06
Cnot8	-0.3104	3.9E-11	1.26E-06
Pid1	-0.31178	1.46E-08	0.000473
Clec4a3	-0.31227	5.28E-12	1.71E-07
Cd33	-0.31253	6.48E-23	2.09E-18
Tram1	-0.31266	4.71E-19	1.52E-14
Cyb5a	-0.31292	2.97E-13	9.58E-09
Prpsap2	-0.31308	1.06E-10	3.43E-06
Hhex	-0.31337	3.76E-14	1.21E-09
Cmtm8	-0.31369	5.83E-16	1.88E-11
Mrfap1	-0.31412	1.11E-11	3.57E-07
Gmpr	-0.31429	2.66E-15	8.59E-11
Cbl	-0.31502	1.15E-14	3.7E-10
Col15a1	-0.31595	7.14E-12	2.31E-07
Rab14	-0.31646	5.09E-22	1.64E-17
Tmem64	-0.31653	2.87E-15	9.26E-11
Tpcn1	-0.31682	7.76E-14	2.51E-09
Gm3248	-0.31706	8.63E-28	2.79E-23
Zfp532	-0.31723	3.27E-11	1.06E-06
Pbx1	-0.3174	1.42E-12	4.6E-08
Myo1d	-0.31747	1.73E-33	5.58E-29
Tspan7	-0.31848	1.2E-13	3.87E-09
Mlec	-0.31878	5.92E-14	1.91E-09
Dock1	-0.32026	1.52E-10	4.92E-06
Serinc1	-0.32068	3.92E-16	1.27E-11
Rtn4	-0.32073	8.03E-22	2.59E-17
Rnf7	-0.32116	3.05E-12	9.86E-08
Itgb1	-0.32133	2.43E-17	7.85E-13
Ralgps2	-0.32136	8.52E-11	2.75E-06
Ubc	-0.3218	6.14E-29	1.98E-24
Mib2	-0.32258	2.23E-19	7.2E-15
Wls	-0.32263	8.92E-13	2.88E-08
Frmd4b	-0.32334	1.49E-19	4.81E-15
Fer	-0.32342	4.99E-10	1.61E-05
Zeb1	-0.32396	2.34E-08	0.000755
Tln2	-0.3241	2.42E-08	0.000781
Acss1	-0.32439	2.15E-14	6.93E-10
Atp2c1	-0.32464	2.12E-13	6.83E-09
Zeb2os	-0.32486	2.96E-12	9.54E-08
Ctnnd2	-0.32512	5.41E-28	1.75E-23
Sipa1	-0.32523	1.13E-17	3.64E-13
Mgat5	-0.32537	1.05E-08	0.000338
Map2k1	-0.3256	7.3E-10	2.36E-05
Hsd17b11	-0.32567	1.35E-15	4.37E-11
Abcb4	-0.32574	1.29E-23	4.17E-19
Abi3	-0.32687	4.1E-26	1.32E-21
Sec62	-0.32719	1.27E-11	4.11E-07

Sypl	-0.32804	5.79E-15	1.87E-10
Rin2	-0.32865	1.56E-20	5.05E-16
Mapk3	-0.32918	1.28E-13	4.13E-09
Il6ra	-0.32967	2.91E-18	9.4E-14
Sbf2	-0.33044	1.04E-11	3.34E-07
Naa35	-0.33099	8.36E-16	2.7E-11
Large1	-0.33133	2.2E-14	7.1E-10
Map4k4	-0.33151	2.76E-09	8.91E-05
Pik3cg	-0.33182	5.08E-16	1.64E-11
Hook3	-0.33234	2.61E-09	8.43E-05
Acp2	-0.33237	3.8E-14	1.23E-09
Rsrp1	-0.3327	1.32E-21	4.27E-17
Slc35c2	-0.33284	7.27E-17	2.35E-12
Tbc1d5	-0.3329	2.67E-15	8.61E-11
Hp1bp3	-0.33367	3.97E-16	1.28E-11
Bach2	-0.33462	1.99E-13	6.42E-09
Ldhb	-0.33473	9.92E-20	3.2E-15
Tm9sf2	-0.33544	3.71E-18	1.2E-13
Mbnl1	-0.3356	9.44E-36	3.05E-31
Slc24a3	-0.33658	3.32E-07	0.010725
Atxn7l3b	-0.33684	2.14E-11	6.91E-07
Lag3	-0.33805	3.21E-13	1.04E-08
Psenen	-0.33826	2.34E-16	7.55E-12
Cdk5r1	-0.33874	1.5E-30	4.85E-26
Nr3c2	-0.33926	2.61E-13	8.42E-09
Rap1gds1	-0.33937	4.31E-16	1.39E-11
Tmcc3	-0.33971	1.02E-13	3.31E-09
Fgfr2	-0.33985	5.93E-14	1.92E-09
Ubl3	-0.34041	2.34E-18	7.57E-14
Pde4d	-0.34046	1.79E-13	5.78E-09
Lysmd4	-0.34075	7.15E-17	2.31E-12
Rilpl1	-0.34078	3.11E-16	1E-11
Stab1	-0.34147	6.86E-20	2.22E-15
Tacc1	-0.34166	4.86E-17	1.57E-12
Gfm2	-0.34226	7.04E-14	2.27E-09
Rbfox1	-0.34337	7.05E-15	2.28E-10
Dennd2c	-0.34392	2.01E-16	6.5E-12
Rab6b	-0.34422	5.76E-18	1.86E-13
Git2	-0.3443	1.74E-22	5.63E-18
Tmco1	-0.34494	2.64E-15	8.54E-11
Nrip1	-0.34523	1.94E-16	6.28E-12
Siglece	-0.34645	2.17E-13	7E-09
Pla2g4a	-0.3465	3.97E-12	1.28E-07
Rabac1	-0.34662	1.71E-20	5.51E-16
Cadm1	-0.34665	3.23E-08	0.001044
Pld1	-0.3467	4.15E-13	1.34E-08
Npc1	-0.34721	7.08E-15	2.29E-10
Abca1	-0.34746	4.61E-17	1.49E-12

Dglucy	-0.34771	7.38E-10	2.38E-05
Bmpr2	-0.34791	5.36E-13	1.73E-08
Chn2	-0.34795	3.94E-18	1.27E-13
Chst7	-0.34872	2.69E-13	8.7E-09
Asb2	-0.34945	4.28E-13	1.38E-08
Dok3	-0.35052	1.45E-16	4.69E-12
Ccl6	-0.35113	7.29E-07	0.023539
Comt	-0.35205	3.39E-21	1.09E-16
Sipa1l1	-0.35219	6.15E-18	1.99E-13
Mfng	-0.35229	1.03E-18	3.31E-14
Ctc1	-0.3523	5.33E-18	1.72E-13
Pla2g15	-0.35234	8.75E-21	2.83E-16
Csad	-0.35237	2.05E-17	6.63E-13
Ncoa4	-0.35242	8.49E-17	2.74E-12
Hnmt	-0.35341	8.75E-27	2.83E-22
Gm42418	-0.35381	1.56E-14	5.05E-10
Snx17	-0.35407	1.4E-15	4.51E-11
Snta1	-0.35448	2.82E-16	9.1E-12
Lipa	-0.35733	7.7E-17	2.49E-12
Csf3r	-0.35917	1.13E-16	3.64E-12
C5ar2	-0.3596	2.19E-18	7.08E-14
Nfam1	-0.36008	3.95E-15	1.28E-10
Pald1	-0.36182	1.54E-15	4.97E-11
Nav1	-0.36228	1.8E-15	5.8E-11
Iffo1	-0.36271	1.53E-15	4.95E-11
Twf2	-0.36299	7.12E-19	2.3E-14
Man1a	-0.363	6.61E-23	2.13E-18
Ulk2	-0.36319	1.54E-13	4.96E-09
Nr3c1	-0.36321	1.15E-13	3.71E-09
Tmem144	-0.36376	4.17E-19	1.35E-14
Dusp6	-0.36398	7E-24	2.26E-19
Prrc2a	-0.36418	9.35E-19	3.02E-14
Ptpra	-0.3642	1.78E-16	5.74E-12
Tal1	-0.3642	3.4E-19	1.1E-14
Slc29a1	-0.36457	4.6E-16	1.48E-11
Cyb561a3	-0.36527	3.48E-13	1.12E-08
Adrb2	-0.36613	1.1E-26	3.56E-22
Tmem204	-0.36676	4.24E-36	1.37E-31
Sec11c	-0.36795	1.8E-21	5.81E-17
Nbea	-0.36809	7.46E-07	0.024086
Mtus1	-0.36931	2.6E-21	8.39E-17
Zfp697	-0.36958	1.47E-20	4.73E-16
Klhl9	-0.37308	3.96E-21	1.28E-16
Dnm2	-0.37322	1.05E-23	3.39E-19
Klhl7	-0.37357	8.24E-18	2.66E-13
Fcrl1	-0.37402	2.09E-25	6.74E-21
Tsc22d4	-0.37411	1.49E-24	4.81E-20
Tpp1	-0.37436	2.35E-24	7.58E-20

Rcsd1	-0.37438	3.36E-21	1.09E-16
Ccng2	-0.37467	7.39E-20	2.39E-15
Tnfrsf17	-0.37612	5.32E-34	1.72E-29
Soga1	-0.37658	2.41E-12	7.77E-08
Cdh23	-0.37663	9.87E-17	3.19E-12
Ggta1	-0.37729	1.01E-18	3.24E-14
Adprh	-0.37793	5.48E-17	1.77E-12
Rps6ka1	-0.3786	5.85E-28	1.89E-23
Tm6sf1	-0.37951	2.31E-26	7.44E-22
Blnk	-0.38024	2.77E-22	8.96E-18
Tnfrsf13b	-0.38041	8.21E-18	2.65E-13
Prkab1	-0.38079	2.4E-18	7.76E-14
Nuak1	-0.3816	3.97E-12	1.28E-07
Arhgap31	-0.38222	4.56E-10	1.47E-05
Slc44a2	-0.38269	1.09E-23	3.52E-19
Pou2f2	-0.38323	4.67E-23	1.51E-18
Peak1	-0.38329	6.83E-20	2.2E-15
Gm2237	-0.38428	1.18E-30	3.81E-26
Il16	-0.38529	3.57E-24	1.15E-19
Gab1	-0.38531	1.29E-22	4.17E-18
Mef2c	-0.38543	1.28E-36	4.14E-32
Zmiz1	-0.38688	2.86E-20	9.24E-16
Acap2	-0.387	1.82E-24	5.86E-20
Rnf216	-0.38729	3.81E-21	1.23E-16
Gtf2i	-0.38766	8.05E-21	2.6E-16
Abhd6	-0.38811	2.31E-19	7.47E-15
Parvg	-0.38986	7.96E-20	2.57E-15
Ccr5	-0.39195	2.09E-25	6.74E-21
Sgce	-0.39227	3.02E-19	9.76E-15
Gcn1	-0.39257	9.03E-19	2.92E-14
Tmem44	-0.39347	5.28E-35	1.71E-30
Bach1	-0.39402	1.4E-21	4.51E-17
Tmem273	-0.39417	7.49E-19	2.42E-14
Gm4258	-0.39456	4.6E-22	1.48E-17
Kcnip3	-0.39492	9.81E-21	3.17E-16
Gm3468	-0.3969	1.57E-37	5.08E-33
Ptpre	-0.39949	1.25E-18	4.03E-14
Snx18	-0.40017	1.7E-31	5.49E-27
Lrp1	-0.40018	1.12E-27	3.62E-23
Med12l	-0.40026	3.78E-19	1.22E-14
Nkain2	-0.40057	2.65E-42	8.55E-38
Klf3	-0.40082	2.32E-20	7.5E-16
Timp2	-0.401	2.26E-24	7.28E-20
Specc1	-0.4013	2.18E-15	7.03E-11
Nid2	-0.40159	3.97E-38	1.28E-33
Syngr1	-0.40165	3.3E-29	1.06E-24
Saraf	-0.40215	3.19E-34	1.03E-29
Lpar6	-0.40254	2.31E-17	7.46E-13

Dgkd	-0.40312	1.77E-21	5.73E-17
Gnb2	-0.40319	7.46E-24	2.41E-19
Ptpro	-0.40326	6.13E-22	1.98E-17
Sft2d2	-0.40418	3.31E-27	1.07E-22
Tlr5	-0.40501	1.84E-43	5.94E-39
Tmem100	-0.40528	7.02E-23	2.26E-18
Pacc1	-0.4058	4.11E-25	1.33E-20
Gna15	-0.40666	2.57E-27	8.29E-23
Rptor	-0.40708	1.06E-19	3.41E-15
Olfml2b	-0.40767	1.35E-25	4.36E-21
Msrb2	-0.40795	2.35E-30	7.57E-26
Plod3	-0.40936	1.48E-24	4.77E-20
Gabbr1	-0.40957	3.47E-35	1.12E-30
Lyzl4	-0.40981	3.37E-25	1.09E-20
Zfp69	-0.41112	1.31E-21	4.23E-17
Ighm	-0.41295	8.88E-22	2.87E-17
Edem2	-0.41311	1.38E-25	4.46E-21
Mlxipl	-0.41339	8.81E-26	2.84E-21
Cd53	-0.41471	2.25E-41	7.26E-37
Arhgap27	-0.41521	4.83E-24	1.56E-19
Lyl1	-0.41656	9.28E-22	2.99E-17
Elk3	-0.41741	7.69E-15	2.48E-10
Zfp652	-0.41765	6.71E-19	2.17E-14
Ephx1	-0.42034	7.05E-24	2.28E-19
Ints6l	-0.42036	1.09E-19	3.53E-15
Gm2629	-0.4206	6.73E-24	2.17E-19
Exoc6b	-0.4211	1.07E-14	3.44E-10
Fmnl1	-0.42192	5.48E-25	1.77E-20
St6gal1	-0.42273	2.37E-29	7.64E-25
Man1c1	-0.42331	8.6E-21	2.77E-16
Rgs2	-0.42534	2.26E-24	7.3E-20
Phyhd1	-0.42567	2.5E-26	8.08E-22
Gm40841	-0.42579	2.59E-35	8.35E-31
Dock8	-0.4258	6.85E-27	2.21E-22
Myo18b	-0.42648	2.25E-39	7.28E-35
Ubash3b	-0.42681	6.58E-19	2.13E-14
Garnl3	-0.42709	3.18E-17	1.03E-12
Rock2	-0.42819	1.05E-20	3.4E-16
Asap1	-0.42845	1.13E-18	3.66E-14
Lrrc25	-0.42911	1.52E-24	4.9E-20
Plod1	-0.42916	2.66E-26	8.58E-22
Grap	-0.42978	2.98E-24	9.62E-20
G530011O	-0.43001	2.32E-19	7.49E-15
Oxr1	-0.43015	2.96E-15	9.57E-11
Nfia	-0.43101	1.69E-20	5.47E-16
Hfe	-0.43107	1.62E-27	5.21E-23
Pmepa1	-0.43136	2.43E-18	7.84E-14
Nckap1l	-0.43166	1.14E-26	3.68E-22

Snx29	-0.43179	2.91E-23	9.4E-19
Gm33699	-0.43306	1.02E-28	3.29E-24
Ggt5	-0.43407	4.97E-31	1.6E-26
Ivns1abp	-0.43511	3.13E-35	1.01E-30
Csnk1e	-0.43627	1.39E-22	4.48E-18
Ddah2	-0.43866	1.79E-38	5.78E-34
Fli1	-0.43908	1.57E-38	5.08E-34
Myadm	-0.43927	1.81E-24	5.86E-20
Slc46a1	-0.43942	2.63E-20	8.49E-16
Serpinf1	-0.44067	4.44E-26	1.43E-21
Hexb	-0.44243	4.3E-151	1.4E-146
Tspan18	-0.44252	2.42E-31	7.82E-27
Myo1b	-0.44281	5.57E-22	1.8E-17
Ptp4a3	-0.44756	4.02E-23	1.3E-18
Dip2b	-0.44868	9.17E-23	2.96E-18
Abhd12	-0.44884	1.65E-60	5.33E-56
Comtd1	-0.4493	7.8E-27	2.52E-22
Gm34589	-0.45024	6.84E-34	2.21E-29
Dlc1	-0.45103	6.13E-23	1.98E-18
Fmnl3	-0.45165	4.73E-31	1.53E-26
Ppp1r9a	-0.45217	4.55E-22	1.47E-17
Whrn	-0.45225	1.23E-18	3.96E-14
Atp8a2	-0.45293	8.51E-22	2.75E-17
Nptn	-0.4533	1.53E-35	4.95E-31
Hdac9	-0.45344	3.2E-14	1.03E-09
Itga6	-0.45419	1.49E-28	4.82E-24
Numa1	-0.45484	1.07E-24	3.44E-20
Tfe3	-0.4549	1.15E-25	3.71E-21
Zfp706	-0.45526	1.82E-26	5.87E-22
Gas6	-0.45529	6.03E-24	1.95E-19
Slc7a8	-0.45532	1.32E-31	4.27E-27
Gm42047	-0.45834	0.00049	1
Tgfb1	-0.45974	5.23E-37	1.69E-32
Ctsf	-0.46144	7.4E-34	2.39E-29
Thrsp	-0.4617	2.12E-44	6.85E-40
Pwwp2a	-0.46264	1.83E-23	5.9E-19
Tnfrsf21	-0.46309	2.97E-35	9.59E-31
Tgfbr1	-0.46485	1.42E-49	4.59E-45
St3gal5	-0.46504	2E-26	6.45E-22
Gm48099	-0.46605	3.86E-18	1.25E-13
Itgb5	-0.46606	2.09E-42	6.74E-38
Ttc28	-0.46757	3.31E-21	1.07E-16
Adam15	-0.46793	1.47E-32	4.76E-28
Fat3	-0.4708	5.09E-15	1.64E-10
Sqor	-0.47294	8.27E-33	2.67E-28
Sncaip	-0.47401	4.04E-29	1.31E-24
Mtdh	-0.47455	3.19E-60	1.03E-55
Rnf13	-0.47477	2.47E-50	7.99E-46

Prkce	-0.47553	4.93E-15	1.59E-10
Bin2	-0.47582	8.53E-46	2.75E-41
Eif4g2	-0.47622	1.88E-36	6.06E-32
Ifngr1	-0.47727	1.2E-65	3.87E-61
Tmem135	-0.47734	2.31E-34	7.46E-30
Ypel3	-0.4781	9.8E-29	3.16E-24
Lpcat3	-0.48204	2.52E-32	8.15E-28
A830008E2	-0.48239	4.56E-20	1.47E-15
Commd8	-0.4824	1.61E-33	5.19E-29
Ppm1l	-0.48394	1.55E-26	4.99E-22
Gtf2h2	-0.48406	9.62E-39	3.11E-34
Irf2bpl	-0.48487	3.39E-25	1.09E-20
Abl1	-0.48644	2.8E-26	9.05E-22
Rreb1	-0.48783	2.96E-27	9.56E-23
Frmd4a	-0.48874	1.21E-46	3.89E-42
Tuba1a	-0.48888	1.61E-24	5.21E-20
Zdhhc14	-0.48919	8.3E-25	2.68E-20
Eef2k	-0.4893	8.74E-29	2.82E-24
Gm6277	-0.49259	9E-36	2.91E-31
Cep170	-0.49321	1.45E-30	4.69E-26
Rtn1	-0.4933	1.01E-25	3.25E-21
Exoc4	-0.49352	1.59E-24	5.13E-20
Glul	-0.49354	5.13E-57	1.66E-52
Plcl1	-0.4939	3.77E-19	1.22E-14
Magi1	-0.49464	7.34E-15	2.37E-10
Pard3b	-0.49537	1.67E-20	5.38E-16
Asph	-0.4967	7.57E-36	2.44E-31
Khdrbs3	-0.49742	7.75E-44	2.5E-39
Wasf2	-0.49923	1.03E-54	3.32E-50
Pip4k2a	-0.49953	3.75E-35	1.21E-30
1700017B(	-0.50179	8.02E-41	2.59E-36
Smap2	-0.50216	4.73E-48	1.53E-43
AC149090.	-0.50391	2.72E-38	8.79E-34
Kcnk12	-0.50453	1.26E-48	4.07E-44
Eng	-0.50658	9.38E-32	3.03E-27
Ctsd	-0.50734	7.1E-84	2.29E-79
Fry	-0.50934	8.27E-32	2.67E-27
Col27a1	-0.51054	7.6E-33	2.45E-28
Slc12a2	-0.51106	2.06E-28	6.65E-24
Pros1	-0.51161	1.39E-46	4.5E-42
Slc16a6	-0.51241	7.58E-36	2.45E-31
Klhdc8b	-0.51496	7.11E-57	2.29E-52
Mid1ip1	-0.51581	3.51E-39	1.13E-34
Cltc	-0.51604	8.59E-49	2.77E-44
Dhrs3	-0.51713	9.29E-36	3E-31
Upk1b	-0.5179	4.61E-16	1.49E-11
Otulinl	-0.51856	4.63E-60	1.5E-55
Trp53cor1	-0.51921	1.57E-30	5.06E-26

Mef2a	-0.52181	3.42E-69	1.1E-64
Olfml3	-0.52408	9.58E-60	3.09E-55
Rab3il1	-0.52475	7.76E-43	2.5E-38
Kif21b	-0.52573	5.14E-28	1.66E-23
Slc46a3	-0.52684	1.91E-32	6.18E-28
Cyth4	-0.52844	1.78E-75	5.74E-71
Arhgap45	-0.52942	5.53E-52	1.78E-47
Etv5	-0.53021	2.71E-37	8.74E-33
Mtss1	-0.53185	3.99E-35	1.29E-30
Slco2b1	-0.5333	9.52E-63	3.07E-58
Phf14	-0.53334	1.54E-31	4.96E-27
Kcnma1	-0.5336	1.17E-18	3.77E-14
Idh2	-0.53698	6.67E-41	2.15E-36
P3h2	-0.53752	2.62E-30	8.47E-26
Mgll	-0.53764	3.55E-40	1.15E-35
Cep68	-0.53924	1.89E-36	6.12E-32
Cmtm6	-0.53958	2.15E-51	6.94E-47
Cask	-0.54045	1.37E-33	4.41E-29
Txnip	-0.54164	1.34E-49	4.32E-45
Gal3st4	-0.54185	1.79E-37	5.77E-33
Gm10790	-0.54311	3.84E-43	1.24E-38
Evi2a	-0.54329	5.25E-43	1.69E-38
Ctnnd1	-0.54457	6.03E-36	1.95E-31
Pde3b	-0.54498	3.92E-46	1.27E-41
Abcc3	-0.54645	2.99E-36	9.67E-32
Cd300a	-0.54745	4.07E-33	1.31E-28
Mctp1	-0.55148	2.23E-23	7.21E-19
Csf1r	-0.55252	1.1E-164	3.5E-160
F11r	-0.55252	4.91E-72	1.59E-67
Serinc3	-0.55317	2.3E-136	7.3E-132
Rnf167	-0.55849	1.09E-45	3.52E-41
Gpr165	-0.56352	3.58E-63	1.16E-58
Inpp4b	-0.56521	1.92E-64	6.2E-60
Cyfip1	-0.56577	4.12E-85	1.33E-80
Bbs9	-0.56589	3.51E-26	1.13E-21
Epb41l2	-0.56741	2.17E-75	7.01E-71
Gpr155	-0.57133	6.98E-42	2.25E-37
Tmem119	-0.57214	1.2E-86	3.88E-82
Fam110a	-0.5732	3.08E-45	9.95E-41
Itga9	-0.57378	7.07E-32	2.28E-27
2610203C2	-0.57418	2.29E-33	7.38E-29
Ldlrad4	-0.57514	9.48E-43	3.06E-38
Cryl1	-0.57599	6.74E-36	2.18E-31
Mid1	-0.57715	1.27E-34	4.11E-30
Tjp1	-0.57807	6.86E-41	2.21E-36
Jarid2	-0.57849	2.7E-55	8.72E-51
Gm26542	-0.57854	8.8E-29	2.84E-24
Apbb1ip	-0.57904	1.2E-101	3.84E-97

Gpr183	-0.58109	1.15E-52	3.72E-48
Usp2	-0.58352	3.53E-47	1.14E-42
Scamp5	-0.58472	5.82E-41	1.88E-36
Arhgap5	-0.58484	1.37E-64	4.42E-60
Cttnbp2nl	-0.59085	2.75E-61	8.88E-57
Arap3	-0.59153	3.06E-52	9.88E-48
Gp9	-0.59434	3.77E-68	1.22E-63
Camk1	-0.5972	8.66E-64	2.8E-59
Jam2	-0.60253	2.08E-46	6.7E-42
Hexa	-0.60539	4.81E-74	1.55E-69
Pdgfb	-0.60891	1.62E-42	5.22E-38
4933406I1	-0.61493	2.31E-42	7.47E-38
Sirpa	-0.61506	2.3E-102	7.37E-98
Plxnb2	-0.6153	3.06E-50	9.89E-46
Rgs10	-0.61676	5.2E-89	1.68E-84
Slc9a9	-0.61735	1.36E-55	4.38E-51
Nos1ap	-0.61764	6.12E-32	1.97E-27
Chd9	-0.61944	5.07E-85	1.64E-80
Mylip	-0.62078	3.74E-58	1.21E-53
Bin1	-0.6209	2.18E-88	7.02E-84
Sgk1	-0.62103	4.2E-61	1.36E-56
Rasgrp3	-0.62108	9.05E-54	2.92E-49
Gm3739	-0.62219	6.8E-71	2.2E-66
Ptprm	-0.63052	4.44E-23	1.43E-18
Trem2	-0.63184	1.4E-110	4.6E-106
Cd164	-0.64327	5.94E-67	1.92E-62
Scoc	-0.64595	3.05E-54	9.86E-50
0610040J0	-0.64973	4.52E-58	1.46E-53
Klf12	-0.65348	4.16E-37	1.34E-32
Hpgds	-0.65468	1.13E-83	3.66E-79
Rapgef5	-0.65585	1.06E-57	3.43E-53
Adamts16	-0.66022	2.92E-72	9.44E-68
Rnaset2b	-0.66337	1.46E-64	4.71E-60
Qk	-0.66373	2.34E-73	7.54E-69
Gm5086	-0.66738	9.58E-48	3.09E-43
Fhit	-0.67024	6.58E-30	2.12E-25
Cd68	-0.67267	1.16E-95	3.76E-91
Sft2d1	-0.67613	1E-99	3.28E-95
Zfhx3	-0.67789	6.8E-80	2.2E-75
Sall1	-0.68168	2.93E-64	9.45E-60
Ltc4s	-0.68581	1.59E-73	5.14E-69
Plcl2	-0.69481	8.92E-51	2.88E-46
Siglech	-0.70006	1.1E-103	3.7E-99
Tanc2	-0.7043	8.74E-87	2.82E-82
Snn	-0.70525	1.17E-67	3.77E-63
Sdk1	-0.70609	4.5E-21	1.45E-16
Maml3	-0.71037	3.14E-55	1.01E-50
Agmo	-0.71076	3.87E-61	1.25E-56

D830030K:	-0.71493	4.97E-75	1.6E-70
Basp1	-0.71582	4.1E-110	1.3E-105
Arhgef40	-0.71826	8.29E-64	2.68E-59
Mir99ahg	-0.72716	4.03E-67	1.3E-62
Fchsd2	-0.73909	2.02E-62	6.52E-58
Il6st	-0.74065	3.18E-63	1.03E-58
Ckb	-0.7438	1.4E-102	4.58E-98
Plxna4	-0.74644	6.75E-37	2.18E-32
Slc40a1	-0.75423	8.64E-72	2.79E-67
Serpine2	-0.75476	5.89E-97	1.9E-92
St3gal6	-0.75509	6E-99	1.92E-94
Srgap2	-0.75541	5.4E-113	1.8E-108
Abca9	-0.75734	6.69E-89	2.16E-84
Marcks	-0.75991	1E-159	3.3E-155
Rnase4	-0.76942	5.7E-99	1.83E-94
Sparc	-0.77025	6.6E-150	2.1E-145
Cxxc5	-0.77109	1.22E-79	3.93E-75
Fam102b	-0.77149	1.54E-68	4.96E-64
Zfp710	-0.77337	5.25E-55	1.7E-50
Vsir	-0.77507	3.1E-152	1E-147
Prkn	-0.78715	3.6E-51	1.16E-46
Ifi27	-0.80462	2.85E-63	9.2E-59
Susd3	-0.80638	5.62E-96	1.81E-91
Rtn4rl1	-0.81826	8.63E-96	2.79E-91
Tmem176a	-0.81865	7.12E-65	2.3E-60
Calm2	-0.83628	6.6E-106	2.1E-101
Nav3	-0.83824	1.66E-75	5.34E-71
Plxdc2	-0.84679	2.3E-153	7.5E-149
Sox4	-0.85864	2.1E-79	6.79E-75
Elmo1	-0.86367	5.9E-139	1.9E-134
Adgrg1	-0.86368	2.27E-94	7.33E-90
Bank1	-0.87821	1.53E-70	4.93E-66
Pmp22	-0.89087	1.1E-108	3.6E-104
Slc2a5	-0.89236	6.42E-85	2.07E-80
Lrba	-0.89836	7.81E-95	2.52E-90
Ifitm10	-0.90419	4.69E-95	1.51E-90
Selplg	-0.93551	5.6E-227	1.8E-222
Maf	-0.94589	1.3E-122	4.1E-118
Numb	-0.96895	7E-145	2.3E-140
Cfh	-0.97188	8.3E-166	2.7E-161
Arsb	-0.97925	2.7E-112	8.6E-108
Selenop	-0.98951	2.4E-165	7.6E-161
Golm1	-1.00205	1E-166	3.4E-162
Cx3cr1	-1.02735	1.5E-249	4.8E-245
8030442B(	-1.03455	7.9E-111	2.5E-106
P2ry13	-1.04686	7.2E-149	2.3E-144
Gm19951	-1.0733	6.07E-52	1.96E-47
Cst3	-1.16119	1.7E-300	5.6E-296

Tmem176b	-1.20156	7.4E-139	2.4E-134
Ecscr	-1.21566	1.7E-157	5.6E-153
Nav2	-1.24292	2.6E-138	8.4E-134
Hpgd	-1.25788	1.7E-169	5.4E-165
Gpr34	-1.46005	3E-243	9.8E-239
Fscn1	-1.49179	1.9E-213	6.2E-209
P2ry12	-1.5932	0	0
Csmd3	-1.63133	8.3E-158	2.7E-153
Fah	-1.64448	4.3E-300	1.4E-295
Ttr	-1.74041	2.44E-32	7.88E-28
Fcrls	-2.35806	0	0

SUPPLEMENTAL TABLE 4. Differentially Regulated Genes in WT versus CB2R$^{-/-}$ GVHD Microglia

Gene	avg_log2FC	p_val	p_val_adj
Gm47283	0.523913	7.8E-67	2.52E-62
Mir142hg	0.408831	1.58E-54	5.1E-50
Zfp36	0.406314	1.73E-38	5.6E-34
G530011O06Rik	0.40226	2.34E-59	7.56E-55
H3f3b	0.400692	1.16E-48	3.73E-44
Uba52	0.368005	1.34E-65	4.33E-61
Atf3	0.364562	6.72E-22	2.17E-17
Jund	0.352425	4.04E-38	1.3E-33
Cst3	0.349709	1.4E-72	4.53E-68
Fos	0.349141	2.25E-16	7.26E-12
Hist1h1e	0.344081	3.96E-43	1.28E-38
Gm10076	0.325133	4.46E-48	1.44E-43
Zfp36l1	0.314942	1.66E-16	5.37E-12
Gm34455	0.308338	4.28E-28	1.38E-23
Jun	0.304113	3.04E-20	9.81E-16
Hist1h2bc	0.299136	4.31E-13	1.39E-08
Ccl4	0.298441	4.81E-08	0.001551
Zfand5	0.277478	2.85E-21	9.21E-17
Hist1h2ac	0.260357	3.69E-20	1.19E-15
Hist1h1c	0.259677	4.76E-30	1.54E-25
Olfml3	0.258959	2.86E-25	9.25E-21
Ltc4s	0.257464	4.36E-18	1.41E-13
Timp2	0.249441	2.45E-26	7.9E-22
Btg2	0.248186	3.9E-10	1.26E-05
Tnf	0.24434	5.98E-12	1.93E-07
Cd63	0.244056	2.49E-14	8.03E-10
Ccl5	0.239295	0.900621	1
Gpr34	0.235808	7.14E-20	2.3E-15
Tyrobp	0.229426	6.24E-40	2.01E-35
mt-Nd4l	0.227318	2.13E-20	6.87E-16
Fosb	0.223403	4.91E-23	1.58E-18
Trem2	0.222643	5.86E-33	1.89E-28
Mid1	0.221497	2.95E-26	9.52E-22
Nfkbiz	0.217497	2.14E-11	6.9E-07
Ctsd	0.216533	1.8E-22	5.81E-18
Cd9	0.212324	7.28E-23	2.35E-18
Ccl3	0.211851	1.21E-09	3.92E-05
Fcrls	0.208982	1.29E-17	4.18E-13
Siglech	0.208899	7.08E-21	2.29E-16
Rgs1	0.205949	2.26E-07	0.007309
Lyz2	0.20521	4.23E-15	1.37E-10
Tmx4	0.203751	4.99E-10	1.61E-05
Ier5	0.202569	5.29E-18	1.71E-13

Sqstm1	0.198842	6.39E-15	2.06E-10
Selenop	0.197911	7.11E-15	2.3E-10
Hexb	0.197257	1.77E-52	5.72E-48
mt-Nd5	0.196784	2.79E-20	9.02E-16
2810013P06Rik	0.196661	7.97E-21	2.57E-16
Ddx3x	0.195149	1.41E-09	4.55E-05
Fcer1g	0.19499	7.03E-29	2.27E-24
Il1a	0.194291	2.95E-10	9.52E-06
C1qb	0.194129	5.04E-39	1.63E-34
Junb	0.193173	1.04E-09	3.36E-05
Btg1	0.19146	2.25E-14	7.26E-10
Pmepa1	0.184971	1.31E-15	4.22E-11
E330020D12Rik	0.184363	1.06E-07	0.003412
Rhob	0.184118	2.78E-13	8.97E-09
Rpl35	0.183194	7E-19	2.26E-14
Hsp90aa1	0.182547	1.19E-12	3.84E-08
Apoe	0.182246	1.46E-06	0.047062
Rpl37a	0.181229	5.31E-25	1.72E-20
Prkg1	0.181191	4.38E-06	0.141529
Rgs2	0.177521	7.57E-10	2.44E-05
Itm2b	0.176651	4.14E-32	1.34E-27
Mafb	0.176199	3.37E-13	1.09E-08
Hist1h2ap	0.174716	2.77E-18	8.94E-14
Tpt1	0.174694	1.19E-31	3.83E-27
Egr1	0.174648	0.025553	1
Cd68	0.174196	2.7E-14	8.72E-10
Tent5c	0.174046	3.05E-12	9.85E-08
Fam102b	0.173482	1.4E-12	4.53E-08
Rps27	0.172116	9.8E-20	3.16E-15
Plek	0.171905	4.83E-13	1.56E-08
Kcnma1	0.171196	4.07E-11	1.31E-06
Rpl36a	0.170757	5.28E-18	1.7E-13
Ccl12	0.169747	0.0004	1
Prxl2c	0.169734	9.81E-07	0.03166
Rps21	0.169407	3.03E-22	9.77E-18
Rps15a	0.168202	3.49E-22	1.13E-17
Sgk1	0.16377	1.13E-05	0.365475
Ctsl	0.163008	2.52E-19	8.13E-15
Glul	0.16101	0.034026	1
Cd83	0.160681	0.002593	1
Creg1	0.160357	4.97E-16	1.6E-11
Actg1	0.159525	4.5E-13	1.45E-08
Ctsz	0.158701	1.55E-21	5.02E-17
mt-Nd2	0.155615	6.03E-14	1.95E-09
Gnas	0.154175	2.84E-10	9.18E-06
Cx3cr1	0.15398	2.4E-17	7.74E-13
Ctsb	0.153554	9.38E-13	3.03E-08
Selenok	0.15283	1.9E-14	6.13E-10

Rpl39	0.152724	6.95E-20	2.24E-15
mt-Cytb	0.151846	6.57E-14	2.12E-09
Rasgef1b	0.151775	1.06E-10	3.41E-06
Rpl30	0.151622	6.78E-20	2.19E-15
Cd81	0.150558	2.75E-17	8.89E-13
Ubb	0.150413	2.09E-17	6.76E-13
Ppib	0.149814	2.34E-12	7.57E-08
Lgmn	0.148436	9E-26	2.9E-21
Fau	0.148144	8.82E-19	2.85E-14
Rps19	0.147252	3.96E-17	1.28E-12
Sparc	0.147164	7.05E-12	2.28E-07
Actb	0.145372	2.18E-13	7.02E-09
Ssr4	0.144988	3.91E-13	1.26E-08
Ighm	0.144269	1.73E-10	5.58E-06
Ckb	0.144237	1.82E-11	5.86E-07
Gas6	0.143671	1.69E-07	0.005468
Syngr1	0.143598	3.78E-09	0.000122
mt-Nd4	0.143475	7.77E-15	2.51E-10
Hist1h3d	0.143372	5.24E-17	1.69E-12
Gm15726	0.142472	3.73E-17	1.2E-12
Arl6ip1	0.141943	2.16E-11	6.96E-07
Ptma	0.141514	2E-18	6.45E-14
Rplp1	0.140916	1.41E-18	4.55E-14
Gpr183	0.140771	8.88E-06	0.286711
Cxcl9	0.140579	0.924126	1
Lamp1	0.139146	4.71E-14	1.52E-09
C1qc	0.138154	6.44E-21	2.08E-16
Npc2	0.137632	7E-15	2.26E-10
P2ry12	0.137234	4.23E-11	1.36E-06
Rpl13	0.136625	8.31E-16	2.68E-11
Smad7	0.136515	2.64E-08	0.000854
Atp8a2	0.136323	8.76E-10	2.83E-05
Rps12	0.136218	1.27E-17	4.09E-13
Ubc	0.13599	1.34E-16	4.31E-12
Fcgr3	0.135367	2.03E-09	6.57E-05
Abhd12	0.134656	6.13E-12	1.98E-07
Hsp90b1	0.134425	1.06E-10	3.42E-06
Rnase4	0.13404	1.68E-09	5.42E-05
Nav3	0.133347	3.01E-06	0.097334
St3gal6	0.133325	1.39E-07	0.004473
Rpl36	0.132634	9.59E-13	3.1E-08
Cap1	0.132049	1.3E-13	4.18E-09
Rps24	0.131813	1.97E-16	6.36E-12
Rpl11	0.131259	2.11E-14	6.82E-10
Zeb2os	0.130648	1.17E-09	3.79E-05
Tmem258	0.130315	1.28E-09	4.14E-05
Hist1h1d	0.13006	4.64E-15	1.5E-10
Rps26	0.129907	2.18E-11	7.05E-07

Kctd12	0.129393	1.96E-11	6.32E-07
Bhlhe41	0.128491	6.53E-09	0.000211
4933421O10Rik	0.127689	3.7E-12	1.2E-07
Hmgb2	0.126986	2.23E-10	7.21E-06
Rnasek	0.126826	2.27E-07	0.007344
Rpl34	0.126473	3.24E-14	1.05E-09
Pfdn5	0.126125	2.85E-11	9.21E-07
Ptms	0.126108	0.000398	1
Klf4	0.12597	8.67E-11	2.8E-06
Rps7	0.12559	2.15E-14	6.93E-10
Socs3	0.125497	1.12E-07	0.003615
Manf	0.124632	3.2E-08	0.001032
Basp1	0.124396	2.74E-08	0.000883
Klf2	0.124022	1.23E-06	0.039859
Fat3	0.123855	0.010692	1
Rpl23a	0.12382	2.29E-11	7.4E-07
mt-Nd3	0.123463	2.68E-10	8.67E-06
Bsg	0.123325	1.69E-10	5.45E-06
Slc15a3	0.123317	0.000147	1
Arhgap22	0.123019	1.17E-08	0.000379
Efhd2	0.122803	9.06E-07	0.029256
Ifitm10	0.122246	3.12E-05	1
Tmed10	0.122052	1.29E-09	4.18E-05
Rps4x	0.121723	2.95E-12	9.52E-08
Hnrnpa3	0.121677	1.63E-10	5.26E-06
Arl4c	0.121542	9E-08	0.002905
Cd52	0.121495	4.94E-07	0.015953
Hist1h2ae	0.121321	1.66E-11	5.37E-07
Cyba	0.12117	1.35E-10	4.37E-06
Pdia3	0.120943	1.48E-09	4.78E-05
Gng5	0.120717	4.04E-10	1.31E-05
Slamf9	0.120547	1.67E-05	0.540209
Pdia6	0.120139	4.08E-10	1.32E-05
Pdgfb	0.119987	1.11E-06	0.035679
Rps3	0.119738	6.17E-13	1.99E-08
mt-Atp8	0.119336	6.01E-08	0.001941
Rpl28	0.119198	2.69E-10	8.69E-06
Rpl17	0.119181	2.79E-12	9E-08
Cmtm6	0.119015	1.11E-08	0.000357
Klhl6	0.118908	1.47E-05	0.474291
Dnajb9	0.118665	7.05E-09	0.000228
Tanc2	0.118032	8.32E-11	2.69E-06
Atp6v0c	0.117983	4.81E-07	0.015531
Ccr5	0.11698	0.000852	1
Rpl27a	0.116561	1.22E-12	3.94E-08
Naca	0.116377	2.11E-09	6.81E-05
Ndufa13	0.115269	2.35E-08	0.000758
Hist1h4d	0.115073	2.82E-08	0.000911

Rpl37	0.114835	6.06E-12	1.96E-07
Selenos	0.114525	9.43E-08	0.003044
mt-Nd1	0.114216	1.02E-10	3.3E-06
Rps27a	0.11419	6.58E-12	2.13E-07
Dad1	0.113702	5.55E-09	0.000179
4930557K07Rik	0.11353	0.439794	1
Calm2	0.113306	7.52E-09	0.000243
Kif21b	0.11287	3.91E-07	0.012639
Rpl35a	0.112262	6.25E-12	2.02E-07
Wsb1	0.112076	5.3E-08	0.001711
Rps5	0.111944	1.62E-10	5.24E-06
Ppp1r15a	0.111827	3.05E-11	9.83E-07
Mrpl52	0.111652	8.33E-07	0.026893
Rpl7	0.111565	1.5E-11	4.85E-07
Uqcrb	0.111246	5.47E-08	0.001766
Tmem50a	0.111185	5.14E-08	0.001659
Mettl23	0.110793	2.13E-10	6.87E-06
Whrn	0.110719	5.07E-08	0.001636
Rpl32	0.110507	2.09E-12	6.75E-08
Rpl26	0.110361	1.11E-11	3.59E-07
Sft2d1	0.11019	6.85E-09	0.000221
Fth1	0.110189	1.57E-07	0.005076
Rpl21	0.110003	2.14E-13	6.9E-09
Rplp2	0.10995	1.08E-12	3.49E-08
Mylip	0.109517	1.15E-06	0.037285
Rps16	0.108244	1.41E-10	4.56E-06
Rps20	0.10821	5.8E-11	1.87E-06
Tmed3	0.108078	2.46E-09	7.94E-05
Rpl22	0.108001	3.92E-08	0.001265
Mrc2	0.107836	9.24E-05	1
Cxcl16	0.107023	0.000862	1
Dpm3	0.106561	3.02E-09	9.75E-05
Rps9	0.106486	1.46E-11	4.73E-07
Clta	0.10635	4.07E-10	1.31E-05
Golm1	0.106287	1.77E-07	0.005702
A930007I19Rik	0.106011	0.000179	1
Cotl1	0.105955	0.000486	1
Ywhaz	0.105789	2.24E-06	0.072432
Plxna4	0.105247	0.000123	1
Calr	0.105194	1.31E-07	0.004229
Rps3a1	0.104946	3.55E-10	1.15E-05
Sec11c	0.1048	4.31E-07	0.013926
Ostc	0.104649	2.37E-07	0.007641
Rpl9	0.104501	2.21E-09	7.15E-05
Krtcap2	0.104423	9.73E-07	0.031407
Lpl	0.104295	0.020055	1
Pmp22	0.104282	5.15E-06	0.166319
Nr6a1os	0.104178	0.001902	1

Rps15	0.103435	3.61E-08	0.001165
Rpl23	0.103422	2.03E-12	6.54E-08
Nr1d2	0.103	5.14E-10	1.66E-05
Rps6	0.102999	2.62E-09	8.45E-05
Gm11867	0.102236	1.12E-18	3.63E-14
Rps13	0.102069	1.85E-09	5.98E-05
Rpl6	0.101866	1.1E-07	0.003567
Rpl22l1	0.10148	1.77E-07	0.005721
Rps14	0.101089	4.72E-09	0.000152
Ecscr	0.100892	5.95E-08	0.001921
Rpl19	0.100774	4E-11	1.29E-06
Rplp0	0.100742	1.88E-10	6.07E-06
Fkbp2	0.099716	3.13E-06	0.1012
mt-Co2	0.099689	2.46E-09	7.95E-05
2410006H16Rik	0.099204	1.27E-05	0.410404
Rpl24	0.09916	1.55E-09	5.02E-05
Cox4i1	0.098971	9.65E-08	0.003115
Ctso	0.098469	1.19E-05	0.384564
Tmsb4x	0.098087	4.97E-12	1.61E-07
Gm34921	0.097858	1.1E-11	3.56E-07
Rps25	0.097836	1.51E-09	4.87E-05
Slc3a2	0.097306	7.13E-07	0.023031
Ncl	0.09704	1.44E-05	0.463775
Ctsf	0.09686	6.66E-07	0.021495
Itm2c	0.096596	2.36E-07	0.007607
Nptn	0.096587	4.14E-06	0.133821
Cd300c2	0.096542	5.42E-08	0.00175
Eif1	0.09645	3.7E-07	0.011938
Sox4	0.09636	0.000337	1
Rps8	0.095922	6.75E-08	0.002181
Ndufc2	0.095864	1.94E-05	0.625987
Eef1a1	0.095839	4.15E-09	0.000134
Spcs2	0.095535	2.28E-07	0.007364
Eif5	0.094932	2.64E-06	0.085237
Kpna4	0.094873	1.94E-05	0.626363
Srgn	0.094817	3.61E-06	0.116653
Dnajc3	0.094776	7.75E-07	0.025017
Id2	0.094758	0.005033	1
Rpl38	0.094715	5.85E-09	0.000189
Chsy1	0.094479	2.63E-05	0.849202
Prkn	0.094456	0.009256	1
Tpp1	0.094379	2.13E-07	0.006877
Rpl8	0.094322	3.53E-09	0.000114
Qk	0.094153	1.02E-06	0.032812
Rpl18	0.094091	8.64E-08	0.002789
Cd164	0.09396	2.19E-05	0.707319
Bst2	0.093945	5.1E-05	1
Rpl5	0.093933	2.81E-08	0.000908

Ndufa6	0.093416	6.7E-05	1
Atraid	0.093145	7.84E-06	0.25303
Ifrd1	0.092795	5.64E-07	0.018218
Rps11	0.092538	7.86E-10	2.54E-05
Zfp706	0.092511	3.74E-06	0.120766
Hexa	0.091318	8.82E-06	0.284857
Surf4	0.091267	9.5E-05	1
Ociad1	0.090972	2.79E-06	0.090093
Tgfbr1	0.090802	4.77E-06	0.153904
mt-Atp6	0.090718	1.14E-08	0.000369
Gem	0.090191	2.67E-10	8.6E-06
Gm26887	0.090166	1.09E-18	3.53E-14
Rpl12	0.08997	2.47E-09	7.98E-05
Sdf2l1	0.089891	2.94E-06	0.094826
Snrpg	0.089519	2.2E-06	0.071033
Klk8	0.088941	8.96E-07	0.028924
Tmem37	0.088926	3.36E-06	0.108488
Tex14	0.088888	0.000487	1
C1qa	0.088839	1.62E-10	5.23E-06
Psenen	0.088797	1.29E-07	0.004151
Arf4	0.088245	0.001363	1
Plk3	0.088048	0.000707	1
Trmt10a	0.087861	3.05E-07	0.00984
Rpl27	0.087721	1.55E-07	0.005019
Dusp1	0.086834	0.000179	1
Rps23	0.086766	4.47E-10	1.44E-05
Ccl9	0.086625	8.8E-06	0.284186
Mef2c	0.085639	3.34E-05	1
Oaz1	0.085458	7.79E-07	0.025155
Rpl7a	0.085442	1.31E-05	0.422704
Rps29	0.085344	1.57E-09	5.07E-05
Il1b	0.085162	0.052924	1
Tmco1	0.085035	0.00016	1
Rpl10	0.084944	1.59E-07	0.005146
Hspa5	0.084768	4.82E-06	0.155486
Rps10	0.08435	2.05E-08	0.000662
Impact	0.084153	0.000131	1
Rack1	0.083705	4.32E-07	0.013937
Tma7	0.083515	0.000201	1
Laptm5	0.08347	1.38E-08	0.000446
Rps28	0.083425	9.35E-08	0.003017
Shisa5	0.083352	0.000692	1
App	0.083288	0.000381	1
Mtch1	0.083006	5.95E-05	1
5033421B08Rik	0.082976	0.000286	1
Pard3b	0.082863	0.085578	1
Gm2629	0.08277	0.000651	1
H1f0	0.082672	0.002318	1

Hnrnpk	0.082488	1.29E-05	0.415836
Nars	0.081994	0.000135	1
Ube2j1	0.081687	2.77E-05	0.894899
Calm1	0.080933	3.91E-05	1
Pnrc1	0.080891	6.1E-06	0.197053
Eif3k	0.080878	0.000439	1
Cox6b1	0.080568	3.83E-06	0.123718
B2m	0.080426	1.45E-07	0.004686
Cd14	0.080137	0.000415	1
Tmed9	0.080083	2.04E-05	0.657258
Mtmr2	0.080039	0.000509	1
Mrfap1	0.079789	5.46E-05	1
Rpl15	0.079714	2.19E-05	0.706802
Cuta	0.079615	7.27E-06	0.234616
Snhg8	0.079595	3.06E-05	0.988977
Bcas2	0.079582	2.05E-05	0.660668
Mt1	0.079402	0.000499	1
Gal3st4	0.078945	0.001364	1
Tmem176b	0.078878	0.01657	1
Edem1	0.078854	9.27E-06	0.299178
Gabarap	0.078161	6.67E-06	0.215237
Rpl29	0.07815	6.05E-07	0.019528
Rpl10a	0.078108	5.91E-06	0.190658
Ninj1	0.077927	2.48E-05	0.800379
Selenof	0.077893	1.24E-05	0.399504
Alkbh1	0.077507	0.000322	1
Ang	0.077457	0.000475	1
Rpl18a	0.077371	1.06E-07	0.003425
mt-Co3	0.077302	5.28E-07	0.017047
Uqcrh	0.077234	4.48E-05	1
Itgam	0.077214	0.000137	1
1700023H06Rik	0.077197	6.88E-12	2.22E-07
Adrb1	0.077122	0.00303	1
Snhg12	0.077036	0.000265	1
Sdf4	0.076449	1.11E-05	0.358661
Saraf	0.07631	4.04E-06	0.130468
Mat2a	0.0762	0.007383	1
Reep5	0.075673	1.31E-05	0.422373
Dmac2l	0.075631	0.418353	1
Rpl41	0.075304	6.42E-05	1
Irf9	0.075209	0.197684	1
Atp5e	0.075097	0.00037	1
Syngr2	0.074905	2.82E-05	0.910567
Ptpro	0.074831	0.007219	1
Rasgrp3	0.074789	0.031721	1
Tmem119	0.074775	2.83E-05	0.912635
Rabac1	0.074396	1.43E-05	0.460854
Rpl13a	0.074395	3.8E-07	0.012264

Nbea	0.074329	0.002872	1
2010013B24Rik	0.074027	5.03E-07	0.01625
Sec61g	0.073926	1.97E-06	0.063759
Wdr83os	0.073917	1.94E-05	0.626988
Immp2l	0.073824	0.016535	1
Ctsa	0.073789	2.84E-05	0.915588
Gngt2	0.073759	1.45E-05	0.466864
Dgkd	0.073604	0.00489	1
Glmp	0.073504	2.32E-06	0.07495
Dusp6	0.073478	0.004062	1
Spint1	0.073181	0.000616	1
Gde1	0.072885	0.00053	1
Tm2d2	0.072857	0.000174	1
Ftl1	0.072532	0.001612	1
Npm1	0.07234	1.47E-05	0.474136
Ccdc134	0.072319	1.29E-05	0.416234
Sall3	0.072163	0.001168	1
Atp5g2	0.072023	9.3E-05	1
Lgals3	0.071914	0.713169	1
Tmem208	0.071654	0.00015	1
Eef1d	0.071586	0.000402	1
Srsf2	0.071495	0.00029	1
Nsa2	0.070736	0.00269	1
Atp6v0b	0.070468	0.000158	1
Hist1h2be	0.07009	0.000236	1
Mctp1	0.07005	0.021703	1
Aif1	0.070039	5.77E-05	1
mt-Co1	0.069996	2.34E-05	0.755484
Adgrg1	0.069774	0.011028	1
Abi3	0.069706	0.001387	1
Khdrbs3	0.069646	2.36E-06	0.076349
Pde3b	0.069642	0.000584	1
Plekhf2	0.069622	0.002456	1
Trps1	0.069539	0.128241	1
Gla	0.069467	2.75E-05	0.888896
Bcap31	0.069376	9.27E-05	1
Tmed2	0.069171	5.57E-07	0.017997
F11r	0.069161	0.005669	1
Scand1	0.068882	0.000331	1
Ctsh	0.068806	1.23E-05	0.395577
Hspe1	0.068721	0.000544	1
Rpl14	0.068453	3.18E-05	1
Ifitm3	0.068177	0.019033	1
Pcna	0.068081	8.7E-09	0.000281
Son	0.068022	4.06E-06	0.131156
Ndufb1-ps	0.067867	0.002139	1
Rpn2	0.067851	0.000106	1
Atp5a1	0.067813	0.000194	1

Arrdc3	0.067738	5.89E-06	0.19003
Vsir	0.067676	0.001734	1
Med22	0.067613	4.27E-06	0.137909
Hsp90ab1	0.067605	1.16E-05	0.375964
Plpbp	0.067479	0.000258	1
Ssr3	0.067446	0.001678	1
Tcn2	0.067398	0.000533	1
Ndufa4	0.067271	1.38E-05	0.446487
Herpud1	0.067268	0.001202	1
Rer1	0.067228	0.000488	1
Sept7	0.067119	0.000213	1
Canx	0.067047	0.000521	1
Gatm	0.066947	0.054951	1
H2-Q7	0.066845	0.00073	1
Fhad1	0.066722	2.78E-05	0.897119
Rnf19b	0.066611	0.941157	1
Aup1	0.066534	0.001762	1
1810024B03Rik	0.066525	0.010484	1
Dnajb11	0.066371	0.000263	1
Pdhb	0.066237	0.003883	1
Fam168b	0.066181	3.25E-05	1
Itgb1	0.066082	0.000261	1
Skil	0.066042	0.000115	1
Btaf1	0.066041	0.001694	1
Bach2	0.066015	0.049804	1
Tmed7	0.065746	0.003694	1
Pycard	0.065733	0.000435	1
Higd2a	0.065441	0.000491	1
Sar1b	0.065425	0.001545	1
Mydgf	0.065377	0.00052	1
Btf3	0.065311	0.000494	1
Samsn1	0.065279	0.000218	1
Jam2	0.065211	7.91E-05	1
Dnase2a	0.065137	0.000103	1
Capza2	0.06511	0.000395	1
Zfp202	0.064816	0.009189	1
Lst1	0.064619	0.000175	1
Slc9a9	0.064439	0.007718	1
Chst8	0.06419	0.001036	1
Tex261	0.064122	0.014495	1
Txnl1	0.064006	0.004396	1
AI506816	0.063679	0.002735	1
Ier2	0.063578	0.190469	1
Eef1b2	0.063479	6.39E-06	0.206156
Daglb	0.063472	0.003425	1
Fmnl3	0.063434	0.002057	1
Sirpa	0.063404	0.001623	1
Slc7a8	0.063355	0.00212	1

Atp1b3	0.063289	0.000735	1
Cd180	0.063201	0.102112	1
Ldlr	0.063169	0.03592	1
Rpl4	0.063062	0.00019	1
Hfe	0.063054	0.000247	1
Cbfa2t3	0.063033	7.42E-05	1
Hdac8	0.062971	0.094565	1
Tmem86a	0.062622	0.000616	1
Tmem256	0.062614	0.003332	1
Magi1	0.062494	0.098641	1
Taf7	0.06226	0.000736	1
Ifngr2	0.062194	0.002808	1
Thoc7	0.062093	0.002523	1
Lrpap1	0.062063	0.000822	1
Cnpy2	0.062055	0.000549	1
Rbfox1	0.061862	0.0487	1
Atp5d	0.061827	0.000959	1
Cables1	0.061598	0.004428	1
Hnrnpdl	0.061591	0.000265	1
Rpl3	0.061341	1.72E-06	0.055456
Man1a2	0.061339	0.000179	1
Slc25a5	0.061287	0.000563	1
Ywhab	0.061065	0.007305	1
Emc7	0.060999	0.002509	1
Zfp827	0.060855	2.46E-06	0.079404
Clic1	0.060815	0.000112	1
Tspan31	0.060786	0.003445	1
Gna15	0.060774	0.002268	1
Laptm4a	0.060728	0.000155	1
Ero1l	0.060627	0.001349	1
Evi2a	0.060387	0.007862	1
Rps27l	0.060278	0.000214	1
Glrp1	0.060201	0.06279	1
Vkorc1	0.060167	0.001497	1
Fcgr2b	0.06009	0.004098	1
Tomm7	0.060061	0.000533	1
Mrc1	0.059979	0.015425	1
Tmed5	0.059738	0.000281	1
Bmyc	0.059458	0.017644	1
Lyzl4	0.059375	0.012499	1
Ssr2	0.059356	0.001019	1
Alg5	0.05933	0.001121	1
Slc44a1	0.059297	0.007167	1
Ndufb3	0.059179	0.008745	1
Eif2b4	0.059169	0.038502	1
9530068E07Rik	0.059133	0.000356	1
Ggh	0.058799	0.000496	1
Gfer	0.05871	0.0034	1

Cnbp	0.05867	0.000464	1
Ago1	0.058617	0.002388	1
Cnih4	0.058581	0.000187	1
Rgs10	0.058577	0.004114	1
Rrp1	0.058536	0.003079	1
Nkap	0.058344	0.003255	1
Gm26542	0.058257	0.038883	1
Tmbim4	0.058246	0.00688	1
Gnl3	0.058174	0.000481	1
Edem2	0.05804	0.017463	1
Ppp1r10	0.058036	0.001667	1
Saysd1	0.057972	0.001534	1
Cyc1	0.057955	0.003625	1
Igf1r	0.057873	0.002775	1
Odc1	0.057781	7.17E-06	0.231451
Sept9	0.057736	9.85E-06	0.318064
Gfm2	0.057567	0.042564	1
Cacnb2	0.057531	0.200009	1
Irf2bpl	0.057465	0.000415	1
Gm37168	0.057241	7.3E-05	1
Chd3	0.057163	0.007652	1
Zfp869	0.057083	0.001391	1
Tnrc6c	0.05693	0.008739	1
Ell2	0.056924	0.041212	1
Ldhb	0.056912	0.000642	1
Emc6	0.056906	0.001038	1
Cst7	0.056689	0.756047	1
Lpxn	0.056626	0.016679	1
B3gat3	0.05661	0.001732	1
Snx17	0.056533	0.006326	1
Emc10	0.056436	0.014565	1
Cox6c	0.056428	0.000268	1
Naa35	0.056368	0.015894	1
Slc7a5	0.056326	0.000612	1
Pdgfa	0.056324	0.020499	1
Ank2	0.056168	0.008792	1
Mcfd2	0.056097	0.000231	1
Serinc1	0.055972	0.00256	1
Klf12	0.055746	0.065097	1
Aurkaip1	0.055668	0.044722	1
Ybx1	0.055604	0.009186	1
Hscb	0.055601	0.000319	1
Rel	0.05553	0.20745	1
Ppp1r2	0.055429	0.000979	1
Atp5f1	0.055324	0.003515	1
Gcc2	0.055261	0.011967	1
Ehd4	0.055208	0.0429	1
Hcst	0.055103	0.000151	1

Zfp318	0.055067	0.022125	1
Ergic3	0.054912	0.001265	1
Ddx50	0.054857	0.011799	1
Wdr36	0.054749	0.010632	1
Bin2	0.054574	0.000245	1
Cd53	0.054501	0.000236	1
Tef	0.054439	0.016896	1
H3f3a	0.054409	4.71E-05	1
Rhoa	0.054353	0.000516	1
Clptm1l	0.054343	0.000624	1
Spty2d1	0.054234	0.015956	1
Gm12703	0.054177	7.35E-06	0.237451
Nrbf2	0.054023	0.004364	1
Agmo	0.05393	0.13562	1
Vkorc1l1	0.053823	0.114548	1
Ptpn18	0.053673	0.003674	1
Arhgdia	0.053604	0.007064	1
Tmem100	0.053548	0.000268	1
Dazap2	0.053482	0.022271	1
Snrpf	0.053405	0.005615	1
Csnk1e	0.053365	0.053262	1
Serpine2	0.053295	0.002977	1
Gspt1	0.053285	0.015675	1
Il21r	0.05321	0.057139	1
Ndufb11	0.053026	0.001703	1
Sall1	0.052946	0.013924	1
Pttg1ip	0.05288	0.003635	1
Maml3	0.052843	0.009901	1
Pet100	0.052819	0.000677	1
Nagpa	0.052803	0.007317	1
Pin4	0.05278	0.031546	1
Slc25a4	0.052764	0.042061	1
Fgf13	0.052644	0.256882	1
Tmem44	0.052641	0.018047	1
Atp5g1	0.052641	0.002846	1
Cass4	0.052558	0.205635	1
Spcs1	0.052504	0.000246	1
Cox5b	0.052503	0.000189	1
Abca1	0.052462	0.107153	1
Hist1h2bg	0.052434	3.77E-06	0.121748
Rpl7l1	0.052392	0.004335	1
Cmtm3	0.052243	0.013874	1
Ly6e	0.052095	0.003466	1
H2-D1	0.052038	0.000282	1
Atp5h	0.052034	0.031483	1
Neat1	0.051952	0.024525	1
Tecr	0.051932	0.006454	1
Sys1	0.05193	0.000417	1

Sem1	0.051866	0.001917	1
Ptgs1	0.051762	0.005982	1
Mfap3	0.051632	0.002591	1
Ranbp1	0.051398	0.000396	1
Pdia4	0.051292	0.010733	1
Adprh	0.051061	0.010772	1
Gm44577	0.051013	0.000513	1
Camk1	0.050907	0.003316	1
Slc37a2	0.050851	0.102074	1
Trf	0.050811	0.000295	1
Klf10	0.050437	0.000337	1
Eif3e	0.050364	0.006659	1
Tmem14c	0.050338	0.000283	1
Arhgap32	0.050332	0.09243	1
Slc39a7	0.050277	0.003661	1
Cd86	0.050216	0.001405	1
Morf4l1	0.05006	0.006622	1
Ccdc107	0.050048	0.000236	1
Tmem59	0.050046	0.006375	1
Acadl	0.050016	0.015178	1
Anp32b	0.050004	0.005784	1
M6pr	0.049882	0.004789	1
Gm2a	0.049771	0.030246	1
Ddrgk1	0.049754	0.006507	1
Arpc2	0.04974	0.116151	1
Lcmt1	0.049708	0.028517	1
Sec62	0.049675	0.001445	1
Tnfaip8l2	0.049624	0.012053	1
Selplg	0.0496	0.017768	1
Coq10b	0.049586	0.021176	1
Bax	0.049408	0.002565	1
Ywhah	0.049278	0.000717	1
Fads1	0.049245	0.023151	1
Hnrnph2	0.049239	0.001205	1
D630023F18Rik	0.049202	1.93E-05	0.621986
Ddost	0.049199	0.000802	1
Lmo2	0.04918	0.042204	1
Plau	0.049119	0.005207	1
Hnrnpa2b1	0.049109	0.047814	1
Tmem30a	0.049087	0.009323	1
Eif4a1	0.049045	0.002185	1
Rpsa	0.048948	0.004162	1
Kat7	0.048869	0.039342	1
Hist1h4c	0.048836	1.17E-06	0.037897
Arpc4	0.048773	0.112581	1
Etf1	0.048766	0.017529	1
Lsm6	0.048711	0.081703	1
Amfr	0.048667	0.006846	1

Fkbp7	0.048636	6.98E-05	1
Serpinf1	0.048585	0.025963	1
Fam173a	0.048568	0.0026	1
Derl1	0.048539	0.000844	1
Slc38a10	0.04842	0.028106	1
Uqcrfs1	0.048259	0.018863	1
Khk	0.048257	0.008905	1
Marcks	0.048257	0.004684	1
Baiap2	0.048226	0.049083	1
Ndfip2	0.048204	0.010219	1
Otud1	0.048051	0.017108	1
Nenf	0.048033	0.00295	1
Ssr1	0.047934	0.012101	1
Cd72	0.047815	0.869463	1
8030442B05Rik	0.047797	0.019222	1
Ncln	0.047702	0.133989	1
Pmaip1	0.047701	0.006125	1
Naa20	0.047688	0.153854	1
Pold4	0.047607	0.008445	1
Cebpzos	0.047515	0.003935	1
Slc25a25	0.047435	0.115744	1
Slc16a6	0.047333	0.134097	1
Ddx18	0.047255	0.004551	1
Terf1	0.047204	0.061207	1
Cldnd1	0.047019	0.002708	1
Sema4d	0.04697	0.066233	1
Larp7	0.046965	0.002168	1
Cask	0.046961	0.053602	1
Eef2	0.046953	0.012961	1
Cpd	0.046915	0.185769	1
Tgoln1	0.046838	0.005824	1
Slc29a3	0.046825	0.047825	1
Bcl2a1b	0.046824	0.078492	1
Arpc3	0.046813	0.001841	1
Gm10138	0.046775	0.080052	1
Mpg	0.046706	0.001434	1
Smc5	0.046656	0.007811	1
Brix1	0.046635	1.55E-05	0.500022
Sap18	0.046616	0.000509	1
Uqcrq	0.046597	0.010278	1
Tor2a	0.046445	0.00067	1
Rpl36al	0.046379	0.000438	1
Necap2	0.046204	0.03652	1
Cstb	0.046137	0.001099	1
Errfi1	0.046091	0.011224	1
Gimap6	0.046065	3.49E-05	1
Rps18	0.046045	0.000513	1
Chd6	0.046041	0.197728	1

Hsd17b10	0.046032	0.009778	1
Col27a1	0.045983	0.089856	1
Dapk1	0.045932	0.044897	1
Ppcdc	0.045926	0.13785	1
Ptpmt1	0.0459	0.017393	1
Micu3	0.045857	0.288539	1
Iws1	0.04582	0.030307	1
1700110C19Rik	0.045799	0.010942	1
Mfng	0.045541	0.00521	1
Camk2n1	0.045457	0.081223	1
Slc39a1	0.045447	0.017571	1
Rfc3	0.045418	0.000259	1
Spast	0.045396	0.174404	1
D8Ertd738e	0.045359	0.062587	1
Rbm42	0.045297	0.000485	1
Ndufab1	0.045253	0.036682	1
Eif3c	0.04517	0.025629	1
Mfsd14a	0.045036	0.007131	1
Pde8b	0.044996	0.013752	1
Rps17	0.044996	0.004887	1
Hspa4	0.04499	0.03073	1
Tram1	0.044892	0.036135	1
Srp14	0.044834	0.000663	1
Sec61b	0.044818	0.014588	1
Cox7a2l	0.044802	0.000595	1
Ptges3	0.044797	0.00376	1
Ablim1	0.044741	0.00866	1
Poc1b	0.044648	0.043295	1
Commd3	0.044551	0.001048	1
Ifi30	0.044512	0.003624	1
Slamf8	0.044455	0.033522	1
Msmo1	0.044378	0.004267	1
Cops9	0.044322	0.011226	1
Havcr2	0.044297	0.02218	1
Tvp23b	0.044291	0.05728	1
Golga5	0.044246	0.095816	1
Cenpb	0.044165	0.000197	1
Ebna1bp2	0.044123	0.013594	1
Fdft1	0.044073	0.002227	1
Golt1b	0.043985	0.011516	1
Dusp3	0.043974	0.002743	1
Pgk1	0.043967	0.039185	1
Dennd2c	0.043934	0.035512	1
Uqcrc2	0.043846	0.051837	1
Micos13	0.043825	0.02448	1
Vmp1	0.043807	0.008766	1
Spg20	0.043792	0.046443	1
Ide	0.043742	0.003027	1

Aga	0.043736	0.006805	1
Brip1os	0.043727	0.152494	1
Rbm3	0.043686	0.000287	1
Napa	0.043686	0.006016	1
Lmnb1	0.043686	0.004569	1
Cox7c	0.043676	0.000196	1
Btbd19	0.043524	0.006684	1
Actl6a	0.043499	0.001969	1
Timm13	0.043422	0.015324	1
Ndufs8	0.043401	0.019175	1
Zswim8	0.043396	0.101642	1
Dennd4a	0.043394	0.090655	1
Sycp2	0.043393	0.32489	1
Insig1	0.043335	8.96E-05	1
Eif4a2	0.043299	0.015762	1
Dek	0.04314	0.006504	1
Ndufa7	0.043121	0.003086	1
Hspa9	0.043055	0.002264	1
Adh5	0.043008	0.005195	1
Uqcr11	0.042965	0.008627	1
Eif3h	0.042947	0.023847	1
Gm14023	0.042906	7.36E-05	1
Tbl3	0.0429	0.043143	1
Erp29	0.042766	0.013088	1
Csf1	0.042694	0.474176	1
Arhgef40	0.042538	0.028881	1
Golgb1	0.042509	0.027718	1
1110032A03Rik	0.042497	0.009867	1
Nf1	0.04248	0.023589	1
Polr2k	0.042233	0.00134	1
Leprotl1	0.042153	0.003838	1
Donson	0.042122	0.000158	1
Atg2a	0.042071	0.011222	1
Arl8b	0.042003	0.033537	1
Hadha	0.041965	0.015333	1
C5ar1	0.041855	0.362957	1
Xrcc5	0.041795	0.661723	1
Derl2	0.041703	0.047506	1
Rnf130	0.041685	0.013304	1
Thra	0.041681	0.000271	1
Eif2s1	0.041637	0.152611	1
Washc1	0.041573	0.012148	1
Med31	0.041539	0.002399	1
Adss	0.041509	0.00585	1
Cox8a	0.041478	0.000656	1
Sumo2	0.041472	0.012145	1
Utp18	0.041461	0.050332	1
Med12l	0.041456	0.030712	1

Eif3g	0.041382	0.001412	1
Prkar1a	0.041301	0.009782	1
Cnih1	0.041057	0.014637	1
Hif1a	0.040895	0.04618	1
Rnf157	0.040851	0.031086	1
Hpgd	0.040843	0.323382	1
Gatd3a	0.040826	0.000506	1
Ets1	0.040756	0.003089	1
Pex1	0.040729	0.537548	1
Ccnd3	0.040728	0.015624	1
Lamtor4	0.040637	0.000906	1
Vps4b	0.040413	0.014081	1
Grap	0.040403	0.162928	1
Slc20a1	0.040327	0.01037	1
Ildr1	0.040294	2.18E-05	0.705115
Sdcbp	0.040245	0.02356	1
Arv1	0.040202	0.01882	1
1700017B05Rik	0.040104	0.016476	1
Acvrl1	0.040103	0.018056	1
Atad5	0.039968	0.479903	1
Rpn1	0.039928	0.034012	1
Ndufs7	0.039891	0.090999	1
Degs1	0.039852	0.007269	1
Tspan9	0.03985	0.024398	1
Cct2	0.039827	0.082393	1
Bin1	0.039813	0.029024	1
Limd2	0.039754	0.062136	1
Fis1	0.039717	0.003471	1
Pld3	0.039696	0.001331	1
Cgrrf1	0.039677	0.01736	1
Edf1	0.03967	0.019502	1
Ilf2	0.039657	0.402645	1
Cyp20a1	0.039609	0.001752	1
Snhg4	0.039565	0.04809	1
Mfge8	0.039519	0.00421	1
Nedd8	0.039517	0.010235	1
Liph	0.039422	0.011003	1
Gnai2	0.039418	0.022628	1
Gm16192	0.039386	0.000726	1
Nup205	0.03934	0.147476	1
Ski	0.039311	0.06084	1
Sowahc	0.039242	0.234781	1
Gm5617	0.039228	0.054152	1
Myl6	0.03922	0.000927	1
Prox2	0.039206	0.093204	1
Rabgap1l	0.03913	0.048825	1
Esf1	0.039109	0.004597	1
Bod1l	0.039107	0.013375	1

Nop14	0.039105	0.054	1
Phf5a	0.039014	0.042685	1
H2afz	0.038989	8.43E-05	1
Scaf11	0.038874	0.032295	1
Gpr84	0.038844	0.404451	1
Naglu	0.038837	0.032334	1
Vdac1	0.038786	0.004722	1
Pdcd4	0.038783	0.064616	1
Tmtc3	0.038762	0.227941	1
Chmp5	0.038744	0.083237	1
Map1lc3b	0.038703	0.00132	1
Romo1	0.038664	0.05966	1
Fuca1	0.038648	0.018004	1
Cd244a	0.0386	0.092835	1
Hcls1	0.038598	0.0696	1
Alg6	0.038548	0.021256	1
Atp5o	0.038524	0.0244	1
Usp22	0.038518	0.002326	1
Rin2	0.03849	0.029742	1
Lipa	0.038459	0.127957	1
Slc50a1	0.038446	0.062346	1
Stab1	0.038437	0.254924	1
Ldlrad4	0.038429	0.007114	1
Slamf6	0.03839	0.018055	1
Polr2a	0.038377	0.029344	1
Mrpl54	0.038348	0.002887	1
Epc1	0.03834	0.199006	1
Zbtb24	0.038318	0.003161	1
Zfp697	0.038308	0.819235	1
mt-Nd6	0.038298	0.000962	1
Srek1	0.038068	0.028918	1
Ndufb2	0.038059	0.02826	1
Pbdc1	0.037935	0.034811	1
Scamp2	0.037902	0.065478	1
Tmem160	0.037888	0.00323	1
Galk1	0.037868	0.001571	1
Sulf2	0.037834	0.12619	1
Nav2	0.037832	0.087175	1
Mnt	0.037776	7.03E-05	1
Camk2g	0.037742	0.076822	1
Plbd2	0.037718	0.007338	1
Atf4	0.037655	0.004559	1
Epn2	0.03764	0.708871	1
Ccdc115	0.03763	0.083167	1
Acox3	0.037573	0.075844	1
Ifitm2	0.037532	5.91E-05	1
Smpdl3a	0.037492	1.49E-05	0.480088
Gnptg	0.03748	0.031162	1

C330007P06Rik	0.037439	0.203693	1
Got1	0.03741	0.005143	1
AU022793	0.037361	0.035732	1
Tmem33	0.037334	0.062496	1
Sgta	0.03732	0.009965	1
Cks2	0.037315	0.281358	1
Srek1ip1	0.03726	0.027378	1
Jup	0.037257	0.093845	1
Nr2c2	0.037252	0.085894	1
Cisd1	0.03722	0.021648	1
Arhgap30	0.037149	0.120903	1
BC005624	0.037079	0.037535	1
Eif3a	0.037075	0.014874	1
Rpl31	0.037073	0.020702	1
Gm38843	0.037055	0.035488	1
Exoc5	0.037042	0.048181	1
Ywhaq	0.037037	0.153247	1
Slc35e1	0.037022	0.011879	1
Commd10	0.03699	0.15597	1
Vps28	0.03696	0.040359	1
Slc25a3	0.036956	0.001439	1
Dctn2	0.036928	0.105269	1
Arpc5	0.036918	0.008118	1
Eif2ak4	0.036885	0.196998	1
Snw1	0.036832	0.038875	1
Tbc1d15	0.036809	0.066104	1
Erh	0.036794	0.000626	1
Gpx4	0.03679	0.005351	1
Rab5a	0.03673	0.01385	1
Taz	0.036729	0.01411	1
Ywhae	0.036688	0.055945	1
Armh3	0.036649	0.181442	1
Sf1	0.036635	0.06177	1
Cops6	0.036578	0.018758	1
Atp6v1a	0.036559	0.280798	1
Il6st	0.036529	0.059773	1
Grpel1	0.036489	0.021895	1
Slc7a6os	0.036429	0.089723	1
D330023K18Rik	0.036412	0.115485	1
Bloc1s1	0.036399	3.18E-06	0.10265
Psap	0.036362	0.118119	1
Med7	0.036328	0.018295	1
Cacybp	0.036293	0.023672	1
Nfkbid	0.036291	0.005248	1
Clec4a2	0.036275	0.038887	1
Cox6a1	0.036196	0.002433	1
Pex2	0.036144	0.031482	1
Gtf2h2	0.036126	0.039769	1

Eif3i	0.036115	0.009115	1
Alox5ap	0.036102	0.017315	1
Parp1	0.036095	0.6778	1
Ccnq	0.036081	0.002551	1
Ppia	0.036034	0.002357	1
Emc3	0.036026	0.005202	1
Marc2	0.036017	0.022986	1
Mapre1	0.035974	0.011997	1
Comtd1	0.03591	0.009195	1
Slc25a11	0.035881	0.01539	1
Park7	0.035855	0.051554	1
Med23	0.035852	0.109786	1
Igfbp4	0.035734	0.0022	1
Memo1	0.035705	0.026078	1
Cyfip1	0.035657	0.117663	1
Akr1b3	0.035645	0.019236	1
Fbxl12	0.035636	0.059148	1
Ptp4a3	0.035619	0.058334	1
Elp1	0.035606	0.076305	1
Rtraf	0.035592	0.001166	1
Aph1a	0.035548	0.162043	1
Mpdu1	0.035484	0.045591	1
Akirin2	0.035435	0.003419	1
Elob	0.035396	0.003449	1
Gm9725	0.03535	0.00177	1
Grcc10	0.035284	0.04349	1
Map2k2	0.035228	0.098254	1
Mrps5	0.035222	0.006167	1
Hnrnpu	0.035215	0.047336	1
Pigb	0.035183	0.464967	1
Snrpa	0.035127	0.024429	1
Rrp1b	0.035106	0.171809	1
Snrpe	0.035092	0.017715	1
Gm47664	0.035081	0.008377	1
Prpf39	0.035003	0.120642	1
Trappc4	0.034904	0.082245	1
Gsg1	0.034898	0.036359	1
Mpc1	0.034852	0.149356	1
Eif2ak3	0.034823	0.378437	1
Ppwd1	0.034809	0.440171	1
Pabpn1	0.034729	0.05652	1
Dlst	0.034709	0.157239	1
Dcxr	0.034694	0.096325	1
Myl12a	0.034688	0.014885	1
Zmat2	0.03465	0.009261	1
Coq2	0.0346	0.187191	1
Atp13a3	0.034542	0.023434	1
Gaa	0.034527	0.010023	1

Lyrm2	0.034515	0.007929	1
Sgce	0.034498	0.49258	1
Hmgxb4	0.034409	0.248166	1
Tmem9b	0.034345	0.009227	1
Psmc4	0.034252	0.028457	1
Inpp4b	0.034223	0.096768	1
Xrn2	0.034204	0.088269	1
BC031181	0.03417	0.002406	1
Ccnl1	0.034169	0.081193	1
Kdelr2	0.034145	0.019469	1
Uevld	0.034121	0.056052	1
Zswim4	0.03412	0.026	1
Cdk5r1	0.034118	0.008887	1
Ykt6	0.03409	0.033827	1
Nipsnap3b	0.034086	0.233791	1
Gusb	0.034047	0.090824	1
Chchd1	0.03404	0.047361	1
Bbip1	0.034036	0.032675	1
Mrpl9	0.033982	0.548262	1
Mrps28	0.03388	0.041212	1
Osm	0.033879	0.087282	1
Psmb6	0.033871	0.170632	1
2610528A11Rik	0.03386	0.000616	1
Art3	0.033856	0.015842	1
Esr1	0.033844	0.883713	1
Neurl3	0.033777	0.162964	1
Pgp	0.03375	0.107321	1
Gnb1l	0.033673	0.186484	1
Asap3	0.03362	0.031985	1
Mrpl41	0.033505	0.007223	1
Spag7	0.033472	0.019492	1
Hnrnpab	0.033419	0.024739	1
Clec5a	0.033406	0.09516	1
Cirbp	0.0334	0.296979	1
Sms	0.033398	0.077553	1
Usp2	0.03336	0.039432	1
Elovl1	0.033303	0.095507	1
Dnm1l	0.033291	0.040047	1
Nav1	0.033269	0.203123	1
Zfp36l2	0.033262	0.353769	1
Yeats2	0.03326	0.185907	1
Ppig	0.03326	0.106489	1
Nop10	0.033257	0.09076	1
Ctnnd1	0.033228	0.446988	1
Fancc	0.033214	0.024177	1
Pum3	0.033214	0.248596	1
Lrrc3	0.033197	0.003517	1
Kazn	0.033188	0.008627	1

Fhit	0.03317	0.635386	1
Mppe1	0.033169	0.015864	1
Fam217b	0.03305	0.146298	1
Alyref2	0.033029	0.004394	1
Chn2	0.032998	0.037444	1
2410002F23Rik	0.032926	0.005295	1
Cndp2	0.032914	0.042551	1
Chka	0.032904	0.035476	1
Fbxo8	0.032835	0.145125	1
Pfkfb3	0.032806	0.636399	1
Ggta1	0.032769	0.075794	1
Hist2h2ac	0.032713	0.000245	1
Itpripl2	0.032689	0.04535	1
Rps6ka5	0.03268	0.163006	1
Bnip3	0.032659	0.023114	1
Sgpl1	0.032647	0.067422	1
Rell1	0.032642	0.001967	1
Tcea1	0.032631	0.012276	1
Ubl5	0.032621	0.010605	1
Polr2f	0.032592	0.118999	1
Usp1	0.032591	0.58553	1
Stau2	0.032581	0.211876	1
Pnkd	0.032576	0.012643	1
Top3a	0.032574	0.121901	1
Prdx4	0.032574	0.05403	1
Gadd45g	0.032464	0.000751	1
Cd37	0.032422	0.191235	1
Fam32a	0.032422	0.12225	1
Jmjd1c	0.032377	0.147783	1
Cops2	0.032362	0.300336	1
Vps37c	0.032357	0.167464	1
Copb1	0.03232	0.102853	1
Cox7b	0.03232	0.011901	1
Nop53	0.032317	0.103462	1
Rubcnl	0.032271	0.069805	1
Hmgcr	0.032226	0.075892	1
Aggf1	0.032209	0.143065	1
Herpud2	0.032202	0.444088	1
Trib1	0.03218	0.101124	1
Phf23	0.032115	0.416081	1
Psma4	0.032103	0.020486	1
Cux1	0.03208	0.065269	1
Mdc1	0.032057	0.048619	1
Gdi2	0.032017	0.057725	1
Mrpl16	0.031997	0.004638	1
Tmem176a	0.031972	0.045876	1
Rsl24d1	0.031956	0.056813	1
Eftud2	0.031927	0.046537	1

Ginm1	0.031841	0.051181	1
Anxa5	0.031808	0.137316	1
Paip1	0.031768	0.464483	1
Ebp	0.031754	0.102712	1
Actr3	0.031745	0.167976	1
Dpm1	0.031742	0.103986	1
Mid1ip1	0.03167	0.028035	1
Tmem123	0.031663	0.10337	1
Osbpl3	0.031549	0.497085	1
Slc35b1	0.031537	0.109866	1
Lamp2	0.031507	0.031928	1
Tug1	0.031484	0.084612	1
Plaur	0.031366	0.097041	1
Exoc1	0.031363	0.011591	1
Arl2bp	0.031225	0.068766	1
Lair1	0.031195	0.002933	1
Tmem50b	0.031178	0.346775	1
Tmed4	0.031166	0.002532	1
Klhl7	0.031141	0.008476	1
Lipo3	0.031087	0.092836	1
Nktr	0.031065	0.065719	1
Pja2	0.031027	0.708281	1
Txnrd2	0.03101	0.213911	1
Lrch4	0.030985	0.017587	1
Srfbp1	0.030979	0.180474	1
Slc23a2	0.030925	0.085793	1
Midn	0.030876	0.041516	1
Spsb1	0.030862	0.05298	1
Gm20186	0.0308	0.000273	1
Cfl1	0.030784	0.009906	1
Fzd4	0.030773	0.0162	1
Med10	0.030761	0.002338	1
Atg12	0.030748	0.084204	1
Mogs	0.03073	0.193192	1
Ddx21	0.030724	0.119099	1
1700028E10Rik	0.030681	0.785574	1
Lyl1	0.030678	0.092518	1
Il4ra	0.030669	0.054355	1
Rbmxl1	0.030622	0.208413	1
Hnrnpl	0.030611	0.050265	1
Atp5k	0.030581	0.03816	1
Lman1	0.030487	0.003186	1
Pfdn2	0.030475	0.109224	1
B3gnt2	0.030474	0.337835	1
Ddx54	0.030445	0.002543	1
Kcnip3	0.030421	0.264926	1
Ak1	0.030414	0.101346	1
Spns2	0.030396	0.117017	1

H2-K1	0.030364	0.001434	1
Pycr2	0.030353	0.035122	1
Atp6v1g1	0.030348	0.071298	1
Gm12905	0.030329	0.031239	1
Atp6ap1	0.030279	0.024646	1
Srr	0.030268	0.464074	1
Mpv17l2	0.030262	0.172959	1
P3h2	0.030261	0.371254	1
Pex19	0.030255	0.102744	1
Atp6v0e	0.030234	0.009812	1
Bcap29	0.030229	0.187472	1
Tmem109	0.0302	0.043756	1
Nfatc2ip	0.030181	0.06558	1
Cep63	0.030174	0.379921	1
Vma21	0.030173	0.02181	1
Mrpl46	0.030128	0.003307	1
Hnrnpm	0.030075	0.172645	1
Eif1a	0.029974	0.132245	1
Lonrf1	0.02993	0.003623	1
Tnfrsf1a	0.02992	0.027365	1
Tpd52	0.029874	0.014812	1
Tmem41b	0.029851	0.024102	1
Mgat4a	0.029845	0.1446	1
Cdipt	0.02983	0.083269	1
Snrnp35	0.029815	0.02502	1
Bag1	0.029809	0.083555	1
Plod3	0.029799	0.035089	1
Ubxn1	0.02977	0.008566	1
Sub1	0.029731	0.073751	1
Snx5	0.029718	0.109519	1
Nme2	0.029712	0.018648	1
Trim7	0.029592	0.345777	1
Smim10l1	0.02955	0.018204	1
Cenpv	0.029451	0.132796	1
Hcfc1	0.029429	0.03532	1
Tmem223	0.029397	0.114107	1
Eef1g	0.029379	0.154564	1
Glrx3	0.02935	0.127046	1
Tmem141	0.029333	0.040841	1
Hspa14	0.029324	0.01288	1
4632427E13Rik	0.029323	0.351009	1
2810402E24Rik	0.029316	0.002325	1
Dhps	0.029301	0.095024	1
Snrpa1	0.029288	0.039149	1
Gm30198	0.029279	0.063536	1
Tomm22	0.029242	0.106834	1
Cnot8	0.029238	0.547264	1
Ipp	0.029189	0.127735	1

Csf1r	0.029182	0.013641	1
Bag3	0.029173	0.056464	1
Tbpl1	0.029142	0.240374	1
Fbxo3	0.029141	0.015059	1
Tbc1d9b	0.029133	0.219819	1
Mrto4	0.029129	0.001871	1
Arhgap35	0.02911	0.466243	1
Uggt2	0.029099	0.390374	1
Ryk	0.029077	0.400681	1
Jtb	0.029037	0.013083	1
Taf13	0.029028	0.065296	1
Mfap1a	0.028965	0.073938	1
Gm33858	0.028941	0.032305	1
Rwdd1	0.028938	0.053072	1
Tmbim6	0.02892	0.169858	1
Fance	0.028918	0.063999	1
Trappc2l	0.028849	0.15115	1
Trp53cor1	0.028816	0.849008	1
A630089N07Rik	0.028743	0.651663	1
Zmpste24	0.028735	0.15364	1
G3bp1	0.028722	0.178737	1
Clgn	0.028673	0.007455	1
Ssna1	0.028635	0.050324	1
Slc8b1	0.028624	0.041708	1
Grina	0.028618	0.009478	1
Phgdh	0.028611	0.090559	1
Ebi3	0.02859	0.052352	1
Wdpcp	0.028536	0.654067	1
Cltb	0.028473	0.104659	1
Srrm2	0.028467	0.007631	1
Eif4ebp1	0.028457	0.005535	1
Tm2d3	0.028451	0.085604	1
Qpctl	0.028433	0.573333	1
Psat1	0.028408	0.043221	1
Psmc6	0.028393	0.394087	1
Olfr111	0.02838	0.205762	1
Il11ra1	0.028355	0.140525	1
Agpat2	0.028331	0.026962	1
Msrb2	0.028315	0.136119	1
Ndufb5	0.028315	0.047361	1
Rmnd5a	0.02831	0.102498	1
Slc17a5	0.028282	0.408471	1
Spi1	0.028281	0.032738	1
H2-M3	0.028247	0.002966	1
Sigmar1	0.028206	0.011559	1
Psmc3	0.028186	0.060374	1
4933406I18Rik	0.028167	0.4167	1
Adi1	0.028159	0.005515	1

Lefty1	0.028053	0.000167	1
Kpnb1	0.028046	0.015318	1
Crybb1	0.028041	0.774065	1
Leng9	0.028035	0.043856	1
Garnl3	0.028005	0.342575	1
Tmem234	0.027985	0.14123	1
Mrps12	0.027983	0.011757	1
Slmap	0.027954	0.080415	1
Fgfr1op2	0.027944	0.205418	1
Supt4a	0.027905	0.060701	1
Ndufa1	0.027891	0.012982	1
Sec63	0.027827	0.628121	1
Prkacb	0.027812	0.281409	1
Adam3	0.027775	0.189669	1
Polr2j	0.027766	0.175296	1
Arl5b	0.027727	0.029427	1
Mrs2	0.02769	0.773356	1
Dnajc17	0.027639	0.041241	1
Rnf11	0.027588	0.066695	1
Mrpl12	0.027572	0.052233	1
Ncf4	0.027546	0.241546	1
Fcgr1	0.027541	0.08595	1
AI504432	0.027506	0.031119	1
Dusp16	0.027499	0.155509	1
Hvcn1	0.027482	0.033083	1
Mmd	0.027465	0.215192	1
Sf3b5	0.027462	0.023758	1
Lman2	0.027453	0.018877	1
P2ry13	0.027398	0.090451	1
Naxd	0.027321	0.099882	1
Fkbp3	0.027293	0.351399	1
Hnrnpa1	0.027291	0.027272	1
Smarcd1	0.027226	0.091583	1
Zfp266	0.02721	0.339654	1
Gm39469	0.027207	0.641647	1
Slc39a12	0.027166	0.062355	1
Slc25a33	0.027137	0.095751	1
Dhrs7	0.02712	0.005127	1
Hp1bp3	0.027096	0.134516	1
Morf4l2	0.02709	0.049063	1
Spcs3	0.027058	0.050356	1
Abcg2	0.027049	0.178755	1
Junos	0.027036	0.076783	1
Uba6	0.027029	0.995596	1
Pds5b	0.027018	0.223101	1
Svbp	0.026978	0.026925	1
Polr2e	0.026958	0.006376	1
Arap3	0.026901	0.311802	1

Ccni	0.026896	0.056239	1
Chchd5	0.026877	0.252169	1
Ston2	0.026864	0.1398	1
Tomm20	0.026847	0.027311	1
Ap1m1	0.026831	0.268887	1
Marcksl1	0.026825	0.058468	1
Pdlim4	0.026805	0.029302	1
Sh3bp5	0.026795	0.145389	1
Fcrl1	0.026759	0.389082	1
Slc2a1	0.026753	0.06851	1
Dmac1	0.026751	0.052656	1
Sf3b4	0.026739	0.042668	1
Npc1	0.026727	0.101952	1
Ssu72	0.026678	0.221842	1
Ap2m1	0.026645	0.292157	1
Stub1	0.026643	0.083192	1
Pigm	0.026609	0.5407	1
Alyref	0.026551	0.250882	1
Clns1a	0.026526	0.272641	1
Smc3	0.026516	0.413753	1
Gnpda2	0.026507	0.051025	1
Ecsit	0.026473	0.317639	1
Nbas	0.026462	0.158633	1
Puf60	0.026427	0.062624	1
Zdhhc3	0.026406	0.564685	1
Bub3	0.026398	0.198973	1
Nudt16l1	0.026397	0.058077	1
Ralgps1	0.026387	0.416775	1
Srsf11	0.026387	0.011967	1
Dpagt1	0.026379	0.095014	1
Fyn	0.026377	0.69839	1
Hacd3	0.026367	0.247801	1
D830025C05Rik	0.026352	0.016457	1
Rnf13	0.026333	0.08903	1
Rassf1	0.026333	0.157351	1
Naxe	0.026309	0.050615	1
Susd3	0.026303	0.182614	1
Cnnm3	0.0263	0.04067	1
Nucb1	0.026287	0.088379	1
Sept10	0.026285	0.608523	1
Ppp1ca	0.02628	0.033856	1
Vps52	0.026249	0.019436	1
Tmem11	0.026246	0.139999	1
Hist2h2aa1	0.026221	0.003349	1
Med30	0.026178	0.020955	1
Lamtor3	0.026142	0.382756	1
Slc15a4	0.026141	0.058698	1
Irf2bp2	0.026139	0.185558	1

Cebpz	0.026134	0.025428	1
Snx18	0.026127	0.082569	1
Fuca2	0.026107	0.165918	1
Sec11a	0.026097	0.037516	1
Smo	0.026081	0.195969	1
Srsf4	0.026075	0.591612	1
Cct4	0.026058	0.120728	1
Rbis	0.026033	0.044322	1
Fbxw9	0.026005	0.653796	1
Coa3	0.025989	0.150728	1
Pde4d	0.025979	0.115728	1
Blvrb	0.025965	0.257486	1
Fam110a	0.025964	0.021714	1
Prag1	0.025964	0.073013	1
Smagp	0.025944	0.080984	1
Grn	0.025923	0.259761	1
Tspan13	0.025906	0.00935	1
Cd302	0.025902	0.192036	1
Pola1	0.025901	0.560687	1
Ckap4	0.025893	0.028706	1
Surf1	0.025875	0.107663	1
Akirin1	0.025869	0.198686	1
Iffo1	0.025852	0.376342	1
Mtfr1l	0.025852	0.635418	1
Tmem159	0.025834	0.438985	1
Rab11b	0.025808	0.05944	1
Fam167b	0.025806	0.1531	1
Pde12	0.025772	0.140609	1
Ankra2	0.025769	0.019046	1
Rcn2	0.025758	0.012362	1
Sf3b1	0.02575	0.025937	1
Gpat4	0.025723	0.129389	1
Ppp2r2d	0.025653	0.289533	1
Trappc6a	0.025628	0.05777	1
Leng8	0.025613	0.269291	1
Ndufaf2	0.025591	0.46539	1
Tsc1	0.025553	0.255672	1
Abcb4	0.025547	0.025712	1
Nxf1	0.025502	0.248554	1
Zfp653	0.025492	0.260762	1
Mr1	0.025483	0.08179	1
Ctss	0.025457	0.027079	1
Decr1	0.025443	0.254025	1
Bag5	0.025417	0.122547	1
Set	0.025398	0.041853	1
St6galnac4	0.025397	0.004658	1
Telo2	0.025351	0.104336	1
Tbcb	0.02533	0.477219	1

Sec61a1	0.025249	0.25519	1
Ppm1a	0.025242	0.109259	1
Fam114a2	0.02522	0.127927	1
Psmb8	0.025183	0.127871	1
Arhgap31	0.025177	0.372932	1
Disc1	0.025172	0.270452	1
Pex5	0.025155	0.052811	1
Mapk6	0.025132	0.20349	1
Msl3	0.025098	0.052741	1
Cuedc2	0.025083	0.103895	1
Ppp6r1	0.025055	0.50551	1
Isy1	0.025046	0.133617	1
Tm7sf3	0.025046	0.04055	1
Trim65	0.025046	0.473504	1
Zdhhc5	0.02502	0.151846	1
Yif1a	0.025014	0.0057	1
Ahsa2	0.025006	0.071259	1
Map2k3	0.024983	0.116122	1
Tpd52l2	0.024965	0.025535	1
Mtch2	0.024948	0.057577	1
Srrm1	0.024944	0.329009	1
Kctd6	0.024918	0.023481	1
Csnk2b	0.024909	0.877618	1
Exosc3	0.024898	0.011539	1
Arf2	0.024892	0.106375	1
Selenot	0.024892	0.137013	1
Prorsd1	0.024853	0.092808	1
Pcbp1	0.02484	0.258219	1
Erlec1	0.024814	0.165494	1
Nol11	0.024802	0.512106	1
Gba	0.02475	0.425435	1
Xylt1	0.024749	0.022008	1
Rnf187	0.024726	0.327735	1
Ly6i	0.024705	0.119538	1
Ints11	0.024705	0.86954	1
Mrpl33	0.024653	0.103599	1
Kdsr	0.024632	0.031121	1
Cyb5r3	0.024577	0.226706	1
Glipr1	0.024561	0.162612	1
Atp6ap2	0.024544	0.021199	1
Arfip1	0.024541	0.017982	1
Ttc7b	0.024514	0.181738	1
Tspyl2	0.024503	0.00017	1
Gm9949	0.024484	0.024982	1
Setd3	0.02448	0.369207	1
Tceal9	0.02445	0.061767	1
Prpf19	0.024438	0.293555	1
Flii	0.024432	0.569488	1

Rnf121	0.024382	0.7165	1
Gm31410	0.02438	0.898686	1
Agk	0.024338	0.079954	1
Armcx3	0.024337	0.070794	1
Pdcd5	0.024336	0.20434	1
Ncstn	0.024236	0.032775	1
Vwa5a	0.024185	0.269107	1
Igsf8	0.024138	0.309854	1
Luc7l2	0.024128	0.170176	1
Rngtt	0.024128	0.165615	1
Urb2	0.024126	0.169837	1
Ccl6	0.024103	0.860165	1
Cib1	0.024095	0.0691	1
Ccng2	0.024061	0.024187	1
Cyp51	0.024043	0.041029	1
Cetn2	0.024028	0.067609	1
Slc29a1	0.024014	0.155146	1
Map7	0.024012	0.03424	1
Rcl1	0.024005	0.048297	1
Sdf2	0.024003	0.362049	1
Mad2l2	0.023973	0.055823	1
Uggt1	0.023965	0.107224	1
Mtln	0.023933	0.099715	1
Pabpc1	0.023927	0.226707	1
Lag3	0.023924	0.096078	1
Ran	0.023911	0.008967	1
Nhlrc3	0.023911	0.097492	1
Vti1b	0.023909	0.069276	1
Eif3f	0.023897	0.006634	1
Ash2l	0.023877	0.504077	1
Eef1e1	0.023852	0.027369	1
Eva1b	0.023837	0.12736	1
Gm20721	0.023832	0.007211	1
Timm17b	0.023811	0.001176	1
Akap1	0.023809	0.305298	1
Cyth2	0.023793	0.002115	1
Snhg15	0.023788	0.024521	1
Copz1	0.023773	0.67765	1
Atp6v1d	0.023749	0.117802	1
Rgs7bp	0.023711	0.105268	1
Ntmt1	0.02371	0.176125	1
Zfp846	0.02371	0.487714	1
Fam133b	0.023709	0.100867	1
Bclaf3	0.023688	0.540684	1
Arl1	0.023656	0.008456	1
Tnip2	0.02364	0.149078	1
Dhrs3	0.023633	0.031937	1
Ms4a6d	0.023617	0.151777	1

Nudcd2	0.023612	0.496155	1
Syf2	0.023602	0.1213	1
Gm48855	0.0236	0.851446	1
Usp28	0.023596	0.022418	1
Armc10	0.023592	0.407005	1
Atxn7l3b	0.023584	0.476046	1
Cdc27	0.023564	0.717316	1
Lgals3bp	0.023563	0.165737	1
Dhx29	0.023494	0.957747	1
Rbsn	0.023482	0.084816	1
Ndufv3	0.02348	0.155624	1
Per1	0.023472	0.016212	1
Lmbrd2	0.023464	0.139442	1
Zfp335os	0.023463	0.007318	1
Gpr155	0.02345	0.366127	1
Vcp	0.023449	0.154237	1
Lnpk	0.02339	0.21576	1
Lacc1	0.023388	0.191086	1
Tgm2	0.023374	0.506693	1
Snapc3	0.02337	0.102404	1
Xlr	0.023357	0.071389	1
Eif4a3	0.023356	0.082661	1
Matr3	0.023349	0.358048	1
Agtrap	0.023341	0.050785	1
Tspan18	0.02334	0.166889	1
Mospd2	0.023322	0.347166	1
Nus1	0.023296	0.501531	1
Gm30054	0.023274	0.016833	1
Il6	0.023272	0.085113	1
Magt1	0.023259	0.055067	1
Zfyve21	0.02325	0.041207	1
Atp6v1f	0.023243	0.031602	1
Plxnb2	0.023232	0.077879	1
Rabep2	0.023187	0.065758	1
Klhdc4	0.023146	0.007041	1
Myo1b	0.023145	0.414279	1
Tmem60	0.023143	0.054576	1
Sap30bp	0.023115	0.276871	1
Colgalt1	0.023093	0.221847	1
CAAA01118383.1	0.023082	0.247961	1
Upf3a	0.023076	0.133735	1
Rab5if	0.023059	0.106823	1
Haghl	0.02305	0.161463	1
Ccdc47	0.023042	0.098522	1
4930581F22Rik	0.023039	0.119951	1
Ndufa2	0.023034	0.049943	1
Aprt	0.022955	0.167718	1
Khsrp	0.022954	0.913915	1

Fancl	0.022937	0.595171	1
Ndufb7	0.022925	0.060609	1
Cnot6	0.02292	0.116498	1
Commd7	0.022896	0.073375	1
Tmem9	0.022895	0.022103	1
Bcl7b	0.022885	0.066091	1
Mpc2	0.022867	0.168613	1
Cbx4	0.022863	0.019677	1
Tuba1c	0.022858	0.201124	1
Acp1	0.022829	0.100419	1
Top1	0.022739	0.043852	1
Pdcl	0.02272	0.102443	1
Zbtb10	0.022674	0.091778	1
Lpcat2	0.022641	0.041361	1
Paqr7	0.022609	0.263815	1
Arhgap19	0.022606	0.352299	1
C1qbp	0.022589	0.210784	1
Gsr	0.022578	0.77496	1
Tmem251	0.022563	0.135683	1
Tppp	0.022541	0.309057	1
H2-Q6	0.022535	0.150803	1
Dtnb	0.022469	0.13313	1
Peak1	0.022468	0.159637	1
Ipo5	0.022433	0.224046	1
5730480H06Rik	0.022424	0.487552	1
Sra1	0.022409	0.021107	1
Ndufa11	0.022405	0.026906	1
Trmt112	0.022392	0.02106	1
Exosc10	0.022377	0.116764	1
Csnk1g2	0.022349	0.2218	1
Emc4	0.022349	0.370776	1
Apmap	0.022344	0.122374	1
Ppp2r1a	0.022336	0.071475	1
Prxl2b	0.022329	0.04345	1
Ercc5	0.022277	0.335182	1
Enc1	0.022271	0.325156	1
Hbs1l	0.022253	0.200275	1
Glyr1	0.022253	0.769683	1
Fmc1	0.022223	0.210557	1
Wdr76	0.022198	0.07947	1
Epb41l2	0.022194	0.083462	1
Nfe2l1	0.022193	0.41246	1
Actr1b	0.022137	0.515673	1
Golga4	0.022087	0.059947	1
Zfp143	0.02206	0.353185	1
Ctbs	0.022055	0.265434	1
Sgpp1	0.022053	0.772817	1
Plekhg5	0.022052	0.076058	1

Naga	0.022019	0.103816	1
Klhl9	0.022006	0.580043	1
Spata24	0.021999	0.028536	1
Cybc1	0.021992	0.097491	1
Hmga1	0.021972	0.021415	1
Fopnl	0.021948	0.736609	1
Ubald1	0.021944	0.015631	1
Gmpr	0.021931	0.108236	1
Tpbgl	0.021922	0.45684	1
Srsf6	0.02192	0.013461	1
Aldh9a1	0.021889	0.273345	1
Nlrp3	0.021885	0.258647	1
Rmdn1	0.021846	0.014305	1
Timm50	0.021814	0.114362	1
Eif2s2	0.021806	0.061482	1
Gimap1	0.021781	0.036316	1
Ago4	0.021775	0.410264	1
Jagn1	0.021761	0.10641	1
Ly86	0.021725	0.606529	1
Iah1	0.021691	0.07132	1
Nemp2	0.021635	0.149386	1
4930522L14Rik	0.021613	0.163469	1
Rnf2	0.021548	0.366857	1
Slc39a6	0.021543	0.184389	1
Pou2f2	0.02153	0.716003	1
Arhgap11a	0.021502	0.696805	1
Zfp622	0.021488	0.223552	1
Gk	0.021479	0.289176	1
Cfap20	0.021459	0.576588	1
Eif4enif1	0.021452	0.646422	1
Idh1	0.021445	0.050114	1
Rhoc	0.021413	0.022392	1
Zfp729b	0.021401	0.899625	1
Phb2	0.02129	0.027036	1
B3galt6	0.021273	0.701373	1
Dnajc30	0.021272	0.452398	1
Rxylt1	0.021265	0.007159	1
Snu13	0.021263	0.058132	1
Tctex1d2	0.021248	0.040413	1
Serbp1	0.021222	0.352441	1
Manbal	0.021212	0.082354	1
Fyttd1	0.021183	0.276113	1
Rmc1	0.021182	0.132181	1
Coro1b	0.021173	0.014559	1
Zfand2a	0.021152	0.358186	1
Fundc2	0.021142	0.065951	1
Ppp3cb	0.021113	0.177787	1
Ddx49	0.021103	0.605045	1

Ost4	0.021087	0.124429	1
2610002M06Rik	0.021077	0.112551	1
Eif2b1	0.021033	0.107731	1
Slc48a1	0.02101	0.020698	1
Erg28	0.021002	0.303253	1
Hnrnpf	0.020992	0.014082	1
Isyna1	0.020988	0.505268	1
Ciao2a	0.020986	0.073894	1
Ctdnep1	0.020985	0.072696	1
Adrm1	0.020978	0.110019	1
Prdx6	0.020976	0.082594	1
Lamtor5	0.020957	0.196655	1
Vhl	0.020951	0.783594	1
Tle5	0.020921	0.390049	1
Nudt16	0.020915	0.073407	1
Znrd1	0.020912	0.746308	1
A930024E05Rik	0.020897	0.205932	1
Pstk	0.020894	0.100693	1
Gmfg	0.020892	0.177019	1
Rabepk	0.020884	0.034681	1
Smpd4	0.020884	0.039845	1
Phlpp1	0.020861	0.333835	1
Oxa1l	0.020859	0.12925	1
Yy1	0.020854	0.300041	1
Gps2	0.020814	0.075903	1
Tango6	0.020803	0.691115	1
Lta4h	0.020802	0.719634	1
Zfp799	0.020781	0.139818	1
Cmpk1	0.020772	0.274002	1
Fdps	0.020725	0.260122	1
Ddx1	0.020716	0.218208	1
Nudt9	0.02071	0.018859	1
Trim24	0.020686	0.09491	1
Klf6	0.020662	0.620273	1
Ostf1	0.020648	0.069877	1
Klhl15	0.020629	0.374862	1
Ndufs5	0.020607	0.062318	1
Noc3l	0.02058	0.662872	1
Ctdspl	0.020567	0.202922	1
Hspd1	0.020556	0.067397	1
Srpr	0.020539	0.070027	1
Ids	0.020538	0.271381	1
Fut10	0.020502	0.447272	1
Srsf3	0.0205	0.112734	1
Ndufb10	0.020499	0.023539	1
Mettl14	0.020497	0.298981	1
Srrd	0.020483	0.087268	1
Lage3	0.020477	0.263614	1

Faim	0.020477	0.083197	1
Surf2	0.020426	0.015042	1
Dhx36	0.020404	0.027236	1
2310015A10Rik	0.020398	0.276715	1
Trir	0.020381	0.024916	1
Zfpl1	0.020374	0.122543	1
Vdac2	0.020362	0.085495	1
Timm22	0.020361	0.09714	1
Zfp704	0.020342	0.696006	1
Psmd4	0.020323	0.046458	1
Bnip1	0.020319	0.721194	1
Ube2s	0.020299	0.032098	1
Ebag9	0.020289	0.478192	1
Alkbh2	0.020264	0.172533	1
Tmem173	0.020252	0.386488	1
Cwc15	0.020225	0.297272	1
Senp6	0.020172	0.189191	1
Mea1	0.020171	0.152374	1
Scp2	0.020166	0.135556	1
Sec24c	0.020161	0.019652	1
Rpp21	0.020149	0.089757	1
Homer3	0.02013	0.10973	1
Ctbp1	0.020129	0.435538	1
Hmgn2	0.020107	0.10691	1
Nucks1	0.020098	0.024879	1
Nudc	0.020078	0.189564	1
Atad3a	0.020036	0.202284	1
Naa38	0.020019	0.099841	1
Snapc1	0.020013	0.63556	1
Ufl1	0.020006	0.76105	1
Rapgef5	0.019998	0.606229	1
H2afv	0.019982	0.265545	1
P4hb	0.019979	0.143154	1
Snrpb2	0.019968	0.024958	1
Ptbp1	0.019957	0.353481	1
Selenoh	0.019956	0.10989	1
Aimp2	0.019933	0.027501	1
Lsm1	0.019928	0.700549	1
Nsmce1	0.019898	0.429024	1
Polr2m	0.019889	0.289043	1
Siglece	0.019888	0.319209	1
Esyt2	0.019885	0.571534	1
Ears2	0.019881	0.121087	1
Thrsp	0.019872	0.275389	1
Src	0.019862	0.123076	1
Ldb1	0.019839	0.170623	1
Rela	0.019822	0.09735	1
Atp5md	0.019806	0.211621	1

Mdm4	0.019788	0.813606	1
Ints12	0.019788	0.152211	1
Socs4	0.019753	0.122516	1
Idua	0.019725	0.119828	1
Coa6	0.019712	0.122838	1
Hjurp	0.01971	0.319211	1
Ccnd1	0.019696	0.29745	1
Tceal8	0.019685	0.07445	1
Bckdk	0.019638	0.818068	1
Slc39a10	0.019635	0.074622	1
Dld	0.019623	0.162272	1
Egln2	0.019617	0.456747	1
Zmym3	0.019597	0.116164	1
Btg3	0.019557	0.019835	1
Churc1	0.019539	0.020653	1
Trmt13	0.019489	0.58528	1
Zdhhc20	0.019488	0.080919	1
Gps1	0.019466	0.239457	1
Ramp1	0.019444	0.783301	1
Med24	0.019443	0.567504	1
Gxylt1	0.019408	0.350245	1
Iffo2	0.019396	0.18838	1
Cisd2	0.019369	0.93386	1
Smad4	0.019361	0.228203	1
Nsun6	0.019353	0.858819	1
Pecam1	0.019346	0.453893	1
Cln5	0.019344	0.171927	1
Ipo9	0.019341	0.042676	1
Cops8	0.019336	0.133507	1
Atpif1	0.019328	0.019369	1
Tgfbi	0.019317	0.459445	1
Fzr1	0.01931	0.032697	1
Fam78a	0.019295	0.228217	1
Iqsec1	0.019235	0.404388	1
Dnajc1	0.019224	0.362263	1
Suds3	0.019209	0.547287	1
Etfdh	0.019187	0.679613	1
Srp54a	0.01915	0.276575	1
Psmg4	0.019123	0.087178	1
Mcrip1	0.019117	0.433277	1
Prdx3	0.019113	0.285694	1
Slc2a8	0.019111	0.107908	1
Ppp3r1	0.019078	0.640171	1
Haus8	0.01906	0.016758	1
P2ry10b	0.019053	0.190772	1
Hsd3b7	0.019032	0.043157	1
Uqcr10	0.01902	0.441522	1
Mars	0.018987	0.194585	1

Gmeb2	0.018959	0.104563	1
Dcun1d4	0.018949	0.90825	1
Fibp	0.018936	0.27234	1
Arl10	0.018923	0.424284	1
Slc30a5	0.018914	0.314527	1
Hat1	0.018907	0.36761	1
Trim36	0.018906	0.32058	1
Trp53bp2	0.018874	0.013918	1
Mrpl51	0.01886	0.347496	1
Smim26	0.01885	0.428995	1
Rab3il1	0.018847	0.128331	1
Abhd2	0.018819	0.274132	1
Actr5	0.018812	0.086813	1
Psma3	0.018802	0.161659	1
Adamts10	0.018799	0.188902	1
Wipi1	0.018797	0.154763	1
2900093K20Rik	0.018781	0.213612	1
Ankrd10	0.018776	0.091876	1
Rnf7	0.018756	0.057995	1
Srp9	0.018728	0.29244	1
Sumo3	0.018721	0.020897	1
Tmem43	0.018647	0.228969	1
Plin2	0.018637	0.520848	1
Alg2	0.018609	0.137771	1
Ttc32	0.018595	0.331654	1
Ndfip1	0.018582	0.239761	1
Banf1	0.018564	0.121717	1
Hccs	0.01855	0.423359	1
B4gat1	0.018549	0.06589	1
Cnrip1	0.01854	0.081833	1
Chchd2	0.018529	0.002928	1
Cfl2	0.018516	0.074682	1
Tubgcp2	0.018505	0.269488	1
Ccdc82	0.018458	0.209538	1
Sema4g	0.018457	0.190747	1
Atp5mpl	0.018446	0.205827	1
Ppfibp2	0.018439	0.121581	1
Stt3a	0.018403	0.076069	1
Pam16	0.018353	0.264402	1
Ubn1	0.018353	0.167954	1
Lman2l	0.018334	0.413253	1
Smn1	0.018331	0.18931	1
Rab35	0.018325	0.292969	1
Dctn3	0.01832	0.538418	1
Ggps1	0.018319	0.66844	1
Tm9sf2	0.018314	0.180835	1
Cwf19l1	0.018305	0.255793	1
Cul2	0.018298	0.803922	1

Asah1	0.018267	0.180711	1
Ptch1	0.018234	0.431835	1
Lpcat3	0.018199	0.270873	1
Pde8a	0.018187	0.00889	1
Htatsf1	0.018181	0.080322	1
Ccdc28b	0.018169	0.504785	1
Arsk	0.018167	0.099014	1
Cpox	0.018088	0.169069	1
Epb41l3	0.018087	0.325468	1
Mpp7	0.01808	0.317114	1
Tcaim	0.018075	0.809414	1
Ddt	0.018066	0.04743	1
Ighmbp2	0.018056	0.291574	1
Prdx5	0.018046	0.199424	1
Hmgb1	0.018043	0.046952	1
Pou6f1	0.018015	0.241862	1
Aldh6a1	0.018014	0.044072	1
Lrrc59	0.018006	0.069191	1
Acvr1	0.017998	0.074644	1
Noc2l	0.017988	0.112386	1
Zswim7	0.017978	0.012478	1
Fnbp1l	0.017943	0.470248	1
Eea1	0.017932	0.168599	1
Fam50a	0.017924	0.116176	1
Bace1	0.01788	0.299313	1
Eif5a	0.017872	0.02963	1
Snip1	0.01787	0.13167	1
Ctnna1	0.017841	0.943043	1
Snrnp27	0.017837	0.595506	1
Tut4	0.017829	0.403942	1
Rlim	0.017813	0.142035	1
Mettl5	0.017772	0.404733	1
Fut11	0.017745	0.193304	1
Mrpl30	0.017722	0.607648	1
Tuba1a	0.01772	0.156865	1
Flot2	0.017697	0.852635	1
Slc52a2	0.01769	0.243994	1
Lsm7	0.017687	0.563024	1
Lims1	0.017679	0.407237	1
Usp24	0.017657	0.442276	1
Plxna4os1	0.01765	0.219921	1
Clip1	0.017628	0.345789	1
Lamc1	0.017606	0.230132	1
Gsdme	0.017568	0.147343	1
Ncapd3	0.017558	0.589266	1
Vps39	0.01754	0.168863	1
Rbx1	0.017537	0.439572	1
Aip	0.017533	0.194927	1

Tsn	0.017521	0.031638	1
Ift46	0.017513	0.197412	1
Fus	0.0175	0.29075	1
B4galt5	0.017443	0.244313	1
Rab4b	0.017432	0.184561	1
Dcakd	0.017428	0.301171	1
Was	0.017411	0.211174	1
Mrpl18	0.017411	0.470441	1
Brk1	0.017403	0.013558	1
Chmp2a	0.017401	0.377465	1
0610012G03Rik	0.017359	0.110325	1
Hexdc	0.017354	0.289697	1
Amdhd2	0.017338	0.043584	1
Yif1b	0.017313	0.051062	1
Mfsd5	0.017311	0.012156	1
Firre	0.017302	0.337256	1
Crem	0.017291	0.166864	1
Ncbp2	0.01728	0.074247	1
Prdx2	0.017262	0.167577	1
Atn1	0.017255	0.802671	1
Zfp422	0.017247	0.095085	1
Epm2aip1	0.017242	0.217172	1
Tiprl	0.017216	0.167249	1
Thumpd2	0.017214	0.126536	1
Mettl17	0.01721	0.494489	1
Abhd17b	0.01719	0.514246	1
Abcd4	0.017185	0.611058	1
Zfp839	0.017179	0.773026	1
Stx7	0.017135	0.29455	1
Slc46a1	0.01713	0.868737	1
Riok1	0.017072	0.295295	1
Golph3	0.017058	0.259764	1
LTO1	0.017056	0.260912	1
St5	0.017051	0.32865	1
Mtss1	0.017041	0.184616	1
Gls	0.017035	0.404819	1
Ift22	0.017019	0.302593	1
Plekha4	0.017009	0.285334	1
Dhx16	0.016925	0.252722	1
Gnai3	0.016921	0.432513	1
Lemd2	0.016895	0.044057	1
Ndufa8	0.016878	0.01574	1
Serf2	0.016874	0.051463	1
Pign	0.016864	0.616366	1
Sri	0.016855	0.534697	1
Gm26759	0.016808	0.001892	1
Elmo1	0.016782	0.292139	1
Mkrn2	0.016779	0.134632	1

Gpaa1	0.016779	0.444892	1
Imp3	0.016773	0.117999	1
Tmx1	0.016767	0.219154	1
Guca1a	0.016766	0.054316	1
Nol7	0.016765	0.050734	1
Ift20	0.016765	0.204018	1
Ap4e1	0.016755	0.674114	1
Gm10790	0.016754	0.403111	1
Phb	0.01675	0.238329	1
Capns1	0.016744	0.454081	1
Prpsap1	0.016733	0.282338	1
Tfam	0.016719	0.690569	1
Fam160b2	0.016718	0.419104	1
Fbxl5	0.016711	0.234769	1
Mrps22	0.016699	0.510174	1
Rbbp7	0.016664	0.613052	1
Pin1	0.016649	0.181973	1
Psmd8	0.016637	0.054151	1
9130015G15Rik	0.016636	0.74803	1
Cd22	0.016633	0.389201	1
Cycs	0.016625	0.152355	1
Ralgps2	0.016589	0.174232	1
Timm10	0.016566	0.363897	1
Las1l	0.016562	0.776326	1
Npat	0.016542	0.478948	1
2810405F17Rik	0.016533	0.312858	1
Zfp91	0.016514	0.081112	1
Thrap3	0.016508	0.182135	1
Rbm34	0.016495	0.655337	1
Cenpo	0.016462	0.620534	1
Zfp24	0.016447	0.7964	1
Tprgl	0.016435	0.621669	1
Calm3	0.016434	0.111645	1
Lrp10	0.016394	0.341325	1
Pes1	0.016384	0.229115	1
Pcif1	0.016379	0.140201	1
E430024P14Rik	0.016354	0.633052	1
Ero1lb	0.016353	0.175509	1
Tax1bp3	0.016353	0.197149	1
Ate1	0.016349	0.961879	1
Hspb3	0.016335	0.1066	1
Cep44	0.016304	0.055091	1
Riox2	0.016303	0.025775	1
Cyb5d2	0.016292	0.686831	1
Tpm1	0.016292	0.102287	1
Pdha1	0.016289	0.080372	1
Arf1	0.016286	0.030202	1
Arid3b	0.016279	0.260183	1

Mfsd1	0.016277	0.317519	1
Ccdc152	0.016263	0.231768	1
Rnase6	0.016251	0.042623	1
Stoml2	0.016244	0.15719	1
Sdc4	0.016236	0.56043	1
Yipf4	0.01621	0.238631	1
Gadd45gip1	0.016201	0.809391	1
Adssl1	0.016191	0.26193	1
Pkp4	0.016151	0.588117	1
Samm50	0.01615	0.53944	1
Gm26782	0.016147	0.29422	1
Mak16	0.016131	0.218178	1
Depdc7	0.01612	0.137827	1
Cct5	0.016112	0.076643	1
Cdc23	0.016109	0.1984	1
Anp32a	0.016106	0.030398	1
Nr3c1	0.016092	0.859565	1
Pla2g12a	0.016092	0.940039	1
Sar1a	0.016082	0.318272	1
Meaf6	0.016057	0.451101	1
Mapk3	0.016051	0.375351	1
Cep57	0.016019	0.020904	1
Frg1	0.016017	0.125219	1
Nol8	0.016008	0.486946	1
Klc4	0.016007	0.159689	1
Klhl12	0.016007	0.373013	1
Naa10	0.016001	0.151518	1
Poglut2	0.015992	0.267473	1
Idh3g	0.01595	0.221516	1
Fam98b	0.01594	0.79639	1
2310033P09Rik	0.015935	0.29248	1
Eif3j1	0.015934	0.23103	1
Lats2	0.015912	0.342171	1
Aplp2	0.015904	0.896993	1
Rce1	0.015901	0.110095	1
Dynll2	0.0159	0.23465	1
Swi5	0.015892	0.092157	1
Tor1a	0.015872	0.147916	1
Tbc1d8b	0.015857	0.243567	1
Pdzd11	0.015829	0.190487	1
Tmem97	0.015811	0.1376	1
Prdx1	0.0158	0.123975	1
A430005L14Rik	0.015742	0.122884	1
6030468B19Rik	0.015699	0.218104	1
Zfp68	0.015698	0.430049	1
Pomp	0.015685	0.038436	1
Cops4	0.015669	0.897625	1
Cenpc1	0.015643	0.630039	1

Purb	0.01564	0.079491	1
Fcgrt	0.015635	0.165956	1
Fdxr	0.015633	0.164569	1
Tmem268	0.015609	0.151907	1
Denr	0.015591	0.326419	1
Ddb1	0.015575	0.862467	1
Snx19	0.01555	0.878759	1
Htatip2	0.01555	0.190728	1
Pxdc1	0.01554	0.711227	1
Timm23	0.015539	0.478582	1
Zcrb1	0.015537	0.419013	1
Tmem185a	0.015528	0.0177	1
Zc2hc1a	0.01552	0.731158	1
Dnajb4	0.015505	0.147737	1
Dimt1	0.015501	0.319933	1
Cct6a	0.015497	0.257113	1
Arhgap21	0.015461	0.750836	1
Atpsckmt	0.015451	0.778809	1
Pkib	0.015445	0.146637	1
Cfap410	0.015443	0.151736	1
Sept11	0.015428	0.468578	1
Vps36	0.015412	0.23467	1
Coq9	0.015393	0.965217	1
Maf1	0.015388	0.509021	1
Ufc1	0.015376	0.036622	1
Ncbp1	0.015375	0.318673	1
Cyp4v3	0.015354	0.103406	1
Smad5	0.015354	0.694085	1
Ccdc85b	0.015341	0.268716	1
Stk35	0.01534	0.541681	1
Yipf3	0.015339	0.191179	1
1700021F05Rik	0.015331	0.271752	1
Zfyve16	0.015306	0.784273	1
Higd1a	0.015288	0.217501	1
Caprin1	0.015281	0.067839	1
Fer	0.015267	0.700236	1
Tubb2b	0.015229	0.00569	1
Scamp5	0.015215	0.804527	1
Gm33699	0.015209	0.112518	1
Papola	0.015176	0.578646	1
Npm3	0.015168	0.078378	1
Nr2c2ap	0.015153	0.064787	1
Dync1li1	0.015151	0.727718	1
Use1	0.01515	0.091496	1
Fam3c	0.015135	0.152346	1
Twistnb	0.015134	0.226893	1
Usp49	0.01513	0.853733	1
Itgav	0.015123	0.397142	1

Cdk2ap2	0.015078	0.03585	1
Ly96	0.015072	0.198479	1
Cactin	0.015072	0.115946	1
Zfp69	0.015041	0.216591	1
Med12	0.015032	0.337699	1
Man1a	0.015015	0.522163	1
Uqcc3	0.015012	0.214621	1
Ralb	0.014957	0.392381	1
Edc3	0.014951	0.674314	1
Mif4gd	0.01495	0.27516	1
Atg2b	0.014921	0.311732	1
Fundc1	0.014917	0.225139	1
Kynu	0.014915	0.522841	1
Tmem250-ps	0.014891	0.121234	1
Sptssa	0.014883	0.114786	1
Ankrd46	0.01487	0.14287	1
Map3k4	0.014869	0.342208	1
Commd4	0.014852	0.085449	1
Fam8a1	0.014804	0.591804	1
Gcnt2	0.014802	0.150046	1
Clcn3	0.014794	0.163626	1
Ctcf	0.014793	0.3353	1
Rangap1	0.014782	0.460557	1
Bcor	0.014774	0.522741	1
Sin3b	0.014733	0.084669	1
Timm10b	0.014714	0.166301	1
Eef2kmt	0.014713	0.881477	1
Snf8	0.014704	0.936391	1
Mfap1b	0.014688	0.361419	1
BC017158	0.014671	0.381903	1
Ubxn2a	0.014669	0.60699	1
Selenon	0.014663	0.290326	1
Mrps27	0.014662	0.801817	1
Milr1	0.014613	0.494945	1
Ndufs6	0.014594	0.164933	1
Ythdc1	0.014574	0.887463	1
Gltp	0.014572	0.254065	1
Capn10	0.014571	0.268432	1
Kcnj2	0.014548	0.153357	1
Smpd5	0.014514	0.697544	1
Washc5	0.014496	0.061865	1
Elp2	0.014486	0.114486	1
B4galt7	0.014486	0.417021	1
Ints13	0.014478	0.117282	1
Apc	0.014476	0.160333	1
Pigk	0.014474	0.917148	1
2610035D17Rik	0.014469	0.450393	1
Pnkp	0.014469	0.687361	1

Ncoa4	0.014468	0.817049	1
Rtn4	0.014455	0.098755	1
Gm41611	0.014442	0.116791	1
Cd33	0.014441	0.394152	1
Magoh	0.01443	0.631606	1
Guk1	0.014414	0.352218	1
Sh3glb2	0.014405	0.497527	1
Slc39a8	0.014403	0.357593	1
Casp1	0.014369	0.859324	1
Gabarapl2	0.01436	0.158617	1
Zdhhc13	0.014317	0.143308	1
Elovl5	0.014311	0.898592	1
Bcl7a	0.014299	0.528417	1
Arhgap23	0.014297	0.348905	1
Trmu	0.014269	0.542133	1
Rusc2	0.014266	0.394901	1
Oaf	0.014256	0.067771	1
Atf1	0.014248	0.087568	1
Hikeshi	0.014235	0.757532	1
Osbpl2	0.014235	0.171775	1
Actn4	0.014211	0.736532	1
Pomt1	0.014198	0.42666	1
Zfp275	0.014191	0.09488	1
Gba2	0.014175	0.862797	1
Pomgnt1	0.014174	0.790688	1
Mrpl39	0.014171	0.17539	1
Fam89b	0.014171	0.96879	1
Skp1a	0.014169	0.358843	1
Vapa	0.014167	0.391139	1
Smc1a	0.014149	0.215064	1
Ube2d3	0.014148	0.076356	1
Dhx34	0.014144	0.272961	1
Zfp944	0.014144	0.708111	1
Dcun1d2	0.01414	0.63075	1
Birc6	0.014128	0.644297	1
Bmt2	0.01411	0.89084	1
Tsr1	0.014103	0.981354	1
Dstn	0.0141	0.787267	1
U2af1	0.014079	0.48313	1
Tagln2	0.014078	0.038622	1
Mthfsd	0.014064	0.305278	1
Osgin2	0.014062	0.045755	1
Ssrp1	0.014049	0.23505	1
Cep170	0.014026	0.247452	1
Ndel1	0.014015	0.916073	1
Pcyt2	0.014013	0.425699	1
Tmbim1	0.014009	0.345556	1
Slc35d2	0.014003	0.18516	1

Fh1	0.013987	0.143283	1
Lbh	0.013956	0.232686	1
Fam149b	0.013925	0.79851	1
Cct7	0.013917	0.272764	1
Rb1cc1	0.013901	0.806539	1
Adcy3	0.013865	0.680697	1
Lsm3	0.013864	0.096726	1
Srm	0.013862	0.29397	1
Polr2d	0.01386	0.098389	1
Btf3l4	0.013851	0.297253	1
Ppp2r3d	0.013851	0.875061	1
Hnrnpll	0.013841	0.183456	1
Slc30a4	0.01384	0.378209	1
Rexo4	0.01384	0.945403	1
Bzw1	0.013818	0.011483	1
2210016L21Rik	0.013814	0.618645	1
Slc2a5	0.013771	0.323345	1
Scai	0.013762	0.450608	1
Nfix	0.013756	0.411055	1
Laptm4b	0.013747	0.291472	1
Pura	0.013736	0.168735	1
Psmd7	0.01369	0.218566	1
1810058I24Rik	0.013688	0.21453	1
Inpp1	0.013669	0.986033	1
Bmi1	0.013658	0.654854	1
Dpy19l3	0.013646	0.402305	1
Nek6	0.013641	0.194361	1
Dffa	0.013626	0.12333	1
Zfpm1	0.013617	0.288519	1
Ric1	0.013617	0.985785	1
Hyou1	0.013608	0.667654	1
Poldip2	0.013593	0.785636	1
Cnot9	0.013582	0.401129	1
Slx1b	0.013565	0.245278	1
Plgrkt	0.013552	0.483467	1
Jpt1	0.013543	0.155446	1
St13	0.01354	0.098463	1
Mrpl23	0.013517	0.242067	1
Stk3	0.013509	0.817668	1
Nelfe	0.013477	0.154275	1
Ppp1r7	0.013444	0.339701	1
Cdk5rap3	0.01344	0.15481	1
Gm43464	0.013433	0.309708	1
Arhgap27	0.013432	0.395639	1
Farp1	0.013418	0.742413	1
Cmas	0.013413	0.913646	1
Adipor1	0.013391	0.077097	1
Ccdc88a	0.013373	0.116612	1

Tmem237	0.013371	0.222624	1
Kif5b	0.013361	0.518509	1
Slc16a3	0.013351	0.08106	1
Acsl1	0.013347	0.491215	1
Smim11	0.013342	0.141073	1
Mcts1	0.013338	0.22003	1
Mlxipl	0.013337	0.179798	1
Srsf5	0.013327	0.829394	1
Mrps7	0.013311	0.466338	1
Pafah1b3	0.013308	0.160698	1
Pgrmc1	0.013308	0.197176	1
Yipf5	0.013306	0.238436	1
Nphp1	0.013289	0.30388	1
Gm43466	0.013265	0.796612	1
Ndufb4	0.013257	0.43715	1
Kctd18	0.01324	0.510566	1
Etfb	0.013227	0.079592	1
Cysltr1	0.013213	0.633772	1
Isoc2a	0.013212	0.569742	1
Zfp961	0.013194	0.62278	1
Zhx3	0.013186	0.958846	1
Mef2a	0.013176	0.659625	1
Sh3glb1	0.013173	0.639512	1
Arfgap2	0.013165	0.046777	1
Dnajc2	0.013163	0.168887	1
Tyw5	0.013142	0.126037	1
Mrps36	0.013131	0.223825	1
Sclt1	0.01313	0.413254	1
Cpsf7	0.01313	0.494262	1
Rgs19	0.013109	0.08734	1
Stt3b	0.013096	0.278981	1
Etaa1	0.01309	0.919774	1
Atxn10	0.013061	0.20294	1
Naaa	0.013057	0.638582	1
Scoc	0.013052	0.331908	1
Wdr92	0.013038	0.976069	1
Med9	0.013038	0.709408	1
Wrap73	0.013038	0.674601	1
Mapre2	0.013001	0.253862	1
Psma7	0.01298	0.102715	1
Srebf2	0.012956	0.00922	1
Slc38a9	0.012933	0.830198	1
Aars	0.012931	0.697994	1
Rrm2b	0.012852	0.750071	1
4930556J24Rik	0.012847	0.219373	1
Myc	0.012814	0.79871	1
Leo1	0.012786	0.098425	1
Dcaf10	0.01278	0.773118	1

Trappc1	0.01278	0.337872	1
Rbmx	0.012762	0.492702	1
Atpaf1	0.01274	0.18169	1
Pdcd10	0.012738	0.272496	1
Pigc	0.012735	0.482042	1
Snn	0.012732	0.733916	1
Trmt6	0.012715	0.724973	1
Yju2	0.012703	0.241599	1
Mcm6	0.012698	0.262946	1
Ercc1	0.012674	0.204959	1
4931406C07Rik	0.012672	0.72228	1
Rab10os	0.012657	0.572662	1
Tnfaip3	0.012656	0.505254	1
Tmem106c	0.012629	0.197518	1
Gtpbp10	0.012629	0.868279	1
Msn	0.012607	0.300207	1
Cox20	0.012606	0.63087	1
Snx12	0.012598	0.895038	1
Utp14a	0.012594	0.570227	1
Git2	0.012588	0.46109	1
Gm16541	0.012586	0.722306	1
Dmac2	0.012565	0.331283	1
Nfic	0.012555	0.996987	1
Czib	0.012551	0.658303	1
Polr2i	0.012513	0.125122	1
Arf5	0.012487	0.63065	1
Rab33b	0.012473	0.24815	1
Smim19	0.012473	0.118326	1
Smim15	0.012453	0.757448	1
Arf6	0.012447	0.593058	1
Psmb10	0.012445	0.117447	1
Cdk10	0.012445	0.551736	1
Rpap1	0.012365	0.873732	1
Maip1	0.01236	0.616791	1
Arap2	0.012355	0.244085	1
Psmb1	0.012352	0.204812	1
Ssbp4	0.01235	0.245452	1
Mlh1	0.01235	0.567858	1
AW011738	0.012343	0.818559	1
Gtpbp3	0.012341	0.856041	1
Dhrs7b	0.01234	0.964351	1
C1d	0.012336	0.540293	1
Rab2a	0.012335	0.47775	1
Esd	0.012329	0.419105	1
Elp5	0.012309	0.348892	1
Tial1	0.012287	0.423615	1
Pnpla2	0.012284	0.404106	1
Nfx1	0.012282	0.815964	1

Ccdc51	0.01226	0.636553	1
2610001J05Rik	0.012255	0.200763	1
Herc2	0.012255	0.698671	1
Gtf3c4	0.012252	0.139398	1
Mrpl37	0.012248	0.586096	1
Alg1	0.012244	0.915764	1
Dolpp1	0.012235	0.119126	1
Dbi	0.012235	0.044578	1
Slc7a1	0.012227	0.126588	1
Gins1	0.012224	0.202553	1
Hsbp1	0.012205	0.310238	1
Zscan25	0.012191	0.099595	1
Trpv4	0.012173	0.201238	1
Psme2	0.012161	0.392994	1
Ormdl1	0.012155	0.017693	1
Ube2e3	0.012152	0.738816	1
Bet1l	0.012138	0.325114	1
B230307C23Rik	0.012128	0.427384	1
Entr1	0.012114	0.702028	1
Rgl3	0.012107	0.167353	1
Chm	0.012103	0.517321	1
Batf3	0.012087	0.720621	1
Gm39556	0.012086	0.661367	1
Abhd6	0.012081	0.174496	1
Casp3	0.012056	0.963082	1
Mrpl20	0.012045	0.064795	1
Hspbp1	0.012043	0.727109	1
Sema4a	0.012026	0.564098	1
Inka1	0.012023	0.108647	1
Lsm8	0.012002	0.281782	1
Pdcl3	0.011991	0.197522	1
Dnajc21	0.011977	0.234044	1
Mtf2	0.011949	0.29523	1
Rab24	0.011944	0.76883	1
Sun2	0.011931	0.379804	1
Cln8	0.011927	0.635448	1
Fem1b	0.011919	0.696889	1
Tiparp	0.011915	0.723506	1
Haus6	0.011892	0.157114	1
Pdcd6ip	0.011885	0.318302	1
Acvr2a	0.011869	0.688707	1
Axin1	0.011825	0.538375	1
Xylt2	0.011812	0.32971	1
Atmin	0.01181	0.276019	1
Scaper	0.011805	0.514284	1
Slc35a1	0.011801	0.175873	1
Erlin2	0.011795	0.161671	1
Rnf219	0.011787	0.681994	1

Zfp641	0.011787	0.529917	1
Uba2	0.011772	0.268325	1
Cox17	0.011769	0.053287	1
Zfp955a	0.011764	0.704169	1
Gm30329	0.011743	0.014777	1
Ddx52	0.01174	0.35113	1
Mrps14	0.011735	0.313841	1
Mmgt2	0.011728	0.42395	1
Bola2	0.011709	0.062515	1
Vegfb	0.011695	0.217284	1
Elp3	0.011684	0.365869	1
Zdhhc9	0.01168	0.295606	1
Zfp593	0.011677	0.126413	1
Rnpc3	0.011663	0.500527	1
Cdca7	0.011654	0.266044	1
Zfp52	0.011636	0.742837	1
Itgb2	0.011623	0.318702	1
Zfp993	0.011619	0.190142	1
Rnh1	0.011619	0.565036	1
Nsd2	0.011557	0.352117	1
Ephx1	0.011555	0.651518	1
Tsc22d2	0.011553	0.404909	1
Ube2n	0.01154	0.14878	1
Sema4c	0.011525	0.903262	1
Mrpl55	0.011519	0.413866	1
Dvl3	0.011518	0.334679	1
Srsf10	0.011517	0.507083	1
Ergic2	0.011511	0.151482	1
Wdsub1	0.01151	0.977146	1
Tradd	0.011509	0.77239	1
Pdlim5	0.011497	0.439461	1
Hnrnph1	0.011438	0.116468	1
Hint1	0.011437	0.048723	1
Rab18	0.011431	0.116767	1
Suv39h1	0.01142	0.503804	1
Arpc5l	0.011407	0.122006	1
Tarbp2	0.011399	0.539911	1
P2ry6	0.011397	0.288584	1
U2af2	0.011392	0.646493	1
Poglut3	0.011383	0.544051	1
Smap1	0.011376	0.722844	1
Alg9	0.011348	0.172717	1
Atrip	0.011332	0.411792	1
Mafk	0.011322	0.204602	1
Tnfaip8	0.011313	0.590725	1
Sbds	0.011298	0.682545	1
Trpm7	0.011284	0.071097	1
Cnp	0.011262	0.374127	1

Rai1	0.011254	0.349404	1
Inpp5f	0.011226	0.761468	1
Clcc1	0.011215	0.094856	1
C9orf72	0.011205	0.278773	1
Cript	0.011201	0.926221	1
Cstf2t	0.011193	0.167616	1
Dhrs1	0.011187	0.685317	1
Psmd12	0.011187	0.235538	1
Cd82	0.011177	0.192361	1
Gpatch3	0.011176	0.351419	1
Ccdc59	0.011175	0.032976	1
Enpp1	0.011168	0.700175	1
Mettl9	0.011141	0.333478	1
Afmid	0.011127	0.428878	1
Ly6m	0.011121	0.482932	1
Ciapin1	0.011094	0.590461	1
Chchd7	0.011093	0.107071	1
Rft1	0.011091	0.730412	1
1810030O07Rik	0.011074	0.57212	1
Heca	0.011061	0.246843	1
Sec13	0.011059	0.237468	1
Snhg5	0.011056	0.670746	1
Hook3	0.011052	0.823525	1
Cybb	0.011035	0.095724	1
Spns1	0.011033	0.136573	1
Polr3d	0.011022	0.384617	1
Nit1	0.010996	0.243739	1
2300009A05Rik	0.010986	0.115839	1
2610021A01Rik	0.010966	0.686874	1
Bptf	0.010963	0.586753	1
Sdhc	0.01096	0.305234	1
Hagh	0.010958	0.903459	1
Vbp1	0.010954	0.29742	1
Itgb1bp1	0.010911	0.146222	1
Zfp868	0.010908	0.519317	1
Ttyh3	0.010907	0.203126	1
1810037I17Rik	0.010894	0.037984	1
Xpnpep3	0.010881	0.837873	1
N4bp2	0.010878	0.461917	1
Ghdc	0.010877	0.603129	1
Alkbh5	0.010863	0.993029	1
Tmem38b	0.010854	0.44024	1
Unc50	0.01085	0.082877	1
Ttc4	0.010848	0.339457	1
Mfsd8	0.010834	0.49569	1
Ptrhd1	0.010834	0.592612	1
Scnm1	0.010825	0.319273	1
Dynlt3	0.010822	0.125199	1

Arvcf	0.01081	0.822662	1
Ormdl2	0.01081	0.028505	1
Oat	0.010804	0.31337	1
Arhgap29	0.010797	0.792	1
2310009B15Rik	0.010793	0.414875	1
Mrpl11	0.010791	0.120476	1
Jrkl	0.010785	0.562062	1
Cdk4	0.010781	0.798217	1
Traf2	0.010767	0.7892	1
Prpf31	0.010762	0.696883	1
Hspa8	0.010755	0.430442	1
Phpt1	0.010748	0.039892	1
Csgalnact2	0.01074	0.697194	1
Man2c1	0.010736	0.804628	1
Polr1d	0.010724	0.359502	1
Arpp19	0.010713	0.103067	1
Ghitm	0.01068	0.159349	1
Ube2m	0.010671	0.19589	1
Gm15964	0.010669	0.300924	1
Dohh	0.010662	0.432863	1
Zfp770	0.010636	0.13752	1
Dkc1	0.010633	0.785308	1
Eif2b2	0.01062	0.425261	1
Rps19bp1	0.010615	0.156153	1
Map1lc3a	0.010591	0.657947	1
Casc4	0.010583	0.320771	1
Psme3	0.010578	0.086462	1
Mcur1	0.010565	0.634869	1
Pfdn6	0.010563	0.587219	1
Zscan22	0.010559	0.712683	1
Ndufa3	0.010533	0.21732	1
Ctnnb1	0.010524	0.06152	1
Nap1l4	0.010521	0.903743	1
Lrig2	0.010514	0.904171	1
Psme1	0.010501	0.3248	1
Rhbdd2	0.010496	0.081155	1
Kri1	0.010495	0.493692	1
Zfp414	0.01049	0.615246	1
Mllt1	0.010489	0.874654	1
Tex264	0.010485	0.260854	1
Alg3	0.010478	0.53462	1
Rabggtb	0.010465	0.746896	1
Cdc5l	0.010464	0.796369	1
Tefm	0.010459	0.549195	1
Gdpd5	0.010458	0.580129	1
Zfas1	0.010449	0.150802	1
Fam122a	0.010438	0.162372	1
6430590A07Rik	0.010408	0.166473	1

Mrgbp	0.0104	0.958104	1
Ilvbl	0.010396	0.315036	1
Smug1	0.010392	0.676547	1
Endod1	0.010391	0.25737	1
Cox11	0.010368	0.348794	1
Sec24d	0.010366	0.744235	1
Uqcc2	0.010353	0.427136	1
Vps35	0.01035	0.46644	1
Nudt5	0.010322	0.309133	1
Adgra3	0.01032	0.043835	1
Olfml2b	0.010316	0.410169	1
Pigq	0.010295	0.103088	1
Oxld1	0.010293	0.327179	1
Pfn1	0.010271	0.297852	1
Ammecr1l	0.01026	0.965291	1
Esco1	0.010244	0.229792	1
6230400D17Rik	0.010236	0.221945	1
Lzic	0.010222	0.890142	1
Csnk2a1	0.010219	0.377189	1
Parp6	0.010208	0.953549	1
Cyp2r1	0.010193	0.043269	1
Umps	0.010181	0.595717	1
Mboat7	0.010167	0.356057	1
Dele1	0.010167	0.851246	1
Trabd	0.010156	0.256735	1
Rundc1	0.010148	0.634722	1
Pbx1	0.010119	0.797296	1
Pigx	0.010084	0.692564	1
Mrps10	0.010079	0.167837	1
Ndufb6	0.01006	0.277016	1
Gm43462	0.010057	0.035785	1
Trmt12	0.010044	0.708207	1
Gm17066	0.010037	0.446361	1
Trappc2	0.010032	0.200657	1
Abhd10	0.010018	0.850824	1
Chst1	0.010009	0.286123	1
Prrc2b	-0.01	0.740367	1
Acyp1	-0.01001	0.252092	1
Cracr2b	-0.01001	0.583314	1
Pts	-0.01001	0.942703	1
Ttc38	-0.01001	0.596399	1
Nkapd1	-0.01003	0.34888	1
Cklf	-0.01003	0.655762	1
Zswim3	-0.01003	0.160172	1
Znhit6	-0.01003	0.21932	1
Scyl2	-0.01004	0.782934	1
Pafah1b2	-0.01004	0.826649	1
Lyar	-0.01006	0.741915	1

Rnf17	-0.01006	0.957827	1
BC005561	-0.01006	0.277931	1
Prdm9	-0.01007	0.19886	1
Azi2	-0.01009	0.944237	1
Klhl24	-0.0101	0.697379	1
Rdh5	-0.0101	0.437845	1
Rdh11	-0.01011	0.507284	1
Exosc7	-0.01012	0.459489	1
Gcsh	-0.01016	0.950244	1
Arglu1	-0.01016	0.79113	1
Mtmr9	-0.01017	0.350588	1
Dnase1l1	-0.01018	0.288878	1
Akr1e1	-0.0102	0.9266	1
Setd4	-0.01021	0.319539	1
Mtor	-0.01024	0.674742	1
Lin52	-0.01025	0.679896	1
Trim3	-0.01026	0.164368	1
Mcph1	-0.01027	0.4496	1
Gtf3c1	-0.01027	0.887041	1
Gm32591	-0.01028	0.461853	1
Hnrnpr	-0.01028	0.975805	1
Rbfa	-0.01028	0.788909	1
Tirap	-0.01028	0.491925	1
Rbm41	-0.01029	0.28686	1
Insig2	-0.01033	0.86037	1
Abhd14a	-0.01033	0.346371	1
Nde1	-0.01033	0.897234	1
Usp8	-0.01035	0.505663	1
Ints8	-0.01035	0.432768	1
Tcp11l2	-0.01036	0.595516	1
Gemin2	-0.01037	0.228291	1
Bud23	-0.01037	0.362855	1
Mrpl24	-0.01038	0.960668	1
4933412E12Rik	-0.0104	0.878171	1
Btbd8	-0.0104	0.529926	1
Lgals9	-0.01041	0.992134	1
Usf1	-0.01044	0.740549	1
Zdhhc8	-0.01044	0.360426	1
Oaz2	-0.01046	0.348044	1
Sdhaf1	-0.01046	0.913227	1
AU022252	-0.01047	0.757012	1
Hpf1	-0.01048	0.315766	1
Usp47	-0.01049	0.331098	1
Snrpd2	-0.01049	0.60669	1
Faap100	-0.01049	0.267079	1
1600020E01Rik	-0.01049	0.505737	1
Pknox1	-0.0105	0.443464	1
Mypopos	-0.0105	0.764359	1

Taf6	-0.0105	0.936174	1
Mphosph9	-0.01051	0.586262	1
Jarid2	-0.01052	0.301883	1
Sp140	-0.01052	0.658965	1
A630001O12Rik	-0.01053	0.23986	1
Borcs5	-0.01054	0.150137	1
Ppp2ca	-0.01054	0.805001	1
Gclc	-0.01054	0.569407	1
Kansl2	-0.01054	0.528795	1
Kctd3	-0.01055	0.643127	1
Smarca2	-0.01055	0.645955	1
Cmya5	-0.01056	0.109645	1
Rad21	-0.01056	0.89996	1
Ppp1r8	-0.01056	0.723202	1
Scyl1	-0.01057	0.480221	1
Scarb1	-0.01058	0.854982	1
Cdk16	-0.01058	0.812533	1
Rrs1	-0.01059	0.370477	1
Cgas	-0.0106	0.140237	1
Gm11713	-0.01064	0.148883	1
Sod2	-0.01065	0.930863	1
Sarnp	-0.01065	0.979539	1
Nop58	-0.01066	0.741387	1
Galnt11	-0.01067	0.588677	1
Yme1l1	-0.01068	0.655695	1
Zfp260	-0.01073	0.911464	1
Cluap1	-0.01073	0.886835	1
Rcn1	-0.01073	0.745098	1
Flcn	-0.01074	0.487804	1
Zfp384	-0.01075	0.930447	1
Gna13	-0.01076	0.310594	1
Ctsc	-0.01077	0.527199	1
Ythdf2	-0.01078	0.561717	1
Akt1s1	-0.01078	0.387667	1
Kxd1	-0.01079	0.979268	1
Crkl	-0.01079	0.341159	1
Trappc3	-0.0108	0.390707	1
Fdx2	-0.01081	0.962058	1
2810006K23Rik	-0.01081	0.556331	1
Ppip5k1	-0.01081	0.128219	1
Pctp	-0.01081	0.568744	1
Aldh3a2	-0.01082	0.922796	1
Fubp3	-0.01083	0.701098	1
Rab9	-0.01083	0.782424	1
Neurl4	-0.01084	0.396168	1
Abi2	-0.01086	0.627309	1
Bckdha	-0.01087	0.302437	1
Gm16201	-0.01088	0.059938	1

Rap2c	-0.01088	0.682606	1
Nono	-0.01088	0.686616	1
Faf2	-0.01089	0.150045	1
Rab31	-0.01089	0.862102	1
Sqor	-0.0109	0.61849	1
Rpl10-ps3	-0.0109	0.326868	1
Gbgt1	-0.01094	0.487032	1
Narf	-0.01095	0.601804	1
BC002059	-0.01095	0.429148	1
Fbl	-0.01095	0.980642	1
Apeh	-0.01095	0.474443	1
Ccdc58	-0.01095	0.740083	1
Usp12	-0.01097	0.517732	1
Szt2	-0.01097	0.348708	1
Mettl7a1	-0.01098	0.328567	1
Chpf2	-0.01098	0.34179	1
Grpel2	-0.01099	0.930842	1
H2-DMa	-0.01099	0.811661	1
Cyb5r1	-0.01099	0.508653	1
Dnajc4	-0.011	0.913278	1
Xbp1	-0.01102	0.753141	1
Smim20	-0.01104	0.985734	1
Slx4	-0.01104	0.305282	1
Actr8	-0.01105	0.434538	1
Hectd3	-0.01105	0.321265	1
Ints4	-0.01109	0.990742	1
Hspb11	-0.0111	0.619436	1
Prkd2	-0.01111	0.492099	1
Rcc2	-0.01111	0.65403	1
Ccdc61	-0.01111	0.374154	1
Zfp90	-0.01111	0.718318	1
Gpatch4	-0.01112	0.225877	1
Sec31a	-0.01112	0.926934	1
B4galnt1	-0.01113	0.888287	1
Slc25a36	-0.01113	0.779186	1
Ppp2r5d	-0.01114	0.19459	1
Mtus1	-0.01114	0.928435	1
Tifab	-0.01116	0.55724	1
Sipa1	-0.01119	0.705492	1
Zdhhc7	-0.0112	0.679182	1
Zfp788	-0.01122	0.14934	1
Cant1	-0.01122	0.67362	1
Abraxas1	-0.01122	0.411103	1
Dnajc10	-0.01123	0.970045	1
Hibadh	-0.01131	0.899945	1
Gsk3b	-0.01132	0.775227	1
Cd46	-0.01134	0.957455	1
Ppp1r16a	-0.01134	0.288817	1

Eif2d	-0.01134	0.452775	1
Tmem184c	-0.01135	0.196244	1
Rnf44	-0.01135	0.835117	1
Pitpna	-0.01136	0.794475	1
4933434E20Rik	-0.01136	0.932232	1
Rab5b	-0.01138	0.626697	1
Gosr1	-0.01138	0.38715	1
Slc27a1	-0.01139	0.273137	1
Abl2	-0.01139	0.458928	1
2210016F16Rik	-0.0114	0.679501	1
Ankrd13d	-0.0114	0.278104	1
Aar2	-0.0114	0.276076	1
Siah2	-0.0114	0.406563	1
Srp68	-0.01142	0.47576	1
Drosha	-0.01142	0.586735	1
Itprid2	-0.01143	0.729203	1
Hps3	-0.01145	0.555271	1
Tmem39a	-0.01146	0.70355	1
Mapk1ip1	-0.01148	0.975982	1
Capn3	-0.01149	0.385849	1
Impdh2	-0.01151	0.534511	1
Rmi1	-0.01154	0.288486	1
Klhl26	-0.01156	0.617299	1
Prkar2a	-0.01158	0.524353	1
Lrrc14	-0.0116	0.339951	1
Cdadc1	-0.01162	0.589545	1
Ascc2	-0.01162	0.999487	1
Med25	-0.01163	0.700977	1
Ddx6	-0.01164	0.671556	1
Gm26672	-0.01164	0.782997	1
Dera	-0.01164	0.452724	1
Klhl36	-0.01165	0.712351	1
Plekhn1	-0.01174	0.61213	1
Hs6st1	-0.01174	0.3714	1
Sel1l	-0.01175	0.898739	1
Mfsd4b4	-0.01175	0.559033	1
Ulbp1	-0.01175	0.09552	1
Pigg	-0.01176	0.030876	1
Tesk2	-0.01176	0.716872	1
Hook2	-0.01179	0.928078	1
Bysl	-0.01179	0.47357	1
Esrra	-0.01181	0.303193	1
Coasy	-0.01181	0.928359	1
Lsm10	-0.01182	0.468199	1
Zfp607a	-0.01182	0.618673	1
Rasal3	-0.01183	0.390171	1
Tfrc	-0.01183	0.584133	1
Tmem39b	-0.01185	0.539372	1

Hmg20b	-0.01186	0.863117	1
Triap1	-0.01187	0.363501	1
Hes6	-0.01189	0.80104	1
Supt5	-0.01189	0.554708	1
Mrpl34	-0.01191	0.721122	1
C130050O18Rik	-0.01192	0.572217	1
Lbr	-0.01193	0.327693	1
Dpm2	-0.01196	0.803132	1
Clp1	-0.01196	0.165382	1
BC004004	-0.01197	0.467952	1
Uvssa	-0.01198	0.500699	1
Zfyve19	-0.01198	0.673569	1
Cep135	-0.01198	0.833625	1
Zfp326	-0.01199	0.986262	1
Clk3	-0.012	0.540471	1
Zfp512b	-0.01201	0.447456	1
Lamtor1	-0.01201	0.712963	1
Panx1	-0.01202	0.165889	1
Psmd5	-0.01202	0.393011	1
A530017D24Rik	-0.01203	0.073334	1
Dynlt1f	-0.01205	0.785676	1
Cox14	-0.01207	0.941967	1
Dnaaf5	-0.01207	0.19314	1
Mrps26	-0.01207	0.305272	1
Tsr3	-0.01208	0.505685	1
Bcl2l12	-0.01209	0.106056	1
Fggy	-0.01209	0.720927	1
Rbbp4	-0.0121	0.683512	1
9530062K07Rik	-0.01211	0.487583	1
Eif3d	-0.01211	0.927054	1
Ppp2r5a	-0.01212	0.573835	1
Prrc1	-0.01214	0.368494	1
B230219D22Rik	-0.01215	0.865782	1
Batf	-0.01215	0.446381	1
Cnksr3	-0.01215	0.576666	1
Tmem156	-0.01216	0.248538	1
Rfxap	-0.01218	0.51062	1
AW549877	-0.01219	0.43084	1
Lclat1	-0.01219	0.456792	1
4930549G23Rik	-0.0122	0.26695	1
Cdc42se1	-0.01222	0.255953	1
Ubxn2b	-0.01222	0.817533	1
Inppl1	-0.01228	0.370686	1
Aste1	-0.01229	0.175667	1
Rcc1	-0.0123	0.404277	1
Med8	-0.01231	0.299541	1
1500011B03Rik	-0.01232	0.641869	1
S100a10	-0.01234	0.923202	1

Slc25a19	-0.01235	0.038739	1
Dip2b	-0.01236	0.577832	1
Flna	-0.01236	0.705867	1
Nat10	-0.01237	0.69532	1
Smox	-0.01237	0.536828	1
Med28	-0.01238	0.652103	1
Zc3hc1	-0.01239	0.043276	1
Wdr82	-0.01239	0.598423	1
Gm32036	-0.0124	0.283779	1
Snx8	-0.01241	0.993261	1
Rgp1	-0.01241	0.600621	1
Itga5	-0.01241	0.955168	1
Il10rb	-0.01243	0.8703	1
1700123O20Rik	-0.01243	0.670944	1
Exosc8	-0.01244	0.587789	1
Slc35e3	-0.01245	0.623346	1
Zfp87	-0.0125	0.008462	1
Klhl23	-0.0125	0.247406	1
Cyren	-0.01251	0.127113	1
Polr1b	-0.01252	0.18168	1
Prkcq	-0.01253	0.775614	1
Zfp821	-0.01254	0.545155	1
Pitpnm1	-0.01256	0.833881	1
Nsmf	-0.01256	0.079322	1
Yae1d1	-0.01256	0.549916	1
Trim35	-0.01257	0.900508	1
Tulp3	-0.01257	0.196806	1
Ndufaf7	-0.01257	0.652595	1
Tmem150a	-0.01259	0.649734	1
Pus1	-0.0126	0.507891	1
Sp3os	-0.01262	0.67838	1
Zfp956	-0.01262	0.040029	1
Slc35f6	-0.01264	0.834382	1
Cdk2	-0.01266	0.373581	1
Cops5	-0.01266	0.328418	1
Ndufa9	-0.01268	0.688853	1
Ahdc1	-0.01269	0.474867	1
Gm50012	-0.01271	0.977642	1
Gprasp1	-0.01271	0.285103	1
Cmtr1	-0.01272	0.251659	1
Tlr3	-0.01272	0.862162	1
Ubxn8	-0.01272	0.624047	1
Tmem154	-0.01274	0.306689	1
Magohb	-0.01274	0.271246	1
Tnni2	-0.01276	0.876538	1
Arhgap9	-0.0128	0.952445	1
Psme4	-0.0128	0.985484	1
Agpat5	-0.01281	0.861195	1

Irak1	-0.01282	0.885607	1
Gpr137	-0.01282	0.670224	1
Bloc1s6	-0.01282	0.407818	1
Abracl	-0.01282	0.676257	1
Cbx1	-0.01283	0.674836	1
Klc2	-0.01283	0.693198	1
Foxo1	-0.01285	0.836153	1
P3h3	-0.01286	0.921329	1
Ptger4	-0.01286	0.583959	1
Phyh	-0.01287	0.924844	1
Lmbrd1	-0.0129	0.412532	1
Vps9d1	-0.01291	0.718602	1
Rab14	-0.01292	0.53433	1
Zfp608	-0.01295	0.366057	1
Wdr75	-0.01298	0.748187	1
Lncppara	-0.01298	0.572997	1
Ggnbp2	-0.01298	0.798828	1
Arap1	-0.01298	0.356848	1
Ankrd13a	-0.01299	0.885931	1
AI480526	-0.01301	0.250904	1
Eci1	-0.01302	0.803021	1
Isoc1	-0.01303	0.740346	1
Ube2v2	-0.01304	0.540693	1
Med6	-0.01306	0.58892	1
Lrrk1	-0.01307	0.791112	1
Gtf2h1	-0.01309	0.130661	1
Phactr2	-0.01309	0.673817	1
Fam160a2	-0.0131	0.169944	1
Dubr	-0.0131	0.423743	1
Usp42	-0.0131	0.053767	1
Vamp8	-0.01311	0.933544	1
Zranb2	-0.01312	0.77232	1
Mrpl4	-0.01313	0.954596	1
Dolk	-0.01315	0.148533	1
Ubp1	-0.01316	0.949978	1
Acrbp	-0.01317	0.305486	1
Qars	-0.01318	0.40009	1
Fbxo45	-0.01319	0.383723	1
Tipin	-0.01319	0.900615	1
Plin3	-0.01321	0.764335	1
Zfp429	-0.01322	0.280698	1
Gm10125	-0.01323	0.960981	1
Ndufs4	-0.01323	0.762853	1
Grk5	-0.01324	0.06751	1
Nr2f6	-0.01324	0.536637	1
Cbr4	-0.01325	0.408492	1
Nr1h2	-0.01325	0.650072	1
Adam10	-0.01327	0.76348	1

Ppm1d	-0.01328	0.686555	1
E2f3	-0.01328	0.566971	1
Thoc3	-0.0133	0.089717	1
Cd2bp2	-0.01331	0.636222	1
Btbd2	-0.01331	0.491614	1
Tmem165	-0.01332	0.519972	1
Zfp748	-0.01332	0.75106	1
Naf1	-0.01333	0.920566	1
Dgka	-0.01335	0.511505	1
Agl	-0.01335	0.47594	1
Ipo13	-0.01335	0.356258	1
Tdp1	-0.01336	0.926869	1
Fbxl17	-0.01336	0.667874	1
Atp5l	-0.01336	0.550984	1
Sirt3	-0.01337	0.949917	1
Stoml1	-0.01338	0.493464	1
Med20	-0.01338	0.640267	1
Cep83os	-0.01339	0.386564	1
Dnmt1	-0.0134	0.713272	1
Atp5c1	-0.01342	0.977003	1
Abi1	-0.01343	0.73645	1
Rab1b	-0.01344	0.81805	1
Zscan29	-0.01344	0.197666	1
Fra10ac1	-0.01346	0.619829	1
Ergic1	-0.01346	0.429911	1
Nt5c3	-0.01347	0.446854	1
Mrpl53	-0.01347	0.30132	1
Gm15635	-0.01349	0.377099	1
Rbm19	-0.01349	0.141766	1
Pbx3	-0.0135	0.615875	1
Rpgrip1	-0.0135	0.287217	1
Mvp	-0.01352	0.402202	1
Dhx35	-0.01352	0.282146	1
Rap1a	-0.01352	0.356342	1
Gm43331	-0.01353	0.081143	1
Ino80b	-0.01353	0.668858	1
Wdr35	-0.01354	0.189181	1
Pmpcb	-0.01355	0.986079	1
Mrpl47	-0.01355	0.171473	1
Rnf24	-0.01356	0.446656	1
Sfswap	-0.01357	0.092229	1
Nup50	-0.01359	0.371834	1
Kpna1	-0.01362	0.35738	1
2310061I04Rik	-0.01362	0.060446	1
Hmbox1	-0.01363	0.369804	1
Ndor1	-0.01364	0.34105	1
Hpn	-0.01364	0.797761	1
Slc38a1	-0.01364	0.956667	1

Bnip2	-0.01367	0.826642	1
Gm27010	-0.01367	0.013124	1
Ticam1	-0.01367	0.969578	1
Il1rl2	-0.01367	0.193214	1
Slc26a2	-0.0137	0.506153	1
Jmy	-0.0137	0.385151	1
Ncoa6	-0.01372	0.36157	1
Suz12	-0.01372	0.482075	1
Simc1	-0.01375	0.894369	1
Pip4p1	-0.01375	0.914559	1
Trmt10c	-0.01376	0.632064	1
Cox10	-0.01377	0.113954	1
Gstcd	-0.01378	0.705979	1
Usp6nl	-0.01378	0.990444	1
Gigyf2	-0.01379	0.40802	1
Kras	-0.01379	0.914541	1
Mbd3	-0.0138	0.944915	1
Abcb10	-0.01381	0.04283	1
Hdhd2	-0.01381	0.753147	1
Pkdcc	-0.01383	0.628955	1
Prune1	-0.01384	0.608664	1
Zpr1	-0.01387	0.88198	1
Atp5g3	-0.01387	0.77821	1
Qsox1	-0.01387	0.33949	1
Metap1d	-0.01388	0.6563	1
Siah1a	-0.01388	0.453274	1
Gm28417	-0.01389	0.454149	1
Tcf12	-0.01389	0.728058	1
Gm15513	-0.01389	0.21488	1
Tor4a	-0.0139	0.325972	1
Copb2	-0.01391	0.470431	1
Gm19605	-0.01392	0.174876	1
Kat6a	-0.01393	0.978148	1
Gm11084	-0.01395	0.492618	1
Sv2a	-0.01397	0.399822	1
Rcc1l	-0.01397	0.870624	1
Lias	-0.01397	0.646586	1
Zfp212	-0.01397	0.773845	1
Rybp	-0.01399	0.641346	1
Bcl2a1a	-0.01399	0.893552	1
Mapre3	-0.01399	0.261896	1
Cdk9	-0.014	0.672705	1
Ppfia4	-0.01401	0.535558	1
Fbxw8	-0.01402	0.165373	1
Brpf1	-0.01403	0.654685	1
Eri1	-0.01407	0.65099	1
Casc3	-0.01408	0.445656	1
Ticam2	-0.01408	0.289238	1

Cdkn2aip	-0.01409	0.513474	1
Prickle1	-0.01409	0.314755	1
Tra2b	-0.0141	0.489699	1
Il1rap	-0.01414	0.742416	1
Gm11973	-0.01414	0.295733	1
Acvr1b	-0.01417	0.271849	1
Ube2d2a	-0.01417	0.818779	1
Adgb	-0.01419	0.218329	1
Slc41a3	-0.01419	0.92859	1
Ctps	-0.0142	0.76268	1
Dhdh	-0.01421	0.10132	1
Qser1	-0.01421	0.6042	1
2610008E11Rik	-0.01423	0.394376	1
Zbtb7b	-0.01424	0.11674	1
Tnfsf8	-0.01427	0.614063	1
Gatad1	-0.0143	0.644828	1
Degs2	-0.01431	0.112756	1
Mms22l	-0.01431	0.150745	1
Edrf1	-0.01432	0.239643	1
Zhx2	-0.01434	0.695602	1
Trdmt1	-0.01434	0.296469	1
Mettl16	-0.01435	0.770702	1
Rhbdd3	-0.01437	0.315234	1
Sdad1	-0.01439	0.640912	1
Hexim1	-0.0144	0.404355	1
Sf3a1	-0.0144	0.507737	1
Ubtf	-0.0144	0.822681	1
Snhg3	-0.01443	0.648103	1
Sirt2	-0.01443	0.562027	1
Pot1b	-0.01443	0.48679	1
Gas8	-0.01444	0.254695	1
Cmtm4	-0.01444	0.566138	1
Slc35c1	-0.01445	0.586855	1
5530601H04Rik	-0.01445	0.208427	1
Wdr74	-0.01445	0.147294	1
Ppa1	-0.01445	0.690893	1
Wdr5	-0.01446	0.283038	1
Slc8a1	-0.01446	0.394495	1
Pdyn	-0.01448	0.44876	1
Gpam	-0.01452	0.592606	1
Mbtps1	-0.01452	0.875465	1
Card6	-0.01453	0.144815	1
Rcbtb1	-0.01454	0.447284	1
Crtc2	-0.01455	0.089335	1
Naip2	-0.01456	0.257005	1
Mras	-0.01458	0.770769	1
Homer1	-0.01459	0.178593	1
Mettl4	-0.01459	0.699371	1

Ube3a	-0.01459	0.518662	1
Ccnt2	-0.0146	0.958535	1
Acad11	-0.0146	0.86414	1
Otulin	-0.01463	0.980176	1
Ube2l6	-0.01465	0.34299	1
Wdr4	-0.01465	0.970553	1
Osgepl1	-0.01466	0.466351	1
Polr1c	-0.01467	0.478116	1
Mospd1	-0.01469	0.257861	1
Sf3b2	-0.0147	0.941596	1
Rxrb	-0.01471	0.537542	1
Rae1	-0.01471	0.385533	1
Camta2	-0.01471	0.495371	1
Mfsd11	-0.01472	0.291377	1
Fam117a	-0.01474	0.090798	1
Tut1	-0.01474	0.032331	1
Rtca	-0.01474	0.968292	1
Gclm	-0.01474	0.174383	1
Thap3	-0.01476	0.772676	1
Itpr2	-0.01477	0.466307	1
Dip2a	-0.01477	0.783088	1
Nubp2	-0.01478	0.402421	1
Htra3	-0.01478	0.232516	1
Gpr18	-0.01478	0.486834	1
Abcc3	-0.01479	0.679369	1
Rapsn	-0.0148	0.758578	1
4930517O19Rik	-0.0148	0.883626	1
Arhgdib	-0.01481	0.401042	1
Ccdc73	-0.01482	0.395939	1
Trp53rka	-0.01485	0.291668	1
Slc31a2	-0.01487	0.929019	1
Klf3	-0.01487	0.817808	1
Chchd6	-0.01487	0.427642	1
9430091E24Rik	-0.01488	0.581073	1
Dgkh	-0.0149	0.490421	1
Htra2	-0.0149	0.591985	1
Ece1	-0.0149	0.235932	1
Chst7	-0.0149	0.954449	1
Cbx7	-0.01491	0.073294	1
Blnk	-0.01491	0.404246	1
Psmb9	-0.01491	0.682268	1
Dcps	-0.01491	0.316862	1
Cipc	-0.01491	0.137389	1
Ubxn7	-0.01492	0.465535	1
Sbf2	-0.01493	0.728747	1
Snx15	-0.01495	0.890242	1
2310058D17Rik	-0.01496	0.011313	1
Vps29	-0.01496	0.592728	1

Gpn1	-0.01497	0.055342	1
Gm13269	-0.01497	0.993892	1
Skiv2l	-0.01497	0.110637	1
Fxr1	-0.01497	0.660813	1
Gpatch2l	-0.01498	0.496607	1
Hdac1	-0.01499	0.901033	1
Sgsm3	-0.01499	0.111845	1
Ftsj3	-0.01499	0.411766	1
Parp16	-0.01499	0.937717	1
Pop5	-0.015	0.380316	1
C2cd2l	-0.015	0.215264	1
Clk4	-0.01501	0.333367	1
Zfp426	-0.01501	0.260668	1
Zfp1	-0.01501	0.185304	1
Pced1b	-0.01501	0.839643	1
Wdr11	-0.01504	0.336981	1
Cdan1	-0.01505	0.212323	1
Stk25	-0.01506	0.111014	1
Wrn	-0.01507	0.753552	1
Cryzl2	-0.01509	0.624702	1
Ly9	-0.0151	0.495563	1
Vps72	-0.01511	0.511705	1
Ikbip	-0.01512	0.084138	1
Vps11	-0.01512	0.579564	1
Pa2g4	-0.01512	0.838942	1
Ms4a6b	-0.01513	0.954437	1
Setdb2	-0.01513	0.016013	1
Mipep	-0.01514	0.505914	1
Senp7	-0.01515	0.144781	1
Gm28198	-0.01515	0.703615	1
Adnp2	-0.01516	0.485848	1
Gm32856	-0.01516	0.580406	1
Rps6kc1	-0.01516	0.762743	1
Fam13a	-0.01517	0.218059	1
Urm1	-0.01518	0.733357	1
Acss1	-0.0152	0.460826	1
Sec16a	-0.01521	0.939268	1
Dpp7	-0.01521	0.971998	1
Akr1a1	-0.01522	0.682811	1
Kpna6	-0.01524	0.584979	1
Fmn1	-0.01524	0.867137	1
Epha2	-0.01524	0.664658	1
Kars	-0.01525	0.320882	1
Hdac3	-0.01525	0.738174	1
Chac2	-0.01526	0.02113	1
D6Wsu163e	-0.01526	0.424001	1
Qsox2	-0.01527	0.161615	1
Tm9sf1	-0.01528	0.728989	1

C130026I21Rik	-0.01528	0.193285	1
Mlh3	-0.01528	0.092058	1
Tcf25	-0.01529	0.871778	1
Parvg	-0.01531	0.852468	1
Pex3	-0.01533	0.735774	1
Tom1l1	-0.01534	0.938426	1
Sgcb	-0.01535	0.500302	1
Epc2	-0.01536	0.802241	1
Pdcd2	-0.01536	0.939641	1
Smc2	-0.01537	0.887832	1
Sec23a	-0.01538	0.551386	1
Rac2	-0.01539	0.494925	1
Diaph2	-0.0154	0.432002	1
H2-Ob	-0.0154	0.660855	1
Eif4g2	-0.0154	0.84768	1
Gtpbp6	-0.01542	0.6046	1
Sfxn2	-0.01543	0.296773	1
Baz2a	-0.01545	0.189053	1
Phf10	-0.01545	0.207712	1
Rnf8	-0.01546	0.935957	1
1810013L24Rik	-0.01546	0.856373	1
Furin	-0.01548	0.728633	1
Glb1l	-0.01548	0.370414	1
Gm28791	-0.01548	0.134627	1
Ppt1	-0.01549	0.799157	1
Smyd3	-0.01549	0.169694	1
D130062J10Rik	-0.0155	0.205342	1
Ninl	-0.0155	0.398358	1
Mta2	-0.0155	0.688895	1
Gga1	-0.01551	0.751226	1
Rnf168	-0.01551	0.454575	1
Tbc1d25	-0.01551	0.041044	1
Ctse	-0.01555	0.213763	1
Ofd1	-0.01556	0.346606	1
Dcaf8	-0.01556	0.127335	1
Fbxo25	-0.01556	0.755856	1
Atg9a	-0.01556	0.601991	1
Rbm12b2	-0.01557	0.32413	1
Mfn2	-0.01558	0.854457	1
Gpcpd1	-0.01558	0.902497	1
Ube2v1	-0.01559	0.942299	1
Xrcc6	-0.0156	0.300901	1
Eya4	-0.0156	0.931441	1
Brwd1	-0.0156	0.74548	1
Lat2	-0.01561	0.992207	1
Patl2	-0.01561	0.914844	1
Trnt1	-0.01561	0.308672	1
Urb1	-0.01562	0.518162	1

Stk16	-0.01562	0.06357	1
Usp54	-0.01563	0.326242	1
Ensa	-0.01564	0.936083	1
Top2b	-0.01564	0.87165	1
Zfp141	-0.01565	0.690718	1
Gm7030	-0.01565	0.024114	1
Trappc9	-0.01566	0.372077	1
Lrrc29	-0.01568	0.31472	1
Auh	-0.01571	0.701651	1
Api5	-0.01572	0.966984	1
Trim30d	-0.01572	0.158483	1
Cds1	-0.01574	0.511045	1
Mrpl43	-0.01574	0.75442	1
Zfp984	-0.01576	0.199492	1
March3	-0.01576	0.206799	1
Ints10	-0.01576	0.827759	1
Slc38a2	-0.01577	0.750348	1
Rpf2	-0.01578	0.651293	1
Pnn	-0.01578	0.380031	1
Mfhas1	-0.0158	0.726703	1
Rad54l2	-0.01581	0.169053	1
Adprhl2	-0.01582	0.834867	1
Mrpl38	-0.01582	0.579246	1
Zrsr2	-0.01583	0.803541	1
Stard5	-0.01584	0.132557	1
Psd4	-0.01586	0.846664	1
Hacd4	-0.01587	0.701054	1
Rsf1	-0.01587	0.511191	1
Snx13	-0.01587	0.578551	1
Clasp2	-0.01589	0.812438	1
E430024I08Rik	-0.01589	0.361951	1
Creb3l2	-0.01589	0.538106	1
Sco2	-0.0159	0.647644	1
Nup85	-0.0159	0.016969	1
Pofut2	-0.01591	0.51517	1
Cenpj	-0.01592	0.135887	1
Il17ra	-0.01592	0.992266	1
5830432E09Rik	-0.01592	0.136613	1
Smad2	-0.01592	0.334149	1
0610009B22Rik	-0.01593	0.565087	1
Ankrd26	-0.01594	0.161705	1
Otub1	-0.01595	0.766921	1
Sap130	-0.01597	0.27666	1
Pyroxd1	-0.01597	0.560451	1
Rhno1	-0.01597	0.231889	1
Zbtb14	-0.01597	0.302966	1
Tmem203	-0.01598	0.615046	1
Slc25a17	-0.01598	0.439782	1

4930473A02Rik	-0.01598	0.507792	1
Ccdc117	-0.01598	0.115575	1
Smcr8	-0.01601	0.420903	1
Bms1	-0.01601	0.475776	1
Fbxw2	-0.01601	0.548719	1
Fbxo6	-0.01604	0.563679	1
Crbn	-0.01604	0.370242	1
Vps41	-0.01604	0.793661	1
Ccdc171	-0.01605	0.937591	1
Uspl1	-0.01606	0.552397	1
Polg	-0.01607	0.834264	1
Cic	-0.01609	0.525087	1
Nkiras1	-0.01612	0.410371	1
Gatd1	-0.01612	0.401932	1
Cfap97	-0.01613	0.588164	1
Rad51d	-0.01614	0.050078	1
Mb21d2	-0.01615	0.056655	1
Zbtb8os	-0.01616	0.983158	1
Tcp11l1	-0.01616	0.561836	1
Ddb2	-0.01616	0.007998	1
Gnpda1	-0.01617	0.986575	1
Svil	-0.01618	0.457234	1
Fbxw5	-0.01618	0.884299	1
Zfp830	-0.0162	0.579425	1
Mks1	-0.01621	0.448647	1
Zbtb17	-0.01622	0.640636	1
Gm13708	-0.01622	0.237365	1
Polr2g	-0.01622	0.831553	1
Gm29488	-0.01622	0.367478	1
2900005J15Rik	-0.01624	0.024606	1
Tsc2	-0.01625	0.758011	1
Pwwp3a	-0.01625	0.120001	1
Gm17268	-0.01626	0.032092	1
Ppp6c	-0.01628	0.744351	1
Timm44	-0.01629	0.408812	1
Brap	-0.0163	0.172293	1
Gar1	-0.0163	0.538435	1
Psmd14	-0.01631	0.636025	1
Tmem205	-0.01631	0.46	1
Cdc26	-0.01632	0.77222	1
Fam129b	-0.01632	0.064482	1
Eloa	-0.01633	0.896917	1
0610030E20Rik	-0.01635	0.655464	1
Uhrf1bp1	-0.01635	0.827599	1
Spsb3	-0.01635	0.363018	1
Gm36198	-0.01636	0.842059	1
Bsdc1	-0.01637	0.103419	1
Mcat	-0.0164	0.437843	1

Ankrd50	-0.01643	0.414332	1
Pias4	-0.01643	0.093189	1
Ulk1	-0.01643	0.25971	1
Zfp983	-0.01645	0.340477	1
Sh3gl1	-0.01645	0.88001	1
Ibtk	-0.01645	0.477376	1
1600010M07Rik	-0.01646	0.855968	1
Psmf1	-0.01646	0.263823	1
Pola2	-0.01646	0.690883	1
G3bp2	-0.01646	0.641027	1
Zfp408	-0.01646	0.04946	1
Cul9	-0.01647	0.904904	1
Tardbp	-0.01648	0.604858	1
Rbm48	-0.01649	0.488451	1
St7	-0.01649	0.607215	1
Ube2q2	-0.01651	0.485049	1
Pus10	-0.01653	0.289337	1
Zfp740	-0.01653	0.692393	1
Usp37	-0.01654	0.60569	1
Pak1ip1	-0.01654	0.517295	1
Gmfb	-0.01654	0.956437	1
Wsb2	-0.01655	0.683914	1
Ahsa1	-0.01657	0.098131	1
Rfx5	-0.01659	0.508833	1
Coq8b	-0.0166	0.788491	1
Oxr1	-0.01661	0.648238	1
Cox7a2	-0.01662	0.63411	1
Acadsb	-0.01662	0.804798	1
Pabpc4	-0.01663	0.734891	1
Ccdc84	-0.01663	0.540638	1
Abhd16a	-0.01666	0.890528	1
Prps2	-0.01667	0.77467	1
Il2rg	-0.01668	0.724554	1
Mgll	-0.0167	0.649793	1
Fdx1	-0.0167	0.941284	1
Mib2	-0.01672	0.35194	1
Mrpl22	-0.01673	0.222745	1
Serinc3	-0.01673	0.132479	1
Srl	-0.01674	0.038729	1
Atl3	-0.01674	0.638696	1
Sgsm2	-0.01675	0.246783	1
Ndufaf3	-0.01676	0.614603	1
Jkamp	-0.01676	0.476344	1
Hmgn1	-0.01677	0.892709	1
Oma1	-0.01677	0.85194	1
Nox1	-0.01682	0.727246	1
Sugt1	-0.01683	0.909301	1
Elk3	-0.01684	0.664521	1

Gtf3c3	-0.01684	0.812892	1
Gm4356	-0.01687	0.026567	1
Polr3b	-0.0169	0.875203	1
Hps4	-0.0169	0.993251	1
Lasp1	-0.01691	0.898736	1
Polr3k	-0.01692	0.045972	1
R74862	-0.01693	0.292466	1
Sat1	-0.01693	0.197222	1
Rpa3	-0.01694	0.257354	1
Dnajc15	-0.01694	0.9236	1
Ddx5	-0.01695	0.319304	1
Eif1ad	-0.01695	0.374182	1
Anapc11	-0.01695	0.844345	1
Gm6712	-0.01696	0.147912	1
Slc25a16	-0.01696	0.429781	1
Myd88	-0.01697	0.247805	1
Prelid3b	-0.01699	0.970132	1
Cpt1a	-0.01702	0.56166	1
Stam	-0.01702	0.927524	1
Ndufv1	-0.01703	0.602982	1
Zfp472	-0.01703	0.274844	1
Ap5z1	-0.01703	0.019461	1
Uck1	-0.01704	0.286728	1
Rdx	-0.01704	0.616143	1
E230016M11Rik	-0.01704	0.744356	1
Bcorl1	-0.01705	0.585881	1
Fam76a	-0.01705	0.854298	1
Zfp687	-0.01708	0.444579	1
Zfp180	-0.01708	0.226809	1
Traf7	-0.01712	0.184031	1
Cep250	-0.01713	0.13177	1
Txn2	-0.01714	0.317998	1
Rpf1	-0.01715	0.811525	1
Cenpt	-0.01715	0.077109	1
Ring1	-0.01716	0.887933	1
Cox18	-0.01716	0.457548	1
Atp9b	-0.01717	0.365994	1
Gm42917	-0.01718	0.099802	1
Arfgap1	-0.01719	0.552996	1
Leng1	-0.01719	0.820925	1
Wdfy3	-0.0172	0.55418	1
Hcfc2	-0.01721	0.69185	1
Papss1	-0.01721	0.266717	1
Arl13b	-0.01722	0.056124	1
Tmem131	-0.01725	0.143402	1
Wdr25	-0.01725	0.667363	1
Ctr9	-0.01727	0.658708	1
Cars	-0.01727	0.340879	1

Efcab2	-0.01728	0.595496	1
Tmem265	-0.01729	0.188619	1
Ypel3	-0.01731	0.362093	1
Pitpnb	-0.01731	0.76398	1
Farsa	-0.01732	0.484615	1
Tfip11	-0.01734	0.509631	1
Mvb12a	-0.01735	0.423847	1
Trnau1ap	-0.01735	0.562018	1
Tsnax	-0.01735	0.874414	1
Luzp1	-0.01737	0.779598	1
Alkbh3	-0.01737	0.826417	1
Dram2	-0.01737	0.731475	1
Ppp1r3b	-0.01738	0.705701	1
Iqgap1	-0.01742	0.703588	1
Adgrl2	-0.01745	0.976202	1
Zfp330	-0.01747	0.851614	1
Ric8a	-0.01747	0.149886	1
Cep95	-0.0175	0.328989	1
Hivep1	-0.0175	0.204893	1
Adnp	-0.01752	0.934386	1
Vps4a	-0.01752	0.25328	1
Tagap	-0.01753	0.099712	1
Ppil4	-0.01756	0.280775	1
Dhodh	-0.01756	0.218968	1
Rab12	-0.01758	0.524172	1
Rab28	-0.01758	0.854602	1
0610010F05Rik	-0.0176	0.28997	1
Huwe1	-0.01761	0.706635	1
Acsf2	-0.01762	0.277861	1
Ip6k2	-0.01763	0.474424	1
Tcerg1	-0.01766	0.626352	1
Ccdc91	-0.01767	0.121865	1
Tbc1d16	-0.01768	0.376668	1
Acad10	-0.01768	0.38628	1
Gpd2	-0.01769	0.398188	1
Ubtd2	-0.01769	0.325207	1
Git1	-0.01769	0.146149	1
Mbtps2	-0.0177	0.242744	1
Ccdc90b	-0.0177	0.478232	1
Fam214b	-0.0177	0.075817	1
Inpp5a	-0.01771	0.579527	1
1110038B12Rik	-0.01771	0.703573	1
Zfp263	-0.01772	0.786451	1
Zfp410	-0.01772	0.644986	1
Med19	-0.01774	0.878434	1
Nedd1	-0.01777	0.597931	1
Usf3	-0.01778	0.833165	1
4833438C02Rik	-0.01778	0.27678	1

D030028A08Rik	-0.0178	0.129982	1
Phip	-0.01782	0.694276	1
Fam151b	-0.01783	0.048897	1
Mphosph10	-0.01784	0.754989	1
Ptdss2	-0.01784	0.42484	1
Alg11	-0.01786	0.337669	1
Zfp60	-0.01787	0.136206	1
Impa1	-0.01787	0.275995	1
Cyb5b	-0.01789	0.476761	1
Ppt2	-0.0179	0.652229	1
H2-DMb2	-0.01791	0.500019	1
Mitd1	-0.01792	0.199808	1
D2hgdh	-0.01793	0.180754	1
Frs2	-0.01794	0.696918	1
Pgpep1	-0.01797	0.392514	1
Mrps31	-0.01798	0.454895	1
Prr3	-0.01799	0.257968	1
Arcn1	-0.01799	0.720667	1
Tmem86b	-0.01801	0.10086	1
C1galt1c1	-0.01802	0.957644	1
Rp9	-0.01802	0.75432	1
Wtap	-0.01804	0.961783	1
Fam98a	-0.01804	0.272959	1
Supt7l	-0.01804	0.039272	1
Pop1	-0.01806	0.535318	1
Pcyox1	-0.01806	0.607912	1
Minpp1	-0.01809	0.927487	1
Mrpl10	-0.01812	0.283725	1
Rnf146	-0.01812	0.431921	1
Nr3c2	-0.01812	0.669287	1
Sfxn1	-0.01817	0.873892	1
Zfp654	-0.01818	0.439774	1
Tasp1	-0.01818	0.103774	1
Unc93b1	-0.01821	0.386571	1
Fmo5	-0.01823	0.357125	1
Pkn1	-0.01824	0.598329	1
BC005537	-0.01825	0.935372	1
Gtf2e2	-0.01825	0.480142	1
Zdhhc1	-0.01826	0.094418	1
Ccnt1	-0.01828	0.366337	1
6030458C11Rik	-0.01829	0.203711	1
Gm4117	-0.01829	0.478378	1
Prkaca	-0.01831	0.521118	1
Adam9	-0.01831	0.629486	1
Ptar1	-0.01831	0.383353	1
Abhd5	-0.01832	0.094082	1
Ap1s1	-0.01832	0.735326	1
Ipo4	-0.01833	0.35756	1

Nt5c	-0.01834	0.544014	1
Arhgap5	-0.01834	0.853117	1
Kat2a	-0.01834	0.071672	1
Zer1	-0.01834	0.71854	1
Slc30a1	-0.01835	0.236612	1
Gm26749	-0.01838	0.436046	1
Edc4	-0.01838	0.131856	1
Ppfibp1	-0.01842	0.207909	1
Cdc42	-0.01844	0.692412	1
Otud5	-0.01845	0.765641	1
Creb5	-0.01845	0.739327	1
Itpripl1	-0.01846	0.766888	1
Nf2	-0.01846	0.101681	1
Josd2	-0.01847	0.415863	1
Prelid1	-0.01848	0.545553	1
Prox1	-0.01849	0.225271	1
Ppm1g	-0.01849	0.282944	1
4833420G17Rik	-0.01854	0.88025	1
Rbm5	-0.01856	0.677383	1
Gm15952	-0.01858	0.339578	1
Mapk7	-0.01858	0.08518	1
Hebp1	-0.0186	0.575706	1
Map3k2	-0.01862	0.61496	1
Ccl2	-0.01862	0.194686	1
Relt	-0.01863	0.163823	1
Txlna	-0.01863	0.449033	1
Msh2	-0.01864	0.134348	1
Sdhaf3	-0.01865	0.188653	1
Gm44899	-0.01867	0.287266	1
Ddx19b	-0.01868	0.438278	1
Zfp626	-0.01869	0.802977	1
Man2a2	-0.01869	0.919752	1
Rnf103	-0.0187	0.990203	1
Opa1	-0.0187	0.537477	1
Nupr1	-0.0187	0.571304	1
Bend6	-0.01872	0.970593	1
Cars2	-0.01872	0.775058	1
Med27	-0.01872	0.153443	1
Card9	-0.01873	0.682383	1
Usp32	-0.01873	0.715372	1
Ercc6	-0.01873	0.385238	1
Surf6	-0.01874	0.166452	1
Rprd1b	-0.01875	0.416425	1
4921524J17Rik	-0.01876	0.243943	1
Gys1	-0.01877	0.143624	1
Zfp672	-0.01879	0.588038	1
E130102H24Rik	-0.01879	0.523227	1
Ints14	-0.01881	0.693224	1

Nln	-0.01882	0.993224	1
Ddx39	-0.01883	0.490186	1
Scpep1	-0.01884	0.403864	1
Ccdc32	-0.01887	0.986248	1
Catspere2	-0.01888	0.815488	1
Gm20559	-0.01889	0.14019	1
Slc35d1	-0.01891	0.040836	1
Vps50	-0.01892	0.438482	1
Pum1	-0.01892	0.932322	1
Tbc1d10b	-0.01894	0.599328	1
Bcl3	-0.01894	0.081587	1
E4f1	-0.01896	0.98717	1
Wdr3	-0.01896	0.105666	1
Agap1	-0.01896	0.709662	1
Senp3	-0.01898	0.108116	1
Ube2i	-0.01898	0.306939	1
Rnf19a	-0.019	0.780065	1
Slc25a40	-0.019	0.862787	1
Gab1	-0.01903	0.70306	1
Galnt7	-0.01905	0.914121	1
Taf11	-0.01906	0.128552	1
Ap1b1	-0.01906	0.58313	1
Bscl2	-0.01907	0.916919	1
Evi2	-0.0191	0.030834	1
Snhg9	-0.0191	0.681603	1
Slc25a43	-0.01911	0.074139	1
Vps13b	-0.01911	0.450826	1
Slc25a46	-0.01912	0.322678	1
Alg13	-0.01912	0.085421	1
Mettl25	-0.01912	0.518118	1
Pphln1	-0.01912	0.519074	1
Hmgxb3	-0.01913	0.731925	1
Bcl10	-0.01915	0.198708	1
Snapin	-0.01916	0.936628	1
Ocrl	-0.01917	0.403093	1
Fam160b1	-0.01917	0.40744	1
Zbtb4	-0.01917	0.532816	1
Slc25a53	-0.01919	0.772326	1
Slc25a13	-0.01921	0.11479	1
Cbfa2t2	-0.01921	0.699621	1
Calcrl	-0.01923	0.184963	1
Aasdh	-0.01924	0.069608	1
Mcm7	-0.01924	0.384344	1
Usp10	-0.01925	0.288843	1
Afg3l2	-0.01926	0.475124	1
Eef1akmt4	-0.01927	0.094835	1
Aarsd1	-0.01928	0.800449	1
Cr1l	-0.01928	0.586355	1

Lsm12	-0.01928	0.650768	1
Hsd17b4	-0.01929	0.980188	1
Cope	-0.01931	0.747504	1
Dpy19l1	-0.01931	0.306496	1
Mlkl	-0.01932	0.192321	1
Samd8	-0.01933	0.688299	1
Rc3h2	-0.01933	0.090545	1
Smarcd2	-0.01933	0.149282	1
Ap3d1	-0.01934	0.818805	1
Map4k2	-0.01935	0.658269	1
Ftx	-0.01935	0.473577	1
Cluh	-0.01938	0.7488	1
Col15a1	-0.01939	0.100419	1
Smndc1	-0.01939	0.685365	1
Pprc1	-0.01939	0.789784	1
Tmem87a	-0.0194	0.303898	1
Taf12	-0.01941	0.271639	1
Gm3448	-0.01941	0.027263	1
Galc	-0.01943	0.729439	1
Ice2	-0.01944	0.597108	1
Nt5c2	-0.01944	0.856884	1
Atp6v1g2	-0.01945	0.056774	1
Rtcb	-0.01948	0.548198	1
Mief1	-0.01949	0.444646	1
Psmd10	-0.0195	0.86545	1
Sun1	-0.0195	0.33731	1
Zfp729a	-0.01955	0.146823	1
Cnot6l	-0.01956	0.537821	1
Vps37b	-0.01956	0.429555	1
Zfp182	-0.01957	0.601059	1
Hdhd5	-0.01957	0.166473	1
C1galt1	-0.01957	0.771374	1
Gm37768	-0.01958	0.345468	1
Golga7	-0.01959	0.758462	1
Gm41764	-0.01961	0.560191	1
Sept2	-0.01963	0.501017	1
Ganab	-0.01964	0.348098	1
Gm21860	-0.01964	0.346568	1
Ing4	-0.01964	0.4856	1
Wasl	-0.01966	0.7271	1
Plcl1	-0.01968	0.358744	1
Ppp4r2	-0.01969	0.406349	1
Cxxc1	-0.01969	0.341764	1
Zbtb44	-0.0197	0.202478	1
Zbtb41	-0.0197	0.815512	1
Paxip1	-0.01971	0.593522	1
Nlrx1	-0.01971	0.041726	1
Zfp74	-0.01973	0.104416	1

Acaa1a	-0.01975	0.632722	1
Fam172a	-0.01975	0.368019	1
Dlg3	-0.01975	0.248444	1
Rad9b	-0.01975	0.012316	1
St14	-0.01976	0.829655	1
Tigd2	-0.01979	0.633874	1
6330418K02Rik	-0.01981	0.27768	1
Cnep1r1	-0.01981	0.56544	1
Cd101	-0.01982	0.365953	1
Pkd1	-0.01983	0.980153	1
Adsl	-0.01983	0.542054	1
Shfl	-0.01986	0.406563	1
Ipo11	-0.01987	0.804581	1
Kmt5a	-0.01988	0.629888	1
Tmem120a	-0.0199	0.368616	1
Nol9	-0.01991	0.317328	1
Tfg	-0.01992	0.502863	1
Aldh16a1	-0.01994	0.197834	1
Tspan5	-0.01996	0.482744	1
Trappc6b	-0.01997	0.065993	1
Nipal3	-0.01997	0.00278	1
Nsun4	-0.01997	0.173487	1
Sh2b3	-0.01998	0.516556	1
Sirt7	-0.01999	0.876832	1
Pdcd7	-0.01999	0.296393	1
Cyhr1	-0.02	0.18834	1
Krr1	-0.02	0.509488	1
Hdac11	-0.02001	0.573563	1
Spg7	-0.02001	0.909021	1
Nmt1	-0.02001	0.236274	1
Xndc1	-0.02005	0.985754	1
Ubl4a	-0.02005	0.539767	1
Snapc4	-0.02006	0.108499	1
Phrf1	-0.02007	0.04496	1
Rad52	-0.02007	0.132994	1
Tmem216	-0.02007	0.575281	1
Pwp1	-0.02008	0.304956	1
Snx20	-0.02008	0.271989	1
Mfsd13a	-0.02008	0.161969	1
Psen1	-0.02009	0.803124	1
4930469K13Rik	-0.02009	0.480504	1
Bnip3l	-0.0201	0.758386	1
Glud1	-0.0201	0.976333	1
Fars2	-0.02011	0.503382	1
Usp48	-0.02011	0.377281	1
Paip2b	-0.02011	0.330924	1
Pfkfb2	-0.02012	0.396198	1
Glcci1	-0.02012	0.613762	1

Snap47	-0.02013	0.045986	1
Aebp2	-0.02014	0.272883	1
Ddx46	-0.02015	0.678858	1
Cep104	-0.02016	0.116018	1
Slc25a26	-0.02016	0.922282	1
Zc3h3	-0.02016	0.133505	1
Fastkd1	-0.02017	0.016964	1
Cdk2ap1	-0.02017	0.714926	1
Mta1	-0.02018	0.268631	1
Rpp25l	-0.02018	0.541394	1
Strn4	-0.02018	0.808731	1
Rbms2	-0.02019	0.109217	1
Rpa1	-0.02019	0.503755	1
Mob3c	-0.02019	0.909665	1
Igip	-0.0202	0.159532	1
Usp31	-0.02021	0.09609	1
Gnptab	-0.02022	0.286287	1
Ipo7	-0.02022	0.553152	1
Nos1ap	-0.02024	0.673551	1
Trim47	-0.02026	0.356342	1
Rpp14	-0.02026	0.117422	1
Chmp3	-0.02027	0.284951	1
Gas7	-0.02027	0.958472	1
Pgm1	-0.02027	0.340481	1
Stambp	-0.02028	0.77202	1
Ctdp1	-0.02028	0.481699	1
Abce1	-0.02029	0.99089	1
Mrps18b	-0.02031	0.489196	1
Sharpin	-0.02031	0.709851	1
Adap1	-0.02031	0.738698	1
Ccnh	-0.02032	0.310142	1
Sod1	-0.02032	0.965617	1
Gdi1	-0.02034	0.425895	1
Slc35e2	-0.02034	0.722356	1
Wwp1	-0.02036	0.754973	1
Dph5	-0.02037	0.873508	1
Usp45	-0.02038	0.547571	1
Ddx42	-0.02038	0.64459	1
Lsg1	-0.02038	0.782631	1
Gpatch2	-0.02038	0.319832	1
Mfn1	-0.02039	0.53022	1
2900097C17Rik	-0.02039	0.893594	1
Tulp4	-0.02041	0.257418	1
Zbtb37	-0.02045	0.263649	1
Cab39l	-0.02045	0.254823	1
Brms1l	-0.02047	0.831684	1
Camkk2	-0.02048	0.416833	1
Cyb561d1	-0.02048	0.024303	1

Ifi27	-0.02052	0.592402	1
Pag1	-0.02057	0.494214	1
Crebl2	-0.02059	0.257117	1
Inpp5d	-0.02059	0.100647	1
Psip1	-0.02059	0.442321	1
Imp4	-0.0206	0.939305	1
Dnajc14	-0.0206	0.250056	1
Kdm4b	-0.02061	0.503752	1
Pald1	-0.02061	0.268051	1
Prrc2c	-0.02061	0.439549	1
Bahd1	-0.02061	0.104863	1
Fam234a	-0.02061	0.871121	1
Dock9	-0.02062	0.387615	1
Tent4a	-0.02062	0.167723	1
Rhbdd1	-0.02065	0.847738	1
Purg	-0.02067	0.533232	1
Nek4	-0.02068	0.414033	1
Rpap2	-0.02068	0.411779	1
Tmem186	-0.02071	0.211649	1
Atad2b	-0.02071	0.168913	1
Tmem68	-0.02071	0.542531	1
Clcn5	-0.02073	0.824989	1
Stx6	-0.02075	0.77027	1
Lyrm1	-0.02075	0.633421	1
Tbcel	-0.02076	0.136089	1
Rpe	-0.02077	0.949438	1
Tgs1	-0.02078	0.768501	1
Mrps25	-0.02079	0.36474	1
Polr3h	-0.0208	0.038823	1
Taf1a	-0.02081	0.006006	1
Fastk	-0.02084	0.125251	1
1700030K09Rik	-0.02085	0.080749	1
Pnpla6	-0.02086	0.12581	1
Rrp15	-0.02086	0.630692	1
Ms4a6c	-0.02088	0.421828	1
Pld2	-0.0209	0.200832	1
Mrpl13	-0.0209	0.611418	1
Gpn2	-0.02095	0.223767	1
Atp13a2	-0.02096	0.598358	1
Dbnl	-0.02097	0.142771	1
Pcid2	-0.02098	0.138053	1
Neu1	-0.02099	0.300892	1
Dhx33	-0.02101	0.192215	1
Ap4b1	-0.02102	0.042333	1
BC051142	-0.02102	0.093028	1
Zbtb46	-0.02103	0.398032	1
Ildr2	-0.02103	0.490606	1
Baz1b	-0.02103	0.894151	1

Pef1	-0.02107	0.273076	1
Ecpas	-0.02109	0.554727	1
Nsmce4a	-0.0211	0.866987	1
Zmym4	-0.02111	0.671993	1
Oip5os1	-0.02113	0.922453	1
Immt	-0.02114	0.445178	1
Lcp1	-0.02116	0.501703	1
Gnpat	-0.02117	0.955782	1
Mpi	-0.02117	0.69296	1
Spsb2	-0.02118	0.086366	1
Slc9a1	-0.02118	0.437006	1
Helz2	-0.02119	0.037592	1
Nbn	-0.0212	0.265121	1
Fbxo36	-0.0212	0.109859	1
Snx9	-0.02122	0.367878	1
Snap29	-0.02123	0.124257	1
Dnal1	-0.02124	0.087079	1
Lysmd3	-0.02124	0.063484	1
Mosmo	-0.02124	0.213695	1
Tk1	-0.02125	0.27127	1
Tubb5	-0.02125	0.542344	1
Mxd4	-0.02125	0.860999	1
Ehd1	-0.02129	0.808747	1
Sik3	-0.0213	0.953543	1
Ebf3	-0.02132	0.400167	1
Slf1	-0.02133	0.20432	1
A530088E08Rik	-0.02133	0.042411	1
Tlnrd1	-0.02133	0.014702	1
Znrf3	-0.02133	0.55478	1
Usp38	-0.02136	0.412691	1
Trappc13	-0.02137	0.385439	1
Alg8	-0.02138	0.475626	1
Asph	-0.02138	0.825445	1
Sgms1	-0.02139	0.431237	1
Isg20l2	-0.0214	0.234644	1
Xpa	-0.0214	0.228273	1
Spindoc	-0.02142	0.1588	1
Pdik1l	-0.02142	0.335526	1
H2afy	-0.02144	0.812262	1
Wdr73	-0.02145	0.608245	1
Ifit3	-0.02146	0.000767	1
Man1b1	-0.02146	0.567871	1
Gm47601	-0.02147	0.648213	1
Cyb561a3	-0.02151	0.916528	1
Mrps11	-0.02152	0.426459	1
Oxsm	-0.02153	0.087339	1
Atat1	-0.02158	0.017425	1
Slc20a2	-0.02158	0.680248	1

AI413582	-0.02158	0.392567	1
Etfa	-0.0216	0.439935	1
Flywch1	-0.02161	0.04084	1
Ern1	-0.02162	0.188266	1
Zfp655	-0.02163	0.975841	1
Scrib	-0.02163	0.065584	1
Trub2	-0.02164	0.233785	1
Pnpt1	-0.02164	0.089244	1
Rassf2	-0.02166	0.582855	1
Gm47167	-0.02167	0.636041	1
Dnajb12	-0.02168	0.172389	1
Kdm5b	-0.0217	0.132025	1
Slc9a3r1	-0.02171	0.257307	1
2410004B18Rik	-0.02171	0.274044	1
Nufip1	-0.02172	0.158584	1
Lhpp	-0.02172	0.109921	1
Zc3h8	-0.02174	0.267911	1
Vamp1	-0.02174	0.191506	1
Sbf1	-0.02175	0.434282	1
Gatb	-0.02175	0.518621	1
Atp1a1	-0.02176	0.749455	1
Creb3	-0.02177	0.590184	1
Ccr1	-0.02177	0.91292	1
Rbm28	-0.02178	0.684556	1
Nudt7	-0.02179	0.341095	1
Erp44	-0.02181	0.33654	1
Nmi	-0.02182	0.474402	1
Rgmb	-0.02183	0.371943	1
Map1s	-0.02183	0.800345	1
Tspan32	-0.02185	0.58619	1
Pde7a	-0.02187	0.323921	1
Shld2	-0.02187	0.362066	1
Ptpn12	-0.02188	0.248462	1
Helq	-0.02189	0.082398	1
Nol10	-0.0219	0.130171	1
Zbtb25	-0.02191	0.051075	1
Ndufs1	-0.02191	0.034655	1
Amotl1	-0.02193	0.63731	1
Nudt13	-0.02193	0.059683	1
Dgkq	-0.02193	0.107834	1
Wdr91	-0.02195	0.140075	1
Uhrf1bp1l	-0.02195	0.464375	1
Trim2	-0.02196	0.14338	1
Bad	-0.02196	0.280609	1
Scarf2	-0.02197	0.233176	1
Fbxw11	-0.02198	0.395059	1
Dazap1	-0.022	0.753546	1
Snrnp70	-0.02201	0.316185	1

Slamf1	-0.02202	0.055309	1
Nab1	-0.02203	0.312618	1
Rabif	-0.02204	0.04453	1
Rbm39	-0.02205	0.352558	1
Abcf3	-0.02208	0.544959	1
Morc2a	-0.02209	0.187482	1
Deptor	-0.0221	0.194734	1
Prr13	-0.02212	0.350854	1
Rrp7a	-0.02213	0.101881	1
Aatk	-0.02214	0.017782	1
Lemd3	-0.02215	0.541704	1
Gm43378	-0.02217	0.012761	1
Eif4ebp2	-0.02217	0.039959	1
Zfp276	-0.0222	0.391336	1
Cenpp	-0.02221	0.79852	1
Gm16118	-0.02223	0.124961	1
Tes	-0.02224	0.588789	1
Lilrb4a	-0.02224	0.532261	1
Hgs	-0.02225	0.316391	1
Kif1c	-0.02225	0.023462	1
Camsap2	-0.02225	0.761506	1
Hs1bp3	-0.02227	0.221499	1
Cab39	-0.02227	0.969153	1
Helz	-0.02228	0.034309	1
Fam13b	-0.02228	0.311155	1
Fech	-0.02231	0.641972	1
Unk	-0.02233	0.522013	1
Micos10	-0.02234	0.361237	1
Aig1	-0.02235	0.588306	1
Polr3f	-0.02235	0.278124	1
Unc119b	-0.02237	0.189877	1
8030462N17Rik	-0.02239	0.449994	1
Stx17	-0.0224	0.688711	1
Hlcs	-0.0224	0.493253	1
Arfrp1	-0.02246	0.667113	1
Zfp664	-0.02247	0.035593	1
Cwf19l2	-0.02247	0.990759	1
Pxmp4	-0.02247	0.060603	1
Ehbp1l1	-0.02249	0.067416	1
Pros1	-0.0225	0.374416	1
Fnip1	-0.0225	0.194986	1
Spg11	-0.02253	0.207817	1
Fam162a	-0.02253	0.271518	1
Braf	-0.02255	0.786403	1
Emc2	-0.02255	0.429064	1
Pogk	-0.02255	0.321435	1
Stag1	-0.02256	0.28666	1
Farp2	-0.02257	0.135713	1

Aimp1	-0.02258	0.820857	1
Clk1	-0.02258	0.618755	1
Appbp2	-0.0226	0.602434	1
Ezh1	-0.02261	0.370569	1
Ppp1r12c	-0.02263	0.269553	1
Selenoo	-0.02263	0.297932	1
Rubcn	-0.02264	0.265839	1
Copg1	-0.02265	0.165038	1
Ctps2	-0.02266	0.155156	1
Gm16552	-0.02267	0.292243	1
Fktn	-0.02268	0.109973	1
Dnhd1	-0.0227	0.029614	1
Ethe1	-0.0227	0.904338	1
Mrpl44	-0.02271	0.066505	1
Hdac2	-0.02271	0.697462	1
Tmem229b	-0.02273	0.225686	1
Sac3d1	-0.02274	0.214619	1
Acadm	-0.02274	0.27846	1
Nipsnap2	-0.02275	0.377647	1
Wipi2	-0.02277	0.13787	1
Gm5431	-0.02278	0.075088	1
Stip1	-0.0228	0.310935	1
Zc3h14	-0.02281	0.332828	1
Ap3s2	-0.02282	0.407916	1
Rsf1os1	-0.02283	0.363856	1
Smu1	-0.02283	0.589214	1
Gm36279	-0.02283	0.023622	1
Macf1	-0.02283	0.078859	1
Rmdn2	-0.02286	0.005229	1
Kcnq1ot1	-0.02287	0.629487	1
Zfp346	-0.02288	0.754748	1
Cnnm4	-0.02288	0.084972	1
Tlr13	-0.02289	0.956594	1
Dnajc19	-0.02289	0.795697	1
Dnttip2	-0.02291	0.109343	1
Tfcp2	-0.02291	0.135689	1
Xkr6	-0.02292	0.506859	1
Rptor	-0.02293	0.418101	1
Fes	-0.02293	0.803893	1
Acad12	-0.02298	0.334342	1
Xpc	-0.02298	0.038813	1
Rbck1	-0.02299	0.060682	1
Hbp1	-0.023	0.494718	1
Clic4	-0.023	0.104465	1
Atp6v1h	-0.02301	0.736518	1
Ptbp3	-0.02301	0.524949	1
Dusp12	-0.02305	0.518741	1
Gm32849	-0.02305	0.153376	1

Polb	-0.02305	0.118888	1
Zfp933	-0.02305	0.411011	1
Ube2w	-0.02305	0.50387	1
Rtf2	-0.02306	0.40355	1
Stk11ip	-0.02306	0.15695	1
Sdhd	-0.02312	0.945625	1
Parn	-0.02312	0.901629	1
Lhfpl2	-0.02313	0.512458	1
Mlycd	-0.02315	0.424818	1
6430548M08Rik	-0.02317	0.10241	1
Adprm	-0.02318	0.069206	1
Gcnt1	-0.0232	0.516458	1
Pcnx	-0.0232	0.151118	1
Ptgfrn	-0.02321	0.114839	1
Rnf149	-0.02325	0.682955	1
Abcf1	-0.02326	0.799927	1
Rfesd	-0.02327	0.172911	1
Zcwpw2	-0.02327	0.060159	1
Stxbp5	-0.02328	0.415046	1
Pgs1	-0.02331	0.683614	1
Gtf2f1	-0.02332	0.816897	1
Amd1	-0.02334	0.177577	1
Dmxl2	-0.02337	0.189937	1
Zfp397	-0.02338	0.157497	1
Rnf40	-0.0234	0.080451	1
Dcaf13	-0.0234	0.620917	1
Taf4b	-0.02342	0.700949	1
Xxylt1	-0.02345	0.48728	1
Ncaph2	-0.02345	0.168815	1
Paip2	-0.02348	0.635671	1
Acbd5	-0.02351	0.637726	1
Fbf1	-0.02351	0.003666	1
Rsrc1	-0.02351	0.428647	1
Cdkal1	-0.02351	0.245369	1
Ttc8	-0.02352	0.689441	1
Tomm40	-0.02352	0.062708	1
Cd84	-0.02355	0.681446	1
Tpm3	-0.02356	0.351937	1
Gm49359	-0.02359	0.863269	1
Tsen2	-0.02361	0.113454	1
Pcgf3	-0.02361	0.125762	1
Gan	-0.02361	0.062765	1
Slc10a7	-0.02365	0.719319	1
Casd1	-0.02365	0.238835	1
Ammecr1	-0.02366	0.137379	1
Ints7	-0.02369	0.788698	1
Gm27003	-0.0237	0.061068	1
Erich1	-0.0237	0.130478	1

Abraxas2	-0.02371	0.685912	1
Chuk	-0.02371	0.07505	1
Sh3kbp1	-0.02373	0.395914	1
Tmem126b	-0.02374	0.041055	1
Scfd1	-0.02375	0.987044	1
Fbxo30	-0.02378	0.825531	1
Wdr13	-0.02382	0.207685	1
AU041133	-0.02384	0.148786	1
Tlk1	-0.02385	0.33157	1
Myo18a	-0.02386	0.124789	1
Cep295	-0.02386	0.235126	1
Eif2b3	-0.02386	0.465907	1
Tle4	-0.02386	0.572706	1
Castor2	-0.0239	0.137178	1
Fam214a	-0.0239	0.347784	1
Kyat3	-0.0239	0.000277	1
Smap2	-0.0239	0.187853	1
Arhgef10l	-0.02392	0.037646	1
Haus2	-0.02395	0.187274	1
Zfp943	-0.02395	0.353581	1
Osbp	-0.02396	0.365491	1
Ranbp2	-0.02396	0.651397	1
Nop56	-0.02397	0.463178	1
Zmat3	-0.02397	0.134074	1
Cmc1	-0.024	0.431662	1
Eif2ak1	-0.024	0.666735	1
Gm13091	-0.02402	0.005647	1
Ciao2b	-0.02403	0.076508	1
Tal1	-0.02405	0.045971	1
Nkiras2	-0.02405	0.096751	1
Layn	-0.02407	0.463129	1
Zfp958	-0.02408	0.391219	1
Gm14798	-0.02409	0.006191	1
Slc33a1	-0.0241	0.749689	1
Bri3bp	-0.02412	0.065171	1
Vamp2	-0.02412	0.048915	1
Vamp4	-0.02412	0.161711	1
Armc7	-0.02412	0.334844	1
Hk1	-0.02413	0.005959	1
Ganc	-0.02413	0.571311	1
F3	-0.02414	0.02448	1
Sap30l	-0.02415	0.109304	1
Ube2g2	-0.02416	0.134045	1
Mcee	-0.02418	0.390208	1
Shq1	-0.02419	0.29991	1
Ssb	-0.02421	0.811059	1
Mpp5	-0.02422	0.51948	1
Myg1	-0.02422	0.550293	1

Gm15886	-0.02422	0.03071	1
Pitpnc1	-0.02423	0.388177	1
Slirp	-0.02428	0.412704	1
Mmaa	-0.02428	0.375138	1
Mapkbp1	-0.02428	0.161415	1
Smarce1	-0.02431	0.890998	1
Dpf2	-0.02432	0.33152	1
Foxp1	-0.02435	0.317726	1
Rac1	-0.02435	0.34906	1
Slc35a5	-0.02436	0.096927	1
Lrp1	-0.02438	0.538879	1
Cul5	-0.02439	0.981336	1
Zfp444	-0.02441	0.033895	1
Khnyn	-0.02442	0.225539	1
Tmlhe	-0.02443	0.692334	1
Ube2o	-0.02443	0.294704	1
Ubald2	-0.02445	0.570794	1
Pacsin1	-0.02448	0.080731	1
Tinf2	-0.02451	0.251711	1
Cd200r1	-0.02453	0.026418	1
Nudt19	-0.02453	0.145431	1
Ap4m1	-0.02454	0.499097	1
Dpp3	-0.02455	0.242216	1
Emc1	-0.02456	0.126295	1
Fmr1	-0.02456	0.996079	1
Galns	-0.02457	0.035536	1
Dab2	-0.02458	0.711261	1
2700062C07Rik	-0.02459	0.10855	1
Tpk1	-0.02459	0.977095	1
Nsfl1c	-0.0246	0.293775	1
Dzip3	-0.0246	0.049909	1
Preb	-0.02462	0.64505	1
Glod4	-0.02465	0.288083	1
Fry	-0.02466	0.743022	1
Ralbp1	-0.02466	0.17149	1
Gm20342	-0.02468	0.111502	1
Gm27201	-0.02468	0.178281	1
Nckipsd	-0.02469	0.17987	1
Asb7	-0.0247	0.245747	1
Brf1	-0.0247	0.337971	1
Gch1	-0.0247	0.198624	1
Plcb3	-0.0247	0.735089	1
Carf	-0.02471	0.074866	1
Lrmda	-0.02471	0.099114	1
Atp11b	-0.02472	0.318093	1
Tubgcp4	-0.02474	0.391723	1
Napg	-0.02474	0.243321	1
Kntc1	-0.02477	0.960628	1

Ttc27	-0.02477	0.032766	1
Zfp668	-0.02477	0.034265	1
1700109H08Rik	-0.02478	0.180511	1
Foxk2	-0.02478	0.600589	1
Mbd5	-0.02479	0.354317	1
Aen	-0.02481	0.091309	1
Phf11a	-0.02481	0.067996	1
Sympk	-0.02482	0.287518	1
Dgcr8	-0.02482	0.048783	1
Arl8a	-0.02482	0.637796	1
Stat5a	-0.02484	0.101352	1
Malt1	-0.02485	0.992848	1
Commd6	-0.02486	0.549444	1
8030453O22Rik	-0.02487	0.159598	1
Gcn1	-0.02488	0.292014	1
Nip7	-0.02489	0.098872	1
Sh2b2	-0.0249	0.427481	1
Map3k12	-0.02494	0.001358	1
Slco4a1	-0.02495	0.968277	1
Ppp1r13b	-0.02496	0.215393	1
Qtrt2	-0.02497	0.069889	1
Epg5	-0.02497	0.36966	1
4833411C07Rik	-0.02498	0.04346	1
Ddx56	-0.02499	0.116176	1
Samd4b	-0.02499	0.924045	1
Zfp329	-0.02502	0.960355	1
Zfp942	-0.02504	0.144753	1
Eif2b5	-0.02506	0.210859	1
Il20rb	-0.02507	0.017997	1
Mrpl57	-0.02512	0.171385	1
Zfp169	-0.02516	0.456011	1
Gorasp2	-0.02517	0.105714	1
Shoc2	-0.02518	0.47604	1
Ncoa5	-0.02519	0.014448	1
Rap1gap2	-0.0252	0.236916	1
Ncor1	-0.02521	0.071962	1
Rps6kb2	-0.02522	0.044818	1
Vezt	-0.02523	0.164339	1
Dctn6	-0.02525	0.202929	1
Fblim1	-0.02525	0.812075	1
Mllt11	-0.02528	0.646852	1
Lrsam1	-0.0253	0.139743	1
Cdc123	-0.02533	0.307273	1
Specc1	-0.02534	0.370841	1
Tle1	-0.02534	0.004324	1
Pex6	-0.02535	0.229745	1
Pygo2	-0.02537	0.054069	1
Dyrk4	-0.02538	0.003054	1

Bet1	-0.02538	0.343963	1
Zfp771	-0.02538	0.115036	1
Armt1	-0.0254	0.05856	1
Pik3ca	-0.02542	0.576893	1
Mtx2	-0.02542	0.16488	1
Senp5	-0.02544	0.149568	1
Eif4e3	-0.02544	0.829253	1
Alms1	-0.02546	0.578746	1
Zfp800	-0.02546	0.416772	1
Sec22b	-0.02548	0.097922	1
Tnfsf13	-0.0255	0.052612	1
Hrh2	-0.02554	0.067547	1
Vps26a	-0.02555	0.396757	1
Slc25a51	-0.02556	0.129842	1
Arid3a	-0.02557	0.735893	1
Itgax	-0.02558	0.466473	1
Tbc1d19	-0.02561	0.458625	1
Mocs1	-0.02561	0.017702	1
Atp13a1	-0.02564	0.188821	1
Champ1	-0.02565	0.167729	1
Prkce	-0.02566	0.444169	1
Csf2ra	-0.02566	0.273048	1
Fntb	-0.02568	0.019155	1
Kmt2b	-0.02568	0.215477	1
Zfp715	-0.02568	0.724431	1
Lmf2	-0.02569	0.014988	1
Cwc22	-0.02571	0.59802	1
Rtn4ip1	-0.0258	0.291498	1
Fubp1	-0.0258	0.452952	1
Megf11	-0.02582	0.090968	1
Setd7	-0.02582	0.697187	1
Zswim6	-0.02583	0.530953	1
Tlk2	-0.02585	0.271054	1
Sf3b3	-0.02585	0.643442	1
Tollip	-0.02586	0.59783	1
Rbm43	-0.02587	0.074208	1
Akt2	-0.02589	0.083422	1
Ogdh	-0.02589	0.530003	1
Ermp1	-0.0259	0.136928	1
Mtbp	-0.02591	0.062558	1
Lsm14a	-0.02592	0.383594	1
Mindy3	-0.02597	0.201411	1
Rcor3	-0.02598	0.001909	1
Vps33b	-0.02599	0.019698	1
Katnbl1	-0.02601	0.321706	1
Zfp780b	-0.02601	0.286037	1
Trim11	-0.02604	0.737279	1
Akap7	-0.02604	0.603569	1

Gm13684	-0.02605	0.088384	1
Rnf144b	-0.02606	0.91151	1
Arrb1	-0.02609	0.434056	1
Parg	-0.02609	0.164605	1
Mgat1	-0.02614	0.506008	1
Csad	-0.02614	0.461458	1
Eif4e	-0.02614	0.302611	1
Wwp2	-0.02616	0.270958	1
Pex11b	-0.02616	0.330062	1
Zfp395	-0.02619	0.021562	1
Nop9	-0.0262	0.20147	1
Polk	-0.02621	0.804145	1
Zfhx2	-0.02622	0.013959	1
Map2k4	-0.02624	0.274361	1
Ppp4r3b	-0.02624	0.143345	1
Cemip2	-0.02627	0.021733	1
Bola3	-0.02627	0.125227	1
Kdm2b	-0.02627	0.902432	1
2900076A07Rik	-0.0263	0.133485	1
Fam89a	-0.02632	0.114051	1
Spock1	-0.02632	0.123184	1
Gm17018	-0.02636	0.029491	1
Akt1	-0.02636	0.156492	1
Atrn	-0.02638	0.153469	1
Fam185a	-0.02642	0.081771	1
Fam126a	-0.02642	0.329173	1
Ube2b	-0.02642	0.609075	1
Cnot10	-0.02643	0.081808	1
3300002I08Rik	-0.02643	0.153094	1
Psma5	-0.02644	0.2977	1
Zbtb18	-0.02644	0.658366	1
Bcl6	-0.02645	0.375747	1
Yipf1	-0.02646	0.320002	1
Lztr1	-0.02647	0.044343	1
Cdkn2aipnl	-0.02648	0.132209	1
Zfp113	-0.02648	0.097494	1
Tsg101	-0.02649	0.448646	1
Siglec1	-0.02649	0.376329	1
Ruvbl2	-0.0265	0.46309	1
Tsacc	-0.02653	0.020421	1
Zfp563	-0.02653	0.056848	1
Dnajc13	-0.02653	0.272915	1
Pelp1	-0.02654	0.350171	1
AI987944	-0.02655	0.079512	1
Kif13a	-0.02655	0.168397	1
Mthfd2l	-0.02656	0.076279	1
Nfkbil1	-0.02657	0.334159	1
Ppp1r9b	-0.02658	0.363309	1

Dop1a	-0.0266	0.269803	1
Hdgfl2	-0.02664	0.064839	1
Kdm3b	-0.02665	0.066279	1
Baz1a	-0.02668	0.324527	1
Uba1	-0.0267	0.144157	1
Uros	-0.0267	0.040071	1
Rbm4b	-0.0267	0.791775	1
Mbnl2	-0.02671	0.186193	1
Zkscan17	-0.02673	0.568567	1
Tmem127	-0.02673	0.533104	1
Cdk19	-0.02675	0.350545	1
Snx7	-0.02679	0.087587	1
D930016D06Rik	-0.0268	0.026435	1
Arl6ip6	-0.0268	0.98001	1
Gtf2a1	-0.02683	0.382604	1
Ppip5k2	-0.02684	0.057466	1
Icosl	-0.02684	0.378836	1
Myo6	-0.02685	0.767804	1
Cryl1	-0.02686	0.083945	1
Ywhag	-0.02686	0.434522	1
Gm26936	-0.02687	0.11392	1
Washc4	-0.02689	0.324775	1
Fam207a	-0.02689	0.301336	1
Pacs2	-0.02693	0.255321	1
Tpp2	-0.02695	0.690758	1
Ccdc122	-0.02695	0.451863	1
Zfp777	-0.02696	0.0521	1
Rala	-0.02696	0.587355	1
Ap3s1	-0.02696	0.330956	1
Slc4a7	-0.02699	0.451073	1
Masp1	-0.027	0.341831	1
Wdr34	-0.027	0.009753	1
Gpr146	-0.02701	0.542266	1
Napb	-0.02702	0.007086	1
Gab3	-0.02709	0.214641	1
2610044O15Rik8	-0.02709	0.095246	1
Mxi1	-0.0271	0.836001	1
Smyd2	-0.0271	0.121082	1
Tbl1x	-0.0271	0.476966	1
Rsad2	-0.0271	0.101627	1
Taf1	-0.02711	0.026445	1
Stk24	-0.02711	0.364381	1
Cd274	-0.02714	0.102794	1
Rnft1	-0.02715	0.858719	1
Glrx	-0.02716	0.111071	1
Chd9	-0.02716	0.233108	1
Prkaa1	-0.02719	0.3245	1
Pms1	-0.0272	0.07144	1

Wdr77	-0.0272	0.263413	1
Zfp658	-0.02721	0.786953	1
Polh	-0.02723	0.243844	1
Gemin7	-0.02724	0.876891	1
Slc39a11	-0.02729	0.135964	1
Traf6	-0.0273	0.743289	1
Dagla	-0.0273	0.129843	1
Mapk8ip3	-0.0273	0.021095	1
Fam135a	-0.02732	0.119294	1
B430306N03Rik	-0.02734	0.067676	1
Nup188	-0.02735	0.087457	1
Mxd1	-0.02736	0.121934	1
Vipas39	-0.02739	0.057305	1
Mink1	-0.0274	0.066561	1
Gm26724	-0.0274	0.04089	1
Hpgds	-0.0274	0.351857	1
Clpx	-0.02741	0.420096	1
Gm7480	-0.02744	0.016783	1
Gid4	-0.02745	0.270569	1
Slc45a4	-0.02746	0.249169	1
Letm1	-0.02747	0.180298	1
Mical3	-0.02749	0.524664	1
Sesn2	-0.02749	0.088683	1
Mss51	-0.0275	0.011928	1
Recql5	-0.0275	0.096072	1
Ipo8	-0.02753	0.419753	1
Tbc1d31	-0.02754	0.018389	1
Mrps6	-0.02756	0.164268	1
Rbm10	-0.02758	0.081921	1
Tmem120b	-0.02758	0.516284	1
Eif4g1	-0.02759	0.406863	1
Pms2	-0.0276	0.183771	1
Sacm1l	-0.02761	0.265049	1
Arid4b	-0.02761	0.724899	1
Ttc17	-0.02763	0.116132	1
Fam210a	-0.02764	0.472838	1
Nae1	-0.02764	0.403628	1
Snrk	-0.02766	0.184878	1
Sord	-0.02767	0.135185	1
Calhm2	-0.0277	0.389185	1
Irf5	-0.02772	0.542058	1
Uhmk1	-0.02773	0.884052	1
Sin3a	-0.02773	0.774927	1
Sltm	-0.02774	0.375063	1
Cacul1	-0.02775	0.281102	1
Sprtn	-0.02776	0.360384	1
Atg5	-0.02778	0.530814	1
Cdk5rap1	-0.02779	0.342612	1

Pter	-0.02781	0.416339	1
Gsap	-0.02784	0.117985	1
Atg16l2	-0.02785	0.514502	1
Scaf1	-0.02786	0.203913	1
Igf2bp3	-0.02788	0.36481	1
Pik3c3	-0.02788	0.218734	1
Brd1	-0.02788	0.397463	1
Dnajc16	-0.02789	0.556043	1
Ppp1r12a	-0.02791	0.14627	1
Slc6a6	-0.02792	0.57732	1
Tlr1	-0.02794	0.344648	1
Fam129a	-0.02798	0.465243	1
Tubb4b	-0.02803	0.227177	1
Angel2	-0.02803	0.620622	1
Vps53	-0.02804	0.717252	1
Gm15764	-0.02805	0.251716	1
Zfp385a	-0.02805	0.404992	1
Bpnt1	-0.02806	0.117166	1
Txlng	-0.02806	0.038405	1
Pgm3	-0.02807	0.028187	1
Ulk2	-0.02808	0.22393	1
Ptpra	-0.02808	0.189507	1
Llph	-0.02808	0.9495	1
B3glct	-0.02808	0.789673	1
Zfp746	-0.0281	0.052809	1
G2e3	-0.02812	0.089221	1
Lpcat1	-0.02812	0.350454	1
Abca7	-0.02812	0.47407	1
Pccb	-0.02812	0.147339	1
Tbc1d22a	-0.02814	0.088864	1
Gm34408	-0.02816	0.004615	1
Rin3	-0.02816	0.172802	1
Pskh1	-0.02818	0.051731	1
Casp12	-0.02818	0.052809	1
2510009E07Rik	-0.02819	0.467434	1
Rmdn3	-0.0282	0.103956	1
Zfp810	-0.02824	0.706494	1
Ddx39b	-0.02825	0.350536	1
Dock7	-0.02825	0.960192	1
Cc2d1b	-0.02828	0.494421	1
Orai1	-0.02828	0.626602	1
Fbxl18	-0.02828	0.002127	1
Cnot1	-0.02831	0.209028	1
Recql	-0.02834	0.116249	1
Ubfd1	-0.02837	0.45907	1
Ssbp1	-0.02837	0.22994	1
Rab8a	-0.02838	0.428491	1
Tmf1	-0.02838	0.945366	1

Poli	-0.02839	0.001398	1
Pias2	-0.02839	0.728432	1
Gm10130	-0.0284	0.029951	1
Wdr37	-0.02841	0.546525	1
Tab3	-0.02846	0.296404	1
Scamp1	-0.02848	0.592571	1
Gnl1	-0.02848	0.012506	1
Piezo1	-0.02849	0.342703	1
Atp10a	-0.0285	0.169006	1
Szrd1	-0.0285	0.437166	1
Psmg1	-0.02852	0.520705	1
Otulinl	-0.02854	0.090675	1
Ugcg	-0.02855	0.172518	1
Gatad2a	-0.02857	0.74525	1
Dcaf1	-0.0286	0.735298	1
Prmt5	-0.02862	0.04667	1
Osbpl1a	-0.02863	0.706876	1
Cdk5rap2	-0.02865	0.275737	1
Enoph1	-0.02865	0.033879	1
Epn1	-0.02866	0.112726	1
Capg	-0.02867	0.49638	1
Trip12	-0.02868	0.521044	1
Gtf3c2	-0.02873	0.762156	1
Pptc7	-0.02875	0.102051	1
Ubl7	-0.02876	0.010796	1
Tshz1	-0.02876	0.349435	1
Lrrc8a	-0.02877	0.263323	1
Erap1	-0.02877	0.415973	1
Cdk13	-0.02877	0.161135	1
Slc25a45	-0.02879	0.109095	1
Dync2h1	-0.0288	0.380442	1
Carmil1	-0.0288	0.652174	1
Eif4h	-0.02881	0.247476	1
Ugp2	-0.02883	0.164322	1
Akap10	-0.02883	0.412846	1
Gpr35	-0.02885	0.876927	1
Prdm2	-0.02885	0.667999	1
Zfp142	-0.02887	0.251705	1
Odf2l	-0.02887	0.036394	1
Ttc13	-0.02887	0.635844	1
Agps	-0.02888	0.326295	1
Med14	-0.02889	0.222879	1
Fnbp4	-0.02893	0.434309	1
Tdrd3	-0.02893	0.369287	1
Tab1	-0.02893	0.302153	1
Glmn	-0.02896	0.098268	1
Vps16	-0.02896	0.238153	1
Lpar6	-0.02898	0.321137	1

Os9	-0.02899	0.873664	1
Coro1a	-0.029	0.512932	1
Dnaja2	-0.02901	0.642823	1
Pank2	-0.02901	0.18022	1
Gm21057	-0.02902	0.073668	1
Gm15965	-0.02904	0.019889	1
Zfp280d	-0.02907	0.223379	1
Dpysl2	-0.02907	0.083565	1
Ist1	-0.02908	0.087604	1
Shb	-0.0291	0.30908	1
Gm13212	-0.02911	0.032922	1
Cish	-0.02912	4.87E-05	1
Ms4a4c	-0.02912	0.489199	1
Gm16091	-0.02913	0.023623	1
Mtmr12	-0.02914	0.476494	1
Rtn1	-0.02916	0.57127	1
Cryba4	-0.02916	0.560682	1
Smim13	-0.02917	0.111529	1
Arl6ip5	-0.02917	0.802717	1
Bahcc1	-0.02918	0.309826	1
Prdm15	-0.02918	0.002923	1
Zc3h7a	-0.02918	0.125179	1
Cebpg	-0.02919	0.417111	1
Uckl1	-0.02922	0.008362	1
Ramac	-0.02923	0.584999	1
Trappc10	-0.02925	0.130558	1
Relch	-0.02928	0.418782	1
Sf3b6	-0.02929	0.304683	1
Acp6	-0.02929	0.536414	1
Blm	-0.02929	0.331177	1
Il7r	-0.02933	0.283338	1
Rlf	-0.02933	0.377741	1
Tgfbrap1	-0.02934	0.343197	1
Ampd3	-0.02934	0.437844	1
Uchl3	-0.02934	0.170337	1
Nfrkb	-0.02934	0.208919	1
Ccl7	-0.02936	0.002694	1
Klc1	-0.02939	0.089503	1
4930402H24Rik	-0.0294	0.226716	1
Hip1	-0.0294	0.504453	1
Agpat1	-0.02941	0.075888	1
P2rx1	-0.02942	0.006308	1
Agtpbp1	-0.02944	0.190217	1
Mcu	-0.02944	0.09311	1
Ap3m2	-0.02944	0.142275	1
Hacl1	-0.02947	0.050329	1
Sik2	-0.02948	0.412748	1
Ypel5	-0.02948	0.373444	1

Ralgapb	-0.02949	0.328125	1
Aldh2	-0.02951	0.404875	1
Trip6	-0.02951	0.026826	1
Rnf181	-0.02953	0.207643	1
Daxx	-0.02953	0.063322	1
Gbp9	-0.02953	0.328805	1
Nfe2l2	-0.02956	0.176511	1
Eps8	-0.02956	0.025988	1
Msi2	-0.02956	0.6157	1
Ppp1cc	-0.02956	0.488146	1
Bmpr1a	-0.02957	0.41096	1
Dcaf17	-0.02957	0.514079	1
Eef2k	-0.0296	0.27218	1
Nek9	-0.0296	0.437941	1
Gm6034	-0.02961	0.122925	1
Ly6a	-0.02963	0.460234	1
Srp72	-0.02963	0.327872	1
Ncoa3	-0.02963	0.318656	1
Tsga10	-0.02963	0.98897	1
E130307A14Rik	-0.02964	0.922105	1
Ptrh2	-0.02967	0.134784	1
Ascc1	-0.02968	0.226233	1
Tbc1d24	-0.0297	0.186636	1
Ythdf1	-0.0297	0.116524	1
Znrf1	-0.02971	0.49088	1
Rbm14	-0.02972	0.044287	1
Riok2	-0.02974	0.898067	1
Rilpl1	-0.02976	0.816886	1
6330562C20Rik	-0.02979	0.088683	1
Tmem242	-0.02979	0.128395	1
Tgds	-0.02981	0.118438	1
Tjp1	-0.02983	0.118484	1
Gm49662	-0.02985	0.02261	1
Ccdc85c	-0.02985	0.00849	1
Pram1	-0.02986	0.002201	1
Elf1	-0.02987	0.073633	1
Acsl5	-0.02989	0.355146	1
Slc39a9	-0.02989	0.236453	1
Plekha2	-0.02992	0.267261	1
Trip11	-0.02992	0.727047	1
Sec22a	-0.02992	0.805758	1
Fam20b	-0.02995	0.069948	1
Enox2	-0.02995	0.009901	1
Elac1	-0.02996	0.14289	1
Tnfsf10	-0.02997	0.023884	1
Skp2	-0.02998	0.238838	1
Gm14966	-0.02999	0.023959	1
Arl2	-0.03	0.659754	1

Reep4	-0.03002	0.19777	1
Plekhb2	-0.03003	0.165357	1
C2cd5	-0.03009	0.54136	1
Gm26827	-0.0301	0.840588	1
Cpne1	-0.0301	0.166217	1
Ppp1r9a	-0.03012	0.630391	1
Lrrfip1	-0.03013	0.172808	1
Psme2b	-0.03014	0.289194	1
Abcd1	-0.03015	0.86034	1
Dcun1d3	-0.03015	0.164517	1
Pold3	-0.03018	0.341892	1
Asb8	-0.03019	0.264078	1
Ireb2	-0.03022	0.347908	1
Cln3	-0.03023	0.210383	1
Nmrk1	-0.03023	0.014399	1
Orc3	-0.03028	0.085413	1
Stau1	-0.03029	0.198048	1
Phf1	-0.0303	0.014804	1
Bcl2l11	-0.03031	0.895841	1
Glt8d1	-0.03031	0.275548	1
Raet1e	-0.03032	0.15918	1
Arl14ep	-0.03032	0.6252	1
Nfkb2	-0.03032	0.600976	1
Ogfr	-0.03033	0.957615	1
Wdr12	-0.03033	0.046353	1
Zbtb11	-0.03035	0.097088	1
Trap1	-0.03036	0.065338	1
Sos2	-0.03038	0.727631	1
Lmbr1	-0.03042	0.115292	1
Rab6a	-0.03043	0.780984	1
Arfip2	-0.03045	0.037505	1
Hars	-0.03052	0.018924	1
Ccl25	-0.03054	0.0493	1
Tm9sf3	-0.03057	0.310703	1
Zfp644	-0.03057	0.725347	1
Rcan1	-0.0306	0.172088	1
Relb	-0.03063	0.142434	1
Brd9	-0.03064	0.507646	1
Ccdc186	-0.03066	0.560857	1
Eif2ak2	-0.0307	0.181342	1
Gm11944	-0.03072	0.017871	1
Rassf5	-0.03072	0.206933	1
Ints9	-0.03073	0.212737	1
Atg4b	-0.03074	0.525207	1
Itfg1	-0.03074	0.686292	1
Kantr	-0.03074	0.136787	1
Prpf38b	-0.03076	0.346161	1
Det1	-0.03076	0.101903	1

Rnf220	-0.03079	0.13434	1
Mtm1	-0.0308	0.502784	1
Dlg4	-0.03081	0.041698	1
Map3k8	-0.03081	0.269695	1
Utp6	-0.03082	0.693606	1
Ext1	-0.03084	0.295513	1
AW554918	-0.03085	0.578711	1
Zcchc14	-0.03086	0.057435	1
Mex3c	-0.03087	0.179935	1
Pmm2	-0.03088	0.118182	1
Ppp1r18	-0.03088	0.990597	1
Fam118b	-0.03088	0.126589	1
Zfp976	-0.03089	0.034069	1
Tigar	-0.03089	0.046058	1
Cpne3	-0.03091	0.208799	1
Map3k20	-0.03091	0.420114	1
Rnf6	-0.03092	0.166951	1
1700037C18Rik	-0.03092	0.382132	1
Adck1	-0.03096	0.052304	1
Tti1	-0.03099	0.10805	1
Pcnx4	-0.03101	0.031283	1
Rbm4	-0.03102	0.516708	1
Foxj2	-0.03103	0.164538	1
Akna	-0.03107	0.412779	1
Rars2	-0.03109	0.176605	1
Fam98c	-0.03112	0.043775	1
Nlrp1b	-0.03113	0.05767	1
Ptpre	-0.03113	0.297361	1
Ttc9c	-0.03114	0.100551	1
Btbd1	-0.03114	0.488378	1
Ttc3	-0.03115	0.966768	1
Gemin8	-0.03117	0.266719	1
Taok2	-0.03118	0.127374	1
Taf2	-0.03119	0.202985	1
Nme7	-0.0312	0.009703	1
Gm49980	-0.0312	0.001504	1
Adat1	-0.03122	0.013762	1
Nt5dc1	-0.03125	0.571503	1
Utp25	-0.03128	0.0726	1
Gm50237	-0.03131	0.031838	1
Pmvk	-0.03133	0.064477	1
Gpatch8	-0.03135	0.520206	1
Sh2b1	-0.03137	0.230776	1
Adck2	-0.03139	0.104347	1
Stxbp2	-0.03145	0.187189	1
Pi4k2a	-0.03145	0.884103	1
Plekhm1	-0.03146	0.034077	1
Pde6d	-0.03148	0.145483	1

Nsun3	-0.03148	0.290616	1
Ncf1	-0.03153	0.412244	1
Ano6	-0.03154	0.849887	1
Tfdp2	-0.03155	0.140484	1
Cbx5	-0.03156	0.405037	1
Cstf3	-0.03159	0.20213	1
Snx4	-0.03161	0.404087	1
Tmem181a	-0.03163	0.83768	1
Prpf4	-0.03164	0.063068	1
Ube2k	-0.03166	0.152771	1
Oga	-0.03167	0.584959	1
Zbtb26	-0.03167	0.007788	1
D1Ertd622e	-0.03168	0.209971	1
Hmg20a	-0.03171	0.079396	1
Mecr	-0.03171	0.776217	1
Gpalpp1	-0.03174	0.327404	1
Cog4	-0.03174	0.183318	1
Xrcc1	-0.03174	0.017489	1
Speg	-0.03175	0.477033	1
Coa5	-0.03177	0.046624	1
Sirt6	-0.03177	0.005615	1
Prdm11	-0.03178	0.155031	1
Naa15	-0.03179	0.664087	1
Irf7	-0.0318	0.007799	1
Mettl1	-0.03181	0.290734	1
Ythdf3	-0.03183	0.312781	1
Prmt1	-0.03185	0.017565	1
Zzz3	-0.03185	0.133571	1
Rbbp8	-0.03187	0.499032	1
Pfkl	-0.03191	0.077007	1
Tsen34	-0.03192	0.23639	1
Sntb2	-0.03192	0.370925	1
Yars	-0.03194	0.293937	1
Ulk4	-0.03195	0.763167	1
Depdc5	-0.03196	0.259679	1
Scarb2	-0.03197	0.352525	1
Aopep	-0.03198	0.080091	1
Mdm2	-0.03199	0.381162	1
Usp33	-0.032	0.186259	1
Ikzf2	-0.032	0.167814	1
Gm10848	-0.032	0.984002	1
Polr3a	-0.03201	0.380788	1
Rint1	-0.03201	0.116189	1
Abhd17a	-0.03204	0.181633	1
Slc35b3	-0.03205	0.373511	1
Apobr	-0.03207	0.363065	1
Siglecf	-0.03207	0.191464	1
Vrk3	-0.03208	0.083986	1

Rsph3b	-0.03212	0.012688	1
Arfgef2	-0.03212	0.054513	1
Styx	-0.03213	0.129476	1
Msra	-0.03214	0.92434	1
Gm46218	-0.03214	0.330823	1
Klhdc10	-0.03216	0.089875	1
Parp2	-0.03216	0.259005	1
Uhrf2	-0.03217	0.068972	1
Zfp938	-0.03219	0.001092	1
Fan1	-0.03222	0.354557	1
Rabep1	-0.03222	0.114705	1
Stard7	-0.03223	0.283463	1
Tstd2	-0.03223	0.054444	1
Ikzf5	-0.03224	0.04938	1
Pstpip1	-0.03226	0.045791	1
Zfp518a	-0.03229	0.180786	1
Mob2	-0.03229	0.351682	1
Usp30	-0.03231	0.012268	1
Extl2	-0.03231	0.108852	1
Sptbn1	-0.03232	0.313884	1
Usp14	-0.03233	0.50621	1
Eif1b	-0.03234	0.481786	1
Ptpn6	-0.03236	0.37033	1
Pdxk	-0.03236	0.010007	1
Nr6a1	-0.03237	0.7993	1
Rnf135	-0.03238	0.106413	1
Capn1	-0.03239	0.012001	1
Atp6v1b2	-0.03239	0.548949	1
Wdr20	-0.0324	0.316315	1
Rbm33	-0.03241	0.830877	1
Ino80	-0.03241	0.268136	1
Rufy1	-0.03242	0.837531	1
Cyp4f18	-0.03242	0.307058	1
Map3k1	-0.03244	0.291481	1
Cul3	-0.03244	0.579422	1
Supt20	-0.03246	0.953118	1
Mpv17	-0.03246	0.023877	1
Ncoa7	-0.03247	0.02719	1
Trip4	-0.03247	0.202499	1
Araf	-0.03247	0.474642	1
Tor1aip2	-0.03248	0.341348	1
Mia2	-0.03248	0.381844	1
Gmip	-0.03249	0.192176	1
Zfp809	-0.0325	0.11719	1
Malat1	-0.0325	0.09112	1
Dlat	-0.03251	0.261498	1
Lsm2	-0.03253	0.040448	1
Tmem87b	-0.03255	0.261373	1

Slc25a22	-0.0326	0.007501	1
Gm4070	-0.03262	0.128721	1
Mpv17l	-0.03262	0.395673	1
Slx4ip	-0.03264	0.345114	1
Poldip3	-0.03267	0.13303	1
Cttnbp2nl	-0.0327	0.285016	1
Uap1	-0.03271	0.11979	1
Knop1	-0.03273	0.212279	1
R3hdm1	-0.03273	0.113379	1
Tmem71	-0.03273	0.037227	1
Eri3	-0.03274	0.066236	1
Mtmr6	-0.03274	0.038387	1
Ten1	-0.03276	0.161486	1
Prmt9	-0.03277	0.090606	1
Chil1	-0.03278	0.001908	1
Zdhhc14	-0.03278	0.913353	1
Iqce	-0.03279	0.001744	1
Kctd21	-0.03279	0.012264	1
Tecpr1	-0.03281	0.240579	1
E530011L22Rik	-0.03281	0.031428	1
Snrnp48	-0.03281	0.366391	1
Runx1	-0.03284	0.08253	1
Babam2	-0.03285	0.06902	1
Ccdc66	-0.03286	0.013081	1
Fhod1	-0.03287	0.528927	1
Rasa4	-0.03287	0.320567	1
Kdm1a	-0.03289	0.483159	1
Zc3h18	-0.03289	0.208727	1
Oas1a	-0.03293	0.08498	1
Irf3	-0.03293	0.121935	1
Acox1	-0.03293	0.066643	1
Brwd3	-0.03293	0.326445	1
Brd7	-0.03295	0.362821	1
Ythdc2	-0.03296	0.205895	1
Papolg	-0.03299	0.114022	1
Npepl1	-0.03302	0.091184	1
Zfp277	-0.03303	0.318152	1
Pex14	-0.03304	0.092996	1
Nmt2	-0.03307	0.916359	1
Incenp	-0.03308	0.069593	1
Ngrn	-0.03309	0.020666	1
Larp4	-0.03309	0.264883	1
Usp20	-0.03309	0.022368	1
Spata13	-0.0331	0.161201	1
Pcgf5	-0.03311	0.655703	1
Gm26520	-0.03313	0.279334	1
Heatr6	-0.03314	0.103673	1
Gm12353	-0.03314	0.014142	1

R3hdm2	-0.03314	0.181324	1
Apba1	-0.03319	0.248415	1
Dtx2	-0.0332	0.170403	1
Tor1b	-0.03321	0.127531	1
Slc30a6	-0.03323	0.064628	1
Gnl3l	-0.03324	0.008294	1
Gm45894	-0.03325	0.001401	1
Cyb5r4	-0.03326	0.142004	1
Vars	-0.03326	0.550819	1
Ift74	-0.0333	0.098216	1
Tbk1	-0.03332	0.468257	1
Agap3	-0.03332	0.075013	1
Clpb	-0.03335	0.339265	1
Ctdspl2	-0.03337	0.514025	1
Tmem135	-0.0334	0.35125	1
C330018D20Rik	-0.0334	0.0402	1
Cnnm2	-0.03342	0.941269	1
Gpc2	-0.03343	0.037958	1
Rab3gap1	-0.03346	0.334847	1
Timmdc1	-0.03348	0.063155	1
Arl5c	-0.03349	0.168028	1
Klhdc1	-0.0335	0.008672	1
Parp10	-0.03353	0.016797	1
Frmd8	-0.03354	0.133188	1
Tjp2	-0.03354	0.058652	1
Fbxl4	-0.03355	0.460057	1
Rbm6	-0.03357	0.098507	1
Zfp120	-0.0336	0.348373	1
Rnf34	-0.03363	0.546087	1
Ptpn11	-0.03364	0.281108	1
Nfat5	-0.03365	0.882566	1
Zfp579	-0.03367	0.139939	1
Coro7	-0.03367	0.243697	1
Rpgr	-0.03367	0.004026	1
Slc7a7	-0.03367	0.163083	1
Etv1	-0.03368	0.360726	1
Gm4924	-0.03369	0.365277	1
2610037D02Rik	-0.03371	0.244761	1
Hdac4	-0.03374	0.083193	1
Chfr	-0.03376	0.116615	1
Fbxl19	-0.03376	0.003893	1
Apopt1	-0.03377	0.075195	1
Dnajc24	-0.03379	0.039618	1
Cep192	-0.0338	0.505383	1
Anapc1	-0.03381	0.124177	1
Dis3	-0.03386	0.358549	1
Nup160	-0.03388	0.039495	1
Fam45a	-0.03388	0.445451	1

Flad1	-0.0339	0.274686	1
4930444A19Rik	-0.0339	0.024608	1
Rfx7	-0.03392	0.375933	1
Uso1	-0.03392	0.256075	1
Uba3	-0.03394	0.049061	1
Qtrt1	-0.03395	0.790164	1
D10Wsu102e	-0.03396	0.100695	1
Rnf10	-0.03396	0.271184	1
Psma2	-0.03399	0.22834	1
Anxa7	-0.03402	0.235875	1
Fbxl3	-0.03402	0.98244	1
Ccnk	-0.03403	0.072632	1
Agpat3	-0.03403	0.786245	1
Casp7	-0.03404	0.633125	1
Slc11a2	-0.03406	0.05772	1
Sec23ip	-0.03407	0.024295	1
Nbr1	-0.03411	0.088676	1
Cmklr1	-0.03413	0.198919	1
Armc8	-0.03416	0.036081	1
Phf3	-0.03416	0.932244	1
Fam114a1	-0.03417	0.060949	1
Pspc1	-0.03418	0.717075	1
Anxa11	-0.03419	0.008356	1
Capn7	-0.03421	0.242927	1
Pan3	-0.03421	0.301726	1
Zfp609	-0.03423	0.374774	1
Dennd4c	-0.03423	0.285852	1
Snx25	-0.03424	0.676184	1
Ndufa10	-0.03426	0.828159	1
Kbtbd2	-0.03426	0.704523	1
Itgb3bp	-0.03428	0.066837	1
Tmem106b	-0.03428	0.269695	1
Tfe3	-0.03428	0.430877	1
Rfwd3	-0.0343	0.167726	1
Lats1	-0.03431	0.277129	1
Megf9	-0.03433	0.020657	1
Sdhb	-0.03441	0.671408	1
I830077J02Rik	-0.03442	0.046728	1
Lima1	-0.03444	0.206304	1
Gpr180	-0.03444	0.245123	1
Kdm4c	-0.03445	0.349644	1
Zfp281	-0.03447	0.006374	1
Camkmt	-0.03448	0.349603	1
Map4k3	-0.03449	0.247776	1
Ppp2cb	-0.0345	0.039269	1
Ap1ar	-0.03452	0.310383	1
Ice1	-0.03455	0.367855	1
Fxr2	-0.03456	0.301426	1

Crk	-0.03458	0.117059	1
Cog3	-0.03462	0.063241	1
Wdr70	-0.03463	0.221335	1
Utp11	-0.03463	0.098271	1
Snhg20	-0.03465	0.018969	1
Paqr3	-0.03466	0.208802	1
Thap4	-0.03466	0.020772	1
Tcf20	-0.03468	0.58871	1
Sfxn5	-0.03469	0.077095	1
Nfxl1	-0.0347	0.071732	1
Xpo5	-0.03471	0.018963	1
Raph1	-0.03471	0.02046	1
Zkscan8	-0.03474	0.109209	1
Hspa12a	-0.03476	0.098509	1
Trio	-0.03476	0.338573	1
Orai2	-0.03476	0.494744	1
Dmtf1	-0.03478	0.176098	1
Atp6v0a2	-0.03479	0.245003	1
Dpy19l4	-0.03479	0.057536	1
Galm	-0.0348	0.097707	1
Dync1li2	-0.03481	0.383808	1
Otud6b	-0.03481	0.13241	1
Gsdmd	-0.03482	0.141702	1
Rbm18	-0.03485	0.481374	1
Ckap5	-0.03486	0.061407	1
Als2	-0.03487	0.470452	1
Rnf14	-0.03494	0.041288	1
Tpst2	-0.03495	0.118173	1
Atp2b1	-0.03497	0.298411	1
Atic	-0.03498	0.029254	1
Rheb	-0.03498	0.767117	1
Gm17435	-0.03501	0.093022	1
Inka2	-0.03502	0.042649	1
Ifit3b	-0.03503	0.012964	1
Crim1	-0.03505	0.443441	1
Pigl	-0.03508	0.225338	1
Gm46411	-0.03509	0.006257	1
Mrtfb	-0.03512	0.073057	1
Kcnk13	-0.03513	0.269738	1
Smurf2	-0.03514	0.184355	1
Glb1	-0.03514	0.121649	1
Plekha1	-0.03515	0.299384	1
Sdha	-0.03515	0.365812	1
Lars	-0.03517	0.455445	1
Naa60	-0.03517	0.523984	1
Nupl2	-0.03519	0.297028	1
Gemin5	-0.03519	0.112065	1
Vps13d	-0.03519	0.276177	1

Pou2f1	-0.03521	0.516231	1
Polr3gl	-0.03522	0.31004	1
Phactr4	-0.03528	0.21097	1
Tmx3	-0.03529	0.166121	1
Ift80	-0.03534	0.001541	1
Ndufs2	-0.03535	0.097252	1
Scap	-0.03537	0.317965	1
Tmem63b	-0.0354	0.036085	1
Otud7b	-0.03543	0.23122	1
Wdfy1	-0.03544	0.513423	1
2310001H17Rik	-0.03544	0.058514	1
Nifk	-0.03545	0.081013	1
Gng10	-0.03547	0.071632	1
Dync1i2	-0.03548	0.521164	1
Sap30	-0.03548	0.012328	1
Irf2bp1	-0.03549	0.000312	1
Rock2	-0.0355	0.051686	1
Gtf2e1	-0.0355	0.019104	1
Limk2	-0.03551	0.080665	1
Chmp4b	-0.03554	0.163106	1
Baiap2l1	-0.03557	0.000586	1
Sinhcaf	-0.03558	0.031639	1
H13	-0.03559	0.230074	1
Nckap1l	-0.0356	0.129973	1
Zcchc2	-0.03561	0.111598	1
4933423P22Rik	-0.03562	0.262384	1
Spout1	-0.03562	0.0332	1
Mfsd4a	-0.03562	0.008368	1
Zfp974	-0.03563	0.529921	1
Spata2	-0.03564	0.031694	1
Cebpd	-0.03565	0.166854	1
Maco1	-0.03566	0.149977	1
Cep78	-0.03566	0.027154	1
Dars2	-0.03567	0.00772	1
St3gal2	-0.03571	0.019804	1
Katna1	-0.03571	0.005176	1
Caml	-0.03572	0.093089	1
Setd5	-0.03572	0.055162	1
C030034L19Rik	-0.03572	0.103631	1
Creb1	-0.03577	0.185359	1
Arih2	-0.03581	0.118277	1
Arntl	-0.03581	0.183235	1
Atl2	-0.03582	0.079882	1
Prpf40b	-0.03582	0.006479	1
Stx4a	-0.03584	0.090272	1
Tbc1d32	-0.03584	0.058553	1
Gm17227	-0.03585	0.420616	1
Nfkb1	-0.03586	0.093608	1

Ttf1	-0.03587	0.197705	1
Sil1	-0.03589	0.549177	1
Sumf2	-0.03591	0.094229	1
Cacna1a	-0.03595	0.367079	1
Mtmr10	-0.03598	0.284116	1
Cs	-0.03599	0.067671	1
Atxn2l	-0.036	0.058948	1
Pak2	-0.03601	0.117646	1
Rhobtb2	-0.03601	0.090944	1
Mga	-0.03603	0.38256	1
Gm28375	-0.03603	0.114123	1
Slc30a9	-0.03605	0.22218	1
Rab22a	-0.03606	0.216551	1
Shprh	-0.03615	0.167677	1
Slc12a9	-0.03616	0.033329	1
6030442K20Rik	-0.03617	0.049003	1
Rab39	-0.03619	0.287051	1
Zfp217	-0.03619	0.116157	1
Safb	-0.0362	0.021543	1
Gtf2f2	-0.0362	0.071327	1
Trappc12	-0.0362	0.353918	1
Tgtp2	-0.03622	0.007986	1
Abcb1b	-0.03626	0.910871	1
Tomm70a	-0.03627	0.072596	1
Exoc2	-0.03627	0.388198	1
Opa3	-0.03629	0.043347	1
Myo9a	-0.03629	0.097472	1
Slc22a5	-0.0363	0.205441	1
Aven	-0.0363	0.004695	1
Got2	-0.03631	0.274034	1
Ube3b	-0.03636	0.337658	1
Aagab	-0.03636	0.162879	1
Ube2r2	-0.03636	0.303757	1
Ep400	-0.03639	0.372332	1
Tlr12	-0.03641	0.083928	1
Btbd10	-0.03642	0.039239	1
Nsun5	-0.03642	0.011717	1
Ppm1b	-0.03643	0.023011	1
Pgam5	-0.03643	0.063392	1
Apobec1	-0.03644	0.009222	1
Dtnbp1	-0.03645	0.569902	1
Tex2	-0.03645	0.218621	1
Gtpbp4	-0.03651	0.392098	1
Tet2	-0.03655	0.120246	1
Zfp131	-0.03655	0.461749	1
Pcgf6	-0.03656	0.02512	1
Fig4	-0.03658	0.333078	1
Uty	-0.03661	0.13209	1

Ubr1	-0.03665	0.112309	1
2810403D21Rik	-0.03666	0.023033	1
Rprd1a	-0.03666	0.055898	1
Tmc6	-0.0367	0.094029	1
Ptpn4	-0.0367	0.326065	1
Kitl	-0.03671	0.01891	1
Abcc1	-0.03674	0.743714	1
Erlin1	-0.03676	0.042308	1
Prcp	-0.03677	0.08226	1
Zcchc4	-0.03678	0.031962	1
Actr2	-0.03679	0.036942	1
Gm20404	-0.03679	0.031698	1
Kat6b	-0.03681	0.326286	1
Sh3bp4	-0.03682	0.005764	1
Clec2d	-0.03682	0.083396	1
Pnisr	-0.03683	0.083525	1
Mast3	-0.03683	0.563363	1
Rassf3	-0.03684	0.424749	1
Tm6sf1	-0.03686	0.632844	1
Capzb	-0.03686	0.127603	1
Clasrp	-0.03686	0.01812	1
Usp19	-0.03689	0.45329	1
Gapdh	-0.0369	0.997355	1
Brpf3	-0.03691	0.305774	1
Prkd3	-0.03691	0.354523	1
Aspscr1	-0.03692	0.019796	1
Bak1	-0.03693	0.026366	1
Zfp606	-0.03693	0.008137	1
Srgap2	-0.03694	0.030827	1
Nin	-0.03694	0.368525	1
Gpd1l	-0.03697	0.145599	1
Ext2	-0.03701	0.254082	1
Med26	-0.03702	0.134643	1
Cep85l	-0.03704	0.323453	1
Cdv3	-0.03705	0.280967	1
Zfp369	-0.03708	0.033768	1
Trak1	-0.03708	0.696153	1
Vps8	-0.03709	0.383459	1
Rnf141	-0.03711	0.014895	1
Rnf185	-0.03711	0.270461	1
Cdc37l1	-0.03713	0.060204	1
Nacc2	-0.03714	0.073505	1
Mipol1	-0.03717	0.573072	1
Nabp2	-0.03718	0.095165	1
Wdr43	-0.0372	0.215362	1
Usp21	-0.0372	0.007527	1
Crybg1	-0.03725	0.01762	1
Ccdc77	-0.03726	0.014315	1

Gm48765	-0.03729	0.039813	1
Tfdp1	-0.03729	0.036125	1
Ndst1	-0.0373	0.100191	1
Gm16759	-0.03731	0.027579	1
2010315B03Rik	-0.03737	0.046395	1
Cdk14	-0.03739	0.091038	1
Arpc1b	-0.03743	0.164169	1
Zcchc7	-0.03743	0.067085	1
Ptpn2	-0.03745	0.03047	1
Kat5	-0.03745	0.013215	1
Cog8	-0.0375	0.223171	1
Tspan7	-0.0375	0.439172	1
Kmt5c	-0.03752	0.000599	1
Ing2	-0.03753	0.035591	1
Zdhhc17	-0.03757	0.350226	1
Ccdc130	-0.03758	0.064068	1
Arid2	-0.03759	0.264204	1
Exoc3	-0.03764	0.383007	1
Impa2	-0.03764	0.127928	1
Mtx1	-0.03766	0.129392	1
Notch1	-0.03771	0.062089	1
Tcf3	-0.03772	0.129857	1
Pkd2	-0.03773	0.081256	1
Slc49a4	-0.03774	0.750209	1
Dap3	-0.03775	0.14453	1
Nfs1	-0.03778	0.26684	1
Krit1	-0.03783	0.142855	1
Myo9b	-0.03783	0.071928	1
Lrpprc	-0.03784	0.067656	1
Bmpr2	-0.03786	0.543738	1
Rilpl2	-0.03787	0.286863	1
Mir99ahg	-0.03788	0.244986	1
Spata5	-0.03789	0.3294	1
Gna11	-0.03791	0.01483	1
Gramd3	-0.03793	0.071864	1
Naa16	-0.03794	0.11699	1
Rsrc2	-0.03794	0.454158	1
Ttpal	-0.03799	0.052173	1
Trit1	-0.03799	0.333288	1
Tbp	-0.038	0.116102	1
H2-Q4	-0.03801	0.420864	1
Camsap1	-0.03802	0.277171	1
Nova1	-0.03808	0.187693	1
AW112010	-0.03815	0.984616	1
Cfap74	-0.03815	0.035799	1
Smc6	-0.03818	0.312463	1
Mob1a	-0.03825	0.23941	1
Chst11	-0.03826	0.458831	1

Tmpo	-0.03828	0.22904	1
Eif5b	-0.03831	0.149049	1
Sp110	-0.03832	0.042712	1
Cat	-0.03834	0.023888	1
Lncpint	-0.03834	0.805498	1
Hinfp	-0.03834	0.01868	1
Rsu1	-0.03841	0.30078	1
Cyp4f13	-0.03843	0.170291	1
Stard3	-0.03843	0.205909	1
Ubr2	-0.03843	0.037262	1
Evi5	-0.03846	0.390583	1
Zfyve9	-0.03849	0.22903	1
Irak2	-0.0385	0.788896	1
Cdh23	-0.03851	0.143798	1
Stambpl1	-0.03853	0.332165	1
Map4k5	-0.03856	0.269642	1
Tgfb1	-0.03859	0.050471	1
Plekhm2	-0.03859	0.102708	1
Letm2	-0.03866	0.000526	1
Fam49b	-0.03866	0.071496	1
Mttp	-0.03867	0.282872	1
Nipa2	-0.03867	0.098191	1
Alox5	-0.03872	0.309696	1
Gopc	-0.03873	0.00854	1
Lfng	-0.03875	0.084991	1
Lrrc8c	-0.03877	0.191974	1
Kdm6b	-0.03878	0.854475	1
Bfar	-0.03879	0.324699	1
Naa40	-0.03879	0.061909	1
Map3k10	-0.03881	0.012905	1
Rnf167	-0.03881	0.252554	1
Smg1	-0.03882	0.35711	1
Slco2b1	-0.03883	0.255888	1
Atp8b1	-0.03887	0.086578	1
Ralgapa1	-0.03889	0.163537	1
Necap1	-0.03893	0.006828	1
Abhd18	-0.03898	0.036002	1
Smchd1	-0.03898	0.050788	1
Ppp2r5c	-0.03902	0.088955	1
Arsg	-0.03908	0.442282	1
Rbl2	-0.03908	0.109636	1
Nrd1	-0.03911	0.415982	1
Slc25a44	-0.03913	0.025129	1
Ssh1	-0.03914	0.033398	1
Ctnnbl1	-0.03914	0.348254	1
Tent2	-0.03918	0.023734	1
Spred1	-0.03919	0.178658	1
Fbxo11	-0.0392	0.296931	1

Plekho1	-0.03923	0.125833	1
Bach1	-0.03929	0.776589	1
Pfdn1	-0.0393	0.156536	1
Sf3a3	-0.03933	0.069804	1
Lmf1	-0.03935	0.366619	1
Dedd	-0.03936	0.100118	1
Ano10	-0.0394	0.11613	1
Mthfsl	-0.03942	0.374368	1
Mphosph8	-0.03943	0.167988	1
Ssbp2	-0.03944	0.152216	1
Med1	-0.03946	0.094438	1
Dusp22	-0.03947	0.027553	1
Tdg	-0.03947	0.131281	1
A630001G21Rik	-0.03948	0.355627	1
Lrba	-0.03949	0.924275	1
Tmem131l	-0.03949	0.299285	1
Gart	-0.0395	0.460648	1
Med15	-0.03955	0.198663	1
Akap9	-0.03956	0.313139	1
Tnfrsf1b	-0.0396	0.070289	1
Ube3c	-0.03965	0.232818	1
Ccdc192	-0.03971	0.107908	1
Echdc1	-0.03972	0.010846	1
Map4	-0.03975	0.12917	1
Mettl6	-0.03976	0.135414	1
Pdxdc1	-0.03976	0.256563	1
Ypel2	-0.0398	0.355899	1
Ptpn1	-0.03982	0.218896	1
Prpsap2	-0.03982	0.213903	1
Hnrnph3	-0.03982	0.321766	1
Bcl7c	-0.03984	0.047882	1
Mff	-0.03984	0.116796	1
Ralgapa2	-0.03987	0.094448	1
Dapk3	-0.03992	0.228509	1
9330159M07Rik	-0.03992	0.054198	1
Maml1	-0.03993	0.386195	1
Vps13a	-0.03996	0.221139	1
Clint1	-0.03996	0.146913	1
Wnk1	-0.03997	0.043308	1
Zc3h13	-0.04	0.281808	1
Odf2	-0.04	0.028358	1
Ccdc50	-0.04002	0.059277	1
Xkr4	-0.04002	0.012918	1
Atf7ip	-0.04004	0.209466	1
Pisd	-0.0401	0.15876	1
Selenoi	-0.0401	0.088419	1
Dhx9	-0.04011	0.824935	1
Crebbp	-0.04013	0.07628	1

Cds2	-0.04013	0.110043	1
Fbrs	-0.04015	0.003656	1
Lifr	-0.04015	0.858247	1
Mpp1	-0.04016	0.44586	1
Bbx	-0.04016	0.228863	1
Rasgrf2	-0.04018	0.03108	1
Snx32	-0.04018	0.124467	1
Trim30b	-0.0402	0.002963	1
Ttc7	-0.04022	0.419496	1
Cdkl3	-0.04025	0.086067	1
Pwwp2a	-0.04027	0.247445	1
Uri1	-0.04029	0.287644	1
Gramd4	-0.04034	0.000558	1
Scly	-0.04038	0.043328	1
Fbxo34	-0.0404	0.152508	1
2310022A10Rik	-0.04041	0.00321	1
Thap12	-0.04047	0.256176	1
Ccar1	-0.04048	0.146931	1
Phf11b	-0.04052	0.097997	1
Gm30881	-0.04054	0.052629	1
Zbed4	-0.04055	0.298745	1
Snx6	-0.04057	0.088883	1
Cyth3	-0.04063	0.229101	1
Dse	-0.04066	0.057732	1
Mapkap1	-0.0407	0.177479	1
Zranb3	-0.04072	0.085493	1
Slc14a1	-0.04074	0.006904	1
Filip1l	-0.04074	0.429201	1
Eefsec	-0.04075	0.571967	1
Arhgef18	-0.04078	0.02188	1
Mgat4b	-0.04078	0.148784	1
Farsb	-0.04078	0.04368	1
Slc44a2	-0.04085	0.22154	1
Trim30c	-0.04086	0.004737	1
Senp2	-0.04086	0.204784	1
Rhobtb1	-0.04086	0.405877	1
Ptpn23	-0.04088	0.046566	1
Arnt	-0.04091	0.016075	1
Rps27rt	-0.04093	0.054916	1
Emsy	-0.04094	0.007362	1
Dhx30	-0.04094	0.070813	1
Mmp14	-0.04095	0.10135	1
Atg3	-0.04097	0.093228	1
Washc3	-0.04099	0.048507	1
Parp12	-0.041	0.052098	1
Cep57l1	-0.04101	0.111702	1
Cast	-0.04102	0.30961	1
Hhex	-0.04102	0.153025	1

Npepps	-0.04102	0.094685	1
Tex10	-0.04104	0.419914	1
Mcoln1	-0.04108	0.089672	1
Gm28981	-0.04109	0.011183	1
Loxl3	-0.0411	0.163909	1
Traf5	-0.04111	0.121661	1
Mbd6	-0.04113	0.01129	1
Dyrk1a	-0.04118	0.415952	1
5430427O19Rik	-0.0412	0.284106	1
Fam120a	-0.04121	0.045267	1
Gm50035	-0.04124	0.287798	1
Yes1	-0.04128	0.108909	1
Invs	-0.04131	0.163577	1
Galnt2	-0.04131	0.027668	1
Oprm1	-0.04136	0.004326	1
Traf3ip3	-0.04137	0.025495	1
Secisbp2l	-0.04138	0.269124	1
Socs5	-0.04141	0.078958	1
Slc16a10	-0.04143	0.316793	1
H2-T22	-0.04146	0.107385	1
Senp1	-0.04148	0.017552	1
Nrf1	-0.04148	0.069077	1
Tcf4	-0.04148	0.116899	1
Smg5	-0.04149	0.058532	1
Caap1	-0.04152	0.003734	1
Tmem161b	-0.04153	0.083091	1
Heatr3	-0.04156	0.132499	1
Arhgef2	-0.04157	0.036253	1
Zfand6	-0.04159	0.330886	1
Etnk1	-0.04165	0.060288	1
Lrrc8d	-0.04167	0.384123	1
Krcc1	-0.04174	0.214649	1
Ppp5c	-0.04174	0.097356	1
Ift52	-0.04178	0.334105	1
Pqlc2	-0.04187	0.010226	1
Kansl3	-0.04188	0.162486	1
Nipbl	-0.04189	0.23233	1
Prdm10	-0.04189	0.378262	1
Fem1c	-0.04189	0.627407	1
Gm4890	-0.0419	0.001057	1
Sfmbt1	-0.0419	0.055567	1
Dgkz	-0.04191	0.072733	1
Cdc14b	-0.04191	0.058808	1
Tmem168	-0.04191	0.265587	1
Idnk	-0.04192	0.037969	1
Fxyd5	-0.04195	0.513001	1
Cep112	-0.04197	0.085042	1
Dock4	-0.04197	0.011556	1

Rnf4	-0.04199	0.058641	1
Lekr1	-0.042	0.334535	1
Zkscan5	-0.04201	0.002604	1
Phkg2	-0.04202	0.041992	1
Acly	-0.04203	0.157632	1
Dctn5	-0.04205	0.207872	1
Fndc3a	-0.04205	0.067218	1
Dgat1	-0.04216	0.033064	1
Vta1	-0.04218	0.046787	1
Thumpd3	-0.04218	0.02189	1
Dcp1a	-0.04226	0.046747	1
Ppm1l	-0.04227	0.039214	1
Ube4a	-0.04228	0.114015	1
Rrp36	-0.04231	0.111117	1
Stag2	-0.04234	0.320777	1
Phf11d	-0.04242	0.009983	1
Atp6v0d1	-0.04242	0.294749	1
Mkrn1	-0.04243	0.107648	1
Rictor	-0.04244	0.246316	1
AC149090.1	-0.04245	0.113369	1
Col4a3bp	-0.04246	0.095317	1
Med13	-0.04248	0.09541	1
Bco2	-0.04248	0.047309	1
Trim25	-0.04251	0.022145	1
Trim33	-0.04263	0.441921	1
Rtp4	-0.04264	0.370821	1
Arfgap3	-0.04264	0.109635	1
Atxn3	-0.04267	0.241343	1
Rfc1	-0.04269	0.438959	1
Micu1	-0.0427	0.088344	1
Klhl28	-0.04272	0.043721	1
Iscu	-0.04272	0.150631	1
Cep164	-0.04272	0.087173	1
Mapkapk3	-0.04273	0.013604	1
Eml4	-0.04273	0.088573	1
Sergef	-0.04273	0.100693	1
Zfp646	-0.04273	0.005656	1
Ssbp3	-0.04276	0.018363	1
Ermard	-0.04279	0.111649	1
Pgap2	-0.04282	0.061751	1
Dock6	-0.04283	0.273952	1
Hipk3	-0.04283	0.781655	1
Fam102a	-0.04288	0.093946	1
Gipc1	-0.04289	0.005704	1
Phtf1	-0.0429	0.07667	1
Kdm3a	-0.04293	0.640794	1
Tor1aip1	-0.04297	0.015561	1
Senp8	-0.04299	0.009324	1

Ube2d1	-0.04299	0.174735	1
Arhgap4	-0.04307	0.728176	1
Mef2d	-0.04309	0.032168	1
Cmtm7	-0.04309	0.131911	1
Cryzl1	-0.0431	0.025173	1
Ppp6r2	-0.0431	0.220277	1
Ash1l	-0.04319	0.193113	1
Dcp1b	-0.04323	0.369449	1
Olfr56	-0.04324	0.00414	1
Zcchc24	-0.04325	0.102032	1
Mthfd2	-0.04327	0.161456	1
Ccdc25	-0.0433	0.206957	1
Cep68	-0.04332	0.036896	1
Eps15l1	-0.04338	0.072553	1
Psen2	-0.04341	0.058965	1
Adora3	-0.04347	0.121909	1
1700010I14Rik	-0.04347	0.033319	1
Serp1	-0.04352	0.468485	1
Xrcc4	-0.04353	0.04231	1
Rnasel	-0.04355	0.081599	1
Vps37a	-0.04358	0.341602	1
0610040J01Rik	-0.0436	0.223468	1
Setx	-0.04362	0.43442	1
Ahr	-0.04362	0.033355	1
Ifi209	-0.04364	0.088169	1
Bcar3	-0.04365	0.025363	1
Pfkfb4	-0.04366	0.364866	1
Kmt5b	-0.04372	0.317396	1
Fnbp1	-0.04373	0.017422	1
Dok1	-0.04377	0.003449	1
Myom1	-0.0438	0.032645	1
Tmem260	-0.04381	0.231388	1
Wipf1	-0.04381	0.03185	1
Cul4a	-0.04383	0.49376	1
Cnot4	-0.04384	0.168519	1
H2-T24	-0.04385	0.072107	1
Gm43713	-0.04388	0.103167	1
Stard8	-0.04388	0.229733	1
Fbxw7	-0.0439	0.02502	1
Hmces	-0.04393	0.029447	1
Sp1	-0.04394	0.093305	1
Lrch1	-0.04396	0.114388	1
Sec14l1	-0.04401	0.223789	1
Clock	-0.04401	0.19745	1
Nectin4	-0.04402	0.029346	1
Trim12a	-0.04403	0.415144	1
Fndc3b	-0.04408	0.272796	1
Carnmt1	-0.04409	0.005624	1

Por	-0.04412	0.024577	1
Itpr3	-0.04414	0.018855	1
Card11	-0.04414	0.005983	1
Tbc1d22b	-0.04417	0.101374	1
Gm42031	-0.04419	0.002943	1
Washc2	-0.0442	0.215496	1
Tut7	-0.04422	0.644132	1
Cerk	-0.04429	0.036783	1
Ncbp3	-0.04435	0.263011	1
Arpc1a	-0.04436	0.201635	1
Zfp26	-0.04436	0.063849	1
Lrig3	-0.0444	0.010015	1
1110002L01Rik	-0.04445	0.015638	1
Cep162	-0.04446	0.06128	1
Nup153	-0.04447	0.324847	1
Nagk	-0.04451	0.055741	1
Cox16	-0.04452	0.137971	1
Zfyve1	-0.04456	0.190661	1
Gm31763	-0.04456	0.000627	1
Cramp1l	-0.04461	0.049474	1
Rab6b	-0.04466	0.056256	1
Mtrex	-0.04467	0.183047	1
Ophn1	-0.04469	0.035764	1
Bcl9	-0.04471	0.016427	1
Tbl1xr1	-0.04474	0.079465	1
Taf4	-0.04475	0.141584	1
Atm	-0.0448	0.323103	1
Dhx58	-0.04486	0.008956	1
Gm11808	-0.0449	0.076672	1
Srrt	-0.04492	0.054932	1
Mecp2	-0.04492	0.093182	1
Plcb2	-0.04493	0.116403	1
Hspbap1	-0.04494	0.024257	1
Lrrc58	-0.04495	0.303411	1
Atp11c	-0.04497	0.168522	1
Chd8	-0.045	0.071863	1
Daam1	-0.045	0.030737	1
Zfp568	-0.04501	0.084992	1
Lrp12	-0.04504	0.76679	1
Strn3	-0.04506	0.11368	1
Hmox2	-0.04509	0.071871	1
Exog	-0.0451	4.65E-05	1
Pde4a	-0.04514	0.027116	1
Gm28501	-0.04515	0.010499	1
Rnf31	-0.04516	0.02797	1
Cnst	-0.04517	0.036269	1
Fut8	-0.0452	0.038324	1
Limd1	-0.04522	0.267914	1

Nadk2	-0.04523	0.032407	1
Ptpa	-0.04525	0.064663	1
Pdpr	-0.04529	0.084237	1
Gcnt7	-0.0453	0.001153	1
Soga1	-0.04535	0.21348	1
Phka1	-0.04535	0.018299	1
Sucla2	-0.04537	0.072367	1
Crebzf	-0.0454	0.07652	1
Taf5l	-0.04549	0.000767	1
Gm11508	-0.04551	0.017411	1
Arhgap17	-0.04551	0.039976	1
Ddx31	-0.04552	0.614408	1
Trpm2	-0.04553	0.088429	1
Inpp5b	-0.04554	0.047198	1
Parp4	-0.04558	0.194188	1
Dnm2	-0.04561	0.010393	1
Ogfrl1	-0.04564	0.196297	1
Thoc1	-0.04565	0.364877	1
Kdm5d	-0.04567	0.217976	1
Mob4	-0.04568	0.495829	1
Ttll5	-0.04568	0.151243	1
Pcca	-0.04571	0.085078	1
Heatr1	-0.04571	0.016856	1
Vwa8	-0.04572	0.499884	1
Ecd	-0.04573	0.111918	1
Lrrc41	-0.04574	0.111808	1
Smim14	-0.04576	0.16429	1
Scfd2	-0.04577	0.073772	1
Scaf8	-0.04579	0.089726	1
Prmt3	-0.0458	0.33954	1
Desi2	-0.04581	0.346604	1
Zfp738	-0.04585	0.007103	1
Ifit1	-0.04587	0.021426	1
Gm28286	-0.04591	0.000126	1
Snap23	-0.04593	0.117471	1
Kdm4a	-0.04593	0.003701	1
Adcy9	-0.04593	0.027157	1
Ylpm1	-0.04593	0.023477	1
Cand1	-0.04594	0.057452	1
Ggt5	-0.04596	0.119104	1
Pcf11	-0.04598	0.116376	1
Myo10	-0.046	0.000987	1
Fgd2	-0.04604	0.024646	1
Carm1	-0.0461	0.120979	1
Kctd5	-0.04611	0.071564	1
Smarca4	-0.04611	0.032759	1
Gm12743	-0.04615	0.003625	1
Add1	-0.04615	0.108108	1

Tubgcp3	-0.04619	0.019477	1
Zfp638	-0.04623	0.118395	1
Mllt6	-0.04625	0.002364	1
Itsn2	-0.04629	0.071684	1
Golph3l	-0.04634	0.064784	1
Kmt2c	-0.04635	0.246626	1
Anxa3	-0.04636	0.011084	1
Nploc4	-0.04637	0.003391	1
Actr1a	-0.04639	0.077771	1
Lrrfip2	-0.04639	0.04272	1
Trmt61b	-0.04639	0.000449	1
Bin3	-0.04645	0.036443	1
Zmynd8	-0.04646	0.041055	1
Zzef1	-0.04646	0.099854	1
Rps6ka2	-0.04646	0.014139	1
U2surp	-0.04647	0.473606	1
Coro1c	-0.04648	0.106931	1
Sdhaf2	-0.04652	0.03954	1
Pik3ip1	-0.04653	0.004951	1
Prr14	-0.04655	0.044158	1
Zyg11b	-0.04656	0.133385	1
Pacs1	-0.04659	0.24347	1
Galk2	-0.0466	0.104892	1
Mtif2	-0.04661	0.097815	1
Treml2	-0.04661	0.008013	1
Ahcyl2	-0.04665	0.0127	1
Gm15478	-0.04666	0.044264	1
St3gal5	-0.04667	0.1587	1
Retreg1	-0.04667	0.101281	1
Cdip1	-0.04674	0.056615	1
Zbtb40	-0.04674	0.006039	1
Pogz	-0.04675	0.113027	1
Ppp1cb	-0.04683	0.090342	1
Gpr65	-0.04684	0.185306	1
Large1	-0.04684	0.068631	1
Lcor	-0.04684	0.035423	1
Zfhx3	-0.04686	0.016538	1
Mta3	-0.04688	0.043369	1
Tmem273	-0.04689	0.175791	1
Nub1	-0.04689	0.28882	1
Nme6	-0.04691	0.13392	1
Pibf1	-0.04692	0.05498	1
Ttll3	-0.04693	0.012199	1
Prkdc	-0.04696	0.08874	1
Ap2a1	-0.04698	0.025471	1
Zbtb2	-0.04702	0.010056	1
Prpf40a	-0.04705	0.134656	1
Gm15523	-0.04706	0.001624	1

Dtd1	-0.04708	0.011907	1
Phf8	-0.04709	0.07019	1
Entpd7	-0.04712	0.008163	1
Mier1	-0.04715	0.225593	1
Snx29	-0.04715	0.029352	1
Atg16l1	-0.04721	0.145373	1
Anapc4	-0.04723	0.133488	1
Ccz1	-0.04726	0.142618	1
Commd1	-0.04726	0.49184	1
Ankrd27	-0.04726	0.05581	1
G6pdx	-0.04727	0.017637	1
Srcap	-0.04729	0.800341	1
Insyn2b	-0.04733	0.143368	1
Slc11a1	-0.04736	0.060399	1
Ap3m1	-0.04742	0.163826	1
Brox	-0.04742	0.080635	1
Tnfrsf13b	-0.04742	0.106676	1
Plagl2	-0.04742	0.036704	1
Luc7l	-0.04744	0.060237	1
2610020C07Rik	-0.04745	0.328825	1
Ankib1	-0.04748	0.014051	1
Fbxl2	-0.04749	0.008682	1
Usp46	-0.04749	0.115724	1
Khdc4	-0.04749	0.086471	1
Fgr	-0.04753	0.05118	1
Fanca	-0.04754	0.000137	1
Stam2	-0.04757	0.088927	1
Setdb1	-0.04758	0.092581	1
Slf2	-0.04763	0.159044	1
Sec24a	-0.04764	0.250439	1
Traf3	-0.04771	0.086797	1
Slc16a7	-0.04773	0.102116	1
Gak	-0.04775	0.076719	1
Plpp1	-0.04776	0.023926	1
Vcl	-0.04778	0.091012	1
Nup210l	-0.0478	0.000107	1
Fam120b	-0.0478	0.537294	1
Eapp	-0.04782	0.004003	1
Ap5m1	-0.04783	0.047424	1
Tmem189	-0.04784	0.118604	1
Tjap1	-0.04785	0.027905	1
Batf2	-0.04787	0.001197	1
Rcbtb2	-0.0479	0.770537	1
Clasp1	-0.04791	0.004387	1
Disp1	-0.04795	0.054428	1
Prpf6	-0.04798	0.171723	1
Mon1a	-0.04799	0.00014	1
Il27	-0.048	0.010561	1

Cbfb	-0.04801	0.224819	1
Mark4	-0.04804	0.039034	1
Bri3	-0.04806	0.732248	1
Cpeb4	-0.04809	0.077868	1
Wasf2	-0.0481	0.006053	1
Ccdc174	-0.0481	0.018734	1
2810004N23Rik	-0.04816	0.044322	1
Twf1	-0.04818	0.382678	1
Gtf2a2	-0.04819	0.084274	1
St3gal3	-0.04821	0.04869	1
Uba7	-0.04821	0.061718	1
Pde4dip	-0.04822	0.109687	1
Xiap	-0.04823	0.031137	1
Gm46367	-0.04827	0.383515	1
Stxbp4	-0.04828	0.002214	1
Txnrd1	-0.04828	0.628445	1
Zfp65	-0.04836	0.000111	1
Sult1a1	-0.04844	0.061773	1
Far1os	-0.04847	0.000898	1
Tmcc1	-0.04853	0.047882	1
Pcbd2	-0.04854	0.252304	1
Notch4	-0.04854	0.003613	1
Nfam1	-0.04857	0.035305	1
Hectd1	-0.04858	0.137574	1
Lipe	-0.04858	0.049031	1
Abca3	-0.04867	0.014911	1
Gm15564	-0.04869	0.006302	1
Fam120c	-0.0487	0.003815	1
B3galt5	-0.04879	0.043272	1
Stimate	-0.04882	0.3368	1
2510039O18Rik	-0.04887	0.068029	1
Lypla1	-0.04892	0.059491	1
Rnf41	-0.04894	0.011666	1
Ubap2l	-0.04897	0.014371	1
Rab5c	-0.04897	0.157226	1
Miip	-0.04897	0.014203	1
Pde1b	-0.049	0.009082	1
Gigyf1	-0.04902	0.160792	1
Ifi35	-0.04903	0.119308	1
Tbc1d20	-0.04905	0.033847	1
Ago3	-0.04908	0.084609	1
Wdfy4	-0.0491	0.046714	1
Pxk	-0.04911	0.116571	1
Tm9sf4	-0.04913	0.087442	1
2610507B11Rik	-0.04917	0.338792	1
Prmt7	-0.04919	0.068823	1
Brip1	-0.04921	0.024507	1
Zmym5	-0.04922	0.015318	1

Psd3	-0.04922	0.050466	1
Ppp1r15b	-0.04925	0.088874	1
Rttn	-0.04928	0.03286	1
Rest	-0.0493	0.02508	1
1500004A13Rik	-0.04933	0.10502	1
Gpbp1l1	-0.04933	0.009942	1
Ncoa2	-0.04934	0.146136	1
Zfp532	-0.04935	0.090708	1
Pdhx	-0.04937	0.003788	1
Elk4	-0.04941	0.061467	1
Lgals8	-0.04943	0.117721	1
Hmox1	-0.04945	0.316709	1
Slc24a3	-0.04947	0.256626	1
Mapkapk2	-0.04949	0.343812	1
Rapgef6	-0.04954	0.165457	1
Ccdc88b	-0.04955	0.03396	1
Snta1	-0.04958	0.016878	1
Phf14	-0.0496	0.044345	1
Gm10974	-0.0496	0.000522	1
Sos1	-0.04962	0.197178	1
A930015D03Rik	-0.04965	0.029153	1
Vamp5	-0.04968	0.013942	1
Amz1	-0.0497	0.507155	1
Anapc15	-0.04971	0.237418	1
Tab2	-0.04972	0.076265	1
Mad1l1	-0.04972	0.062497	1
Znrf2	-0.04974	0.107214	1
Tpcn2	-0.04977	0.002945	1
Ngly1	-0.0498	0.043401	1
Nfyc	-0.04983	0.079059	1
Pecr	-0.04987	0.180404	1
Zfp282	-0.04992	0.045484	1
Sh3pxd2a	-0.05003	0.073947	1
Rab3gap2	-0.05003	0.137291	1
Trim26	-0.05004	0.092905	1
Chordc1	-0.05004	0.063248	1
Tec	-0.05005	0.123706	1
2610203C22Rik	-0.05005	0.181814	1
Ltn1	-0.05005	0.019193	1
Zfp592	-0.05005	0.120551	1
Zfp871	-0.05006	0.062291	1
Cep290	-0.05007	0.01825	1
Wdr41	-0.05013	0.032616	1
Elavl4	-0.05015	0.028801	1
Ap1g1	-0.05025	0.133158	1
Gkap1	-0.05026	0.023799	1
Grk2	-0.05028	0.015776	1
Chchd4	-0.05029	0.017825	1

Rai14	-0.05033	0.094185	1
Rb1	-0.05033	0.108874	1
Rab21	-0.05038	0.076926	1
Kdm6a	-0.05049	0.196826	1
Zfp866	-0.05049	0.091214	1
Gvin1	-0.0505	0.036181	1
Pbrm1	-0.05053	0.119389	1
Klhl20	-0.05053	0.021548	1
Ehmt1	-0.05057	0.073225	1
Hira	-0.05061	0.18322	1
Zfp292	-0.05064	0.022556	1
Zfp945	-0.05065	0.04149	1
Ubap2	-0.05068	0.020915	1
Bbs9	-0.0507	0.065038	1
Lonp2	-0.05071	0.097028	1
N4bp2l2	-0.05071	0.272347	1
Csnk1a1	-0.05077	0.074936	1
Gpr107	-0.0508	0.247226	1
Slc25a20	-0.05091	0.048344	1
Arl5a	-0.05097	0.021984	1
Kdm5c	-0.05097	0.173151	1
Atf2	-0.05099	0.080555	1
Tpr	-0.051	0.872594	1
Dcaf12	-0.05101	0.043807	1
C5ar2	-0.05103	0.03194	1
Polr2c	-0.05106	0.191396	1
Gm42601	-0.05107	0.13207	1
Arhgap45	-0.05116	0.048992	1
Msrb1	-0.05117	0.003121	1
Egln1	-0.05118	0.166936	1
Asb3	-0.0512	0.280466	1
Jak1	-0.05121	0.108406	1
Max	-0.05124	0.169643	1
Smg6	-0.05127	0.039358	1
Dexi	-0.05129	0.000928	1
Bdp1	-0.0513	0.140032	1
Cep70	-0.05144	0.006354	1
Gm15563	-0.05144	0.274877	1
Tpi1	-0.05147	0.308924	1
Gmcl1	-0.05153	0.023787	1
Gabpb2	-0.05157	0.067081	1
Gm44148	-0.0516	0.001469	1
Khdrbs1	-0.05161	0.264301	1
Gatad2b	-0.05162	0.139841	1
Jpx	-0.05167	0.001476	1
Ppme1	-0.05168	0.019758	1
Pum2	-0.05175	0.120979	1
Cntrl	-0.05178	0.147124	1

Cdc14a	-0.05178	0.070427	1
Herc3	-0.0518	0.041101	1
Avl9	-0.0518	0.157376	1
Klhl2	-0.05181	0.014615	1
Zmynd11	-0.05182	0.356511	1
Entpd1	-0.05182	0.035367	1
Abhd15	-0.0519	0.078972	1
Zscan21	-0.05191	0.004519	1
Dcun1d1	-0.05194	0.077024	1
Cog5	-0.05194	0.060011	1
Zcchc17	-0.05196	0.05786	1
Qrich1	-0.05198	0.018456	1
Ube2j2	-0.05201	0.295243	1
Atxn7l1	-0.05207	0.153793	1
Smyd4	-0.05208	0.042564	1
Luc7l3	-0.05208	0.321722	1
Wapl	-0.05209	0.010816	1
Glce	-0.05213	0.002669	1
Kif13b	-0.05215	0.016197	1
Unkl	-0.05218	0.349211	1
Immp1l	-0.05219	0.016272	1
Med21	-0.05223	0.037978	1
Gm10134	-0.05224	0.000817	1
Chd2	-0.05226	0.331704	1
AW146154	-0.05227	0.006153	1
Shld1	-0.05228	0.096306	1
Stat3	-0.05232	0.024157	1
Rnf216	-0.05233	0.006917	1
Mre11a	-0.05235	0.024901	1
Upk1b	-0.05237	0.021387	1
Ptgr2	-0.05237	0.00443	1
Kif16b	-0.05242	0.160168	1
Nek7	-0.05242	0.00464	1
Chd7	-0.05244	0.014431	1
S100pbp	-0.05248	0.032782	1
Rasa2	-0.05259	0.121924	1
Abr	-0.05262	0.084365	1
Wdr47	-0.05262	0.041817	1
Tada3	-0.05266	0.03367	1
Gm7072	-0.05274	0.032685	1
Cdk12	-0.05274	0.020055	1
Cpeb3	-0.05279	0.059474	1
Strada	-0.05281	0.0249	1
Appl1	-0.05284	0.143162	1
Gm20045	-0.05285	0.001245	1
Asb13	-0.05286	0.001605	1
Wls	-0.05287	0.055057	1
Fam111a	-0.05288	0.012581	1

Acap2	-0.05289	0.024054	1
Bach2os	-0.0529	0.002411	1
Ogt	-0.05293	0.255875	1
Hnrnpa0	-0.05295	0.055337	1
Wdr44	-0.05295	0.209207	1
Plaat3	-0.05298	0.051488	1
Zfc3h1	-0.05298	0.040245	1
Ifih1	-0.05301	0.027093	1
Snx14	-0.05302	0.008926	1
Tmem243	-0.05307	0.073404	1
Prim2	-0.05311	0.08589	1
Wdr59	-0.05311	5.99E-05	1
Nlrp1a	-0.05316	0.029222	1
Tor3a	-0.05316	0.031169	1
Crlf3	-0.05319	0.030175	1
Gdap2	-0.05319	0.004058	1
Man2c1os	-0.05324	0.022745	1
Nck1	-0.05325	0.120912	1
Otud4	-0.05326	0.009912	1
Rsbn1l	-0.05329	0.094732	1
Arfgef1	-0.05331	0.04555	1
Nsmaf	-0.05336	0.066772	1
Trpv2	-0.05336	0.058715	1
Baz2b	-0.05346	0.110151	1
Gtf2ird2	-0.05348	0.014841	1
Rab7	-0.05357	0.009748	1
Faf1	-0.05361	0.013771	1
Pilra	-0.05365	0.305755	1
Trappc8	-0.05368	0.020683	1
Rit1	-0.0537	0.001342	1
Taok1	-0.05374	0.052808	1
Med13l	-0.05378	0.074666	1
Gapvd1	-0.05378	0.043497	1
Scmh1	-0.05379	0.462334	1
Zfp407	-0.0538	0.194177	1
Ubac2	-0.0538	0.095955	1
Nrp2	-0.05381	0.866666	1
Ube4b	-0.05381	0.072531	1
Tcf7l2	-0.05382	0.018631	1
Tnrc6a	-0.05384	0.075885	1
Ppp2r5e	-0.0539	0.107809	1
Zc3h7b	-0.05392	0.032945	1
Psma6	-0.05392	0.098333	1
Bcat2	-0.05403	0.011274	1
Zmat5	-0.05411	0.00466	1
Ap2a2	-0.05415	0.009653	1
Dhx8	-0.05418	0.203107	1
Osbpl9	-0.05418	0.19458	1

Tmem184b	-0.0542	0.037374	1
Smarcc1	-0.0542	0.132689	1
Soat1	-0.0542	0.316141	1
Afap1	-0.0542	0.001592	1
Ankrd44	-0.05434	0.022516	1
Paox	-0.05434	0.003026	1
Nxpe3	-0.05434	0.028249	1
Rfc2	-0.05436	0.06125	1
Slc25a38	-0.05439	0.008222	1
Neurl1a	-0.0544	0.015421	1
Iars	-0.05441	0.034639	1
Rc3h1	-0.05442	0.088858	1
Lap3	-0.05442	0.016607	1
Nabp1	-0.05445	0.003231	1
Zfp951	-0.05452	0.038486	1
Rogdi	-0.05454	0.213617	1
Gns	-0.05455	0.005081	1
Usp9x	-0.05456	0.068126	1
Ube2l3	-0.05459	0.15073	1
Rnf170	-0.0546	0.002487	1
Btbd9	-0.05462	0.063286	1
Gm43821	-0.05465	0.021683	1
Srbd1	-0.05465	0.040662	1
Spen	-0.05479	0.101606	1
Acbd3	-0.05483	0.019523	1
Pafah1b1	-0.05485	0.014301	1
Gpr137b	-0.05491	0.0161	1
Zfp703	-0.05491	0.01611	1
C87436	-0.05493	0.058641	1
Dlg1	-0.05497	0.397354	1
Virma	-0.05497	0.008285	1
Nacc1	-0.05498	0.000357	1
Htt	-0.055	0.014014	1
Sec23b	-0.05501	0.010868	1
Mtf1	-0.05512	0.012277	1
Jmjd6	-0.05513	0.012062	1
Hipk1	-0.05517	0.031277	1
Larp4b	-0.0552	0.147274	1
Jdp2	-0.0552	0.078775	1
Suco	-0.05525	0.199429	1
Camta1	-0.05532	0.057695	1
Picalm	-0.05533	0.01417	1
Vps13c	-0.05533	0.047247	1
Arhgef11	-0.05538	0.045331	1
Gse1	-0.05539	0.013676	1
Sorl1	-0.0554	0.161677	1
Thada	-0.05542	0.036545	1
2700049A03Rik	-0.05547	0.034169	1

Tg	-0.05551	0.005584	1
Upf2	-0.05552	0.056267	1
Acbd6	-0.05555	0.059025	1
1110051M20Rik	-0.05558	0.156914	1
Mapk1	-0.05559	0.015536	1
Kifap3	-0.05563	0.097488	1
Tfeb	-0.05566	0.033003	1
BC035044	-0.05567	0.00438	1
Ino80d	-0.05568	0.003576	1
Ankle2	-0.05568	0.020264	1
Fbxl20	-0.05572	0.028807	1
Dnaja3	-0.05577	0.013295	1
Ankrd12	-0.0558	0.04169	1
Hgf	-0.05587	0.297933	1
Ccdc191	-0.05588	0.002568	1
Nup155	-0.05588	0.026681	1
Amn1	-0.05602	0.090199	1
Meis1	-0.05604	0.049331	1
Sepsecs	-0.0561	0.098382	1
Fam117b	-0.05614	0.277033	1
Taco1	-0.05624	0.008612	1
Ddx3y	-0.05633	0.001282	1
Phtf2	-0.05638	0.036957	1
Patz1	-0.05642	0.001002	1
Arf3	-0.05648	0.004305	1
Cfdp1	-0.05651	0.490274	1
Nufip2	-0.05652	0.208739	1
Vps45	-0.05652	0.015426	1
Pdss2	-0.05657	0.033979	1
Igsf6	-0.05658	0.114262	1
Btk	-0.0566	0.081129	1
Gm40841	-0.05661	0.187566	1
Osbpl7	-0.05662	0.012012	1
4932438A13Rik	-0.0567	0.04648	1
Acot9	-0.05672	0.009195	1
Atxn2	-0.05677	0.191605	1
Ikzf1	-0.05682	0.004058	1
Upp2	-0.05682	0.006483	1
Supt3	-0.05684	0.101964	1
Zfyve26	-0.05684	0.023145	1
Atg4a	-0.05685	0.092346	1
AY512915	-0.05686	0.046293	1
Dnajc7	-0.05695	0.028395	1
Rabl6	-0.05699	0.04888	1
Ppp4r3a	-0.057	0.074827	1
Plcl2	-0.05701	0.060537	1
Hace1	-0.05702	0.006163	1
AB124611	-0.05705	0.076123	1

Tmod3	-0.05706	0.129225	1
Zfyve28	-0.05714	0.089271	1
Nt5m	-0.05715	0.016056	1
Kat14	-0.05719	0.006554	1
Gm4258	-0.0572	0.209656	1
Ikbkg	-0.05725	0.006469	1
Uimc1	-0.05726	0.122207	1
Tmem140	-0.0573	0.078106	1
Tmcc3	-0.05731	0.067705	1
Vav1	-0.0574	0.019485	1
5031425E22Rik	-0.0574	0.111183	1
Maea	-0.05742	0.135988	1
Gm2000	-0.05742	0.017997	1
Ubr4	-0.05743	0.059672	1
Ppm1k	-0.05744	0.001112	1
Rbms1	-0.05745	0.091342	1
Fchsd2	-0.05751	0.063623	1
Slc35a3	-0.05772	0.076175	1
Safb2	-0.05772	0.039843	1
Tra2a	-0.05776	0.018586	1
Myo1g	-0.05779	0.007071	1
Stim2	-0.0578	0.002433	1
Dmxl1	-0.0578	0.032326	1
Dnajc5	-0.05788	0.034172	1
Nfatc1	-0.0579	0.03423	1
Pcsk7	-0.05791	0.018374	1
Catsper2	-0.05792	0.021646	1
Sae1	-0.05794	0.224224	1
Tcirg1	-0.05795	0.014716	1
Slc30a7	-0.05796	0.001486	1
Pi4ka	-0.05798	0.010412	1
Ppfia1	-0.058	0.035799	1
Rnmt	-0.05815	0.015428	1
Smad3	-0.05817	0.334677	1
Lactb2	-0.05817	0.589932	1
2700097O09Rik	-0.05818	0.015188	1
Atg4c	-0.05819	0.002776	1
Ints3	-0.05822	0.00366	1
Herc1	-0.05824	0.044007	1
Ola1	-0.05825	0.039253	1
Pik3r5	-0.05837	0.015588	1
Rtn3	-0.05839	0.095933	1
Dpp8	-0.05841	0.157791	1
Gtf2i	-0.05845	0.000905	1
Wars	-0.05846	0.059602	1
Tent4b	-0.05852	0.064815	1
Ift88	-0.05854	0.051678	1
Ppp3cc	-0.05854	0.015724	1

Xpnpep1	-0.05854	0.073076	1
Arid1b	-0.05855	0.004429	1
Casp4	-0.05857	0.251542	1
Galnt10	-0.05863	0.002604	1
Pla2g7	-0.05866	0.003695	1
Ifi207	-0.05869	0.00469	1
Elmsan1	-0.05874	0.007149	1
Pggt1b	-0.05878	0.073758	1
Oas3	-0.05879	0.002755	1
Tmem170b	-0.0589	0.033805	1
Arhgap18	-0.05901	0.096954	1
Map3k5	-0.05907	0.012736	1
Zfp512	-0.05913	0.016942	1
Hltf	-0.05916	0.041067	1
Pikfyve	-0.05916	0.066389	1
Slc37a3	-0.05918	1.01E-05	0.326331
Itpr1	-0.05927	0.0007	1
Xpr1	-0.05929	0.039279	1
Zmiz1	-0.05932	0.016451	1
Dnajb6	-0.05934	0.009298	1
Tbcd	-0.05937	0.025122	1
Fam91a1	-0.05938	0.053939	1
Rcor1	-0.05939	0.008204	1
Foxn3	-0.05944	0.000235	1
Nisch	-0.05947	0.005495	1
Gramd1a	-0.05948	0.115602	1
Rbpms	-0.05951	0.057462	1
Srpk1	-0.05953	0.018505	1
Tmem126a	-0.05954	0.004388	1
Cyld	-0.05957	0.291932	1
Sumo1	-0.05958	0.018969	1
Slc9a8	-0.05961	0.168452	1
Erbin	-0.05961	0.022008	1
Stx18	-0.05961	0.119038	1
Cnot2	-0.05966	0.033289	1
E230029C05Rik	-0.05972	0.030152	1
Mtr	-0.05985	0.029814	1
Shtn1	-0.05987	0.01097	1
Zdhhc6	-0.05991	0.012204	1
Dip2c	-0.05998	0.101343	1
Slc38a7	-0.06001	0.009895	1
Chic2	-0.06001	0.001853	1
Hk2	-0.06003	0.012529	1
Ncoa1	-0.06009	0.009476	1
Dstyk	-0.06016	0.031988	1
Mafg	-0.06018	0.198449	1
Il18	-0.06018	0.001788	1
Scd2	-0.06019	3.28E-05	1

4931406P16Rik	-0.06037	0.309037	1
Mdfic	-0.06039	0.033491	1
Sp3	-0.06042	0.005624	1
Pgap1	-0.06042	0.012528	1
Sass6	-0.06046	0.00539	1
Lrp6	-0.06047	0.032691	1
Prrc2a	-0.0605	3.13E-05	1
Efl1	-0.06052	0.002184	1
Ankrd11	-0.06055	0.001057	1
Zmat1	-0.06061	0.012417	1
Mark3	-0.06061	0.052257	1
Slain2	-0.06064	0.033599	1
D130040H23Rik	-0.06066	0.024267	1
Bcl2a1d	-0.06067	0.044185	1
Cpped1	-0.06072	0.053853	1
Apaf1	-0.06072	0.165652	1
Cd40	-0.06073	0.068039	1
Tbc1d13	-0.06075	0.07359	1
D430042O09Rik	-0.06079	0.002857	1
Map2k5	-0.0608	0.019806	1
Tasor2	-0.06084	0.025411	1
Inpp4a	-0.06085	0.068741	1
Gm20470	-0.06087	0.002531	1
Tlr4	-0.06089	0.004146	1
Dis3l2	-0.0609	0.024554	1
Nsd3	-0.06093	0.012384	1
Dennd1c	-0.06093	0.16067	1
Usp35	-0.061	0.124824	1
Sesn1	-0.06108	0.001226	1
Atf6	-0.06108	0.063631	1
Arid5a	-0.0611	0.000976	1
Zfyve27	-0.06113	0.008288	1
Tmem209	-0.06114	0.007585	1
Thoc2	-0.0612	0.005477	1
Xpo6	-0.06121	0.007666	1
Fam193b	-0.06124	0.013621	1
Lin54	-0.06128	0.004924	1
Drg1	-0.06134	0.002073	1
Rnf180	-0.06151	0.022698	1
Urgcp	-0.06154	0.023978	1
Mkln1	-0.06158	0.002939	1
Mbtd1	-0.06158	0.071098	1
Xrn1	-0.06159	0.008251	1
Atrnl1	-0.06159	0.032491	1
Umad1	-0.06162	0.010142	1
Iqsec2	-0.06168	0.002382	1
Slc4a2	-0.0617	0.000163	1
Mprip	-0.06172	0.029514	1

Apbb2	-0.06184	0.054539	1
Pdk1	-0.06184	0.009804	1
Gm5086	-0.06186	0.482642	1
Trmt1l	-0.06187	0.001038	1
Rps6ka1	-0.06189	0.010457	1
Micu2	-0.0619	0.038068	1
Ubr5	-0.06195	0.023525	1
Stx12	-0.06196	0.086022	1
Nceh1	-0.06201	0.001469	1
Gna12	-0.06202	0.153725	1
Mysm1	-0.06204	0.115486	1
Tnpo1	-0.06211	0.014261	1
Litaf	-0.06213	0.069159	1
Msh3	-0.06213	0.1102	1
Tsc22d3	-0.06214	0.000818	1
Dcaf5	-0.06214	0.087485	1
Rhot1	-0.06218	0.010326	1
Rapgef1	-0.06225	0.002084	1
Lnpep	-0.06226	0.021993	1
Sgk3	-0.06226	0.087496	1
Gtpbp2	-0.06229	0.071328	1
Gng12	-0.06229	0.042614	1
Trpc4ap	-0.06235	0.018651	1
Fto	-0.06238	0.060016	1
Etv3	-0.0624	0.021171	1
Adhfe1	-0.06241	9.34E-05	1
Snx24	-0.06242	0.17981	1
Magi3	-0.06244	0.001265	1
Mbp	-0.06245	0.096483	1
Cdk17	-0.06257	0.066606	1
Usp7	-0.06258	0.024622	1
Ehbp1	-0.06264	0.052202	1
Mtmr3	-0.06268	0.023816	1
Ift140	-0.06269	0.00353	1
Tacc1	-0.06271	0.00269	1
Gm4841	-0.06276	0.001465	1
H2-Oa	-0.06277	0.022327	1
Tk2	-0.06283	0.002831	1
Nras	-0.06286	0.171769	1
Stmn1	-0.06289	0.33757	1
Akt3	-0.063	0.327989	1
Vrk2	-0.06302	0.00361	1
Slk	-0.06305	0.004267	1
Cdc42bpb	-0.06306	0.024943	1
Nod2	-0.06311	0.000302	1
Rnf115	-0.06315	0.025081	1
Gm37240	-0.06316	0.024264	1
Srpk2	-0.06318	0.170072	1

Wdr33	-0.06321	0.039685	1
Gbp3	-0.06322	0.017783	1
Dym	-0.06324	0.041514	1
Nemf	-0.06328	0.065062	1
Tep1	-0.06329	0.000417	1
Mavs	-0.06331	0.000397	1
Abcc4	-0.06335	0.006069	1
Nrip1	-0.06335	0.004792	1
Sbno2	-0.0634	0.01204	1
Pten	-0.0634	0.03051	1
B4galt4	-0.06343	0.037375	1
Itgal	-0.06344	0.001086	1
Il15	-0.06347	0.049024	1
Vapb	-0.06352	0.015135	1
Tyk2	-0.06352	0.010477	1
Btrc	-0.06357	0.035335	1
Cep85	-0.06369	0.002249	1
Tceanc2	-0.06372	0.001631	1
Gm16124	-0.06376	0.014381	1
3830406C13Rik	-0.06379	0.003761	1
Utrn	-0.06386	0.015478	1
Nedd4l	-0.06388	0.020086	1
Ambra1	-0.06395	0.000666	1
Ikbke	-0.06395	1.6E-05	0.516215
Gm16337	-0.06396	0.002106	1
Ppil2	-0.06405	0.003811	1
Isg15	-0.06413	0.045841	1
Siglecg	-0.06419	0.002801	1
Dclre1c	-0.0642	0.247714	1
Gm42962	-0.06424	0.048692	1
Fbh1	-0.06428	0.008065	1
Cdkn1a	-0.06433	0.021328	1
Mettl15	-0.06437	0.028465	1
Def6	-0.0644	0.006891	1
Dync1h1	-0.06442	0.026111	1
Zfp652	-0.06444	0.022394	1
Hectd4	-0.06452	0.055596	1
Akap13	-0.06458	0.003432	1
Iars2	-0.06461	0.023593	1
Gm40645	-0.06461	0.012004	1
Metap1	-0.06469	1.55E-05	0.499636
Crlf2	-0.06473	0.036908	1
Samd9l	-0.06475	0.013648	1
Stard9	-0.06479	0.016125	1
Map3k3	-0.06479	0.001891	1
Pex13	-0.0648	0.001749	1
Tlr2	-0.06489	0.006792	1
2210408F21Rik	-0.0649	0.003597	1

Atxn1	-0.06491	0.028818	1
Hipk2	-0.06497	0.062829	1
Dnajb14	-0.06497	0.235619	1
Spred2	-0.06499	0.043127	1
Strn	-0.06501	0.089649	1
Cd80	-0.06501	0.003455	1
Abcd2	-0.06501	0.238906	1
Nfia	-0.06504	0.01026	1
F830016B08Rik	-0.06504	0.041322	1
Lrmp	-0.06506	0.031849	1
2210408I21Rik	-0.06507	0.00561	1
Slc9a7	-0.0651	0.008127	1
Ube2h	-0.0651	0.082108	1
Nlk	-0.06512	0.010763	1
St6galnac6	-0.06514	0.00125	1
Rabgap1	-0.06521	0.024899	1
Flnb	-0.06524	0.046276	1
Rab11fip2	-0.06526	0.006957	1
Prex1	-0.06531	0.00364	1
Csnk2a2	-0.06532	0.011952	1
Zfp934	-0.06534	0.013661	1
Rffl	-0.06535	0.092886	1
Zfp451	-0.06545	0.032936	1
Gpbp1	-0.06548	0.01176	1
Mnat1	-0.06553	0.069509	1
Stard3nl	-0.06553	0.016158	1
Nup214	-0.06554	0.034824	1
Ifnar1	-0.06556	0.005743	1
B3galnt1	-0.06558	0.01909	1
D530033B14Rik	-0.0656	0.014695	1
Pde4b	-0.06563	0.011259	1
Btbd7	-0.06575	0.033829	1
Tbck	-0.06583	0.279738	1
Cers5	-0.066	0.034935	1
Il10ra	-0.066	0.000196	1
Rbm27	-0.06604	0.013292	1
Itga9	-0.06606	0.461753	1
Nup98	-0.06616	0.060862	1
Kif1b	-0.06618	0.055354	1
Fkbp15	-0.06618	0.002595	1
6530409C15Rik	-0.06624	8.86E-05	1
Ldah	-0.0663	0.018748	1
Ifi204	-0.06641	0.000322	1
Stxbp3	-0.06656	0.003364	1
Bckdhb	-0.06657	0.013858	1
Cspp1	-0.06658	0.043076	1
Kdm2a	-0.0666	0.020778	1
Rhbdf2	-0.06661	0.000356	1

Txndc16	-0.06663	0.154186	1
Smurf1	-0.06664	0.020368	1
Lsp1	-0.06665	0.014916	1
Ptk2b	-0.06668	0.005684	1
Cp	-0.06668	0.01108	1
Togaram1	-0.06676	0.091251	1
Dcaf6	-0.0668	0.00481	1
Trrap	-0.06685	0.005348	1
Sec24b	-0.06691	0.006204	1
Rnf111	-0.06692	0.156833	1
Tnip1	-0.06693	0.00082	1
Gm13710	-0.06694	0.053253	1
Evl	-0.06694	0.002233	1
Tnpo3	-0.06702	0.003565	1
Smarcc2	-0.06705	0.019634	1
Edem3	-0.06707	0.050349	1
Gm20429	-0.06708	0.000252	1
Slc12a6	-0.06713	0.015377	1
Heatr5a	-0.06721	0.001864	1
Sort1	-0.06722	0.036701	1
P2ry14	-0.06741	0.003844	1
Ehmt2	-0.06742	0.000404	1
Stxbp1	-0.06742	6.06E-06	0.195749
Fam193a	-0.06746	0.0028	1
Nudcd3	-0.0675	0.002433	1
Tasor	-0.06753	0.014522	1
Chchd3	-0.06753	0.002451	1
Aida	-0.06759	0.00014	1
Zmym6	-0.06759	0.000499	1
Ube2g1	-0.06763	0.042934	1
Paxbp1	-0.06769	0.003051	1
Tom1l2	-0.06778	0.004837	1
Ttc39b	-0.06779	0.028962	1
Ap3b1	-0.0679	0.003131	1
Socs2	-0.06793	0.303814	1
Ttc28	-0.06802	0.012575	1
Tmem175	-0.06803	0.025325	1
Cmip	-0.06805	0.004632	1
Eps15	-0.06807	0.025099	1
B430010I23Rik	-0.06813	0.503486	1
Tmem241	-0.06824	0.1362	1
Mgrn1	-0.06824	0.009	1
Arhgef7	-0.06826	0.00678	1
C2cd3	-0.06832	0.0006	1
Irf8	-0.06839	0.008531	1
Ilrun	-0.06841	0.009526	1
Ints6	-0.06845	0.001625	1
Zfp148	-0.06858	0.000358	1

Alpk1	-0.06859	0.001119	1
Xpo7	-0.06864	0.016654	1
Sppl3	-0.06866	0.002814	1
Aatf	-0.0687	0.053417	1
Phf20	-0.06876	0.007625	1
Gm20528	-0.06879	0.06439	1
Chd4	-0.06881	0.052591	1
Mthfr	-0.06883	0.000127	1
Gm34280	-0.06885	0.000462	1
A330023F24Rik	-0.0689	0.005963	1
Tyw1	-0.06894	0.006974	1
Phka2	-0.06894	0.007405	1
Ncor2	-0.06899	0.029237	1
Themis2	-0.069	0.005218	1
Bmf	-0.069	0.001883	1
Chd1	-0.06903	0.084494	1
Brd4	-0.06903	0.002095	1
Trim56	-0.06908	0.000853	1
Ric8b	-0.0691	0.000389	1
Sap25	-0.06913	0.002677	1
Manba	-0.06915	0.031574	1
Setd2	-0.06926	0.001756	1
Sh3bp2	-0.06931	0.006932	1
Cd2ap	-0.06951	0.001832	1
Kidins220	-0.06968	0.005286	1
Gm16599	-0.06971	0.066833	1
Ier3	-0.06977	0.345722	1
Rsbn1	-0.06985	0.005456	1
Fbxo42	-0.06986	0.008812	1
Zfp160	-0.06987	0.001744	1
2900089D17Rik	-0.06988	0.023561	1
Ppard	-0.06988	0.035772	1
Terf2	-0.06993	0.007613	1
Hcar2	-0.07004	0.0325	1
Cfb	-0.07004	0.027257	1
Gm42477	-0.07019	0.001115	1
Smad1	-0.0702	0.002406	1
Ctif	-0.07029	0.000726	1
Adcy7	-0.07032	0.246236	1
Adar	-0.07036	0.002575	1
Mettl8	-0.07044	0.001156	1
Gm35154	-0.07044	0.081508	1
Herc4	-0.07044	0.002467	1
Zbtb5	-0.07045	1.62E-05	0.522235
Prr5l	-0.07045	0.037794	1
Plxdc1	-0.07048	0.007125	1
Fam53b	-0.07052	0.000117	1
Vps35l	-0.07052	0.017656	1

Asb2	-0.07054	0.007264	1
Stk38	-0.07056	0.013164	1
Snx10	-0.07056	0.002562	1
Elp4	-0.07058	0.017768	1
Cd44	-0.07058	0.000746	1
Gramd1b	-0.07064	0.007633	1
Rbm26	-0.0707	0.047063	1
Trim12c	-0.07073	0.040657	1
Marf1	-0.07083	0.013735	1
Phf20l1	-0.07085	0.066914	1
Sptan1	-0.07085	0.009587	1
Zbtb38	-0.07089	0.000141	1
Gtpbp1	-0.07099	0.029808	1
Dido1	-0.07109	0.005026	1
Galnt1	-0.07117	0.004818	1
Ptprm	-0.07121	0.165925	1
R3hcc1l	-0.07122	0.002078	1
Nup93	-0.07124	0.002621	1
Fam49a	-0.0713	0.007793	1
Dst	-0.07134	5.14E-05	1
Numa1	-0.07137	0.00377	1
Nvl	-0.07137	0.016081	1
Nubpl	-0.07137	0.077958	1
Elf2	-0.07145	0.307795	1
Fli1	-0.07149	0.006161	1
Hps5	-0.07149	0.003951	1
Snx2	-0.0715	0.019757	1
Rab37	-0.07161	0.008436	1
Cep350	-0.07177	0.002773	1
Slfn9	-0.07177	0.000346	1
Mast4	-0.07181	0.083956	1
Mroh1	-0.07183	0.001205	1
Zfp438	-0.07186	0.002685	1
Sbno1	-0.07188	0.102559	1
Gng2	-0.0719	0.003994	1
Zc3h4	-0.07196	0.006756	1
Lpgat1	-0.07199	0.065385	1
Tdrd7	-0.07207	0.001966	1
Slc46a3	-0.07207	0.054436	1
Prorp	-0.07208	0.001289	1
Stx8	-0.07213	0.009822	1
Pdpk1	-0.07215	0.036674	1
Morc3	-0.07215	0.003515	1
Dlgap4	-0.07216	0.055694	1
Ppm1e	-0.07217	0.003569	1
Etv5	-0.07217	0.002991	1
Far1	-0.07229	0.004649	1
Socs7	-0.07233	0.000688	1

Smarcad1	-0.07234	0.017533	1
Tspan14	-0.07244	0.00568	1
Gm42047	-0.07248	0.007778	1
Lyn	-0.07249	1.33E-06	0.042966
Bcas3	-0.07264	0.00819	1
Armc9	-0.07267	0.008673	1
Sipa1l2	-0.0727	0.013236	1
Copg2	-0.07274	0.015155	1
Zmym2	-0.07278	0.001378	1
Wdr7	-0.07282	0.005015	1
Dock2	-0.07282	4.25E-07	0.013706
Slc12a2	-0.07294	0.044314	1
Fryl	-0.07296	0.005677	1
Kat2b	-0.07297	0.001658	1
Cyp27a1	-0.07297	0.004364	1
Tbc1d8	-0.07298	0.109868	1
Ppp6r3	-0.07303	0.002987	1
Golim4	-0.07304	0.034395	1
Atf7	-0.07315	0.005327	1
Pla2g4a	-0.07319	0.000755	1
Rbpj	-0.07329	0.033677	1
March5	-0.07341	0.007239	1
Ercc6l2	-0.07354	0.000288	1
Gm27017	-0.07358	3.64E-05	1
Banp	-0.07363	9.66E-06	0.311768
5330438D12Rik	-0.07366	0.012038	1
Cited2	-0.0737	0.030258	1
Tsc22d4	-0.07383	0.001542	1
Dop1b	-0.07384	0.005223	1
Adgre1	-0.07397	0.006954	1
Abcc5	-0.074	0.005712	1
Cep128	-0.07402	0.023504	1
Gm37494	-0.07407	0.044134	1
Agbl3	-0.07419	0.009755	1
1600014C10Rik	-0.07421	0.000838	1
Brd2	-0.07424	0.013527	1
Kmt2e	-0.07425	0.064078	1
Ipcef1	-0.07427	8.31E-05	1
Fyco1	-0.07428	0.005427	1
P2rx4	-0.07429	0.040065	1
Anxa6	-0.0743	7.42E-05	1
Vti1a	-0.07442	0.003131	1
Usp50	-0.07449	0.071993	1
Acsl4	-0.07452	0.001704	1
Tuba1b	-0.07463	0.025965	1
B3galnt2	-0.07464	4.05E-06	0.130649
Raf1	-0.07464	0.000901	1
Rbbp6	-0.07465	0.009218	1

Lrp5	-0.07468	0.013422	1
Zdhhc21	-0.07469	0.001253	1
Gm43813	-0.07483	0.00021	1
Celf1	-0.07484	0.001081	1
Fbxo33	-0.07504	0.000611	1
Pxn	-0.07524	0.001383	1
Ankfy1	-0.07525	0.012682	1
Rap2a	-0.07526	0.010645	1
Osbpl8	-0.07541	0.011047	1
Nfatc3	-0.0755	0.003967	1
Pip4k2a	-0.07553	0.000403	1
Zadh2	-0.07564	0.000354	1
Vav2	-0.07565	0.009343	1
Ndufs3	-0.07572	0.010067	1
Nsmce2	-0.07574	0.005609	1
Ap1s2	-0.07575	0.00366	1
Ranbp9	-0.07575	0.007893	1
1700012D14Rik	-0.07586	8.25E-06	0.266435
Fmnl1	-0.07593	0.000666	1
Tbc1d5	-0.07596	5.08E-05	1
Dars	-0.07603	0.008107	1
Gm31243	-0.07603	0.011947	1
Ep300	-0.07604	0.000327	1
Eya3	-0.07614	0.0062	1
Stim1	-0.07616	0.003102	1
Zfp950	-0.07626	4.63E-05	1
Sertad2	-0.0763	0.04084	1
Ddx17	-0.07635	0.004161	1
Tnrc6b	-0.07639	0.00124	1
Fmnl2	-0.07647	0.085922	1
Fbrsl1	-0.07658	0.000149	1
Eed	-0.07684	0.014274	1
1110019D14Rik	-0.07688	0.003297	1
Asxl2	-0.07696	0.006355	1
Rnf166	-0.07711	0.005277	1
5830444B04Rik	-0.07717	3.07E-05	0.989833
Tent5a	-0.07719	0.008823	1
Prkch	-0.07719	0.042468	1
Rufy2	-0.07723	3.35E-05	1
Rab11fip3	-0.07724	4.25E-05	1
Resf1	-0.07736	0.00465	1
Tln2	-0.07741	0.089376	1
Anapc16	-0.07742	0.006531	1
Tifa	-0.07748	0.00025	1
Kansl1	-0.07751	5.98E-05	1
Slc43a2	-0.07753	0.001716	1
Cers6	-0.07757	0.061846	1
Arih1	-0.07762	0.003952	1

Kmt2a	-0.07766	0.002464	1
Arhgef12	-0.07766	0.016454	1
Tango2	-0.07772	0.001443	1
Ubn2	-0.07773	0.00055	1
Dennd1a	-0.07777	0.002142	1
Tia1	-0.07781	0.004477	1
Tnfaip2	-0.07783	0.256697	1
Nuak1	-0.07794	0.017516	1
Ubash3b	-0.07795	0.000273	1
Larp1	-0.07796	0.00518	1
Rasa1	-0.07796	6.3E-05	1
Zeb1	-0.07802	0.002332	1
Zkscan3	-0.07802	0.000369	1
Slc36a1	-0.07831	0.00109	1
Mtmr14	-0.07837	0.003065	1
Ppp3ca	-0.07837	1.52E-05	0.491312
1110059E24Rik	-0.07839	0.003405	1
Ifi47	-0.07841	3.97E-06	0.128215
Zbp1	-0.07846	0.001277	1
Setd1b	-0.0786	0.001436	1
Me2	-0.07882	2.72E-05	0.877565
Anapc10	-0.07891	0.004179	1
Strbp	-0.07892	0.00417	1
Gm26510	-0.07898	0.005318	1
Nsd1	-0.07898	0.001169	1
Pias1	-0.07901	0.000138	1
Afg1l	-0.07911	0.00382	1
Ago2	-0.07916	0.000144	1
C4b	-0.07921	0.019881	1
Bcl2	-0.07926	0.12301	1
Mitf	-0.07936	0.006516	1
Ggact	-0.07957	8.71E-05	1
Naa25	-0.07977	4.39E-05	1
Exoc4	-0.07989	0.000545	1
Slc2a9	-0.07991	0.003033	1
Snd1	-0.07992	0.000843	1
Stat5b	-0.07997	0.047768	1
Ccdc62	-0.08014	0.028456	1
Foxn2	-0.08026	0.010367	1
Limk1	-0.0803	0.007123	1
Fbxw17	-0.08031	5.02E-05	1
Fgd3	-0.08038	0.01139	1
Cradd	-0.08056	0.181006	1
Tpst1	-0.08073	0.000241	1
Mertk	-0.08075	0.000596	1
Hs2st1	-0.08094	0.017903	1
Zfp992	-0.08101	0.013109	1
Man1c1	-0.08102	0.038952	1

Ptafr	-0.08104	0.001155	1
Pip4k2b	-0.08112	0.000604	1
Zfp516	-0.08115	0.010882	1
Riok3	-0.08117	0.001245	1
Xaf1	-0.08122	0.001076	1
Maz	-0.08153	5.98E-08	0.001931
Rad50	-0.08156	0.013001	1
Cdyl2	-0.08157	0.012467	1
Usp40	-0.08164	0.001682	1
Wbp1l	-0.08172	6.51E-05	1
Sik1	-0.0818	2.45E-07	0.007899
Ccdc6	-0.0821	0.000564	1
Ppp1r12b	-0.08218	0.000103	1
Rasa3	-0.0823	0.031984	1
Ube2f	-0.08247	0.024017	1
Gm12216	-0.0827	0.003826	1
Glg1	-0.08286	0.00042	1
Nbeal1	-0.08296	0.000864	1
Phc2	-0.08299	0.005861	1
Spire1	-0.08303	0.001948	1
Rab40c	-0.08326	0.002047	1
N4bp1	-0.08327	6.15E-05	1
Ddx58	-0.08338	0.005181	1
Ptprj	-0.08344	0.000161	1
Gnb1	-0.0835	0.000492	1
Itch	-0.08352	0.000236	1
St7l	-0.08364	0.000563	1
Cflar	-0.08375	0.000316	1
Frmd4a	-0.08376	6.79E-07	0.021911
Cop1	-0.08377	0.001391	1
Ankrd17	-0.08393	0.002806	1
Pnpla7	-0.08422	0.010565	1
D5Ertd579e	-0.0843	0.000524	1
Pld1	-0.08439	0.024304	1
Zfand3	-0.08448	0.000328	1
Hivep2	-0.0845	0.210842	1
Brd3	-0.08461	0.000428	1
Rreb1	-0.08469	1.41E-05	0.456714
Bcl2l1	-0.08474	0.003914	1
A830008E24Rik	-0.08475	0.017619	1
Celf2	-0.08477	0.000162	1
Hyal5	-0.08486	0.00021	1
Ankhd1	-0.08493	0.002454	1
Pacsin2	-0.08499	0.000722	1
Klf7	-0.08503	0.000646	1
Tap1	-0.08522	0.000102	1
Itpkb	-0.08524	0.000295	1
Bank1	-0.08528	0.0134	1

Birc3	-0.08551	0.000866	1
Rab10	-0.08553	0.001243	1
Phf12	-0.08565	0.004537	1
AU040320	-0.08571	0.001977	1
Cd69	-0.0858	0.026024	1
Dennd2a	-0.08591	3.27E-05	1
P4ha1	-0.086	0.049156	1
Prkag2	-0.08604	0.000579	1
A530072M11Rik	-0.08609	3.69E-06	0.119199
Il6ra	-0.08612	0.003536	1
Kif3b	-0.08613	0.00017	1
Rspry1	-0.08614	0.004966	1
Rbks	-0.08629	1.35E-05	0.434693
Abtb2	-0.08638	3.52E-05	1
Map2k1	-0.08644	3.48E-06	0.112482
St6gal1	-0.08647	0.114672	1
Mcmbp	-0.08648	0.009022	1
Ascc3	-0.08662	0.00141	1
Pid1	-0.0867	0.038372	1
Lcorl	-0.08675	0.000976	1
Tns3	-0.08679	0.000217	1
Maf	-0.08681	0.000196	1
Pkig	-0.08684	2.58E-05	0.831761
Foxk1	-0.08686	0.002798	1
Pla2g15	-0.08689	0.003035	1
Rnf38	-0.08693	0.00129	1
5430405H02Rik	-0.08718	0.000466	1
Ip6k1	-0.08718	6.89E-06	0.222544
Calhm6	-0.08754	5.89E-05	1
Prkcd	-0.08777	5.82E-05	1
Pds5a	-0.08794	0.005045	1
Pip5k1a	-0.08804	0.00229	1
Ppp4r1	-0.08816	0.001837	1
Mllt10	-0.0882	0.001242	1
Fgd4	-0.0882	0.003942	1
Tank	-0.08821	0.008542	1
Atg10	-0.0883	0.000739	1
Hk3	-0.08837	0.00222	1
Rab27a	-0.08841	0.000226	1
Syk	-0.08861	0.004231	1
Arel1	-0.08862	0.001336	1
Arhgef3	-0.08863	0.002402	1
Gnaq	-0.08865	9.92E-08	0.003204
Mob1b	-0.0891	0.000362	1
Npnt	-0.08918	0.165642	1
Abl1	-0.08923	0.002317	1
Klhl5	-0.08932	0.002218	1
Rgl1	-0.08933	0.006903	1

Znfx1	-0.08949	5.04E-05	1
Ewsr1	-0.08951	0.000171	1
Susd6	-0.0897	0.00022	1
Extl3	-0.08976	0.000275	1
Dock8	-0.08981	8.88E-05	1
Csnk1g3	-0.08989	0.027222	1
Jak2	-0.08996	0.000622	1
Slc25a12	-0.09007	0.003266	1
Tnfsf13b	-0.09039	0.00011	1
Dock10	-0.09041	3.77E-06	0.121679
Itpk1	-0.09044	9.32E-06	0.300821
Aak1	-0.0905	0.000172	1
Oasl1	-0.09058	0.001956	1
Eepd1	-0.09078	0.000769	1
Gm46224	-0.09079	0.000708	1
Cnot3	-0.0908	2.22E-05	0.716791
Fam241a	-0.09094	5.95E-07	0.019219
Axl	-0.09094	0.004852	1
Nadk	-0.09103	0.003054	1
Zfr	-0.0914	0.000983	1
Rcsd1	-0.09174	0.000906	1
Abcg1	-0.09179	0.00137	1
Tbxas1	-0.0918	0.000104	1
Ppp1r11	-0.09181	9.59E-07	0.030969
Mau2	-0.09184	0.001681	1
Pak1	-0.09195	0.000652	1
Zfp280c	-0.092	0.000539	1
Tmem104	-0.0922	0.000208	1
Mycbp2	-0.09225	9.32E-06	0.301028
Zfx	-0.09228	0.000794	1
Ranbp10	-0.09241	0.000746	1
Atp6v0a1	-0.09242	0.003638	1
Epsti1	-0.09245	9.5E-05	1
Cul1	-0.09273	0.000162	1
Rhoq	-0.09314	0.000291	1
Dock11	-0.09319	0.003565	1
N4bp2l1	-0.0932	0.000209	1
Prpf4b	-0.09325	0.014282	1
1700084C06Rik	-0.09326	0.000419	1
Ifi206	-0.0934	0.000396	1
Skap2	-0.09345	3.48E-05	1
Fcho2	-0.0935	0.000111	1
Rbm47	-0.09358	1.5E-05	0.484492
Gab2	-0.09367	7.72E-07	0.024923
Ppargc1b	-0.09368	2.45E-05	0.790097
Gon4l	-0.09369	0.000124	1
Tbc1d4	-0.09374	0.006269	1
Smg7	-0.09382	0.009103	1

Grk3	-0.09393	0.001069	1
Ap2b1	-0.09416	5.96E-05	1
Gm48678	-0.09434	0.001082	1
Swt1	-0.09441	0.001858	1
Gm26740	-0.09451	0.001478	1
Stk10	-0.09455	0.014939	1
Fam222b	-0.09456	0.00074	1
Maml2	-0.0947	8.71E-05	1
Dglucy	-0.09483	0.005243	1
Cept1	-0.09489	0.00049	1
Numb	-0.09512	4.17E-05	1
Phf21a	-0.09519	0.000377	1
Heatr5b	-0.09557	1.91E-05	0.617989
Gm20275	-0.09558	0.000685	1
Ubr3	-0.09572	0.000294	1
Rps6ka3	-0.09594	0.00125	1
Vcpip1	-0.09628	0.000225	1
Camk2d	-0.09662	1.92E-05	0.620834
Fbxw4	-0.09666	3.38E-06	0.109277
Gbf1	-0.09682	0.003283	1
Nfatc2	-0.09696	0.00923	1
Klhl18	-0.09697	1.14E-05	0.368638
Csf2rb	-0.09702	0.001181	1
Sufu	-0.09711	0.000239	1
Arhgef1	-0.09716	2.9E-05	0.936577
Abca9	-0.09716	0.002124	1
Peli1	-0.09722	1.38E-05	0.446004
Mrtfa	-0.09767	5.92E-06	0.19116
Gpi1	-0.09792	0.000324	1
Camk1d	-0.09799	5.01E-06	0.161859
Etv6	-0.098	1.8E-07	0.005807
Epb41	-0.09812	0.000212	1
Bicra	-0.09822	0.000494	1
H2-DMb1	-0.09825	0.011652	1
Sncaip	-0.09826	0.030999	1
Nod1	-0.09827	1.74E-07	0.005611
Gm43305	-0.09832	2.69E-05	0.868707
4732471J01Rik	-0.09859	0.000523	1
Lars2	-0.09873	0.000206	1
Nuak2	-0.09888	1.01E-05	0.325491
Diaph1	-0.09892	0.000307	1
Pml	-0.09939	0.000579	1
Wwox	-0.0998	0.006494	1
Rap1gds1	-0.09998	4.37E-07	0.014113
Clec16a	-0.10009	5.17E-05	1
Kcmf1	-0.10011	0.00042	1
Usp25	-0.10032	0.000224	1
Snhg16	-0.10037	4.42E-13	1.43E-08

Ston1	-0.10041	0.000206	1
Tiam1	-0.10056	0.004348	1
Ptpn9	-0.10073	0.001529	1
Gm12185	-0.10086	0.000381	1
Spop	-0.10094	0.000136	1
Madd	-0.10095	0.000185	1
Clcn7	-0.10105	9.77E-05	1
Ripk1	-0.10138	0.000207	1
9930111J21Rik2	-0.10147	3.73E-06	0.120458
Zfp106	-0.10165	0.000897	1
Zbtb34	-0.10172	2.26E-05	0.728055
Vcam1	-0.10179	0.016371	1
Plxdc2	-0.10179	3.07E-08	0.000991
Dennd1b	-0.10193	0.000366	1
Slfn8	-0.10194	0.000242	1
Dleu2	-0.10199	1.58E-05	0.509715
Prkca	-0.10207	8.66E-05	1
Asxl1	-0.10218	0.009117	1
Phkb	-0.10231	0.000305	1
Trafd1	-0.10243	2.45E-05	0.79019
Dapp1	-0.10272	0.000151	1
March7	-0.10276	0.000207	1
Pik3r6	-0.10303	0.000264	1
Dtx3l	-0.10316	1.08E-05	0.347081
Rmnd1	-0.10326	8.29E-06	0.267675
Fam219a	-0.10338	0.002268	1
Tbc1d1	-0.10372	0.008068	1
Zbtb7a	-0.10398	0.000143	1
Trim34a	-0.10445	5.92E-05	1
Kdm7a	-0.10495	1.97E-05	0.637516
Arhgap12	-0.10499	0.000461	1
Igtp	-0.10546	8.74E-05	1
Icam1	-0.10549	0.023766	1
Slc2a3	-0.10553	2.09E-06	0.067463
Zfp40	-0.10579	3.19E-05	1
Clybl	-0.10586	0.013386	1
Msr1	-0.10599	3.24E-05	1
Elf4	-0.10644	0.00036	1
Man2a1	-0.10667	0.000127	1
Csf3r	-0.10697	3.22E-06	0.104118
Gm49797	-0.10703	1.05E-05	0.337904
Dennd6a	-0.10724	0.000353	1
Stx16	-0.1074	3.34E-05	1
Akap8l	-0.10742	2.5E-05	0.805752
Rprd2	-0.10748	0.000155	1
Arid4a	-0.10768	5.97E-05	1
Mark2	-0.10769	0.000107	1
Aftph	-0.10786	0.000557	1

Gm35188	-0.10793	0.0242	1
Fgfr2	-0.10803	0.000109	1
Tnrc18	-0.10822	0.001832	1
Rnf150	-0.1083	0.005437	1
L3mbtl3	-0.10836	7.01E-06	0.226159
Asap1	-0.10843	4.13E-08	0.001334
Usp18	-0.10865	2.86E-05	0.923441
Sla	-0.10886	0.01297	1
Tnfrsf11a	-0.10894	0.0007	1
Rab43	-0.10917	5.77E-05	1
H2-T23	-0.10925	2.14E-05	0.690484
Sgip1	-0.10944	0.002184	1
Arhgap39	-0.10945	5.54E-05	1
Rufy3	-0.10987	0.000896	1
Zc3h12c	-0.10991	0.008257	1
Nfkbia	-0.10993	9.03E-07	0.029153
Plcg2	-0.11001	1.54E-06	0.049569
Prkcb	-0.11056	1.24E-05	0.40122
Rnpep	-0.11107	4.84E-06	0.156237
Pvt1	-0.11108	3.06E-06	0.098907
Tet3	-0.11144	3.95E-06	0.12758
Gm17106	-0.11261	4.17E-05	1
Tgfa	-0.11294	6.85E-06	0.221245
Crebrf	-0.11343	0.000198	1
Dna2	-0.11345	0.000289	1
Rras2	-0.11378	0.013903	1
Wdfy2	-0.1138	7.92E-06	0.255696
Plekhm3	-0.11381	8.64E-05	1
Fnip2	-0.11406	0.016089	1
Pim1	-0.1141	0.055152	1
Vgll4	-0.11417	0.000126	1
Gphn	-0.11424	0.000914	1
Sp100	-0.11451	1.8E-05	0.579683
Fgl2	-0.11454	1.85E-07	0.005973
Socs1	-0.11454	7.44E-09	0.00024
Uvrag	-0.11461	3.14E-08	0.001013
Stk4	-0.11466	3.41E-07	0.01101
Rps6kb1	-0.11471	9.62E-06	0.310426
Ifi213	-0.11511	7.75E-08	0.002503
Mthfs	-0.11536	1.16E-05	0.373508
Adap2os	-0.1157	6.37E-05	1
Slc31a1	-0.1159	1.02E-07	0.003309
Acer3	-0.11594	1.35E-07	0.00437
Myo1f	-0.11617	5.54E-08	0.001788
Arid1a	-0.11688	1.08E-08	0.000349
Gbp7	-0.11698	1.2E-07	0.003865
Sh2d3c	-0.11719	6.12E-07	0.019745
Mbnl1	-0.11747	4.11E-08	0.001327

Bicral	-0.11811	2.93E-07	0.009469
Slc38a6	-0.11837	0.00176	1
Lcp2	-0.11849	1.24E-06	0.040194
Slc1a3	-0.11862	0.000168	1
Pik3ap1	-0.11885	6.54E-07	0.021108
Cadm1	-0.11923	0.008487	1
Rassf4	-0.11962	6.07E-07	0.019586
Gbp6	-0.11965	7.28E-06	0.235103
Cbl	-0.11967	2.08E-07	0.006718
Pik3cd	-0.11967	1.88E-05	0.607587
Irs2	-0.11978	0.00017	1
Hs3st3b1	-0.12011	0.000123	1
Oas2	-0.12034	0.000129	1
Mllt3	-0.12049	5.34E-05	1
Exoc6	-0.12141	5.49E-07	0.017712
Tns1	-0.12154	8.77E-05	1
Rapgef2	-0.12177	0.000111	1
A930037H05Rik	-0.1222	0.000173	1
Notch2	-0.12222	1.52E-05	0.490231
Ubtd1	-0.12268	1.21E-05	0.391722
Foxo3	-0.12336	1E-05	0.322766
Mgmt	-0.1236	0.025921	1
Slfn5	-0.12383	0.001011	1
Taok3	-0.12403	1.92E-08	0.000621
Atp7a	-0.12469	4.31E-08	0.001393
Synj1	-0.12479	1.54E-08	0.000497
Atp8a1	-0.12507	2.02E-10	6.54E-06
Tlr7	-0.1253	6.6E-06	0.213103
Irf2	-0.12548	6.51E-08	0.002101
Irf1	-0.12553	1.53E-05	0.493831
Rab8b	-0.12562	0.000125	1
Arhgap25	-0.12568	1.6E-07	0.005156
Hck	-0.12569	2.25E-08	0.000728
Lyst	-0.12774	4.95E-06	0.159881
Rp2	-0.12788	3.98E-05	1
Atg7	-0.12789	5.95E-07	0.019217
Sfi1	-0.12851	1.2E-09	3.87E-05
Zc3hav1	-0.12855	5.48E-06	0.176806
Vps54	-0.1289	8.81E-09	0.000285
A330040F15Rik	-0.12972	2.79E-07	0.009018
Apobec3	-0.13007	4.29E-07	0.013863
Sipa1l3	-0.13009	5.64E-08	0.001822
Lrrc4	-0.13053	0.000718	1
Rere	-0.13138	9.6E-10	3.1E-05
Parp8	-0.13168	1.57E-05	0.507964
E230001N04Rik	-0.13267	7.21E-20	2.33E-15
Eif4g3	-0.13271	8.58E-09	0.000277
Jade2	-0.13293	9.79E-08	0.00316

Nsf	-0.13328	3.23E-05	1
Sdccag8	-0.13443	2.53E-08	0.000818
Selenow	-0.1347	6.69E-09	0.000216
Il15ra	-0.13491	6.01E-06	0.193915
Hpcal1	-0.13496	7.29E-08	0.002352
Tbc1d14	-0.13668	4.81E-08	0.001553
Lmln	-0.13683	0.000493	1
Tbc1d9	-0.13712	2.43E-05	0.783004
Cacna1d	-0.13831	2.3E-05	0.743513
Gm2245	-0.13984	1.45E-05	0.46826
Anks1	-0.13986	1.34E-07	0.004337
Mapk14	-0.14166	1.31E-08	0.000424
Parp11	-0.14356	4.01E-07	0.012959
Dnmt3a	-0.14432	3.66E-11	1.18E-06
Hdac9	-0.14537	0.002216	1
Aoah	-0.146	0.007522	1
Parp9	-0.14794	4.48E-08	0.001445
Zfp710	-0.14809	9.9E-12	3.2E-07
Ddhd1	-0.14896	5.94E-06	0.191632
Psmb7	-0.14919	1.79E-13	5.78E-09
March1	-0.15078	3.07E-09	9.91E-05
Trim30a	-0.1521	8.08E-10	2.61E-05
Ccnd2	-0.15213	4.34E-09	0.00014
Trim14	-0.15219	2.99E-08	0.000965
Osbpl11	-0.1526	9.27E-05	1
Frmd4b	-0.15263	9.19E-13	2.97E-08
Rnf169	-0.15307	1.21E-07	0.003894
Gm22146	-0.15345	2.34E-14	7.56E-10
Pik3r1	-0.15376	7.39E-11	2.39E-06
Tmem164	-0.15558	6.65E-07	0.021465
St6galnac2	-0.1557	1.21E-69	3.9E-65
Cnr2	-0.15657	2.69E-09	8.69E-05
Nrros	-0.1567	1.07E-11	3.44E-07
Abcg3	-0.15687	7.97E-05	1
Lrch3	-0.15699	3.73E-10	1.2E-05
Ssh2	-0.15749	1.67E-12	5.4E-08
Ppm1h	-0.15801	1.41E-13	4.54E-09
Nrp1	-0.15859	4.53E-07	0.014641
Ppp1r21	-0.15864	7.38E-08	0.002384
Mlxip	-0.1591	6.08E-08	0.001962
Lysmd4	-0.15981	1.59E-05	0.513587
Arhgap15	-0.16058	0.004207	1
4930599N23Rik	-0.16144	9.11E-12	2.94E-07
Klf13	-0.16214	3.46E-06	0.111632
Ripor2	-0.16219	1.57E-06	0.050537
Cdk8	-0.16317	6.18E-14	2E-09
Oasl2	-0.16423	5.8E-07	0.018728
Ifi211	-0.16426	9.9E-10	3.19E-05

Prr14l	-0.16459	3.07E-09	9.92E-05
Ccm2	-0.16463	6.23E-09	0.000201
Ddx60	-0.16514	0.000532	1
Nol4l	-0.16521	0.000294	1
Slfn2	-0.16572	4.7E-09	0.000152
Parp14	-0.16573	2.18E-10	7.05E-06
Coro2a	-0.16612	1.29E-06	0.041505
Rsrp1	-0.16654	1.57E-11	5.08E-07
Arsb	-0.16895	2.44E-05	0.787135
Stat2	-0.16916	1.72E-10	5.56E-06
Gm26917	-0.16959	0.862007	1
Fyb	-0.17042	7.68E-14	2.48E-09
Nlrc5	-0.17247	1.12E-11	3.61E-07
Nampt	-0.17424	4.67E-10	1.51E-05
Morrbid	-0.17449	1.47E-06	0.047412
Zeb2	-0.17488	6.35E-15	2.05E-10
Herc6	-0.17662	2.11E-07	0.006798
B4galt1	-0.17808	9.39E-13	3.03E-08
Irgm2	-0.17882	1.03E-10	3.32E-06
Cd300lf	-0.17902	7.81E-08	0.002521
Xdh	-0.1808	4.25E-16	1.37E-11
Pstpip2	-0.18478	4.74E-09	0.000153
Ddit4	-0.18488	8.5E-27	2.74E-22
Gbp2	-0.18577	1.19E-07	0.003839
Arid5b	-0.18848	1E-07	0.003235
Cd36	-0.18879	1.56E-09	5.05E-05
Rnf213	-0.19225	9.98E-11	3.22E-06
Cxcl10	-0.1944	0.000103	1
Irgm1	-0.19731	1.17E-16	3.78E-12
Ciita	-0.19979	1.43E-20	4.61E-16
Rad51b	-0.20335	1.03E-05	0.331906
Snx30	-0.20961	8.04E-10	2.6E-05
Stat1	-0.21533	2.04E-17	6.57E-13
Aff1	-0.23686	6.97E-15	2.25E-10
Gbp4	-0.24005	5.28E-19	1.7E-14
Csmd3	-0.25138	1.16E-10	3.75E-06
Samhd1	-0.25612	1.86E-16	6.01E-12
Gm4951	-0.25659	9.26E-15	2.99E-10
Gbp8	-0.26499	1.33E-19	4.31E-15
AY036118	-0.27094	5.9E-47	1.9E-42
Cmss1	-0.34057	2.5E-32	8.07E-28
Gbp5	-0.35455	8.07E-26	2.6E-21
Fkbp5	-0.36708	2.35E-13	7.6E-09
Gm42418	-0.37335	1.56E-91	5.04E-87
H2-Eb1	-0.38619	6.49E-20	2.1E-15
Cd74	-0.45547	2.03E-27	6.55E-23
H2-Aa	-0.46031	1.4E-25	4.51E-21
H2-Ab1	-0.46463	6.43E-25	2.08E-20

Iigp1	-0.5506	4.29E-24	1.39E-19

Supplemental Table 5: Flow Cytometry Antibodies and Reagents

Target	Clone	Fluorochrome(s)	Vendor
B220	RA3-6B2	APC	BD Biosciences
CD4	RM4-5	APC	eBioscience
CD4	RM4-5	e450, FITC	BD Biosciences
CD4	GK1.5	PE	BD Biosciences
CD4	GK1.5	SB600	eBioscience
CD8	53-6.7	APC, PE-Cy7, BUV737	BD Biosciences
CD11b	M1/70	e450	eBioscience
CD45.2	104	APC	BD Biosciences
CD45.2	104	BV785	BioLegend
CD80	16-10A1	APC	eBioscience
CD86	GL-1	PE-Cy5.5	BioLegend
F4/80	BM8	PE	eBioscience
GM-CSF	MP1-22E9	PE-Cy7	eBioscience
$H2K^b$	AF6-88.5	FITC, BUV395	BD Biosciences
$H2K^b$	AF6-88.5.5.3	PE	eBioscience
$H2K^d$	SF1-1.1	FITC, PE	BD Biosciences
$H2K^k$	36-7-5	PE	BD Biosciences
IFNγ	XMG1.2	APC	eBioscience
IL-6	MQ2-13A5	e450	eBioscience
MHC Class II	M5/114.15.2	AF700	eBioscience
NK1.1	PK136	PE	BD Biosciences
TCRβ	H57-597	APC	BD Biosciences
TCRβ	H57-597	PE, APC-e780	eBioscience
Tmem119	V3RT1GOsz	PE-Cy7	eBioscience
TNFα	MP6-XT22	PerCP-Cy5.5	BioLegend
LIVE/DEAD	N/A	Aqua	Invitrogen
FC Block	2.4G2	N/A	BD Biosciences

PE=Phycoerythrin, FITC=Fluorescein isothiocyanate, APC=Allophycocyanin, PE-Cy7= Phycoerythrin-Cyanine7, e450=eFlour 450, PerCP-Cy5.5=Peridinin chlorophyll protein-Cyanine5.5, PE-Cy5.5=Phycorythrin-Cyanine5.5, BV=Brilliant Violet, BUV=Brilliant Ultraviolet, AF700=Alexa Fluor 700, eAPC-780=Allophycocyanin-eFluor 780, SB600=Super Bright 600

Supplemental Table 6: RT-qPCR primers (listed 5'-3')

**Target**	**5' primer sequence**	**3' primer sequence**
IFNγ	TCAAGTGGCATAGATGTGGAAGAA	TGGCTCTGCAGGATTTTCATG
TNFα	CTTCTGTCTACTGAACTTCGGG	CAGGCTTGTCACTCGAATTTTG
IL-6	GTTCTCTGGGAAATCGTGGA	TCCAGTTTGGTAGCATCCATC
GM-CSF	TTTACTTTTCCTGGGCAT	TAGCTGGCTGTCATGTTCAA
