# Supplementary figures and images for: Type 2 cannabinoid receptor expression on microglial cells regulates neuroinflammation during graft-versus-host disease
Source: J Clin Invest. 2024 Apr 25;134(11):e175205. doi: 10.1172/JCI175205 (PMC11142740; doi:10.1172/JCI175205)

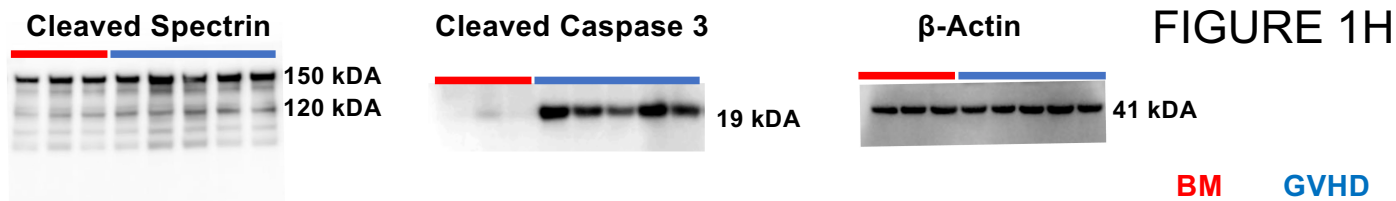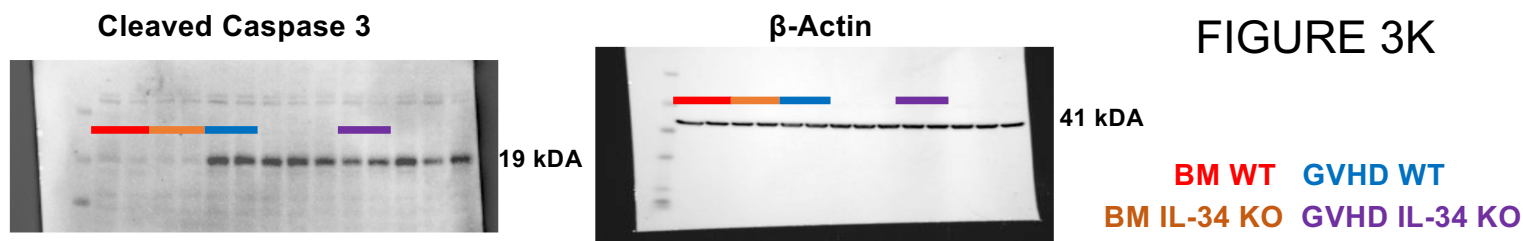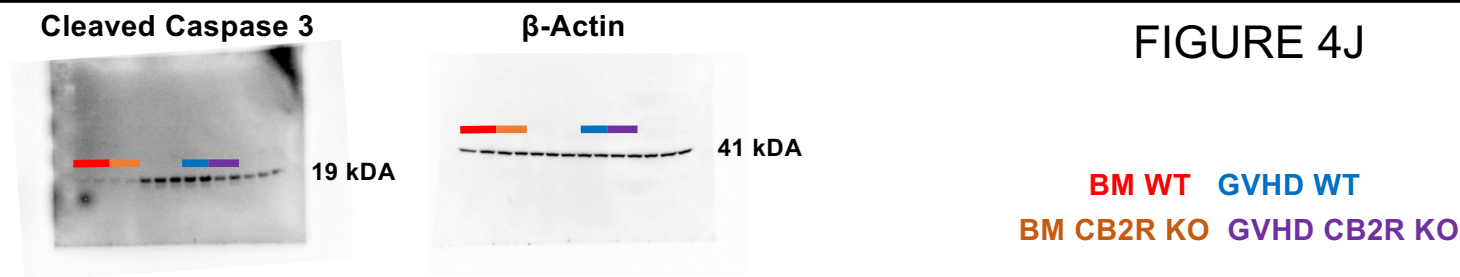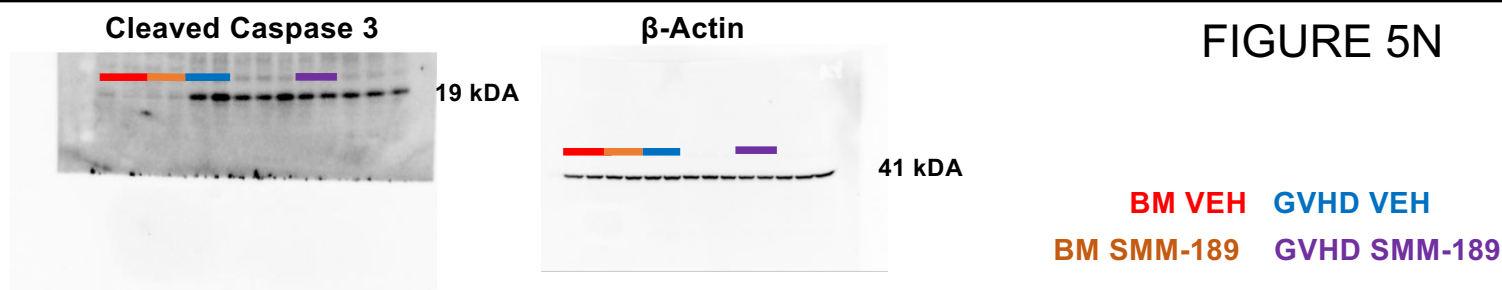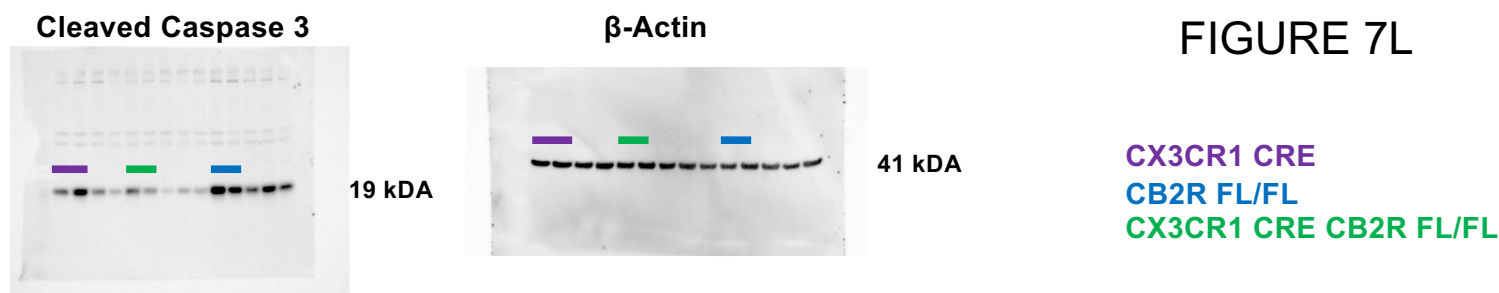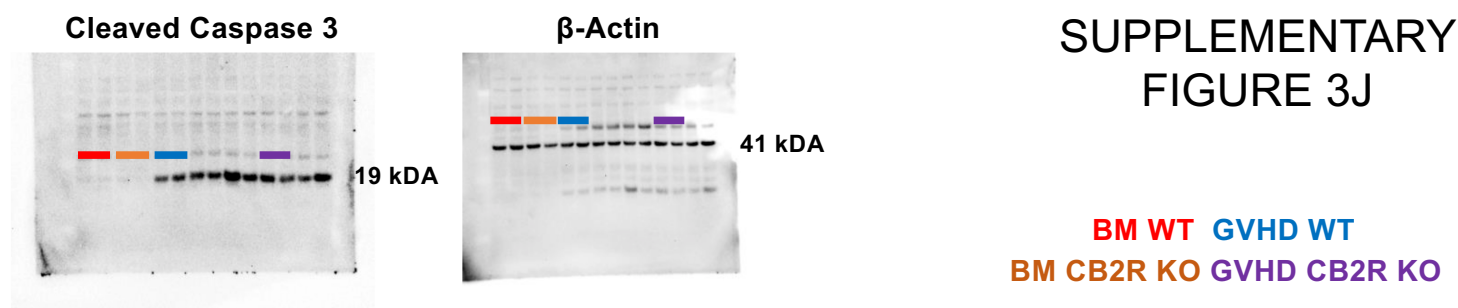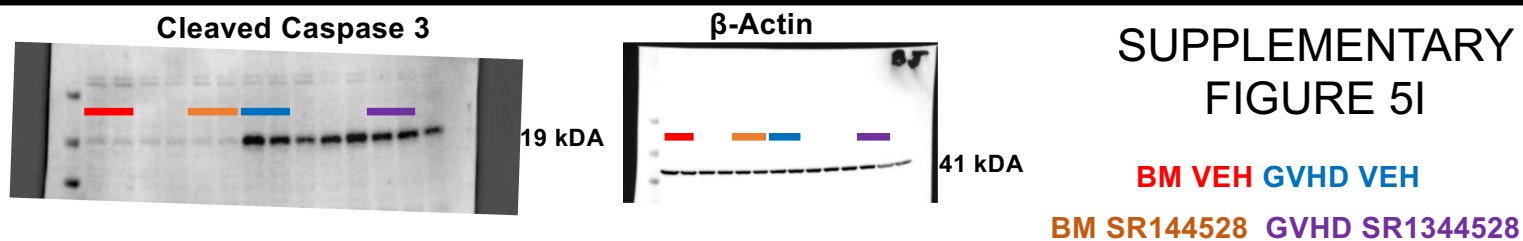

Supplement: Unedited blot and gel images [file jci-134-175205-s202.pdf]
